# Variation in the diversity-productivity relationship in young forests of the eastern United States

**DOI:** 10.1371/journal.pone.0187106

**Published:** 2017-11-15

**Authors:** Santosh Ojha, Luben Dimov

**Affiliations:** Department of Biological and Environmental Sciences, Alabama A&M University, Normal, Alabama, United States of America; Cary Institute of Ecosystem Studies, UNITED STATES

## Abstract

The diversity–productivity relationship has not been studied as extensively in forests as in other ecosystems. We address this gap in our knowledge by examining the relationship of productivity (primarily the periodic annual increment in aboveground biomass, but also the mean annual increment) with five species diversity indices, stand, and environmental factors. We used 967 naturally regenerated Forest Inventory and Analysis plots with stand age ≤30 years, located in the conterminous thirty-one eastern states, and satisfying strict selection requirements. Generally, mixed-species (heterospecific) stands were as productive as or even somewhat more productive than pure (monospecific) stands. The periodic and mean annual increments were both positively correlated with species richness (R^2^ = 0.04 and 0.20, p<0.001). Similarly, the zero-order and partial correlations with productivity were positive for four of the diversity indices (species richness, functional diversity, phylogenetic diversity, and phylogenetic species richness) and not significant for the fifth (functional dispersion). Greater diversity was more important on low-productivity sites and in stands with low stocking. As forests generally get more diverse and productive away from the poles, we tested if the nature of the productivity-diversity relationship changed latitudinally. Productivity was weakly positively correlated with four of the diversity indices north of 40° latitude, but weakly negatively with three of the indices to the south. Our examination of the productivity–diversity relationship in stands containing either of the two most dominant species, quaking aspen or loblolly pine, revealed that pure loblolly pine stands were somewhat more productive than only three of the eight mixtures with loblolly in the composition, while pure aspen stands were no more productive than any of the aspen mixtures. Overall, monospecific stands did not seem to have a clear productivity advantage over mixtures. The findings of this study have implications for woody biomass production, carbon sequestration by forests, and biodiversity conservation.

## Introduction

Diversity–productivity relationships have been studied in various terrestrial and aquatic ecosystems, but the patterns and mechanisms for these relationships are still debated [1–3]. A positive diversity-productivity relationship has been found in manipulative grassland experiments [4–7], while in studies of forest ecosystems, the relationship has been found to be either positive, negative, no relationship, hump-shaped, or U-shaped [8–14]. Positive diversity-productivity relationships for tropical regions and unimodal relationships for temperate regions are commonly reported in the literature [13]. The shape of the diversity productivity relationship varies with spatial scale: unimodal are common at the local to landscape scales, whereas positive linear are found at larger spatial scales [10,15]. A number of factors have been found to influence the diversity-productivity relationship in forest ecosystems: plant density [16,17], site quality [17,18], environment [14,18,19], seed dispersal limitation [20], evolutionary history and latitude [21], successional status [1,9], soil fertility [22,23], and spatial scale [15,24].

Species richness and species composition simultaneously influence ecosystem processes and high-diversity communities can yield greater productivity or carbon storage than the best-chosen monocultures [4]. The mixtures, however, sometimes, do not produce more yield than the monoculture of a highly productive species [25]. There is still a lack of empirical knowledge pertaining to the level of productivity of diverse forests compared to less diverse forests particularly in young and undisturbed natural forest ecosystems for different stand stocking levels, site quality classes, and species functional groups (e.g., based on shade tolerance and taxonomic group).

Although species richness is widely used as a biodiversity metric to predict biomass productivity [17,18,26], functional diversity, which accounts for functional traits of the species, and phylogenetic diversity, which explains evolutionary relationships among species, can also be useful for explaining variation in forest productivity [17,18]. The relationship between species richness and functional diversity is used for exploring the effective dimensionality and complementarity of trait space among species, where increased dimensions increase the importance of species richness and decrease functional redundancy [27]. In some cases, functional diversity has greater explanatory power than species richness [28].There is a need to identify biodiversity metrics for various forest ecosystems to better explain the variation in forest productivity with change in species richness.

We measured productivity as the periodic annual increment (PAI) or the mean annual increment (MAI), which are the average annual increase in stand dry live above-ground biomass over the period since the previous measurement (PAI) or since stand establishment (MAI), respectively. We provide results primarily for PAI, because of low degree of confidence in the stand age estimates and because of potential differences in the growth rates at different ages. However, examining MAI does have some advantages–it expresses the average productivity over the entire life of the stand and takes into account rare events such as pathogen outbreaks, severe droughts, etc. And because all studied stands are relatively young, the differences in rate of growth are not as great as is the case with stands that have much greater age range. Thus, our study examined primarily the relationship between the PAI, but also to an extent the MAI, of the above-ground biomass and the predictors: five diversity indices (species richness, functional diversity, functional dispersion, phylogenetic diversity, and phylogenetic species richness), stand variables (quadratic mean diameter, height, compacted crown ratio, and relative stand density), and environmental variables (precipitation, temperature, slope, aspect, and elevation) for different stand stocking classes, site quality classes, and species functional groups (shade tolerance and major species groups). Additionally, we assessed biodiversity metrics to identify their ability to explain the variation in productivity. We also examined the nature of the species richness-productivity relationship across the stands of the two most dominant species of the study area–quaking aspen (*Populus tremoloides*) and loblolly pine (*Pinus taeda*). Specifically, for different stand stocking classes, site quality classes, and species functional groups, we investigated if 1) species richness has positive association with productivity, 2) species richness has stronger influence than climate, and environmental variables on productivity, and 3) phylogenetic diversity or functional diversity are better than species richness in accounting for the variation in productivity.

## Materials and methods

### Study area

**Fig 1:**
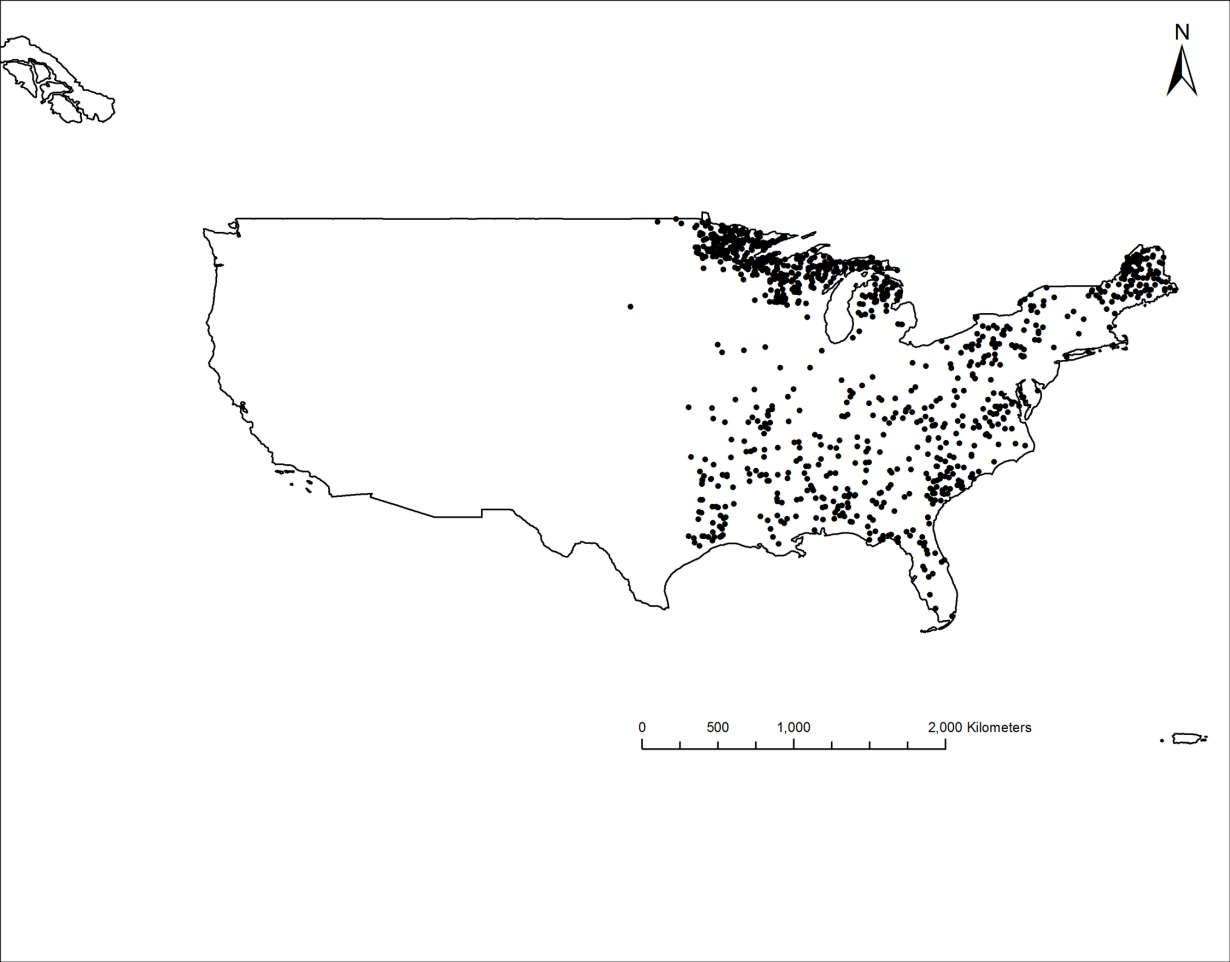
Approximate location of the sample plots in the eastern United States.

The study area consists of the 31 conterminous eastern states of the United States, from North Dakota in the northwest to Texas in the southwest (Fig 1). The climate of the region is classified as temperate continental, humid subtropical, temperate oceanic, warm semi-arid, cold semi-arid, warm continental, temperate continental, and cool continental under the Köppen climate classification [29]. The forests of the eastern region are very diverse in composition and distribution and include the boreal black spruce (*Picea mariana*)–tamarack (*Larix laricina*) forest type, oak-hickory, maple-beech-birch, and the extensive pine forests of the south [30]. The climatic variability and edaphic heterogeneity are among the factors that contribute to high biodiversity in the region.

### Data

We used data from the nationally standardized periodic national forest inventory data from the United States Department of Agriculture (USDA) Forest Service’s Forest Inventory and Analysis (FIA) program. The FIA staff provided us with plots that were remeasured between 1999 and 2014. The time range between measurements of the same plot was 9.3 years, from 1.5 to 10.8 (mean 6.85±0.08 years). For proper comparison of the plot biomass through time, we selected only plots that were established under the new plot design that was in use after 1999, a total of 967 plots. We chose plots with stand age of 30 years or less to reduce the likelihood of harvesting since stand establishment. Additionally, the plots had no visible natural or anthropogenic disturbance since the last measurement, or within the last five years for new plots. According to the FIA procedures, a disturbance must be at least 0.405 ha (1 acre) in size and a significant level of disturbance (mortality or damage to >25% of the trees) needs to have occurred for a plot is to be coded as having experienced disturbance. We used only plots that were classified as forest land with at least 10% cover by trees. The data we used was only from naturally regenerated stands. In summary, we used plots from only naturally regenerated stands, with stand age ≤30 years, with no visible disturbance since the last inventory, with at least 10% tree cover, measured in the specified time period, and sampled using the new FIA plot design. The plots are located in forests managed under various ownership (private, public (both federal and state government ownership), industrial, and others).

The FIA sampling procedures are described in detail in the FIA manuals. In short, the FIA plots are located on a 4.8 km grid and each plot represents about 2,331 hectares [31]. The FIA plot design consists of four circular 7.3 m radius subplots spaced 36.6 m apart in a triangular arrangement with three subplots at each corner and one subplot in the center of the triangle. In each subplot, a circular microplot with 2.07 m radius is nested to measure saplings between 2.54 cm and 12.7 cm in diameter at breast height (dbh, diameter at 1.37 m above the ground). Woodland species with multiple stems are measured at the root collar if their basal diameter is equal to or over 2.54 cm. We used data from both the subplots and the microplots, so our data took into account all stems over 2.54 cm in dbh.

The climate variables used in this study were mean annual precipitation and mean annual temperature. The climate data was extracted from spatial data of 800 m resolution from the database of the PRISM Climate Group [32]. The mean values in the climate data were the 30 year normals (the average) for the period from 1981 to 2010.

### Data analysis

We used the dry live aboveground biomass (AGB) of the live trees, saplings and woodland tree species, which were calculated using published biomass equations [33] and adjustment factors for the tree components as per the FIA protocols [34]. We examined primarily the periodic annual increment (PAI), but also the mean annual increment (MAI) of the live AGB and we treat them as a measure of productivity.

We computed the periodic annual AGB increment (PAI, Mg ha^-1^ yr^-1^) as follows:

$$Y=\frac{\sum_{i=1}^{n}b_{{i(t}_{2})-}\sum_{i=1}^{n}b_{{i(t}_{1})}}{t_{2}-t_{1}}$$ (1)

Where Y is the periodic annual increment (growth) of total AGB of all live trees between the measurements at time t_1_ and t_2_ for a plot, and b is the biomass of the i_th_ tree. In calculating the biomass on the plots during the second period, we included the volume of the newly recruited stems (ingrowth) as it represents a portion of the sequestered carbon over the studied period, which is also harvestable and usable. If a tree died after the first measurement, its live biomass during the second inventory was zero, thus not contributing to the amount of live biomass during the second measurement. We used the data from the first of the two measurements for calculating the values of the predictor variables (e.g., species richness) in the models for PAI.

We calculated the mean annual AGB increment (MAI, Mg ha^-1^ yr^-1^) as follows:

$$Y=\frac{\sum_{i=1}^{n}b_{i(t)}}{t}$$ (2)

Where b is the biomass of i_th_ tree at time t. We obtained MAI by dividing the total dry live AGB of each plot by the mean stand age of that plot.

The quadratic mean diameter (QMD) of each plot was estimated using the basal area of all plot stems that were larger than 2.54 cm in dbh. The mean height of the trees on a plot was calculated as the average height of all stems with dbh of 12.7 cm or more. We had 138 plots (out of 967) with no tree height records). Compacted crown ratio (CCR) is the length of the crown supporting live, healthy foliage divided by the total height of the tree, expressed as percent and measured at the individual tree level [33]. The mean CCR of a plot is the average CCR of all individual trees on the plot. The stand age on the plots was less than 30 years, so all the plots were kept in a single age class in our analysis.

We did a transformation of the response variable as the distribution of the error terms was quite skewed and the variance of the error terms was not constant [35]. As the response variable PAI contained both positive and negative values, we applied log-modulus transformation to retain value-sign and to make the data more normal [36]. In the results we provide the reasons for the observed negative PAI on select plots. The log-modulus transformation was done as follows:

x′=sign(x)*log⁡(|x|+1) (3)

where x’ is the log-modulus transformed value of x. We refer to log-modulus transformed PAI hereafter as “LN PAI”.

The Gaussian distribution of MAI was left skewed, so a square root transformation was applied to improve its normality [37]. We refer to this variable hereafter as “square root MAI”. Beers transformation [38] was applied for aspect transformation by modifying the azimuth from 0–360° to values ranging from 0 to 2. A value of 2 represents the northeast facing slopes (more mesic conditions) and a value of 0 the southwest facing slopes (more xeric conditions). The Beers transformation equation is expressed as follows:

$$A^{\prime }=COS(RADIANS(45-A))+1$$ (4)

Where A’ is transformed aspect value and A is the azimuth in degrees. The azimuth transformation is needed because the minimum and maximum values of the azimuth (0 and 360) actually represent the same direction, north.

The arcsine transformation was applied to slope and CCR [37], as both of them were expressed in percent. The transformed variables were used in bivariate correlation, general linear model, and multiple regression, whereas untransformed variables were used on scatterplots, bar graphs, and relevant tables.

The importance value percent (IVP) of each species was calculated as an average of relative density percent, relative frequency percent, and relative dominance percent that was used to rank the dominance of species across the study area [39].

The relative stand density (RD) of the plots was the ratio between the current stand density index (SDI) and the maximum SDI [40]. The SDI is the relationship between tree density and size in fully stocked pure stands [41]. The formula is also used for unevenaged stands with a slight modification known as the additive or summation method, where individual tree SDIs are added up to obtain the stand SDI [42]:

$$SDI=\sum {tph}_{i}{\left(\frac{{DBH}_{i}}{25}\right)}^{1.6}$$ (5)

Where tph_i_ is number of trees per hectare for the _*i*_th tree in the stand, and DBH_i_ is the diameter of the *i*^th^ tree in the stand (cm). The maximum SDI of the plot was calculated using published regression equation developed for eastern forests [40] that was based on wood specific gravity [43] of the tree species.

We generated stand stocking classes, site quality (also referred to as site productivity) classes, shade tolerance groups, and major species groups as categorical variables. We formed three stand stocking classes that were at similar levels as the “key” SDI values used by others (see Table 1 in [44]): (1) low stocked (relative density less than 0.3), (2) medium stocked (relative density greater than 0.3 and less than 0.6), and (3) high stocked (relative density more than 0.6. We used the FIA’s site productivity (quality) classification, whose classes range from the lowest productivity class 7 to the highest productivity class 1. Our data lacked plots from site productivity class 1, so all plots were grouped into three site productivity classes as: 1) low (FIA site productivity classes 6 and 7 with wood growth potential less than 3.4 m^3^ ha^-1^ yr^-1^), 2) medium (FIA site productivity classes 4 and 5 with wood growth potential between 3.4 and 8.3 m^3^ ha^-1^ yr^-1^), and 3) high (FIA site productivity classes 2 and 3 with wood growth potential greater than 8.3 m^3^ ha^-1^ yr^-1^). We used two functional groups: 1) shade tolerance (trait) and 2) major species group (taxonomic). The major three shade tolerance classes are: shade intolerant, intermediate, and shade tolerant. Plots were classified in three classes for shade tolerance: (1) intolerant—plots had only shade intolerant species, (2) tolerant—plots had only shade tolerant species, and (3) mixed—plots comprised both shade intolerant and tolerant species. There were very few plots of pure intermediate species, so we did not create an intermediate shade tolerance class. The tree species are broadly classified into four major groups: pines, other softwoods, soft hardwoods, and hard hardwoods [33]. Based on this, we used three classes: (1) conifer/pine (plots comprising pines and other softwoods), (2) hardwoods (plots comprising soft hardwoods and hard hardwoods), and (3) mixed (plots comprising pines, other softwoods, and either soft hardwoods, hard hardwoods, or both hardwood types).

We used the Tukey-Kramer procedure for pairwise comparisons between PAI means for stand stocking classes, site productivity classes, shade tolerance classes, major species groups, and for species richness levels as it produces narrow confidence intervals and is more conservative, robust, and powerful [45]. However, if the assumption of homogeneity of variances is not satisfied, the Tukey-Kramer procedure could produce false results [45]. Therefore, we used Satterthwaite/Kenward-Roger approximation for the degrees of freedom to solve the problem of the unequal sample sizes (different number of plots in the different stocking levels, productivity classes, etc.) and variance heterogeneity of the groups [46]. The Kenward-Roger method also adjusts the covariance matrix with a small-sample bias, when one of the groups had too small a sample size compared to the others [46]. The adjustments take into account the denominator degrees of freedom that are not constant across estimates.

The species richness-productivity relationship was also assessed across the plots containing the two most dominant tree species, quaking aspen (*Populus tremuloides* Michx.) and loblolly pine (*Pinus taeda* L.) to check whether the species richness-productivity relationships differ significantly between stands containing either of these common species.

We calculated the Pearson bivariate correlation between productivity and the explanatory variables and used general linear model (GLM) procedures to study the relationship between productivity and species richness.

The stepwise multiple linear regression was applied between the response variable productivity and the predictor variables across stand stocking classes, site productivity classes, shade tolerance groups, and major species groups. The procedure was applied to investigate the relative importance of species richness among the predictors. The two best fitted multiple regression models for each relationship were selected among a set of a priori candidate models. The criteria for model selection were (1) the model with lowest Akaike information criteria (AIC), and (2) alternate best model with fewer predictors. The alternate model was produced to see whether species richness still remained in the model with fewer predictors. The equation of the multiple linear regression model [47] was as follows:

$$\hat{P}=b_{0}+b_{1}QMD+b_{2}HT+b_{3}CCR+b_{4}SPR+b_{5}SL+b_{6}AS+b_{7}PPT+b_{8}TEMP+b_{9}ELEV$$ (6)

where $\hat{P}$ is the predicted or expected value of productivity. QMD is quadratic mean diameter, HT is mean height, CCR is compacted crown ratio, SPR is species richness, SL is slope, AS is aspect, PPT is mean precipitation, TEMP is mean temperature, ELEV is elevation, b_0_ is the intercept, and b_1_ through b_9_ are regression coefficients.

We also calculated standardized beta coefficient of the multiple regression models to compare the relative strength of the effect of each individual independent variable. The coefficients were obtained by converting the regression variables to z-scores before running the analysis.

### Diversity Indices

We used species richness (a count of the number of species), two functional diversity indices (functional diversity and functional dispersion), and two phylogenetic diversity indices (phylogenetic diversity and phylogenetic species richness) as measures of diversity.

To compute functional diversity, a careful selection of functional traits is essential [27,48]. We selected species functional traits that were related to species’ silvicultural and phenological characteristics, biomass and growth, and response to climate and environmental conditions. The data on 15 different traits of all the 147 tree and shrub species were collected from the literature and the online plant database of the United States Department of Agriculture [33,43,49,50] as either continuous or categorical, as follows. The four important functional traits were continuous: wood specific gravity, maximum height at maturity, root length, and seed mass, were collected as continuous data. The other 11 traits were used as categorical data: leaf type, CN ratio, coppice potential, growth rate, known allelopath, nitrogen fixation, tree crown shape and orientation, drought tolerance, salinity tolerance, seed spread rate, and shade tolerance. The selected species functional traits are important and commonly used for estimation of forest ecosystem functional diversity [51,52].

We computed two functional diversity indices for each plot from the trait values—plot based functional diversity (FD) [27,51–53] and functional dispersion (FDis) [51,54]. Only four functional traits were used for calculating FD, while all fifteen functional traits were used for calculating FDis. The FD index was the total branch length of the functional dendrogram that was generated by the species functional traits. FD was generated by the cumulative effects of species richness, number of functional groups, community composition, and species identity [27] and was calculated using Euclidean distance in average linkage algorithm [52]. The FDis index was estimated from multi-trait dispersion and is a multidimensional index [51]. FDis was the average distance of each species to the community trait centroid of all species, where relative abundances of the species were taken into account [51,54]. Because of the mixture of both quantitative and qualitative traits, FDis was computed using Gower distance in average linkage algorithm [52].

Faith’s phylogenetic diversity (PD) was estimated for each plot based on cladistics/phylogenetic relationships among the taxa that takes into account the phylogenetic differences among species [55]. PD was the sum of the branch lengths of a phylogenetic tree linking all species. Phylogenetic species richness (PSR) is the measure of the evolutionary relatedness between the species in the community. PSR measures richness of a community where phylogenetically diverse species add greater biodiversity than phylogenetically related species [56].

As forests further from the poles are generally more diverse and more productive, we examined the nature of the productivity-diversity relationship in the northern and the southern forests by dividing them into two groups–those located north or south of 40° latitude. Northern plots were predominantly represented by aspen-birch, maple-beech-birch, and spruce-fir forest type, while southern plots were represented predominantly by loblolly-shortleaf pine, longleaf-slash pine, and oak-pine forest type.

Partial correlation was applied to measure degree of association between productivity and the diversity indices while controlling climatic and environmental variables across different stand stocking classes, site productivity classes, shade tolerance classes, and major species groups.

The statistical packages IBM SPSS^®^ 21 and SAS^®^ 9.3 were used for data analysis and statistical inferences. SAS^®^ PROC mixed procedure with multiplicity adjustment for Tukey was used for pairwise comparison of the means. Species IVP and tree community analysis were done using PC-ORD^®^ Version 6.12. Species richness, the two functional diversity and two phylogenetic diversity indices were all computed using the software Fdiversity [57]. The full dataset, species richness traits, metadata, the FAI database description and user manual, and a research note concerning wood and bark specific gravity are provided as Supplementary Information in files S1 File through S5 File.

## Results

A total of 147 tree and shrub species were present across the 967 plots at the time of the earlier of the two measurements. Plot-level species richness ranged from 1 to 15. The PAI and MAI ranged from -6.78 to 21.91 Mg ha^-1^ and 0.01 to 15.04 Mg ha^-1^ yr^-1^, respectively (Table 1). We observed a negative PAI (average of 12.9% reduction in biomass), all due to natural mortality, in 41 of the plots. The period between FIA re-measurements of these particular plots ranged from 2.5 to 10.8 years, mean 6.0 years. The greatest biomass reduction in the plots with negative PAI was due to mortality of quaking aspen or green ash (*Fraxinus pennsylvanica* Marshall), which were also the two species with the greatest and second greatest initial biomass, respectively, in the plots with negative PAI. Aspen experienced high rates of mortality during the studied period due to record droughts or pathogens throughout the continent [58,59], including in the eastern US [60,61]. The non-native invasive insect emerald ash borer (*Agrilus planipennis* Fairmaire) has widely infested and has been killing trees of all ash species (*Fraxinus spp*.*)* in the eastern US since 2002 [62]. Green ash was much more prevalent on the 41 plots with negative PAI, where it was the 2^nd^ most common species by biomass, than on the remaining plots, where it was the 18^th^ most dominant species. We used plots with both positive and negative PAI values in the models.

**Table 1:** Mean, standard deviation, minimum and maximum values of the variables across the plots in young forests in the eastern US.

Variables	Mean	SD	Min	Max
**Diameter at breast height (dbh)**^**1**^ **(cm)**	7.5	4.11	2.5	43.54
**Basal area (m**^**2**^ **ha**^**-1**^**)**	11.65	8.59	0.09	46.87
**Height (m)**	11.5	3.60	2.9	27.6
**Density (stems ha**^**-1**^**)**	3377	2705.28	15	16480
**Relative stand density**	0.23	0.16	0	0.92
**Compacted crown ratio (percent)**	0.44	0.17	0	0.99
**Stand age (yr)**	14.3	5.01	3	30
**Total AGB (Mg ha**^**-1**^**)**	33.13	30.15	0.06	246.84
**Periodic annual AGB increment (PAI, Mg ha**^**-1**^ **yr**^**-1**^**)**	3.19	2.76	-6.78	21.91
**Mean annual AGB increment (MAI, Mg ha**^**-1**^ **yr**^**-1**^**)**	2.3	1.94	0.01	15.04
**Plot species richness (SPR)**	4.6	2.98	1	15
**Functional diversity (FD)**	9.7	6.05	0	30.96
**Functional dispersion (Fdis)**	1.5	0.82	0	3.51
**Phylogenetic diversity (PD)**	16.1	7.28	2	38
**Phylogenetic species richness (PSR)**	2.7	1.43	0.22	9.50
**Precipitation (cm)**	102	25.7	51	166
**Mean temperature (**°**C)**	9.3	5.73	2.5	24.4
**Slope (percent)**	6.3	9.56	0	71
**Elevation (m)**	301	179.59	0	1109

^1^ Mean dbh refers to the quadratic mean diameter

**Fig 2:**
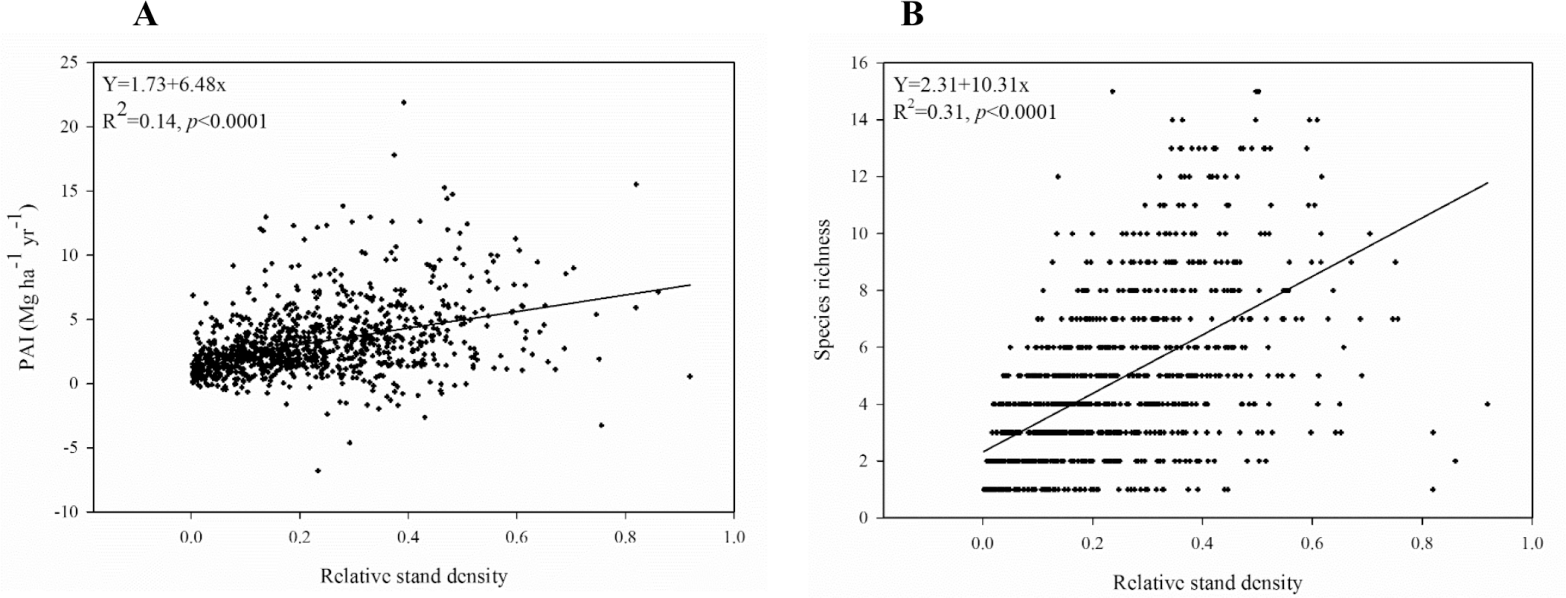
Scatterplots between the variables relative stand density and A) PAI and B) species richness across 967 FIA plots in young forests of the eastern US.

Based on the importance value percent (IVP), the three most dominant species in descending order were quaking aspen (*Populus tremoloides* Michx.), loblolly pine (*Pinus taeda* L.), and red maple (*Acer rubrum* L.) (S1 Table, which also shows the IVP of all species). Plots with three species were the most common (152 plots), while plots with 15 species occurred the least frequently (4 plots) (S1 Fig). The correlation of LN PAI with relative density and species richness was positive (S2 Table, Fig 2A). Among the climate and environmental variables, precipitation, temperature, and elevation were the ones correlated with LN PAI (S2 Table). Species richness had a positive relationship with relative stand density, although the association was weak (Fig 2B).

**Fig 3:**
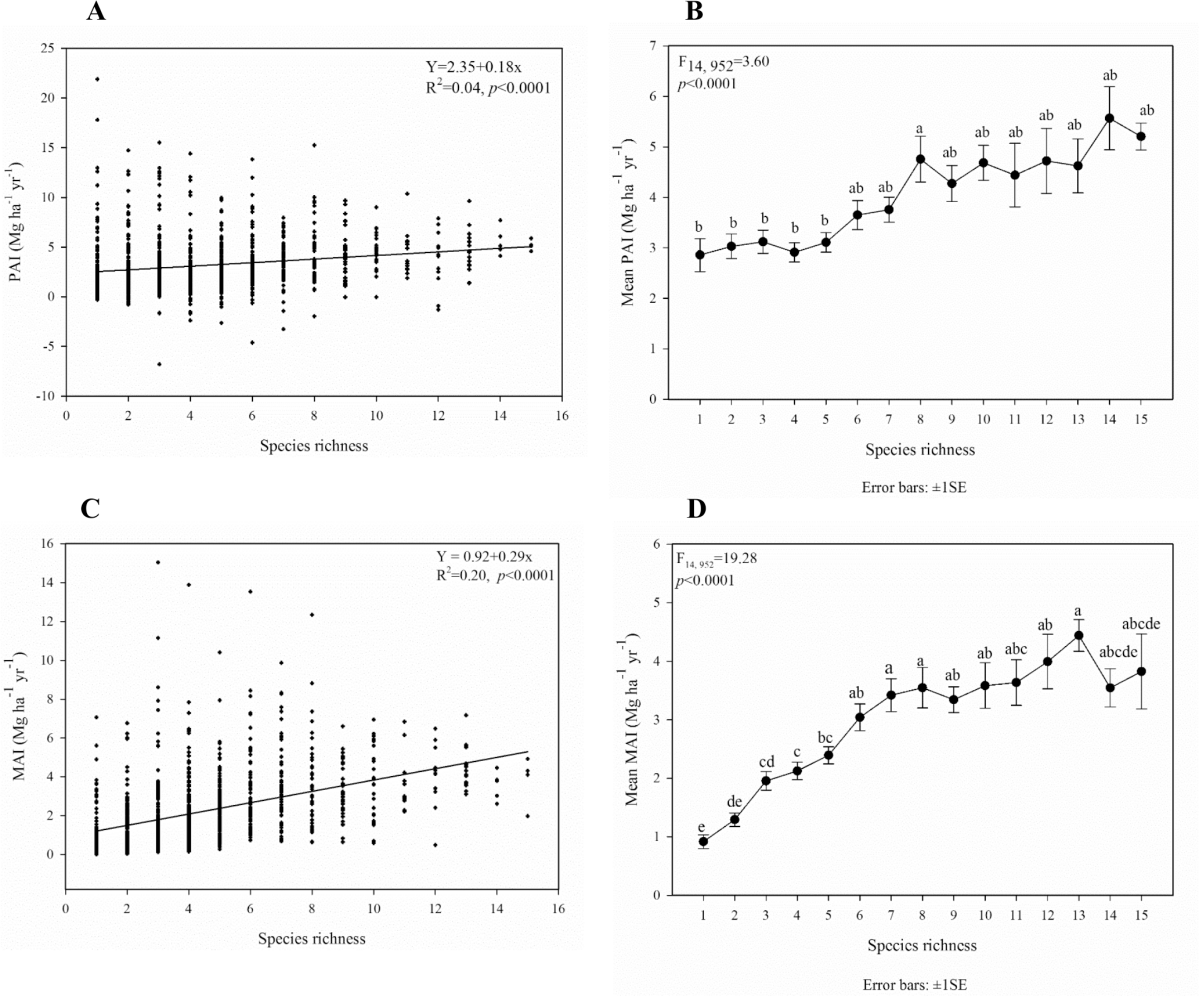
Relationships between A) PAI and species richness, B) mean PAI and species richness, C) MAI and species richness, and D) mean MAI and species richness. Multiple comparisons (Tukey-Kramer test) were performed between PAI LS-means/ MAI LS-means of species richness levels.

PAI had a positive but very weak linear relationship with tree species richness (R^2^ = 0.04, F_14, 952_ = 3.60, *p*<0.0001; Fig 3A and 3B). The plots of species richness 8 had greater mean PAI than plots of richness levels 1 to 5. Based on the mean PAI, the plots with species richness 1 to 7 and 9 to 15 were equally productive. Similarly, MAI exhibited a positive, but stronger, linear relationship with species richness (R^2^ = 0.20, *p*<0.001; Fig 3C). Although the mean MAI was different for the different species richness levels (F_14, 952_ = 19.28, *p*<0.0001), the MAI of the two highest diversity plots, with species richness 14 and 15, was not significantly different from the MAI of low diversity plots (Fig 3D), but the number of plots with 14 and 15 species in them was rather small (S1 Fig), and their standard error was fairly large. Additionally, the plots with species richness of 6 to 15 species were all equally productive (Fig 3D). MAI in the pure and mixed stands was significantly different at some of the diversity levels–for instance, MAI increased significantly with the increase in species richness from 1, to 3, and to 6 species (Fig 3D).

### Species richness-productivity relationships across the stands of the two most dominant tree species

Quaking aspen and loblolly pine were present on 363 and 137 plots, respectively. The species richness ranged from 1 to 12 species in the aspen plots and 1 to 15 species in the loblolly pine plots. Aspen and loblolly pine were in monospecific stands on 23 and 10 plots, respectively. There were relatively few plots with pine or aspen where species richness was more than 9. Therefore, we used plots with species richness up to 9 for both species when analyzing the diversity-productivity relationships.

**Fig 4:**
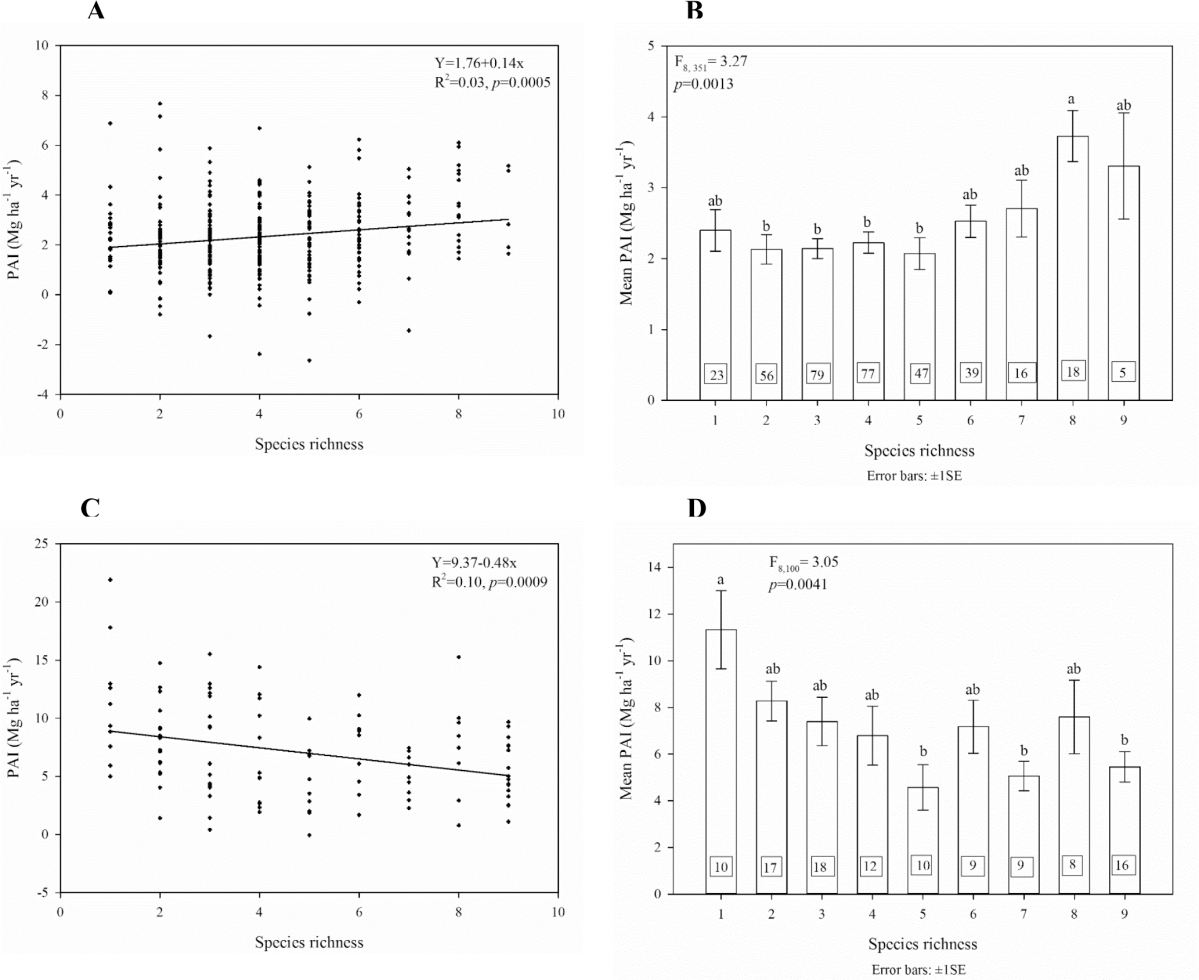
Relationship between PAI and species richness for quaking aspen (A and B) across 360 plots, and loblolly pine (C and D) across 109 plots. Multiple comparisons (Tukey-Kramer test) were done between the PAI means of the species richness levels.

For quaking aspen stands, species richness had a positive relationship with PAI, although the relationship was very weak (R^2^ = 0.03, *p* = 0.0005) (Fig 4A). The multiple comparisons between the PAI means revealed greater mean PAI in plots with species richness 8 than plots with species richness 2 to 5 (F_8, 351_ = 3.27, *p* = 0.0013; Fig 4B). All aspen mixtures were as productive as the pure aspen stands. For loblolly pine however, the linear relationship between species richness and PAI was negative and weak (R^2^ = 0.10, *p* = 0.0009) (Fig 4C). In loblolly pine stands, greater species richness did not generally correspond to neither greater nor smaller PAI, except that the pure loblolly pine stands were more productive than loblolly pine stands with species richness 5, 7 or 9 (F_8, 100_ = 3.05, *p* = 0.0041; Fig 4D). Even when temperature, precipitation, slope, aspect and elevation were controlled, the partial correlation analysis had the same result—a positive but weak correlation between species richness and PAI in quaking aspen stands (r = 0.12, *p* = 0.03), but a negative, though also weak, correlation for loblolly pine forests (r = -0.31, *p* = 0.001).

### Effect of stand stocking, site productivity, and species functional groups on the species richness-productivity relationship

As could be expected, PAI was greater in the stands with greater stand stocking. The range of species richness was almost the same in the three stocking classes and was up to 15 species per plot (Table 2). Species richness had a positive correlation with LN PAI in low stocked stands, although the association was not strong. Species richness was not significantly correlated with LN PAI in medium and high stocked stands (Table 2).

**Table 2:** The GLM for LN PAI and species richness (SPR) by different classes of stand stocking, site productivity, shade tolerance, and major species groups. Different superscripts within a class/group indicate significant differences at α = 0.05 (Tukey-Kramer multiple comparison test). The range is shown in parenthesis.

	Classification		AGBG		SPR		GLM LN^1^(PAI) = f(SPR)		
		Plots	LS-Mean	SE	Mean	SE	R^2^	*F*	*p*
**Stand stocking classes**									
** **	**Low stocked**	681	2.66^b^ (-6.78, 13.84)	0.08	3.68 (1, 15)	0.08	0.05	2.90	0.0006
	**Medium stocked**	262	4.42^a^ (-2.64, 21.91)	0.21	6.96 (1, 15)	0.21	0.05	1.01	0.4487
	**High stocked**	24	4.74^a^ (-3.26, 15.52)	0.78	6.42 (1, 14)	0.67	0.42	0.79	0.6522
**Site productivity classes**									
** **	**Low**	319	2.46^c^ (-6.78, 8.82)	0.11	3.80 (1, 14)	0.13	0.13	4.12	<0.0001
	**Medium**	589	3.42^b^ (-3.26, 21.91)	0.12	4.88 (1, 15)	0.12	0.04	1.89	0.0251
	**High**	59	4.78^a^ (-1.02, 12.16)	0.40	6.71 (1, 15)	0.55	0.16	0.60	0.8538
**Shade tolerance classes**									
** **	**Intolerant**	191	3.16^a^ (-1.52, 21.91)	0.25	1.79 (1, 6)	0.07	0.03	0.97	0.4348
	**Tolerant**	35	1.52^b^ (-0.08, 6.54)	0.22	1.83 (1, 5)	0.18	0.06	0.49	0.7399
	**Mixed**	733	3.28^a^ (-6.78, 15.52)	0.09	5.55 (2, 15)	0.10	0.07	4.16	<0.0001
**Major species group**									
** **	**Conifer/pine**	62	4.58^a^ (-0.30, 21.91)	0.57	1.37 (1, 4)	0.09	0.01	0.28	0.8362
	**Hardwoods**	439	2.47^b^ (-6.78, 11.29)	0.10	3.89 (1, 14)	0.12	0.12	4.52	<0.0001
	**Mixed**	466	3.68^a^ (-3.26, 15.52)	0.13	5.78 (2, 15)	0.14	0.06	2.38	0.0043

^1^LN refers to the Log-modulus transformation; PAI stands for “periodic annual increment in aboveground biomass (Mg ha^-1^ yr^-1^)”; f(SPR) stands for “function of species richness”.

Not surprisingly, PAI also increased with the increase in site productivity. The range of species richness in the three site productivity classes was very similar. The association between species richness and LN PAI was positive on low and medium site productivity sites, but not significant on high-productivity sites (Table 2).

There was no difference between the PAI on plots with only intolerant and plots with mixtures of intolerant and tolerant species (Table 2). Species richness was lower in the plots with only intolerant and the plots with only tolerant species (maximum richness of up to 6 and 5 species, respectively) than plots with mixed species (maximum richness of 15 species). Species richness had a positive relationship with LN PAI in stands containing a mix of tolerant and intolerant species.

The PAI of the stands containing only conifer/pine species and of the mixed (conifer/pine-hardwood) stands were not significantly different from each other (Table 2). Both types were in turn more productive than hardwoods-only stands. Species richness had no significant relationship with LN PAI in conifer/pine stands, where species richness was very low, ranging from 1 to just 4. However, species richness did have a positive association with LN PAI in both of the other groups–hardwoods-only and mixed pine-hardwood stands, both of which also had high species diversity (richness up to 15 species).

Similarly, MAI also increased with increasing stand stocking and site productivity (S3 Table). The association between square root MAI and species richness was significant and fairly strong across the plots with low stand stocking and low site productivity. Although square root MAI and species richness had a significant positive relationship across all three shade tolerance classes, the association was strongest in the tolerant stands. The square root MAI–species richness relationship was positive in hardwood and mixed conifer/pine-hardwood stands, but not significant in conifer/pine stands (S3 Table). When compared with PAI, the MAI had stronger association with species richness across all classes.

### Multiple linear regression models for productivity

The best multiple linear regression models explained between 11% and 42% of the variation in LN PAI (and even greater 29–69% in square root MAI) across the different classes of stand stocking, site productivity, shade tolerance, and major species group (S4 and S5 Tables, Table 3). In general, the most contributing variables, according to the absolute value of the standardized regression coefficients, were temperature, precipitation, and elevation, followed by quadratic mean diameter, height, compacted crown ratio, and species richness, while slope and aspect were not significant predictors in any models. The environmental variables contributed more than the stand variables in the models. Species richness was a significant predictor of productivity in low stocked stands, low productive sites, mixtures of intolerant and tolerant species, hardwoods, and mixtures of conifer and hardwood (S4 Table, Table 3).

**Table 3:** Best multiple regression predictive models for LN PAI by stand stocking class, site productivity class, shade tolerance class, and major species groups across the 829 FIA plots in young forests in the eastern US. The models with the lowest Akaike's Information Criterion (AIC) were selected from eight priori candidate models of each classification. Bold values are significant at α = 0.05.

Classification			Intercept	QMD	HT	CCR	SPR	SL	AS	PPT	TEMP	ELEV	EDF	Adj-R^2^	AIC	*p*
**All plots**		Model with lowest AIC	**0.582**	**-0.019**	**0.021**	**0.389**	**0.020**	.	.	**0.002**	**0.018**	.	822	0.14	-1429.43	<0.0001
		Alternate best model with fewer predictors	**1.017**	**-0.016**	**0.013**	.	**0.020**	.	.	.	**0.024**	.	824	0.12	-1413.73	<0.0001
**Stand stocking class**																
	**Low stocked**	Model with lowest AIC	**0.561**	**-0.019**	**0.019**	**0.498**	**0.029**	.	.	**0.002**	**0.012**	.	541	0.13	-1062.17	<0.0001
		Alternate best model with fewer predictors	**0.957**	**-0.015**	.	**0.378**	**0.037**	.	.	.	**0.014**	.	543	0.11	-1051.47	<0.0001
	**Medium stocked**	Model with lowest AIC	**0.677**	.	.	**0.852**	.	.	.	0.003	**0.017**	.	253	0.13	-389.62	<0.0001
		Alternate best model with fewer predictors	**0.870**	.	.	**0.895**	.	.	.	.	**0.027**	.	254	0.12	-389.29	<0.0001
	**High stocked**	Model with lowest AIC	**3.807**	**-0.215**	.	.	.	.	-0.295	.	.	**-0.0014**	20	0.22	-29.27	0.04
		Alternate best model with fewer predictors	-Not	found-												
**Site productivity class**																
	**Low**	Model with lowest AIC	**0.721**	**-0.025**	**0.015**	**0.518**	**0.056**	-0.091	.	.	.	**0.0004**	256	0.22	-504.45	<0.0001
		Alternate best model with fewer predictors	**0.888**	**-0.023**	.	**0.409**	**0.058**	.	.	.	.	**0.0003**	258	0.20	-501.93	<0.0001
	**Medium**	Model with lowest AIC	**0.668**	-0.010	**0.016**	0.276	.	.	.	**0.003**	**0.017**	.	501	0.12	-857.64	<0.0001
		Alternate best model with fewer predictors	**0.681**	.	0.012	0.224	.	.	.	**0.003**	**0.015**	.	502	0.11	-856.43	<0.0001
	**High**	Model with lowest AIC	**1.046**	.	**0.037**	.	.	.	.	.	0.021	**-0.0011**	55	0.39	-128.88	<0.0001
		Alternate best model with fewer predictors	**1.516**	.	**0.030**	.	.	.	.	.	.	**-0.0016**	56	0.37	-128.34	<0.0001
**Shade tolerance class**																
	**Intolerant**	Model with lowest AIC	**1.157**	**-0.016**	0.022	.	.	.	.	-0.006	**0.069**	.	114	0.26	-181.75	<0.0001
		Alternate best model with fewer predictors	**1.450**	-0.013	.	.	.	.	.	**-0.007**	**0.070**	.	115	0.25	-181.27	<0.0001
	**Tolerant**	Model with lowest AIC	-1.559	.	0.050	1.106	.	.	.	**0.017**	-0.030	.	21	0.30	-53.04	0.01
		Alternate best model with fewer predictors	-1.540	.	**0.064**	**1.326**	.	.	.	**0.012**	.	.	22	0.25	-51.80	0.02
	**Mixed**	Model with lowest AIC	**0.586**	**-0.027**	**0.021**	**0.392**	**0.023**	.	.	**0.003**	**0.014**	.	670	0.14	-1217.76	<0.0001
		Alternate best model with fewer predictors	**0.926**	**-0.017**	.	.	**0.025**	.	.	**0.004**	.	.	673	0.12	-1202.81	<0.0001
**Major species groups**																
	**Conifer/pine**	Model with lowest AIC	0.182	.	**0.072**	**0.898**	.	.	-0.229	**0.008**	.	**-0.0018**	42	0.42	-74.69	<0.0001
		Alternate best model with fewer predictors	-0.584	.	**0.072**	**0.991**	.	.	.	.	**0.074**	.	44	0.37	-73.04	<0.0001
	**Hardwoods**	Model with lowest AIC	**0.528**	**-0.025**	**0.019**	.	**0.052**	.	0.076	**0.005**	**-0.015**	.	334	0.20	-639.03	<0.0001
		Alternate best model with fewer predictors	**0.706**	**-0.026**	**0.024**	.	**0.042**	.	.	**0.003**	.	.	336	0.19	-636.27	<0.0001
	**Mixed**	Model with lowest AIC	**0.660**	**-0.017**	**0.019**	**0.393**	.	.	.	**0.003**	**0.025**	.	434	0.16	-809.68	<0.0001
		Alternate best model with fewer predictors	**0.954**	.	.	**0.335**	**0.015**	.	.	.	**0.025**	.	436	0.15	-806.20	<0.0001

Where LN = Log-modulus transformation; PAI = periodic annual increment in aboveground biomass (Mg ha^-1^ yr^-1^); QMD = Quadratic mean diameter (cm); HT = average height (m); CCR = Compacted crown ratio; SPR = Species richness; SL = Slope (arcsine transformed); AS = Aspect (Beers transformed); PPT = Mean precipitation (cm); TEMP = Mean temperature (°C); ELEV = Elevation (m); EDF = Error degrees of freedom; *p* = statistical significance value. For multiple regression analysis, we excluded 138 (out of 967) plots because of no height data in those plots.

Based on the magnitude of the standardized coefficients, species richness had stronger relationship with LN PAI than did the climate and environmental factors on low productivity sites and in stands composed of only hardwood species (S4 Table).

When we used square root MAI as response variable, species richness was a significant predictor in nearly all models (S5 Table). It was not, however, in the models for medium and high stocked stands, high productivity sites, and stands of only conifer species in the composition. The parameter estimate for species richness was positive in all models where richness was a significant predictor.

The increase in species richness corresponded to an increase in square root MAI in all cases except in medium and high stocked stands, on high productivity sites, and in stands of only pines/conifers in the composition, where richness was not a significant predictor (S5 Table). But in no models did greater species richness correlate with a decrease in productivity. Based on the magnitude of the standardized parameter estimates, species richness had stronger positive relationship with square root MAI than did climate and environmental factors in low stocking stands, low and medium productivity sites, in stands with only shade tolerant species, and stands with only hardwood species (S5 Table). The variables slope, aspect, precipitation and elevation were less important variables in comparison to other predictors.

### Correlation between species richness, functional diversity, phylogenetic diversity, and productivity

The zero-order and partial correlation between LN PAI and four of the five diversity indices (species richness, functional diversity, Faith’s phylogenetic diversity, and phylogenetic species richness, but not functional dispersion) across the plots indicated a positive correlation between productivity and diversity (Table 4). Among the five studied diversity indices, species richness and phylogenetic species richness had the strongest correlation with productivity. The bivariate correlations between LN PAI and the diversity indices were positive in low stand stocking and low site productivity classes with or without controlling the climatic and environmental variables (Table 4). Productivity had greater correlation with species richness and functional diversity than with other diversity indices in low stocked stands and on low productive sites when controlling the climatic and environmental variables. Functional dispersion had negative association with productivity in medium stocked stands and medium productivity sites. Overall, the correlation with the indices weakened with the increase in stand stocking and site productivity. In the stands with mixtures of tolerant and intolerant species and stands with only hardwoods, productivity had a positive correlation with the diversity indices with or without controlling of climatic and environmental variables (except the significant negative correlation with functional dispersion in mixed tolerant-intolerant stands; Table 4). Species richness and phylogenetic species richness were important biodiversity metrics in plots with mixtures of intolerant and tolerant species and plots with only hardwoods. The correlations between productivity and the studied diversity indices were non-significant in conifer/pine forests. However, the correlations became significant for these same conifer/pine forests when climatic and environmental variables were controlled (Table 4). Contrary to this, there was no significant correlation between productivity and species richness in conifer/pine-hardwood mixed stands when climatic and environmental variables were controlled.

**Table 4:** Zero-order and partial correlation between LN PAI and diversity indices by stand stocking classes, site productivity classes, shade tolerance classes, and major species groups across the 967 FIA plots in forests of the eastern US. Bold values are significant at α = 0.05.

		Zero-order						Partial					
								Controlled variables (AS, SL, PPT, TEMP, ELEV)					
Classification		Plots	SPR	FD	FDis	PD	PSR	DF	SPR	FD	FDis	PD	PSR
**All plots**		965	**0.25**	**0.14**	0.02	**0.13**	**0.25**	960	**0.17**	**0.12**	0.01	**0.09**	**0.17**
**Stand stocking class**													
	**Low stocked**	679	**0.21**	**0.14**	**0.08**	**0.09**	**0.17**	674	**0.19**	**0.15**	**0.08**	**0.08**	**0.14**
	**Medium stocked**	260	0.09	-0.09	**-0.25**	-0.02	**0.21**	255	-0.04	-0.12	**-0.26**	-0.06	0.12
	**High stocked**	22	-0.06	-0.20	-0.45	-0.23	0.12	17	-0.09	-0.16	-0.41	-0.2	0.12
**Site productivity classes**													
	**Low**	317	**0.33**	**0.29**	**0.22**	**0.24**	**0.20**	312	**0.33**	**0.27**	**0.21**	**0.21**	**0.19**
	**Medium**	587	**0.15**	0.01	**-0.10**	0.02	**0.22**	582	0.04	-0.02	**-0.12**	-0.03	**0.12**
	**High**	57	**0.29**	0.19	-0.05	0.26	**0.31**	52	0.24	0.23	-0.01	0.17	0.29
**Shade tolerance classes**													
	**Intolerant**	189	0.11	-0.06	-0.07	**-0.22**	0.12	184	0.11	-0.01	-0.05	-0.11	0.02
	**Tolerant**	33	0.17	0.11	-0.03	-0.02	0.15	28	0.11	0.01	-0.08	0.11	0.46
	**Mixed**	731	**0.25**	**0.12**	**-0.08**	**0.15**	**0.25**	726	**0.15**	**0.08**	**-0.10**	**0.09**	**0.18**
**Major species group**													
	**Conifer/pine**	60	0.11	0.13	0.04	0.23	-0.27	55	**0.27**	**0.36**	0.23	**0.31**	-
	**Hardwoods**	437	**0.31**	**0.25**	**0.18**	**0.27**	**0.27**	432	**0.32**	**0.26**	**0.17**	**0.26**	**0.26**
	**Mixed**	464	**0.19**	0.01	**-0.22**	0.07	0.18	459	0.06	0.00	**-0.19**	0.02	0.08

Where LN = Log-modulus transformation; PAI = Periodic annual increment in aboveground biomass (Mg ha^-1^ yr^-1^); DF = Degrees of freedom; SPR = species richness; FD = functional diversity; FDis = functional dispersion; PD = Faith’s phylogenetic diversity; PSR = Phylogenetic species richness; AS = Aspect; SL = Slope; PPT = Mean precipitation; TEMP = Mean temperature; ELEV = Elevation

The number of plots per unit of land area was greater for the northern region (north of 40° latitude) than the south. The two geographic areas also represented different forest types, climate, and environmental conditions. With controlled stand density, climate, and environmental variables, the correlation between species richness and LN PAI was positive for the plots north of 40° latitude (r = 0.16, *p*<0.001, n = 610), but there was no relationship between the variables for the plots south of 40° latitude (r = -0.07, *p* = 0.23, n = 341). The functional diversity (r = 0.10, *p* = 0.013, n = 610), functional dispersion (r = 0.12, *p* = 0.004, n = 610), and Faith’s phylogenetic diversity (r = 0.08, *p* = 0.042, n = 603) similarly had positive partial correlation with LN PAI for the northern plots, but had negative correlation with LN PAI for the southern plots with r = -0.13, -0.28, and -0.15, respectively. According to the IVP of the species (S1 Table), aspen was the most dominant in the north and loblolly pine in the south.

## Discussion

One benefit of using data from plots in the entire eastern forest was that we identified a fairly large number of undisturbed plots. That was because the plots were in relatively young stands; with no record of thinning or other removals since the previous inventory. Additionally, it was unlikely such treatments were carried out before that, due to the young stand age and therefore lack of merchantable trees.

The observed species richness of between 1 and 15, average 4.6 species per plot, was comparable to that reported by others. A previous study [17] using 79,324 FIA plots across the 48 contiguous states found mean species richness of 2.40 for low stocked-low productivity sites and 6.97 for high stocked-medium productivity sites. Our study found average PAI of 3.19 Mg ha^-1^ yr^-1^, which is comparatively low, possibly due to the use of such young stands that have not reached the peak of their periodic and mean annual increment. Another estimate of aboveground production of woody biomass (which included both net growth and woody biomass lost due to mortality) using data of the eastern US forests found a range of 0.6 to 28 Mg ha^-1^ yr^-1^, average of 5.2, for hardwood forests and 0.2 to 31 Mg ha^-1^ yr^-1^, average of 4.9, for softwood forests [63].

A number of earlier studies [18,64] based on national inventory data considered stand density in the analysis of the diversity-productivity relationship. They found that when compared to species richness, stand density was a stronger determinant of productivity. We similarly found that in comparison to species richness, relative stand density had a stronger association with PAI (Fig 2; S2 Table). The competitive and complementary interactions between tree species change with increasing stand density depending upon the availability of the limiting resources and upon climatic conditions [65]. Complementarity effects cause greater productivity when the increase in tree density strengthen the complementarity interactions more than it intensifies the competition for limiting resources among species in the community [66].

While the compacted crown ratio, height, and quadratic mean diameter showed no significant direct correlation with the PAI in the measurement period, there was a negative association between compacted crown ratio and MAI, possibly due to the inverse relationship between crown size and stand density. Trees in high density stands generally form small crowns due to competition.

Although it was weak, the relationship between PAI and species richness was positive across the plots of the study area (Fig 3). MAI exhibited an even stronger association with species richness than did PAI. While our work does not explore the mechanisms, two often cited reasons for a positive diversity-productivity relationship are that 1) the probability for niche partitioning, or facilitation between species, is greater in species-rich communities, which enables them to more efficiently access and use limited resources [6], and 2) in species rich communities there is a greater probability of having some highly productive dominant species in the community due to the selection or sampling effect [6,18].

The relationship between species richness and PAI also depended on stand stocking and site productivity. Our findings are consistent with the common observation in forest ecology that a positive association between diversity and productivity is most likely to occur on sites with low productivity [17] and low stocking [16,17]. Species richness appeared more important than environmental and climatic factors for the variation in PAI in low productivity sites (Table 3). The presence of functionally different species enhances forest productivity in low productive sites, which supports the stress gradient hypothesis in which complementary interactions among species are found under harsher environmental conditions [67]. Highly productive and dominant species can compete and grow well on more productive sites, where species diversity is weakly associated with productivity [17]. Others [18] similarly reported stronger and positive diversity-productivity relationship in the harsher conditions of the boreal forests compared to temperate forests, concluding that species interactions are dominated by competitive exclusion in productive environments, whereas facilitative interactions are possible and more likely in stressful environments.

Our observed higher productivity in the stands of species of mixed shade tolerance is considered possible because of complementary resource use between species that occur particularly due to light and soil resource partitioning, through canopy and root stratification and sometimes from the facilitative improvement of a species from a nitrogen-fixing species in the mixture [68]. A stratified canopy with shade intolerant species in the overstory and shade tolerant species in the understory use available light resources more efficiently than stands containing only shade intolerant or only shade tolerant species [25,69].

The stronger association between species richness and PAI in hardwood stands than in mixed (conifer/pine-hardwood) stands and the greater PAI in mixed stands than hardwood stands are consistent with the claims from other studies. A stand with a combination of species differing in functional characteristics such as shade tolerance, height growth rate, crown structure, leaf phenology, and root depth can be highly productive because of the complementary resource use or facilitation [25]. When comparing monospecific, two, or three species mixtures to the four and five species mixtures in sclerophilous and conifer forests in Catalonia, Spain [14], the stemwood production in the latter was 19.6% and 45.8% greater, respectively. The conifer/pine stands in our study had very low species diversity (species richness 1 to 4) and showed no significant relationship with PAI, which is possibly due to the presence of low diversity in species functional traits and low functional variation among species. However, the dominant tree species in species mixtures are also important determinants for productivity [70]. The species characteristics of a mixture are important factors that sometimes determine whether mixtures are more productive than monospecific stands of similar age, tree stocking, soil characteristics, and management history. In boreal forests, greater productivity has been observed of birch (*Betula* spp.) and spruce (*Picea abies*) mixtures than of pure spruce stands, but lower productivity of birch and Scotts pine (*Pinus sylvestris*) mixture than pure pine stands [14].

Species richness did not seem correspond to greater, or lower, PAI in stands of only intolerant or only tolerant species, but was an important factor in stands composed of a mix of shade intolerant and shade tolerant species. Additionally, having more species in the composition was more important for the productivity of stands containing only hardwoods than for the productivity of stands that already contained both hardwoods and conifers. Similarly, an earlier study [71] reported an average of 48% more aboveground wood productivity in mixed pine-oak stands than in pure stands in Mediterranean forests. The mixing of species with diverse ecological requirements, such as light and water demanding pines and more shade and drought tolerant oaks, increases the complementary and more optimal use of resources [71].

Unlike species richness, the height, crown ratio, precipitation, and temperature were all important factors for the productivity in conifer/pine stands, as they appeared in at least one of the two best models. Most conifers in the dataset from the studied region were pines with fairly similar resource requirements, at least in comparison to the diverse requirements of the various hardwoods. Because of these similarities, the competitive inhibition is strongest in pines growing in monocultures or in mixtures with other pines/conifers [71], where the increase of species richness due to more conifer/pine species does not add more productivity due to poor complementarity among the species.

Species richness was more strongly associated with PAI productivity than temperature and precipitation in tolerant-intolerant mixes and stands of mixed hardwoods. Our result supports the hypothesis that the facilitative interactions among species occur in species rich communities, where stands consist of diverse functional variation among species and share dissimilar ecological niches of the species that leads to efficient utilization of limiting resources through resource partitioning, resulting in higher biomass productivity.

Our results concerning the relationship between species richness and PAI in stands dominated by aspen and those dominated by loblolly pine support our previous findings from plots in Alabama [72] of a positive relationship between species richness and productivity in hardwood forests, but not in conifer/pine forests.

The observed positive correlation between PAI and diversity indices functional diversity, Faith’s phylogenetic diversity, and phylogenetic species richness indicates that each of these diversity indices are important in explaining the variation in PAI productivity across the studied plots. Other studies have found that species richness can be a substitute for functional diversity when the diversity indices show linear relationship [48]. Species richness has also been found to be just as important as phylogenetic diversity to describe forest productivity [18]. Other researchers have found a correlation between functional diversity and species richness and suggested that either can be used as a biodiversity metric to describe ecosystem functioning [73].

A partial correlation, after controlling for climate and environmental variables, showed that stand stocking had a significant impact on the correlation between the diversity indices and PAI. Species richness, functional diversity, functional dispersion, Faith’s phylogenetic diversity, and phylogenetic species richness were all significantly correlated with PAI in low stocked stands with or without controlling for climate and environmental variables. And only the functional dispersion shifted to negative association with PAI with an increase in stocking. While Faith’s phylogenetic diversity index is not an independent metric of species richness [18], it captured almost as much variation as functional dispersion for explaining PAI in low stocked stands. Phylogenetic species richness explained as much variation as functional diversity on PAI in low stocked stands. Phylogenetic diversity is generally considered, however, an appropriate biodiversity metric because it explains evolutionary history and often correlates well with genetic diversity [74].

There was a significant effect of site productivity on the relationship between PAI and the diversity indices when climatic and environmental variables were controlled. Species richness and functional diversity for low productivity sites, and phylogenetic species richness for medium productivity sites were the best biodiversity metrics for explaining PAI productivity of the respective sites. Others have found that species richness and phylogenetic species clustering are the best biodiversity metrics for above ground biomass prediction in low productivity sites [17].

Phylogenetic species richness, Faith’s phylogenetic diversity, and species richness were the best diversity indices for explaining the variation of the productivity in stands containing a mix of shade tolerant and intolerant species. In hardwoods however, species richness, followed by functional diversity and phylogenetic diversity were the most important indices in accounting for the variation in PAI. The climatic and environmental factors influenced the association between productivity and diversity in conifer/pine stands–without controlling these two factors the relationship was not significant, but controlling them resulted in a significant relationship.

The Pearson correlation between PAI and all but one of the diversity indices was positive and weak for the plots north of 40° latitude, where hardwood species were dominant, but negative and weak with three of the five diversity indices for the plots south of 40° latitude, where pines were dominant. Previous work [75], also using FIA data, reported no association between species diversity and height diversity and the dependent variable productivity in two forest cover types–aspen and sugar maple-beech-birch (although they calculated productivity differently). They concluded that competing shade tolerant species in aspen stands apparently reduced aspen growth more than they added to total stand productivity. We found the opposite–our young aspen stands showed overall greater productivity with the increase in the number of species in the stand.

## Conclusion

Greater tree species diversity in forest ecosystems may not always or everywhere associate with higher productivity. In our study, greater species diversity was associated with greater species functional variation and productivity on low productivity sites. Species diversity correlated very weakly with productivity in highly productive sites where a few dominant species may regulate the productivity. The presence of multiple functional groups in hardwood stands, hardwood-conifer mixed stands, or shade tolerant-intolerant mixed stands likely increase the dimensions of the functional traits, resulting in increased productivity. In our study, the results concerning the growth of stands composed of species from different shade tolerance levels, as compared to stands whose composition includes species of only one tolerance level, suggest that the niche partitioning is likely to be at least partially responsible for the observed equal or greater productivity of mixtures. Species richness was not correlated with above ground biomass productivity in conifer/pine forests. Species richness had a stronger relationship with periodic annual aboveground biomass increment, when compared to climate and environmental factors, for low stocking stands and low productive sites. The results could help foresters control stand structures and species compositions to optimize growth and satisfy the needs for woody biomass and carbon sequestration. If the goal of the management is to maximize biomass production and maintain diversity, having mixed stands comprising functionally diverse species may be beneficial. Increasing and maintaining species diversity in degraded stands or on sites with harsh environmental conditions by increasing the diversity of functional traits may possibly increase tree aboveground biomass productivity.

## Supporting information

S1 Fig Bar diagram showing the number of plots for all 15 species richness levels. (TIF)

S1 Table The Importance Value Percent (IVP) of all species across all 967 plots in descending order. (DOCX)

S2 Table Pearson bivariate correlation between LN (PAI) and the predictor variables. (DOCX)

S3 Table The GLM for square root MAI and species richness (SPR) by different classes of stand stocking, site productivity, shade tolerance, and major species groups. (DOCX)

S4 Table Best multiple regression predictive models for LN PAI by stand stocking class, site productivity class, shade tolerance class, and major species groups across 829 FIA plots in young forests in the eastern US. (DOCX)

S5 Table Best multiple regression predictive models for square root MAI by stand stocking, site productivity, shade tolerance, and major species groups across the 829 FIA plots in young forests in the eastern US. (DOCX)

S1 File USDA FIA data set. (CSV)

S2 File Species traits dataset. (CSV)

S3 File Metadata. (DOCX)

S4 File FIA database description and user manual. (PDF)

S5 File Specific gravity and other properties of wood and bark (Research note). (PDF)
