# Supplementary figures and images for: Variation in the diversity-productivity relationship in young forests of the eastern United States
Source: PLoS One. 2017 Nov 15;12(11):e0187106. doi: 10.1371/journal.pone.0187106 (PMC5687711; doi:10.1371/journal.pone.0187106)

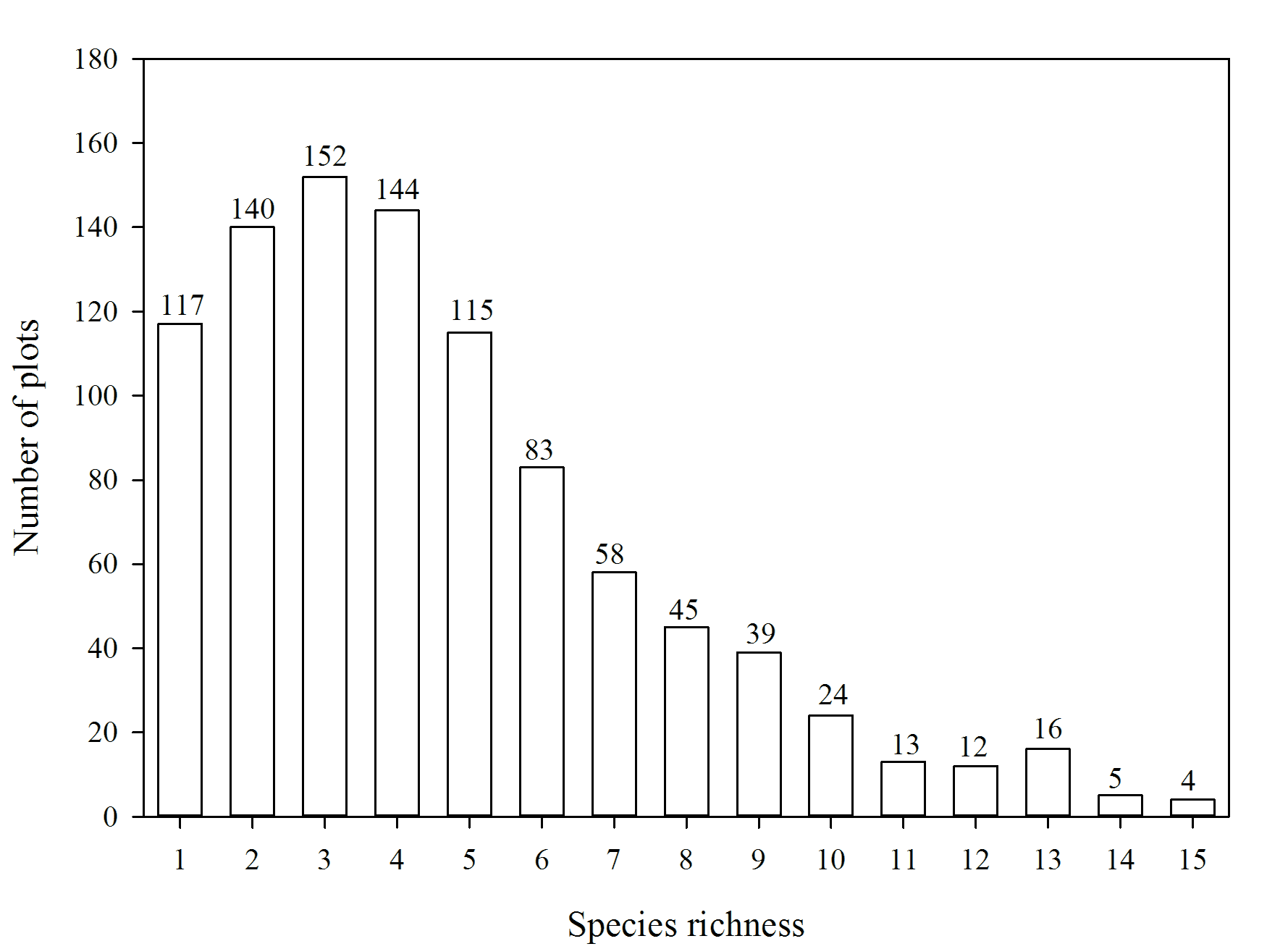

Supplement: S1 Fig — (TIF) [file pone.0187106.s001.tif]
