# Supplementary material for: Variation in the diversity-productivity relationship in young forests of the eastern United States
Source: PLoS One. 2017 Nov 15;12(11):e0187106. doi: 10.1371/journal.pone.0187106 (PMC5687711; doi:10.1371/journal.pone.0187106)
Supplement: S1 Table — (DOCX) [file pone.0187106.s002.docx]

**Table S1. The Importance Value Percent (IVP) of all species across all 967 plots in descending order.**

| FIA Species Code | Common name | Scientific name | Density (Stems ha^-1^) | Basal area (m^2^ ha^-1^) | Relative Frequency (percent) | Relative Density (percent) | Relative Dominance (percent) | Importance value (percent) |
| --- | --- | --- | --- | --- | --- | --- | --- | --- |
| 746 | quaking aspen | *Populus tremuloides* | 98.14 | 0.402 | 8.10 | 24.45 | 7.89 | 13.48 |
| 131 | loblolly pine | *Pinus taeda* | 37.23 | 0.769 | 3.06 | 9.28 | 15.09 | 9.14 |
| 316 | red maple | *Acer rubrum* | 32.21 | 0.356 | 8.18 | 8.02 | 6.99 | 7.73 |
| 611 | sweetgum | *Liquidambar styraciflua* | 17.23 | 0.290 | 3.17 | 4.29 | 5.69 | 4.38 |
| 12 | balsam fir | *Abies balsamea* | 17.26 | 0.133 | 3.81 | 4.30 | 2.61 | 3.58 |
| 375 | paper birch | *Betula papyrifera* | 11.78 | 0.111 | 4.10 | 2.93 | 2.17 | 3.07 |
| 621 | yellow-poplar | *Liriodendron tulipifera* | 9.84 | 0.214 | 1.90 | 2.45 | 4.20 | 2.85 |
| 318 | sugar maple | *Acer saccharum* | 8.22 | 0.181 | 2.54 | 2.05 | 3.55 | 2.71 |
| 762 | black cherry | *Prunus serotina* | 7.33 | 0.083 | 3.77 | 1.83 | 1.63 | 2.41 |
| 743 | bigtooth aspen | *Populus grandidentata* | 12.54 | 0.076 | 2.34 | 3.13 | 1.48 | 2.32 |
| 827 | water oak | *Quercus nigra* | 6.73 | 0.123 | 2.03 | 1.68 | 2.41 | 2.04 |
| 802 | white oak | *Quercus alba* | 4.81 | 0.133 | 1.98 | 1.20 | 2.61 | 1.93 |
| 833 | northern red oak | *Quercus rubra* | 3.84 | 0.093 | 1.96 | 0.96 | 1.82 | 1.58 |
| 129 | eastern white pine | *Pinus strobus* | 2.77 | 0.129 | 1.38 | 0.69 | 2.52 | 1.53 |
| 531 | American beech | *Fagus grandifolia* | 4.07 | 0.077 | 1.43 | 1.01 | 1.50 | 1.32 |
| 693 | blackgum | *Nyssa sylvatica* | 3.92 | 0.056 | 1.74 | 0.98 | 1.10 | 1.27 |
| 741 | balsam poplar | *Populus balsamifera* | 6.06 | 0.038 | 1.34 | 1.51 | 0.75 | 1.20 |
| 544 | green ash | *Fraxinus pennsylvanica* | 4.41 | 0.042 | 1.58 | 1.10 | 0.83 | 1.17 |
| 812 | southern red oak | *Quercus falcata* | 2.44 | 0.074 | 1.34 | 0.61 | 1.44 | 1.13 |
| 371 | yellow birch | *Betula alleghaniensis* | 3.27 | 0.057 | 1.40 | 0.82 | 1.13 | 1.12 |
| 541 | white ash | *Fraxinus americana* | 2.78 | 0.052 | 1.32 | 0.69 | 1.01 | 1.01 |
| 543 | black ash | *Fraxinus nigra* | 3.17 | 0.048 | 1.27 | 0.79 | 0.94 | 1.00 |
| 97 | red spruce | *Picea rubens* | 4.07 | 0.046 | 1.07 | 1.01 | 0.90 | 0.99 |
| 972 | American elm | *Ulmus americana* | 2.26 | 0.048 | 1.47 | 0.56 | 0.94 | 0.99 |
| 761 | pin cherry | *Prunus pensylvanica* | 4.66 | 0.011 | 1.52 | 1.16 | 0.22 | 0.97 |
| 409 | mockernut hickory | *Carya alba* | 2.31 | 0.051 | 1.20 | 0.57 | 1.00 | 0.93 |
| 111 | slash pine | *Pinus elliottii* | 3.72 | 0.064 | 0.51 | 0.93 | 1.26 | 0.90 |
| 94 | white spruce | *Picea glauca* | 2.01 | 0.050 | 0.94 | 0.50 | 0.98 | 0.81 |
| 837 | black oak | *Quercus velutina* | 2.01 | 0.043 | 1.07 | 0.50 | 0.84 | 0.81 |
| 125 | red pine | *Pinus resinosa* | 2.64 | 0.052 | 0.71 | 0.66 | 1.02 | 0.80 |
| 110 | shortleaf pine | *Pinus echinata* | 2.46 | 0.054 | 0.67 | 0.61 | 1.06 | 0.78 |
| 491 | flowering dogwood | *Cornus florida* | 2.44 | 0.011 | 1.34 | 0.61 | 0.21 | 0.72 |
| 823 | bur oak | *Quercus macrocarpa* | 1.41 | 0.050 | 0.80 | 0.35 | 0.98 | 0.71 |
| 835 | post oak | *Quercus stellata* | 2.08 | 0.035 | 0.83 | 0.52 | 0.68 | 0.67 |
| 951 | American basswood | *Tilia americana* | 2.11 | 0.042 | 0.54 | 0.52 | 0.82 | 0.63 |
| 971 | winged elm | *Ulmus alata* | 1.80 | 0.023 | 1.00 | 0.45 | 0.45 | 0.63 |
| 403 | pignut hickory | *Carya glabra* | 1.08 | 0.030 | 0.91 | 0.27 | 0.58 | 0.59 |
| 67 | southern redcedar | *Juniperus virginiana* | 1.51 | 0.030 | 0.80 | 0.38 | 0.59 | 0.59 |
| 132 | Virginia pine | *Pinus virginiana* | 2.18 | 0.038 | 0.49 | 0.54 | 0.75 | 0.59 |
| 372 | sweet birch | *Betula lenta* | 2.80 | 0.022 | 0.60 | 0.70 | 0.43 | 0.58 |
| 261 | eastern hemlock | *Tsuga canadensis* | 1.01 | 0.047 | 0.51 | 0.25 | 0.92 | 0.56 |
| 832 | chestnut oak | *Quercus prinus* | 1.72 | 0.038 | 0.47 | 0.43 | 0.75 | 0.55 |
| 901 | black locust | *Robinia pseudoacacia* | 1.68 | 0.019 | 0.78 | 0.42 | 0.38 | 0.53 |
| 315 | striped maple | *Acer pensylvanicum* | 2.86 | 0.004 | 0.76 | 0.71 | 0.09 | 0.52 |
| 922 | black willow | *Salix nigra* | 1.29 | 0.039 | 0.47 | 0.32 | 0.76 | 0.52 |
| 820 | laurel oak | *Quercus laurifolia* | 1.77 | 0.029 | 0.54 | 0.44 | 0.56 | 0.51 |
| 831 | willow oak | *Quercus phellos* | 1.37 | 0.033 | 0.49 | 0.34 | 0.65 | 0.50 |
| 241 | northern white-cedar | *Thuja occidentalis* | 0.80 | 0.033 | 0.62 | 0.20 | 0.65 | 0.49 |
| 694 | swamp tupelo | *Nyssa biflora* | 1.38 | 0.042 | 0.27 | 0.34 | 0.82 | 0.48 |
| 105 | jack pine | *Pinus banksiana* | 1.58 | 0.015 | 0.76 | 0.39 | 0.29 | 0.48 |
| 994 | Chinese tallowtree | *Triadica sebifera* | 2.17 | 0.031 | 0.29 | 0.54 | 0.60 | 0.48 |
| 391 | American hornbeam | *Carpinus caroliniana* | 1.91 | 0.010 | 0.74 | 0.47 | 0.19 | 0.47 |
| 379 | gray birch | *Betula populifolia* | 2.60 | 0.008 | 0.49 | 0.65 | 0.15 | 0.43 |
| 521 | common persimmon | *Diospyros virginiana* | 0.97 | 0.008 | 0.85 | 0.24 | 0.17 | 0.42 |
| 701 | eastern hophornbeam | *Ostrya virginiana* | 1.72 | 0.007 | 0.69 | 0.43 | 0.13 | 0.42 |
| 931 | sassafras | *Sassafras albidum* | 1.12 | 0.006 | 0.80 | 0.28 | 0.11 | 0.40 |
| 653 | sweetbay | *Magnolia virginiana* | 1.12 | 0.027 | 0.33 | 0.28 | 0.53 | 0.38 |
| 95 | black spruce | *Picea mariana* | 1.21 | 0.012 | 0.60 | 0.30 | 0.24 | 0.38 |
| 711 | sourwood | *Oxydendrum arboreum* | 1.23 | 0.012 | 0.58 | 0.31 | 0.24 | 0.37 |
| 806 | scarlet oak | *Quercus coccinea* | 1.17 | 0.014 | 0.56 | 0.29 | 0.27 | 0.37 |
| 313 | boxelder | *Acer negundo* | 0.98 | 0.022 | 0.36 | 0.25 | 0.44 | 0.35 |
| 591 | American holly | *Ilex opaca* | 1.09 | 0.007 | 0.65 | 0.27 | 0.13 | 0.35 |
| 602 | black walnut | *Juglans nigra* | 0.49 | 0.026 | 0.31 | 0.12 | 0.52 | 0.32 |
| 221 | baldcypress | *Taxodium distichum* | 0.74 | 0.028 | 0.16 | 0.18 | 0.55 | 0.30 |
| 731 | American sycamore | *Platanus occidentalis* | 0.60 | 0.021 | 0.29 | 0.15 | 0.40 | 0.28 |
| 975 | slippery elm | *Ulmus rubra* | 0.55 | 0.013 | 0.47 | 0.14 | 0.25 | 0.28 |
| 408 | black hickory | *Carya texana* | 0.77 | 0.013 | 0.36 | 0.19 | 0.25 | 0.27 |
| 809 | northern pin oak | *Quercus ellipsoidalis* | 0.98 | 0.008 | 0.42 | 0.25 | 0.15 | 0.27 |
| 500 | hawthorn spp. | *Crataegus spp.* | 1.25 | 0.003 | 0.40 | 0.31 | 0.07 | 0.26 |
| 641 | Osage-orange | *Maclura pomifera* | 0.92 | 0.017 | 0.20 | 0.23 | 0.33 | 0.26 |
| 461 | sugarberry | *Celtis laevigata* | 0.52 | 0.020 | 0.22 | 0.13 | 0.39 | 0.25 |
| 319 | mountain maple | *Acer spicatum* | 1.09 | 0.001 | 0.42 | 0.27 | 0.02 | 0.24 |
| 356 | serviceberry spp. | *Amelanchier spp.* | 0.63 | 0.002 | 0.51 | 0.16 | 0.04 | 0.24 |
| 742 | eastern cottonwood | *Populus deltoides* | 0.63 | 0.023 | 0.11 | 0.16 | 0.45 | 0.24 |
| 813 | cherrybark oak | *Quercus pagoda* | 0.46 | 0.014 | 0.31 | 0.11 | 0.28 | 0.24 |
| 407 | shagbark hickory | *Carya ovata* | 0.51 | 0.012 | 0.29 | 0.13 | 0.24 | 0.22 |
| 71 | tamarack (native) | *Larix laricina* | 0.58 | 0.008 | 0.36 | 0.15 | 0.15 | 0.22 |
| 824 | blackjack oak | *Quercus marilandica* | 0.66 | 0.011 | 0.27 | 0.16 | 0.21 | 0.21 |
| 838 | live oak | *Quercus virginiana* | 0.52 | 0.012 | 0.27 | 0.13 | 0.24 | 0.21 |
| 552 | honeylocust | *Gleditsia triacanthos* | 0.58 | 0.011 | 0.25 | 0.15 | 0.22 | 0.20 |
| 121 | longleaf pine | *Pinus palustris* | 0.57 | 0.011 | 0.22 | 0.14 | 0.21 | 0.19 |
| 763 | chokecherry | *Prunus virginiana* | 0.66 | 0.001 | 0.40 | 0.16 | 0.02 | 0.19 |
| 373 | river birch | *Betula nigra* | 0.45 | 0.017 | 0.09 | 0.11 | 0.33 | 0.18 |
| 402 | bitternut hickory | *Carya cordiformis* | 0.25 | 0.011 | 0.20 | 0.06 | 0.22 | 0.16 |
| 462 | hackberry | *Celtis occidentalis* | 0.49 | 0.005 | 0.27 | 0.12 | 0.10 | 0.16 |
| 660 | apple spp. | *Malus spp.* | 0.42 | 0.006 | 0.22 | 0.10 | 0.11 | 0.15 |
| 721 | redbay | *Persea borbonia* | 0.43 | 0.004 | 0.27 | 0.11 | 0.07 | 0.15 |
| 222 | pondcypress | *Taxodium ascendens* | 0.51 | 0.009 | 0.13 | 0.13 | 0.18 | 0.15 |
| 107 | sand pine | *Pinus clausa* | 0.98 | 0.005 | 0.07 | 0.25 | 0.11 | 0.14 |
| 555 | loblolly-bay | *Gordonia lasianthus* | 0.63 | 0.006 | 0.11 | 0.16 | 0.11 | 0.13 |
| 471 | eastern redbud | *Cercis canadensis* | 0.43 | 0.001 | 0.22 | 0.11 | 0.03 | 0.12 |
| 341 | ailanthus | *Ailanthus altissima* | 0.35 | 0.007 | 0.11 | 0.09 | 0.13 | 0.11 |
| 682 | red mulberry | *Morus rubra* | 0.38 | 0.003 | 0.18 | 0.10 | 0.05 | 0.11 |
| 921 | peachleaf willow | *Salix amygdaloides* | 0.55 | 0.001 | 0.18 | 0.14 | 0.01 | 0.11 |
| 825 | swamp chestnut oak | *Quercus michauxii* | 0.14 | 0.005 | 0.16 | 0.03 | 0.10 | 0.10 |
| 404 | pecan | *Carya illinoinensis* | 0.18 | 0.005 | 0.11 | 0.05 | 0.10 | 0.09 |
| 691 | water tupelo | *Nyssa aquatica* | 0.18 | 0.008 | 0.04 | 0.05 | 0.17 | 0.09 |
| 817 | shingle oak | *Quercus imbricaria* | 0.29 | 0.002 | 0.11 | 0.07 | 0.04 | 0.08 |
| 822 | overcup oak | *Quercus lyrata* | 0.08 | 0.006 | 0.09 | 0.02 | 0.12 | 0.08 |
| 311 | Florida maple | *Acer barbatum* | 0.15 | 0.002 | 0.13 | 0.04 | 0.04 | 0.07 |
| 840 | dwarf post oak | *Quercus margarettiae* | 0.22 | 0.003 | 0.09 | 0.05 | 0.05 | 0.07 |
| 935 | American mountain-ash | *Sorbus americana* | 0.29 | 0.001 | 0.13 | 0.07 | 0.01 | 0.07 |
| 410 | sand hickory | *Carya pallida* | 0.20 | 0.003 | 0.07 | 0.05 | 0.05 | 0.06 |
| 115 | spruce pine | *Pinus glabra* | 0.17 | 0.003 | 0.09 | 0.04 | 0.06 | 0.06 |
| 130 | Scotch pine | *Pinus sylvestris* | 0.18 | 0.002 | 0.09 | 0.05 | 0.04 | 0.06 |
| 317 | silver maple | *Acer saccharinum* | 0.17 | 0.002 | 0.07 | 0.04 | 0.05 | 0.05 |
| 401 | water hickory | *Carya aquatica* | 0.17 | 0.002 | 0.07 | 0.04 | 0.05 | 0.05 |
| 655 | mountain or Fraser magnolia | *Magnolia fraseri* | 0.09 | 0.005 | 0.02 | 0.02 | 0.10 | 0.05 |
| 652 | southern magnolia | *Magnolia grandiflora* | 0.11 | 0.002 | 0.09 | 0.03 | 0.03 | 0.05 |
| 993 | chinaberry | *Melia azedarach* | 0.18 | 0.002 | 0.07 | 0.05 | 0.03 | 0.05 |
| 128 | pond pine | *Pinus serotina* | 0.08 | 0.004 | 0.07 | 0.02 | 0.07 | 0.05 |
| 920 | willow spp. | *Salix spp.* | 0.26 | 0.000 | 0.09 | 0.07 | 0.01 | 0.05 |
| 405 | shellbark hickory | *Carya laciniosa* | 0.14 | 0.001 | 0.07 | 0.03 | 0.02 | 0.04 |
| 651 | Cucumber tree | *Magnolia acuminata* | 0.08 | 0.001 | 0.09 | 0.02 | 0.02 | 0.04 |
| 126 | pitch pine | *Pinus rigida* | 0.11 | 0.001 | 0.07 | 0.03 | 0.02 | 0.04 |
| 819 | turkey oak | *Quercus laevis* | 0.12 | 0.001 | 0.09 | 0.03 | 0.01 | 0.04 |
| 841 | dwarf live oak | *Quercus minima* | 0.12 | 0.001 | 0.09 | 0.03 | 0.01 | 0.04 |
| 830 | pin oak | *Quercus palustris* | 0.11 | 0.001 | 0.07 | 0.03 | 0.02 | 0.04 |
| 987 | buttonwood-mangrove | *Conocarpus erectus* | 0.20 | 0.001 | 0.02 | 0.05 | 0.01 | 0.03 |
| 548 | Carolina ash | *Fraxinus caroliniana* | 0.08 | 0.000 | 0.07 | 0.02 | 0.00 | 0.03 |
| 989 | American mangrove | *Rhizophora mangle* | 0.28 | 0.000 | 0.02 | 0.07 | 0.01 | 0.03 |
| 323 | chalk maple | *Acer leucoderme* | 0.03 | 0.000 | 0.04 | 0.01 | 0.01 | 0.02 |
| 345 | mimosa, silktree | *Albizia julibrissin* | 0.03 | 0.001 | 0.04 | 0.01 | 0.01 | 0.02 |
| 915 | other palms | *Family Arecaceae* | 0.02 | 0.002 | 0.02 | 0.00 | 0.04 | 0.02 |
| 988 | white-mangrove | *Laguncularia racemosa* | 0.09 | 0.000 | 0.02 | 0.02 | 0.00 | 0.02 |
| 70 | larch spp. | *Larix spp.* | 0.06 | 0.000 | 0.02 | 0.02 | 0.01 | 0.02 |
| 722 | water-elm, planer tree | *Planera aquatica* | 0.11 | 0.001 | 0.02 | 0.03 | 0.02 | 0.02 |
| 766 | American plum | *Prunus americana* | 0.11 | 0.000 | 0.02 | 0.03 | 0.00 | 0.02 |
| 771 | sweet cherry, domesticated | *Prunus avium* | 0.03 | 0.000 | 0.04 | 0.01 | 0.00 | 0.02 |
| 826 | chinkapin oak | *Quercus muehlenbergii* | 0.05 | 0.001 | 0.04 | 0.01 | 0.02 | 0.02 |
| 951 | American basswood | *Tilia americana* | 0.05 | 0.001 | 0.04 | 0.01 | 0.02 | 0.02 |
| 951 | American basswood | *Tilia americana* | 0.05 | 0.001 | 0.02 | 0.01 | 0.02 | 0.02 |
| 999 | Other or unknown live tree | *Tree unknown* | 0.05 | 0.000 | 0.04 | 0.01 | 0.01 | 0.02 |
| 974 | Siberian elm | *Ulmus pumila* | 0.08 | 0.001 | 0.02 | 0.02 | 0.03 | 0.02 |
| 314 | black maple | *Acer nigrum* | 0.02 | 0.000 | 0.02 | 0.00 | 0.00 | 0.01 |
| 332 | yellow buckeye | *Aesculus flava* | 0.06 | 0.000 | 0.02 | 0.02 | 0.00 | 0.01 |
| 451 | southern catalpa | *Catalpa bignonioides* | 0.03 | 0.001 | 0.02 | 0.01 | 0.01 | 0.01 |
| 663 | sweet crab apple | *Malus coronaria* | 0.02 | 0.001 | 0.02 | 0.00 | 0.01 | 0.01 |
| 712 | paulownia, empress-tree | *Paulownia tomentosa* | 0.02 | 0.001 | 0.02 | 0.00 | 0.01 | 0.01 |
| 91 | Norway spruce | *Picea abies* | 0.02 | 0.000 | 0.02 | 0.00 | 0.01 | 0.01 |
| 842 | bluejack oak | *Quercus incana* | 0.02 | 0.000 | 0.02 | 0.00 | 0.01 | 0.01 |
| 845 | dwarf chinkapin oak | *Quercus prinoides* | 0.02 | 0.000 | 0.02 | 0.00 | 0.00 | 0.01 |
| 834 | Shumard oak | *Quercus shumardii* | 0.02 | 0.000 | 0.02 | 0.00 | 0.00 | 0.01 |
| 800 | oak spp. | *Quercus spp.* | 0.02 | 0.000 | 0.02 | 0.00 | 0.00 | 0.01 |
| 828 | Texas red oak | *Quercus texana* | 0.02 | 0.000 | 0.02 | 0.00 | 0.01 | 0.01 |
| 381 | chittamwood, gum bumelia | *Sideroxylon lanuginosum* | 0.02 | 0.000 | 0.02 | 0.00 | 0.01 | 0.01 |
| 977 | rock elm | *Ulmus thomasii* | 0.02 | 0.000 | 0.02 | 0.00 | 0.00 | 0.01 |
