# Supplementary material for: Variation in the diversity-productivity relationship in young forests of the eastern United States
Source: PLoS One. 2017 Nov 15;12(11):e0187106. doi: 10.1371/journal.pone.0187106 (PMC5687711; doi:10.1371/journal.pone.0187106)
Supplement: S2 Table — (DOCX) [file pone.0187106.s003.docx]

**Table S2. Pearson bivariate correlation between LN (PAI) and the predictor variables.**

The ** and * indicate a significant correlation at α=0.01 and 0.05 levels, respectively.

|  | LN  (PAI) | RD | HT | QMD | SPR | CCR | SL | AS | PPT | TEMP | ELEV |
| --- | --- | --- | --- | --- | --- | --- | --- | --- | --- | --- | --- |
| LN  (PAI) | 1 | 0.33** | -0.006 | -0.04 | 0.25** | 0.01 | 0.05 | -0.02 | 0.27** | 0.27** | -0.18** |
| RD |  | 1 | 0.21** | 0.06 | 0.56** | -0.35** | 0.12** | -0.07* | 0.25** | 0.28** | -0.16** |
| HT |  |  | 1 | 0.16** | 0.20** | -0.31** | 0.09* | -0.00 | -0.35** | -0.36** | 0.23** |
| QMD |  |  |  | 1 | 0.03 | 0.11** | -0.02 | 0.04 | 0.20** | 0.28** | -0.17** |
| SPR |  |  |  |  | 1 | -0.20** | 0.31** | -0.14** | 0.30** | 0.26** | -0.15** |
| CCR |  |  |  |  |  | 1 | -0.04 | 0.03 | 0.07* | -0.00 | -0.01 |
| SL |  |  |  |  |  |  | 1 | -0.51** | 0.08* | -0.03 | 0.26** |
| AS |  |  |  |  |  |  |  | 1 | -0.04 | 0.02 | -0.16** |
| PPT |  |  |  |  |  |  |  |  | 1 | 0.80** | -0.48** |
| TEMP |  |  |  |  |  |  |  |  |  | 1 | -0.70** |
| ELEV |  |  |  |  |  |  |  |  |  |  | 1 |

Where LN (PAI) = Log-modulus transformed periodic annual aboveground biomass increment; RD= relative stand density; HT=Height (m); QMD= Quadratic mean diameter (cm); SPR=Species richness; CCR= Compacted crown ratio; SL=Slope (arcsine transformed); AS= Aspect (Beers transformed); PPT= Mean precipitation (cm); TEMP= Mean temperature (°C); ELEV= Elevation (m).
