# Supplementary material for: Variation in the diversity-productivity relationship in young forests of the eastern United States
Source: PLoS One. 2017 Nov 15;12(11):e0187106. doi: 10.1371/journal.pone.0187106 (PMC5687711; doi:10.1371/journal.pone.0187106)
Supplement: S3 Table — (DOCX) [file pone.0187106.s004.docx]

**Table S3. The GLM for square root MAI and species richness (SPR) by different classes of stand stocking, site productivity, shade tolerance, and major species groups.**

Different superscripts within a class/group indicate significant differences at α=0.05 (Tukey-Kramer multiple comparison test). The range is shown in parenthesis.

|  |  |  | AGBG | | SPR | | GLM  Sq root^1^(MAI)= f(SPR) | | |
| --- | --- | --- | --- | --- | --- | --- | --- | --- | --- |
|  | Class | Plots | LS-Mean | SE | Mean | SE | R^2^ | *F* | *p* |
| Stand stocking class | Low stocked | 681 | 1.52^c^ (0.01, 15.04) | 0.059 | 3.68 (1, 15) | 0.084 | 0.24 | 17.46 | <0.0001 |
|  | Medium stocked | 262 | 3.92^b^ (0.88, 12.34) | 0.096 | 6.96 (1, 15) | 0.208 | 0.10 | 1.96 | 0.02 |
|  | High stocked | 24 | 5.42^a^ (2.06, 8.61) | 0.316 | 6.42 (1, 14) | 0.667 | 0.46 | 0.94 | 0.53 |
| Site productivity class | Low | 319 | 1.83^c^ (0.01, 15.04) | 0.107 | 3.80 (1, 14) | 0.131 | 0.39 | 17.01 | <0.0001 |
|  | Medium | 589 | 2.40^b^ (0.01, 13.89) | 0.079 | 4.88 (1, 15) | 0.124 | 0.26 | 14.35 | <0.0001 |
|  | High | 59 | 3.33^a^ (0.08, 12.34) | 0.249 | 6.71 (1, 15) | 0.549 | 0.37 | 1.82 | 0.07 |
| Shade tolerance class | Intolerant | 191 | 1.29^b^ (0.01, 8.45) | 0.134 | 1.79 (1, 6) | 0.070 | 0.16 | 6.84 | <0.0001 |
|  | Tolerant | 35 | 0.86^b^ (0.01, 7.84) | 0.314 | 1.83 (1, 5) | 0.181 | 0.48 | 6.98 | 0.0004 |
|  | Mixed | 733 | 2.60^a^ (0.03, 15.04) | 0.068 | 5.55 (2, 15) | 0.104 | 0.21 | 14.37 | <0.0001 |
| Major species group | Conifer/pine | 62 | 1.37^c^ (0.01, 7.06) | 0.243 | 1.37 (1, 4) | 0.087 | 0.01 | 0.18 | 0.91 |
|  | Hardwoods | 439 | 2.06^b^ (0.01, 15.04) | 0.091 | 3.89 (1, 14) | 0.118 | 0.39 | 21.22 | <0.0001 |
|  | Mixed | 466 | 2.59^a^ (0.05, 13.54) | 0.089 | 5.78 (2, 15) | 0.142 | 0.22 | 10.11 | <0.0001 |

^1^Sq root refers to the square root; MAI refers “mean annual aboveground biomass increment (Mg ha^-1^ yr^-1^)”; f(SPR) stands for “function of species richness”
