# Supplementary material for: Variation in the diversity-productivity relationship in young forests of the eastern United States
Source: PLoS One. 2017 Nov 15;12(11):e0187106. doi: 10.1371/journal.pone.0187106 (PMC5687711; doi:10.1371/journal.pone.0187106)
Supplement: S4 Table — (DOCX) [file pone.0187106.s005.docx]

**Table S4. Best multiple regression predictive models for LN PAI by stand stocking class, site productivity class, shade tolerance class, and major species groups across 829 FIA plots in young forests in the eastern US.**

The models with the lowest Akaike's Information Criterion (AIC) were selected from eight priori candidate models of each classification. Standardized coefficients (Beta) are presented as parameter estimates to show relative importance of predictors in the model. Bold values are significant at α=0.05.

| Classification | |  | Intercept | QMD | HT | CCR | SPR | SL | AS | PPT | TEMP | ELEV | EDF | Adj-R^2^ | AIC | *p* |
| --- | --- | --- | --- | --- | --- | --- | --- | --- | --- | --- | --- | --- | --- | --- | --- | --- |
| All plots | | Model with lowest AIC |  | **-0.18** | **0.17** | **0.12** | **0.13** | . | . | **0.14** | **0.23** | . | 822 | 0.14 | -1429.43 | <0.0001 |
|  |  | Alternate best model with fewer predictors |  | **-0.15** | **0.10** | . | **0.13** | . | . | . | **0.31** | . | 824 | 0.12 | -1413.73 | <0.0001 |
| Stand stocking class | |  |  |  |  |  |  |  |  |  |  |  |  |  |  |  |
|  | Low stocked | Model with lowest AIC |  | **-0.22** | **0.18** | **0.19** | **0.15** | . | . | **0.13** | **0.17** | . | 541 | 0.13 | -1062.17 | <0.0001 |
|  |  | Alternate best model with fewer predictors |  | **-0.17** | . | **0.15** | **0.20** | . | . | . | **0.20** | . | 543 | 0.11 | -1051.47 | <0.0001 |
|  | Medium stocked | Model with lowest AIC |  | . | . | **0.15** | . | . | . | 0.15 | **0.20** | . | 253 | 0.13 | -389.62 | <0.0001 |
|  |  | Alternate best model with fewer predictors |  | . | . | **0.16** | . | . | . | . | **0.33** | . | 254 | 0.12 | -389.29 | <0.0001 |
|  | High stocked | Model with lowest AIC |  | **-0.58** | . | . | . | . | -0.37 | . | . | **-0.48** | 20 | 0.22 | -29.27 | 0.04 |
|  |  | Alternate best model with fewer predictors | -Not | found- |  |  |  |  |  |  |  |  |  |  |  |  |
| Site productivity class | |  |  |  |  |  |  |  |  |  |  |  |  |  |  |  |
|  | Low | Model with lowest AIC |  | **-0.30** | **0.13** | **0.19** | **0.31** | -0.09 | . | . | . | **0.16** | 256 | 0.22 | -504.45 | <0.0001 |
|  |  | Alternate best model with fewer predictors |  | **-0.27** | . | **0.15** | **0.32** | . | . | . | . | **0.14** | 258 | 0.20 | -501.93 | <0.0001 |
|  | Medium | Model with lowest AIC |  | -0.08 | **0.12** | 0.09 | . | . | . | **0.19** | **0.23** | . | 501 | 0.12 | -857.64 | <0.0001 |
|  |  | Alternate best model with fewer predictors |  | . | 0.09 | 0.07 | . | . | . | **0.19** | **0.20** | . | 502 | 0.11 | -856.43 | <0.0001 |
|  | High | Model with lowest AIC |  | . | **0.29** | . | . | . | . | . | 0.26 | **-0.42** | 55 | 0.39 | -128.88 | <0.0001 |
|  |  | Alternate best model with fewer predictors |  | . | **0.23** | . | . | . | . | . | . | **-0.61** | 56 | 0.37 | -128.34 | <0.0001 |
| Shade tolerance class | |  |  |  |  |  |  |  |  |  |  |  |  |  |  |  |
|  | Intolerant | Model with lowest AIC |  | **-0.17** | 0.14 | . | . | . | . | -0.30 | **0.80** | . | 114 | 0.26 | -181.75 | <0.0001 |
|  |  | Alternate best model with fewer predictors |  | -0.13 | . | . | . | . | . | **-0.37** | **0.81** | . | 115 | 0.25 | -181.27 | <0.0001 |
|  | Tolerant | Model with lowest AIC |  | . | 0.45 | 0.39 | . | . | . | **0.94** | -0.50 | . | 21 | 0.30 | -53.04 | 0.01 |
|  |  | Alternate best model with fewer predictors |  | . | **0.57** | **0.46** | . | . | . | **0.65** | . | . | 22 | 0.25 | -51.80 | 0.02 |
|  | Mixed | Model with lowest AIC |  | **-0.21** | **0.16** | **0.11** | **0.15** | . | . | **0.19** | **0.18** | . | 670 | 0.14 | -1217.76 | <0.0001 |
|  |  | Alternate best model with fewer predictors |  | **-0.13** | . | . | **0.16** | . | . | **0.25** | . | . | 673 | 0.12 | -1202.81 | <0.0001 |
| Major species groups | |  |  |  |  |  |  |  |  |  |  |  |  |  |  |  |
|  | Conifer/pine | Model with lowest AIC |  | . | **0.37** | **0.34** | . | . | -0.20 | **0.43** | . | **-0.59** | 42 | 0.42 | -74.69 | <0.0001 |
|  |  | Alternate best model with fewer predictors |  | . | **0.37** | **0.38** | . | . | . | . | **0.86** | . | 44 | 0.37 | -73.04 | <0.0001 |
|  | Hardwoods | Model with lowest AIC |  | **-0.27** | **0.17** | . | **0.30** | . | 0.10 | **0.29** | **-0.18** | . | 334 | 0.20 | -639.03 | <0.0001 |
|  |  | Alternate best model with fewer predictors |  | **-0.29** | **0.21** | . | **0.24** | . | . | **0.16** | . | . | 336 | 0.19 | -636.27 | <0.0001 |
|  | Mixed | Model with lowest AIC |  | **-0.11** | **0.14** | **0.12** | . | . | . | **0.15** | **0.36** | . | 434 | 0.16 | -809.68 | <0.0001 |
|  |  | Alternate best model with fewer predictors |  | . | . | **0.10** | **0.11** | . | . | . | **0.36** | . | 436 | 0.15 | -806.20 | <0.0001 |

Where LN= Log-modulus transformation; PAI=periodic annual aboveground biomass increment (Mg ha^-1^ yr^-1^); QMD= Quadratic mean diameter (cm); HT= Average height (m); CCR= Compacted crown ratio; SPR=Species richness; SL=Slope (arcsine transformed); AS= Aspect (Beers transformed); PPT= Mean precipitation (cm); TEMP= Mean temperature (°C); ELEV= Elevation (m); EDF=Error degrees of freedom; *p*=Statistical significance value. For multiple regression analysis, we excluded 138 (out of 967) plots because of no height data in those plots.
