# Supplementary material for: Variation in the diversity-productivity relationship in young forests of the eastern United States
Source: PLoS One. 2017 Nov 15;12(11):e0187106. doi: 10.1371/journal.pone.0187106 (PMC5687711; doi:10.1371/journal.pone.0187106)
Supplement: S3 File — (DOCX) [file pone.0187106.s009.docx]

**Metadata (Species Traits)**

List of tree traits used in estimating plot level functional diversity and functional dispersion indices.

| Variable code | Trait name | Variable type | Categories | | | | References |
| --- | --- | --- | --- | --- | --- | --- | --- |
| LEAFTYPE | Leaf type | Categorical | 1. Evergreen | 2. Decidious |  |  | [1] |
| CNR | CN ratio | Categorical | 1. Low | 2. Medium | 3.High |  | [2,3] |
| COPPICEPOT | Coppice potential | Categorical | 1.Yes | 2. NO |  |  | [2,3] |
| GROWTHRATE | Growth rate | Categorical | 1. Slow | 2.moderate | 3. Rapid |  | [2,3] |
| ALLELOPATH | Known allelopath | Categorical | 1.Yes | 2. NO |  |  | [2,3] |
| NFIXATION | Nitrogen fixation | Categorical | 1. None | 2. Low. | 3. Medium | 4. High | [2,3] |
| SHAPEORIENT | Shape and orientation | Categorical | 1. Climbing | 2. Columnar | 3. Conical | 4. Decumbent | [2,3] |
|  |  |  | 5. Erect | 6. Irregular | 7. Oval | 8. Prostrate |  |
|  |  |  | 9. Rounded | 10. Semi-erect | 11. Vase |  |  |
| DROUGHTTOL | Drought tolerance | Categorical | 1. None | 2. Low. | 3. Medium | 4. High | [2,3] |
| SALINITYTOL | Salinity tolerance | Categorical | 1. None | 2. Low. | 3. Medium | 4. High | [2,3] |
| SEEDSPREAD | Seed spread rate | Categorical | 1. None | 2. Slow | 3.moderate | 4. Rapid | [2,3] |
| SHADETOL | Shade tolerance | Categorical | 1. Intolerant | 2. Intermediate | 3. Tolerant |  | [2,3 |
| SPGRVTY | Wood specific gravity (12% MC volume basis dry weight) | Continuous |  |  |  |  | [4] |
| HTMATURE | Max height at maturity (m) | Continuous |  |  |  |  | [2,3] |
| SEEDMASS | Seed mass (seeds per pound) | Continuous |  |  |  |  | [2,3] |
| ROOTDEPTH | Root length (m) | Continuous |  |  |  |  | [2,3] |

**Metadata (FIA Plots)**

Description of the following used data variables are given in FIA’s user’s manual 4 for phase 2 [1]

| PLT_CN | CONDPROP_UNADJ | DRYBIO_SAPLING |
| --- | --- | --- |
| PREV_PLT_CN | PREVCOND | DRYBIO_WDLD_SPP |
| CND_CN | SUBP | DRYBIO_BG |
| SBP_CN | TREE | SUBP_SLOPE |
| SCD_CN | STATUSCD | SUBP_ASPECT |
| TRE_CN | SPCD | FORTYPCD |
| PREV_TRE_CN | SPGRPCD | STDAGE |
| STATECD | DIA | SITECLCD |
| CYCLE | HT | SICOND |
| INVYR | CR | SIBASE |
| COUNTYCD | STOCKING | SISP |
| PLOT | CCLCD | GSSTK |
| MEASYEAR | TPA_UNADJ | GSSTKCD |
| MEASMON | VOLCFGRS | DSTRBCD1 |
| MEASDAY | VOLCFNET | TRTCD1 |
| REMPER | DRYBIO_BOLE | PHYSCLCD |
| CONDID | DRYBIO_TOP | DRYBIO_AG |
| COND_STATUS_CD | DRYBIO_STUMP |  |

**References:**

1. Woudenberg SW, Conkling BL, O’Connell BM, LaPoint EB, Turner JA, Waddell KL. The Forest Inventory and Analysis Database: Database description and users manual version 4.0 for Phase 2. 2010; Available: http://www.treesearch.fs.fed.us/pubs/37446

2. Advanced Search | USDA PLANTS [Internet]. [cited 16 Mar 2017]. Available: https://plants.usda.gov/adv_search.html

3. Fire Effects Information System [Internet]. [cited 16 Mar 2017]. Available: https://www.feis-crs.org/feis/faces/SearchByOther.xhtml

4. Miles PD, Smith BW. Specific Gravity and Other Properties of Wood and Bark for 156 Tree Species Found in North America [Internet]. Newtown square PA 19073-3294: United States Department of Agriculture Forest Service Northern Research Station; 2009 p. 39. Report No.: NRS-38. Available: http://www.nrs.fs.fed.us/pubs/rn/rn_nrs38.pdf
