# Supplementary material for: Variation in the diversity-productivity relationship in young forests of the eastern United States
Source: PLoS One. 2017 Nov 15;12(11):e0187106. doi: 10.1371/journal.pone.0187106 (PMC5687711; doi:10.1371/journal.pone.0187106)
Supplement: S4 File — (PDF) [file pone.0187106.s010.pdf]

The Forest Inventory and Analysis Database: Database Description and Users Manual Version 4.0 for Phase 2

Sharon W. Woudenberg, Barbara L. Conkling,
Barbara M. O'Connell, Elizabeth B. LaPoint,
Jeffery A. Turner, Karen L. Waddell

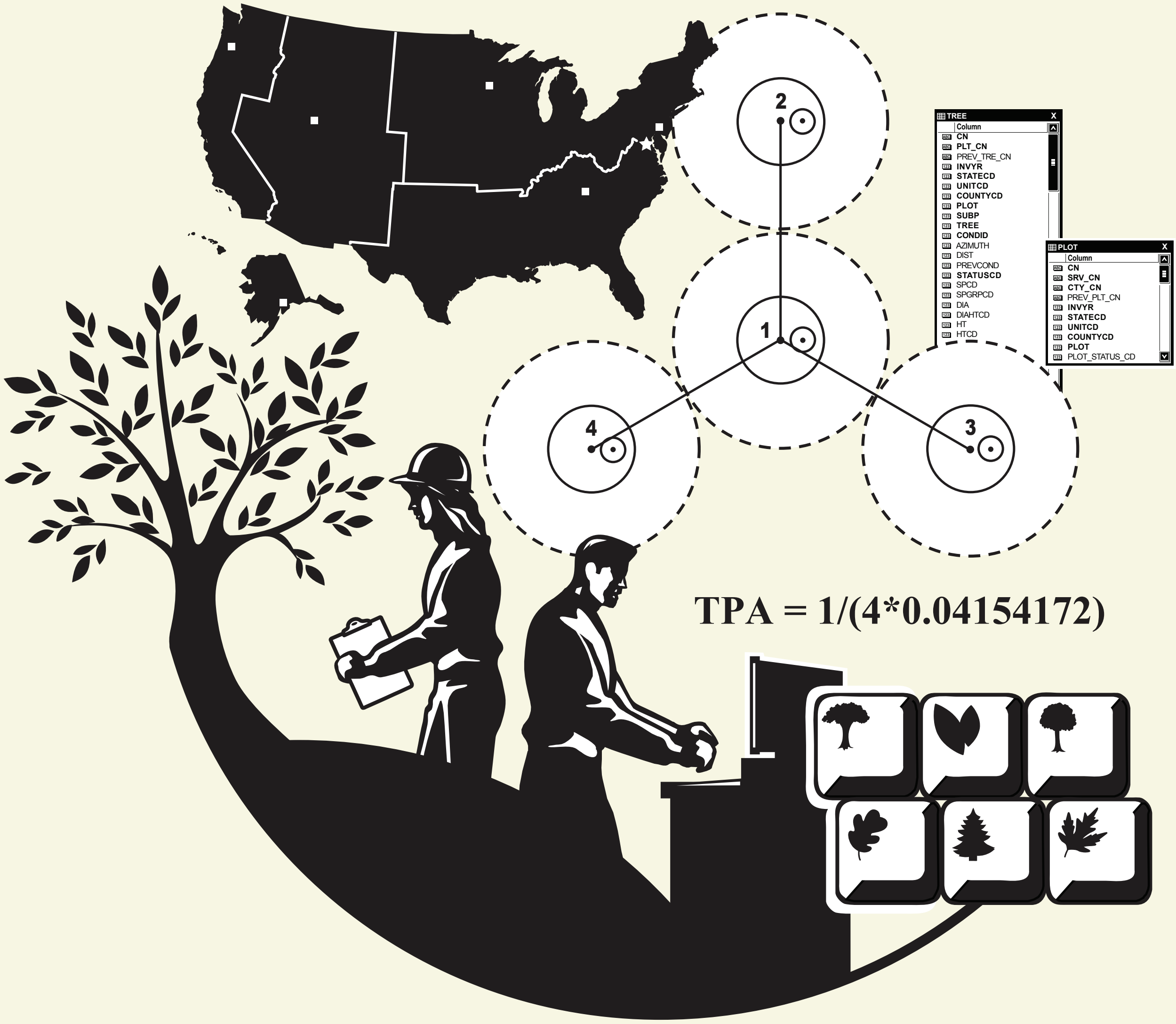

United States Department of Agriculture
Forest Service
Rocky Mountain Research Station
General Technical Report RMRS-GTR-245
December 2010

Woudenberg, Sharon W.; Conkling, Barbara L.; O'Connell, Barbara M.; LaPoint, Elizabeth B.; Turner, Jeffery A.; Waddell, Karen L. 2010. **The Forest Inventory and Analysis Database: Database description and users manual version 4.0 for Phase 2**. Gen. Tech. Rep. RMRS-GTR-245. Fort Collins, CO: U.S. Department of Agriculture, Forest Service, Rocky Mountain Research Station. 336 p.

Abstract

This document is based on previous documentation of the nationally standardized Forest Inventory and Analysis database (Hansen and others 1992; Woudenberg and Farrenkopf 1995; Miles and others 2001). Documentation of the structure of the Forest Inventory and Analysis database (FIADB) for Phase 2 data, as well as codes and definitions, is provided. Examples for producing population level estimates are also presented. This database provides a consistent framework for storing forest inventory data across all ownerships for the entire United States. These data are available to the public.

Keywords: Forest Inventory and Analysis, inventory database, users manual, monitoring

Authors

Sharon W. Woudenberg is a Supervisory Forester with the Inventory and Monitoring Program, USDA Forest Service, Rocky Mountain Research Station, Ogden, Utah.

Barbara L. Conkling is a Research Assistant Professor in the Department of Forestry and Environmental Resources, North Carolina State University, Raleigh, North Carolina.

Barbara M. O'Connell is a Forester with the Forest Inventory and Analysis Program, USDA Forest Service, Northern Research Station, Newtown Square, Pennsylvania.

Elizabeth B. LaPoint is a Forester with the Natural Resources Inventory, Monitoring and Assessment Program, Northern Research Station, Durham, New Hampshire.

Jeffery A. Turner is a Forester with the Forest Inventory and Analysis Program, USDA Forest Service, Southern Research Station, Knoxville, Tennessee.

Karen L. Waddell is a Forester with the Resource Monitoring and Assessment Program, USDA Forest Service, Pacific Northwest Research Station, Portland, Oregon.

You may order additional copies of this publication by sending your mailing information in label form through one of the following media. Please specify the publication title and number.

Publishing Services

Telephone (970) 498-1392
FAX (970) 498-1122
E-mail rschneider@fs.fed.us
Web site http://www.fs.fed.us/rmrs
Mailing Address Publications Distribution
Rocky Mountain Research Station
240 West Prospect Road
Fort Collins, CO 80526

Preface

Forest Inventory and Analysis (FIA) is a continuing endeavor mandated by Congress in the Forest and Rangeland Renewable Resources Planning Act of 1974 and the McSweeney-McNary Forest Research Act of 1928. FIA's primary objective is to determine the extent, condition, volume, growth, and depletion of timber on the Nation's forest land. Before 1999, all inventories were conducted on a periodic basis. The passage of the 1998 Farm Bill requires FIA to collect data annually on plots within each State. This kind of up-to-date information is essential to frame realistic forest policies and programs. USDA Forest Service regional research stations are responsible for conducting these inventories and publishing summary reports for individual States.

In addition to published reports, the Forest Service provides data collected in each inventory to those interested in further analysis. This report describes a standard format in which data can be obtained. This standard format, referred to as the Forest Inventory and Analysis Database (FIADB) structure, was developed to provide users with as much data as possible in a consistent manner among States. A number of inventories conducted prior to the implementation of the annual inventory are available in the FIADB. However, various data attributes may be empty or the items may have been collected or computed differently. Annual inventories use a common plot design and common data collection procedures nationwide, resulting in greater consistency among FIA work units than earlier inventories. Data field definitions note inconsistencies caused by different sampling designs and processing methods.

Acknowledgments

In addition to those listed as authors, the following people provided additional contributions to this document:

George Breazeale, Computer Specialist, USDA Forest Service, Pacific Northwest Research Station, Portland, Oregon. Brian Cordova, Computer Programmer/Analyst, Harry Reid Center for Environmental Studies, University of Nevada-Las Vegas, Las Vegas, Nevada. Joseph Donnegan, Supervisory Biological Scientist, USDA Forest Service, Pacific Northwest Research Station, Portland, Oregon. Mark Hansen, Research Forester (retired), Forest Inventory and Analysis Program, USDA Forest Service, Northern Research Station, Saint Paul, Minnesota (currently Research Associate, Department of Forest Resources, University of Minnesota, Saint Paul, Minnesota). Jason R. Meade, Forester, USDA Forest Service, Southern Research Station, Knoxville, Tennessee. James Menlove, Ecologist, USDA Forest Service, Rocky Mountain Research Station, Ogden, Utah. Patrick Miles, Research Forester, USDA Forest Service, Northern Research Station, Saint Paul, Minnesota. Scott A. Pugh, Forester, USDA Forest Service, Northern Research Station, Houghton, Michigan. Ted Ridley, IT Specialist, USDA Forest Service, Southern Research Station, Knoxville, Tennessee. John D. Shaw, Biological Scientist, USDA Forest Service, Rocky Mountain Research Station, Inventory and Monitoring Program, Ogden, Utah.

Research support was provided by the USDA Forest Service Research Stations listed above and in part through the Research Joint Venture Agreements and 07-JV-11330146-134, 08-JV-11330146-078, and 09-JV-11330146-087 between the U.S. Department of Agriculture, Forest Service, Southern Research Station, and North Carolina State University.

The use of trade or firm names in this publication is for reader information and does not imply endorsement by the U.S. Department of Agriculture of any product or service.

Contents

Preface i
Acknowledgments i

Chapter 1 — Introduction 1
- Purpose of this Manual 1
- The FIA Program 2
- The FIA Database 3
- Changes From the Previous Database Version 4

Chapter 2 — FIA Sampling and Estimation Procedures 8
- Sampling and Stratification Methodology 8
- Plot Location 8
- Plot Design, Condition Delineation, and Types of Data Attributes 10
- Types of Attributes 11
- Expansion factors 13
- Accuracy Standards 14

Chapter 3 — Database Structure 16
- Table Descriptions 16
- Keys Presented with the Tables 22
- Survey Table (Oracle table name is SURVEY) 24
- County Table (Oracle table name is COUNTY) 28
- Plot Table (Oracle table name is PLOT) 30
- Condition Table (Oracle table name is COND) 43
- Subplot Table (Oracle table name is SUBPLOT) 73
- Subplot Condition Table (Oracle table name is SUBP_COND) 80
- Tree Table (Oracle table name is TREE) 84
- Seedling Table (Oracle table name is SEEDLING) 119
- Site Tree Table (Oracle table name is SITETREE) 124
- Boundary Table (Oracle table name is BOUNDARY) 130
- Subplot Condition Change Matrix (Oracle table name is SUBP_COND_CHNG_MTRX) 135
- Tree Regional Biomass Table (Oracle table name is TREE_REGIONAL_BIOMASS) 139
- Population Estimation Unit Table (Oracle table name is POP_ESTN_UNIT) 141
- Population Evaluation Table (Oracle table name is POP_EVAL) 144
- Population Evaluation Attribute Table (Oracle table name is POP_EVAL_ATTRIBUTE) 147
- Population Evaluation Group Table (Oracle table name is POP_EVAL_GRP) 149
- Population Evaluation Type Table (Oracle table name is POP_EVAL_TYP) 153
- Population Plot Stratum Assignment Table (Oracle table name is POP_PLOT_STRATUM_ASSGN) 155
- Population Stratum Table (Oracle table name is POP_STRATUM) 159
- Reference Population Attribute Table (Oracle table name is REF_POP_ATTRIBUTE) 163

Reference Population Evaluation Type Description Table (Oracle table name is REF_POP_EVAL_TYP_DESCR) 165
Reference Forest Type Table (Oracle table name is REF_FOREST_TYPE) 167
Reference Species Table (Oracle table name is REF_SPECIES) 169
Reference Species Group Table (Oracle table name is REF_SPECIES_GROUP) 184
Reference Habitat Type Description Table (Oracle table name is REF_HABTYP_DESCRIPTION) 186
Reference Habitat Type Publication Table (Oracle table name is REF_HABTYP_PUBLICATION) 188
Reference Citation Table (Oracle table name is REF_CITATION) 190
Reference Forest Inventory and Analysis Database Version Table (Oracle table name is REF_FIADB_VERSION) 192
Reference State Elevation Table (Oracle table name is REF_STATE_ELEV) 194
Reference Unit Table (Oracle table name is REF_UNIT) 196

Chapter 4 — Calculating Population Estimates and Their Associated Sampling Errors 198

Literature Cited 241
Appendix A. Index of Column Names 243
Appendix B. Forest Inventory and Analysis (FIA) Plot Design Codes and Definitions by FIA Work Unit. 259
Appendix C. State, Survey Unit, and County Codes 264
Appendix D. Forest Type Codes and Names 306
Appendix E. Administrative National Forest Codes and Names 310
Appendix F. Tree Species Codes, Names, and Occurrences 313
Appendix G. Tree Species Group Codes 322
Appendix H. Damage Agent codes for PNW 323
Appendix I. FIA Inventories by State, Year, and Type 328
Appendix J. Biomass Estimation in the FIADB 330

Chapter 1 -- Introduction

Purpose of This Manual

This manual is the definitive guide to the Forest Inventory and Analysis database (FIADB). This document replaces General Technical Report NC-218 (Miles and others 2001), which covered version 1.0 of the FIADB, and subsequent updates that appeared as online documentation to the FIADB through version 3.0. Although it is used widely within the Forest Inventory and Analysis (FIA) program, a substantial part, if not the majority, of the intended audience includes those outside FIA who are interested in using FIA data for their own analyses. Awareness of the potential uses of FIA data by users outside the FIA community is growing, and the data become increasingly useful as additional attributes are collected. However, as is the case with any data source, it is incumbent upon the user to understand not only the data definitions and acquisition methods, but also the context in which the data were collected. This manual is intended to help current and potential users understand the necessary details of the FIADB.

This manual has four chapters. The remainder of chapter 1 includes general introductions to the FIA program and the FIA database, including brief histories of both. It provides a convenient overview for those who have an interest in using FIA data, but have not yet become familiar with the FIA program. Chapter 2 provides descriptions of FIA sampling methods, including plot location and design, data measurement and computation, and general estimation procedures. Chapter 3 describes the tables that comprise the database, the attributes stored in each table, and the linkages between tables. Descriptions of the attributes, their data format, valid values, and other important details are given, but the appropriate field manuals should be consulted for exact specifications regarding data collection methods. Users with a good understanding of chapter 3 and fundamental database management skills should be able to conduct a wide range of analyses. Chapter 4 explains the standard methods used to compile population-level estimates from FIADB, and applies the new estimation procedures documented by Bechtold and Patterson (2005). These procedures are based on adoption of the annual inventory system and the mapped plot design, and constitute a major change when compared to previous compilation procedures. However, the new compilation procedures should allow more flexible analyses, especially as additional panels are completed under the annual inventory system.

There are several conventions used in this manual. The names of attributes (i.e., columns within tables) and table names appear in capital letters (e.g., PLOT table). Some attribute names appear in two or more tables. In most cases, such as the State code (STATECD), the attribute has the same definition in all tables. However, there are situations where attributes with the same name are defined differently in each table. One such example is the VALUE attribute in the REF_FOREST_TYPE table, which is used to identify the forest type and refers to appendix D. However, the VALUE attribute in the REF_UNIT table is used to indicate the FIA survey unit identification number from appendix C. In most cases, such as in the table descriptions in chapter 3, the attribute name will be used alone and the affiliation with a particular table is implied by the context. In cases where an attribute name has a different meaning in two or more tables, a compound naming convention, using the table name followed by the attribute name, will be used. In the VALUE attribute example, the name REF_FOREST_TYPE.VALUE refers to the VALUE

attribute in the REF_FOREST_TYPE table, while REF_UNIT.VALUE refers to the VALUE attribute in the REF_UNIT table.

The FIA Program

The FIA program is mandated by Congress in the Forest and Rangeland Renewable Resources Planning Act of 1974 and the McSweeney-McNary Forest Research Act of 1928. The mission of FIA is to determine the extent, condition, volume, growth, and depletions of timber on the Nation's forest land. FIA is the only program that collects, publishes, and analyzes data from all ownerships of forest land in the United States (Smith 2002). Throughout the 80-year history of the program, inventories have been conducted by a number of geographically dispersed FIA work units. Currently, the national FIA program is implemented by four regionally distributed work units that are coordinated by a National Office in Washington, DC (see figure 1). The four FIA work units are named by the Research Station in which they reside. Station abbreviations are used within this document and they are defined as Pacific Northwest Research Station (PNWRS), Northern Research Station (NRS), Rocky Mountain Research Station (RMRS), and Southern Research Station (SRS). NRS was recently formed from the merger of North Central Research Station (NCRS) and Northeastern Research Station (NERS). Some data items still retain these designations.

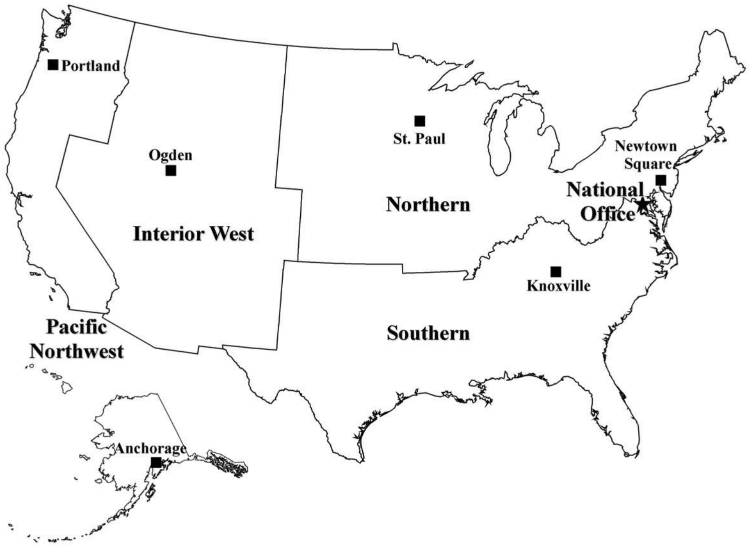

Figure 1. Boundaries of the four regionally distributed FIA work units and locations of program offices.

Starting in 1929, FIA accomplished its mission by conducting periodic forest inventories on a State-by-State basis. With the completion of Arizona, New Mexico, and Nevada in 1962, all 48 coterminous States had at least one periodic inventory (Van Hooser and others 1993). Repeat intervals for inventorying individual States have varied widely. By the late 1990s, most States had been inventoried more than once under the periodic inventory system; however, not all periodic data are available in electronic form (appendix I lists all periodic data available in the FIADB and the year in which annual inventory began).

With the passage of the 1998 Farm Bill, the FIA program was required to move from a periodic inventory to an annualized system, with a portion of all plots within a State measured each year (Gillespie 1999). Starting in 1999, States were phased into the annual inventory system (appendix I). At the time of publication of this document, annual inventory has not yet been started in Wyoming and Interior Alaska. Although the 1998 Farm Bill specified that 20 percent of the plots within each State would be visited annually, funding limitations have resulted in the actual portion of plots measured annually ranging between 10 and 20 percent, depending on the State.

Periodic and annual data are analyzed to produce reports at State, regional, and national levels. In addition to published reports, data are made available to the public for those who are interested in conducting their own analyses. Downloadable data, available online at http://fia.fs.fed.us/tools-data/, follow the format described in this document. Also available at this site are tools to make population estimates. The web-based EVALIDator tool or the Forest Inventory Data Online (FIDO) tool provide interactive access to the FIADB.

The FIA Database

The Forest Inventory and Analysis Database (FIADB) was developed to provide users with data in a consistent format, spanning all States and inventories. The first version of FIADB replaced two FIA regional databases; the Eastern States (Eastwide database) documented by Hansen and others (1992), and Western States (Westwide database) documented by Woudenberg and Farrenkopf (1995). A new national plot design (see chapter 2) provided the impetus for replacing these two databases, and FIA work units adopted the new design in all State inventories initiated after 1998. The FIADB table structure is currently derived from the National Information Management System (NIMS), which was designed to process and store annual inventory data. This is the fourth version of the single national FIA database to be released. A number of changes in the FIADB structure have been made to accommodate the data processing and storage requirements of NIMS. As a result, data from periodic inventories are stored in a format consistent with annual inventory data.

FIADB files are available for periodic inventory data collected as early as 1977 (see appendix I). A wide variety of plot designs and regionally defined attributes were used in periodic inventories, often differing by State. Because of this, some data attributes may not be populated or certain data may have been collected or computed differently. During some periodic inventories, ground plot data were collected on nonreserved timberland only. Low productivity forest land, reserved (areas reserved from timber harvesting), and nonforested areas usually were not ground sampled. To account for the total area of a State, "place holder" plots were created to represent these nonsampled areas, which are identified by plot design code 999 in FIADB (PLOT.DESIGNCD = 999). For these plots, many attributes that are normally populated for forested plots will be blank. Users should be aware that while place holder plots account for the area of nonsampled forest land, they do not account for the corresponding forest attributes (such as volume, growth, or mortality) that may exist in those areas.

Annual inventories, initiated sometime after 1999 depending on the State, use a nationally standardized plot design and common data collection procedures resulting in greater consistency among FIA work units than earlier inventories. However, as part of a continuing effort to improve the inventory, some changes in methodology and attribute definitions have been implemented after the new design was put into practice. Beginning in 1998, FIA started using a National Field Guide referenced as Field Guide 1.0. The database contains an attribute labeled MANUAL that stores the

version number of the field guide under which the data were collected. When both the plot design is coded as being the national design (PLOT.DESIGNCD = 1) and the field guide is coded with a number greater than or equal to 1, certain attributes are defined as being "core" while others are allowed to be "core optional." Core attributes must be collected by every FIA work unit, using the same definition and set of codes. In contrast, collection of core optional attributes are decided upon by individual FIA work units, using the same national protocol, predefined definition, and set of codes. Many attributes, regardless of whether or not they are core or core optional, are only populated for forested conditions, and are blank for other conditions (such as nonforest or water). Attributes described in chapter 3 are noted if they are core optional.

Users who wish to analyze data using aggregations of multiple State inventories or multiple inventories within States should become familiar with changes in methodology and attribute definitions (see chapters 2 and 3). For each attribute in the current version of FIADB, an effort has been made to provide the current definition of the attribute, as well as any variations in definition that may have been used among various FIA work units. In other words, although inventory data have been made available in a common data format, users should be aware of differences that might affect their analyses.

Changes From the Previous Database Version

Database users should also be aware that changes are made for each version of FIADB. Sometimes the changes are minimal, such as simply rewriting explanatory text for clarification or adding new codes to a particular attribute. Database tables and/or attributes may be added or removed. In this release (4.0), a number of reference tables have been added. Also, two tables were added to modify the way population estimates are handled. Another important table addition is the Subplot Condition Change Matrix table that tracks changes in any condition class attribute between two visits to a plot. In appendix F, several changes were made in the SPGRPCD column. Tables 1-5 summarize the major modifications to FIADB Version 4.0.

Table 1. Database entire tables added in FIADB V4.0

Name of table added	Table description
SUBP_COND_CHNG_MTRX	Subplot Condition Change Matrix
TREE_REGIONAL_BIOMASS	Tree Regional Biomass
POP_EVAL_TYP	Population Evaluation Type
REF_POP_EVAL_TYP_DESCR	Reference Population Evaluation Type Description
REF_FOREST_TYPE	Reference Forest Type
REF_SPECIES	Reference Species
REF_SPECIES_GROUP	Reference Species Group
REF_HABTYP_DESCRIPTION	Reference Habitat Type Description
REF_HABTYP_PUBLICATION	Reference Habitat Type Publication
REF_CITATION	Reference Citation
REF_FIADB_VERSION	Reference Forest Inventory and Analysis Database Version
REF_STATE_ELEV	Reference State Elevation
REF_UNIT	Reference Unit

Table 2. Database table attribute additions in FIADB V4.0

Name of table affected	Name of column added to table
SURVEY	RSCD
SURVEY	ANN_INVENTORY
PLOT	INTENSITY
PLOT	NF_SAMPLING_STATUS_CD
PLOT	NF_PLOT_STATUS_CD
PLOT	NF_PLOT_NONSAMPLE_REASN_CD
PLOT	P2VEG_SAMPLING_STATUS_CD
PLOT	P2VEG_SAMPLING_LEVEL_DETAIL_CD
PLOT	INVASIVE_SAMPLING_STATUS_CD
PLOT	INVASIVE_SPECIMEN_RULE_CD
COND	CARBON_DOWN_DEAD
COND	CARBON_LITTER
COND	CARBON_SOIL_ORG
COND	CARBON_STANDING_DEAD
COND	CARBON_UNDERSTORY_AG
COND	CARBON_UNDERSTORY_BG
COND	HARVEST_TYPE1_SRS
COND	HARVEST_TYPE2_SRS
COND	HARVEST_TYPE3_SRS
COND	NF_COND_STATUS_CD
COND	NF_COND_NONSAMPLE_REASN_CD
COND	CANOPY_CVR_SAMPLE_METHOD_CD
COND	LIVE_CANOPY_CVR_PCT
COND	LIVE_MISSING_CANOPY_CVR_PCT
COND	NBR_LIVE_STEMS
SUBPLOT	NF_SUBP_STATUS_CD
SUBPLOT	NF_SUBP_NONSAMPLE_REASN_CD
SUBPLOT	P2VEG_SUBP_STATUS_CD
SUBPLOT	P2VEG_SUBP_NONSAMPLE_REASN_CD
SUBPLOT	INVASIVE_SUBP_STATUS_CD
SUBPLOT	INVASIVE_NONSAMPLE_REASN_CD
TREE	DRYBIO_BOLE
TREE	DRYBIO_TOP
TREE	DRYBIO_STUMP
TREE	DRYBIO_SAPLING
TREE	DRYBIO_WDLD_SPP
TREE	DRYBIO_BG
TREE	CARBON_AG
TREE	CARBON_BG
TREE	PREV_PNTN_SRS
POP_ESTN_UNIT	P1SOURCE

Name of table affected	Name of column added to table
POP_EVAL	START_INVYR
POP_EVAL	END_INVYR
POP_EVAL_ATTRIBUTE	CN
POP_EVAL_ATTRIBUTE	STATECD
POP_EVAL_GRP	NOTES
REF_POP_ATTRIBUTE	CN
REF_POP_ATTRIBUTE	FOOTNOTE

Table 3. Database table attribute deletions in FIADB V4.0

Name of table affected	Name of column deleted
PLOT	CREW_TYPE
PLOT	MANUAL_DB
PLOT	REPLACED_PLOT_NBR
PLOT	LAST_INVYR_MEASURED
COND	TRTOPCD
COND	PASTNFCD
COND	DISTANCE_WATER_SRS
TREE	PREVSUBC
SITETREE	SITREE_EQU_NO_PNWRS

Table 4. Database table attributes renamed in FIADB V4.0

Name of table affected	Old attribute name	New attribute name
PLOT	GROWCD	GROW_TYP_CD
PLOT	MORTCD	MORT_TYP_CD
COND	TRTCD1_SRS	HARVEST_TYPE1_SRS
COND	TRTCD2_SRS	HARVEST_TYPE2_SRS
COND	TRTCD3_SRS	HARVEST_TYPE3_SRS
SUBPLOT	STATUSCD	SUBP_STATUS_CD
SITETREE	COND_CLASS_LIST	CONDLIST

Table 5. Database table attributes moved to another table in FIADB V4.0

Original table	New table	Column moved and renamed
TREE	TREE_REGIONAL_BIOMASS	REGIONAL_DRYBIOT
TREE	TREE_REGIONAL_BIOMASS	REGIONAL_DRYBIOM

A change was made in the stocking equation assignment for various tree species and was applied to all annual inventory plot data. This change can result in a different computed forest type for a given plot. Several new forest types have been added and some changes were made in the way forest types are grouped.

Another significant change relates to biomass and carbon. FIA adopted a standard methodology to compute biomass of various tree components, which are used to convert biomass to carbon estimates. Previous biomass estimates, which were derived using a variety of equations, have been moved to a new table called TREE_REGIONAL_BIOMASS. Users can choose which attribute to summarize and can make comparisons between the estimates derived from the different methodologies.

Modeled condition level carbon attributes have been added to the FIADB and can be used to obtain results similar to those found in the U.S. Environmental Protection Agency's (EPA's) Greenhouse Gas Inventory (http://epa.gov/climatechange/emissions/).

Chapter 2 -- FIA Sampling and Estimation Procedures

To use the FIADB effectively, users should acquire a basic understanding of FIA sampling and estimation procedures. Generally described, FIA uses what may be characterized as a three-phase sampling scheme. Phase 1 (P1) is used for stratification, while Phase 2 (P2) consists of plots that are visited or photo-interpreted. A subset of Phase 2 plots are designated as Phase 3 (P3) plots (formerly known as Forest Health Monitoring (FHM) plots) where additional health indicator attributes are collected. Phases 1 and 2 are described in this chapter, but Phase 3 is described in a separate user's manual (Woodall and others 2010). The exception is P3 crown attributes, which are described in the TREE table of this document.

Sampling and Stratification Methodology

Remote Sensing (P1)
The basic level of inventory in the FIA program is the State, which begins with the interpretation of a remotely sensed sample, referred to as Phase 1 (P1). The intent of P1 is to classify the land into various remote sensing classes for the purpose of developing meaningful strata. A stratum is a group of plots that have the same or similar remote sensing classifications. Stratification is a statistical technique used by FIA to aggregate Phase 2 ground samples into groups to reduce variance when stratified estimation methods are used. The total area of the estimation unit is assumed to be known.

Each Phase 2 ground plot is assigned to a stratum and the weight of the stratum is based on the proportion of the stratum within the estimation unit. Estimates of population totals are then based on the sum of the product of the known total area, the stratum weight, and the mean of the plot level attribute of interest for each stratum. The expansion factor for each stratum within the estimation unit is the product of the known total area and the stratum weight divided by the number of Phase 2 plots in the stratum.

Selection criteria for remote sensing classes and computation of area expansion factors differ from State to State. Users interested in the details of how these expansion factors are assigned to ground plots for a particular State should contact the appropriate FIA work unit (see table 6).

Ground Sampling (P2)
FIA ground plots, or Phase 2 plots, are designed to cover a 1-acre sample area; however, not all trees on the acre are measured. Ground plots may be new plots that have never been measured, or re-measurement plots that were measured during one or more previous inventories. Recent inventories use a nationally standard, fixed-radius plot layout for sample tree selection (see figure 2). Various arrangements of fixed-radius and variable-radius (prism) subplots were used to select sample trees in older inventories.

Plot Location

The FIADB includes coordinates for every plot location in the database, whether it is forested or not, but these are not the precise locations of the plot centers. In an amendment to the Food Security Act of 1985 (reference 7 USC 2276 § 1770), Congress directed FIA to ensure the privacy of private landowners. Exact plot coordinates could be used in conjunction with other publicly available data to link plot data to specific landowners, in violation of requirements set by Congress. In addition to

the issue of private landowner privacy, the FIA program had concerns about plot integrity and vandalism of plot locations on public lands. A revised policy has been implemented and methods for making approximate coordinates available for all plots have been developed. These methods are collectively known as "fuzzing and swapping" (Lister and others 2005).

In the past, FIA provided approximate coordinates for its periodic data in the FIADB. These coordinates were within 1.0 mile of the exact plot location (this is called fuzzing). However, because some private individuals own extensive amounts of land in certain counties, the data could still be linked to these owners. In order to maintain the privacy requirements specified in the amendments to the Food Security Act of 1985, up to 20 percent of the private plot coordinates are swapped with another similar private plot within the same county (this is called swapping). This method creates sufficient uncertainty at the scale of the individual landowner such that privacy requirements are met. It also ensures that county summaries and any breakdowns by categories, such as ownership class, will be the same as when using the true plot locations. This is because only the coordinates of the plot are swapped – all the other plot characteristics remain the same. The only difference will occur when users want to subdivide a county using a polygon. Even then, results will be similar because swapped plots are chosen to be similar based on attributes such as forest type, stand-size class, latitude, and longitude (each FIA work unit has chosen its own attributes for defining similarity).

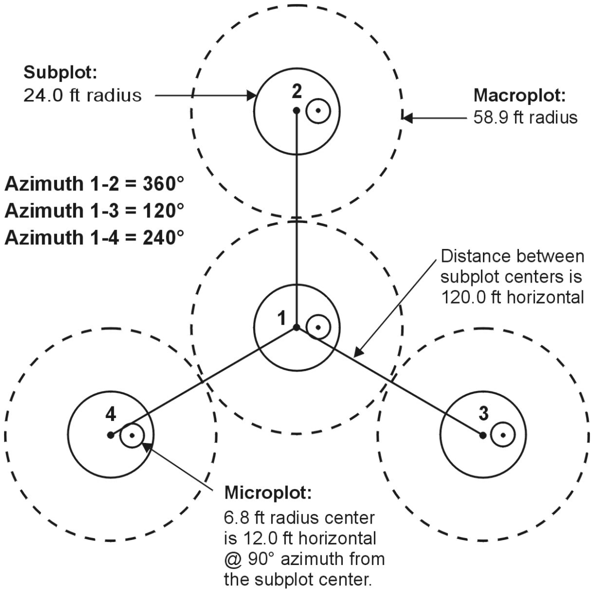

Figure 2. The FIA mapped plot design. Subplot 1 is the center of the cluster with subplots 2, 3, and 4 located 120 feet away at azimuths of 360°, 120°, and 240°, respectively.

For plot data collected under the current plot design, plot numbers are reassigned to sever the link to other coordinates stored in the FIADB prior to the change in the law. Private plots are also swapped using the method described above; remeasured plots are swapped independent of the periodic data. All plot coordinates are fuzzed, but less than before – within 0.5 mile for most plots and up to 1.0 mile on a small subset of them. This was done to make it difficult to locate the plot on the ground, while maintaining a good correlation between the plot data and map-based characteristics.

For most user applications, such as woodbasket analyses and estimates of other large areas, fuzzed and swapped coordinates provide a sufficient level of accuracy. However, some FIA customers require more precision of plot locations in order to perform analyses by user-defined polygons and for relating FIA plot data to other map-based information, such as soils maps and satellite imagery. In order to accommodate this need, FIA provides spatial data services that allow most of the desired analyses while meeting privacy requirements. The possibilities and limitations for these types of analyses are case-specific, so interested users should contact their local FIA work unit for more information.

Plot Design, Condition Delineation, and Types of Data Attributes

Plot Designs

The current national standard FIA plot design was originally developed for the Forest Health Monitoring program (Scott and others 1993). It was adopted by FIA in the mid-1990s and used for the last few periodic inventories and all annual inventories. The standard plot consists of four 24.0-foot radius subplots (approximately 0.0415 or 1/24 acre) (see figure 2), on which trees 5.0 inches and greater in diameter are measured. Within each of these subplots is nested a 6.8-foot radius microplot (approximately 1/300th acre) on which trees smaller than 5.0 inches in diameter are measured. A core optional variant of the standard design includes four "macroplots," each with a radius of 58.9 feet (approximately 1/4 acre) that originate at the centers of the 24.0-foot radius subplots. Breakpoint diameters between the 24-foot radius subplots and the macroplots vary and are specified in the macroplot breakpoint diameter attribute (PLOT.MACRO_BREAKPOINT_DIA).

Prior to adoption of the current plot design, a wide variety of plot designs were used. Periodic inventories might include a mixture of designs, based on forest type, ownership, or time of plot measurement. In addition, similar plot designs (e.g., 20 BAF variable-radius plots) might have been used with different minimum diameter specifications (e.g., 1-inch versus 5-inch). Details on these designs are included in appendix B (plot design codes).

Conditions

An important distinguishing feature between the current plot design and previous designs is that different conditions are "mapped" on the current design (see figure 3). In older plot designs, adjustments were made to the location of the plot center or the subplots were rearranged such that the entire plot sampled a single condition. In the new design, the plot location and orientation remains fixed, but boundaries between conditions are mapped and recorded. Conditions are defined by changes in land use or changes in vegetation that occur along more-or-less distinct boundaries. Reserved status, owner group, forest type, stand-size class, regeneration status, and stand density are used to define forest conditions. For example, the subplots may cover forest and nonforest areas, or it may cover a single forested area that can be partitioned into two or more distinct stands. Although mapping is used to separate forest and nonforest conditions, different nonforest conditions occurring on a plot are not mapped during initial plot establishment. Each condition occurring on the plot is assigned a condition proportion, and all conditions on a plot add up to 1.0. For plot designs other than the mapped design, condition proportion is always equal to 1.0 in FIADB.

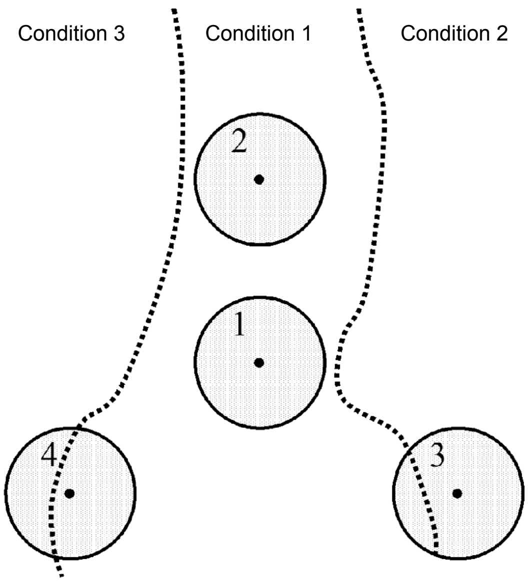

Figure 3. The FIA mapped plot design. Subplot 1 is the center of the cluster with subplots 2, 3, and 4 located 120 feet away at azimuths of 360°, 120°, and 240°, respectively. When a plot straddles two or more conditions, the plot area is divided by condition.

Types of Attributes

Measured, Assigned, and Computed Attributes

In addition to attributes that are collected in the field, FIADB includes attributes that are populated in the office. Examples of field attributes include tree diameter and height, and slope and aspect of the plot and subplot. Attributes that are populated in the office include assigned attributes, such as county and owner group codes, or computed attributes, such as tree and area expansion factors, and tree volumes.

For measured attributes, this document provides only basic information on the methodology used in the field. The authoritative source for methodology is the Forest Inventory and Analysis National Core Field Guide used during the inventory in which the data were collected (see http://www.fia.fs.fed.us/library/field-guides-methods-proc/) . The MANUAL attribute in the PLOT table documents the version number where data collection protocols can be found.

Values of attributes that are assigned in the office are determined in several ways, depending on the attribute. For example, ownership may be determined using geographic data or local government records. Other attributes, such as Congressional District and Ecological Subsection are assigned values based on data management needs.

Some computed attributes in the database are derived using other attributes in the database. Ordinarily, such attributes would not be included in a database table because they could be

computed using the supplied attributes. However, some data compilation routines are complex or vary within or among FIA work units, so these computed attributes are populated for the convenience of database users.

One example of a computed attribute is site index, which is computed at the condition level. Site index is generally a function of height and age, although other attributes may be used in conjunction. In addition, several different site index equations may be available for a species within its range. Height and age data are included in the TREE table, but only certain trees (see SITETREE table) are included in the site index attribute that is reported for the condition. As a result, it would be time-consuming for users to replicate the process required to calculate site index at the condition level. For convenience, the condition (COND) table includes site index (SICOND), the species for which it is calculated (SISP), and the site index base age (SIBASE).

In most cases computed attributes should be sufficient for users' needs, because the equations and algorithms used to compute them have been determined by the FIA program to be the best available for the plot location. However, for most computed attributes the relevant tree and plot level attributes used to compute them are included in the database, so users may do their own calculations if desired.

Regional Attributes

A number of regionally specific attributes are available in FIADB. These regional attributes are identified by FIA work unit, both in the table structure description (e.g., the attribute is named with an extension such as NERS) and in the attribute description (e.g., the attribute description text contains the phrase "Only collected by…"). For specific questions about the data from a particular FIA work unit, please contact the individuals listed in table 6.

Table 6. Contacts at individual FIA work units

FIA Work Unit	RSCD	States	Database Contact	Phone	Analyst Contact	Phone
Rocky Mountain (RMRS)	22	AZ,CO,ID,MT,NV, NM,UT,WY	Mark Rubey	801-625-5647	John Shaw	801-625-5673
North Central (NCRS)*	23	IL,IN,IA,KS,MI,MN, MO,NE,ND,SD,WI	Carol Alerich	610-557-4068	Will McWilliams	610-557-4050
Northeastern (NERS)*	24	CT,DE,ME,MD,MA, NH,NJ,NY,OH,PA, RI, VT,WV	Carol Alerich	610-557-4068	Will McWilliams	610-557-4050
Pacific Northwest (PNWRS)	26,27	AK,CA,HI,OR,WA	Ron Wanek	503-808-2048	Karen Waddell	503-808-2046
Southern (SRS)	33	AL,AR,FL,GA,KY, LA,MS,NC,OK,SC, TN,TX,VA, PR, VI	Jeff Turner	865-862-2053	Tom Brandeis	865-862-2030

*The North Central Research Station (NCRS) and the Northeastern Research Station (NERS) have merged to become one research station, the Northern Research Station. The former regional designations are kept to accommodate the data.

More information on attribute types is included in chapter 3.

Expansion Factors

Tree Expansion Factors

The expansion factor(s) used to scale each tree on a plot to a per-acre basis is dependent on the plot design. For fixed-plot designs, scaling is straightforward, with the number of trees per acre (TPA) represented by one tree equal to the inverse of the plot area in acres. The general formula is shown by equation [1]:

[1] $TPA = 1/(N*A)$

Where N is the number of subplots, and
A is the area of each subplot.

For example, the TPA of each tree ≥5.0 inches in diameter occurring on the current plot design would be calculated using equation [2]:

[2] $TPA = 1/(4*0.04154172) = 6.018046$

This expansion factor can be found in the TPA_UNADJ attribute in the TREE table (see chapter 3) for plots measured with the annual plot design. In variable-radius plot designs, the per-acre expansion factor is determined by the diameter of the tree, the basal area factor (BAF), and the number of subplots used in the plot design. The general formula is shown by equation [3]:

[3] $TPA = (BAF / 0.005454*DIA^2)/N$

Where BAF is the variable-radius basal area factor in square feet,
DIA is diameter of the tally tree in inches, and
N is the number of subplots in the plot design.

For example, if a 11.5-inch tree is tallied using a 10 BAF prism on a variable-radius design plot that uses five subplots, the calculation is:

[4] $TPA = (10 / 0.005454*11.5^2)/5 = 2.773$

A 5.2-inch tree will have a greater expansion factor:

[5] $TPA = (10 / 0.005454*5.2^2)/5 = 13.562$

Although it is not necessary to calculate expansion factors for different plot designs because they are stored in TPA_UNADJ, information on plot design can be found by using the code from the DESIGNCD attribute in the PLOT table to look up the plot design specifications in appendix B.

Plot Area Expansion Factors

Some previous versions of FIADB have included area expansion factors in the PLOT table that were used to scale plot-level data to population-level estimates (see EXPCURR and related attributes in Miles and others 2001). In this version of FIADB, area expansion factors have been removed from the PLOT table. Instead, there is one area expansion factor (EXPNS) stored in the POP_STRATUM table. This change is needed because of the way annual inventory data are compiled. Under the annual inventory system, new plots are added each year. Adjustment factors that are used to compensate for denied access, inaccessible, and other reasons for not sampling may

differ each time new data replaces older data. Both the number of acres each plot represents and the adjustments for the proportion of plots not sampled may change each year. In order to allow users to obtain population estimates for any grouping of data, an adjustment factor has been calculated and stored for each set of data being compiled. There is a separate adjustment factor for each fixed plot size: microplot, subplot, and macroplot. These attributes are also stored in the POP_STRATUM table. Each time the data are stratified differently, the adjustments and expansion factor may change. Therefore, FIA provides a different expansion factor every time the data are restratified.

FIA has chosen the term 'evaluation' to describe this process of storing different stratifications of data either for an individual set of data or for the changing sets of data through time. Each aggregation of data is given an evaluation identifier (EVALID). The user can select population estimates for the most current set of data or for previous sets of data. In addition to being able to calculate population estimates, users can now calculate sampling error information because FIA is storing all of the Phase 1 information used for the stratification. That information is stored for each estimation unit, which is usually a geographic subset of the State (see the POP_ESTN_UNIT table). For more information about evaluations and calculation of area expansion factors, see chapter 4.

A different method of population estimation is being implemented in this version of FIADB. In FIADB 3.0, users would select the appropriate evaluation sequence number (EVAL_CN_FOR_xxx) from the POP_EVAL_GRP table. This evaluation sequence number allowed them to select the appropriate plots and associated expansions. The newly added POP_EVAL_TYP table allows users to perform the similar queries, retrieving the same information, and will allow for a variety of evaluations to be added in the future. The previous method will continue to work in version 4.0.

Accuracy Standards

Forest inventory plans are designed to meet sampling error standards for area, volume, growth, and removals provided in the Forest Service directive (FSH 4809.11) known as the Forest Survey Handbook (U.S. Department of Agriculture 2008). These standards, along with other guidelines, are aimed at obtaining comprehensive and comparable information on timber resources for all parts of the country. FIA inventories are commonly designed to meet the specified sampling errors at the State level at the 67 percent confidence limit (one standard error). The Forest Survey Handbook mandates that the sampling error for area cannot exceed 3 percent error per 1 million acres of timberland. A 5 percent (Eastern United States) or 10 percent (Western United States) error per 1 billion cubic feet of growing-stock on timberland is applied to volume, removals, and net annual growth. Unlike the mandated sampling error for area, sampling errors for volume, removals, and growth are only targets.

FIA inventories are extensive inventories that provide reliable estimates for large areas. As data are subdivided into smaller and smaller areas, such as a geographic unit or a county, the sampling errors increase and the reliability of the estimates goes down.

- A State with 5 million acres of timberland would have a maximum allowable sampling error of 1.3 percent (3% x $(1{,}000{,}000)^{0.5}$ / $(5{,}000{,}000)^{0.5}$).
- A geographic unit within that State with 1 million acres of timberland would have a 3.0 percent maximum allowable sampling error (3% x $(1{,}000{,}000)^{0.5}$ / $(1{,}000{,}000)^{0.5}$).
- A county within that State with 100 thousand acres would have a 9.5 percent maximum allowable sampling error (3% x $(1{,}000{,}000)^{0.5}$ / $(100{,}000)^{0.5}$) at the 67 percent confidence level.

The greater allowance for sampling error in smaller areas reflects the decrease in sample size as estimation area decreases.

Estimation procedures and the calculation of confidence intervals for typical FIA tables are discussed in chapter 4. Additional information on estimation and confidence intervals can be found in Bechtold and Patterson (2005).

Chapter 3 -- Database Structure

This chapter provides information about the database tables, including detailed descriptions of all attributes within the tables. Each column or attribute in a table is listed with its unabbreviated name, followed by a description of the attribute. Attributes that are coded include a list of the codes and their meanings. Appendix A is an index of the attributes, sorted alphabetically by column name, showing the table where the column is found including the attribute number in the table. Some overview information is presented below, followed by a section with complete information about all tables and attributes.

Table Descriptions

There are nineteen data tables and eleven reference tables in the Phase 1 and Phase 2 portions of the FIA Database.

- SURVEY table – Contains one record for each year an inventory is conducted in a State for annual inventory or one record for each periodic inventory.
  - SURVEY.CN = PLOT.SRV_CN links the unique inventory record for a State and year to the plot records.
- COUNTY table – Reference table for the county codes and names. This table also includes survey unit codes.
  - COUNTY.CN = PLOT.CTY_CN links the unique county record to the plot record.
- PLOT table – Provides information relevant to the entire 1-acre field plot. This table links to most other tables, and the linkage is made using PLOT.CN = *TABLE_NAME*.PLT_CN (*TABLE_NAME* is the name of any table containing the column name PLT_CN). Below are some examples of linking PLOT to other tables.
  - PLOT.CN = COND.PLT_CN links the unique plot record to the condition class record(s).
  - PLOT.CN = SUBPLOT.PLT_CN links the unique plot record to the subplot records.
  - PLOT.CN = TREE.PLT_CN links the unique plot record to the tree records.
  - PLOT.CN = SEEDLING.PLT_CN links the unique plot record to the seedling records.
- COND table – Provides information on the discrete combination of landscape attributes that define the condition (a condition will have the same land class, reserved status, owner group, forest type, stand-size class, regeneration status, and stand density).
  - PLOT.CN = COND.PLT_CN links the condition class record (s) to the plot table.
  - COND.PLT_CN = SITETREE.PLT_CN and COND.CONDID = SITETREE.CONDID links the condition class record to the site tree data.
  - COND.PLT_CN = TREE.PLT_CN and COND.CONDID = TREE.CONDID links the condition class record to the tree data.
- SUBPLOT table – Describes the features of a single subplot. There are multiple subplots per 1-acre field plot and there can be multiple conditions sampled on each subplot.
  - PLOT.CN = SUBPLOT.PLT_CN links the unique plot record to the subplot records.
  - SUBPLOT.PLT_CN = COND.PLT_CN and SUBPLOT.MACRCOND = COND.CONDID links the macroplot conditions to the condition class record.
  - SUBPLOT.PLT_CN = COND.PLT_CN and SUBPLOT.SUBPCOND = COND.CONDID links the subplot conditions to the condition class record.
  - SUBPLOT.PLT_CN = COND.PLT_CN and SUBPLOT.MICRCOND = COND.CONDID links the microplot conditions to the condition class record.

- SUBP_COND table – Contains information about the proportion of a subplot in a condition.
  - PLOT.CN = SUBP_COND.PLT_CN links the subplot condition class record to the plot table.
  - SUBP_COND.PLT_CN = COND.PLT_CN and SUBP_COND.CONDID = COND.CONDID links the condition class records found on the four subplots to the subplot description.
- TREE table – Provides information for each tree 1 inch in diameter and larger found on a microplot, subplot, or core optional macroplot.
  - PLOT.CN = TREE.PLT_CN links the tree records to the unique plot record.
  - COND.PLT_CN = TREE.PLT_CN and COND.CONDID = TREE.CONDID links the tree records to the unique condition record.
- SEEDLING table – Provides a count of the number of live trees of a species found on a microplot that are less than 1 inch in diameter but at least 6 inches in length for conifer species or at least 12 inches in length for hardwood species.
  - PLOT.CN = SEEDLING.PLT_CN links the seedling records to the unique plot record.
- SITETREE table – Provides information on the site tree(s) collected in order to calculate site index and/or site productivity information for a condition.
  - PLOT.CN = SITETREE.PLT_CN links the site tree records to the unique plot record.
  - SITETREE.PLT_CN = COND.PLT_CN and SITETREE.CONDID = COND.CONDID links the site tree record(s)to the unique condition class record.
- BOUNDARY table – Provides a description of the demarcation line between two conditions that occur on a single subplot.
  - PLOT.CN = BOUNDARY.PLT_CN links the boundary records to the unique plot record.
- SUBP_COND_CHNG_MTRX table – Contains information about the mix of current and previous conditions that occupy the same area on the subplot.
  - PLOT.CN = SUBP_COND_CHNG_MTRX.PLT_CN links the subplot condition change matrix records to the unique plot record.
  - PLOT.PREV_PLOT_CN = SUBP_COND_CHNG_MTRX.PREV_PLT_CN links the subplot condition change matrix records to the unique previous plot record.
- TREE_REGIONAL_BIOMASS table – Contains biomass estimates computed using equations and methodology that varies by FIA work unit. This table retains valuable information for generating biomass estimates that match earlier published reports.
  - TREE.CN = TREE_REGIONAL_BIOMASS.TRE_CN links a tree regional biomass record to the corresponding unique tree.
- POP_ESTN_UNIT table – An estimation unit is a geographic area that can be drawn on a map. It has known area and the sampling intensity must be the same within a stratum within an estimation unit. Generally estimation units are contiguous areas, but exceptions are made when certain ownerships, usually National Forests, are sampled at different intensities. One record in the POP_ESTN_UNIT table corresponds to a single estimation unit.
  - POP_ESTN_UNIT.CN = POP_STRATUM.ESTN_UNIT_CN links the unique stratified geographical area (ESTN_UNIT) to the strata (STRATUMCD) that are assigned to each ESTN_UNIT.
- POP_EVAL table – An evaluation is the combination of a set of plots (the sample) and a set of Phase 1 data (obtained through remote sensing, called a stratification) that can be used to produce population estimates for a State (an evaluation may be created to produce population estimates for a region other than a State, such as the Black Hills National Forest).

A record in the POP_EVAL table identifies one evaluation and provides some descriptive information about how the evaluation may be used.

- POP_ESTN_UNIT.EVAL_CN = POP_EVAL.CN links the unique evaluation identifier (EVALID) in the POP_EVAL table to the unique geographical areas (ESTN_UNIT) that are stratified. Within a population evaluation (EVALID) there can be multiple population estimation units, or geographic areas across which there are a number of values being estimated (e.g., estimation of volume across counties for a given State.)

- POP_EVAL_ATTRIBUTE table – Provides information as to which population estimates can be provided by an evaluation. If an evaluation can produce 22 of the 92 currently supported population estimates, there will be 22 records in the POP_EVAL_ATTRIBUTE table (one per population estimate) for that evaluation.
  - POP_EVAL.CN = POP_EVAL_ATTRIBUTE.EVAL_CN links the unique evaluation identifier to the list of population estimates that can be derived for that evaluation.
- POP_EVAL_GRP table – Provides information on the suite of evaluations that were used to generate a complete set of reports for an inventory. In a typical State inventory report, one evaluation is used to generate an estimate of the total land area; a second evaluation is used to generate current estimates of volume, numbers of trees and biomass; and a third evaluation is used for estimating growth, removals and mortality. One record in the POP_EVAL_GRP record identifies all the evaluations that were used in generating estimates for a State inventory report. Each record in the POP_EVAL table corresponds to an EVAL_CN_FOR_XX column in the POP_EVAL_GRP table, (XX is one of the following: Expall, Expcurr, Expvol, Expgrow, Expmort, or Expremv). Similar information is contained in the POP_EVAL_TYP table, which has been added to this version of the database.
  - POP_EVAL_TYP.EVAL_GRP_CN = POP_EVAL_GRP.CN links the evaluation type record to the evaluation group record.
  - POP_EVAL.CN = POP_EVAL_GRP.EVAL_CN_FOR_EXPALL links the evaluation for all land to the evaluation identifier that includes all plots used to make the estimate.
  - POP_EVAL.CN = POP_EVAL_GRP.EVAL_CN_FOR_EXPCURR links the evaluation for sampled land to the evaluation identifier that includes all sampled plots used to make the estimate.
  - POP_EVAL.CN = POP_EVAL_GRP.EVAL_CN_FOR_EXPVOL links the evaluation for tree volume, biomass, or number of trees to the evaluation identifier that includes all plots used to make these estimates.
  - POP_EVAL.CN = POP_EVAL_GRP.EVAL_CN_FOR_EXPGROW links the evaluation for average annual tree growth to the evaluation identifier that includes all remeasured plots used to make the estimate.
  - POP_EVAL.CN = POP_EVAL_GRP.EVAL_CN_FOR_EXPMORT links the evaluation for average annual tree mortality to the evaluation identifier that includes all remeasured plots used to make the estimate.
  - POP_EVAL.CN = POP_EVAL_GRP.EVAL_CN_FOR_EXPREMV links the evaluation for average annual tree removals to the evaluation identifier that includes all remeasured plots used to make the estimate.
- POP_EVAL_TYP table – Provides information on the type of evaluations that were used to generate a set of tables for an inventory report. In a typical State inventory report, one evaluation is used to generate an estimate of the total land area; a second evaluation is used to generate current estimates of volume, numbers of trees and biomass; and a third evaluation is used for estimating growth, removals and mortality.

  - POP_EVAL_TYP.EVAL_CN = POP_EVAL.CN links the evaluation type record to the evaluation record.
  - POP_EVAL_TYP.EVAL_GRP_CN = POP_EVAL_GRP.CN links the evaluation type record to the evaluation group record.
  - POP_EVAL_TYP.EVAL_TYP = REF_POP_EVAL_TYP_DESCR.EVAL_TYP links an evaluation type record to an evaluation type description reference record.
- POP_PLOT_STRATUM_ASSGN table – Stratum information is assigned to a plot by overlaying the plot's location on the Phase 1 imagery. Plots are linked to their appropriate stratum for an evaluation via the POP_PLOT_STRATUM_ASSGN table.
  - POP_PLOT_STRATUM_ASSGN.PLT_CN = PLOT.CN links the stratum assigned to the plot record.
- POP_STRATUM table – The area within an estimation unit is divided into strata. The area for each stratum can be calculated by determining the proportion of Phase 1 pixels/plots in each stratum and multiplying that proportion by the total area in the estimation unit. Information for a single stratum is stored in a single record of the POP_STRATUM table.
  - POP_STRATUM.CN = POP_PLOT_STRATUM_ASSGN.STRATUM_CN links the defined stratum to each plot.
- REF_CITATION table – Identifies the published source for information on specific gravities, moisture content, and bark as a percent of wood volume that is provided in the REF_SPECIES table.
  - REF_SPECIES.WOOD_SPGR_GREENVOL_DRYWT_CIT = REF_CITATION.CITATION_NBR
  - REF_SPECIES.BARK_SPGR_GREENVOL_DRYWT_CIT = REF_CITATION.CITATION_NBR
  - REF_SPECIES.MC_PCT_GREEN_WOOD_CIT = REF_CITATION.CITATION_NBR
  - REF_SPECIES.MC_PCT_GREEN_BARK_CIT = REF_CITATION.CITATION_NBR
  - REF_SPECIES.WOOD_SPGR_MC12VOL_DRYWT_CIT = REF_CITATION.CITATION_NBR
  - REF_SPECIES.BARK_VOL_PCT_CIT = REF_CITATION.CITATION_NBR
- REF_FIADB_VERSION table – Contains information identifying the format of the currently available FIADB.
- REF_FOREST_TYPE table – A reference table containing forest type codes, descriptive names, forest type group codes and other information. Data users should link codes as shown below and then obtain the information stored in MEANING to convert the code to a name.
  - REF_FOREST_TYPE.VALUE = COND.FORTYPCD links the forest type reference record to the condition forest code used for reporting and analysis purposes.
  - REF_FOREST_TYPE.VALUE = COND.FLDTYPCD links the forest type reference record to the condition forest type code recorded by field crews.
  - REF_FOREST_TYPE.VALUE = COND.FORTYPCDCALC links the forest type reference record to the condition forest type code calculated by an algorithm.
- REF_POP_ATTRIBUTE table – Identifies all of the population estimates that are currently supported, and provides information useful to the estimation procedure. There are currently 92 records in the REF_POP_ATTRIBUTE table providing information ranging from how to calculate forest area to average annual net growth on forestland.

  - REF_POP_ATTRIBUTE.ATTRIBUTE_NBR = POP_EVAL_ATTRIBUTE.ATTRIBUTE_NBR links the description of the unique population estimate to the records of evaluations that can be used to make those estimates.
- REF_POP_EVAL_TYP_DESCR table – A reference table containing the description for each evaluation type.
  - REF_POP_EVAL_TYP_DESCR.EVAL_TYP = POP_EVAL_TYP.EVAL_TYP links an evaluation type description reference record to an evaluation type record.
- REF_SPECIES table – A reference table containing the species code, descriptive common name, scientific name, and many other attributes for each species. For example, data users who want to convert the species code to the associated common name should link codes as shown below and then obtain the information stored in COMMON_NAME.
  - REF_SPECIES.SPCD = TREE.SPCD links the species reference table record to the tree species code.
  - REF_SPECIES.SPCD = SEEDLING.SPCD links the species reference table record to the seedling species code.
  - REF_SPECIES.SPCD = SITETREE.SPCD links the species reference table record to the site tree species code.
- REF_SPECIES_GROUP table – A reference table containing the species group code, descriptive name, and several other attributes for each species group. Data users should link codes as shown below and then obtain the information stored in NAME to convert the code to a descriptive name.
  - REF_SPECIES_GROUP.SPGRPCD = TREE.SPGRPCD links the species group reference table to the tree species group code.
  - REF_SPECIES_GROUP.SPGRPCD = SEEDLING.SPGRPCD links the species reference table record to the seedling species group code.
  - REF_SPECIES_GROUP.SPGRPCD = SITETREE.SPGRPCD links the species reference table record to the site tree species group code.
- REF_STATE_ELEV – Reference table containing information about minimum and maximum elevation found within a State.
  - REF_STATE_ELEV.STATECD = SURVEY.STATECD links the State elevation reference record to the survey record.
- REF_UNIT table – The description for each survey unit in a State.
  - REF_UNIT.STATECD = PLOT.STATECD and REF_UNIT.VALUE = PLOT.UNITCD links the survey unit description (MEANING) to the PLOT record.

Figure 4 helps to illustrate how the Phase 1 and other population estimation tables relate to one another and to the PLOT table.

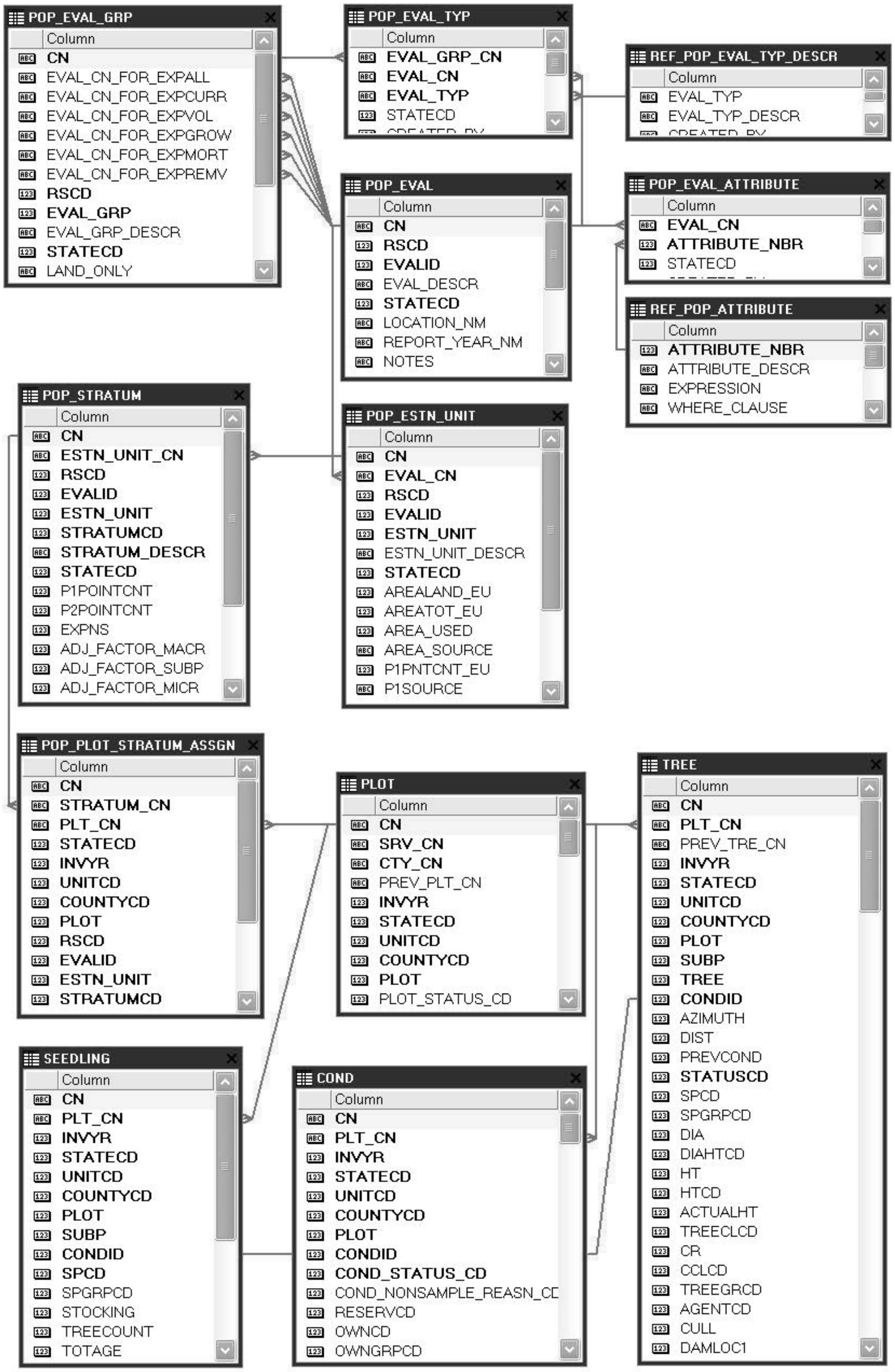

Figure 4. Relationships among Phase 1 and population estimation tables to the Phase 2 plot and other frequently used tables.

Keys Presented with the Tables

Each summarized table in chapter 3 has a list of keys just below the bottom of the table. These keys are used to join data from different tables. The following provides a general definition of each kind of key.

Primary key

A single column in a table whose values uniquely identify each row in an Oracle[1] table. The primary key in each FIADB 4.0 table is the CN column.

The name of the primary key for each table is listed in the table description. It follows the nomenclature of 'TABLEABBREVIATION'_PK. The table abbreviations are:

Table name	Table abbreviation
SURVEY	SRV
COUNTY	CTY
PLOT	PLT
COND	CND
SUBPLOT	SBP
SUBP_COND	SCD
TREE	TRE
SEEDLING	SDL
SITETREE	SIT
BOUNDARY	BND
SUBP_COND_CHNG_MTRX	CMX
TREE_REGIONAL_BIOMASS	TRB
POP_ESTN_UNIT	PEU
POP_EVAL	PEV
POP_EVAL_ATTRIBUTE	PEA
POP_EVAL_GRP	PEG
POP_EVAL_TYP	PET
POP_PLOT_STRATUM_ASSGN	PPSA
POP_STRATUM	PSM
REF_POP_ATTRIBUTE	PAE
REF_POP_EVAL_TYP_DESCR	PED
REF_FOREST_TYPE	RFT
REF_SPECIES	SPC
REF_SPECIES_GROUP	SPG
REF_HABTYP_DESCRIPTION	RHN
REF_HABTYP_PUBLICATION	RPN
REF_CITATION	CIT
REF_FIADB_VERSION	RFN
REF_STATE_ELEV	RSE
REF_UNIT	UNT

[1] The use of trade or firm names in this publication is for reader information only and does not imply endorsement by the U.S. Department of Agriculture of any product or service.

Unique key

Multiple columns in a table whose values uniquely identify each row in an Oracle table. There can be one and only one row for each unique key value.

The unique key varies for each FIADB 4.0 table. The unique key for the PLOT table is STATECD, INVYR, UNITCD, COUNTYCD, and PLOT. The unique key for the COND table is PLT_CN and CONDID.

The name of the unique key for each table is listed in the table description. It follows the nomenclature of 'TABLEABBREVIATION'_UK.

Natural key

A type of unique key made from existing attributes in the table. It is stored as an index in this database.

Not all FIADB 4.0 tables have a natural key. For example, there is no natural key in the PLOT table, rather the natural key and the unique key are the same. The natural key for the COND table is STATECD, INVYR, UNITCD, COUNTYCD, PLOT, and CONDID.

The name of the natural key for each table is listed in the table description. It follows the nomenclature of 'TABLEABBREVIATION'_NAT_I.

Foreign key

A column in a table that is used as a link to a matching column in another Oracle table.

A foreign key connects a record in one table to one and only one record in another table. Foreign keys are used both to link records between data tables and as a check (or constraint) to prevent "unrepresented data." For example, if there are rows of data in the TREE table for a specific plot, there needs to be a corresponding data row for that same plot in the PLOT table. The foreign key in the TREE table is the attribute PLT_CN, which links specific rows in the TREE table to one record in the PLOT table using the plot attribute CN.

The foreign key for the COND table is PLT_CN. There is always a match of the PLT_CN value to the CN value in the PLOT table.

The name of the foreign key for each table is listed in the table description. It follows the nomenclature of
'SOURCETABLEABBREVIATION'_'MATCHINGTABLEABBREVIATION'_FK, where the source table is the table containing the foreign key and the matching table is the table the foreign key matches. The foreign key usually matches the CN column of the matching table. Most tables in FIADB 4.0 have only one foreign key, but tables can have multiple foreign keys.

Survey Table (Oracle table name is SURVEY)

	Column name	Descriptive name	Oracle data type
1	CN	Sequence number	VARCHAR2(34)
2	INVYR	Inventory year	NUMBER(4)
3	P3_OZONE_IND	Phase 3 ozone indicator	VARCHAR2(1)
4	STATECD	State code	NUMBER(4)
5	STATEAB	State abbreviation	VARCHAR2(2)
6	STATENM	State name	VARCHAR2(28)
7	RSCD	Region or station code	NUMBER(2)
8	ANN_INVENTORY	Annual inventory	VARCHAR2(1)
9	NOTES	Notes	VARCHAR2(2000)
10	CREATED_BY	Created by	VARCHAR2(30)
11	CREATED_DATE	Created date	DATE
12	CREATED_IN_INSTANCE	Created in instance	VARCHAR2(6)
13	MODIFIED_BY	Modified by	VARCHAR2(30)
14	MODIFIED_DATE	Modified date	DATE
15	MODIFIED_IN_INSTANCE	Modified in instance	VARCHAR2(6)
16	CYCLE	Inventory cycle number	NUMBER(2)
17	SUBCYCLE	Inventory subcycle number	NUMBER(2)

Type of Key	Column(s) order	Tables to link	Abbreviated notation
Primary	(CN)	N/A	SRV_PK
Unique	(STATECD, INVYR, P3_OZONE_IND, CYCLE)	N/A	SRV_UK

1. CN — Sequence number. A unique sequence number used to identify a survey record.

2. INVYR — Inventory year. The year that best represents when the inventory data were collected. Under the annual inventory system, a group of plots is selected each year for sampling. The selection is based on a panel system. INVYR is the year in which the majority of plots in that group were collected (plots in the group have the same panel and, if applicable, subpanel). Under periodic inventory, a reporting inventory year was selected, usually based on the year in which the majority of the plots were collected or the mid-point of the years over which the inventory spanned. For either annual or periodic inventory, INVYR is not necessarily the same as MEASYEAR.

   Exceptions:
   INVYR = 9999. INVYR is set to 9999 to distinguish Phase 3 plots taken by the western FIA work units that are “off subpanel.” This is due to differences in measurement intervals between Phase 3 (measurement interval = 5 years) and Phase 2 (measurement interval = 10 years) plots. Only users interested in performing certain Phase 3 data analyses should access plots with this anomalous value in INVYR.

INVYR <100. INVYR <100 indicates that population estimates were derived from a pre-NIMS regional processing system and the same plot either has been or may soon be re-processed in NIMS as part of a separate evaluation. The NIMS processed copy of the plot follows the standard INVYR format. This only applies to plots collected in the South (SURVEY.RSCD = 33) with the national design or a similar regional design (PLOT.DESIGNCD = 1 or 220-233) that were collected when the inventory year was 1998 through 2005.

INVYR = 98 is equivalent to 1998 but processed through regional system
INVYR = 99 is equivalent to 1999 but processed through regional system
INVYR = 0 is equivalent to 2000 but processed through regional system
INVYR = 1 is equivalent to 2001 but processed through regional system
INVYR = 2 is equivalent to 2002 but processed through regional system
INVYR = 3 is equivalent to 2003 but processed through regional system
INVYR = 4 is equivalent to 2004 but processed through regional system
INVYR = 5 is equivalent to 2005 but processed through regional system

3. P3_OZONE_IND

Phase 3 ozone indicator. Values are Y (yes) and N (no). If Y, then the Survey is for a P3 ozone inventory. If N, then the Survey is not for a P3 ozone inventory. Note that P3_OZONE_IND is part of the unique key because ozone data are stored as a separate inventory (survey); therefore, combinations of STATECD and INVYR may occur more than one time.

4. STATECD — State code. Bureau of the Census Federal Information Processing Standards (FIPS) two-digit code for each State. Refer to appendix C.

5. STATEAB — State abbreviation. The two-character State abbreviation. Refer to appendix C.

6. STATENM — State name. Refer to appendix C.

7. RSCD — Region or Station Code. Identification number of the Forest Service National Forest System Region or Station (FIA work unit) that provided the inventory data (see appendix C for more information).

Code	Description
22	Rocky Mountain Research Station (RMRS)
23	North Central Research Station (NCRS)
24	Northeastern Research Station (NERS)
26	Pacific Northwest Research Station (PNWRS)
27	Pacific Northwest Research Station (PNWRS)-Alaska
33	Southern Research Station (SRS)

8. ANN_INVENTORY

Annual Inventory. An indicator to show if a particular inventory was collected as an annual inventory or a periodic inventory. Values are Y or N, and Y means that the inventory is annual.

9. NOTES Notes. An optional item where notes about the inventory may be stored.

10. CREATED_BY Created by. The employee who created the record. This attribute is intentionally left blank in download files.

11. CREATED_DATE

Created date. The date the record was created. Date will be in the form DD-MON-YYYY.

12. CREATED_IN_INSTANCE

Created in instance. The database instance in which the record was created. Each computer system has a unique database instance code and this attribute stores that information to determine on which computer the record was created.

13. MODIFIED_BY

Modified by. The employee who modified the record. This field will be blank (null) if the data have not been modified since initial creation. This attribute is intentionally left blank in download files.

14. MODIFIED_DATE

Modified date. The date the record was last modified. This field will be blank (null) if the data have not been modified since initial creation. Date will be in the form DD-MON-YYYY.

15. MODIFIED_IN_INSTANCE

Modified in instance. The database instance in which the record was modified. This field will be blank (null) if the data have not been modified since initial creation.

16. CYCLE Inventory cycle number. A number assigned to a set of plots, measured over a particular period of time from which a State estimate using all possible plots is obtained. A cycle number >1 does not necessarily mean that information for previous cycles resides in the database. A cycle is relevant for periodic and annual inventories.

17. SUBCYCLE Inventory subcycle number. For an annual inventory that takes n years to measure all plots, subcycle shows in which of the n years of the cycle the

data were measured. Subcycle is 0 for a periodic inventory. Subcycle 99 may be used for plots that are not included in the estimation process.

County Table (Oracle table name is COUNTY)

	Column name	Descriptive name	Oracle data type
1	STATECD	State code	NUMBER(4)
2	UNITCD	Survey unit code	NUMBER(2)
3	COUNTYCD	County code	NUMBER(3)
4	COUNTYNM	County name	VARCHAR2(50)
5	CN	Sequence number	VARCHAR2(34)
6	CREATED_BY	Created by	VARCHAR2(30)
7	CREATED_DATE	Created date	DATE
8	CREATED_IN_INSTANCE	Created in instance	VARCHAR2(6)
9	MODIFIED_BY	Modified by	VARCHAR2(30)
10	MODIFIED_DATE	Modified date	DATE
11	MODIFIED_IN_INSTANCE	Modified in instance	VARCHAR2(6)

Type of key	Column(s) order	Tables to link	Abbreviated notation
Primary	(CN)	N/A	CTY_PK
Unique	(STATECD, UNITCD, COUNTYCD)	N/A	CTY_UK

1. STATECD State code. Bureau of the Census Federal Information Processing Standards (FIPS) two-digit code for each State. Refer to appendix C.

2. UNITCD Survey unit code. Forest Inventory and Analysis survey unit identification number. Survey units are usually groups of counties within each State. For periodic inventories, Survey units may be made up of lands of particular owners. Refer to appendix C for codes.

3. COUNTYCD County code. The identification number for a county, parish, watershed, borough, or similar governmental unit in a State. FIPS codes from the Bureau of the Census are used. Refer to appendix C for codes.

4. COUNTYNM County name. County name as recorded by the Bureau of the Census for individual counties, or the name given to a similar governmental unit by the FIA program. Only the first 50 characters of the name are used. Refer to appendix C for names.

5. CN Sequence number. A unique sequence number used to identify a county record.

6. CREATED_BY Created by. The employee who created the record. This attribute is intentionally left blank in download files.

7. CREATED_DATE

   Created date. The date the record was created. Date will be in the form DD-MON-YYYY.

8. CREATED_IN_INSTANCE

Created in instance. The database instance in which the record was created. Each computer system has a unique database instance code and this attribute stores that information to determine on which computer the record was created.

9. MODIFIED_BY

Modified by. The employee who modified the record. This field will be blank (null) if the data have not been modified since initial creation. This attribute is intentionally left blank in download files.

10. MODIFIED_DATE

Modified date. The date the record was last modified. This field will be blank (null) if the data have not been modified since initial creation. Date will be in the form DD-MON-YYYY.

11. MODIFIED_IN_INSTANCE

Modified in instance. The database instance in which the record was modified. This field will be blank (null) if the data have not been modified since initial creation.

Plot Table (Oracle table name is PLOT)

	Column name	Descriptive name	Oracle data type
1	CN	Sequence number	VARCHAR2(34)
2	SRV_CN	Survey sequence number	VARCHAR2(34)
3	CTY_CN	County sequence number	VARCHAR2(34)
4	PREV_PLT_CN	Previous plot sequence number	VARCHAR2(34)
5	INVYR	Inventory year	NUMBER(4)
6	STATECD	State code	NUMBER(4)
7	UNITCD	Survey unit code	NUMBER(2)
8	COUNTYCD	County code	NUMBER(3)
9	PLOT	Phase 2 plot number	NUMBER(5)
10	PLOT_STATUS_CD	Plot status code	NUMBER(1)
11	PLOT_NONSAMPLE_REASN_CD	Plot nonsampled reason code	NUMBER(2)
12	MEASYEAR	Measurement year	NUMBER(4)
13	MEASMON	Measurement month	NUMBER(2)
14	MEASDAY	Measurement day	NUMBER(2)
15	REMPER	Remeasurement period	NUMBER(3,1)
16	KINDCD	Sample kind code	NUMBER(2)
17	DESIGNCD	Plot design code	NUMBER(4)
18	RDDISTCD	Horizontal distance to improved road code	NUMBER(2)
19	WATERCD	Water on plot code	NUMBER(2)
20	LAT	Latitude	NUMBER(8,6)
21	LON	Longitude	NUMBER(9,6)
22	ELEV	Elevation	NUMBER(5)
23	GROW_TYP_CD	Type of annual volume growth code	NUMBER(2)
24	MORT_TYP_CD	Type of annual mortality volume code	NUMBER(2)
25	P2PANEL	Phase 2 panel number	NUMBER(2)
26	P3PANEL	Phase 3 panel number	NUMBER(2)
27	ECOSUBCD	Ecological subsection code	VARCHAR2(7)
28	CONGCD	Congressional district code	NUMBER(4)
29	MANUAL	Manual (field guide) version number	NUMBER(3,1)
30	SUBPANEL	Subpanel	NUMBER(2)
31	KINDCD_NC	Sample kind code, North Central	NUMBER(2)
32	QA_STATUS	Quality assurance status	NUMBER(1)
33	CREATED_BY	Created by	VARCHAR2(30)
34	CREATED_DATE	Created date	DATE
35	CREATED_IN_INSTANCE	Created in instance	VARCHAR2(6)
36	MODIFIED_BY	Modified by	VARCHAR2(30)

	Column name	Descriptive name	Oracle data type
37	MODIFIED_DATE	Modified date	DATE
38	MODIFIED_IN_INSTANCE	Modified in instance	VARCHAR2(6)
39	MICROPLOT_LOC	Microplot location	VARCHAR2(12)
40	DECLINATION	Declination	NUMBER(4,1)
41	EMAP_HEX	EMAP hexagon	NUMBER(7)
42	SAMP_METHOD_CD	Sample method code	NUMBER(1)
43	SUBP_EXAMINE_CD	Subplots examined code	NUMBER(1)
44	MACRO_BREAKPOINT_DIA	Macroplot breakpoint diameter	NUMBER(2)
45	INTENSITY	Intensity	VARCHAR2(2)
46	CYCLE	Inventory cycle number	NUMBER(2)
47	SUBCYCLE	Inventory subcycle number	NUMBER(2)
48	ECO_UNIT_PNW	Ecological unit, Pacific Northwest Research Station	VARCHAR2(10)
49	TOPO_POSITION_PNW	Topographic position, Pacific Northwest Research Station	VARCHAR2(2)
50	NF_SAMPLING_STATUS_CD	Nonforest sampling status code	NUMBER(1)
51	NF_PLOT_STATUS_CD	Nonforest plot status cd	NUMBER(1)
52	NF_PLOT_NONSAMPLE_REASN_CD	Nonforest plot nonsampled reason code	NUMBER(2)
53	P2VEG_SAMPLING_STATUS_CD	P2 vegetation sampling status code	NUMBER(1)
54	P2VEG_SAMPLING_LEVEL_DETAIL_CD	P2 vegetation sampling level detail code	NUMBER(1)
55	INVASIVE_SAMPLING_STATUS_CD	Invasive sampling status code	NUMBER(1)
56	INVASIVE_SPECIMEN_RULE_CD	Invasive specimen rule code	NUMBER(1)

Type of Key	Column(s) order	Tables to link	Abbreviated notation
Primary	(CN)	N/A	PLT_PK
Unique	(STATECD, INVYR, UNITCD, COUNTYCD, PLOT)	N/A	PLT_UK
Foreign	(CTY_CN)	PLOT to COUNTY	PLT_CTY_FK
	(SRV_CN)	PLOT to SURVEY	PLT_SRV_FK

1. CN Sequence number. A unique sequence number used to identify a plot record.

2. SRV_CN Survey sequence number. Foreign key linking the plot record to the survey record.

3. CTY_CN County sequence number. Foreign key linking the plot record to the county record.

4. PREV_PLT_CN

Previous plot sequence number. Foreign key linking the plot record to the previous inventory's plot record for this location. Only populated on remeasurement plots.

5. INVYR

Inventory year. The year that best represents when the inventory data were collected. Under the annual inventory system, a group of plots is selected each year for sampling. The selection is based on a panel system. INVYR is the year in which the majority of plots in that group were collected (plots in the group have the same panel and, if applicable, subpanel). Under periodic inventory, a reporting inventory year was selected, usually based on the year in which the majority of the plots were collected or the mid-point of the years over which the inventory spanned. For either annual or periodic inventory, INVYR is not necessarily the same as MEASYEAR.

Exceptions:
INVYR = 9999. INVYR is set to 9999 to distinguish Phase 3 plots taken by the western FIA work units that are "off subpanel." This is due to differences in measurement intervals between Phase 3 (measurement interval = 5 years) and Phase 2 (measurement interval = 10 years) plots. Only users interested in performing certain Phase 3 data analyses should access plots with this anomalous value in INVYR.

INVYR <100. INVYR <100 indicates that population estimates were derived from a pre-NIMS regional processing system and the same plot either has been or may soon be re-processed in NIMS as part of a separate evaluation. The NIMS processed copy of the plot follows the standard INVYR format. This only applies to plots collected in the South (SURVEY.RSCD = 33) with the national design or a similar regional design (DESIGNCD = 1 or 220-233) that were collected when the inventory year was 1998 through 2005.

INVYR = 98 is equivalent to 1998 but processed through regional system
INVYR = 99 is equivalent to 1999 but processed through regional system
INVYR = 0 is equivalent to 2000 but processed through regional system
INVYR = 1 is equivalent to 2001 but processed through regional system
INVYR = 2 is equivalent to 2002 but processed through regional system
INVYR = 3 is equivalent to 2003 but processed through regional system
INVYR = 4 is equivalent to 2004 but processed through regional system
INVYR = 5 is equivalent to 2005 but processed through regional system

6. STATECD

State code. Bureau of the Census Federal Information Processing Standards (FIPS) two-digit code for each State. Refer to appendix C.

7. UNITCD

Survey unit code. Forest Inventory and Analysis survey unit identification number. Survey units are usually groups of counties within each State. For periodic inventories, Survey units may be made up of lands of particular owners. Refer to appendix C for codes.

8. COUNTYCD County code. The identification number for a county, parish, watershed, borough, or similar governmental unit in a State. FIPS codes from the Bureau of the Census are used. Refer to appendix C for codes.

9. PLOT Phase 2 plot number. An identifier for a plot. Along with STATECD, INVYR, UNITCD, COUNTYCD and/or some other combinations of variables, PLOT may be used to uniquely identify a plot.

10. PLOT_STATUS_CD

Plot status code. A code that describes the sampling status of the plot. Blank (null) values may be present for periodic inventories.

Code	Description
1	Sampled – at least one accessible forest land condition present on plot
2	Sampled – no accessible forest land condition present on plot
3	Nonsampled

11. PLOT_NONSAMPLE_REASN_CD

Plot nonsampled reason code. For entire plots that cannot be sampled, one of the following reasons is recorded.

Code	Description
01	Outside U.S. boundary – Entire plot is outside of the U.S. border.
02	Denied access area – Access to the entire plot is denied by the legal owner, or by the owner of the only reasonable route to the plot.
03	Hazardous – Entire plot cannot be accessed because of a hazard or danger, for example cliffs, quarries, strip mines, illegal substance plantations, high water, etc.
05	Lost data – Plot data file was discovered to be corrupt after a panel was completed and submitted for processing.
06	Lost plot – Entire plot cannot be found.
07	Wrong location – Previous plot can be found, but its placement is beyond the tolerance limits for plot location.
08	Skipped visit – Entire plot skipped. Used for plots that are not completed prior to the time a panel is finished and submitted for processing. This code is for office use only.
09	Dropped intensified plot – Intensified plot dropped due to a change in grid density. This code used only by units engaged in intensification. This code is for office use only.
10	Other – Entire plot not sampled due to a reason other than one of the specific reasons already listed.
11	Ocean – Plot falls in ocean water below mean high tide line.

12. MEASYEAR Measurement year. The year in which the plot was completed. MEASYEAR may differ from INVYR.

13. MEASMON Measurement month. The month in which the plot was completed. May be blank (null) for periodic inventory.

Code	Description	Code	Description
01	January	07	July
02	February	08	August
03	March	09	September
04	April	10	October
05	May	11	November
06	June	12	December

14. MEASDAY Measurement day. The day of the month in which the plot was completed. May be blank (null) for periodic inventory.

15. REMPER Remeasurement period. The number of years between measurements for remeasured plots. This attribute is null (blank) for new plots or remeasured plots that are not used for growth, removals, or mortality estimates. For data processed with NIMS, REMPER is the number of years between measurements (to the nearest 0.1 year). For data processed with systems other than NIMS, remeasurement period is based on the number of growing seasons between measurements. Allocation of parts of the growing season by month is different for each FIA work unit. Contact the appropriate FIA work unit for information on how this is done for a particular State. NOTE: it is **not** valid to use REMPER to estimate periodic change.

16. KINDCD Sample kind code. A code indicating the type of plot installation. Database users may also want to examine DESIGNCD to obtain additional information about the kind of plot being selected.

Code	Description
0	Periodic inventory plot
1	Initial installation of a National design plot
2	Remeasurement of previously installed National design plot
3	Replacement of previously installed National design plot
4	Modeled periodic inventory plot (Northeastern and North Central only)

17. DESIGNCD Plot design code. A code indicating the type of plot design used to collect the data. Refer to appendix B for a list of codes and descriptions.

18. RDDISTCD Horizontal distance to improved road code. The straight-line distance from plot center to the nearest improved road, which is a road of any width that is maintained as evidenced by pavement, gravel, grading, ditching, and/or other improvements. Populated for all forested plots using the National Field Guide protocols (MANUAL $\geq$1.0) and populated by some FIA work units for inventory plots collected where MANUAL $<$1.0.

Code	Description
1	100 ft or less
2	101 ft to 300 ft
3	301 ft to 500 ft
4	501 ft to 1000 ft
5	1001 ft to 1/2 mile
6	1/2 to 1 mile
7	1 to 3 miles
8	3 to 5 miles
9	Greater than 5 miles

19. WATERCD — Water on plot code. Water body <1 acre in size or a stream <30 feet wide that has the greatest impact on the area within the forest land portion of the four subplots. The coding hierarchy is listed in order from large permanent water to temporary water. Populated for all forested plots using the National Field Guide protocols (MANUAL ≥1.0) and populated by some FIA work units for inventory plots collected where MANUAL <1.0.

Code	Description
0	None – no water sources within the accessible forest land condition class
1	Permanent streams or ponds too small to qualify as noncensus water
2	Permanent water in the form of deep swamps, bogs, marshes without standing trees present and less than 1.0 acre in size, or with standing trees
3	Ditch/canal – human-made channels used as a means of moving water, e.g., for irrigation or drainage, which are too small to qualify as noncensus water
4	Temporary streams
5	Flood zones – evidence of flooding when bodies of water exceed their natural banks
9	Other temporary water – specified in plot-level notes.

20. LAT — Latitude. The approximate latitude of the plot in decimal degrees using NAD 83 datum. Actual plot coordinates cannot be released because of a Privacy provision enacted by Congress in the Food Security Act of 1985. Therefore, this attribute is approximately +/- 1 mile and, for annual inventory data, most plots are within +/- ½ mile. Annual data have additional uncertainty for private plots caused by swapping plot coordinates for up to 20 percent of the plots. In some cases, the county centroid is used when the actual coordinate is not available.

21. LON — Longitude. The approximate longitude of the plot in decimal degrees using NAD 83 datum. Actual plot coordinates cannot be released because of a Privacy provision enacted by Congress in the Food Security Act of 1985. Therefore, this attribute is approximately +/- 1 mile and, for annual inventory data, most plots are within +/- ½ mile. Annual data have additional uncertainty for private plots caused by swapping plot coordinates for up to 20 percent of the plots. In some cases, the county centroid is used when the actual coordinate is not available.

22. ELEV — Elevation. The distance the plot is located above sea level, recorded in feet (NAD 83 datum). Negative values indicate distance below sea level.

23. GROW_TYP_CD

Type of annual volume growth code. A code indicating how volume growth is estimated. Current annual growth is an estimate of the amount of volume that was added to a tree in the year before the tree was sampled, and is based on the measured diameter increment recorded when the tree was sampled or on a modeled diameter for the previous year. Periodic annual growth is an estimate of the average annual change in volume occurring between two measurements, usually the current inventory and the previous inventory, where the same plot is evaluated twice. Periodic annual growth is the increase in volume between inventories divided by the number of years between each inventory. This attribute is blank (null) if the plot does not contribute to the growth estimate.

Code	Description
1	Current annual
2	Periodic annual

24. MORT_TYP_CD

Type of annual mortality volume code. A code indicating how mortality volume is estimated. Current annual mortality is an estimate of the volume of trees dying in the year before the plot was measured, and is based on the year of death or on a modeled estimate. Periodic annual mortality is an estimate of the average annual volume of trees dying between two measurements, usually the current inventory and previous inventory, where the same plot is evaluated twice. Periodic annual mortality is the loss of volume between inventories divided by the number of years between each inventory. Periodic average annual mortality is the most common type of annual mortality estimated. This attribute is blank (null) if the plot does not contribute to the mortality estimate.

Code	Description
1	Current annual
2	Periodic annual

25. P2PANEL — Phase 2 panel number. The value for P2PANEL ranges from 1 to 7 for annual inventories and is blank (null) for periodic inventories. A panel is a sample in which the same elements are measured on two or more occasions. FIA divides the plots in each State into 5 or 7 panels that can be used to independently sample the population.

26. P3PANEL — Phase 3 panel number. A panel is a sample in which the same elements are measured on two or more occasions. FIA divides the plots in each State into 5 or 7 panels that can be used to independently sample the population. The value for P3PANEL ranges from 1 to 7 for those plots where Phase 3 data were collected. If the plot is not a Phase 3 plot, then this attribute is left blank (null).

27. ECOSUBCD Ecological subsection code. An area of similar surficial geology, lithology, geomorphic process, soil groups, subregional climate, and potential natural communities. Subsection boundaries usually correspond with discrete changes in geomorphology. Subsection information is used for broad planning and assessment. Subsection codes for the coterminous United States were developed as part of the “Forest Service Map of Provinces, Sections, and Subsections of the United States (Cleland and others 2007) (visit http://fsgeodata.fs.fed.us/other_resources/ecosubregions.html). For southeast and south coastal Alaska, the subsection codes are based on the ecological sections as designated in the “Ecoregions and Subregions of Alaska, EcoMap version 2.0” (Nowacki and Brock 1995) (visit http://agdcftp1.wr.usgs.gov/pub/projects/fhm/ecomap.gif). The ECOSUBCD is based on fuzzed and swapped plot coordinates. This attribute is coded for the coterminous United States, southeast and south coastal Alaska, and is left blank (null) in all other instances.

28. CONGCD Congressional district code. A territorial division of a State from which a member of the U.S. House of Representatives is elected. The congressional district code assigned to a plot (regardless of when it was measured) is for the current Congress; the assignment is made based on the plot’s approximate coordinates. CONGCD is a four-digit number. The first two digits are the State FIPS code and the last two digits are the congressional district number. If a State has only one congressional district, the congressional district number is 00. If a plot’s congressional district assignment falls in a State other than the plot’s actual State due to using the approximate coordinates, the congressional district code will be for the nearest congressional district in the correct State. This attribute is coded for the coterminous States and Alaska, and is left blank (null) in all other instances. For more information about the coverage used to assign this attribute, see National Atlas of the United States (2007).

29. MANUAL Manual (field guide) version number. Version number of the Field Guide used to describe procedures for collecting data on the plot. The National FIA Field Guide began with version 1.0; therefore data taken using the National Field procedures will have PLOT.MANUAL ≥1.0. Data taken according to field instructions prior to the use of the National Field Guide have PLOT.MANUAL <1.0.

30. SUBPANEL Subpanel. Subpanel assignment for the plot for those FIA work units using subpaneling. FIA uses a 5-panel system (see P2PANEL) to divide plot sampling over a 5-year period. Funding for western FIA work units is only sufficient to allow plot sampling over a 10-year period. Therefore, panels are further divided into subpanels. This attribute is left blank (null) if subpaneling is not used. In some States, seven panels are used and SUBPANEL is blank (null).

31. KINDCD_NC Sample kind code, North Central. This attribute is populated through 2005 for the former North Central work unit (SURVEY.RSCD = 23) and is blank (null) for all other FIA work units.

Code	Description
0	New/lost
6	Remeasured
8	Old location but not remeasured
20	Skipped
33	Replacement of lost plot

32. QA_STATUS Quality assurance status. A code indicating the type of plot data collected. Populated for all forested subplots using the National Field Guide protocols (MANUAL ≥1.0).

Code	Description
1	Standard production plot
2	Cold check
3	Reference plot (off grid)
4	Training/practice plot (off grid)
5	Botched plot file (disregard during data processing)
6	Blind check
7	Production plot (hot check)

33. CREATED_BY Created by. The employee who created the record. This attribute is intentionally left blank in download files.

34. CREATED_DATE

Created date. The date the record was created. Date will be in the form DD-MON-YYYY.

35. CREATED_IN_INSTANCE

Created in instance. The database instance in which the record was created. Each computer system has a unique database instance code and this attribute stores that information to determine on which computer the record was created.

36. MODIFIED_BY

Modified by. The employee who modified the record. This field will be blank (null) if the data have not been modified since initial creation. This attribute is intentionally left blank in download files.

37. MODIFIED_DATE

Modified date. The date the record was last modified. This field will be blank (null) if the data have not been modified since initial creation. Date will be in the form DD-MON-YYYY.

38. MODIFIED_IN_INSTANCE

Modified in instance. The database instance in which the record was modified. This field will be blank (null) if the data have not been modified since initial creation.

39. MICROPLOT_LOC

Microplot location. Values are 'OFFSET' or 'CENTER.' The offset microplot center is located 12 feet due east (90 degrees) of subplot center. The current standard is that the microplot is located in the 'OFFSET' location, but some earlier inventories, including some early panels of the annual inventory, may contain data where the microplot was located at the 'CENTER' location. Populated for annual inventory and may be populated for periodic inventory.

40. DECLINATION

Declination. (*Core optional.*) The azimuth correction used to adjust magnetic north to true north. All azimuths are assumed to be magnetic azimuths unless otherwise designated. The Portland FIA work unit historically has corrected all compass readings for true north. This field is to be used only in cases where FIA work units are adjusting azimuths to correspond to true north; for FIA work units using magnetic azimuths, this field will always be set = 0 in the office. This field carries a decimal place because the USGS corrections are provided to the nearest half degree. DECLINATION is defined as:

DECLINATION = (TRUE NORTH - MAGNETIC NORTH)

41. EMAP_HEX

EMAP hexagon. The identifier for the approximately 160,000 acre Environmental Monitoring and Assessment Program (EMAP) hexagon in which the plot is located. EMAP hexagons are available to the public, cover the coterminous United States, and have been used in summarizing and aggregating data about numerous natural resources. Populated for annual inventory and may be populated for periodic inventory.

42. SAMP_METHOD_CD

Sample method code. A code indicating if the plot was observed in the field or remotely sensed in the office.

Code	Description
1	Field visited, meaning a field crew physically examined the plot and recorded information at least about subplot 1 center condition (see SUBP_EXAMINE_CD below).
2	Remotely sensed, meaning a determination was made using some type of imagery that a field visit was not necessary. When the plot is sampled remotely, the number of subplots examined (SUBP_EXAMINE_CD) usually equals 1.

43. SUBP_EXAMINE_CD

Subplots examined code. A code indicating the number of subplots examined. By default, PLOT_STATUS_CD = 1 plots have all 4 subplots examined.

Code	Description
1	Only subplot 1 center condition examined and all other subplots assumed (inferred) to be the same
4	All four subplots fully described (no assumptions/inferences)

44. MACRO_BREAKPOINT_DIA

Macroplot breakpoint diameter. (*Core optional.*) A macroplot breakpoint diameter is the diameter (either DBH or DRC) above which trees are measured on the plot extending from 0.01 to 58.9 feet horizontal distance from the center of each subplot. Examples of different breakpoint diameters used by western FIA work units are 24 inches or 30 inches (Pacific Northwest), or 21 inches (Interior West). Installation of macroplots is core optional and is used to have a larger plot size in order to more adequately sample large trees. If macroplots are not being installed, this item will be left blank (null).

45. INTENSITY Intensity. A code used to identify federal base grid annual inventory plots and plots that have been added to intensify a particular sample. Under the federal base grid, one plot is collected in each theoretical hexagonal polygon, which is slightly more than 5,900 acres in size. Plots with INTENSITY = 1 are part of the federal base grid. In some instances, States and/or agencies have provided additional support to increase the sampling intensity for an area. Supplemental plots have INTENSITY set to higher numbers depending on the amount of plot intensification chosen for the particular estimation unit. Populated for annual inventory data only.

46. CYCLE Inventory cycle number. A number assigned to a set of plots, measured over a particular period of time from which a State estimate using all possible plots is obtained. A cycle number >1 does not necessarily mean that information for previous cycles resides in the database. A cycle is relevant for periodic and annual inventories.

47. SUBCYCLE Inventory subcycle number. For an annual inventory that takes n years to measure all plots, subcycle shows in which of the n years of the cycle the data were measured. Subcycle is 0 for a periodic inventory. Subcycle 99 may be used for plots that are not included in the estimation process.

48. ECO_UNIT_PNW

Ecological unit, Pacific Northwest Research Station. Plots taken by PNW FIA are assigned to the ecological unit in which they are located. Certain units have stocking adjustments made to the plots that occur on very low productivity lands, which thereby reduces the estimated potential

productivity of the plot. More information can be found in MacLean (1973). Only collected by certain FIA work units (SURVEY.RSCD = 26 or 27).

49. TOPO_POSITION_PNW

Topographic position, Pacific Northwest Research Station. The topographic position that describes the plot area. Illustrations available in Plot section of PNW field guide located at: http//www.fs.fed.us/pnw/fia/publications/fieldmanuals.shtml. Adapted from information found in Wilson (1900). Only collected by certain FIA work units (SURVEY.RSCD = 26).

Code	Topographic position	Common shape of slope
1	Ridge top or mountain peak over 130 feet	Flat
2	Narrow ridge top or mountain peak over 130 feet wide	Convex
3	Side hill – upper 1/3	Convex
4	Side hill – middle 1/3	No rounding
5	Side hill – lower 1/3	Concave
6	Canyon bottom less than 660 feet wide	Concave
7	Bench, terrace or dry flat	Flat
8	Broad alluvial flat over 660 feet wide	Flat
9	Swamp or wet flat	Flat

50. NF_SAMPLING_STATUS_CD

Nonforest sampling status code. Intentionally left blank. Will be populated in version 5.0.

51. NF_PLOT_STATUS_CD

Nonforest plot status code. Intentionally left blank. Will be populated in version 5.0.

52. NF_PLOT_NONSAMPLE_REASN_CD

Nonforest plot nonsampled reason code. Intentionally left blank. Will be populated in version 5.0.

53. P2VEG_SAMPLING_STATUS_CD

P2 vegetation sampling status code. Intentionally left blank. Will be populated in version 5.0.

54. P2VEG_SAMPLING_LEVEL_DETAIL_CD

P2 vegetation sampling level detail code. Intentionally left blank. Will be populated in version 5.0.

55. INVASIVE_SAMPLING_STATUS_CD

Invasive sampling status code. Intentionally left blank. Will be populated in version 5.0.

56. INVASIVE_SPECIMEN_RULE_CD

Invasive specimen rule code. Intentionally left blank. Will be populated in version 5.0.

Condition Table (Oracle table name is COND)

	Column name	Descriptive name	Oracle data type
1	CN	Sequence number	VARCHAR2(34)
2	PLT_CN	Plot sequence number	VARCHAR2(34)
3	INVYR	Inventory year	NUMBER(4)
4	STATECD	State code	NUMBER(4)
5	UNITCD	Survey unit code	NUMBER(2)
6	COUNTYCD	County code	NUMBER(3)
7	PLOT	Phase 2 plot number	NUMBER(5)
8	CONDID	Condition class number	NUMBER(1)
9	COND_STATUS_CD	Condition status code	NUMBER(1)
10	COND_NONSAMPLE_ REASN_CD	Condition nonsampled reason code	NUMBER(2)
11	RESERVCD	Reserved status code	NUMBER(2)
12	OWNCD	Owner class code	NUMBER(2)
13	OWNGRPCD	Owner group code	NUMBER(2)
14	FORINDCD	Private owner industrial status code	NUMBER(2)
15	ADFORCD	Administrative forest code	NUMBER(4)
16	FORTYPCD	Forest type code, derived by algorithm	NUMBER(3)
17	FLDTYPCD	Field forest type code	NUMBER(3)
18	MAPDEN	Mapping density	NUMBER(1)
19	STDAGE	Stand age	NUMBER(4)
20	STDSZCD	Stand-size class code derived by algorithm	NUMBER(2)
21	FLDSZCD	Field stand-size class code	NUMBER(2)
22	SITECLCD	Site productivity class code	NUMBER(2)
23	SICOND	Site index for the condition	NUMBER(3)
24	SIBASE	Site index base age	NUMBER(3)
25	SISP	Site index species code	NUMBER(4)
26	STDORGCD	Stand origin code	NUMBER(2)
27	STDORGSP	Stand origin species code	NUMBER
28	PROP_BASIS	Proportion basis	VARCHAR2(12)
29	CONDPROP_UNADJ	Condition proportion unadjusted	NUMBER(5,4)
30	MICRPROP_UNADJ	Microplot proportion unadjusted	NUMBER(5,4)
31	SUBPPROP_UNADJ	Subplot proportion unadjusted	NUMBER(5,4)
32	MACRPROP_UNADJ	Macroplot proportion unadjusted	NUMBER(5,4)
33	SLOPE	Slope	NUMBER(3)
34	ASPECT	Aspect	NUMBER(3)
35	PHYSCLCD	Physiographic class code	NUMBER(2)
36	GSSTKCD	Growing-stock stocking code	NUMBER(2)
37	ALSTKCD	All live stocking code	NUMBER(2)
38	DSTRBCD1	Disturbance 1 code	NUMBER(2)

	Column name	Descriptive name	Oracle data type
39	DSTRBYR1	Disturbance year 1	NUMBER(4)
40	DSTRBCD2	Disturbance 2 code	NUMBER(2)
41	DSTRBYR2	Disturbance year 2	NUMBER(4)
42	DSTRBCD3	Disturbance 3 code	NUMBER(2)
43	DSTRBYR3	Disturbance year 3	NUMBER(4)
44	TRTCD1	Stand treatment 1 code	NUMBER(2)
45	TRTYR1	Treatment year 1	NUMBER(4)
46	TRTCD2	Stand treatment 2 code	NUMBER(2)
47	TRTYR2	Treatment year 2	NUMBER(4)
48	TRTCD3	Stand treatment 3 code	NUMBER(2)
49	TRTYR3	Treatment year 3	NUMBER(4)
50	PRESNFCD	Present nonforest code	NUMBER(2)
51	BALIVE	Basal area of live trees	NUMBER(9,4)
52	FLDAGE	Field-recorded stand age	NUMBER(4)
53	ALSTK	All-live-tree stocking percent	NUMBER(7,4)
54	GSSTK	Growing-stock stocking percent	NUMBER(7,4)
55	FORTYPCDCALC	Forest type code calculated	NUMBER(3)
56	HABTYPCD1	Habitat type code 1	VARCHAR2(10)
57	HABTYPCD1_PUB_CD	Habitat type code 1 publication code	VARCHAR2(10)
58	HABTYPCD1_DESCR_ PUB_CD	Habitat type code 1 description publication code	VARCHAR2(10)
59	HABTYPCD2	Habitat type code 2	VARCHAR2(10)
60	HABTYPCD2_PUB_CD	Habitat type code 2 publication code	VARCHAR2(10)
61	HABTYPCD2_DESCR_ PUB_CD	Habitat type code 2 description publication code	VARCHAR2(10)
62	MIXEDCONFCD	Mixed conifer code	VARCHAR2(1)
63	VOL_LOC_GRP	Volume location group	VARCHAR2(200)
64	SITECLCDEST	Site productivity class code estimated	NUMBER(2)
65	SITETREE_TREE	Site tree tree number	NUMBER(4)
66	SITECL_METHOD	Site class method	NUMBER(2)
67	CARBON_DOWN_DEAD	Carbon in down dead	NUMBER(13,6)
68	CARBON_LITTER	Carbon in litter	NUMBER(13,6)
69	CARBON_SOIL_ORG	Carbon in soil organic material	NUMBER(13,6)
70	CARBON_STANDING_DEAD	Carbon in standing dead trees	NUMBER(13,6)
71	CARBON_UNDERSTORY_AG	Carbon in the understory aboveground	NUMBER(13,6)
72	CARBON_UNDERSTORY_BG	Carbon in the understory belowground	NUMBER(13,6)
73	CREATED_BY	Created by	VARCHAR2(30)
74	CREATED_DATE	Created date	DATE
75	CREATED_IN_INSTANCE	Created in instance	VARCHAR2(6)
76	MODIFIED_BY	Modified by	VARCHAR2(30)

	Column name	Descriptive name	Oracle data type
77	MODIFIED_DATE	Modified date	DATE
78	MODIFIED_IN_INSTANCE	Modified in instance	VARCHAR2(6)
79	CYCLE	Inventory cycle number	NUMBER(2)
80	SUBCYCLE	Inventory subcycle number	NUMBER(2)
81	SOIL_ROOTING_DEPTH_PNW	Soil rooting depth, Pacific Northwest Research Station	VARCHAR2(1)
82	GROUND_LAND_CLASS_PNW	Present ground land class, Pacific Northwest Research Station	VARCHAR2(3)
83	PLANT_STOCKABILITY_FACTOR_PNW	Plant stockability factor, Pacific Northwest Research Station	NUMBER
84	STND_COND_CD_PNWRS	Stand condition code, Pacific Northwest Research Station	NUMBER(1)
85	STND_STRUC_CD_PNWRS	Stand structure code, Pacific Northwest Research Station	NUMBER(1)
86	STUMP_CD_PNWRS	Stump code, Pacific Northwest Research Station	VARCHAR2(1)
87	FIRE_SRS	Fire, Southern Research Station	NUMBER(1)
88	GRAZING_SRS	Grazing, Southern Research Station	NUMBER(1)
89	HARVEST_TYPE1_SRS	Harvest type code 1, Southern Research Station	NUMBER(2)
90	HARVEST_TYPE2_SRS	Harvest type code 2, Southern Research Station	NUMBER(2)
91	HARVEST_TYPE3_SRS	Harvest type code 3, Southern Research Station	NUMBER(2)
92	LAND_USE_SRS	Land use, Southern Research Station	NUMBER(2)
93	OPERABILITY_SRS	Operability, Southern Research Station	NUMBER(2)
94	STAND_STRUCTURE_SRS	Stand structure, Southern Research Station	NUMBER(2)
95	NF_COND_STATUS_CD	Nonforest condition status code	NUMBER(1)
96	NF_COND_NONSAMPLE_REASN_CD	Nonforest condition nonsampled reason code	NUMBER(2)
97	CANOPY_CVR_SAMPLE_METHOD_CD	Canopy cover sample method code	NUMBER(2)
98	LIVE_CANOPY_CVR_PCT	Live canopy cover percent	NUMBER(3)
99	LIVE_MISSING_CANOPY_CVR_PCT	Live plus missing canopy cover percent	NUMBER(3)
100	NBR_LIVE_STEMS	Number of live stems	NUMBER(5)

Type of key	Column(s) order	Tables to link	Abbreviated notation
Primary	(CN)	N/A	CND_PK
Unique	(PLT_CN, CONDID)	N/A	CND_UK
Natural	(STATECD, INVYR, UNITCD, COUNTYCD, PLOT, CONDID)	N/A	CND_NAT_I
Foreign	(PLT_CN)	CONDITION to PLOT	CND_PLT_FK

1. CN — Sequence number. A unique sequence number used to identify a condition record.

2. PLT_CN Plot sequence number. Foreign key linking the condition record to the plot record.

3. INVYR Inventory year. The year that best represents when the inventory data were collected. Under the annual inventory system, a group of plots is selected each year for sampling. The selection is based on a panel system. INVYR is the year in which the majority of plots in that group were collected (plots in the group have the same panel and, if applicable, subpanel). Under periodic inventory, a reporting inventory year was selected, usually based on the year in which the majority of the plots were collected or the mid-point of the years over which the inventory spanned. For either annual or periodic inventory, INVYR is not necessarily the same as MEASYEAR.

Exceptions:
INVYR = 9999. INVYR is set to 9999 to distinguish Phase 3 plots taken by the western FIA work units that are "off subpanel." This is due to differences in measurement intervals between Phase 3 (measurement interval = 5 years) and Phase 2 (measurement interval = 10 years) plots. Only users interested in performing certain Phase 3 data analyses should access plots with this anomalous value in INVYR.

INVYR <100. INVYR <100 indicates that population estimates were derived from a pre-NIMS regional processing system and the same plot either has been or may soon be re-processed in NIMS as part of a separate evaluation. The NIMS processed copy of the plot follows the standard INVYR format. This only applies to plots collected in the South (SURVEY.RSCD = 33) with the national design or a similar regional design (PLOT.DESIGNCD = 1 or 220-233) that were collected when the inventory year was 1998 through 2005.

INVYR = 98 is equivalent to 1998 but processed through regional system
INVYR = 99 is equivalent to 1999 but processed through regional system
INVYR = 0 is equivalent to 2000 but processed through regional system
INVYR = 1 is equivalent to 2001 but processed through regional system
INVYR = 2 is equivalent to 2002 but processed through regional system
INVYR = 3 is equivalent to 2003 but processed through regional system
INVYR = 4 is equivalent to 2004 but processed through regional system
INVYR = 5 is equivalent to 2005 but processed through regional system

4. STATECD State code. Bureau of the Census Federal Information Processing Standards (FIPS) two-digit code for each State. Refer to appendix C.

5. UNITCD Survey unit code. Forest Inventory and Analysis survey unit identification number. Survey units are usually groups of counties within each State. For periodic inventories, survey units may be made up of lands of particular owners. Refer to appendix C for codes.

6. COUNTYCD — County code. The identification number for a county, parish, watershed, borough, or similar governmental unit in a State. FIPS codes from the Bureau of the Census are used. Refer to appendix C for codes.

7. PLOT — Phase 2 plot number. An identifier for a plot. Along with STATECD, INVYR, UNITCD, COUNTYCD and/or some other combination of variables, PLOT may be used to uniquely identify a plot.

8. CONDID — Condition class number. Unique identifying number assigned to each condition on a plot. A condition is initially defined by condition class status. Differences in reserved status, owner group, forest type, stand-size class, regeneration status, and stand density further define condition for forest land. Mapped nonforest conditions are also assigned numbers. At the time of the plot establishment, the condition class at plot center (the center of subplot 1) is usually designated as condition class 1. Other condition classes are assigned numbers sequentially at the time each condition class is delineated. On a plot, each sampled condition class must have a unique number that can change at remeasurement to reflect new conditions on the plot.

9. COND_STATUS_CD

Condition status code. A code indicating the basic land cover.

Code	Description
1	Forest land – Land with at least 10 percent cover (or equivalent stocking) by live trees of any size, including land that formerly had such tree cover and that will be naturally or artificially regenerated. To qualify, the area must be at least 1.0 acre in size and 120.0 feet wide. Forest land includes transition zones, such as areas between forest and nonforest lands that have at least 10 percent cover (or equivalent stocking) with live trees and forest areas adjacent to urban and built-up lands. Roadside, streamside, and shelterbelt strips of trees must have a width of at least 120 feet and continuous length of at least 363 feet to qualify as forest land. Unimproved roads and trails, streams, and clearings in forest areas are classified as forest if they are <120 feet wide or an acre in size. Tree-covered areas in agricultural production settings, such as fruit orchards, or tree-covered areas in urban settings, such as city parks, are not considered forest land. For data collected prior to annual inventory (PLOT.MANUAL <1.0), the definition for forest land may have been slightly different (for example, in the past some FIA work units used 5 percent cover rather than 10 percent.)
2	Nonforest land – Any land within the sample that does not meet the definition of accessible forest land or any of the other types of basic land covers. To qualify, the area must be at least 1.0 acre in size and 120.0 feet wide, with some exceptions that are described in the document "Forest inventory and analysis national core field guide, volume 1: field data collection procedures for Phase 2 plots, version 4.0." (http://www.fia.fs.fed.us/library/field-guides-methods-proc/.) Evidence of "possible" or future development or conversion is not considered. A nonforest land condition will remain in the sample and will be examined at the next occasion to see if it has become forest land.
3	Noncensus water – Lakes, reservoirs, ponds, and similar bodies of water 1.0 acre to 4.5 acre in size. Rivers, streams, canals, etc., 30.0 feet to 200 feet wide (1990 U.S. Census definition – U.S. Census Bureau 1994). This definition was used in the 1990 census and applied when the data became available. Earlier inventories defined noncensus water differently.

Code	Description
4	Census water – Lakes, reservoirs, ponds, and similar bodies of water 4.5 acre in size and larger; and rivers, streams, canals, etc., more than 200 feet wide (1990 U.S. Census definition; U.S. Census Bureau 1994).
5	Nonsampled - Any portion of a plot within accessible forest land that cannot be sampled is delineated as a separate condition. There is no minimum size requirement. The reason the condition was not sampled is provided in COND_NONSAMPLE_REASN_CD.

10. COND_NONSAMPLE_REASN_CD

Condition nonsampled reason code. For condition classes that cannot be sampled, one of the following reasons is recorded.

Code	Description
01	Outside U.S. boundary – Condition class is outside the U.S. border.
02	Denied access area – Access to the condition class is denied by the legal owner, or by the owner of the only reasonable route to the condition class.
03	Hazardous situation – Condition class cannot be accessed because of a hazard or danger, for example cliffs, quarries, strip mines, illegal substance plantations, temporary high water, etc.
05	Lost data – The data file was discovered to be corrupt after a panel was completed and submitted for processing. Used for the single condition that is required for this plot. This code is for office use only.
06	Lost plot – Entire plot cannot be found. Used for the single condition that is required for this plot.
07	Wrong location – Previous plot can be found, but its placement is beyond the tolerance limits for plot location. Used for the single condition that is required for this plot.
08	Skipped visit – Entire plot skipped. Used for plots that are not completed prior to the time a panel is finished and submitted for processing. Used for the single condition that is required for this plot. This code is for office use only.
09	Dropped intensified plot - Intensified plot dropped due to a change in grid density. Used for the single condition that is required for this plot. This code used only by units engaged in intensification. This code is for office use only.
10	Other – Condition class not sampled due to a reason other than one of the specific reasons listed.
11	Ocean – Condition falls in ocean water below mean high tide line.

11. RESERVCD Reserved status code. (*Core for accessible forestland; Core optional for other sampled land.*) Reserved land is land that is withdrawn by law(s) prohibiting the management of the land for the production of wood products.

Code	Description
0	Not reserved
1	Reserved

12. OWNCD Owner class code. (*Core for all accessible forestland; Core optional for other sampled land.*) A code indicating the class in which the landowner (at the time of the inventory) belongs. When PLOT.DESIGNCD = 999, OWNCD may be blank (null).

Code	Description
11	National Forest System
12	National Grassland
13	Other Forest Service
21	National Park Service
22	Bureau of Land Management
23	Fish and Wildlife Service
24	Department of Defense/Energy
25	Other federal
31	State
32	Local (County, Municipal, etc)
33	Other non-federal public
46	Undifferentiated private

The following detailed private owner land codes are not available in this database because of the FIA data confidentiality policy. Users needing this type of information should contact the FIA Spatial Data Services (SDS) group by following the instructions provided at: http://www.fia.fs.fed.us/tools-data/spatial/.

Code	Description
41	Corporate
42	Non-governmental conservation/natural resources organization
43	Unincorporated local partnership/association/club
44	Native American (Indian)
45	Individual

13. OWNGRPCD Owner group code. (*Core for all accessible forestland; Core optional for other sampled land.*) A broader group of landowner classes. When PLOT.DESIGNCD = 999, OWNGRPCD may be blank (null).

Code	Description
10	Forest Service (OWNCD 11, 12, 13)
20	Other federal (OWNCD 21, 22, 23, 24, 25)
30	State and local government (OWNCD 31, 32, 33)
40	Private (OWNCD 41, 42, 43, 44, 45, 46)

14. FORINDCD Private owner industrial status code. (*Core for all accessible forestland where owner group is private; Core optional for other sampled land where owner group is private.*) A code indicating whether the landowner owns and operates a primary wood processing plant. A primary wood processing plant is any commercial operation that originates the primary processing of wood on a regular and continuing basis. Examples include: pulp or paper mill, sawmill, panel board mill, post or pole mill.

This attribute is retained in this database for informational purposes but is intentionally left blank (null) because of the FIA data confidentiality policy. Users needing this type of information should contact the FIA Spatial Data Services (SDS) group by following the instructions provided at: http://www.fia.fs.fed.us/tools-data/spatial/.

Code	Description
0	Land is not owned by industrial owner with wood processing plant
1	Land is owned by industrial owner with wood processing plant

15. ADFORCD Administrative forest code. Identifies the administrative unit (Forest Service Region and National Forest) in which the condition is located. The first two digits of the four digit code are for the region number and the last two digits are for the Administrative National Forest number. Refer to appendix E for codes. Populated only for U.S. Forest Service lands OWNGRPCD = 10 and blank (null) for all other owners.

16. FORTYPCD Forest type code. This is the forest type used for reporting purposes. It is primarily derived using a computer algorithm, except when less than 25 percent of the plot samples a particular forest condition.

Usually, FORTYPCD equals FORTYPCDCALC. In certain situations, however, the result from the algorithm (FORTYPCDCALC) is overridden by the field call. The field-recorded forest type code (FLDTYPCD) is stored in this attribute when less than 25 percent of the plot samples the forested condition (CONDPROP_UNADJ <0.25).

In most cases, FORTYPCD is the same as the field-recorded forest type (FLDTYPCD). However, situations of under sampling may cause this attribute to differ from FLDTYPCD.

Nonstocked forest land is land that currently has less than 10 percent stocking but formerly met the definition of forest land. Forest conditions meeting this definition have few, if any, trees sampled. In these instances, the algorithm cannot assign a specific forest type and the resulting forest type code is 999, meaning nonstocked.

Refer to appendix D for the complete list of forest type codes and names.

17. FLDTYPCD Field forest type code. Forest type, assigned by the field crew, based on the tree species or species groups forming a plurality of all live stocking. The field crew assesses the forest type based on the acre of forestland around the plot, in addition to the species sampled on the condition. Refer to appendix D for a detailed list of forest type codes and names. Nonstocked forest land is land that currently has less than 10 percent stocking but formerly met the definition of forest land. When PLOT.MANUAL <2.0, forest conditions that do not meet this stocking level were coded FLDTYPCD = 999. Beginning with manual version 2.0, the crew no longer recorded nonstocked as 999. Instead, they recorded FLDSZCD = 0 to identify nonstocked conditions and entered an estimated forest type for the condition. The crew determined the estimated forest type by either recording the previous forest type on remeasured plots or, on all other plots, the most appropriate forest type to the condition based on the seedlings present or the forest type of the adjacent forest stands. Periodic inventories will differ in the way FLDTYPCD was recorded – it is best to check with individual FIA work units for details. In general, when FLDTYPCD is used for analysis, it is necessary to examine the

values of both FLDTYPCD and FLDSZCD to identify nonstocked forest land.

18. MAPDEN — Mapping density. A code indicating the relative tree density of the condition. Codes other than 1 are used as an indication that a significant difference in tree density is the only factor causing another condition to be recognized and mapped on the plot. May be blank (null) for periodic inventories.

Code	Description
1	Initial tree density class
2	Density class 2 – density different than density of the condition assigned a tree density class of 1
3	Density class 3 – density different than densities of the conditions assigned tree density classes of 1 and 2

19. STDAGE — Stand age. For annual inventories (PLOT.MANUAL ≥1.0), stand age is equal to the field-recorded stand age (FLDAGE) with some exceptions. One exception is if FLDAGE = 999, then stand age is computed. When FLDAGE = 998, STDAGE is blank (null) because no trees were cored in the field. Another exception is that RMRS always computes stand age using field-recorded tree ages from trees in the calculated stand-size class. If no tree ages are available, then RMRS sets this attribute equal to FLDAGE. For all inventories, nonstocked stands have STDAGE set to 0. In periodic inventories, stand age is determined using local procedures. Annual inventory data will contain stand ages assigned to the nearest year. For some older inventories, stand age was set to 10-year classes for stands <100 years old, 20-year age classes for stands between 100 and 200 years, and 100-year age classes if older than 200 years. These classes were converted to store the midpoint of the age class in years. Blank (null) values in the periodic data (PLOT.MANUAL <1.0) indicate that the stand was recorded as mixed age on forested condition classes. Age is difficult to measure and therefore STDAGE may have large measurement errors.

20. STDSZCD — Stand-size class code. A classification of the predominant (based on stocking) diameter class of live trees within the condition assigned using an algorithm. Large diameter trees are at least 11.0 inches diameter for hardwoods and at least 9.0 inches diameter for softwoods. Medium diameter trees are at least 5.0 inches diameter and smaller than large diameter trees. Small diameter trees are <5.0 inches diameter. When <25 percent of the plot samples the forested condition (CONDPROP_UNADJ <0.25), this attribute is set to the equivalent field-recorded stand-size class (FLDSZCD). Populated for all forest annual plots, all forest periodic plots, and all NCRS periodic plots that were measured as “nonforest with trees” (e.g., wooded pasture, windbreaks).

Code	Description
1	Large diameter – Stands with an all live stocking of at least 10 (base 100); with more than 50 percent of the stocking in medium and large diameter trees; and with the stocking of large diameter trees equal to or greater than the stocking of medium diameter trees
2	Medium diameter – Stands with an all live stocking of at least 10 (base 100); with more than 50 percent of the stocking in medium and large diameter trees; and with the stocking of large diameter trees less than the stocking of medium diameter trees
3	Small diameter – Stands with an all live stocking value of at least 10 (base 100) on which at least 50 percent of the stocking is in small diameter trees
5	Nonstocked – Forest land with all live stocking <10

21. FLDSZCD Field stand-size class code. Field-assigned classification of the predominant (based on stocking) diameter class of live trees within the condition. Blank (null) values may be present for periodic inventories.

Code	Description
0	Nonstocked – Meeting the definition of accessible land and one of the following applies (1) <10 percent stocked by trees of any size, and not classified as cover trees (see code 6), or (2) for several western woodland species where stocking standards are not available, <5 percent crown cover of trees of any size.
1	≤4.9 inches (seedlings / saplings). At least 10 percent stocking (or 5 percent crown cover if stocking standards are not available) in trees of any size; and at least 2/3 of the crown cover is in trees <5.0 inches DBH/DRC.
2	5.0 – 8.9 inches (softwoods)/ 5.0 – 10.9 inches (hardwoods). At least 10 percent stocking (or 5 percent crown cover if stocking standards are not available) in trees of any size; and at least one-third of the crown cover is in trees ≥5.0 inches DBH/DRC and the plurality of the crown cover is in softwoods 5.0 – 8.9 inches diameter and/or hardwoods 5.0 –10.9 inches DBH, and/or for western woodland trees 5.0 – 8.9 inches DRC.
3	9.0 – 19.9 inches (softwoods)/ 11.0 – 19.9 inches (hardwoods). At least 10 percent stocking (or 5 percent crown cover if stocking standards are not available) in trees of any size; and at least one-third of the crown cover is in trees ≥5.0 inches DBH/DRC and the plurality of the crown cover is in softwoods 9.0 – 19.9 inches diameter and/or hardwoods between 11.0 –19.9 inches DBH, and for western woodland trees 9.0 – 19.9 inches DRC.
4	20.0 – 39.9 inches. At least 10 percent stocking (or 5 percent crown cover if stocking standards are not available) in trees of any size; and at least one-third of the crown cover is in trees ≥5.0 inches DBH/DRC and the plurality of the crown cover is in trees 20.0 – 39.9 inches DBH.
5	40.0+ inches. At least 10 percent stocking (or 5 percent crown cover if stocking standards are not available) in trees of any size; and at least one-third of the crown cover is in trees ≥5.0 inches DBH/DRC and the plurality of the crown cover is in trees ≥40.0 inches DBH.
6	Cover trees (trees not on species list, used for plots classified as nonforest): <10 percent stocking by trees of any size, and >5 percent crown cover of species that comprise cover trees.

22. SITECLCD Site productivity class code. A classification of forest land in terms of inherent capacity to grow crops of industrial wood. Identifies the potential growth in cubic feet/acre/year and is based on the culmination of mean annual increment of fully stocked natural stands. For data stored in the database that were processed outside of NIMS, this variable may be assigned

based on the site productivity determined with the site trees, or from some other source, but the actual source of the site productivity class code is not known. For data processed with NIMS, this variable may either be assigned based on the site trees available for the plot, or, if no valid site trees are available, this variable is set equal to SITECLCDEST, a default value that is either an estimated or predicted site productivity class. If SITECLCDEST is used to populate SITECLCD, the variable SITECL_METHOD is set to 6.

Code	Description
1	225+ cubic feet/acre/year
2	165-224 cubic feet/acre/year
3	120-164 cubic feet/acre/year
4	85-119 cubic feet/acre/year
5	50-84 cubic feet/acre/year
6	20-49 cubic feet/acre/year
7	0-19 cubic feet/acre/year

23. SICOND — Site index for the condition. This represents the average total length in feet that dominant and co-dominant trees are expected to attain in well-stocked, even-aged stands at the specified base age (SIBASE). Site index is estimated for the condition by either using an individual tree or by averaging site index values that have been calculated for individual site trees (see SITETREE.SITREE) of the same species (SISP). As a result, it may be possible to find additional site index values that are not used in the calculation of SICOND in the SITETREE tables when site index has been calculated for more than one species in a condition. This attribute is blank (null) when no site index data are available.

24. SIBASE — Site index base age. The base age (sometimes called reference age), in years, of the site index curve used to derive site index. Base age may be breast height age or total age, depending on the specifications of the site index curves being used. This attribute is blank (null) when no site tree data are available.

25. SISP — Site index species code. The species upon which the site index is based. In most cases, the site index species will be one of the species that define the forest type of the condition (FORTYPCD). In cases where there are no suitable site trees of the type species, other suitable species may be used. This attribute is blank (null) when no site tree data are available.

26. STDORGCD — Stand origin code. Method of stand regeneration for the trees in the condition. An artificially regenerated stand is established by planting or artificial seeding. Populated for all forest annual plots, all forest periodic plots, and all NCRS periodic plots that were measured as “nonforest with trees” (e.g., wooded pasture, windbreaks).

Code	Description
0	Natural stands
1	Clear evidence of artificial regeneration

27. STDORGSP Stand origin species code. The species code for the predominant artificially regenerated species (only when STDORGCD = 1). See appendix F. May not be populated for some FIA work units when PLOT.MANUAL <1.0.

28. PROP_BASIS Proportion basis. A value indicating what type of fixed-size subplots were installed when this plot was sampled. This information is needed to use the proper adjustment factor for the stratum in which the plot occurs (see POP_STRATUM.ADJ_FACTOR_SUBP and POP_STRATUM.ADJ_FACTOR_MACR.) Usually 24-foot radius subplots are installed and in this case, the value for PROP_BASIS is "SUBP." However, when 58.9-foot radius macroplots are installed, the value is "MACR." This attribute is blank (null) for periodic inventories.

29. CONDPROP_UNADJ

Condition proportion unadjusted. The unadjusted proportion of the plot that is in the condition. This variable is retained for ease of area calculations. It is equal to either SUBPPROP_UNADJ or MACRPROP_UNADJ, depending on the value of PROP_BASIS. The sum of all condition proportions for a plot equals 1. When generating population area estimates, this proportion is adjusted by either the POP_STRATUM.ADJ_FACTOR_MACR or the POP_STRATUM.ADJ_FACTOR_SUBP to account for partially nonsampled plots (access denied or hazardous portions).

30. MICRPROP_UNADJ

Microplot proportion unadjusted. The unadjusted proportion of the microplots that are in the condition. The sum of all microplot condition proportions for a plot equals 1.

31. SUBPPROP_UNADJ

Subplot proportion unadjusted. The unadjusted proportion of the subplots that are in the condition. The sum of all subplot condition proportions for a plot equals 1.

32. MACRPROP_UNADJ

Macroplot proportion unadjusted. The unadjusted proportion of the macroplots that are in the condition. When macroplots are installed, the sum of all macroplot condition proportions for a plot equals 1; otherwise this attribute is left blank (null).

33. SLOPE Slope. The angle of slope, in percent, of the condition. Valid values are 000 through 155 for data collected when PLOT.MANUAL ≥1.0, and 000 through 200 on data collected when PLOT.MANUAL <1.0. When PLOT.MANUAL <1.0, the field crew measured condition slope by sighting along the average incline or decline of the condition. When PLOT.MANUAL ≥1.0, slope is collected on subplots but no longer collected for conditions. When PLOT.MANUAL ≥1.0, the slope from the subplot representing the greatest

percentage of the condition is assigned as a surrogate. In the event that two or more subplots represent the same amount of area in the condition, the slope from the lower numbered subplot is used. Populated for all forest annual plots, all forest periodic plots, and all NCRS periodic plots that were measured as "nonforest with trees" (e.g., wooded pasture, windbreaks).

34. ASPECT — Aspect. The direction of slope, to the nearest degree, for most of the condition. North is recorded as 360. When slope is <5 percent, there is no aspect and this item is set to zero. When PLOT.MANUAL <1.0, the field crew measured condition aspect. When PLOT.MANUAL ≥1.0, aspect is collected on subplots but no longer collected for conditions. NOTE: for plots measured when PLOT.MANUAL ≥1.0, the aspect from the subplot representing the greatest percentage of the condition is assigned as a surrogate. In the event that two or more subplots represent the same percentage of area in the condition, the slope from the lower numbered subplot is used. Populated for all forest annual plots, all forest periodic plots, and all NCRS periodic plots that were measured as "nonforest with trees" (e.g., wooded pasture, windbreaks).

35. PHYSCLCD — Physiographic class code. The general effect of land form, topographical position, and soil on moisture available to trees. These codes are new in annual inventory; older inventories have been updated to these codes when possible. Also populated for the NCRS periodic plots that were measured as "nonforest with trees" (e.g., wooded pasture, windbreaks).

Code	Description
	**Xeric** sites (normally low or deficient in available moisture)
11	Dry Tops – Ridge tops with thin rock outcrops and considerable exposure to sun and wind.
12	Dry Slopes – Slopes with thin rock outcrops and considerable exposure to sun and wind. Includes most mountain/steep slopes with a southern or western exposure.
13	Deep Sands – Sites with a deep, sandy surface subject to rapid loss of moisture following precipitation. Typical examples include sand hills, ridges, and flats in the South, sites along the beach and shores of lakes and streams.
19	Other Xeric – All dry physiographic sites not described above.
	**Mesic** sites (normally moderate but adequate available moisture)
21	Flatwoods – Flat or fairly level sites outside of flood plains. Excludes deep sands and wet, swampy sites.
22	Rolling Uplands – Hills and gently rolling, undulating terrain and associated small streams. Excludes deep sands, all hydric sites, and streams with associated flood plains.
23	Moist Slopes and Coves – Moist slopes and coves with relatively deep, fertile soils. Often these sites have a northern or eastern exposure and are partially shielded from wind and sun. Includes moist mountain tops and saddles.
24	Narrow flood plains/Bottomlands – Flood plains and bottomlands less than 1/4-mile in width along rivers and streams. These sites are normally well drained but are subjected to occasional flooding during periods of heavy or extended precipitation. Includes associated levees, benches, and terraces within a 1/4 mile limit. Excludes swamps, sloughs, and bogs.

Code	Description
25	Broad Floodplains/Bottomlands – Floodplains and bottomlands ¼ mile or wider along rivers and streams. These sites are normally well drained but are subjected to occasional flooding during periods of heavy or extended precipitation. Includes associated levees, benches, and terraces. Excludes swamps, sloughs, and bogs with year-round water problems.
29	Other Mesic – All moderately moist physiographic sites not described above.
	**Hydric** sites (normally abundant or overabundant moisture all year)
31	Swamps/Bogs – Low, wet, flat, forested areas usually quite extensive that are flooded for long periods except during periods of extreme drought. Excludes cypress ponds and small drains.
32	Small Drains – Narrow, stream-like, wet strands of forest land often without a well-defined stream channel. These areas are poorly drained or flooded throughout most of the year and drain the adjacent higher ground.
33	Bays and wet pocosins – Low, wet, boggy sites characterized by peaty or organic soils. May be somewhat dry during periods of extended drought. Examples include sites in the Carolina bays in the Southeast United States.
34	Beaver ponds.
35	Cypress ponds.
39	Other hydric – All other hydric physiographic sites.

36. GSSTKCD Growing-stock stocking code. A code indicating the stocking of the condition by growing-stock trees, including seedlings. Growing-stock trees are those where tree class (TREE.TREECLCD) equals 2 or, for seedlings that do not have tree class assigned where species group (TREE.SPGRPCD) is not equal to 23 (western woodland softwoods), 43 (eastern noncommercial hardwoods), and 48 (western woodland hardwoods). Populated for all forest annual plots, all forest periodic plots, and all NCRS periodic plots that were measured as “nonforest with trees” (e.g., wooded pasture, windbreaks).

Code	Description
1	Overstocked (100+ %)
2	Fully stocked (60 – 99%)
3	Medium stocked (35 – 59%)
4	Poorly stocked (10 – 34%)
5	Nonstocked (0 – 9%)

37. ALSTKCD All live stocking code. A code indicating the stocking of the condition by live trees, including seedlings. Data are in classes as listed for GSSTKCD above. May not be populated for some FIA work units when PLOT.MANUAL <1.0. Populated for all forest annual plots, all forest periodic plots, and all NCRS periodic plots that were measured as “nonforest with trees” (e.g., wooded pasture, windbreaks).

38. DSTRBCD1 Disturbance 1 code. A code indicating the kind of disturbance occurring since the last measurement or within the last 5 years for new plots. The area affected by the disturbance must be at least 1 acre in size. A significant level of disturbance (mortality or damage to 25 percent of the trees in the condition) is required. Populated for all forested conditions using the National Field Guide protocols (PLOT.MANUAL ≥1.0) and populated by some FIA work units where PLOT.MANUAL <1.0. Codes 11, 12, 21, and 22 are valid where PLOT. MANUAL ≥2.0.

Code	Description
0	No visible disturbance
10	Insect Damage
11	Insect damage to understory vegetation
12	Insect damage to trees, including seedlings and saplings
20	Disease Damage
21	Disease damage to understory vegetation
22	Disease damage to trees, including seedlings and saplings
30	Fire damage (from crown and ground fire, either prescribed or natural)
31	Ground fire damage
32	Crown fire damage
40	Animal Damage
41	Beaver (includes flooding caused by beaver)
42	Porcupine
43	Deer/ungulate
44	Bear (CORE OPTIONAL)
45	Rabbit (CORE OPTIONAL)
46	Domestic animal/livestock (includes grazing)
50	Weather Damage
51	Ice
52	Wind (includes hurricane, tornado)
53	Flooding (weather induced)
54	Drought
60	Vegetation (suppression, competition, vines)
70	Unknown / not sure / other (include in NOTES)
80	Human-caused damage – any significant threshold of human-caused damage not described in the DISTURBANCE codes or in the TREATMENT codes.
90	Geologic disturbances
91	Landslide
92	Avalanche track
93	Volcanic blast zone
94	Other geologic event
95	Earth movement / avalanches

39. DSTRBYR1 — Disturbance year 1. Year in which Disturbance 1 is estimated to have occurred. If the disturbance occurs continuously over a period of time, the value 9999 is used. Populated for all forested conditions that have some disturbance using the National Field Guide protocols (PLOT.MANUAL $\geq$1.0) and populated by some FIA work units where PLOT.MANUAL <1.0. If DISTRBCD1 = 0 then DSTRBYR1 = blank (null) or 0.

40. DSTRBCD2 — Disturbance 2 code. The second disturbance code, if the stand has experienced more than one disturbance. See DSTRBCD1 for more information. This attribute is new in annual inventory.

41. DSTRBYR2 — Disturbance year 2. The year in which Disturbance 2 occurred. See DSTRBYR1 for more information. This attribute is new in annual inventory.

42. DSTRBCD3 — Disturbance 3 code. The third disturbance code, if the stand has experienced more than two disturbances. See DSTRBCD1 for more information. This attribute is new in annual inventory.

43. DSTRBYR3 — Disturbance year 3. The year in which Disturbance 3 occurred. See DSTRBYR1 for more information. This attribute is new in annual inventory.

44. TRTCD1 Treatment code 1. A code indicating the type of stand treatment that has occurred since the last measurement or within the last 5 years for new plots. The area affected by the treatment must be at least 1 acre in size. Populated for all forested conditions using the National Field Guide protocols (PLOT.MANUAL ≥1.0) and populated by some FIA work units where PLOT.MANUAL <1.0. When PLOT.MANUAL <1.0, inventories may record treatments occurring within the last 20 years for new plots.

Code	Description
00	No observable treatment.
10	Cutting – The removal of one or more trees from a stand.
20	Site preparation – Clearing, slash burning, chopping, disking, bedding, or other practices clearly intended to prepare a site for either natural or artificial regeneration.
30	Artificial regeneration – Following a disturbance or treatment (usually cutting), a new stand where at least 50 percent of the live trees present resulted from planting or direct seeding.
40	Natural regeneration – Following a disturbance or treatment (usually cutting), a new stand where at least 50 percent of the live trees present (of any size) were established through the growth of existing trees and/or natural seeding or sprouting.
50	Other silvicultural treatment – The use of fertilizers, herbicides, girdling, pruning, or other activities (not covered by codes 10-40) designed to improve the commercial value of the residual stand, or chaining, which is a practice used on western woodlands to encourage wildlife forage.

45. TRTYR1 Treatment year 1. Year in which Stand Treatment 1 is estimated to have occurred. Populated for all forested conditions that have some treatment using the National Field Guide protocols (PLOT.MANUAL ≥1.0) and populated by some FIA work units where PLOT.MANUAL <1.0. If TRTCD1 = 00 then TRTYR1 = blank (null) or 0.

46. TRTCD2 Treatment code 2. A code indicating the type of stand treatment that has occurred since the last measurement or within the last 5 years for new plots. See TRTCD1 for more information.

47. TRTYR2 Treatment year 2. Year in which Stand Treatment 2 is estimated to have occurred. See TRTYR1 for more information.

48. TRTCD3 Treatment code 3. A code indicating the type of stand treatment that has occurred since the last measurement or within the last 5 years for new plots. See TRTCD1 for more information.

49. TRTYR3 Treatment year 3. Year in which Stand Treatment 3 is estimated to have occurred. See TRTYR1 for more information.

50. PRESNFCD Present nonforest code. (*Core for remeasured conditions that were forest before and are now nonforest; Core optional for all conditions where current condition class status is nonforest, regardless of the previous condition.*) A code indicating the current nonforest land use for conditions that were previously classified as forest but are now classified as nonforest. This attribute can be optionally recorded for all nonforest conditions, regardless of

either past land status or whether the condition has a previous measurement. May be populated when PLOT.MANUAL <1.0.

Code	Description
10	Agricultural land
11	Cropland
12	Pasture (improved through cultural practices)
13	Idle farmland
14	Orchard
15	Christmas tree plantation
16	Maintained wildlife opening*
17	Windbreak/Shelterbelt*
20	Rangeland
30	Developed
31	Cultural (business, residential, other intense human activity)
32	Rights-of-way (improved road, railway, power line)
33	Recreation (park, golf course, ski run)
34	Mining*
40	Other (undeveloped beach, marsh, bog, snow, ice)
41	Nonvegetated*
42	Wetland*
43	Beach*
45	Nonforest-Chaparral*

*These codes are currently regional. They will become national in PLOT.MANUAL = 5.0.

51. BALIVE — Basal area of live trees. Basal area in square feet per acre of all live trees over 1 inch DBH/DRC sampled in the condition.

52. FLDAGE — Field-recorded stand age. The stand age as assigned by the field crew. Based on the average total age, to the nearest year, of the trees in the field-recorded stand-size class of the condition, determined using local procedures. For non-stocked stands, 0 is stored. If all of the trees in a condition class are of a species that by regional standards cannot be bored for age (e.g., mountain mahogany, tupelo), 998 is recorded. If tree cores are not counted in the field, but are collected and sent to the office for the counting of rings, 999 is recorded.

53. ALSTK — All-live-tree stocking percent. The sum of stocking percent values of all live trees on the condition. The percent is then assigned to a stocking class, which is found in ALSTKCD. May not be populated for some FIA work units when PLOT.MANUAL <1.0.

54. GSSTK — Growing-stock stocking percent. The sum of stocking percent values of all growing-stock trees on the condition. The percent is then assigned to a stocking class, which is found in GSSTKCD. May not be populated for some FIA work units when PLOT.MANUAL <1.0.

55. FORTYPCDCALC

Forest type code calculated. Forest type is always calculated based on the tree species sampled on the condition. The forest typing algorithm is a hierarchical procedure applied to the tree species sampled on the condition. The algorithm begins by comparing the live tree stocking of softwoods and

hardwoods and continues in a stepwise fashion comparing successively smaller subgroups of the preceding aggregation of initial type groups, selecting the group with the largest aggregate stocking value. The comparison proceeds in most cases until a plurality of a forest type is identified.

Nonstocked forest land is land that currently has less than 10 percent stocking but formerly met the definition of forest land. Forest conditions meeting this definition have few, if any, trees sampled. In these instances, the algorithm cannot assign a specific forest type and the resulting forest type code is 999, meaning nonstocked. See also FORTYPCD and FLDTYPCD for other forest type attributes. Refer to appendix D for a complete list of forest type codes and names.

56. HABTYPCD1 Habitat type code 1. A code indicating the primary habitat type (or community type) for this condition. Unique codes are determined by combining both habitat type code and publication code (HABTYPCD1 and HABTYPCD1_PUB_CD). Habitat type captures information about both the overstory and understory vegetation and usually describes the vegetation that is predicted to become established after all successional stages of the ecosystem are completed without any disturbance. This code can be translated using the publication in which it was named and described (see HABTYPCD1_PUB_CD and HABTYPCD1_DESCR_PUB_CD). Only collected by certain FIA work units (SURVEY.RSCD = 22, 23, or 26).

57. HABTYPCD1_PUB_CD

Habitat type code 1 publication code. A code indicating the publication that lists the name for the habitat type code (HABTYPCD1). Publication information is documented in the REF_HABTYP_PUBLICATION table. Only used by certain FIA work units (SURVEY.RSCD = 22, 23, or 26).

58. HABTYPCD1_DESCR_PUB_CD

Habitat type code 1 description publication code. A code indicating the publication that gives a description for habitat type code 1 (HABTYPCD1). This publication may or may not be the same publication that lists the name of the habitat type (HABTYPCD1_PUB_CD). Publication information is documented in REF_HABTYP_PUBLICATION table. Only used by certain FIA work units (SURVEY.RSCD = 22, 23, or 26).

59. HABTYPCD2 Habitat type code 2. A code indicating the secondary habitat type (or community type) for this condition. Unique codes are determined by combining both habitat type code and publication code (HABTYPCD2 and HABTYPCD2_PUB_CD). Habitat type captures information about both the overstory and understory vegetation and usually describes the vegetation that is predicted to become established after all successional stages of the ecosystem are completed without any disturbance. This code can be translated using the publication in which it was named and described (see

HABTYPCD2_PUB_CD and HABTYPCD2_DESCR_PUB_CD). Only collected by certain FIA work units (SURVEY.RSCD = 22, 23, or 26).

60. HABTYPCD2_PUB_CD

Habitat type code 2 publication code. A code indicating the publication that lists the name for the habitat type code (HABTYPCD2). Publication information is documented in REF_HABTYP_PUBLICATION table. Only used by certain FIA work units (SURVEY.RSCD = 22, 23, or 26).

61. HABTYPCD2_DESCR_PUB_CD

Habitat type code 2 description publication code. A code indicating the publication that gives a description for habitat type code 2 (HABTYPCD2). This publication may or may not be the same publication that lists the name of the habitat type (HABTYPCD2_PUB_CD). Publication information is documented in REF_HABTYP_PUBLICATION table. Only used by certain FIA work units (SURVEY.RSCD = 22, 23, or 26).

62. MIXEDCONFCD

Mixed conifer site code. An indicator to show that the forest condition is a mixed conifer site in California. These sites are a complex association of ponderosa pine, sugar pine, Douglas-fir, white fir, red fir, and/or incense-cedar. Mixed conifer sites use a specific site index equation.This is a yes/no attribute. This attribute is left blank (null) for all other States. Only collected by certain FIA work units (SURVEY.RSCD = 26).

Code	Description
Y	Yes, the condition is a mixed conifer site in California
N	No, the condition is not a mixed conifer site in California

63. VOL_LOC_GRP

Volume location group. An identifier indicating what equations are used for volume, biomass, site index, etc. A volume group is usually designated for a geographic area, such as a State, multiple States, a group of counties, or an ecoregion.

Code	Description
S22LAZN	Northern Arizona Ecosections
S22LAZS	Southern Arizona Ecosections
S22LCOE	Eastern Colorado Ecosections
S22LCOW	Western Colorado Ecosections
S22LID	Idaho Ecosections
S22LMTE	Eastern Montana Ecosections
S22LMTW	Western Montana Ecosections
S22LNV	Nevada Ecosections
S22LNMN	Northern New Mexico Ecosections
S22 LNMS	Southern New Mexico Ecosections

Code	Description
S22LUTNE	Northern & Eastern Utah Ecosections
S22LUTSW	Southern & Western Utah Ecosections
S22LWYE	Eastern Wyoming Ecosections
S22LWYW	Western Wyoming Ecosections
S23LCS	Central States (IL, IN, IW, MO)
S23LLS	Lake States (MI, MN, WI)
S23LPS	Plains States (KS, NE, ND, SD)
S24	Northeastern States (CT, DE, ME, MD, MA, NH, NJ, NY, OH, PA, RI, VT, WV)
S26LCA	California other than mixed conifer forest type
S26LCAMIX	California mixed conifer forest type
S26LEOR	Eastern Oregon
S26LEWA	Eastern Washington
S26LORJJ	Oregon Jackson and Josephine Counties
S26LWOR	Western Oregon
S26LWWA	Western Washington
S26LWACF	Washington Silver Fir Zone
S27LAK1A	Coastal Alaska Southeast
S27LAK1AB	Coastal Alaska Southeast and Central
S27LAK1B	Coastal Alaska Central
S27LAK1C	Coastal Alaska Kodiak and Afognak Islands
S33	Southern Research States (excluding Puerto Rico and the Virgin Islands) – AL, AR, FL, GA, LA, KY, MS, OK, NC, SC, TN, TX, VA
S33PRVI	Puerto Rico and Virgin Islands

64. SITECLCDEST

Site productivity class code estimated. This is a field-recorded code that is an estimated or predicted indicator of site productivity. It is used as the value for SITECLCD if no valid site tree is available. When SITECLCDEST is used as SITECLCD, SITECL_METHOD is set to 6. For data stored in the database that were processed prior to the use of NIMS, this variable is blank (null). Only collected by certain FIA work units (SURVEY.RSCD = 24, 26, 27 or 33).

Code	Description
1	225+ cubic feet/acre/year
2	165-224 cubic feet/acre/year
3	120-164 cubic feet/acre/year
4	85-119 cubic feet/acre/year
5	50-84 cubic feet/acre/year
6	20-49 cubic feet/acre/year
7	0-19 cubic feet/acre/year

65. SITETREE_TREE

Site tree tree number. If an individual site index tree is used to calculate SICOND, this is the tree number of the site tree (SITETREE.TREE column) used. Only collected by certain FIA work units (SURVEY.RSCD = 23 or 33).

66. SITECL_METHOD

Site class method. A code identifying the method for determining site index or estimated site productivity class.

Code	Description
1	Tree measurement (length, age, etc.) collected during this inventory.
2	Tree measurement (length, age, etc.) collected during a previous inventory.
3	Site index or site productivity class estimated either in the field or office.
4	Site index or site productivity class estimated by the height intercept method during this inventory.
5	Site index or site productivity class estimated using multiple site trees.
6	Site index or site productivity class estimated using default values.

67. CARBON_DOWN_DEAD

Carbon in down dead. Carbon (tons per acre) of woody material >3 inches in diameter on the ground, and stumps and their roots >3 inches in diameter. Estimated from models based on geographic area, forest type, and live tree carbon density (Smith and Heath 2008). This modeled attribute is a component of the EPA's Greenhouse Gas Inventory and is not a direct sum of Phase 2 or Phase 3 measurements. This is a per acre estimate and must be multiplied by the appropriate expansion and condition proportion adjustment factor located in the POP_STRATUM table.

68. CARBON_LITTER

Carbon in litter. Carbon (tons per acre) of organic material on the floor of the forest, including fine woody debris, humus, and fine roots in the organic forest floor layer above mineral soil. Estimated from models based on geographic area, forest type, and (except for nonstocked and pinyon-juniper stands) stand age (Smith and Heath 2002). This modeled attribute is a component of the EPA's Greenhouse Gas Inventory and is not a direct sum of Phase 2 or Phase 3 measurements. This is a per acre estimate and must be multiplied by the appropriate expansion and condition proportion adjustment factor located in the POP_STRATUM table.

69. CARBON_SOIL_ORG

Carbon in organic soil. Carbon (tons per acre) in fine organic material below the soil surface to a depth of 1 meter. Does not include roots. Estimated from models based on geographic area and forest type (Smith and Heath 2008). This modeled attribute is a component of the EPA's Greenhouse Gas Inventory and is not a direct sum of Phase 2 or Phase 3 measurements. This is a per acre estimate and must be multiplied by the appropriate expansion and condition proportion adjustment factor located in the POP_STRATUM table.

70. CARBON_STANDING_DEAD

Carbon in standing dead. Carbon (tons per acre) in standing dead trees, including coarse roots, is estimated from models based on geographic area,

forest type, and (except for nonstocked stands) growing stock volume (Smith and Heath 2008). This modeled variable is a component of the EPA's Greenhouse Gas Inventory and is not a direct sum of Phase 2 or Phase 3 measurements. For most users it is preferable to calculate carbon (tons per acre) for annual inventories from the Phase 2 tree data. This is a per acre estimate and must be multiplied by the appropriate expansion and condition proportion adjustment factor located in the POP_STRATUM table.

71. CARBON_UNDERSTORY_AG

Carbon in understory aboveground. Carbon (tons per acre) in the aboveground portions of seedlings, shrubs, and bushes. Estimated from models based on geographic area, forest type, and (except for nonstocked and pinyon-juniper stands) live tree carbon density (Smith and Health 2008). This modeled attribute is a component of the EPA's Greenhouse Gas Inventory and is not a direct sum of Phase 2 or Phase 3 measurements. This is a per acre estimate and must be multiplied by the appropriate expansion and condition proportion adjustment factor located in the POP_STRATUM table.

72. CARBON_UNDERSTORY_BG

Carbon in understory belowground. Carbon (tons per acre) in the belowground portions of seedlings, shrubs, and bushes. Estimated from models based on geographic area, forest type, and (except for nonstocked and pinyon-juniper stands) live tree carbon density (Smith and Heath 2008). This modeled attribute is a component of the EPA's Greenhouse Gas Inventory and is not a direct sum of Phase 2 or Phase 3 measurements. This is a per acre estimate and must be multiplied by the appropriate expansion and condition proportion adjustment factor located in the POP_STRATUM table.

73. CREATED_BY Created by. The employee who created the record. This attribute is intentionally left blank in download files.

74. CREATED_DATE

Created date. The date the record was created. Date will be in the form DD-MON-YYYY.

75. CREATED_IN_INSTANCE

Created in instance. The database instance in which the record was created. Each computer system has a unique database instance code and this attribute stores that information to determine on which computer the record was created.

76. MODIFIED_BY

Modified by. The employee who modified the record. This field will be blank (null) if the data have not been modified since initial creation. This attribute is intentionally left blank in download files.

77. MODIFIED_DATE

Modified date. The date the record was last modified. This field will be blank (null) if the data have not been modified since initial creation. Date will be in the form DD-MON-YYYY.

78. MODIFIED_IN_INSTANCE

Modified in instance. The database instance in which the record was modified. This field will be blank (null) if the data have not been modified since initial creation.

79. CYCLE

Inventory cycle number. A number assigned to a set of plots, measured over a particular period of time from which a State estimate using all possible plots is obtained. A cycle number >1 does not necessarily mean that information for previous cycles resides in the database. A cycle is relevant for periodic and annual inventories.

80. SUBCYCLE

Inventory subcycle number. For an annual inventory that takes n years to measure all plots, subcycle shows in which of the n years of the cycle the data were measured. Subcycle is 0 for a periodic inventory. Subcycle 99 may be used for plots that are not included in the estimation process.

81. SOIL_ROOTING_DEPTH_PNW

Soil rooting depth, Pacific Northwest Research Station. Describes the soil depth (the depth to which tree roots can penetrate) within each forest land condition class. Required for all forest condition classes. This variable is coded 1 when more than half of area in the condition class is estimated to be ≤20 inches deep. Ground pumice, decomposed granite, and sand all qualify as types of soil. Only collected by certain FIA work units (SURVEY.RSCD = 26).

Code	Description
1	≤20 inches
2	>20 inches

82. GROUND_LAND_CLASS_PNW

Present ground land class, Pacific Northwest Research Station. A refinement of forest land that distinguishes timberland and a variety of forest land types. Each code, and corresponding ground land class (GLC) name and description are listed. Only collected by certain FIA work units (SURVEY.RSCD = 26).

Code	Description
120	Timberland – Forest land that is potentially capable of producing at least 20 cubic feet/acre/year at culmination in fully stocked, natural stands (1.4 cubic meters/hectare/year) of continuous crops of trees to industrial roundwood size and quality. Industrial roundwood requires species that grow to size and quality adequate to produce lumber and other manufactured products (exclude fence posts and fuel wood that are not considered manufactured). Timberland is characterized by no severe limitations on artificial or natural restocking with species capable of producing industrial roundwood.

Code	Description
141	Other forest rocky – Other forest land that can produce tree species of industrial roundwood size and quality, but that is unmanageable because the site is steep, hazardous, and rocky, or is predominantly nonstockable rock or bedrock, with trees growing in cracks and pockets. Other forest-rocky sites may be incapable of growing continuous crops due to inability to obtain adequate regeneration success.
142	Other forest unsuitable site (wetland, subalpine, or coastal conifer scrub; California only) – Other forest land that is unsuited for growing industrial roundwood because of one of the following environment factors: willow bogs, spruce bogs, sites with high water tables or even standing water for a portion of the year, and harsh sites due to extreme climatic and soil conditions. Trees present are often extremely slow growing and deformed. Examples: whitebark pine, lodgepole, or mountain hemlock stands at timberline; shore pine along the sparkling blue Pacific Ocean (Monterey, Bishop, and Douglas-fir); willow wetlands with occasional cottonwoods present; Sitka spruce-shrub communities bordering tidal flats and channels along the coast. Includes aspen stands in high-desert areas or areas where juniper/mountain mahogany are the predominant species.
143	Other forest pinyon-juniper – Areas currently capable of 10 percent or more tree stocking with forest trees, with juniper species predominating. These areas are not now, and show no evidence of ever having been,10 percent or more stocked with trees of industrial roundwood form and quality. Stocking capabilities indicated by live juniper trees or juniper stumps and juniper snags less than 25 years dead or cut. Ten percent juniper stocking means 10 percent crown cover at stand maturity. For western woodland juniper species, ten percent stocking means 5 percent crown cover at stand maturity.
144	Other forest-oak (formally oak woodland) – Areas currently 10 percent or more stocked with forest trees, with low quality forest trees of oak, gray pine, madrone, or other hardwood species predominating, and that are not now, and show no evidence of ever having been, 10 percent or more stocked with trees of industrial roundwood form and quality. Trees on these sites are usually short, slow growing, gnarled, poorly formed, and generally suitable only for fuel wood. The following types are included: blue oak, white oak, live oak, oak-gray pine.
146	Other forest unsuitable site (Oregon and Washington only) – Other forest land that is unsuited for growing industrial roundwood because of one of the following environment factors: willow bogs, spruce bogs, sites with high water tables or even standing water for a portion of the year, and harsh sites due to climatic conditions. Trees present are often extremely slow growing and deformed. Examples: whitebark pine or mountain hemlock stands at timberline, shore pine along the Pacific Ocean, willow wetlands with occasional cottonwoods present, and Sitka spruce-shrub communities bordering tidal flats and channels along the coast. Aspen stands in high-desert areas or areas where juniper/mountain mahogany are the predominant species are considered other forest-unsuitable site.
148	Other forest-Cypress (California only) – Forest land with forest trees with cypress predominating. Shows no evidence of having had 10 percent or more cover of trees of industrial roundwood quality and species.
149	Other forest-Low Productivity (this code is calculated in the office) – Forestland capable of growing crops of trees to industrial roundwood quality, but not able to grow wood at the rate of 20 cubic feet/acre/year. Included are areas of low stocking potential and/or very low site index.
150	Other forest curlleaf mountain mahogany – Areas currently capable of 10 percent or more tree stocking with forest trees, with curlleaf mountain mahogany species predominating. These areas are not now, and show no evidence of ever having been, 10 percent or more stocked with trees of industrial roundwood form and quality; 10 percent mahogany stocking means 5 percent crown cover at stand maturity.

83. PLANT_STOCKABILITY_FACTOR_PNW

Plant stockability factor, Pacific Northwest Research Station. Some plots in PNWRS have forest land condition classes that are low site, and are incapable of attaining normal yield table levels of stocking. For such classes, potential productivity (mean annual increment at culmination) must be discounted. Most forested conditions have a default value of 1 assigned; those conditions that meet the low site criteria have a value between 0.1 and 1. Key plant indicators and plant communities are used to assign discount factors, using procedures outlined in MacLean and Bolsinger (1974) and Hanson and others (2002). Only collected by certain FIA work units (SURVEY.RSCD = 26).

84. STND_COND_CD_PNWRS

Stand condition code, Pacific Northwest Research Station. A code that best describes the condition of the stand within forest condition classes. Stand condition is defined here as “ the size, density, and species composition of a plant community following disturbance and at various time intervals after disturbance.” Information on stand condition is used in describing wildlife habitat. Only collected by certain FIA work units (SURVEY.RSCD = 26).

Code	Stand Condition	Definition
0	Not applicable	Condition class is juniper, chaparral, or curlleaf mountain mahogany forest type.
1	Grass-forb	Shrubs <40 percent crown cover and <5 feet tall; plot may range from being largely devoid of vegetation to dominance by herbaceous species (grasses and forbs); tree regeneration generally <5 feet tall and 40 percent cover.
2	Shrub	Shrubs 40 percent crown canopy or greater, of any height; trees <40 percent crown canopy and <1.0 inch DBH/DRC. When average stand diameter exceeds 1.0 inch DBH/DRC, plot is “open sapling” or “closed sapling.”
3	Open sapling, poletimber	Average stand diameter 1.0-8.9 inches DBH/DRC, and tree crown canopy poletimber <60 percent.
4	Closed sapling, pole, sawtimber	Average stand diameter is 1.0-21.0 inches DBH/DRC and crown cover is 60 percent or greater.
5	Open sawtimber	Average stand diameter is 9.0-21.0 inches DBH/DRC, and crown cover is <60 percent.
6	Large sawtimber	Average stand diameter exceeds 21.0 inches DBH/DRC; crown cover may be <100 percent; decay and decadence required for old-growth characteristics is generally lacking, successional trees required by old-growth may be lacking, and dead and down material required by old-growth is lacking.
7	Old-growth	Average stand diameter exceeds 21.0 inches DBH/DRC. Stands over 200 years old with at least two tree layers (overstory and understory), decay in living trees, snags, and down woody material. Some of the overstory layer may be composed of long-lived successional species (i.e., Douglas-fir, western redcedar).

85. STND_STRUC_CD_PNWRS

Stand structure code, Pacific Northwest Research Station. A code indicating the best overall structure of the stand. Only collected by certain FIA work units (SURVEY.RSCD = 26).

Code	Stand Structure	Definition
1	Even-aged single-storied	A single even canopy characterizes the stand. The greatest numbers of trees are in a height class represented by the average height of the stand; there are substantially fewer trees in height classes above and below this mean. The smaller trees are usually tall spindly members that have fallen behind their associates. The ages of trees usually do not differ by more than 20 years.
2	Even-aged two-storied	Stands composed of two distinct canopy layers, such as, an overstory with an understory sapling layer possibly from seed tree and shelterwood operations. This may also be true in older plantations, where shade-tolerant trees may become established. Two relatively even canopy levels can be recognized in the stand. Understory or overtopped trees are common. Neither canopy level is necessarily continuous or closed, but both canopy levels tend to be uniformly distributed across the stand. The average age of each level differs significantly from the other.
3	Uneven-aged	Theoretically, these stands contain trees of every age on a continuum from seedlings to mature canopy trees. In practice, uneven-aged stands are characterized by a broken or uneven canopy layer. Usually the largest number of trees is in the smaller diameter classes. As trees increase in diameter, their numbers diminish throughout the stand. Many times, instead of producing a negative exponential distribution of diminishing larger diameters, uneven-aged stands behave irregularly with waves of reproduction and mortality. Consider any stand with three or more structural layers as uneven-aged. Logging disturbances (examples are selection, diameter limit, and salvage cutting) will give a stand an uneven-aged structure.
4	Mosaic	At least two distinct size classes are represented and these are not uniformly distributed but are grouped in small repeating aggregations, or occur as stringers <120 feet wide, throughout the stand. Each size class aggregation is too small to be recognized and mapped as an individual stand. The aggregations may or may not be even-aged.

86. STUMP_CD_PNWRS

Stump code, Pacific Northwest Research Station. A yes/no attribute indicating whether or not stumps are present on a condition. Only collected by certain FIA work units (SURVEY.RSCD = 26).

Code	Description
Y	Yes, evidence of cutting or management exists; stumps are present
N	No, evidence of cutting was not observed; stumps are not present

87. FIRE_SRS

Fire, Southern Research Station. The presence or absence of fire on the condition since the last survey or within the last 5 years on new/replacement plots. Evidence of fire must occur within the subplot. Only collected by certain FIA work units (SURVEY.RSCD = 33).

Code	Description
0	No evidence of fire since last survey
1	Evidence of burning (either prescribed or wildfire)

88. GRAZING_SRS

Grazing, Southern Research Station. The presence or absence of domestic animal grazing on the condition since the last survey or within the last 5 years on new/replacement plots. Evidence of grazing must occur within the subplot. Only collected by certain FIA work units (SURVEY.RSCD = 33).

Code	Description
0	No evidence of livestock use (by domestic animals)
1	Evidence of grazing (including dung, tracks, trails, etc.)

89. HARVEST_TYPE1_SRS

Harvest type code 1, Southern Research Station. This variable is populated when the corresponding variable TRTCD = 10. Only collected by certain FIA work units (SURVEY.RSCD = 33).

Code	Description
11	Clearcut harvest – The removal of the majority of the merchantable trees in a stand; residual stand stocking is under 50 percent.
12	Partial harvest – Removal primarily consisting of highest quality trees. Residual consists of lower quality trees because of high grading or selection harvest. (i.e., Uneven aged, group selection, high grading, species selection)
13	Seed-tree/shelterwood harvest – Crop trees are harvested leaving seed source trees either in a shelterwood or seed tree. Also includes the final harvest of the seed trees.
14	Commercial thinning – The removal of trees (usually poletimber sized) from poletimber-sized stands leaving sufficient stocking of growing-stock trees to feature in future stand development. Also included are thinning in sawtimber-sized stands where poletimber-sized (or log-sized) trees have been removed to improve quality of those trees featured in a final harvest.
15	Timber Stand Improvement (cut trees only) – The cleaning, release or other stand improvement involving non-commercial cutting applied to an immature stand that leaves sufficient stocking.
16	Salvage cutting – The harvesting of dead or damaged trees or of trees in danger of being killed by insects, disease, flooding, or other factors in order to save their economic value.

90. HARVEST_TYPE2_SRS

Harvest type code 2, Southern Research Station. See HARVEST_TYPE1_SRS.

91. HARVEST_TYPE3_SRS

Harvest type code 3, Southern Research Station. See HARVEST_TYPE1_SRS.

92. LAND_USE_SRS

Land use, Southern Research Station. A classification indicating the present land use of the condition. Collected on all condition records where SURVEY.RSCD = 33 and PLOT.DESIGNCD = 1, 230, 231, 232, or 233, and were processed in NIMS. It may not be populated for other SRS plot designs or for SRS data that have not been processed in NIMS. Only collected by certain FIA work units (SURVEY.RSCD = 33).

Code	Description
01	Timber land (COND.SITECLCD = 1, 2, 3, 4, 5, or 6)
02	Other forest land (COND.SITECLCD = 7)
10	Agricultural land – Land managed for crops, pasture, or other agricultural use and is not better described by one of the following detailed codes. The area must be at least 1.0 acre in size and 120.0 feet wide. NOTE: Codes 14, 15 and 16 are collected only where PLOT.MANUAL ≥1. If PLOT.MANUAL <1, then codes 14 and 15 were coded 11. There was no single rule for coding maintained wildlife openings where PLOT.MANUAL <1, so code 16 may have been coded 10, 11 or 12.
11	Cropland
12	Pasture (improved through cultural practices)
13	Idle farmland
14	Orchard
15	Christmas tree plantation
16	Maintained wildlife openings
20	Rangeland – Land primarily composed of grasses, forbs, or shrubs. This includes lands vegetated naturally or artificially to provide a plant cover managed like native vegetation and does not meet the definition of pasture. The area must be at least 1.0 acre in size and 120.0 feet wide.
30	Developed – Land used primarily by humans for purposes other than forestry or agriculture and is not better described by one of the following detailed codes. NOTE: Code 30 is used to describe all developed land where PLOT.MANUAL <1. The following detailed codes only apply to PLOT.MANUAL ≥1.
31	Cultural: business, residential, and other places of intense human activity
32	Rights-of-way: improved roads, railway, power lines, maintained canal
33	Recreation: parks, skiing, golf courses
34	Mining
40	Other – Land parcels greater than 1.0 acre in size and greater than 120.0 feet wide that do not fall into one of the uses described above or below.
41	Marsh
42	Wetland
43	Beach
45	Nonforest-Chaparral
91	Census Water – Lakes, reservoirs, ponds, and similar bodies of water 4.5 acres in size and larger; and rivers, streams, canals, etc., 30 to 200 feet wide.
92	Noncensus water – Lakes, reservoirs, ponds, and similar bodies of water 1.0 acre to 4.5 acres in size. Rivers, streams, canals, etc., more than 200 feet wide.
99	Nonsampled – Condition not sampled (see COND.COND_NONSAMPLE_REASN_CD for exact reason).

93. OPERABILITY_SRS

Operability, Southern Research Station. The viability of operating logging equipment in the vicinity of the condition. The code represents the most limiting class code that occurs on each forest condition. Only collected by certain FIA work units (SURVEY.RSCD = 33).

Code	Description
0	No problems.
1	Seasonal access due to water conditions in wet weather.
2	Mixed wet and dry areas typical of multi-channeled streams punctuated with dry islands.
3	Broken terrain, cliffs, gullies, outcroppings, etc. that would severely limit equipment, access or use.
4	Year-round water problems (includes islands).
5	Slopes 20-40 percent.
6	Slope greater than 40 percent.

94. STAND_STRUCTURE_SRS

Stand structure, Southern Research Station. The description of the predominant canopy structure for the condition. Only the vertical position of the dominant and codominant trees in the stand are considered. Only collected by certain FIA work units (SURVEY.RSCD = 33).

Code	Description
0	Non-stocked – The condition is less than 10 percent stocked.
1	Single-storied – Most of the dominant/codominant tree crowns form a single canopy (i.e., most of the trees are approximately the same height).
2	Two-storied – The dominant/codominant tree crowns form two distinct canopy layers or stories.
3	Multi-storied – More than two recognizable levels characterize the crown canopy. Dominant/codominant trees of many sizes (diameters and heights) for a multilevel canopy.

95. NF_COND_STATUS_CD

Nonforest condition status code. Intentionally left blank. Will be populated in version 5.0.

96. NF_COND_NONSAMPLE_REASN_CD

Nonforest condition nonsampled reason code. Intentionally left blank. Will be populated in version 5.0.

97. CANOPY_CVR_SAMPLE_METHOD_CD

Canopy cover sample method code. Intentionally left blank. Will be populated in version 5.0.

98. LIVE_CANOPY_CVR_PCT

Live canopy cover percent. Intentionally left blank. Will be populated in version 5.0.

99. LIVE_MISSING_CANOPY_CVR_PCT

Live plus missing canopy cover percent. Intentionally left blank. Will be populated in version 5.0.

100. NBR_LIVE_STEMS

Number of live stems. Intentionally left blank. Will be populated in version 5.0.

Subplot Table (Oracle table name is SUBPLOT)

	Column name	Descriptive name	Oracle data type
1	CN	Sequence number	VARCHAR2(34)
2	PLT_CN	Plot sequence number	VARCHAR2(34)
3	PREV_SBP_CN	Previous subplot sequence number	VARCHAR2(34)
4	INVYR	Inventory year	NUMBER(4)
5	STATECD	State code	NUMBER(4)
6	UNITCD	Survey unit code	NUMBER(2)
7	COUNTYCD	County code	NUMBER(3)
8	PLOT	Phase 2 plot number	NUMBER(5)
9	SUBP	Subplot number	NUMBER(3)
10	SUBP_STATUS_CD	Subplot/macroplot status code	NUMBER(1)
11	POINT_NONSAMPLE_REASN_CD	Point nonsampled reason code	NUMBER(2)
12	MICRCOND	Microplot center condition	NUMBER(1)
13	SUBPCOND	Subplot center condition	NUMBER(1)
14	MACRCOND	Macroplot center condition	NUMBER(1)
15	CONDLIST	Subplot/macroplot condition list	NUMBER(4)
16	SLOPE	Subplot slope	NUMBER(3)
17	ASPECT	Subplot aspect	NUMBER(3)
18	WATERDEP	Snow/water depth	NUMBER(2,1)
19	P2A_GRM_FLG	Periodic to annual growth, removal, and mortality flag	VARCHAR2(1)
20	CREATED_BY	Created by	VARCHAR2(30)
21	CREATED_DATE	Created date	DATE
22	CREATED_IN_INSTANCE	Created in instance	VARCHAR2(6)
23	MODIFIED_BY	Modified by	VARCHAR2(30)
24	MODIFIED_DATE	Modified date	DATE
25	MODIFIED_IN_INSTANCE	Modified in instance	VARCHAR2(6)
26	CYCLE	Inventory cycle number	NUMBER(2)
27	SUBCYCLE	Inventory subcycle number	NUMBER(2)
28	ROOT_DIS_SEV_CD_PNWRS	Root disease severity rating code, Pacific Northwest Research Station	NUMBER(1)
29	NF_SUBP_STATUS_CD	Nonforest subplot status code	NUMBER(1)
30	NF_SUBP_NONSAMPLE_REASN_CD	Nonforest subplot nonsampled reason code	NUMBER(2)
31	P2VEG_SUBP_STATUS_CD	P2 vegetation subplot status code	NUMBER(1)
32	P2VEG_SUBP_NONSAMPLE_REASN_CD	P2 vegetation subplot nonsampled reason code	NUMBER(2)
33	INVASIVE_SUBP_STATUS_CD	Invasive subplot status code	NUMBER(1)
34	INVASIVE_NONSAMPLE_REASN_CD	Invasive nonsampled reason code	NUMBER(2)

Type of key	Column(s) order	Tables to link	Abbreviated notation
Primary	(CN)	N/A	SBP_PK
Unique	(PLT_CN, SUBP)	N/A	SBP_UK
Natural	(STATECD, INVYR, UNITCD, COUNTYCD, PLOT, SUBP)	N/A	SBP_NAT_I
Foreign	(PLT_CN, MICRCOND)	SUBPLOT to COND	SBP_CND_FK2
	(PLT_CN, MACRCOND)	SUBPLOT to COND	SBP_CND_FK3
	(PLT_CN, SUBPCOND)	SUBPLOT to COND	SBP_CND_FK
	(PLT_CN)	SUBPLOT to PLOT	SBP_PLT_FK

Note: The SUBPLOT record may not exist for some periodic inventory data.

1. CN — Sequence number. A unique sequence number used to identify a subplot record.

2. PLT_CN — Plot sequence number. Foreign key linking the subplot record to the plot record.

3. PREV_SBP_CN

   Previous subplot sequence number. Foreign key linking the subplot record to the previous inventory's subplot record for this subplot. Only populated on annual remeasured plots.

4. INVYR — Inventory year. The year that best represents when the inventory data were collected. Under the annual inventory system, a group of plots is selected each year for sampling. The selection is based on a panel system. INVYR is the year in which the majority of plots in that group were collected (plots in the group have the same panel and, if applicable, subpanel). Under periodic inventory, a reporting inventory year was selected, usually based on the year in which the majority of the plots were collected or the mid-point of the years over which the inventory spanned. For either annual or periodic inventory, INVYR is not necessarily the same as MEASYEAR.

   Exceptions:
   INVYR = 9999. INVYR is set to 9999 to distinguish Phase 3 plots taken by the western FIA work units that are "off subpanel." This is due to differences in measurement intervals between Phase 3 (measurement interval = 5 years) and Phase 2 (measurement interval = 10 years) plots. Only users interested in performing certain Phase 3 data analyses should access plots with this anomalous value in INVYR.

   INVYR <100. INVYR <100 indicates that population estimates were derived from a pre-NIMS regional processing system and the same plot either has been or may soon be re-processed in NIMS as part of a separate evaluation. The NIMS processed copy of the plot follows the standard INVYR format. This only applies to plots collected in the South (SURVEY.RSCD = 33) with the national design or a similar regional design (PLOT.DESIGNCD = 1 or

220-233) that were collected when the inventory year was 1998 through 2005.

INVYR = 98 is equivalent to 1998 but processed through regional system
INVYR = 99 is equivalent to 1999 but processed through regional system
INVYR = 0 is equivalent to 2000 but processed through regional system
INVYR = 1 is equivalent to 2001 but processed through regional system
INVYR = 2 is equivalent to 2002 but processed through regional system
INVYR = 3 is equivalent to 2003 but processed through regional system
INVYR = 4 is equivalent to 2004 but processed through regional system
INVYR = 5 is equivalent to 2005 but processed through regional system

5. STATECD — State code. Bureau of the Census Federal Information Processing Standards (FIPS) two-digit code for each State. Refer to appendix C.

6. UNITCD — Survey unit code. Forest Inventory and Analysis survey unit identification number. Survey units are usually groups of counties within each State. For periodic inventories, Survey units may be made up of lands of particular owners. Refer to appendix C for codes.

7. COUNTYCD — County code. The identification number for a county, parish, watershed, borough, or similar governmental unit in a State. FIPS codes from the Bureau of the Census are used. Refer to appendix C for codes.

8. PLOT — Phase 2 plot number. An identifier for a plot. Along with STATECD, INVYR, UNITCD, COUNTYCD and/or some other combinations of variables, PLOT may be used to uniquely identify a plot.

9. SUBP — Subplot number. The number assigned to the subplot. The national plot design (PLOT.DESIGNCD = 1) has subplot number values of 1 through 4. Other plot designs have various subplot number values. See PLOT.DESIGNCD and appendix B for information about plot designs. For more explanation about SUBP, contact the appropriate FIA work unit (table 6).

10. SUBP_STATUS_CD

Subplot/macroplot status code. A code indicating whether forest land was sampled on the subplot/macroplot or not. May be blank (null) in periodic inventories.

Code	Description
1	Sampled – at least one accessible forest land condition present on subplot.
2	Sampled – no accessible forest land condition present on subplot.
3	Nonsampled.

11. POINT_NONSAMPLE_REASN_CD

Point nonsampled reason code. For entire subplots (or macroplots) that cannot be sampled, one of the following reasons is recorded.

Code	Description
01	Outside U.S. boundary – Entire subplot (or macroplot) is outside of the U.S. border.
02	Denied access area – Access to the entire subplot (or macroplot) is denied by the legal owner, or by the owner of the only reasonable route to the subplot (or macroplot).
03	Hazardous situation – Entire subplot (or macroplot) cannot be accessed because of a hazard or danger, for example cliffs, quarries, strip mines, illegal substance plantations, high water, etc.
04	Time limitation – Entire subplot (or macroplot) cannot be sampled due to a time restriction. This code is reserved for areas with limited access, and in situations where it is imperative for the crew to leave before the plot can be completed (e.g., scheduled helicopter rendezvous).
05	Lost data – The plot data file was discovered to be corrupt after a panel was completed and submitted for processing. This code is assigned to entire plots or full subplots that could not be processed.
06	Lost plot – Entire plot cannot be found. Used for the four subplots that are required for this plot.
07	Wrong location – Previous plot can be found, but its placement is beyond the tolerance limits for plot location. Used for the four subplots that are required for this plot.
08	Skipped visit – Entire plot skipped. Used for plots that are not completed prior to the time a panel is finished and submitted for processing. Used for the four subplots that are required for this plot. This code is for office use only.
09	Dropped intensified plot - Intensified plot dropped due to a change in grid density. Used for the four subplots that are required for this plot. This code used only by units engaged in intensification. This code is for office use only.
10	Other – Entire subplot (or macroplot) not sampled due to a reason other than one of the specific reasons already listed.
11	Ocean – Subplot/macroplot falls in ocean water below mean high tide line.

12. MICRCOND Microplot center condition. Condition number for the condition at the center of the microplot.

13. SUBPCOND Subplot center condition. Condition number for the condition at the center of the subplot.

14. MACRCOND Macroplot center condition. Condition number for the condition at the center of the macroplot. Blank (null) if macroplot is not measured.

15. CONDLIST Subplot/macroplot condition list. (*Core optional*.) This is a listing of all condition classes located within the 24.0/58.9-foot radius around the subplot/macroplot center. A maximum of four conditions is permitted on any individual subplot/macroplot. For example: 2300 means these conditions (conditions 2 and 3) are on the subplot/macroplot.

16. SLOPE Subplot slope. The angle of slope, in percent, of the subplot, determined by sighting along the average incline or decline of the subplot. If the slope changes gradually, an average slope is recorded. If the slope changes across the subplot but is predominantly of one direction, the predominant slope is recorded. Valid values are 0 through 155.

17. ASPECT Subplot aspect. The direction of slope, to the nearest degree, of the subplot, determined along the direction of slope. If the aspect changes gradually, an

average aspect is recorded. If the aspect changes across the subplot but is predominantly of one direction, the predominant aspect is recorded. North is recorded as 360. When slope is <5 percent, there is no aspect and it is recorded as 000.

18. WATERDEP Snow/water depth. The approximate depth in feet of water or snow covering the subplot. Populated for all forested subplots using the National Field Guide protocols (PLOT.MANUAL ≥1.0) and populated by some FIA work units where PLOT.MANUAL <1.0.

19. P2A_GRM_FLG

Periodic to annual growth, removal, and mortality flag. A code indicating if this subplot is part of a periodic inventory (usually from a variable-radius plot design) that is only included for the purposes of computing growth, removals and/or mortality estimates. Tree data associated with this subplot does not contribute to current estimates of such attributes as volume, biomass or number of trees. The flag is set to Y for those subplots that are needed for estimation and otherwise is left blank (null).

20. CREATED_BY Created by. The employee who created the record. This attribute is intentionally left blank in download files.

21. CREATED_DATE

Created date. The date the record was created. Date will be in the form DD-MON-YYYY.

22. CREATED_IN_INSTANCE

Created in instance. The database instance in which the record was created. Each computer system has a unique database instance code and this attribute stores that information to determine on which computer the record was created.

23. MODIFIED_BY

Modified by. The employee who modified the record. This field will be blank (null) if the data have not been modified since initial creation. This attribute is intentionally left blank in download files.

24. MODIFIED_DATE

Modified date. The date the record was last modified. This field will be blank (null) if the data have not been modified since initial creation. Date will be in the form DD-MON-YYYY.

25. MODIFIED_IN_INSTANCE

Modified in instance. The database instance in which the record was modified. This field will be blank (null) if the data have not been modified since initial creation.

26. CYCLE

Inventory cycle number. A number assigned to a set of plots, measured over a particular period of time from which a State estimate using all possible plots is obtained. A cycle number >1 does not necessarily mean that information for previous cycles resides in the database. A cycle is relevant for periodic and annual inventories.

27. SUBCYCLE

Inventory subcycle number. For an annual inventory that takes n years to measure all plots, subcycle shows in which of the n years of the cycle the data were measured. Subcycle is 0 for a periodic inventory. Subcycle 99 may be used for plots that are not included in the estimation process.

28. ROOT_DIS_SEV_CD_PNWRS

Root disease severity rating code, Pacific Northwest Research Station. The root disease severity rating that describes the degree of root disease present. Only collected by certain FIA work units (SURVEY.RSCD = 26).

Code	Description
0	No evidence of root disease visible within 50 feet of the 58.9 foot macroplot.
1	Root disease present within 50 feet of the macroplot, but no evidence of disease on the macroplot.
2	Minor evidence of root disease on the macroplot, such as suppressed tree killed by root disease, or a minor part of the overstory showing symptoms of infection. Little or no detectable reduction in canopy closure or volume.
3	Canopy reduction evident, up to 20 percent; usually as a result of death of 1 codominant tree on an otherwise fully stocked site. In absence of mortality, numerous trees showing symptoms of root disease infection.
4	Canopy reduction at least 20 percent; up to 30 percent as a result of root disease mortality. Snags and downed trees removed from canopy by disease as well as live trees with advance symptoms of disease contribute to impact.
5	Canopy reduction 30-50 percent as a result of root disease. At least half of the ground area of macroplot considered infested with evidence of root disease-killed trees. Macroplots representing mature stands with half of their volume in root disease-tolerant species usually do not go much above severity 5 because of the ameliorating effect of the disease-tolerant trees.
6	50-75 percent reduction in canopy with most of the ground area considered infested as evidenced by symptomatic trees. Much of the canopy variation in this category is generally a result of root disease-tolerant species occupying infested ground.
7	At least 75 percent canopy reduction. Macroplots that reach this severity level usually are occupied by only the most susceptible species. There are very few of the original overstory trees remaining although infested ground is often densely stocked with regeneration of susceptible species.
8	The entire macroplot falls within a definite root disease pocket with only one or very few susceptible overstory trees present.
9	The entire macroplot falls within a definite root disease pocket with no overstory trees of the susceptible species present.

29. NF_SUBP_STATUS_CD

Nonforest subplot status code. Intentionally left blank. Will be populated in version 5.0.

30. NF_SUBP_NONSAMPLE_REASN_CD

Nonforest subplot nonsampled reason code. Intentionally left blank. Will be populated in version 5.0.

31. P2VEG_SUBP_STATUS_CD

P2 vegetation subplot status code. Intentionally left blank. Will be populated in version 5.0.

32. P2VEG_SUBP_NONSAMPLE_REASN_CD

P2 vegetation subplot nonsampled reason code. Intentionally left blank. Will be populated in version 5.0.

33. INVASIVE_SUBP_STATUS_CD

Invasive subplot status code. Intentionally left blank. Will be populated in version 5.0.

34. INVASIVE_NONSAMPLE_REASN_CD

Invasive nonsampled reason code. Intentionally left blank. Will be populated in version 5.0.

Subplot Condition Table (Oracle table name is SUBP_COND)

	Column name	Descriptive name	Oracle data type
1	CN	Sequence number	VARCHAR2(34)
2	PLT_CN	Plot sequence number	VARCHAR2(34)
3	INVYR	Inventory year	NUMBER(4)
4	STATECD	State code	NUMBER(4)
5	UNITCD	Survey unit code	NUMBER(2)
6	COUNTYCD	County code	NUMBER(3)
7	PLOT	Phase 2 plot number	NUMBER(5)
8	SUBP	Subplot number	NUMBER(3)
9	CONDID	Condition class number	NUMBER(1)
10	CREATED_BY	Created by	VARCHAR2(30)
11	CREATED_DATE	Created date	DATE
12	CREATED_IN_INSTANCE	Created in instance	VARCHAR2(6)
13	MODIFIED_BY	Modified by	VARCHAR2(30)
14	MODIFIED_DATE	Modified date	DATE
15	MODIFIED_IN_INSTANCE	Modified in instance	VARCHAR2(6)
16	MICRCOND_PROP	Microplot-condition proportion	NUMBER(5,4)
17	SUBPCOND_PROP	Subplot-condition proportion	NUMBER(5,4)
18	MACRCOND_PROP	Macroplot-condition proportion	NUMBER(5,4)
19	NONFR_INCL_PCT_SUBP	Nonforest inclusions percentage of subplot	NUMBER(3)
20	NONFR_INCL_PCT_MACRO	Nonforest inclusions percentage of macroplot	NUMBER(3)
21	CYCLE	Inventory cycle number	NUMBER(2)
22	SUBCYCLE	Inventory subcycle number	NUMBER(2)

Type of key	Column(s) order	Tables to link	Abbreviated notation
Primary	(CN)	N/A	SCD_PK
Unique	(PLT_CN, SUBP, CONDID)	N/A	SCD_UK
Natural	(STATECD, INVYR, UNITCD, COUNTYCD, PLOT, SUBP, CONDID)	N/A	SCD_NAT_I
Foreign	(PLT_CN, CONDID)	SUBP_COND to COND	SCD_CND_FK
	(PLT_CN)	SUBP_COND to PLOT	SCD_PLT_FK
	(PLT_CN, SUBP)	SUBP_COND to SUBPLOT	SCD_SBP_FK

Note: The SUBP_COND record may not exist for some periodic inventory data.

1. CN — Sequence number. A unique sequence number used to identify a subplot condition record.

2. PLT_CN — Plot sequence number. Foreign key linking the subplot condition record to the plot record.

3. INVYR — Inventory year. The year that best represents when the inventory data were collected. Under the annual inventory system, a group of plots is selected each year for sampling. The selection is based on a panel system. INVYR is the year in which the majority of plots in that group were collected (plots in the group have the same panel and, if applicable, subpanel). Under periodic inventory, a reporting inventory year was selected, usually based on the year in which the majority of the plots were collected or the mid-point of the years over which the inventory spanned. For either annual or periodic inventory, INVYR is not necessarily the same as MEASYEAR.

   Exceptions:
   INVYR = 9999. INVYR is set to 9999 to distinguish Phase 3 plots taken by the western FIA work units that are "off subpanel." This is due to differences in measurement intervals between Phase 3 (measurement interval = 5 years) and Phase 2 (measurement interval = 10 years) plots. Only users interested in performing certain Phase 3 data analyses should access plots with this anomalous value in INVYR.

   INVYR <100. INVYR <100 indicates that population estimates were derived from a pre-NIMS regional processing system and the same plot either has been or may soon be re-processed in NIMS as part of a separate evaluation. The NIMS processed copy of the plot follows the standard INVYR format. This only applies to plots collected in the South (SURVEY.RSCD = 33) with the national design or a similar regional design (PLOT.DESIGNCD = 1 or 220-233) that were collected when the inventory year was 1998 through 2005.

   INVYR = 98 is equivalent to 1998 but processed through regional system
   INVYR = 99 is equivalent to 1999 but processed through regional system
   INVYR = 0 is equivalent to 2000 but processed through regional system
   INVYR = 1 is equivalent to 2001 but processed through regional system
   INVYR = 2 is equivalent to 2002 but processed through regional system
   INVYR = 3 is equivalent to 2003 but processed through regional system
   INVYR = 4 is equivalent to 2004 but processed through regional system
   INVYR = 5 is equivalent to 2005 but processed through regional system

4. STATECD — State code. Bureau of the Census Federal Information Processing Standards (FIPS) two-digit code for each State. Refer to appendix C.

5. UNITCD — Survey unit code. Forest Inventory and Analysis survey unit identification number. Survey units are usually groups of counties within each State. For periodic inventories, Survey units may be made up of lands of particular owners. Refer to appendix C for codes.

6. COUNTYCD — County code. The identification number for a county, parish, watershed, borough, or similar governmental unit in a State. FIPS codes from the Bureau of the Census are used. Refer to appendix C for codes.

7. PLOT — Phase 2 plot number. An identifier for a plot. Along with STATECD, INVYR, UNITCD, COUNTYCD and/or some other combination of variables, PLOT may be used to uniquely identify a plot.

8. SUBP — Subplot number. The number assigned to the subplot. The national plot design (PLOT.DESIGNCD = 1) has subplot number values of 1 through 4. Other plot designs have various subplot number values. See PLOT.DESIGNCD and appendix B for information about plot designs. For more explanation about SUBP, contact the appropriate FIA work unit.

9. CONDID — Condition class number. Unique identifying number assigned to each condition on a plot. A condition is initially defined by condition class status. Differences in reserved status, owner group, forest type, stand-size class, regeneration status, and stand density further define condition for forest land. Mapped nonforest conditions are also assigned numbers. At the time of the plot establishment, the condition class at plot center (the center of subplot 1) is usually designated as condition class 1. Other condition classes are assigned numbers sequentially at the time each condition class is delineated. On a plot, each sampled condition class must have a unique number that can change at remeasurement to reflect new conditions on the plot.

10. CREATED_BY — Created by. The employee who created the record. This attribute is intentionally left blank in download files.

11. CREATED_DATE

Created date. The date the record was created. Date will be in the form DD-MON-YYYY.

12. CREATED_IN_INSTANCE

Created in instance. The database instance in which the record was created. Each computer system has a unique database instance code and this attribute stores that information to determine on which computer the record was created.

13. MODIFIED_BY

Modified by. The employee who modified the record. This field will be blank (null) if the data have not been modified since initial creation. This attribute is intentionally left blank in download files.

14. MODIFIED_DATE

Modified date. The date the record was last modified. This field will be blank (null) if the data have not been modified since initial creation. Date will be in the form DD-MON-YYYY.

15. MODIFIED_IN_INSTANCE

Modified in instance. The database instance in which the record was modified. This field will be blank (null) if the data have not been modified since initial creation.

16. MICRCOND_PROP

Microplot-condition proportion. Proportion of this microplot in this condition.

17. SUBPCOND_PROP

Subplot-condition proportion. Proportion of this subplot in this condition.

18. MACRCOND_PROP

Macroplot-condition proportion. Proportion of this macroplot in this condition.

19. NONFR_INCL_PCT_SUBP

Nonforest inclusion percentage of subplot. Nonforest area estimate, expressed as a percentage, of the 24.0-foot, fixed-radius subplot present within a mapped, accessible forestland condition class in Oregon, Washington, and California. Only collected by certain FIA work units (SURVEY.RSCD = 26).

20. NONFR_INCL_PCT_MACRO

Nonforest inclusion percentage of macroplot. Nonforest area estimate, expressed as a percentage, of the 58.9-foot, fixed-radius macroplot present within a mapped, accessible forestland condition class in Oregon, Washington, and California. Only collected by certain FIA work units (SURVEY.RSCD = 26).

21. CYCLE — Inventory cycle number. A number assigned to a set of plots, measured over a particular period of time from which a State estimate using all possible plots is obtained. A cycle number >1 does not necessarily mean that information for previous cycles resides in the database. A cycle is relevant for periodic and annual inventories.

22. SUBCYCLE — Inventory subcycle number. For an annual inventory that takes n years to measure all plots, subcycle shows in which of the n years of the cycle the data were measured. Subcycle is 0 for a periodic inventory. Subcycle 99 may be used for plots that are not included in the estimation process.

Tree Table (Oracle table name is TREE)

	Column name	Descriptive name	Oracle data type
1	CN	Sequence number	VARCHAR2(34)
2	PLT_CN	Plot sequence number	VARCHAR2(34)
3	PREV_TRE_CN	Previous tree sequence number	VARCHAR2(34)
4	INVYR	Inventory year	NUMBER(4)
5	STATECD	State code	NUMBER(4)
6	UNITCD	Survey unit code	NUMBER(2)
7	COUNTYCD	County code	NUMBER(3)
8	PLOT	Phase 2 plot number	NUMBER(5)
9	SUBP	Subplot number	NUMBER(3)
10	TREE	Tree record number	NUMBER(9)
11	CONDID	Condition class number	NUMBER(1)
12	AZIMUTH	Azimuth	NUMBER(3)
13	DIST	Horizontal distance	NUMBER(4,1)
14	PREVCOND	Previous condition number	NUMBER(1)
15	STATUSCD	Status code	NUMBER(1)
16	SPCD	Species code	NUMBER
17	SPGRPCD	Species group code	NUMBER(2)
18	DIA	Current diameter	NUMBER(5,2)
19	DIAHTCD	Diameter height code	NUMBER(1)
20	HT	Total height	NUMBER(3)
21	HTCD	Height method code	NUMBER(2)
22	ACTUALHT	Actual height	NUMBER(3)
23	TREECLCD	Tree class code	NUMBER(2)
24	CR	Compacted crown ratio	NUMBER(3)
25	CCLCD	Crown class code	NUMBER(2)
26	TREEGRCD	Tree grade code	NUMBER(2)
27	AGENTCD	Cause of death (agent) code	NUMBER(2)
28	CULL	Rotten and missing cull	NUMBER(3)
29	DAMLOC1	Damage location 1	NUMBER(2)
30	DAMTYP1	Damage type 1	NUMBER(2)
31	DAMSEV1	Damage severity 1	NUMBER(1)
32	DAMLOC2	Damage location 2	NUMBER(2)
33	DAMTYP2	Damage type 2	NUMBER(2)
34	DAMSEV2	Damage severity 2	NUMBER(1)
35	DECAYCD	Decay class code	NUMBER(2)
36	STOCKING	Tree stocking	NUMBER(7,4)

	Column name	Descriptive name	Oracle data type
37	WDLDSTEM	Woodland tree species stem count	NUMBER(3)
38	VOLCFNET	Net cubic-foot volume	NUMBER(11,6)
39	VOLCFGRS	Gross cubic-foot volume	NUMBER(11,6)
40	VOLCSNET	Net cubic-foot volume in the sawlog portion	NUMBER(11,6)
41	VOLCSGRS	Gross cubic-foot volume in the sawlog portion	NUMBER(11,6)
42	VOLBFNET	Net board-foot volume in the sawlog portion	NUMBER(11,6)
43	VOLBFGRS	Gross board-foot volume in the sawlog portion	NUMBER(11,6)
44	VOLCFSND	Sound cubic-foot volume	NUMBER(11,6)
45	GROWCFGS	Net annual merchantable cubic-foot growth of a growing-stock tree on timberland	NUMBER(11,6)
46	GROWBFSL	Net annual merchantable board-foot growth of a sawtimber size tree on timberland	NUMBER(11,6)
47	GROWCFAL	Net annual sound cubic-foot growth of a live tree on timberland	NUMBER(11,6)
48	MORTCFGS	Cubic-foot volume of a growing-stock tree on timberland for mortality purposes	NUMBER(11,6)
49	MORTBFSL	Board-foot volume of a sawtimber size tree on timberland for mortality purposes	NUMBER(11,6)
50	MORTCFAL	Sound cubic-foot volume of a tree on timberland for mortality purposes	NUMBER(11,6)
51	REMVCFGS	Cubic-foot volume of a growing-stock tree on timberland for removal purposes	NUMBER(11,6)
52	REMVBFSL	Board-foot volume of a sawtimber size tree on timberland for removal purposes	NUMBER(11,6)
53	REMVCFAL	Sound cubic-foot volume of a tree on timberland for removal purposes	NUMBER(11,6)
54	DIACHECK	Diameter check code	NUMBER(2)
55	MORTYR	Mortality year	NUMBER(4)
56	SALVCD	Salvable dead code	NUMBER(2)
57	UNCRCD	Uncompacted live crown ratio	NUMBER(3)
58	CPOSCD	Crown position code	NUMBER(2)
59	CLIGHTCD	Crown light exposure code	NUMBER(2)
60	CVIGORCD	Crown vigor code (sapling)	NUMBER(2)
61	CDENCD	Crown density code	NUMBER(3)
62	CDIEBKCD	Crown dieback code	NUMBER(3)
63	TRANSCD	Foliage transparency code	NUMBER(3)
64	TREEHISTCD	Tree history code	NUMBER(3)

	Column name	Descriptive name	Oracle data type
65	DIACALC	Current diameter calculated	NUMBER(5,2)
66	BHAGE	Breast height age	NUMBER(4)
67	TOTAGE	Total age	NUMBER(4)
68	CULLDEAD	Dead cull	NUMBER(3)
69	CULLFORM	Form cull	NUMBER(3)
70	CULLMSTOP	Missing top cull	NUMBER(3)
71	CULLBF	Board-foot cull	NUMBER(3)
72	CULLCF	Cubic-foot cull	NUMBER(3)
73	BFSND	Board-foot cull soundness	NUMBER(3)
74	CFSND	Cubic-foot-cull soundness	NUMBER(3)
75	SAWHT	Sawlog height	NUMBER(2)
76	BOLEHT	Bole height	NUMBER(3)
77	FORMCL	Form class	NUMBER(1)
78	HTCALC	Current height calculated	NUMBER(3)
79	HRDWD_CLUMP_CD	Hardwood clump code	NUMBER(1)
80	SITREE	Calculated site index	NUMBER(3)
81	CREATED_BY	Created by	VARCHAR2(30)
82	CREATED_DATE	Created date	DATE
83	CREATED_IN_INSTANCE	Created in instance	VARCHAR2(6)
84	MODIFIED_BY	Modified by	VARCHAR2(30)
85	MODIFIED_DATE	Modified date	DATE
86	MODIFIED_IN_INSTANCE	Modified in instance	VARCHAR2(6)
87	MORTCD	Mortality code	NUMBER(1)
88	HTDMP	Height to diameter measurement point	NUMBER(3,1)
89	ROUGHCULL	Rough cull	NUMBER(2)
90	MIST_CL_CD	Mistletoe class code	NUMBER(1)
91	CULL_FLD	Rotten/missing cull, field recorded	NUMBER(2)
92	RECONCILECD	Reconcile code	NUMBER(1)
93	PREVDIA	Previous diameter	NUMBER(5,2)
94	FGROWCFGS	Net annual merchantable cubic-foot growth of a growing-stock tree on forest land	NUMBER(11,6)
95	FGROWBFSL	Net annual merchantable board-foot growth of a sawtimber tree on forest land	NUMBER(11,6)
96	FGROWCFAL	Net annual sound cubic-foot growth of a live tree on forest land	NUMBER(11,6)
97	FMORTCFGS	Cubic-foot volume of a growing-stock tree for mortality purposes on forest land	NUMBER(11,6)

	Column name	Descriptive name	Oracle data type
98	FMORTBFSL	Board-foot volume of a sawtimber tree for mortality purposes on forest land	NUMBER(11,6)
99	FMORTCFAL	Sound cubic-foot volume of a tree for mortality purposes on forest land	NUMBER(11,6)
100	FREMVCFGS	Cubic-foot volume of a growing-stock tree for removal purposes on forest land	NUMBER(11,6)
101	FREMVBFSL	Board-foot volume of a sawtimber size tree for removal purposes on forest land	NUMBER(11,6)
102	FREMVCFAL	Sound cubic-foot volume of the tree for removal purposes on forest land	NUMBER(11,6)
103	P2A_GRM_FLG	Periodic to annual growth, removal, and mortality flag	VARCHAR2(1)
104	TREECLCD_NERS	Tree class code, Northeastern Research Station	NUMBER(2)
105	TREECLCD_SRS	Tree class code, Southern Research Station	NUMBER(2)
106	TREECLCD_NCRS	Tree class code, North Central Research Station	NUMBER(2)
107	TREECLCD_RMRS	Tree class code, Rocky Mountain Research Station	NUMBER(2)
108	STANDING_DEAD_CD	Standing dead code	NUMBER(2)
109	PREV_STATUS_CD	Previous tree status code	NUMBER(1)
110	PREV_WDLDSTEM	Previous woodland stem count	NUMBER(3)
111	TPA_UNADJ	Trees per acre unadjusted	NUMBER(11,6)
112	TPAMORT_UNADJ	Mortality trees per acre unadjusted	NUMBER(11,6)
113	TPAREMV_UNADJ	Removal trees per acre unadjusted	NUMBER(11,6)
114	TPAGROW_UNADJ	Growth trees per acre unadjusted	NUMBER(11,6)
115	DRYBIO_BOLE	Dry biomass in the merchantable bole	NUMBER(13,6)
116	DRYBIO_TOP	Dry biomass in the top of the tree	NUMBER(13,6)
117	DRYBIO_STUMP	Dry biomass in the tree stump	NUMBER(13,6)
118	DRYBIO_SAPLING	Dry biomass of saplings	NUMBER(13,6)
119	DRYBIO_WDLD_SPP	Dry biomass of woodland tree species	NUMBER(13,6)
120	DRYBIO_BG	Dry biomass of the roots	NUMBER(13,6)
121	CARBON_AG	Carbon in the aboveground portion of the tree	NUMBER(13,6)
122	CARBON_BG	Carbon in the belowground portion of the tree	NUMBER(13,6)
123	CYCLE	Inventory cycle number	NUMBER(2)
124	SUBCYCLE	Inventory subcycle number	NUMBER(2)
125	BORED_CD_PNWRS	Tree bored code, Pacific Northwest Research Station	NUMBER(1)
126	DAMLOC1_PNWRS	Damage location 1, Pacific Northwest Research Station	NUMBER(2)
127	DAMLOC2_PNWRS	Damage location 2, Pacific Northwest Research Station	NUMBER(2)

	Column name	Descriptive name	Oracle data type
128	DIACHECK_PNWRS	Diameter check, Pacific Northwest Research Station	NUMBER(1)
129	DMG_AGENT1_CD_PNWRS	Damage agent 1, Pacific Northwest Research Station	NUMBER(2)
130	DMG_AGENT2_CD_PNWRS	Damage agent 2, Pacific Northwest Research Station	NUMBER(2)
131	DMG_AGENT3_CD_PNWRS	Damage agent 3, Pacific Northwest Research Station	NUMBER(2)
132	MIST_CL_CD_PNWRS	Leafy mistletoe class code, Pacific Northwest Research Station	NUMBER(1)
133	SEVERITY1_CD_PNWRS	Damage severity 1, Pacific Northwest Research Station for years 2001-2004	NUMBER(1)
134	SEVERITY1A_CD_PNWRS	Damage severity 1A, Pacific Northwest Research Station	NUMBER(2)
135	SEVERITY1B_CD_PNWRS	Damage severity 1B, Pacific Northwest Research Station	NUMBER(1)
136	SEVERITY2_CD_PNWRS	Damage severity 2, Pacific Northwest Research Station for years 2001-2004	NUMBER(1)
137	SEVERITY2A_CD_PNWRS	Damage severity 2A, Pacific Northwest Research Station starting in 2005	NUMBER(2)
138	SEVERITY2B_CD_PNWRS	Damage severity 2B, Pacific Northwest Research Station starting in 2005	NUMBER(1)
139	SEVERITY3_CD_PNWRS	Damage severity 3, Pacific Northwest Research Station for years 2001-2004	NUMBER(1)
140	UNKNOWN_DAMTYP1_PNWRS	Unknown damage type 1, Pacific Northwest Research Station	NUMBER(1)
141	UNKNOWN_DAMTYP2_PNWRS	Unknown damage type 2, Pacific Northwest Research Station	NUMBER(1)
142	PREV_PNTN_SRS	Previous periodic prism point, tree number, Southern Research Station	NUMBER(4)

Type of key	Column(s)	Tables to link	Abbreviated notation
Primary	(CN)	N/A	TRE_PK
Unique	(PLT_CN, SUBP, TREE)	N/A	TRE_UK
Natural	(STATECD, INVYR, UNITCD, COUNTYCD, PLOT, SUBP, TREE)	N/A	TRE_NAT_I
Foreign	(PLT_CN)	TREE to PLOT	TRE_PLT_FK

1. CN — Sequence number. A unique sequence number used to identify a tree record.

2. PLT_CN — Plot sequence number. Foreign key linking the tree record to the plot record.

3. PREV_TRE_CN

   Previous tree sequence number. Foreign key linking the tree to the previous inventory's tree record for this tree. Only populated on trees remeasured from a previous annual inventory.

4. INVYR — Inventory year. The year that best represents when the inventory data were collected. Under the annual inventory system, a group of plots is selected

each year for sampling. The selection is based on a panel system. INVYR is the year in which the majority of plots in that group were collected (plots in the group have the same panel and, if applicable, subpanel). Under periodic inventory, a reporting inventory year was selected, usually based on the year in which the majority of the plots were collected or the mid-point of the years over which the inventory spanned. For either annual or periodic inventory, INVYR is not necessarily the same as MEASYEAR.

Exceptions:
INVYR = 9999. INVYR is set to 9999 to distinguish Phase 3 plots taken by the western FIA work units that are "off subpanel." This is due to differences in measurement intervals between Phase 3 (measurement interval = 5 years) and Phase 2 (measurement interval = 10 years) plots. Only users interested in performing certain Phase 3 data analyses should access plots with this anomalous value in INVYR.

INVYR <100. INVYR <100 indicates that population estimates were derived from a pre-NIMS regional processing system and the same plot either has been or may soon be re-processed in NIMS as part of a separate evaluation. The NIMS processed copy of the plot follows the standard INVYR format. This only applies to plots collected in the South (SURVEY.RSCD = 33) with the national design or a similar regional design (PLOT.DESIGNCD = 1 or 220-233) that were collected when the inventory year was 1998 through 2005.

INVYR = 98 is equivalent to 1998 but processed through regional system
INVYR = 99 is equivalent to 1999 but processed through regional system
INVYR = 0 is equivalent to 2000 but processed through regional system
INVYR = 1 is equivalent to 2001 but processed through regional system
INVYR = 2 is equivalent to 2002 but processed through regional system
INVYR = 3 is equivalent to 2003 but processed through regional system
INVYR = 4 is equivalent to 2004 but processed through regional system
INVYR = 5 is equivalent to 2005 but processed through regional system

5. STATECD — State code. Bureau of the Census Federal Information Processing Standards (FIPS) two-digit code for each State. Refer to appendix C.

6. UNITCD — Survey unit code. Forest Inventory and Analysis survey unit identification number. Survey units are usually groups of counties within each State. For periodic inventories, Survey units may be made up of lands of particular owners. Refer to appendix C for codes.

7. COUNTYCD — County code. The identification number for a county, parish, watershed, borough, or similar governmental unit in a State. FIPS codes from the Bureau of the Census are used. Refer to appendix C for codes.

8. PLOT — Phase 2 plot number. An identifier for a plot. Along with STATECD, INVYR, UNITCD, COUNTYCD and/or some other combinations of variables, PLOT may be used to uniquely identify a plot.

9.	SUBP	Subplot number. The number assigned to the subplot. The national plot design (PLOT.DESIGNCD = 1) has subplot number values of 1 through 4. Other plot designs have various subplot number values. See PLOT.DESIGNCD and appendix B for information about plot designs. For more explanation about SUBP, contact the appropriate FIA work unit.
10.	TREE	Tree record number. A number used to uniquely identify a tree on a subplot. Tree numbers can be used to track trees when PLOT.DESIGNCD is the same between inventories.
11.	CONDID	Condition class number. Unique identifying number assigned to each condition on a plot. A condition is initially defined by condition class status. Differences in reserved status, owner group, forest type, stand-size class, regeneration status, and stand density further define condition for forest land. Mapped nonforest conditions are also assigned numbers. At the time of the plot establishment, the condition class at plot center (the center of subplot 1) is usually designated as condition class 1. Other condition classes are assigned numbers sequentially at the time each condition class is delineated. On a plot, each sampled condition class must have a unique number that can change at remeasurement to reflect new conditions on the plot.
12.	AZIMUTH	Azimuth. The direction, to the nearest degree, from subplot center (microplot center for saplings) to the center of the base of the tree (geographic center for multi-stemmed woodland species). Due north is represented by 360 degrees. This attribute is populated for live and standing dead trees in a forest condition that were measured on any of the four subplots of the national plot design. It may be populated for other tree records.
13.	DIST	Horizontal distance. The horizontal distance in feet from subplot center (microplot center for saplings) to the center of the base of the tree (geographic center for multi-stemmed woodland species). This attribute is populated for live and standing dead trees in a forest condition that were measured on any of the four subplots of the national plot design. It may be populated for other tree records.
14.	PREVCOND	Previous condition number. Identifies the condition within the plot on which the tree occurred at the previous inventory.
15.	STATUSCD	Status code. A code indicating whether the sample tree is live, cut, or dead at the time of measurement. Includes dead and cut trees, which are required to estimate aboveground biomass and net annual volume for growth, mortality, and removals. This code is not used when querying data for change estimates. Note: New and replacement plots use only codes 1 and 2.

Code	Description
0	No status – Tree is not presently in the sample (remeasurement plots only). Tree was incorrectly tallied at the previous inventory, currently not tallied due to definition or procedural change, or is not tallied due to natural causes. RECONCILECD = 5-9 required for remeasured annual inventory data but not for periodic inventory data.
1	Live tree
2	Dead tree
3	Removed – Cut and removed by direct human activity related to harvesting, silviculture or land clearing. This tree is assumed to be utilized.

16. SPCD — Species code. An FIA tree species code. Refer to appendix F for codes.

17. SPGRPCD — Species group code. A code assigned to each tree species in order to group them for reporting purposes on presentation tables. Codes and their associated names (see REF_SPECIES_GROUP.NAME) are shown in appendix G. Individual tree species and corresponding species group codes are shown in appendix F.

18. DIA — Current diameter. The current diameter (in inches) of the sample tree at the point of diameter measurement. For additional information about where the tree diameter is measured, see DIAHTCD or HTDMP. DIA for live trees contains the measured value. DIA for cut and dead trees presents problems associated with uncertainty of when the tree was cut or died as well as structural deterioration of dead trees. Consult individual FIA work units for explanations of how DIA is collected for dead and cut trees.

19. DIAHTCD — Diameter height code. A code indicating the location at which diameter was measured. For trees with code 1 (DBH), the actual measurement point may be found in HTDMP.

Code	Description
1	Breast height (DBH)
2	Root collar (DRC)

20. HT — Total height. (*Core Phase 2: ≥5.0-inch DBH/DRC live trees; Core optional Phase 2: 1.0-4.9-inch DBH/DRC live trees and ≥5.0-inch DBH/DRC standing dead trees. Core Phase 3: ≥1.0-inch DBH/DRC live trees; Core optional Phase 3: ≥5.0 inch DBH/DRC standing dead trees.*) The total length (height) of a sample tree (in feet) from the ground to the tip of the apical meristem. The total length of a tree is not always its actual length. If the main stem is broken, the actual length is measured or estimated and the missing piece is added to the actual length to estimate total length. The amount added is determined by measuring the broken piece if it can be located on the ground; otherwise it is estimated. The minimum height for timber species is 5 feet and for woodland species is 1 foot.

21. HTCD — Height method code. (*Core Phase 2: ≥5.0-inch DBH/DRC live trees; Core optional Phase 2: 1.0-4.9-inch DBH/DRC live trees and ≥5.0-inch DBH/DRC standing dead trees. Core Phase 3: ≥1.0-inch DBH/DRC live trees; Core*

optional Phase 3: ≥5.0-inch DBH/DRC standing dead trees.) A code indicating how length (height) was determined.

Code	Description
1	Field measured (total and actual length)
2	Total length visually estimated in the field, actual length measured
3	Total and actual lengths are visually estimated
4	Estimated with a model

22. ACTUALHT — Actual height. *(Core Phase 2: live and standing dead trees with broken or missing tops, ≥5.0-inch DBH/DRC; Core optional Phase 2: live trees 1.0-4.9-inch DBH/DRC with broken or missing tops; Core Phase 3: live trees ≥1.0-inch DBH/DRC (with broken or missing tops and standing dead trees ≥5.0-inch DBH/DRC [with broken or missing tops])* The length (height) of the tree to the nearest foot from ground level to the highest remaining portion of the tree still present and attached to the bole. If ACTUALHT = HT, then the tree does not have a broken top. If ACTUALHT <HT, then the tree does have a broken or missing top. The minimum height for timber species is 5 feet and for woodland species is 1 foot.

23. TREECLCD — Tree class code. A code indicating the general quality of the tree. In annual inventory, this is the tree class for both live and dead trees at the time of current measurement. In periodic inventory, for cut and dead trees, this is the tree class of the tree at the time it died or was cut. Therefore, cut and dead trees collected in periodic inventory can be coded as growing-stock.

Code	Description
2	Growing-stock – All live trees of commercial species that meet minimum merchantability standards. In general, these trees have at least one solid 8-foot section, are reasonably free of form defect on the merchantable bole, and at least 34 percent or more of the volume is merchantable. For the California, Oregon, and Washington inventories, a 26 percent or more merchantable volume standard is applied, rather than 34 percent or more. Excludes rough or rotten cull trees.
3	Rough cull – All live trees that do not now, or prospectively, have at least one solid 8-foot section, reasonably free of form defect on the merchantable bole, or have 67 percent or more of the merchantable volume cull; and more than half of this cull is due to sound dead wood cubic-foot loss or severe form defect volume loss. For the California, Oregon, and Washington inventories, 75 percent or more cull, rather than 67 percent or more cull, applies. This class also contains all trees of noncommercial species, or those species where SPGRPCD equals 23 (western woodland softwoods), 43 (eastern noncommercial hardwoods), or 48 (western woodland hardwoods). Refer to appendix F to find the species that have these SPGRPCD codes. For dead trees, this code indicates that the tree is salvable (sound).
4	Rotten cull – All live trees with 67 percent or more of the merchantable volume cull, and more than half of this cull is due to rotten or missing cubic-foot volume loss. California, Oregon, and Washington inventories use a 75 percent cutoff. For dead trees, this code indicates that the tree is nonsalvable (not sound).

24. CR — Compacted crown ratio. The percent of the tree bole supporting live, healthy foliage (the crown is ocularly compacted to fill in gaps) when compared to actual length (ACTUALHT). When PLOT.MANUAL <1.0 the variable may

have been a code, which was converted to the midpoint of the ranges represented by the codes, and is stored as a percentage.

25. CCLCD Crown class code. A code indicating the amount of sunlight received and the crown position within the canopy.

Code	Description
1	Open grown – Trees with crowns that have received full light from above and from all sides throughout all or most of their life, particularly during early development.
2	Dominant – Trees with crowns extending above the general level of the canopy and receiving full light from above and partly from the sides; larger than the average trees in the stand, and with crowns well developed, but possibly somewhat crowded on the sides.
3	Codominant – Trees with crowns forming part of the general level of the crown cover and receiving full light from above, but comparatively little from the side. Usually with medium crowns more or less crowded on the sides.
4	Intermediate – Trees shorter than those in the preceding two classes, with crowns either below or extending into the canopy formed by the dominant and codominant trees, receiving little direct light from above, and none from the sides; usually with small crowns very crowded on the sides.
5	Overtopped – Trees with crowns entirely below the general canopy level and receiving no direct light either from above or the sides.

26. TREEGRCD Tree grade code. A code indicating the quality of sawtimber-sized trees. This attribute is populated for live, growing-stock, sawtimber size trees on subplots 1-4 on national manual plots that are in a forest condition class. This attribute may be populated for other tree records that do not meet the above criteria. For example, it may be populated with the previous tree grade on dead and cut trees. Standards for tree grading are specific to species and differ slightly by research station. Only collected by certain FIA work units (SURVEY.RSCD = 23, 24, or 33). Tree grade codes range from 1 to 5.

27. AGENTCD Cause of death (agent) code. (*Core: all remeasured plots when the tree was alive at the previous visit and at revisit is dead or removed OR the tree is standing dead in the current inventory and the tree is ingrowth, through growth, or a missed live tree; Core optional: all initial plot visits when tree qualifies as a mortality tree.*) When PLOT.MANUAL ≥1.0, this variable was collected on only dead and cut trees. When PLOT.MANUAL <1.0, this variable was collected on all trees (live, dead, and cut). Cause of damage was recorded for live trees if the presence of damage or pathogen activity was serious enough to reduce the quality or vigor of the tree. When a tree was damaged by more than one agent, the most severe damage was coded. When no damage was observed on a live tree, 00 was recorded. Damage recorded for dead trees was the cause of death. When the cause of death could not be determined for a tree, 99 was recorded. Each FIA program records specific codes that may differ from one State to the next. These codes fall within the ranges listed below. For the specific codes used in a particular State, contact the FIA work unit responsible for that State (table 6).

Code	Description
00	No agent recorded (only allowed on live trees in data prior to 1999)
10	Insect
20	Disease
30	Fire
40	Animal
50	Weather
60	Vegetation (e.g., suppression, competition, vines/kudzu)
70	Unknown/not sure/other – includes death from human activity not related to silvicultural or landclearing activity (accidental, random, etc.) TREE NOTES required.
80	Silvicultural or landclearing activity (death caused by harvesting or other silvicultural activity, including girdling, chaining, etc., or to landclearing activity).

28. CULL — Rotten and missing cull. The percent of the cubic-foot volume in a live or dead tally tree that is rotten or missing. This is a calculated value that includes field-recorded cull (CULL_FLD) and any additional cull due to broken top.

29. DAMLOC1 — Damage location 1. (*Core where PLOT.MANUAL = 1.0 through 1.6; Core optional beginning with PLOT.MANUAL = 1.7.*) A code indicating where damage (meeting or exceeding a severity threshold, as defined in the field guide) is present on the tree.

Code	Description
0	No damage
1	Roots (exposed) and stump (up to 12 inches from ground level)
2	Roots, stump, and lower bole
3	Lower bole (lower half of bole between stump and base of live crown)
4	Lower and upper bole
5	Upper bole (upper half of bole between stump and base of live crown)
6	Crownstem (main stem within the live crown)
7	Branches (>1 inch diameter at junction with main stem and within the live crown)
8	Buds and shoots of current year
9	Foliage

30. DAMTYP1 — Damage type 1. (*Core where PLOT.MANUAL = 1.0 through 1.6; Core optional beginning with PLOT.MANUAL = 1.7.*) A code indicating the kind of damage (meeting or exceeding a severity threshold, as defined in the field guide) present. If DAMLOC1 = 0, then DAMTYP1 = blank (null).

Code	Description
01	Canker, gall
02	Conk, fruiting body, or sign of advanced decay
03	Open wound
04	Resinosis or gumosis
05	Crack or seam
11	Broken bole or broken root within 3 feet of bole
12	Broom on root or bole
13	Broken or dead root further than 3 feet from bole
20	Vines in the crown
21	Loss of apical dominance, dead terminal
22	Broken or dead branches

**Code**	**Description**
23	Excessive branching or brooms within the live crown
24	Damaged shoots, buds, or foliage
25	Discoloration of foliage
31	Other

31. DAMSEV1 Damage severity 1. (*Core where PLOT.MANUAL = 1.0 through 1.6; Core optional beginning with PLOT.MANUAL = 1.7.*) A code indicating how much of the tree is affected. Valid severity codes vary by damage type and damage location and must exceed a threshold value, as defined in the field guide. If DAMLOC1 = 0, then DAMSEV1 = blank (null).

**Code**	**Description**
0	01 to 09% of location affected
1	10 to 19% of location affected
2	20 to 29% of location affected
3	30 to 39% of location affected
4	40 to 49% of location affected
5	50 to 59% of location affected
6	60 to 69% of location affected
7	70 to 79% of location affected
8	80 to 89% of location affected
9	90 to 99% of location affected

32. DAMLOC2 Damage location 2. (*Core where PLOT.MANUAL = 1.0 through 1.6; Core optional beginning with PLOT.MANUAL = 1.7.*) A code indicating where secondary damage (meeting or exceeding a severity threshold, as defined in the field guide) is present. Use same codes as DAMLOC1. If DAMLOC1 = 0, then DAMLOC2 = blank (null) or 0.

33. DAMTYP2 Damage type 2. (*Core where PLOT.MANUAL = 1.0 through 1.6; Core optional beginning with PLOT.MANUAL = 1.7.*) A code indicating the kind of secondary damage (meeting or exceeding a severity threshold, as defined in the field guide) present. Use same codes as DAMTYP1. If DAMLOC1 = 0, then DAMTYP2 = blank (null).

34. DAMSEV2 Damage severity 2. (*Core where PLOT.MANUAL = 1.0 through 1.6; Core optional beginning with PLOT.MANUAL = 1.7.*) A code indicating how much of the tree is affected by the secondary damage. Valid severity codes vary by damage type and damage location and must exceed a threshold value, as defined in the field guide. Use same codes as DAMSEV1. If DAMLOC1 = 0, then DAMSEV2 = blank (null).

35. DECAYCD Decay class code. A code indicating the stage of decay in a standing dead tree. Populated where PLOT.MANUAL ≥1.0

Code	Description
1	All limbs and branches are present; the top of the crown is still present; all bark remains; sapwood is intact, with minimal decay; heartwood is sound and hard
2	There are few limbs and no fine branches; the top may be broken; a variable amount of bark remains; sapwood is sloughing with advanced decay; heartwood is sound at base but beginning to decay in the outer part of the upper bole
3	Only limb stubs exist; the top is broken; a variable amount of bark remains; sapwood is sloughing; heartwood has advanced decay in upper bole and is beginning at the base
4	Few or no limb stubs remain; the top is broken; a variable amount of bark remains; sapwood is sloughing; heartwood has advanced decay at the base and is sloughing in the upper bole
5	No evidence of branches remains; the top is broken; <20 percent of the bark remains; sapwood is gone; heartwood is sloughing throughout

36. STOCKING — Tree stocking. The stocking value computed for each live tree. Stocking values are computed using several specific species equations that were developed from normal yield tables and stocking charts. Resultant values are a function of diameter. The stocking of individual trees is used to calculate COND.GSSTK, COND.GSSTKCD, COND.ALSTK, and COND.ALSTKCD.

37. WDLDSTEM — Woodland tree species stem count. The number of live and dead stems used to calculate diameter on a woodland tree. Used for tree species where diameter is measured at the root collar. For a stem to be counted, it must have a minimum stem size of 1 inch in diameter and 1 foot in length. Blank (null) if not a woodland species.

38. VOLCFNET — Net cubic-foot volume. For timber species (trees where the diameter is measured at breast height [DBH]), this is the net volume of wood in the central stem of a sample tree ≥5.0 inches in diameter, from a 1-foot stump to a minimum 4-inch top diameter, or to where the central stem breaks into limbs all of which are <4.0 inches in diameter. For woodland species (trees where the diameter is measured at root collar [DRC]), VOLCFNET is the net volume of wood and bark from the DRC measurement point(s) to a 1½ -inch top diameter; includes branches that are at least 1½ inches in diameter along the length of the branch. This is a per tree value and must be multiplied by TPA_UNADJ to obtain per acre information. This attribute is blank (null) for trees with DIA <5.0 inches. All trees measured after 1998 with DIA ≥5.0 inches (including dead and cut trees) will have entries in this field. Does not include rotten, missing, and form cull (volume loss due to rotten, missing, and form cull defect has been deducted).

39. VOLCFGRS — Gross cubic-foot volume. For timber species (trees where the diameter is measured at breast height [DBH]), this is the total volume of wood in the central stem of sample trees ≥5.0 inches in diameter, from a 1-foot stump to a minimum 4-inch top diameter, or to where the central stem breaks into limbs all of which are <4.0 inches in diameter. For woodland species (trees where the diameter is measured at root collar [DRC]), VOLCFGRS is the total

volume of wood and bark from the DRC measurement point(s) to a 1½ -inch top diameter; includes branches that are at least 1½ inches in diameter along the length of the branch. This is a per tree value and must be multiplied by TPA_UNADJ to obtain per acre information. This attribute is blank (null) for trees with DIA <5.0 inches. All trees measured after 1998 with DIA ≥5.0 inches (including dead and cut trees) have entries in this field. Includes rotten, missing and form cull (volume loss due to rotten, missing, and form cull defect has not been deducted).

40. VOLCSNET Net cubic-foot volume in the sawlog portion. The net volume of wood in the central stem of a timber species tree of sawtimber size (9.0 inches DIA minimum for softwoods, 11.0 inches DIA minimum for hardwoods), from a 1-foot stump to a minimum top diameter, (7.0 inches for softwoods, 9.0 inches for hardwoods) or to where the central stem breaks into limbs, all of which are less than the minimum top diameter. This is a per tree value and must be multiplied by TPA_UNADJ to obtain per acre information. This attribute is blank (null) for softwood trees with DIA <9.0 inches (11.0 inches for hardwoods). All larger trees have entries in this field if they are growing-stock trees (TREECLCD = 2 and STATUSCD = 1). All rough and rotten trees (TREECLCD = 3 or 4) and dead and cut trees (STATUSCD = 2 or 3) are blank (null) in this field.

41. VOLCSGRS Gross cubic-foot volume in the sawlog portion. This is the total volume of wood in the central stem of a timber species tree of sawtimber size (9.0 inches DIA minimum for softwoods, 11.0 inches DIA minimum for hardwoods), from a 1-foot stump to a minimum top diameter (7.0 inches for softwoods, 9.0 inches for hardwoods), or to where the central stem breaks into limbs, all of which are less than the minimum top diameter. This is a per tree value and must be multiplied by TPA_UNADJ to obtain per acre information. This attribute is blank (null) for softwood trees with DIA <9.0 inches (11.0 inches for hardwoods). All larger trees have entries in this field if they are growing-stock trees (TREECLCD = 2 and STATUSCD = 1). All rough and rotten trees (TREECLCD = 3 or 4) and dead and cut trees (STATUSCD = 2 or 3) are blank (null) in this field.

42. VOLBFNET Net board-foot volume in the sawlog portion. This is the net volume (International ¼-inch rule) of wood in the central stem of a timber species tree of sawtimber size (9.0 inches DIA minimum for softwoods, 11.0 inches DIA minimum for hardwoods), from a 1-foot stump to a minimum top diameter (7.0 inches for softwoods, 9.0 inches for hardwoods), or to where the central stem breaks into limbs all of which are less than the minimum top diameter. This is a per tree value and must be multiplied by TPA_UNADJ to obtain per unit area information. This attribute is blank (null) for softwood trees with DIA <9.0 inches (11.0 inches for hardwoods). All larger trees should have entries in this field if they are growing-stock trees (TREECLCD = 2 and STATUSCD = 1). All rough and rotten trees (TREECLCD = 3 or 4) and dead and cut trees (STATUSCD = 2 or 3) are blank (null) in this field.

43. VOLBFGRS Gross board-foot volume in the sawlog portion. This is the total volume (International ¼-inch rule) of wood in the central stem of a timber species tree of sawtimber size (9.0 inches DIA minimum for softwoods, 11.0 inches DIA minimum for hardwoods), from a 1-foot stump to a minimum top diameter (7.0 inches for softwoods, 9.0 inches for hardwoods), or to where the central stem breaks into limbs all of which are less than the minimum top DIA. This is a per tree value and must be multiplied by TPA_UNADJ to obtain per unit area information. This attribute is blank (null) for softwood trees with DIA <9.0 inches (11.0 inches for hardwoods). All larger trees should have entries in this field if they are growing-stock trees (TREECLCD = 2 and STATUSCD = 1). All rough and rotten trees (TREECLCD = 3 or 4) and dead and cut trees (STATUSCD = 2 or 3) are blank (null) in this field.

44. VOLCFSND Sound cubic-foot volume. For timber species (trees where the diameter is measured at breast height [DBH]), the volume of sound wood in the central stem of a sample tree ≥5.0 inches in diameter from a 1-foot stump to a minimum 4-inch top diameter or to where the central stem breaks into limbs all of which are <4.0 inches in diameter. For woodland species (trees where the diameter is measured at root collar [DRC]), VOLCFSND is the net volume of wood and bark from the DRC measurement point(s) to a minimum 1½ -inch top diameter; includes branches that are at least 1½ inches in diameter along the length of the branch. This is a per tree value and must be multiplied by TPA_UNADJ to obtain per acre information. This attribute is blank (null) for trees with DIA <5.0 inches. All trees with DIA ≥5.0 inches (including dead trees) have entries in this field. Does not include rotten and missing cull (volume loss due to rotten and missing cull defect has been deducted).

45. GROWCFGS Net annual merchantable cubic-foot growth of a growing-stock tree on timberland. This is the net change in cubic-foot volume per year of this tree (for remeasured plots, $(V_2 - V_1)/(t_2 - t_1)$; where 1 and 2 denote the past and current measurement, respectively, V is volume, and t indicates year of measurement). Because this value is net growth, it may be a negative number. Negative growth values are usually due to mortality ($V_2 = 0$) but can also occur on live trees that have a net loss in volume because of damage, rot, broken top, or other causes. To expand to a per acre value, multiply by TPAGROW_UNADJ.

46. GROWBFSL Net annual merchantable board-foot growth of a sawtimber size tree on timberland. This is the net change in board-foot (International ¼-inch rule) volume per year of this tree (for remeasured plots $(V_2 - V_1)/(t_2 - t_1)$). Because this value is net growth, it may be a negative number. Negative growth values are usually due to mortality ($V_2 = 0$) but can also occur on live trees that have a net loss in volume because of damage, rot, broken top, or other causes. To expand to a per acre value, multiply by TPAGROW_UNADJ.

47. GROWCFAL Net annual sound cubic-foot growth of a live tree on timberland. The net change in cubic-foot volume per year of this tree (for remeasured plots

$(V_2 - V_1)/(t_2 - t_1)$). Because this value is net growth, it may be a negative number. Negative growth values are usually due to mortality ($V_2 = 0$) but can also occur on live trees that have a net loss in volume because of damage, rot, broken top, or other causes. To expand to a per acre value, multiply by TPAGROW_UNADJ. GROWCFAL differs from GROWCFGS by including all trees, regardless of tree class.

48. MORTCFGS Cubic-foot volume of a growing-stock tree on timberland for mortality purposes. Represents the cubic-foot volume of a growing-stock tree at time of death. To obtain estimates of annual per acre mortality, multiply by TPAMORT_UNADJ.

49. MORTBFSL Board-foot volume of a sawtimber size tree on timberland for mortality purposes. Represents the board-foot (International ¼-inch rule) volume of a sawtimber tree at time of mortality. To obtain estimates of annual per acre mortality, multiply by TPAMORT_UNADJ.

50. MORTCFAL Sound cubic-foot volume of a tree on timberland for mortality purposes. Represents the cubic-foot volume of the tree at time of mortality. To obtain estimates of annual per acre mortality, multiply by TPAMORT_UNADJ. MORTCFAL differs from MORTCFGS by including all trees, regardless of tree class.

51. REMVCFGS Cubic-foot volume of a growing-stock tree on timberland for removal purposes. Represents the cubic-foot volume of the tree at time of removal. To obtain estimates of annual per acre removals, multiply by TPAREMV_UNADJ.

52. REMVBFSL Board-foot volume of a sawtimber size tree on timberland for removal purposes. Represents the board-foot (International ¼-inch rule) volume of the tree at time of removal. To obtain estimates of annual per acre removals, multiply by TPAREMV_UNADJ.

53. REMVCFAL Sound cubic-foot volume of a tree on timberland for removal purposes. Represents the cubic-foot volume of the tree at time of removal. To obtain estimates of annual per acre removals, multiply by TPAREMV_UNADJ. REMVCFAL differs from REMVCFGS by including all trees, regardless of tree class.

54. DIACHECK Diameter check code. A code indicating the reliability of the diameter measurement.

Code	Description
0	Diameter accurately measured
1	Diameter estimated
2	Diameter measured at different location than previous measurement (remeasurement trees only)
5	Diameter modeled in the office (used with periodic inventories)

Note: If both codes 1 and 2 apply, code 2 is used.

55. MORTYR — Mortality year. (*Core optional.*) The estimated year in which a remeasured tree died or was cut. Populated where PLOT.MANUAL ≥1.0 and populated by some FIA work units where PLOT.MANUAL <1.0.

56. SALVCD — Salvable dead code. A standing or down dead tree considered merchantable by regional standards. Contact the appropriate FIA work unit for information on how this code is assigned for a particular State (table 6).

Code	Description
0	Dead not salvable
1	Dead salvable

57. UNCRCD — Uncompacted live crown ratio. (*Core optional Phase 2: ≥5.0-inch live trees; Core Phase 3: ≥1.0-inch live trees.*) Percentage determined by dividing the live crown length by the actual tree length. When PLOT.MANUAL <3.0 the variable was a code, which was converted to the midpoint of the ranges represented by the codes, and is stored as a percentage.

58. CPOSCD — Crown position code. (*Core on Phase 3 plots only.*) The relative position of each tree in relation to the overstory canopy.

Code	Description
1	Superstory
2	Overstory
3	Understory
4	Open canopy

59. CLIGHTCD — Crown light exposure code. (*Core optional on Phase 2 plots; Core on Phase 3 plots only.*) A code indicating the amount of light being received by the tree crown. Collected for all live trees at least 5 inches DBH/DRC. Trees with UNCRCD <35 have a maximum CLIGHTCD of 1.

Code	Description
0	The tree receives no direct sunlight because it is shaded by adjacent trees or other vegetation
1	Receives full light from the top or 1 side
2	Receives full light from the top and 1 side (or 2 sides without the top)
3	Receives full light from the top and 2 sides (or 3 sides without the top)
4	Receives full light from the top and 3 sides
5	Receives full light from the top and 4 sides

60. CVIGORCD — Crown vigor code. (*Core optional on Phase 2 plots; Core on Phase 3 plots only.*) A code indicating the vigor of sapling crowns. Collected for live trees between 1 and 4.9 inches DBH/DRC.

Code	Description
1	Saplings must have an uncompacted live crown ratio of 35 or higher, have <5 percent dieback (deer/rabbit browse is not considered as dieback but is considered missing foliage) and 80 percent or more of the foliage present is normal or at least 50 percent of each leaf is not damaged or missing. Twigs and branches that are dead because of normal shading are not included.
2	Saplings do not meet class 1 or 3 criteria. They may have any uncompacted live crown ratio, may or may not have dieback and may have between 21 and 100 percent of the foliage classified as normal.
3	Saplings may have any uncompacted live crown ratio and have 1 to 20 percent normal foliage or the percent of foliage missing combined with the percent of leaves that are over 50 percent damaged or missing should equal 80 percent or more of the live crown. Twigs and branches that are dead because of normal shading are not included. Code is also used for saplings that have no crown by definition

61. CDENCD Crown density code. (*Core optional on Phase 2 plots; Core on Phase 3 plots only.*) A code indicating how dense the tree crown is, estimated in percent classes. Collected for all live trees at least 5 inches DBH/DRC. Crown density is the amount of crown branches, foliage and reproductive structures that blocks light visibility through the crown.

Code	Description
00	0%
05	1-5%
10	6-10%
15	11-15%
.	.
.	.
.	.
95	91-95%
99	96-100%

62. CDIEBKCD Crown dieback code. (*Core optional on Phase 2 plots; Core on Phase 3 plots only.*) A code indicating the amount of recent dead material in the upper and outer portion of the crown, estimated in percent classes. Collected for all live trees at least 5 inches DBH/DRC.

Code	Description
00	0%
05	1-5%
10	6-10%
15	11-15%
.	.
.	.
.	.
95	91-95%
99	96-100%

63. TRANSCD Foliage transparency code. (*Core optional on Phase 2 plots; Core on Phase 3 plots only.*) A code indicating the amount of light penetrating the foliated portion of the crown, estimated in percent classes. Collected for all live trees at least 5 inches DBH/DRC.

Code	Description
00	0%
05	1-5%
10	6-10%
15	11-15%
.	.
.	.
.	.
95	91-95%
99	96-100%

64. TREEHISTCD Tree history code. Identifies the tree with detailed information as to whether the tree is live, dead, cut, removed due to land use change, etc. Contact the appropriate FIA work unit for the definitions (table 6). Only collected by certain FIA units (SURVEY.RSCD = 23, 24, or 33).

65. DIACALC Current diameter calculated. If the diameter is unmeasurable (i.e., the tree is cut or dead), the diameter is calculated (in inches) and stored in this variable. Only collected by certain FIA work units (SURVEY.RSCD = 23 or 33).

66. BHAGE Breast height age. The age of a live tree derived from counting tree rings from an increment core sample extracted at a height of 4.5 feet above ground. Breast height age is collected for a subset of trees and only for trees that the diameter is measured at breast height (DBH). This data item is used to calculate classification variables such as stand age. For PNWRS, one tree is sampled for BHAGE for each species, within each crown class, and for each condition class present on a plot. Age of saplings (<5.0 inches DBH) may be aged by counting branch whorls above 4.5 feet. No timber hardwood species other than red alder are bored for age. For RMRS, one tree is sampled for each species and broad diameter class present on a plot. Only collected by certain FIA work units (SURVEY.RSCD = 22 or 26) and is left blank (null) when it is not collected.

67. TOTAGE Total age. The age of a live tree derived either from counting tree rings from an increment core sample extracted at the base of a tree where diameter is measured at root collar (DRC), or for small saplings (1.0 to 2.9 inches DBH) by counting all branch whorls, or by adding a species-dependent number of years to breast height age. Total age is collected for a subset of trees and is used to calculate classification variables such as stand age. Only collected by certain FIA work units (SURVEY.RSCD = 22 or 26) and is left blank (null) when it is not collected.

68. CULLDEAD Dead cull. The percent of the gross cubic-foot volume that is cull due to sound dead material. Recorded for all trees that are at least 5.0 inches in diameter. Only collected by certain FIA work units (SURVEY.RSCD = 22). This attribute is blank (null) for trees smaller than 5 inches and is always null for the other FIA work units.

69. CULLFORM Form cull. The percent of the gross cubic-foot volume that is cull due to form defect. Recorded for live trees that are at least 5.0 inches DBH. Only

collected by certain FIA work units (SURVEY.RSCD = 22). This attribute is blank (null) for dead trees, trees smaller than 5 inches DBH, for all trees where the diameter is measured at root collar (DRC), and is always null for the other FIA work units.

70. CULLMSTOP — Missing top cull. The percent of the gross cubic-foot volume that is cull due to a missing (broken) merchantable top. Recorded for trees that are at least 5.0 inches in diameter. The volume estimate does not include any portion of the missing top that is <4.0 inches DOB (diameter outside bark). Many broken top trees may have 0% missing top cull because no merchantable volume was lost. Only collected by certain FIA work units (SURVEY.RSCD = 22). This attribute is blank (null) for trees smaller than 5 inches diameter and is always null for the other FIA work units.

71. CULLBF — Board-foot cull. The percent of the gross board-foot volume that is cull due to rot or form. Only collected by certain FIA work units (SURVEY.RSCD = 24).

72. CULLCF — Cubic-foot cull. The percent of the gross cubic-foot volume that is cull due to rot or form. Only collected by certain FIA work units (SURVEY.RSCD = 24).

73. BFSND — Board-foot-cull soundness. The percent of the board-foot cull that is sound (due to form). Only collected by certain FIA work units (SURVEY.RSCD = 24).

74. CFSND — Cubic-foot-cull soundness. The percent of the cubic-foot cull that is sound (due to form). Only collected by certain FIA work units (SURVEY.RSCD = 24).

75. SAWHT — Sawlog height. The length (height) of a tree, recorded to a 7-inch top (9-inch for hardwoods), where at least one 8-foot log, merchantable or not, is present. On broken topped trees, sawlog length is recorded to the point of the break. Only collected by certain FIA work units (SURVEY.RSCD = 24).

76. BOLEHT — Bole height. The length (height) of a tree, recorded to a 4-inch top, where at least one 4-foot section is present. Only collected by certain FIA work units (SURVEY.RSCD = 24).

77. FORMCL — Form class. A code used in calculating merchantable bole net volume. Recorded for all live hardwood trees tallied that are ≥5.0 inch DBH/DRC. Also recorded for conifers ≥5.0 inch DBH in Region 5 National Forests. Only collected by certain FIA work units (SURVEY.RSCD = 26).

Code	Description
1	First 8 feet above stump is straight
2	First 8 feet above stump is NOT straight or forked; but there is at least one straight 8-foot log elsewhere in the tree
3	No 8-foot logs anywhere in the tree now or in the future due to form

78. HTCALC — Current height calculated. If the height is unmeasurable (i.e., the tree is cut or dead), the height is calculated (in feet) and stored in this variable. Only collected by certain FIA work units (SURVEY.RSCD = 33).

79. HRDWD_CLUMP_CD

Hardwood clump code. A code sequentially assigned to each hardwood clump within each species as they are found on a subplot. Up to 9 hardwood clumps can be identified and coded within each species on each subplot. A clump is defined as having 3 or more live stems originating from a common point on the root system. Western woodland hardwood species are not evaluated for clump code. Clump code data are used to adjust stocking estimates since trees growing in clumps contribute less to stocking than do individual trees. Only collected by certain FIA work units (SURVEY.RSCD = 26).

80. SITREE Calculated site index. Computed for every tree. The site index represents the average total length (in feet) that dominant and co-dominant trees in fully-stocked, even-aged stands (of the same species as this tree) will obtain at key ages (usually 25 or 50 years). Only collected by certain FIA work units (SURVEY.RSCD = 23).

81. CREATED_BY Created by. The employee who created the record. This attribute is intentionally left blank in download files.

82. CREATED_DATE

Created date. The date the record was created. Date will be in the form DD-MON-YYYY.

83. CREATED_IN_INSTANCE

Created in instance. The database instance in which the record was created. Each computer system has a unique database instance code and this attribute stores that information to determine on which computer the record was created.

84. MODIFIED_BY

Modified by. The employee who modified the record. This field will be blank (null) if the data have not been modified since initial creation. This attribute is intentionally left blank in download files.

85. MODIFIED_DATE

Modified date. The date the record was last modified. This field will be blank (null) if the data have not been modified since initial creation. Date will be in the form DD-MON-YYYY.

86. MODIFIED_IN_INSTANCE

Modified in instance. The database instance in which the record was modified. This field will be blank (null) if the data have not been modified since initial creation.

87. MORTCD — Mortality code. (*Core optional.*) Used for a tree that was alive within past 5 years, but has died.

Code	Description
0	Tree does not qualify as mortality
1	Tree does qualify as mortality

88. HTDMP — Height to diameter measurement point. (*Core optional.*) For trees measured directly at 4.5 feet above ground, this item is blank (null). If the diameter is not measured at 4.5 feet, the actual length from the ground, to the nearest 0.1 foot, at which the diameter was measured for each tally tree, 1.0-inch DBH and larger.

89. ROUGHCULL — Rough cull. (*Core optional.*) Percentage of sound dead cull, as a percent of the merchantable bole/portion of the tree.

90. MIST_CL_CD — Mistletoe class code. (*Core optional.*) A rating of dwarf mistletoe infection. Recorded on all live conifer species except juniper. Using the Hawksworth (1979) six-class rating system, the live crown is divided into thirds, and each third is rated using the following scale: 0 is for no visible infection, 1 for <50 percent of branches infected, 2 for >50 percent of branches infected. The ratings for each third are summed together to yield the Hawksworth rating.

Code	Description
0	Hawksworth tree DMR rating of 0, no infection
1	Hawksworth tree DMR rating of 1, light infection
2	Hawksworth tree DMR rating of 2, light infection
3	Hawksworth tree DMR rating of 3, medium infection
4	Hawksworth tree DMR rating of 4, medium infection
5	Hawksworth tree DMR rating of 5, heavy infection
6	Hawksworth tree DMR rating of 6, heavy infection

91. CULL_FLD — Rotten/missing cull, field -recorded. (*Core: ≥5.0-inch live trees; Core optional: ≥5.0-inch standing dead trees.*) The percentage rotten or missing cubic-foot cull volume, estimated to the nearest 1 percent. This estimate does not include any cull estimate above actual length; therefore volume lost from a broken top is not included (see CULL for percent cull including cull from broken top). When field crews estimate volume loss (tree cull), they only consider the cull on the merchantable bole/portion of the tree, from a 1-foot stump to a 4-inch top diameter outside bark (DOB). For western woodland species, the merchantable portion is between the point of DRC measurement to a 1.5-inch top DOB.

92. RECONCILECD

Reconcile code. Recorded for remeasurement locations only. A code indicating the reason a tree either enters or is no longer a part of the inventory.

Code	Description
1	Ingrowth or reversions – either a new tally tree not qualifying as through growth or a new tree on land that was formerly nonforest and now qualifies as forest land (includes reversion or encroachments).
2	Through growth – new tally tree 5 inches DBH/DRC and larger, within the microplot, which was not missed at the previous inventory.
3	Missed live – a live tree missed at previous inventory and that is live, dead, or removed now.
4	Missed dead – a dead tree missed at previous inventory and that is dead or removed now.
5	Shrank – live tree that shrunk below threshold diameter on microplot/subplot/macroplot plot.
6	Missing (moved) – tree was correctly tallied in previous inventory, but has now moved beyond the radius of the plot due to natural causes (i.e., small earth movement, hurricane). Tree must be either live before and still alive now or dead before and dead now. If tree was live before and now dead, this is a mortality tree and should have STATUSCD = 2 (not 0).
7	Cruiser error – erroneously tallied at previous inventory
8	Procedural change – tree was tallied at the previous inventory, but is no longer tallied due to a definition or procedural change.
9	Tree was sampled before, but now the area where the tree was located is nonsampled. All trees on the nonsampled area have RECONCILECD = 9.

93. PREVDIA Previous diameter. The previous diameter (in inches) of the sample tree at the point of diameter measurement. Populated for remeasured trees.

94. FGROWCFGS Net annual merchantable cubic-foot growth of a growing-stock tree on forest land. This is the net change in cubic-foot volume per year of this tree (for remeasured plots, $(V_2 - V_1)/(t_2 - t_1)$; where 1 and 2 denote the past and current measurement, respectively, V is volume, t indicates date of measurement, and $t_2 – t_1$ = PLOT.REMPER). Because this value is net growth, it may be a negative number. Negative growth values are usually due to mortality ($V_2 = 0$) but can also occur on live trees that have a net loss in volume because of damage, rot, broken top, or other causes. To expand to a per acre value, multiply by TPAGROW_UNADJ.

95. FGROWBFSL Net annual merchantable board-foot growth of a sawtimber tree on forest land. This is the net change in board-foot (International ¼ -inch rule) volume per year of this tree (for remeasured plots $(V_2 - V_1)/(t_2-t_1)$). Because this value is net growth, it may be a negative number. Negative growth values are usually due to mortality ($V_2 = 0$) but can also occur on live trees that have a net loss in volume because of damage, rot, broken top, or other causes. To expand to a per acre value, multiply by TPAGROW_UNADJ.

96. FGROWCFAL Net annual sound cubic-foot growth of a live tree on forest land. The net change in cubic-foot volume per year of this tree (for remeasured plots

$(V_2 - V_1)/(t_2 - t_1)$). Because this value is net growth, it may be a negative number. Negative growth values are usually due to mortality ($V_2 = 0$) but can also occur on live trees that have a net loss in volume because of damage, rot, broken top, or other causes. To expand to a per acre value, multiply by TPAGROW_UNADJ. FGROWCFAL differs from FGROWCFGS by including all trees, regardless of tree class.

97. FMORTCFGS Cubic-foot volume of a growing-stock tree for mortality purposes on forest land. Represents the cubic-foot volume of a growing-stock tree at time of mortality. To obtain estimates of annual per acre mortality, multiply by TPAMORT_UNADJ.

98. FMORTBFSL Board-foot volume of a sawtimber tree for mortality purposes on forest land. Represents the board-foot (International ¼-rule) volume of a sawtimber tree at time of mortality. To obtain estimates of annual per acre mortality, multiply by TPAMORT_UNADJ.

99. FMORTCFAL Sound cubic-foot volume of a tree for mortality purposes on forest land. Represents the cubic-foot volume of the tree at time of mortality. To obtain estimates of annual per acre mortality, multiply by TPAMORT_UNADJ. FMORTCFAL differs from FMORTCFGS by including all trees, regardless of tree class.

100. FREMVCFGS Cubic-foot volume of a growing-stock tree for removal purposes on forest land. Represents the cubic-foot volume of the tree at time of removal. To obtain estimates of annual per acre removals, multiply by TPAREMV_UNADJ.

101. FREMVBFSL Board-foot volume of a sawtimber size tree for removal purposes on forest land. Represents the board-foot (International ¼-rule) volume of the tree at time of removal. To obtain estimates of annual per acre removals, multiply by TPAREMV_UNADJ.

102. FREMVCFAL Sound cubic-foot volume of the tree for removal purposes on forest land. Represents the cubic-foot volume of the tree at time of removal. To obtain estimates of annual per acre removals, multiply by TPAREMV_UNADJ. FREMVCFAL differs from FREMVCFGS by including all trees, regardless of tree class.

103. P2A_GRM_FLG

Periodic to annual growth, removal, and mortality flag. A code indicating if this tree is part of a periodic inventory (usually from a variable-radius plot design) that is only included for the purposes of computing growth, removals and/or mortality estimates. This tree does not contribute to current estimates of such attributes as volume, biomass or number of trees. The flag is set to Y for those trees that are needed for estimation and otherwise is left blank (null).

104. TREECLCD_NERS

Tree class code, Northeastern Research Station. In annual inventory, this code represents a classification of the overall quality of a tree that is 5.0 inches DBH and larger. It classifies the quality of a sawtimber tree based on the present condition, or it classifies the quality of a poletimber tree as a prospective determination (i.e., a forecast of potential quality when and if the tree becomes sawtimber size). For more detailed description, see the regional field guide. Only collected by certain FIA work units (SURVEY.RSCD = 24).

Code	Description
1	Preferred – Live tree that would be favored in cultural operations. Mature tree, that is older than the rest of the stand; has less than 20 percent total board foot cull; is expected to live for 5 more years: and is a low risk tree. In general, the tree has the following qualifications:<br>• must be free from "general" damage (i.e., damages that would now or prospectively cause a reduction of tree class, significantly deter growth, or prevent it from producing marketable products in the next 5 years).<br>• should have no more than 10 percent board-foot cull due to form defect.<br>• should have good vigor, usually indicated by a crown ratio of 30 percent or more and dominant or co-dominant.<br>• usually has a grade 1 butt log.
2	Acceptable – This class includes:<br>• live sawtimber tree that does not qualify as a preferred tree but is not a cull tree (see Rough and Rotten Cull).<br>• live poletimber tree that prospectively will not qualify as a preferred tree, but is not now or prospectively a cull tree (see Rough and Rotten Cull).
3	Rough Cull – This class includes:<br>• live sawtimber tree that currently has 67 percent or more predominantly sound board-foot cull; or does not contain one merchantable 12-foot sawlog or two non-contiguous merchantable 8-foot sawlogs.<br>• live poletimber tree that currently has 67 percent or more predominantly sound cubic-foot cull; or prospectively will have 67 percent or more predominantly sound board-foot cull; or will not contain one merchantable 12-foot sawlog or two noncontiguous merchantable 8-foot sawlogs.
4	Rotten Cull – This class includes:<br>• live sawtimber tree that currently has 67 percent or more predominantly unsound board-foot cull.<br>• live poletimber tree that currently has 67 percent or more predominantly unsound cubic-foot cull; or prospectively will have 67 percent or more predominantly unsound board-foot cull.
5	Dead – Tree that has recently died (within the last several years); but still retains many branches (including some small branches and possibly some fine twigs); and has bark that is generally tight and hard to remove from the tree.
6	Snag – Dead tree, or what remains of a dead tree, that is at least 4.5 feet tall and is missing most of its bark. This category includes a tree covered with bark that is very loose. This bark can usually be removed, often times in big strips, with very little effort. A snag is not a recently dead tree. Most often, it has been dead for several years – sometimes, for more than a decade.

105. TREECLCD_SRS

Tree class code, Southern Research Station. A code indicating the general quality of the tree. Prior to the merger of the Southern and Southeastern Research Stations (INVYR ≤1997), growing-stock (code 2) was only

assigned to species that were considered to have commercial value. Since the merger (INVYR >1997), code 2 has been applied to all tree species meeting the growing-stock form, grade, size and soundness requirements, regardless of commercial value. Only collected by certain FIA work units (SURVEY.RSCD = 33).

Code	Description
2	Growing-stock – All trees that have at least one 12-foot log or two 8-foot logs that meet grade and size requirements and at least ⅓ of the total board foot volume is merchantable. Poletimber-sized trees are evaluated based on their potential.
3	Rough cull – Trees that do not contain at least one 12-foot log or two 8-foot logs, or more than ⅓ of the total board foot volume is not merchantable, primarily due to roughness or poor form.
4	Rotten cull: Trees that do not contain at least one 12-foot log or two 8-foot logs, or more than ⅓ of the total board foot volume is not merchantable, primarily due to rotten, unsound wood.

106. TREECLCD_NCRS

Tree class code, North Central Research Station. In annual inventory, a code indicating tree suitability for timber products, or the extent of decay in the butt section of down-dead trees. It is recorded on live standing, standing-dead, and down dead trees that are 1.0 inches DBH and larger. Tree class is basically a check for the straightness and soundness of the sawlog portion on a sawtimber tree or the potential sawlog portion on a poletimber tree or sapling. “Sawlog portion” is defined as the length between the 1-foot stump and the 9.0-inch top diameter of outside bark, DOB, for hardwoods, or the 7.0-inch top DOB for softwoods. For more detailed description, see the regional field guide http://www.nrs.fs.fed.us/fia/data-collection/. Only collected by certain FIA work units (SURVEY.RSCD = 23).

Code	Description
20	Growing-stock – Any live tree of commercial species that is saw-timber size and has at least one merchantable 12-foot sawlog or two merchantable 8-foot sawlogs meeting minimum log-grade requirements. At least one-third of the gross board-foot volume of the sawlog portion must be merchantable material. A merchantable sawlog must be at least 50 percent sound at any point. Any pole timber size tree that has the potential to meet the above specifications.
30	Rough Cull, Salvable, and Salvable-down – Includes any tree of noncommercial species, or any tree that is saw-timber size and has no merchantable sawlog. Over one-half of the volume in the sawlog portion does not meet minimum log-grade specifications due to roughness, excessive sweep or crook, splits, cracks, limbs, or forks. Rough cull pole-size trees do not have the potential to meet the specifications for growing-stock because of forks, limb stoppers, or excessive sweep or crook. A down-dead tree ≥5.0-inch DBH that meets these standards is given a tree/decay code of 30.
31	Short-log Cull – Any live saw-timber-size tree of commercial species that has at least one 8-foot sawlog, but less than a 12-foot sawlog, meeting minimum log-grade specifications. Any live saw-timber-size tree of commercial species that has less than one-third of the volume of the sawlog portion in merchantable logs, but has at least one 8-foot or longer sawlog meeting minimum log-grade specifications. A short sawlog must be 50 percent sound at any point. Pole-size trees never receive a tree class code 31.
40	Rotten Cull – Any live tree of commercial species that is saw-timber size and has no merchantable sawlog. Over one-half of the volume in the sawlog portion does not meet minimum log-grade specifications primarily because of rot, missing sections, or deadwood. Classify any pole-size tree that does not have the potential to meet the specifications for growing-stock because of rot as rotten cull. Assume that all live trees will eventually attain sawlog size at DBH. Predicted death, tree vigor, and plot site index are not considered in determining tree class. A standing-dead tree without an 8-foot or longer section that is at least 50 percent sound has a tree class of 40. On remeasurement of a sapling, if it has died and is still standing it is given a tree class of 40.

107. TREECLCD_RMRS

Tree class code, Rocky Mountain Research Station. A code indicating the general quality of the tree. Only collected by certain FIA work units (SURVEY.RSCD = 22).

Code	Description
1	Sound-live timber species – All live timber trees (species with diameter measured at breast height) that meet minimum merchantability standards. In general, these trees have at least one solid 8-foot section, are reasonably free of form defect on the merchantable bole, and at least 34 percent or more of the volume is merchantable. Excludes rough or rotten cull timber trees.
2	All live woodland species – All live woodland trees (species with diameter measured at root collar). All trees assigned to species groups 23 and 48 belong in this category (see appendix G).

Code	Description
3	Rough-live timber species – All live trees that do not now, or prospectively, have at least one solid 8-foot section, reasonably free of form defect on the merchantable bole, or have 67 percent or more of the merchantable volume cull; and more than half of this cull is due to sound dead wood cubic-foot loss or severe form defect volume loss.
4	Rotten-live timber species – All live trees with 67 percent or more of the merchantable volume cull, and more than half of this cull is due to rotten or missing cubic-foot volume loss.
5	Hard (salvable) dead – dead trees that have less than 67 percent of the volume cull due to rotten or missing cubic-foot volume loss.
6	Soft (nonsalvable) dead – dead trees that have 67 percent or more of the volume cull due to rotten or missing cubic-foot volume loss.

108. STANDING_DEAD_CD

Standing dead code. A code indicating if a tree qualifies as standing dead. To qualify as a standing dead tally tree, the dead tree must be at least 5.0 inches in diameter, have a bole that has an unbroken actual length of at least 4.5 feet, and lean less than 45 degrees from vertical as measured from the base of the tree to 4.5 feet. Populated where PLOT.MANUAL ≥2.0; may be populated using information collected on dead trees in earlier inventories for dead trees.

For western woodland species with multiple stems, a tree is considered down if more than ⅔ of the volume is no longer attached or upright; cut and removed volume is not considered. For western woodland species with single stems to qualify as a standing dead tally tree, dead trees must be at least 5.0 inches in diameter, be at least 1.0 foot in unbroken ACTUAL LENGTH, and lean less than 45 degrees from vertical.

Code	Description
0	No – tree does not qualify as standing dead
1	Yes – tree does qualify as standing dead

109. PREV_STATUS_CD

Previous tree status code. Tree status that was recorded at the previous inventory on all tally trees ≥1.0 inch in diameter.

Code	Description
1	Live tree – live tree at the previous inventory
2	Dead tree – standing dead at the previous inventory

110. PREV_WDLDSTEM

Previous woodland stem count. Woodland tree species stem count that was recorded at the previous inventory.

111. TPA_UNADJ

Trees per acre unadjusted. The number of trees per acre that the sample tree theoretically represents based on the sample design. For fixed radius plots taken with the mapped plot design (PLOT.DESIGNCD = 1), TPA_UNADJ is set to a constant derived from the plot size and equals 6.018046 for trees sampled on subplots, 74.965282 for trees sampled on microplots, and

0.999188 for trees sampled on macroplots. Variable radius plots were often used in earlier inventories, so the value in TPA_UNADJ decreases as the tree diameter increases. Based on the procedures described in Bechtold and Patterson (2005), this attribute can be adjusted using factors stored on the POP_STRATUM table to derive population estimates. Examples of estimating population totals are shown in chapter 4.

112. TPAMORT_UNADJ

Mortality trees per acre unadjusted. The number of mortality trees per acre per year that the sample tree theoretically represents based on the sample design. For fixed radius plots taken with the mapped plot design (PLOT.DESIGNCD =1), TPAMORT_UNADJ is set to a constant derived from the plot size divided by PLOT.REMPER. Variable radius plots were often used in earlier inventories, so the value in TPAMORT_UNADJ decreases as the tree diameter increases. This attribute will be blank (null) if the tree does not contribute to mortality estimates. Based on the procedures described in Bechtold and Patterson (2005), this attribute can be adjusted using factors stored on the POP_STRATUM table to derive population estimates. Examples of estimating population totals are shown in chapter 4.

113. TPAREMV_UNADJ

Removal trees per acre unadjusted. The number of removal trees per acre per year that the sample tree theoretically represents based on the sample design. For fixed radius plots taken with the mapped plot design (PLOT.DESIGNCD =1), TPAREMV_UNADJ is set to a constant derived from the plot size divided by PLOT.REMPER. Variable radius plots were often used in earlier inventories, so the value in TPAREMV_UNADJ decreases as the tree diameter increases. This attribute will be blank (null) if the tree does not contribute to removals estimates. Based on the procedures described in Bechtold and Patterson (2005), this attribute can be adjusted using factors stored on the POP_STRATUM table to derive population estimates. Examples of estimating population totals are shown in chapter 4.

114. TPAGROW_UNADJ

Growth trees per acre unadjusted. The number of growth trees per acre that the sample tree theoretically represents based on the sample design. For fixed radius plots taken with the mapped plot design (PLOT.DESIGNCD = 1), TPAGROW_UNADJ is set to a constant derived from the plot size. Variable radius plots were often used in earlier inventories, so the value in TPAGROW_UNADJ decreases as the tree diameter increases. This attribute will be blank (null) if the tree does not contribute to growth estimates. Based on the procedures described in Bechtold and Patterson (2005), this attribute can be adjusted using factors stored on the POP_STRATUM table to derive population estimates. Examples of estimating population totals are shown in chapter 4.

115. DRYBIO_BOLE

Dry biomass in the merchantable bole. The oven-dry biomass (pounds) in the merchantable bole of timber species [trees where diameter is measured at breast height (DBH)] ≥5 inches in diameter. This is the biomass of sound wood in live and dead trees, including bark, from a 1-foot stump to a minimum 4-inch top diameter of the central stem. This is a per tree value and must be multiplied by TPA_UNADJ to obtain per acre information. This attribute is blank (null) for timber species with DIA <5.0 inches and for woodland species. See DRYBIO_WDLD_SPP for biomass of woodland species and DRYBIO_SAPLING for biomass of timber species with DIA <5 inches. For dead or cut timber trees, this number represents the biomass at the time of death or last measurement. DRYBIO_BOLE is based on VOLCFSND and specific gravity information derived by the Forest Products Lab and others (values stored in the REF_SPECIES table). If VOLCFSND is not available, then either VOLCFGRS * Percent Sound or VOLCFNET * (average ratio of cubic foot sound to cubic foot net volume, calculated as national averages by species group and diameter) is used. The source of specific gravity information for each species can be found by linking the REF_SPECIES table to the REF_CITATION table. Appendix J contains equations used to estimate biomass components in the FIADB.

116. DRYBIO_TOP Dry biomass in the top of the tree. The oven-dry biomass (pounds) in the top and branches (combined) of timber species [trees where diameter is measured at breast height (DBH)] ≥5 inches in diameter. DRYBIO_TOP includes the tip, the portion of the stem above the merchantable bole (i.e., above the 4-inch top diameter), and all branches; excludes foliage. Estimated for live and dead trees. This is a per tree value and must be multiplied by TPA_UNADJ to obtain per acre information. For dead or cut trees, this number represents the biomass at the time of death or last measurement. This attribute is blank (null) for timber species with DIA <5.0 inches and for woodland species. See DRYBIO_WDLD_SPP for biomass of woodland species, and DRYBIO_SAPLING for biomass of timber species with DIA <5.0 inches. Appendix J contains equations used to estimate biomass components in the FIADB.

117. DRYBIO_STUMP

Dry biomass in the tree stump. The oven-dry biomass (pounds) in the stump of timber species [trees where diameter is measured at breast height (DBH)] ≥5 inches in diameter. The stump is that portion of the tree from the ground to the bottom of the merchantable bole (i.e., below 1 foot). This is a per tree value and must be multiplied by TPA_UNADJ to obtain per acre information. Estimated for live and dead trees. For dead or cut trees, this number represents the biomass at the time of death or last measurement. This attribute is blank (null) for timber species with DIA <5.0 inches and for woodland species. See DRYBIO_WDLD_SPP for biomass of woodland species, and DRYBIO_SAPLING for biomass of timber species with

DIA <5.0 inches. Appendix J contains equations used to estimate biomass components in the FIADB.

118. DRYBIO_SAPLING

Dry biomass of saplings. The oven-dry biomass (pounds) of the aboveground portion, excluding foliage, of live trees with a diameter from 1 to 4.9 inches. Calculated for timber species only. The biomass of saplings is based on biomass computed from Jenkins and others (2003), using the observed diameter and an adjustment factor. This is a per tree value and must be multiplied by TPA_UNADJ to obtain per acre information. Appendix J contains equations used to estimate biomass components in the FIADB.

119. DRYBIO_WDLD_SPP

Dry biomass of woodland tree species. The oven-dry biomass (pounds) of the aboveground portion of a live or dead tree, excluding foliage, the tree tip (top of the tree above 1½ inches in diameter), and a portion of the stump from ground to diameter at root collar (DRC). Calculated for woodland species (trees where diameter is measured at DRC) with a diameter ≥1 inch. This is a per tree value and must be multiplied by TPA_UNADJ to obtain per acre information. This attribute is blank (null) for woodland species with DIA <1.0 inch and for all timber species. Appendix J contains equations used to estimate biomass components in the FIADB.

120. DRYBIO_BG Dry biomass of the roots. The oven-dry biomass (pounds) of the belowground portion of a tree, includes coarse roots with a root diameter ≥0.1 inch. This is a modeled estimate, calculated on live trees with a diameter of ≥1 inch and dead trees with a diameter of ≥5 inches, for both timber and woodland. This is a per tree value and must be multiplied by TPA_UNADJ to obtain per acre information. Appendix J contains equations used to estimate biomass components in the FIADB.

121. CARBON_AG Carbon in the aboveground portion of the tree. The carbon (pounds) in the aboveground portion, excluding foliage, of live trees with a diameter ≥1 inch, and dead trees with a diameter ≥5 inches. Calculated for both timber and woodland species. This is a per tree value and must be multiplied by TPA_UNADJ to obtain per acre information. Carbon is assumed to be one-half the value of biomass and is derived by summing the aboveground biomass estimates and multiplying by 0.5 as follows:

CARBON_AG = 0.5 * (DRYBIO_BOLE + DRYBIO_STUMP + DRYBIO_TOP + DRYBIO_SAPLING + DRYBIO_WDLD_SPP)

122. CARBON_BG Carbon in the belowground portion of the tree. The carbon (pounds) of coarse roots >0.1 inch in root diameter. Calculated for live trees with a diameter ≥1 inch, and dead trees with a diameter ≥5 inches, for both timber and woodland species. This is a per tree value and must be multiplied by

TPA_UNADJ to obtain per acre information. Carbon is assumed to be one-half the value of belowground biomass as follows:

CARBON_BG = 0.5 * DRYBIO_BG

123. CYCLE Inventory cycle number. A number assigned to a set of plots, measured over a particular period of time from which a State estimate using all possible plots is obtained. A cycle number >1 does not necessarily mean that information for previous cycles resides in the database. A cycle is relevant for periodic and annual inventories.

124. SUBCYCLE Inventory subcycle number. For an annual inventory that takes n years to measure all plots, subcycle shows in which of the n years of the cycle the data were measured. Subcycle is 0 for a periodic inventory. Subcycle 99 may be used for plots that are not included in the estimation process.

125. BORED_CD_PNWRS

Tree bored code, Pacific Northwest Research Station. Used in conjunction with tree age (BHAGE and TOTAGE). Only collected by certain FIA work units (SURVEY.RSCD = 26).

Code	Description
1	Trees bored or 'whorl counted' at the current inventory
2	Tree age derived from a previous inventory
3	Tree age was extrapolated

126. DAMLOC1_PNWRS

Damage location 1, Pacific Northwest Research Station. The location on the tree where Damage Agent 1 is found. Only collected by certain FIA work units (SURVEY.RSCD = 26).

Code	Location	Definition
0		No damage found.
1	Roots	Above ground up to 12 inches on bole.
2	Bole	Main stem(s) starting at 12 inches above the ground, including forks up to a 4 inch top. (A fork is at least equal to 1/3 diameter of the bole, and occurs at an angle <45 degrees in relation to the bole.) This is not a valid location code for woodland species; use only locations 1, 3, and 4.
3	Branch	All other woody material. Primary branch(s) occur at an angle ≥45° in relation to the bole.
4	Foliage	All leaves, buds, and shoots.

127. DAMLOC2_PNWRS

Damage location 2, Pacific Northwest Research Station. See DAMLOC1_PNWRS. Only collected by certain FIA work units (SURVEY.RSCD = 26).

128. DIACHECK_PNWRS

Diameter check, Pacific Northwest Research Station. A separate estimate of the diameter without the obstruction if the diameter was estimated because of moss/vine/obstruction, etc. Only collected by certain FIA work units (SURVEY.RSCD = 26).

Code	Description
5	Diameter estimated because of moss.
6	Diameter estimated because of vines.
7	Diameter estimated (double nail diameter).

129. DMG_AGENT1_CD_PNWRS

Damage agent 1, Pacific Northwest Research Station. Primary damage agent code in PNW. Up to three damaging agents can be coded in PNW as DMG_AGENT1_CD_PNWRS, DMG_AGENT2_CD_PNWRS, and DMG_AGENT3_CD_PNWRS. A code indicating the tree damaging agent that is considered to be of greatest importance to predict tree growth, survival, and forest composition and structure. Additionally, there are two classes of damaging agents. Class I damage agents are considered more important than class II agents and are thus coded as a primary agent before the class II agents. For more information, see appendix H. Only collected by certain FIA work units (SURVEY.RSCD = 26).

130. DMG_AGENT2_CD_PNWRS

Damage agent 2, Pacific Northwest Research Station. See DAM_AGENT1_CD_PNWRS. Only collected by certain FIA work units (SURVEY.RSCD = 26).

131. DMG_AGENT3_CD_PNWRS

Damage agent 3, Pacific Northwest Research Station. Damage Agent is a 2-digit code with values 01 to 91. Only collected by certain FIA work units (SURVEY.RSCD = 26).

132. MIST_CL_CD_PNWRS

Leafy mistletoe class code, Pacific Northwest Research Station. All juniper species, incense cedars, white fir (CA only) and oak trees are rated for leafy mistletoe infection. This item is used to describe the extent and severity of leafy mistletoe infection (see MIST_CL_CD for dwarf mistletoe information). Only collected by certain FIA work units (SURVEY.RSCD=26).

Code	Description
0	None
7	<50 percent of crown infected
8	≥50 percent of crown infected or any occurrence on the bole

133. SEVERITY1_CD_PNWRS

Damage severity 1, Pacific Northwest Research Station for years 2001-2004. Damage severity depends on the damage agent coded (see appendix H for codes). This is a 2-digit code that indicates either percent of location damaged (01-99), or the appropriate class of damage (values vary from 0-9 depending on the specific Damage Agent). Only collected by certain FIA work units (SURVEY.RSCD = 26).

134. SEVERITY1A_CD_PNWRS

Damage severity 1A, Pacific Northwest Research Station. Damage severity depends on the damage agent coded (see appendix H for codes). This is a 2-digit code indicating either percent of location damaged (01-99), or the appropriate class of damage (values vary from 0-4 depending on the specific Damage Agent). Only collected by certain FIA work units (SURVEY.RSCD = 26).

135. SEVERITY1B_CD_PNWRS

Damage severity 1B, Pacific Northwest Research Station. Damage severity B is only coded when the Damage Agent is white pine blister rust (36). Only collected by certain FIA work units (SURVEY.RSCD = 26).

Code	Description
1	Branch infections located more than 2.0 feet from tree bole.
2	Branch infections located 0.5 to 2.0 feet from tree bole.
3	Branch infection located within 0.5 feet of tree bole OR tree bole infection present.

136. SEVERITY2_CD_PNWRS

Damage severity 2, Pacific Northwest Research Station for years 2001-2004. Damage severity depends on the damage agent coded (see appendix H for codes). This is a 2-digit code indicating either percent of location damaged (01-99), or the appropriate class of damage (values vary from 0-9 depending on the specific Damage Agent). Only collected by certain FIA work units (SURVEY.RSCD = 26).

137. SEVERITY2A_CD_PNWRS

Damage severity 2A, Pacific Northwest Research Station starting in 2005. See SEVERITY1A_CD_PNWRS. Only collected by certain FIA work units (SURVEY.RSCD = 26).

138. SEVERITY2B_CD_PNWRS

Damage severity 2B, Pacific Northwest Research Station starting in 2005. See SEVERITY1B_CD_PNWRS. Only collected by certain FIA work units (SURVEY.RSCD = 26).

139. SEVERITY3_CD_PNWRS

Damage severity 3, Pacific Northwest Research Station for years 2001-2004. Damage severity depends on the damage agent coded (see appendix H for codes). This is a 2-digit code indicating either percent of location damaged (01-99), or the appropriate class of damage (values vary from 0-9 depending on the specific Damage Agent). Only collected by certain FIA work units (SURVEY.RSCD = 26).

140. UNKNOWN_DAMTYP1_PNWRS

Unknown damage type 1, Pacific Northwest Research Station. A code indicating the sign or symptom recorded when UNKNOWN damage code 90 is used. Only collected by certain FIA work units (SURVEY.RSCD = 26).

Code	Description
1	canker/gall
2	open wound
3	resinosis
4	broken
5	damaged or discolored foliage
6	other

141. UNKNOWN_DAMTYP2_PNWRS

Unknown damage type 2, Pacific Northwest Research Station. See UNKNOWN_DAMTYP1_PNWRS. Only collected by certain FIA work units (SURVEY.RSCD = 26).

142. PREV_PNTN_SRS

Previous periodic prism number, tree number, Southern Research Station. In some older Southeast Experiment Station states, the prism point, tree number (PNTN) of the current cycle did not match the previous cycle's prism point, tree number. PREV_PNTN_SRS is used to join the current and the previous prism plot trees.

Seedling Table (Oracle table name is SEEDLING)

	Column name	Descriptive name	Oracle data type
1	CN	Sequence number	VARCHAR2(34)
2	PLT_CN	Plot sequence number	VARCHAR2(34)
3	INVYR	Inventory year	NUMBER(4)
4	STATECD	State code	NUMBER(4)
5	UNITCD	Unit code	NUMBER(2)
6	COUNTYCD	County code	NUMBER(3)
7	PLOT	Phase 2 plot number	NUMBER(5)
8	SUBP	Subplot number	NUMBER(3)
9	CONDID	Condition class number	NUMBER(1)
10	SPCD	Species code	NUMBER
11	SPGRPCD	Species group code	NUMBER(2)
12	STOCKING	Tree stocking	NUMBER(7,4)
13	TREECOUNT	Tree count for seedlings	NUMBER(3)
14	TOTAGE	Total age	NUMBER(3)
15	CREATED_BY	Created by	VARCHAR2(30)
16	CREATED_DATE	Created date	DATE
17	CREATED_IN_INSTANCE	Created in instance	VARCHAR2(6)
18	MODIFIED_BY	Modified by	VARCHAR2(30)
19	MODIFIED_DATE	Modified date	DATE
20	MODIFIED_IN_INSTANCE	Modified in instance	VARCHAR2(6)
21	TREECOUNT_CALC	Tree count used in calculations	NUMBER
22	TPA_UNADJ	Trees per acre unadjusted	NUMBER(11,6)
23	CYCLE	Inventory cycle number	NUMBER(2)
24	SUBCYCLE	Inventory subcycle number	NUMBER(2)

Type of key	Column(s)	Tables to link	Abbreviated notation
Primary	(CN)	N/A	SDL_PK
Unique	(PLT_CN, SUBP, CONDID, SPCD)	N/A	SDL_UK
Natural	(STATECD, INVYR, UNITCD, COUNTYCD, PLOT, SUBP, CONDID, SPCD)	N/A	SDL_NAT_I
Foreign	(PLT_CN)	SEEDLING to PLOT	SDL_PLT_FK

Seedling data collection overview – When PLOT.MANUAL <2.0, the national core procedure was to record the actual seedling count up to six seedlings and then record 6+ if at least six seedlings were present. However, the following regions collected the actual seedling count when PLOT.MANUAL <2.0: Rocky Mountain Research Station (RMRS) and North Central Research Station (NCRS). If PLOT.MANUAL <2.0 and TREECOUNT is blank (null), then a value of 6 in TREECOUNT_CALC represents 6 or more seedlings. In the past, seedlings were often tallied in FIA inventories only to the extent necessary to determine if some minimum number were present,

which means that seedlings were often under-reported. Note: The SEEDLING record may not exist for some periodic inventories.

1. CN — Sequence number. A unique index used to easily identify a seedling.

2. PLT_CN — Plot sequence number. Foreign key linking the seedling record to the plot record.

3. INVYR — Inventory year. The year that best represents when the inventory data were collected. Under the annual inventory system, a group of plots is selected each year for sampling. The selection is based on a panel system. INVYR is the year in which the majority of plots in that group were collected (plots in the group have the same panel and, if applicable, subpanel). Under periodic inventory, a reporting inventory year was selected, usually based on the year in which the majority of the plots were collected or the mid-point of the years over which the inventory spanned. For either annual or periodic inventory, INVYR is not necessarily the same as MEASYEAR.

   Exceptions:
   INVYR = 9999. INVYR is set to 9999 to distinguish Phase 3 plots taken by the western FIA work units that are "off subpanel." This is due to differences in measurement intervals between Phase 3 (measurement interval = 5 years) and Phase 2 (measurement interval = 10 years) plots. Only users interested in performing certain Phase 3 data analyses should access plots with this anomalous value in INVYR.

   INVYR <100. INVYR <100 indicates that population estimates were derived from a pre-NIMS regional processing system and the same plot either has been or may soon be re-processed in NIMS as part of a separate evaluation. The NIMS processed copy of the plot follows the standard INVYR format. This only applies to plots collected in the South (SURVEY.RSCD = 33) with the national design or a similar regional design (PLOT.DESIGNCD = 1 or 220-233) that were collected when the inventory year was 1998 through 2005.

   INVYR = 98 is equivalent to 1998 but processed through regional system
   INVYR = 99 is equivalent to 1999 but processed through regional system
   INVYR = 0 is equivalent to 2000 but processed through regional system
   INVYR = 1 is equivalent to 2001 but processed through regional system
   INVYR = 2 is equivalent to 2002 but processed through regional system
   INVYR = 3 is equivalent to 2003 but processed through regional system
   INVYR = 4 is equivalent to 2004 but processed through regional system
   INVYR = 5 is equivalent to 2005 but processed through regional system

4. STATECD — State code. Bureau of the Census Federal Information Processing Standards (FIPS) two-digit code for each State. Refer to appendix C.

5. UNITCD — Survey unit number. Forest Inventory and Analysis survey unit identification number. Survey units are usually groups of counties within each State. For periodic inventories, Survey units may be made up of lands of particular owners. Refer to appendix C for codes.

6. COUNTYCD — County code. The identification number for a county, parish, watershed, borough, or similar governmental unit in a State. FIPS codes from the Bureau of the Census are used. Refer to appendix C for codes.

7. PLOT — Phase 2 plot number. An identifier for a plot. Along with STATECD, INVYR, UNITCD, COUNTYCD and/or some other combinations of variables, PLOT may be used to uniquely identify a plot.

8. SUBP — Subplot number. The number assigned to the subplot. The national plot design (PLOT.DESIGNCD = 1) has subplot number values of 1 through 4. Other plot designs have various subplot number values. See PLOT.DESIGNCD and appendix B for information about plot designs. For more explanation about SUBP, contact the appropriate FIA work unit (table 6).

9. CONDID — Condition class number. Unique identifying number assigned to each condition on a plot. A condition is initially defined by condition class status. Differences in reserved status, owner group, forest type, stand-size class, regeneration status, and stand density further define condition for forest land. Mapped nonforest conditions are also assigned numbers. At the time of the plot establishment, the condition class at plot center (the center of subplot 1) is usually designated as condition class 1. Other condition classes are assigned numbers sequentially at the time each condition class is delineated. On a plot, each sampled condition class must have a unique number that can change at remeasurement to reflect new conditions on the plot.

10. SPCD — Species code. An FIA species code. Refer to appendix F for codes.

11. SPGRPCD — Species group code. A code assigned to each tree species in order to group them for reporting purposes on presentation tables. Codes and their associated names (see REF_SPECIES_GROUP.NAME) are shown in appendix G. Individual tree species and corresponding species group codes are shown in appendix F.

12. STOCKING — Tree stocking. The stocking value assigned to each count of seedlings, by species. Stocking is a relative term used to describe (in percent) the adequacy of a given stand density in meeting a specific management objective. Species or forest type stocking functions were used to assess the stocking contribution of seedling records. These functions, which were developed using stocking guides, relate the area occupied by an individual tree to the area occupied by a tree of the same size growing in a fully stocked stand of like trees. The stocking of seedling count records is used in the calculation of COND.GSSTKCD and COND.ALSTKCD on the condition record.

13. TREECOUNT Tree count (for seedlings). Indicates the number of seedlings (DIA <1.0 inch) present on the microplot. Conifer seedlings are at least 6 inches tall and hardwood seedlings are at least 12 inches tall. When PLOT.MANUAL <2.0, the national core procedure was to record the actual seedling count up to six seedlings and then record 6+ if at least six seedlings were present. However, the following regions collected the actual seedling count when PLOT.MANUAL <2.0: Rocky Mountain Research Station (RMRS) and North Central Research Station (NCRS). If PLOT.MANUAL <2.0 and TREECOUNT is blank (null), then a value of 6 in TREECOUNT_CALC represents 6 or more seedlings.

14. TOTAGE Total age. The seedling's total age. Total age is collected for a subset of seedling count records, using one representative seedling for the species. The age is obtained by counting the terminal bud scars or the whorls of branches and may be used in the stand age calculation. Only collected by certain FIA work units (SURVEY.RSCD = 22). This attribute may be blank (null) for SURVEY.RSCD = 22 and is always null for the other FIA work units.

15. CREATED_BY Created by. The employee who created the record. This attribute is intentionally left blank in download files.

16. CREATED_DATE

Created date. The date the record was created. Date will be in the form DD-MON-YYYY.

17. CREATED_IN_INSTANCE

Created in instance. The database instance in which the record was created. Each computer system has a unique database instance code and this attribute stores that information to determine on which computer the record was created.

18. MODIFIED_BY

Modified by. The employee who modified the record. This field will be blank (null) if the data have not been modified since initial creation. This attribute is intentionally left blank in download files.

19. MODIFIED_DATE

Modified date. The date the record was last modified. This field will be blank (null) if the data have not been modified since initial creation. Date will be in the form DD-MON-YYYY.

20. MODIFIED_IN_INSTANCE

Modified in instance. The database instance in which the record was modified. This field will be blank (null) if the data have not been modified since initial creation.

21. TREECOUNT_CALC

Tree count used in calculations. This attribute is set either to COUNTCD, which was dropped in FIADB version 2.1, or TREECOUNT. When PLOT.MANUAL <2.0, the national core procedure was to record the actual seedling count up to six seedlings and then record 6+ if at least six seedlings were present. However, the following regions collected the actual seedling count when PLOT.MANUAL <2.0: Rocky Mountain Research Station (RMRS) and North Central Research Station (NCRS). If PLOT.MANUAL <2.0 and TREECOUNT is blank (null), then a value of 6 in TREECOUNT_CALC represents 6 or more seedlings.

22. TPA_UNADJ Trees per acre unadjusted. The number of seedlings per acre that the seedling count theoretically represents based on the sample design. For fixed radius plots taken with the mapped plot design (PLOT.DESIGNCD =1), TPA_UNADJ equals 74.965282 times the number of seedlings counted. For plots taken with other sample designs, this attribute may be blank (null). Based on the procedures described in Bechtold and Patterson (2005), this attribute can be adjusted using factors stored on the POP_STRATUM table to derive population estimates. Examples of estimating population totals are shown in chapter 4.

23. CYCLE Inventory cycle number. A number assigned to a set of plots, measured over a particular period of time from which a State estimate using all possible plots is obtained. A cycle number >1 does not necessarily mean that information for previous cycles resides in the database. A cycle is relevant for periodic and annual inventories.

24. SUBCYCLE Inventory subcycle number. For an annual inventory that takes n years to measure all plots, subcycle shows in which of the n years of the cycle the data were measured. Subcycle is 0 for a periodic inventory. Subcycle 99 may be used for plots that are not included in the estimation process.

Site Tree Table (Oracle table name is SITETREE)

	Column name	Descriptive name	Oracle data type
1	CN	Sequence number	VARCHAR2(34)
2	PLT_CN	Plot sequence number	VARCHAR2(34)
3	PREV_SIT_CN	Previous site tree sequence number	VARCHAR2(34)
4	INVYR	Inventory year	NUMBER(4)
5	STATECD	State code	NUMBER(4)
6	UNITCD	Survey unit code	NUMBER(2)
7	COUNTYCD	County code	NUMBER(3)
8	PLOT	Phase 2 plot number	NUMBER(5)
9	CONDID	Condition class number	NUMBER(1)
10	TREE	Tree number	NUMBER(9)
11	SPCD	Species code	NUMBER
12	DIA	Diameter	NUMBER(5,2)
13	HT	Total height	NUMBER(3)
14	AGEDIA	Tree age at diameter	NUMBER(3)
15	SPGRPCD	Species group code	NUMBER(2)
16	SITREE	Site index for the tree	NUMBER(3)
17	SIBASE	Site index base age	NUMBER(3)
18	SUBP	Subplot number	NUMBER(3)
19	AZIMUTH	Azimuth	NUMBER(3)
20	DIST	Horizontal distance	NUMBER(4,1)
21	METHOD	Site tree method code	NUMBER(2)
22	SITREE_EST	Estimated site index for the tree	NUMBER(3)
23	VALIDCD	Validity code	NUMBER(1)
24	CONDLIST	Condition class list	NUMBER(4)
25	CREATED_BY	Created by	VARCHAR2(30)
26	CREATED_DATE	Created date	DATE
27	CREATED_IN_INSTANCE	Created in instance	VARCHAR2(6)
28	MODIFIED_BY	Modified by	VARCHAR2(30)
29	MODIFIED_DATE	Modified date	DATE
30	MODIFIED_IN_INSTANCE	Modified in instance	VARCHAR2(6)
31	CYCLE	Inventory cycle number	NUMBER(2)
32	SUBCYCLE	Inventory subcycle number	NUMBER(2)

Type of key	Column(s) order	Tables to link	Abbreviated notation
Primary	(CN)	N/A	SIT_PK
Unique	(PLT_CN, CONDID, TREE)	N/A	SIT_UK
Natural	(STATECD, INVYR, UNITCD, COUNTYCD, PLOT, CONDID, TREE)	N/A	SIT_NAT_I

Type of key	Column(s) order	Tables to link	Abbreviated notation
Foreign	(PLT_CN, CONDID)	SITETREE to COND	SIT_CND_FK
	(PLT_CN)	SITETREE to PLOT	SIT_PLT_FK

Note: The SITETREE record may not exist for some periodic inventory data.

1. CN Sequence number. A unique sequence number used to identify a site tree record.

2. PLT_CN Plot sequence number. Foreign key linking the site tree record to the plot record.

3. PREV_SIT_CN Previous site tree sequence number. Foreign key linking the site tree to the previous inventory's site tree record for this tree. Only populated for site trees from previous annual inventories.

4. INVYR Inventory year. The year that best represents when the inventory data were collected. Under the annual inventory system, a group of plots is selected each year for sampling. The selection is based on a panel system. INVYR is the year in which the majority of plots in that group were collected (plots in the group have the same panel and, if applicable, subpanel). Under periodic inventory, a reporting inventory year was selected, usually based on the year in which the majority of the plots were collected or the mid-point of the years over which the inventory spanned. For either annual or periodic inventory, INVYR is not necessarily the same as MEASYEAR.

   Exceptions:
   INVYR = 9999. INVYR is set to 9999 to distinguish Phase 3 plots taken by the western FIA work units that are "off subpanel." This is due to differences in measurement intervals between Phase 3 (measurement interval = 5 years) and Phase 2 (measurement interval = 10 years) plots. Only users interested in performing certain Phase 3 data analyses should access plots with this anomalous value in INVYR.

   INVYR <100. INVYR <100 indicates that population estimates were derived from a pre-NIMS regional processing system and the same plot either has been or may soon be re-processed in NIMS as part of a separate evaluation. The NIMS processed copy of the plot follows the standard INVYR format. This only applies to plots collected in the South (SURVEY.RSCD = 33) with the national design or a similar regional design (PLOT.DESIGNCD = 1 or 220-233) that were collected when the inventory year was 1998 through 2005.

   INVYR = 98 is equivalent to 1998 but processed through regional system
   INVYR = 99 is equivalent to 1999 but processed through regional system
   INVYR = 0 is equivalent to 2000 but processed through regional system
   INVYR = 1 is equivalent to 2001 but processed through regional system

INVYR = 2 is equivalent to 2002 but processed through regional system
INVYR = 3 is equivalent to 2003 but processed through regional system
INVYR = 4 is equivalent to 2004 but processed through regional system
INVYR = 5 is equivalent to 2005 but processed through regional system

5. STATECD — State code. Bureau of the Census Federal Information Processing Standards (FIPS) two-digit code for each State. Refer to appendix C.

6. UNITCD — Survey unit code. Forest Inventory and Analysis survey unit identification number. Survey units are usually groups of counties within each State. For periodic inventories, survey units may be made up of lands of particular owners. Refer to appendix C for codes.

7. COUNTYCD — County code. The identification number for a county, parish, watershed, borough, or similar governmental unit in a State. FIPS codes from the Bureau of the Census are used. Refer to appendix C for codes.

8. PLOT — Phase 2 plot number. An identifier for a plot. Along with STATECD, INVYR, UNITCD, COUNTYCD and/or some other combinations of variables, PLOT may be used to uniquely identify a plot.

9. CONDID — Condition class number. Unique identifying number assigned to each condition on a plot. A condition is initially defined by condition class status. Differences in reserved status, owner group, forest type, stand-size class, regeneration status, and stand density further define condition for forest land. Mapped nonforest conditions are also assigned numbers. At the time of the plot establishment, the condition class at plot center (the center of subplot 1) is usually designated as condition class 1. Other condition classes are assigned numbers sequentially at the time each condition class is delineated. On a plot, each sampled condition class must have a unique number that can change at remeasurement to reflect new conditions on the plot.

10. TREE — Tree number. A number used to uniquely identify a site tree on a condition.

11. SPCD — Species code. A standard tree species code. Refer to appendix F for codes.

12. DIA — Diameter. The current diameter (in inches) of the tree at the point of diameter measurement (DBH/DRC).

13. HT — Total height. The total length (height) of a sample tree (in feet) from the ground to the top of the main stem.

14. AGEDIA — Tree age at diameter. Age (in years) of tree at the point of diameter measurement (DBH/DRC). Age is determined by an increment sample.

15. SPGRPCD — Species group code. A code assigned to each tree species in order to group them for reporting purposes on presentation tables. Codes and their associated names (see REF_SPECIES_GROUP.NAME) are shown in appendix G. Individual tree species and corresponding species group codes are shown in appendix F.

16. SITREE — Site index for the tree. Site index is calculated for dominant and co-dominant trees using one of several methods (see METHOD). It is expressed as height in feet that the tree is expected to attain at a base- or reference age (see SIBASE). Most commonly, site index is calculated using a family of curves that show site index as a function of total length and either breast-height age or total age. The height-intercept (or growth-intercept) method is commonly used for young trees or species that produce conspicuous annual branch whorls; using this method, site index is calculated with the height growth attained for a short period (usually 3 to 5 years) after the tree has reached breast height. Neither age nor total length determination are necessary when using the height-intercept method, so one or more of those variables may be null for a site tree on which the height-intercept method was used.

17. SIBASE — Site index base age. The base age (sometimes called reference age), in years, of the site index curves used to derive site index. Base age is specific to a given family of site index curves, and is usually set close to the common rotation age or the age of culmination of mean annual increment for a species. The most commonly used base ages are 25, 50, 80, and 100 years. It is possible for a given species to have different sets of site index curves in different geographic regions, and each set of curves may use a different base age.

18. SUBP — Subplot number. (*Core optional.*) The number assigned to the subplot. The national plot design (PLOT.DESIGNCD = 1) has subplot number values of 1 through 4. Other plot designs have various subplot number values. See PLOT.DESIGNCD and appendix B for information about plot designs. For more explanation about SUBP, contact the appropriate FIA work unit (table 6).

19. AZIMUTH — Azimuth. (*Core optional.*) The direction, to the nearest degree, from subplot center to the center of the base of the tree (geographic center for multi-stemmed woodland species). Due north is represented by 360 degrees.

20. DIST — Horizontal distance. (*Core optional.*) The horizontal distance in feet from subplot center (microplot center for saplings) to the pith at the base of the tree (geographic center for multi-stemmed woodland species).

21. METHOD — Site tree method code. The method for determining the site index.

Code	Description
1	Tree measurements (length, age, etc.) collected during this inventory.
2	Tree measurements (length, age, etc.) collected during a previous inventory.
3	Site index estimated either in the field or office.
4	Site index determined by the height intercept method during this inventory.

22. SITREE_EST — Estimated site index for the tree. The estimated site index or the site index determined by the height intercept method.

23. VALIDCD — Validity code. A code indicating if this site tree provided a valid result from the site index computation. Some trees collected by the field crew yield a

negative value from the equation due to their age, height or diameter being outside the range of values for which the equation was developed. Computational results for trees that fail are not used to estimate the site index or site productivity class for the condition. If the site calculation for this tree was successful, this attribute is set to 1.

Code	Description
0	Tree failed in site index calculations.
1	Tree was successful in site index calculations.

24. CONDLIST Condition class list. A list of numbers indicating all of the condition classes for which the site index data for this tree can be used.

25. CREATED_BY Created by. The employee who created the record. This attribute is intentionally left blank in download files.

26. CREATED_DATE

Created date. The date the record was created. Date will be in the form DD-MON-YYYY.

27. CREATED_IN_INSTANCE

Created in instance. The database instance in which the record was created. Each computer system has a unique database instance code and this attribute stores that information to determine on which computer the record was created.

28. MODIFIED_BY

Modified by. The employee who modified the record. This field will be blank (null) if the data have not been modified since initial creation. This attribute is intentionally left blank in download files.

29. MODIFIED_DATE

Modified date. The date the record was last modified. This field will be blank (null) if the data have not been modified since initial creation. Date will be in the form DD-MON-YYYY.

30. MODIFIED_IN_INSTANCE

Modified in instance. The database instance in which the record was modified. This field will be blank (null) if the data have not been modified since initial creation.

31. CYCLE Inventory cycle number. A number assigned to a set of plots, measured over a particular period of time from which a State estimate using all possible plots is obtained. A cycle number >1 does not necessarily mean that

information for previous cycles resides in the database. A cycle is relevant for periodic and annual inventories.

32. SUBCYCLE Inventory subcycle number. For an annual inventory that takes n years to measure all plots, subcycle shows in which of the n years of the cycle the data were measured. Subcycle is 0 for a periodic inventory. Subcycle 99 may be used for plots that are not included in the estimation process.

Boundary Table (Oracle table name is BOUNDARY)

	Column name	Descriptive name	Oracle data type
1	CN	Sequence number	VARCHAR2(34)
2	PLT_CN	Plot sequence number	VARCHAR2(34)
3	INVYR	Inventory year	NUMBER(4)
4	STATECD	State code	NUMBER(4)
5	UNITCD	Survey unit code	NUMBER(2)
6	COUNTYCD	County code	NUMBER(3)
7	PLOT	Phase 2 plot number	NUMBER(5)
8	SUBP	Subplot number	NUMBER(3)
9	SUBPTYP	Plot type code	NUMBER(1)
10	BNDCHG	Boundary change code	NUMBER(1)
11	CONTRAST	Contrasting condition	NUMBER(1)
12	AZMLEFT	Left azimuth	NUMBER(3)
13	AZMCORN	Corner azimuth	NUMBER(3)
14	DISTCORN	Corner distance	NUMBER(3)
15	AZMRIGHT	Right azimuth	NUMBER(3)
16	CYCLE	Inventory cycle number	NUMBER(2)
17	SUBCYCLE	Inventory subcycle number	NUMBER(2)
18	CREATED_BY	Created by	VARCHAR2(30)
19	CREATED_DATE	Created date	DATE
20	CREATED_IN_INSTANCE	Created in instance	VARCHAR2(6)
21	MODIFIED_BY	Modified by	VARCHAR2(30)
22	MODIFIED_DATE	Modified date	DATE
23	MODIFIED_IN_INSTANCE	Modified in instance	VARCHAR2(6)

Type of key	Column(s) order	Tables to link	Abbreviated notation
Primary	(CN)	N/A	BND_PK
Unique	(PLT_CN, SUBP, SUBPTYP, AZMLEFT, AZMRIGHT)	N/A	BND_UK
Natural	(STATECD, INVYR, UNITCD, COUNTYCD, PLOT, SUBP, SUBPTYP, AZMLEFT, AZMRIGHT)	N/A	BND_NAT_I
Foreign	(PLT_CN)	BOUNDARY to PLOT	BND_PLT_FK

Note: The BOUNDARY record may not exist for some periodic inventory data.

1. CN Sequence number. A unique sequence number used to identify a boundary record.

2. PLT_CN — Plot sequence number. Foreign key linking the boundary record to the plot record.

3. INVYR — Inventory year. The year that best represents when the inventory data were collected. Under the annual inventory system, a group of plots is selected each year for sampling. The selection is based on a panel system. INVYR is the year in which the majority of plots in that group were collected (plots in the group have the same panel and, if applicable, subpanel). Under periodic inventory, a reporting inventory year was selected, usually based on the year in which the majority of the plots were collected or the mid-point of the years over which the inventory spanned. For either annual or periodic inventory, INVYR is not necessarily the same as MEASYEAR.

   Exceptions:
   INVYR = 9999. INVYR is set to 9999 to distinguish Phase 3 plots taken by the western FIA work units that are "off subpanel." This is due to differences in measurement intervals between Phase 3 (measurement interval = 5 years) and Phase 2 (measurement interval = 10 years) plots. Only users interested in performing certain Phase 3 data analyses should access plots with this anomalous value in INVYR.

   INVYR <100. INVYR <100 indicates that population estimates were derived from a pre-NIMS regional processing system and the same plot either has been or may soon be re-processed in NIMS as part of a separate evaluation. The NIMS processed copy of the plot follows the standard INVYR format. This only applies to plots collected in the South (SURVEY.RSCD = 33) with the national design or a similar regional design (PLOT.DESIGNCD = 1 or 220-233) that were collected when the inventory year was 1998 through 2005.

   INVYR = 98 is equivalent to 1998 but processed through regional system
   INVYR = 99 is equivalent to 1999 but processed through regional system
   INVYR = 0 is equivalent to 2000 but processed through regional system
   INVYR = 1 is equivalent to 2001 but processed through regional system
   INVYR = 2 is equivalent to 2002 but processed through regional system
   INVYR = 3 is equivalent to 2003 but processed through regional system
   INVYR = 4 is equivalent to 2004 but processed through regional system
   INVYR = 5 is equivalent to 2005 but processed through regional system

4. STATECD — State code. Bureau of the Census Federal Information Processing Standards (FIPS) two-digit code for each State. Refer to appendix C.

5. UNITCD — Survey unit code. Forest Inventory and Analysis survey unit identification number. Survey units are usually groups of counties within each State. For periodic inventories, survey units may be made up of lands of particular owners. Refer to appendix C for codes.

6. COUNTYCD — County code. The identification number for a county, parish, watershed, borough, or similar governmental unit in a State. FIPS codes from the Bureau of the Census are used. Refer to appendix C for codes.

7. PLOT — Phase 2 plot number. An identifier for a plot. Along with STATECD, UNITCD, INVYR, COUNTYCD and/or some other combinations of variables, PLOT may be used to uniquely identify a plot.

8. SUBP — Subplot number. The number assigned to the subplot. The national plot design (PLOT.DESIGNCD = 1) has subplot number values of 1 through 4. Other plot designs have various subplot number values. See PLOT.DESIGNCD and appendix B for information about plot designs. For more explanation about SUBP, contact the appropriate FIA work unit.

9. SUBPTYP — Plot type code. Specifies whether the boundary data are for a subplot, microplot, or macroplot.

**Code**	**Description**
1	Subplot boundary
2	Microplot boundary
3	Macroplot boundary

10. BNDCHG — Boundary change code. A code indicating the relationship between previously recorded and current boundary information. Set to blank (null) for new plots (PLOT.KINDCD = 1 or 3).

**Code**	**Description**
0	No change – boundary is the same as indicated on plot map by previous crew.
1	New boundary, or boundary data have been changed to reflect an actual on-the-ground physical change resulting in a difference from the boundaries recorded.
2	Boundary has been changed to correct an error from a previous crew.
3	Boundary has been changed to reflect a change in variable definition.

11. CONTRAST — Contrasting condition. The condition class number of the condition class that contrasts with the condition class located at the subplot center (for boundaries on the subplot or macroplot) or at the microplot center (for boundaries on the microplot), e.g., the condition class present on the other side of the boundary.

12. AZMLEFT — Left azimuth. The azimuth, to the nearest degree, from the subplot, microplot, or macroplot plot center to the farthest left point (facing the contrasting condition class) where the boundary intersects the subplot, microplot, or macroplot plot circumference.

13. AZMCORN — Corner azimuth. The azimuth, to the nearest degree, from the subplot, microplot, or macroplot plot center to a corner or curve in a boundary. If a boundary is best described by a straight line between the two circumference points, then 000 is recorded for AZMCORN.

14. DISTCORN — Corner distance. The horizontal distance, to the nearest 1 foot, from the subplot, microplot, or macroplot plot center to the boundary corner point.

Blank (null) when AZMCORN = 000; populated when BOUNDARY.AZMCORN >000.

15. AZMRIGHT — Right azimuth. The azimuth, to the nearest degree, from subplot, microplot, or macroplot plot center to the farthest right point (facing the contrasting condition) where the boundary intersects the subplot, microplot, or macroplot plot circumference.

16. CYCLE — Inventory cycle number. A number assigned to a set of plots, measured over a particular period of time from which a State estimate using all possible plots is obtained. A cycle number >1 does not necessarily mean that information for previous cycles resides in the database. A cycle is relevant for periodic and annual inventories.

17. SUBCYCLE — Inventory subcycle number. For an annual inventory that takes n years to measure all plots, subcycle shows in which of the n years of the cycle the data were measured. Subcycle is 0 for a periodic inventory. Subcycle 99 may be used for plots that are not included in the estimation process.

18. CREATED_BY — Created by. The employee who created the record. This attribute is intentionally left blank in download files.

19. CREATED_DATE

Created date. The date the record was created. Date will be in the form DD-MON-YYYY.

20. CREATED_IN_INSTANCE

Created in instance. The database instance in which the record was created. Each computer system has a unique database instance code and this attribute stores that information to determine on which computer the record was created.

21. MODIFIED_BY

Modified by. The employee who modified the record. This field will be blank (null) if the data have not been modified since initial creation. This attribute is intentionally left blank in download files.

22. MODIFIED_DATE

Modified date. The date the record was last modified. This field will be blank (null) if the data have not been modified since initial creation. Date will be in the form DD-MON-YYYY.

23. MODIFIED_IN_INSTANCE

Modified in instance. The database instance in which the record was modified. This field will be blank (null) if the data have not been modified since initial creation.

Subplot Condition Change Matrix (Oracle table name is SUBP_COND_CHNG_MTRX)

	Column name	Descriptive name	Oracle data type
1	CN	Sequence number	VARCHAR2(34)
2	STATECD	State code	NUMBER(4)
3	SUBP	Subplot number	NUMBER(1)
4	SUBPTYP	Subplot type	NUMBER(1)
5	PLT_CN	Plot sequence number	VARCHAR2(34)
6	CONDID	Condition class number	NUMBER(1)
7	PREV_PLT_CN	Previous plot sequence number	VARCHAR2(34)
8	PREVCOND	Previous condition class number	NUMBER(1)
9	SUBPTYP_PROP_CHNG	Percent change of subplot condition between previous to current inventory	NUMBER(5,4)
10	CREATED_BY	Created by	VARCHAR2(30)
11	CREATED_DATE	Created date	DATE
12	CREATED_IN_INSTANCE	Created in instance	VARCHAR2(6)
13	MODIFIED_BY	Modified by	VARCHAR2(30)
14	MODIFIED_DATE	Modified date	DATE
15	MODIFIED_IN_INSTANCE	Modified in instance	VARCHAR2(6)

Type of key	Column(s) order	Tables to link	Abbreviated notation
Primary	(CN)	N/A	CMX_PK
Unique	(PLT_CN, PREV_PLT_CN, SUBP, SUBPTYP, CONDID, PREVCOND)	N/A	CMX_UK
Foreign	(PREV_PLT_CN)	SUBP_COND_CHNG_MTRX to PLOT	CMX_PLT_FK
	(PLT_CN)	SUBP_COND_CHNG_MTRX to PLOT	CMX_PLT_FK2

This table contains information about the mix of current and previous conditions that occupy the same area on the subplot. Figure 5 provides an illustration of how the information in this table is derived using data from two points in time that is stored in the BOUNDARY and COND tables.

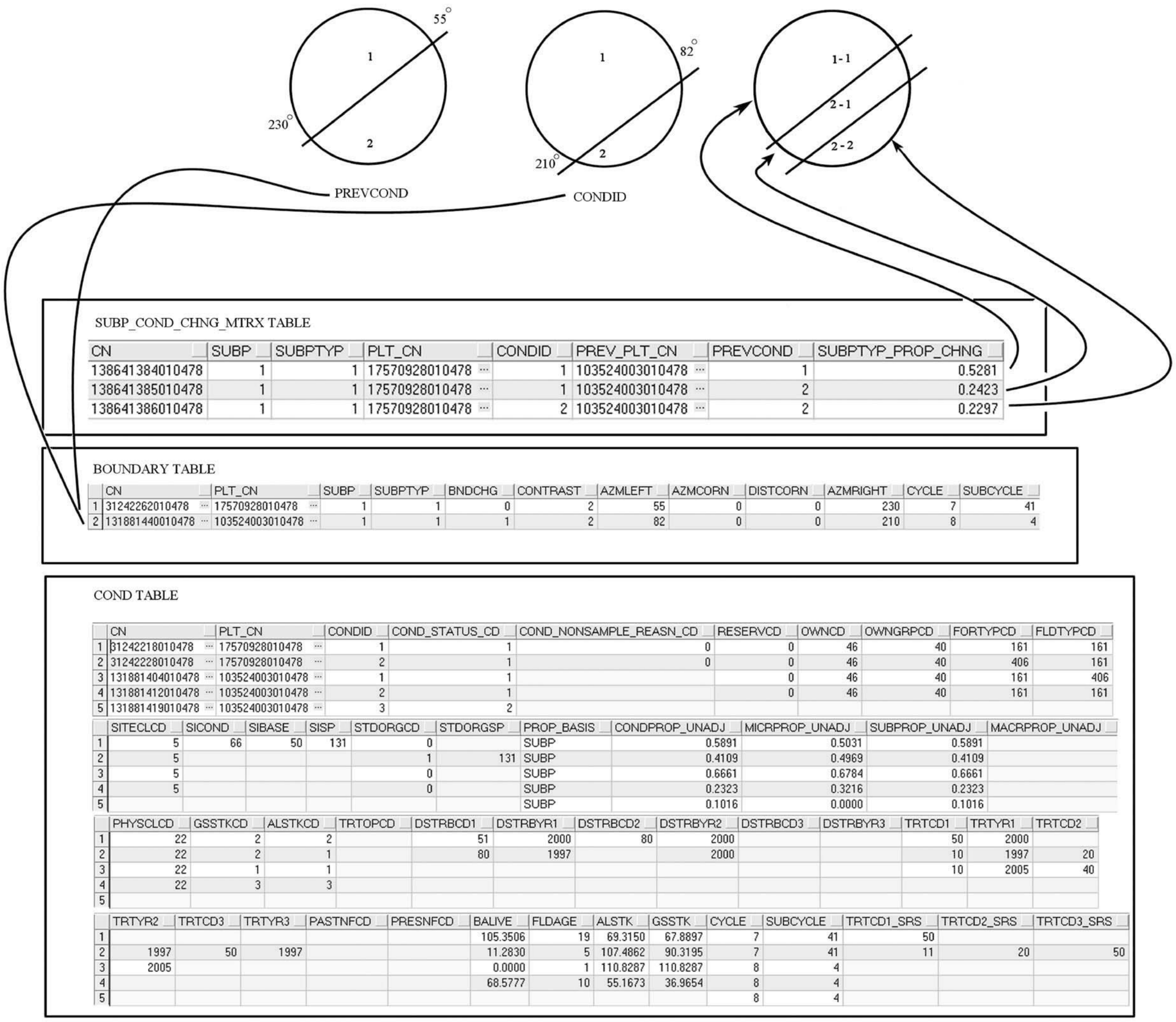

SUBP_COND_CHNG_MTRX TABLE

CN	SUBP	SUBPTYP	PLT_CN	CONDID	PREV_PLT_CN	PREVCOND	SUBPTYP_PROP_CHNG
138641384010478	1	1	17570928010478 ···	1	103524003010478 ···	1	0.5281
138641385010478	1	1	17570928010478 ···	1	103524003010478 ···	2	0.2423
138641386010478	1	1	17570928010478 ···	2	103524003010478 ···	2	0.2297

BOUNDARY TABLE

	CN	PLT_CN	SUBP	SUBPTYP	BNDCHG	CONTRAST	AZMLEFT	AZMCORN	DISTCORN	AZMRIGHT	CYCLE	SUBCYCLE
1	31242262010478 ···	17570928010478 ···	1	1	0	2	55	0	0	230	7	41
2	131881440010478 ···	103524003010478 ···	1	1	1	2	82	0	0	210	8	4

COND TABLE

	CN	PLT_CN	CONDID	COND_STATUS_CD	COND_NONSAMPLE_REASN_CD	RESERVCD	OWNCD	OWNGRPCD	FORTYPCD	FLDTYPCD
1	31242218010478 ···	17570928010478 ···	1	1	0	0	46	40	161	161
2	31242228010478 ···	17570928010478 ···	2	1	0	0	46	40	406	161
3	131881404010478 ···	103524003010478 ···	1	1		0	46	40	161	406
4	131881412010478 ···	103524003010478 ···	2	1		0	46	40	161	161
5	131881419010478 ···	103524003010478 ···	3	2						

	SITECLCD	SICOND	SIBASE	SISP	STDORGCD	STDORGSP	PROP_BASIS	CONDPROP_UNADJ	MICRPROP_UNADJ	SUBPROP_UNADJ	MACRPROP_UNADJ
1	5	66	50	131	0		SUBP	0.5891	0.5031	0.5891	
2	5				1	131	SUBP	0.4109	0.4969	0.4109	
3	5				0		SUBP	0.6661	0.6784	0.6661	
4	5				0		SUBP	0.2323	0.3216	0.2323	
5							SUBP	0.1016	0.0000	0.1016	

	PHYSCLCD	GSSTKCD	ALSTKCD	TRTOPCD	DSTRBCD1	DSTRBYR1	DSTRBCD2	DSTRBYR2	DSTRBCD3	DSTRBYR3	TRTCD1	TRTYR1	TRTCD2
1	22	2	2		51	2000	80	2000			50	2000	
2	22	2	1		80	1997		2000			10	1997	20
3	22	1	1								10	2005	40
4	22	3	3										
5													

	TRTYR2	TRTCD3	TRTYR3	PASTNFCD	PRESNFCD	BALIVE	FLDAGE	ALSTK	GSSTK	CYCLE	SUBCYCLE	TRTCD1_SRS	TRTCD2_SRS	TRTCD3_SRS
1						105.3506	19	69.3150	67.8897	7	41	50		
2	1997	50	1997			11.2830	5	107.4862	90.3195	7	41	11	20	50
3	2005					0.0000	1	110.8287	110.8287	8	4			
4						68.5777	10	55.1673	36.9654	8	4			
5										8	4			

Figure 5. Illustration of the SUBP_COND_CHNG_MTRX table function

1. CN — Sequence number. A unique sequence number used to identify a change matrix table record.

2. STATECD — States code. Bureau of the Census Federal Information Processing Standards (FIPS) two-digit code for each State. Refer to appendix C.

3. SUBP — Subplot number. The number assigned to the subplot. The national plot design (PLOT.DESIGNCD = 1) has subplot number values of 1 through 4. Other plot designs have various subplot number values.

4. SUBPTYP Plot type code. Specifies whether the record is for a subplot, microplot, or macroplot.

Code	Description
1	Subplot
2	Microplot
3	Macroplot

5. PLT_CN Plot sequence number. The foreign key linking the SUBP_COND_CHNG_MTRX record to the PLOT record for the current inventory.

6. CONDID Condition class number. Unique identifying number assigned to each condition on a plot.

7. PREV_PLT_CN

Previous plot sequence number. The foreign key linking the SUBP_COND_CHNG_MTRX record to the PLOT record from the previous inventory.

8. PREVCOND Previous condition class number. Identifies the condition class number from the previous inventory.

9. SUBPTYP_PROP_CHNG

Subplot type proportion change. The unadjusted proportion of the subplot that is in the same geographic area condition for both the previous and current inventory. The sum of all subplot type change proportions for an individual plot equals 4 for each plot type (microplot, subplot, and/or macroplot). Divide the result by 4 to obtain change at the plot level.

10. CREATED_BY Created by. The employee who created the record. This attribute is intentionally left blank in download files.

11. CREATED_DATE

Created date. The date the record was created. Date will be in the form DD-MON-YYYY.

12. CREATED_IN_INSTANCE

Created in instance. The database instance in which the record was created. Each computer system has a unique database instance code and this attribute stores that information to determine on which computer the record was created.

13. MODIFIED_BY

Modified by. The employee who modified the record. This field will be blank (null) if the data have not been modified since initial creation. This attribute is intentionally left blank in download files.

14. MODIFIED_DATE

Modified date. The date the record was last modified. This field will be blank (null) if the data have not been modified since initial creation. Date will be in the form DD-MON-YYYY.

15. MODIFIED_IN_INSTANCE

Modified in instance. The database instance in which the record was modified. This field will be blank (null) if the data have not been modified since initial creation.

Tree Regional Biomass Table (Oracle table name is TREE_REGIONAL_BIOMASS)

	Column name	Descriptive name	Oracle data type
1	TRE_CN	Tree sequence number	VARCHAR2(34)
2	STATECD	State code	NUMBER(4)
3	REGIONAL_DRYBIOT	Regional total tree biomass oven-dry weight	NUMBER(13,6)
4	REGIONAL_DRYBIOM	Regional merchantable stem biomass oven-dry weight	NUMBER(13,6)
5	CREATED_BY	Created by	VARCHAR2(30)
6	CREATED_DATE	Created date	DATE
7	CREATED_IN_INSTANCE	Created in instance	VARCHAR2(6)
8	MODIFIED_BY	Modified by	VARCHAR2(30)
9	MODIFIED_DATE	Modified date	DATE
10	MODIFIED_IN_INSTANCE	Modified in instance	VARCHAR2(6)

Type of key	Column(s) order	Tables to link	Abbreviated notation
Primary	(TRE_CN)	N/A	TRB_PK
Foreign	(TRE_CN)	TREE_REGIONAL_BIOMASS to TREE	TRB_TRE_FK

This table provides biomass estimates of live and dead trees 1 inch in diameter and larger using equations and methods that vary by FIA work unit. Both REGIONAL_DRYBIOT and REGIONAL_DRYBIOM preserve the original data and computation procedures used by FIA work units to calculate DRYBIOT and DRYBIOM in previous versions of FIADB. Users should be aware that for some FIA work units, these biomass estimates may not include bark. Biomass estimates in this table will differ from biomass estimates found on the TREE table records because components such as bark, stump, and top (with branches) are now being stored on the TREE table are derived by applying ratios to stem biomass. The TREE table will be the source of biomass data used in official reporting. However, the TREE_REGIONAL_BIOMASS table contains valuable information for generating biomass estimates that match earlier published reports.

1. TRE_CN — Tree sequence number. Foreign key linking the tree regional biomass record to the tree record.

2. STATECD — States code. Bureau of the Census Federal Information Processing Standards (FIPS) two-digit code for each State. Refer to appendix C.

3. REGIONAL_DRYBIOT

   Regional dry total biomass (pounds). The total aboveground biomass of a sample tree 1.0 inch diameter or larger, including all tops and limbs (but excluding foliage). This is a per tree value and must be multiplied by TPA_UNADJ to obtain per acre information. Calculated in oven-dry pounds per tree. This field should have an entry if DIA is 1.0 inch or larger, regardless of STATUSCD or TREECLCD; zero otherwise. For dead or cut trees, this number represents the biomass at the time of death or last

measurement. Because total biomass has been calculated differently among FIA work units, contact the appropriate FIA work units (see table 6) for information on how biomass was estimated and whether bark was included.

4. REGIONAL_DRYBIOM

Regional dry merchantable stem biomass (pounds). The total gross biomass (including bark) of a tree 5.0 inches DBH or larger from a 1-foot stump to a minimum 4-inch top diameter of the central stem. This is a per tree value and must be multiplied by TPA_UNADJ to obtain per acre information. Calculated in oven-dry pounds per tree. This field should have an entry if DIA is 5.0 inches or larger, regardless of STATUSCD or TREECLCD; zero otherwise. For dead or cut trees, this number represents the biomass at the time of death or last measurement. Because total biomass has been calculated differently among FIA work units, contact the appropriate FIA work unit (see table 6) for information on how biomass was estimated and whether bark was actually included.

5. CREATED_BY Created by. The employee who created the record. This attribute is intentionally left blank in download files.

6. CREATED_DATE

Created date. The date the record was created. Date will be in the form DD-MON-YYYY.

7. CREATED_IN_INSTANCE

Created in instance. The database instance in which the record was created. Each computer system has a unique database instance code and this attribute stores that information to determine on which computer the record was created.

8. MODIFIED_BY

Modified by. The employee who modified the record. This field will be blank (null) if the data have not been modified since initial creation. This attribute is intentionally left blank in download files.

9. MODIFIED_DATE

Modified date. The date the record was last modified. This field will be blank (null) if the data have not been modified since initial creation. Date will be in the form DD-MON-YYYY.

10. MODIFIED_IN_INSTANCE

Modified in instance. The database instance in which the record was modified. This field will be blank (null) if the data have not been modified since initial creation.

Population Estimation Unit Table (Oracle table name is POP_ESTN_UNIT)

	Column name	Descriptive name	Oracle data type
1	CN	Sequence number	VARCHAR2(34)
2	EVAL_CN	Evaluation sequence number	VARCHAR2(34)
3	RSCD	Region or station code	NUMBER(2)
4	EVALID	Evaluation identifier	NUMBER(6)
5	ESTN_UNIT	Estimation unit	NUMBER(6)
6	ESTN_UNIT_DESCR	Estimation unit description	VARCHAR2(255)
7	STATECD	State code	NUMBER(4)
8	AREALAND_EU	Land area within the estimation unit	NUMBER(12,2)
9	AREATOT_EU	Total area within the estimation unit	NUMBER(12,2)
10	AREA_USED	Area used to calculate all expansion factors	NUMBER(12,2)
11	AREA_SOURCE	Area source	VARCHAR2(50)
12	P1PNTCNT_EU	Phase 1 point count for the estimation unit	NUMBER(12)
13	P1SOURCE	Phase 1 source	VARCHAR2(30)
14	CREATED_BY	Created by	VARCHAR2(30)
15	CREATED_DATE	Created date	DATE
16	CREATED_IN_INSTANCE	Created in instance	VARCHAR2(6)
17	MODIFIED_BY	Modified by	VARCHAR2(30)
18	MODIFIED_DATE	Modified date	DATE
19	MODIFIED_IN_INSTANCE	Modified in instance	VARCHAR2(6)

Type of key	Column(s) order	Tables to link	Abbreviated notation
Primary	(CN)	N/A	PEU_PK
Unique	(RSCD, EVALID, ESTN_UNIT)	N/A	PEU_UK
Foreign	(EVAL_CN)	POP_ESTN_UNIT to POP_EVAL	PEU_PEV_FK

1. CN — Sequence number. A unique sequence number used to identify an estimation unit record.

2. EVAL_CN — Evaluation sequence number. Foreign key linking the estimation unit record to the evaluation record.

3. RSCD — Region or Station Code. Identification number of the Forest Service National Forest System Region or Station (FIA work unit) that provided the inventory data (see appendix C for more information).

Code	Description
22	Rocky Mountain Research Station (RMRS)
23	North Central Research Station (NCRS)
24	Northeastern Research Station (NERS)
26	Pacific Northwest Research Station (PNWRS)
27	Pacific Northwest Research Station (PNWRS)-Alaska
33	Southern Research Station (SRS)

4. EVALID Evaluation identifier. The EVALID code and the RSCD code together uniquely identify a set of field plots and associated Phase 1 summary data used to make population estimates.

5. ESTN_UNIT Estimation unit. The specific geographic area that is stratified. Estimation units are often determined by a combination of geographical boundaries, sampling intensity and ownership.

6. ESTN_UNIT_DESCR

   Estimation unit description. A description of the estimation unit (e.g., name of the county).

7. STATECD State code. Bureau of the Census Federal Information Processing Standards (FIPS) two-digit code for each State. Refer to appendix C. For evaluations that do not conform to the boundaries of a single State the value of STATECD should be set to 99.

8. AREALAND_EU

   Land area within the estimation unit. The area of land in acres enclosed by the estimation unit. Census water is excluded.

9. AREATOT_EU

   Total area within the estimation unit. This includes land and census water enclosed by the estimation unit.

10. AREA_USED Area used to calculate all expansion factors. Is equivalent to AREATOT_EU if a station estimates all area, including census water; and to AREALAND_EU if a station estimates land area only.

11. AREA_SOURCE

    Area Source. Identifies the source of the area numbers. Usually the area source is either the U.S. Census Bureau or area estimates based on pixel counts. Example values are "US CENSUS 2000" or "PIXEL COUNT."

12. P1PNTCNT_EU

    Phase 1 point count for the estimation unit. For remotely sensed data this will be the total number of pixels in the estimation unit.

13. P1SOURCE Phase 1 source. Identifies the Phase 1 data source used for this stratification. Examples are NLCD and AERIAL PHOTOS.

14. CREATED_BY Created by. The employee who created the record. This attribute is intentionally left blank in download files.

15. CREATED_DATE

Created date. The date the record was created. Date will be in the form DD-MON-YYYY.

16. CREATED_IN_INSTANCE

Created in instance. The database instance in which the record was created. Each computer system has a unique database instance code and this attribute stores that information to determine on which computer the record was created.

17. MODIFIED_BY

Modified by. The employee who modified the record. This field will be blank (null) if the data have not been modified since initial creation. This attribute is intentionally left blank in download files.

18. MODIFIED_DATE

Modified date. The date the record was last modified. This field will be blank (null) if the data have not been modified since initial creation. Date will be in the form DD-MON-YYYY

19. MODIFIED_IN _INSTANCE

Modified in instance. The database instance in which the record was modified. This field will be blank (null) if the data have not been modified since initial creation.

Population Evaluation Table (Oracle table name is POP_EVAL)

	Column name	Descriptive name	Oracle data type
1	CN	Sequence number	VARCHAR2(34)
2	RSCD	Region or Station code	NUMBER(2)
3	EVALID	Evaluation identifier	NUMBER(6)
4	EVAL_DESCR	Evaluation description	VARCHAR2(255)
5	STATECD	State code	NUMBER(4)
6	LOCATION_NM	Location name	VARCHAR2(255)
7	REPORT_YEAR_NM	Report year name	VARCHAR2(255)
8	NOTES	Notes	VARCHAR2(2000)
9	CREATED_BY	Created by	VARCHAR2(30)
10	CREATED_DATE	Created date	DATE
11	CREATED_IN_INSTANCE	Created in instance	VARCHAR2(6)
12	MODIFIED_BY	Modified by	VARCHAR2(30)
13	MODIFIED_DATE	Modified date	DATE
14	MODIFIED_IN_INSTANCE	Modified in instance	VARCHAR2(6)
15	START_INVYR	Start inventory year	NUMBER(4)
16	END_INVYR	End inventory year	NUMBER(4)

Type of key	Column(s) order	Tables to link	Abbreviated notation
Primary	(CN)	N/A	PEV_PK
Unique	(RSCD, EVALID)	N/A	PEV_UK

1. CN — Sequence number. A unique sequence number used to identify an evaluation record.

2. RSCD — Region or Station Code. Identification number of the Forest Service National Forest System Region or Station (FIA work unit) that provided the inventory data (see appendix C for more information).

Code	Description
22	Rocky Mountain Research Station (RMRS)
23	North Central Research Station (NCRS)
24	Northeastern Research Station (NERS)
26	Pacific Northwest Research Station (PNWRS)
27	Pacific Northwest Research Station (PNWRS)-Alaska
33	Southern Research Station (SRS)

3. EVALID — Evaluation identifier. The EVALID code and the RSCD code together uniquely identify a set of field plots and associated Phase 1 summary data used to make population estimates.

4. EVAL_DESCR — Evaluation description. A description of the area being evaluated (often a State), the time period of the evaluation, and the type of estimates the

evaluation can be used to compute (i.e., all lands, area, volume, growth, removals, and mortality).

5. STATECD — State code. Bureau of the Census Federal Information Processing Standards (FIPS) two-digit code for each State. Refer to appendix C.

6. LOCATION_NM

   Location name. Geographic area as it would appear in the title of a report.

7. REPORT_YEAR_NM

   Report year name. The data collection years that would appear in the title of a report.

8. NOTES — Notes. Notes should include information about the stratification method. May include citation for any publications that used the evaluation.

9. CREATED_BY — Created by. The employee who created the record. This attribute is intentionally left blank in download files.

10. CREATED_DATE

    Created date. The date the record was created. Date will be in the form DD-MON-YYYY.

11. CREATED_IN_INSTANCE

    Created in instance. The database instance in which the record was created. Each computer system has a unique database instance code and this attribute stores that information to determine on which computer the record was created.

12. MODIFIED_BY

    Modified by. The employee who modified the record. This field will be blank (null) if the data have not been modified since initial creation. This attribute is intentionally left blank in download files.

13. MODIFIED_DATE

    Modified date The date the record was last modified. This field will be blank (null) if the data have not been modified since initial creation. Date will be in the form DD-MON-YYYY.

14. MODIFIED_IN_INSTANCE

    Modified in instance. The database instance in which the record was modified. This field will be blank (null) if the data have not been modified since initial creation.

15. START_INVYR

Start inventory year. The starting year for the data included in the evaluation.

16. END_INVYR End inventory year. The ending year for the data included in the evaluation.

Population Evaluation Attribute Table (Oracle table name is POP_EVAL_ATTRIBUTE)

	Column name	Descriptive name	Oracle data type
1	CN	Sequence number	VARCHAR2(34)
2	EVAL_CN	Evaluation sequence number	VARCHAR2(34)
3	ATTRIBUTE_NBR	Attribute number	NUMBER(3)
4	STATECD	State code	NUMBER(4)
5	CREATED_BY	Created by	VARCHAR2(30)
6	CREATED_DATE	Created date	DATE
7	CREATED_IN_INSTANCE	Created in instance	VARCHAR2(6)
8	MODIFIED_BY	Modified by	VARCHAR2(30)
9	MODIFIED_DATE	Modified date	DATE
10	MODIFIED_IN_INSTANCE	Modified in instance	VARCHAR2(6)

Type of key	Column(s) order	Tables to link	Abbreviated notation
Unique	(EVAL_CN, ATTRIBUTE_NBR)	N/A	PEA_UK
Foreign	(ATTRIBUTE_NBR)	POP_EVAL_ATTRIBUTE to REF_POP_ATTRIBUTE	PEA_PAE_FK
	(EVAL_CN)	POP_EVAL_ATTRIBUTE to POP_EVAL	PEA_PEV_FK

1. CN — Sequence number. A unique sequence number used to identify an evaluation attribute record.

2. EVAL_CN — Evaluation sequence number. Foreign key linking the population evaluation attribute record to the population evaluation record.

3. ATTRIBUTE_NBR

   Attribute number. Foreign key linking the population evaluation attribute record to the reference population attribute record.

4. STATECD — State code. Bureau of the Census Federal Information Processing Standards (FIPS) two-digit code for each State. Refer to appendix C.

5. CREATED_BY — Created by. The employee who created the record. This attribute is intentionally left blank in download files.

6. CREATED_DATE

   Created date. The date the record was created. Date will be in the form DD-MON-YYYY.

7. CREATED_IN_INSTANCE

Created in instance. The database instance in which the record was created. Each computer system has a unique database instance code and this attribute stores that information to determine on which computer the record was created.

8. MODIFIED_BY

Modified by. The employee who modified the record. This field will be blank (null) if the data have not been modified since initial creation. This attribute is intentionally left blank in download files.

9. MODIFIED_DATE

Modified date. The date the record was last modified. This field will be blank (null) if the data have not been modified since initial creation. Date will be in the form DD-MON-YYYY.

10. MODIFIED_IN_INSTANCE

Modified in instance. The database instance in which the record was modified. This field will be blank (null) if the data have not been modified since initial creation.

Population Evaluation Group Table (Oracle table name is POP_EVAL_GRP)

	Column name	Descriptive name	Oracle data type
1	CN	Sequence number	VARCHAR2(34)
2	EVAL_CN_FOR_EXPALL	Evaluation sequence number for expansions of all plots	VARCHAR2(34)
3	EVAL_CN_FOR_EXPCURR	Evaluation sequence number for expansions of current area	VARCHAR2(34)
4	EVAL_CN_FOR_EXPVOL	Evaluation sequence number for expansions of volume	VARCHAR2(34)
5	EVAL_CN_FOR_EXPGROW	Evaluation sequence number for expansions of growth	VARCHAR2(34)
6	EVAL_CN_FOR_EXPMORT	Evaluation sequence number for expansions of mortality	VARCHAR2(34)
7	EVAL_CN_FOR_EXPREMV	Evaluation sequence number for expansions of removals	VARCHAR2(34)
8	RSCD	Region or Station code	NUMBER(2)
9	EVAL_GRP	Evaluation group	NUMBER(6)
10	EVAL_GRP_DESCR	Evaluation group description	VARCHAR2(255)
11	STATECD	State code	NUMBER(4)
12	LAND_ONLY	Land only	VARCHAR2(1)
13	CREATED_BY	Created by	VARCHAR2(30)
14	CREATED_DATE	Created date	DATE
15	CREATED_IN_INSTANCE	Created in instance	VARCHAR2(6)
16	MODIFIED_BY	Modified by	VARCHAR2(30)
17	MODIFIED_DATE	Modified date	DATE
18	MODIFIED_IN_INSTANCE	Modified in instance	VARCHAR2(6)
19	NOTES	Notes	VARCHAR2(2000)

Type of key	Column(s) order	Tables to link	Abbreviated notation
Primary	(CN)	N/A	PEG_PK
Unique	(RSCD, EVAL_GRP)	N/A	PEG_UK
Foreign	(EVAL_CN_FOR_EXPALL)	POP_EVAL_GRP to POP_EVAL	PEG_PEV_FK
	(EVAL_CN_FOR_EXPCURR)	POP_EVAL_GRP to POP_EVAL	PEG_PEV_FK_2
	(EVAL_CN_FOR_EXPGROW)	POP_EVAL_GRP to POP_EVAL	PEG_PEV_FK_3
	(EVAL_CN_FOR_EXPMORT)	POP_EVAL_GRP to POP_EVAL	PEG_PEV_FK_4
	(EVAL_CN_FOR_EXPREMV)	POP_EVAL_GRP to POP_EVAL	PEG_PEV_FK_5
	(EVAL_CN_FOR_EXPVOL)	POP_EVAL_GRP to POP_EVAL	PEG_PEV_FK_6

1. CN Sequence number. A unique sequence number used to identify an evaluation group record.

2. EVAL_CN_FOR_EXPALL

Evaluation sequence number for expansions of all plots. This attribute links to the POP_EVAL.CN on the evaluation record. When this attribute is populated, it points to the evaluation used to estimate total area, including both sampled and nonsampled plots. Users must first obtain the correct sequence number in this attribute in order to run queries like those shown in chapter 4. This attribute will be dropped in version 5.0.

3. EVAL_CN_FOR_EXPCURR

Evaluation sequence number for expansions of current area. This attribute links to the POP_EVAL.CN on the evaluation record. When this attribute is populated, it points to the evaluation used to estimate total area, using only sampled plots. Users must first obtain the correct sequence number in this attribute in order to run queries like those shown in chapter 4. This attribute will be dropped in version 5.0.

4. EVAL_CN_FOR_EXPVOL

Evaluation sequence number for expansions of volume. This attribute links to the POP_EVAL.CN of the evaluation record. When this attribute is populated, it points to the evaluation used to estimate volume, biomass or number of trees, based on the sampled plots within the population that qualify for volume estimates. Users must first obtain the correct sequence number in this attribute in order to run queries like those shown in chapter 4. This attribute will be dropped in version 5.0.

5. EVAL_CN_FOR_EXPGROW

Evaluation sequence number for expansions of growth. This attribute links to the POP_EVAL.CN of the evaluation record. When this attribute is populated, it points to the evaluation used to estimate net average annual growth, based on the remeasured plots within the population that qualify for growth estimates. Users must first obtain the correct sequence number in this attribute in order to run queries like those shown in chapter 4. This attribute will be dropped in version 5.0.

6. EVAL_CN_FOR_EXPMORT

Evaluation sequence number for expansions of mortality. This attribute links to the POP_EVAL.CN of the evaluation record. When this attribute is populated, it points to the evaluation used to estimate average annual mortality, based on the remeasured plots within the population that qualify for mortality estimates. Users must first obtain the correct sequence number in this attribute in order to run queries like those shown in chapter 4. This attribute will be dropped in version 5.0.

7. EVAL_CN_FOR_EXPREMV

Evaluation sequence number for expansions of removals. This attribute links to the POP_EVAL.CN of the evaluation record. When this attribute is populated, it points to the evaluation used to estimate annual removals, based on the remeasured plots within the population that qualify for removals estimates. Users must first obtain the correct sequence number in this attribute in order to run queries like those shown in chapter 4. This attribute will be dropped in version 5.0.

8. RSCD — Region or Station Code. Identification number of the Forest Service National Forest System Region or Station (FIA work unit) that provided the inventory data (see appendix C for more information).

Code	Description
22	Rocky Mountain Research Station (RMRS)
23	North Central Research Station (NCRS)
24	Northeastern Research Station (NERS)
26	Pacific Northwest Research Station (PNWRS)
27	Pacific Northwest Research Station (PNWRS)-Alaska
33	Southern Research Station (SRS)

9. EVAL_GRP — Evaluation group. An evaluation group identifies the evaluations that were used in producing a core set of tables. In some cases one evaluation will be used for area and volume and another evaluation for growth, removals and mortality. The value of this attribute is used to select the appropriate State and year of interest to produce a set of summary tables.

10. EVAL_GRP_DESCR

Evaluation group description. A description of the evaluation group that includes the State and range of years for the evaluation, for example, “Minnesota: 1004;2005;2006;2007;2008”. This is useful to include in a summary report to clearly identify the source of the data.

11. STATECD — State code. Bureau of the Census Federal Information Processing Standards (FIPS) two-digit code for each State. Refer to appendix C. For evaluations that do not conform to the boundaries of a single State the value of STATECD should be set to 99.

12. LAND_ONLY — Land only. A code indicating area used in stratifying evaluations. See POP_ESTN_UNIT.AREA_SOURCE for more information.

Code	Description
Y	Only census land was used in the stratification process.
N	Census land and water were used in the stratification process.

13. CREATED_BY — Created by. The employee who created the record. This attribute is intentionally left blank in download files.

14. CREATED_DATE

Created date. The date the record was created. Date will be in the form DD-MON-YYYY.

15. CREATED_IN_INSTANCE

Created in instance. The database instance in which the record was created. Each computer system has a unique database instance code and this attribute stores that information to determine on which computer the record was created.

16. MODIFIED_BY

Modified by. The employee who modified the record. This field will be blank (null) if the data have not been modified since initial creation. This attribute is intentionally left blank in download files.

17. MODIFIED_DATE

Modified date. The date the record was last modified. This field will be blank (null) if the data have not been modified since initial creation. Date will be in the form DD-MON-YYYY.

18. MODIFIED_IN_INSTANCE

Modified in instance. The database instance in which the record was modified. This field will be blank (null) if the data have not been modified since initial creation.

19. NOTES Notes. An optional item where additional information about the evaluation group may be stored.

Population Evaluation Type Table (Oracle table name is POP_EVAL_TYP)

	Column name	Descriptive name	Oracle data type
1	EVAL_GRP_CN	Evaluation group sequence number	VARCHAR2(34)
2	EVAL_CN	Evaluation sequence number	VARCHAR2(34)
3	EVAL_TYP	Evaluation type	VARCHAR2(15)
4	STATECD	State code	NUMBER(4)
5	CREATED_BY	Created by	VARCHAR2(30)
6	CREATED_DATE	Created date	DATE
7	CREATED_IN_INSTANCE	Created in instance	VARCHAR2(6)
8	MODIFIED_BY	Modified by	VARCHAR2(30)
9	MODIFIED_DATE	Modified date	DATE
10	MODIFIED_IN_INSTANCE	Modified in instance	VARCHAR2(6)
11	CN	Sequence number	VARCHAR2(34)

Type of key	Column(s) order	Tables to link	Abbreviated notation
Primary	(CN)	N/A	PET_PK
Unique	(EVAL_GRP_CN, EVAL_CN, EVAL_TYP)	N/A	PET_UK
Foreign	(EVAL_GRP_CN)	POP_EVAL_TYP to POP_EVAL_GRP	PET_PEG_FK
	(EVAL_CN)	POP_EVAL_TYP to POP_EVAL	PET_PEV_FK
	(EVAL_TYP)	POP_EVAL_TYP to REF_POP_EVAL_TYP_DESCR	PET_PED_FK

1. EVAL_GRP_CN

   Evaluation group sequence number. Foreign key linking the population evaluation type record to the population evaluation group record.

2. EVAL_CN — Evaluation sequence number. Foreign key linking the population evaluation type record to the population evaluation record.

3. EVAL_TYP — Evaluation type. Describes the type of evaluation. Evaluation type is needed to generate summary reports for an inventory. For example, a specific evaluation is associated with the evaluation for volume (Expvol). At the present time, seven types of evaluations can be produced. See also the REF_POP_EVAL_TYP_DESCR table.

   Evaluation type values
   Expall
   Expchng
   Expcurr
   Expgrow
   Expmort
   Expremv
   Expvol

4. STATECD State code. Bureau of the Census Federal Information Processing Standards (FIPS) two-digit code for each State. Refer to appendix C.

5. CREATED_BY Created by. The employee who created the record. This attribute is intentionally left blank in download files.

6. CREATED_DATE

   Created date. The date the record was created. Date will be in the form DD-MON-YYYY.

7. CREATED_IN_INSTANCE

   Created in instance. The database instance in which the record was created. Each computer system has a unique database instance code and this attribute stores that information to determine on which computer the record was created.

8. MODIFIED_BY

   Modified by. The employee who modified the record. This field will be blank (null) if the data have not been modified since initial creation. This attribute is intentionally left blank in download files.

9. MODIFIED_DATE

   Modified date. The date the record was last modified. This field will be blank (null) if the data have not been modified since initial creation. Date will be in the form DD-MON-YYYY.

10. MODIFIED_IN_INSTANCE

    Modified in instance. The database instance in which the record was modified. This field will be blank (null) if the data have not been modified since initial creation.

11. CN Sequence number. A unique sequence number used to identify a population evaluation type record

Population Plot Stratum Assignment Table (Oracle table name is POP_PLOT_STRATUM_ASSGN)

	Colum name	Descriptive name	Oracle data type
1	CN	Sequence number	VARCHAR2(34)
2	STRATUM_CN	Stratum sequence number	VARCHAR2(34)
3	PLT_CN	Plot sequence number	VARCHAR2(34)
4	STATECD	State code	NUMBER(4)
5	INVYR	Inventory year	NUMBER(4)
6	UNITCD	Survey unit code	NUMBER(2)
7	COUNTYCD	County code	NUMBER(3)
8	PLOT	Phase 2 plot number	NUMBER(5)
9	RSCD	Region or Station code	NUMBER(2)
10	EVALID	Evaluation identifier	NUMBER(6)
11	ESTN_UNIT	Estimation unit	NUMBER(6)
12	STRATUMCD	Stratum code	NUMBER(6)
13	CREATED_BY	Created by	VARCHAR2(30)
14	CREATED_DATE	Created date	DATE
15	CREATED_IN_INSTANCE	Created in instance	VARCHAR2(6)
16	MODIFIED_BY	Modified by	VARCHAR2(30)
17	MODIFIED_DATE	Modified date	DATE
18	MODIFIED_IN_INSTANCE	Modified in instance	VARCHAR2(6)

Type of key	Column(s) order	Tables to link	Abbreviated notation
Primary	(CN)	N/A	PPSA_PK
Unique	(PLT_CN, STRATUM_CN)	N/A	PPSA_UK
	(STATECD, INVYR, UNITCD, COUNTYCD, PLOT, RSCD, EVALID, ESTN_UNIT, STRATUMCD)	N/A	PPSA_UK2
Foreign	(PLT_CN)	POP_PLOT_STRATUM_ASSGN to PLOT	PPSA_PLT_FK
	(STRATUM_CN)	POP_PLOT_STRATUM_ASSGN to POP_STRATUM	PPSA_PSM_FK

1. CN Sequence number. A unique sequence number used to identify a population plot stratum assignment record.

2. STRATUM_CN

   Stratum sequence number. Foreign key linking the population plot stratum assignment record to the population stratum record.

3. PLT_CN — Plot sequence number. Foreign key linking the population plot stratum assignment record to the plot record.

4. STATECD — State code. Bureau of the Census Federal Information Processing Standards (FIPS) two-digit code for each State. Refer to appendix C.

5. INVYR — Inventory year. The year that best represents when the inventory data were collected. Under the annual inventory system, a group of plots is selected each year for sampling. The selection is based on a panel system. INVYR is the year in which the majority of plots in that group were collected (plots in the group have the same panel and, if applicable, subpanel). Under periodic inventory, a reporting inventory year was selected, usually based on the year in which the majority of the plots were collected or the mid-point of the years over which the inventory spanned. For either annual or periodic inventory, INVYR is not necessarily the same as MEASYEAR.

   Exceptions:
   INVYR = 9999. INVYR is set to 9999 to distinguish Phase 3 plots taken by the western FIA work units that are "off subpanel." This is due to differences in measurement intervals between Phase 3 (measurement interval = 5 years) and Phase 2 (measurement interval = 10 years) plots. Only users interested in performing certain Phase 3 data analyses should access plots with this anomalous value in INVYR.

   INVYR <100. INVYR <100 indicates that population estimates were derived from a pre-NIMS regional processing system and the same plot either has been or may soon be re-processed in NIMS as part of a separate evaluation. The NIMS processed copy of the plot follows the standard INVYR format. This only applies to plots collected in the South (RSCD = 33) with the national design or a similar regional design (PLOT.DESIGNCD = 1 or 220-233) that were collected when the inventory year was 1998 through 2005.

   INVYR = 98 is equivalent to 1998 but processed through regional system
   INVYR = 99 is equivalent to 1999 but processed through regional system
   INVYR = 0 is equivalent to 2000 but processed through regional system
   INVYR = 1 is equivalent to 2001 but processed through regional system
   INVYR = 2 is equivalent to 2002 but processed through regional system
   INVYR = 3 is equivalent to 2003 but processed through regional system
   INVYR = 4 is equivalent to 2004 but processed through regional system
   INVYR = 5 is equivalent to 2005 but processed through regional system

6. UNITCD — Survey unit code. Forest Inventory and Analysis survey unit identification number. Survey units are usually groups of counties within each State. For periodic inventories, Survey units may be made up of lands of particular owners. Refer to appendix C for codes.

7. COUNTYCD — County code. The identification number for a county, parish, watershed, borough, or similar governmental unit in a State. FIPS codes from the Bureau of the Census are used. Refer to appendix C for codes.

8. PLOT — Phase 2 plot number. An identifier for a plot. Along with INVYR, STATECD, UNITCD, COUNTYCD, PLOT may be used to uniquely identify a plot.

9. RSCD — Region or Station Code. Identification number of the Forest Service National Forest System Region or Station (FIA work unit) that provided the inventory data (see appendix C for more information).

Code	Description
22	Rocky Mountain Research Station (RMRS)
23	North Central Research Station (NCRS)
24	Northeastern Research Station (NERS)
26	Pacific Northwest Research Station (PNWRS)
27	Pacific Northwest Research Station (PNWRS) - Alaska
33	Southern Research Station (SRS)

10. EVALID — Evaluation identifier. The EVALID code and the RSCD code together uniquely identify a set of field plots and associated Phase 1 summary data used to make population estimates.

11. ESTN_UNIT — Estimation unit. A geographic area upon which stratification is performed. Sampling intensity is uniform within an estimation unit.

12. STRATUMCD — Stratum code. The code used for a particular stratum, which is unique within an RSCD, EVALID, ESTN_UNIT.

13. CREATED_BY — Created by. The employee who created the record. This attribute is intentionally left blank in download files.

14. CREATED_DATE

Created date. The date the record was created. Date will be in the form DD-MON-YYYY.

15. CREATED_IN_INSTANCE

Created in instance. The database instance in which the record was created. Each computer system has a unique database instance code and this attribute stores that information to determine on which computer the record was created.

16. MODIFIED_BY

Modified by. The employee who modified the record. This field will be blank (null) if the data have not been modified since initial creation. This attribute is intentionally left blank in download files.

17. MODIFIED_DATE

Modified date. The date the record was last modified. This field will be blank (null) if the data have not been modified since initial creation. Date will be in the form DD-MON-YYYY.

18. MODIFIED_IN_INSTANCE

Modified in instance. The database instance in which the record was modified. This field will be blank (null) if the data have not been modified since initial creation.

Population Stratum Table (Oracle table name is POP_STRATUM)

	Column name	Descriptive name	Oracle data type
1	CN	Sequence number	VARCHAR2(34)
2	ESTN_UNIT_CN	Estimation unit sequence number	VARCHAR2(34)
3	RSCD	Region or Station code	NUMBER(2)
4	EVALID	Evaluation identifier	NUMBER(6)
5	ESTN_UNIT	Estimation unit	NUMBER(6)
6	STRATUMCD	Stratum code	NUMBER(6)
7	STRATUM_DESCR	Stratum description	VARCHAR2(255)
8	STATECD	State code	NUMBER(4)
9	P1POINTCNT	Phase 1 point count	NUMBER(12)
10	P2POINTCNT	Phase 2 point count	NUMBER(12)
11	EXPNS	Expansion factor	NUMBER
12	ADJ_FACTOR_MACR	Adjustment factor for the macroplot	NUMBER(5,4)
13	ADJ_FACTOR_SUBP	Adjustment factor for the subplot	NUMBER(5,4)
14	ADJ_FACTOR_MICR	Adjustment factor for the microplot	NUMBER(5,4)
15	CREATED_BY	Created by	VARCHAR2(30)
16	CREATED_DATE	Created date	DATE
17	CREATED_IN_INSTANCE	Created in instance	VARCHAR2(6)
18	MODIFIED_BY	Modified by	VARCHAR2(30)
19	MODIFIED_DATE	Modified date	DATE
20	MODIFIED_IN_INSTANCE	Modified in instance	VARCHAR2(6)

Type of key	Column(s) order	Tables to link	Abbreviated notation
Primary	(CN)	N/A	PSM_PK
Unique	(RSCD, EVALID, ESTN_UNIT, STRATUMCD)	N/A	PSM_UK
Foreign	(ESTN_UNIT_CN)	POP_STRATUM to POP_ESTN_UNIT	PSM_PEU_FK

1. CN — Sequence number. A unique sequence number used to identify a stratum record.

2. ESTN_UNIT_CN

   Estimation unit sequence number. Foreign key linking the stratum record to the estimation unit record.

3. RSCD — Region or Station Code. Identification number of the Forest Service National Forest System Region or Station (FIA work unit) that provided the inventory data (see appendix C for more information).

Code	Description
22	Rocky Mountain Research Station (RMRS)
23	North Central Research Station (NCRS)
24	Northeastern Research Station (NERS)
26	Pacific Northwest Research Station (PNWRS)
27	Pacific Northwest Research Station (PNWRS)-Alaska
33	Southern Research Station (SRS)

4. EVALID — Evaluation identifier. The EVALID code and the RSCD code together uniquely identify a set of field plots and associated Phase 1 summary data used to make population estimates.

5. ESTN_UNIT — Estimation unit. The particular geographic area for which a particular computation applies. Estimation units are determined by a combination of sampling intensity and geographical boundaries.

6. STRATUMCD — Stratum code. A number used to uniquely identify a stratum within an estimation unit.

7. STRATUM_DESCR

   Stratum description. Strata are usually based on land use (e.g., forest or nonforest) but may also be based on other criteria such as ownership (e.g., private/public/national forest).

8. STATECD — State code. Bureau of the Census Federal Information Processing Standards (FIPS) two-digit code for each State. Refer to appendix C. For evaluations that do not conform to the boundaries of a single State the value of STATECD should be set to 99.

9. P1POINTCNT — Phase 1 point count. The number of basic units (pixels or points) in the stratum.

10. P2POINTCNT — Phase 2 point count. The number of field plots that are within the stratum.

11. EXPNS — Expansion factor. The area, in acres, that a stratum represents divided by the number of sampled plots in that stratum. This attribute can be used to obtain estimates of population area when summed across all the plots in the population of interest. Refer to chapter 4 for detailed examples.

12. ADJ_FACTOR_MACR

   Adjustment factor for the macroplot. A value that adjusts the population estimates to account for partially nonsampled plots (access denied and hazardous portions). It is used with condition proportion (COND.CONDPROP_UNADJ) and area expansion (EXPNS) to provide area estimates, when COND.PROP_BASIS = “MACR”. ADJ_FACTOR_MACR is also used with EXPNS and trees per acre unadjusted (TREE.TPA_UNADJ, TREE.TPAMORT_UNADJ, TREE.TPAREMV_UNADJ, TREE.TPAGROW_UNADJ) to provide tree estimates for sampled land. If a

macroplot was not installed, this attribute is left blank (null). Refer to chapter 4 for detailed examples.

13. ADJ_FACTOR_SUBP

Adjustment factor for the subplot. A value that adjusts the population estimates to account for partially nonsampled plots (access denied and hazardous portions). It is used with condition proportion (COND.CONDPROP_UNADJ) and area expansion (EXPNS) to provide area estimates, when COND.PROP_BASIS = "SUBP". ADJ_FACTOR_SUBP is also used with EXPNS and trees per acre unadjusted (TREE.TPA_UNADJ, TREE.TPAMORT_UNADJ, TREE.TPAREMV_UNADJ, TREE.TPAGROW_UNADJ) to provide tree estimates for sampled land. Refer to chapter 4 for detailed examples.

14. ADJ_FACTOR_MICR

Adjustment factor for the microplot. A value that adjusts population estimates to account for partially nonsampled plots (access denied and hazardous portions). It is used with POP_STRATUM.EXPNS and seedlings per acre unadjusted (SEEDLING.TPA_UNADJ) or saplings per acre unadjusted (TREE.TPA_UNADJ where TREE DIA <5.0) to provide tree estimates for sampled land. Refer to chapter 4 for detailed examples.

15. CREATED_BY Created by. The employee who created the record. This attribute is intentionally left blank in download files.

16. CREATED_DATE

Created date. The date the record was created. Date will be in the form DD-MON-YYYY.

17. CREATED_IN_INSTANCE

Created in instance. The database instance in which the record was created. Each computer system has a unique database instance code and this attribute stores that information to determine on which computer the record was created.

18. MODIFIED_BY

Modified by. The employee who modified the record. This field will be blank (null) if the data have not been modified since initial creation. This attribute is intentionally left blank in download files.

19. MODIFIED_DATE

Modified date. The date the record was last modified. This field will be blank (null) if the data have not been modified since initial creation. Date will be in the form DD-MON-YYYY.

20. MODIFIED_IN _INSTANCE

Modified in instance. The database instance in which the record was modified. This field will be blank (null) if the data have not been modified since initial creation.

Reference Population Attribute Table (Oracle table name is REF_POP_ATTRIBUTE)

	Column name	Descriptive name	Oracle data type
1	CN	Sequence number	VARCHAR2(34)
2	ATTRIBUTE_NBR	Attribute number	NUMBER(3)
3	ATTRIBUTE_DESCR	Attribute description	VARCHAR2(255)
4	EXPRESSION	Expression	VARCHAR2(2000)
5	WHERE_CLAUSE	Where clause	VARCHAR2(255)
6	CREATED_BY	Created by	VARCHAR2(30)
7	CREATED_DATE	Created date	DATE
8	CREATED_IN_INSTANCE	Created in instance	VARCHAR2(6)
9	MODIFIED_BY	Modified by	VARCHAR2(30)
10	MODIFIED_DATE	Modified date	DATE
11	MODIFIED_IN_INSTANCE	Modified in instance	VARCHAR2(6)
12	FOOTNOTE	Footnote	VARCHAR2(2000)

Type of key	Column(s) order	Tables to link	Abbreviated notation
Primary	(ATTRIBUTE_NBR)	N/A	PAE_PK

1. CN — Sequence number. A unique sequence number used to identify a reference population attribute record.

2. ATTRIBUTE_NBR

   Attribute number. A numeric code used to identify an attribute record. See codes and descriptions in chapter 4, table 4.1.

3. ATTRIBUTE_DESCR

   Attribute description. Examples include "Area of forestland (acres)" or "All live biomass on forestland oven-dry (short tons)." See codes and descriptions in chapter 4, table 4.1.

4. EXPRESSION

   Expression. SQL expression that identifies variables that are used to generate population estimate identified by ATTRIBUTE_DESCR (chapter 4, table 4.2).

5. WHERE_CLAUSE

   Where clause. SQL where clause that identifies the appropriate method for joining tables and screening records to generate population estimate identified by REF_POP_ATTRIBUTE.ATTRIBUTE_DESCR (chapter 4, table 4.2).

6. CREATED_BY Created by. The employee who created the record. This attribute is intentionally left blank in download files.

7. CREATED_DATE

   Created date. The date the record was created. Date will be in the form DD-MON-YYYY.

8. CREATED_IN_INSTANCE

   Created in instance. The database instance in which the record was created. Each computer system has a unique database instance code and this attribute stores that information to determine on which computer the record was created.

9. MODIFIED_BY

   Modified by. The employee who modified the record. This field will be blank (null) if the data have not been modified since initial creation. This attribute is intentionally left blank in download files.

10. MODIFIED_DATE

    Modified date. The date the record was last modified. This field will be blank (null) if the data have not been modified since initial creation. Date will be in the form DD-MON-YYYY.

11. MODIFIED_IN_INSTANCE

    Modified in instance. The database instance in which the record was modified. This field will be blank (null) if the data have not been modified since initial creation.

12. FOOTNOTE Footnote. Intentionally left blank. Will be populated in verion 5.0

Reference Population Evaluation Type Description Table (Oracle table name is REF_POP_EVAL_TYP_DESCR)

	Column name	Descriptive name	Oracle data type
1	EVAL_TYP	Evaluation type	VARCHAR2(15)
2	EVAL_TYP_DESCR	Evaluation type description	VARCHAR2(255)
3	CREATED_BY	Created by	VARCHAR2(30)
4	CREATED_DATE	Created date	DATE
5	CREATED_IN_INSTANCE	Created in instance	VARCHAR2(6)
6	MODIFIED_BY	Modified by	VARCHAR2(30)
7	MODIFIED_DATE	Modified date	DATE
8	MODIFIED_IN_INSTANCE	Modified in instance	VARCHAR2(6)
9	CN	Sequence number	VARCHAR2(34)

Type of key	Column(s) order	Tables to link	Abbreviated notation
Primary	(CN)	N/A	PED_PK
Unique	(EVAL_TYP)	N/A	PED_UK

1. EVAL_TYP Evaluation type. Evaluation types (EVAL_TYP) and the description of the evaluation types (EVAL_TYP_DESCR) are:

Evaluation type	Evaluation type description
Expall	All plots: sampled and nonsampled
Expchng	Sampled plots used for area change estimates
Expcurr	Sampled plots used for current area estimates
Expgrow	Sampled plots used for tree growth estimates
Expmort	Sampled plots used for tree mortality estimates
Expremv	Sampled plots used for tree removal estimates
Expvol	Sampled plots used for tree inventory estimates

2. EVAL_TYP_DESCR

   Evaluation type description. The description for each evaluation type (EVAL_TYP). See the list of codes and descriptions in EVAL_TYP.

.

3. CREATED_BY Created by. The employee who created the record. This attribute is intentionally left blank in download files.

4. CREATED_DATE

   Created date. The date the record was created. Date will be in the form DD-MON-YYYY.

5. CREATED_IN_INSTANCE

   Created in instance. The database instance in which the record was created. Each computer system has a unique database instance code and this attribute

stores that information to determine on which computer the record was created.

6. MODIFIED_BY

Modified by. The employee who modified the record. This field will be blank (null) if the data have not been modified since initial creation. This attribute is intentionally left blank in download files.

7. MODIFIED_DATE

Modified date. The date the record was last modified. This field will be blank (null) if the data have not been modified since initial creation. Date will be in the form DD-MON-YYYY.

8. MODIFIED_IN_INSTANCE

Modified in instance. The database instance in which the record was modified. This field will be blank (null) if the data have not been modified since initial creation.

9. CN

Sequence number. A unique sequence number used to identify a reference population evaluation type description record.

Reference Forest Type Table (Oracle table name is REF_FOREST_TYPE)

	Column name	Descriptive name	Oracle data type
1	VALUE	Value	NUMBER(3)
2	MEANING	Meaning	VARCHAR2(80)
3	TYPGRPCD	Forest type group code	NUMBER(3)
4	MANUAL_START	Manual start	NUMBER(3,1)
5	MANUAL_END	Manual end	NUMBER(3,1)
6	ALLOWED_IN_FIELD	Allowed in field	VARCHAR2(1)
7	CREATED_BY	Created by	VARCHAR2(30)
8	CREATED_DATE	Created date	DATE
9	CREATED_IN_INSTANCE	Created in instance	VARCHAR2(6)
10	MODIFIED_BY	Modified by	VARCHAR2(30)
11	MODIFIED_DATE	Modified date	DATE
12	MODIFIED_IN_INSTANCE	Modified in instance	VARCHAR2(6)

Type of key	Column(s) order	Tables to link	Abbreviated notation
Primary	(VALUE)	N/A	RFT_PK

1. VALUE Value. A code used for the forest type (COND.FORTYPCD, COND.FLDTYPCD, COND.FORTYPCDCALC). Refer to appendix D.

2. MEANING Meaning. The descriptive name corresponding with the forest type code (VALUE). The names associated with these codes are used to label rows or columns in National standard presentation tables. Refer to appendix D.

3. TYPGRPCD Forest type group code. A code assigned to individual forest types in order to group them for reporting purposes. Refer to appendix D.

4. MANUAL_START

   Manual start. The first version of the Field Guide (PLOT.MANUAL) that the forest type code (VALUE) began to be used.

5. MANUAL_END

   Manual end. The last version of the Field Guide (PLOT.MANUAL) that the forest type code (VALUE) was valid. When MANUAL_END is blank (null), the code is still valid.

6. ALLOWED_IN_FIELD

   Allowed in field. An indicator to show if a code (VALUE) is allowed to be used by the field crews. This is a Yes/No (Y/N) field. Specifically, forest type group codes are not allowed in the Field Guide nor is the code for a nonstocked forest type (VALUE = 999).

7 CREATED_BY Created by. The employee who created the record. This attribute is intentionally left blank in download files.

8. CREATED_DATE

Created date. The date the record was created. Date will be in the form DD-MON-YYYY.

9. CREATED_IN_INSTANCE

Created in instance. The database instance in which the record was created. Each computer system has a unique database instance code and this attribute stores that information to determine on which computer the record was created.

10. MODIFIED_BY

Modified by. The employee who modified the record. This field will be blank (null) if the data have not been modified since initial creation. This attribute is intentionally left blank in download files.

11. MODIFIED_DATE

Modified date. The date the record was last modified. This field will be blank (null) if the data have not been modified since initial creation. Date will be in the form DD-MON-YYYY.

12. MODIFIED_IN_INSTANCE

Modified in instance. The database instance in which the record was modified. This field will be blank (null) if the data have not been modified since initial creation.

Reference Species Table (Oracle table name is REF_SPECIES)

	Column name	Descriptive name	Oracle data type
1	SPCD	Species code	NUMBER
2	COMMON_NAME	Common name of species	VARCHAR2(100)
3	GENUS	Genus	VARCHAR2(40)
4	SPECIES	Species name	VARCHAR2(50)
5	VARIETY	Variety	VARCHAR2(50)
6	SUBSPECIES	Subspecies name	VARCHAR2(50)
7	SPECIES_SYMBOL	Species symbol	VARCHAR2(8)
8	E_SPGRPCD	East species group code	NUMBER(2)
9	W_SPGRPCD	West species group code	NUMBER(2)
10	MAJOR_SPGRPCD	Major species group code	NUMBER(1)
11	STOCKING_SPGRPCD	Stocking species group code	NUMBER(3)
12	FOREST_TYPE_SPGRPCD	Forest type species group code	NUMBER(3)
13	EXISTS_IN_NCRS	Exists in the North Central Research Station States	VARCHAR2(1)
14	EXISTS_IN_NERS	Exists in the Northeastern Research Station States	VARCHAR2(1)
15	EXISTS_IN_PNWRS	Exists in the Pacific Northwest Research Station States	VARCHAR2(1)
16	EXISTS_IN_RMRS	Exists in the Rocky Mountain Research Station States	VARCHAR2(1)
17	EXISTS_IN_SRS	Exists in the Southern Research Station States	VARCHAR2(1)
18	SITETREE	Site tree	VARCHAR2(1)
19	SFTWD_HRDWD	Softwood or hardwood	VARCHAR2(1)
20	ST_EXISTS_IN_NCRS	Site tree exists in the North Central Research Station region	VARCHAR2(1)
21	ST_EXISTS_IN_NERS	Site tree exists in the Northeastern Research Station region	VARCHAR2(1)
22	ST_EXISTS_IN_PNWRS	Site tree exists in the Pacific Northwest Research Station region	VARCHAR2(1)
23	ST_EXISTS_IN_RMRS	Site tree exists in the Rocky Mountain Research Station region	VARCHAR2(1)
24	ST_EXISTS_IN_SRS	Site tree exists in the Southern Research Station region	VARCHAR2(1)
25	EAST	East	VARCHAR2(1)
26	WEST	West	VARCHAR2(1)
27	WOODLAND	Woodland species	VARCHAR2(1)
28	MANUAL_START	Manual start	NUMBER(3,1)
29	MANUAL_END	Manual end	NUMBER(3,1)
30	CREATED_BY	Created by	VARCHAR2(30)
31	CREATED_DATE	Created date	DATE
32	CREATED_IN_INSTANCE	Created in instance	VARCHAR2(6)

	Column name	Descriptive name	Oracle data type
33	MODIFIED_BY	Modified by	VARCHAR2(30)
34	MODIFIED_DATE	Modified date	DATE
35	MODIFIED_IN_INSTANCE	Modified in instance	VARCHAR2(6)
36	CORE	Core	VARCHAR2(1)
37	JENKINS_SPGRPCD	Jenkins species group code	NUMBER(8,5)
38	JENKINS_TOTAL_B1	Jenkins total B1	NUMBER(8,5)
39	JENKINS_TOTAL_B2	Jenkins total B2	NUMBER(8,5)
40	JENKINS_STEM_WOOD_RATIO_B1	Jenkins stem wood ratio B1	NUMBER(8,5)
41	JENKINS_STEM_WOOD_RATIO_B2	Jenkins stem wood ratio B2	NUMBER(8,5)
42	JENKINS_STEM_BARK_RATIO_B1	Jenkins stem bark ratio B1	NUMBER(8,5)
43	JENKINS_STEM_BARK_RATIO_B2	Jenkins stem bark ratio B2	NUMBER(8,5)
44	JENKINS_FOLIAGE_RATIO_B1	Jenkins foliage ratio B1	NUMBER(8,5)
45	JENKINS_FOLIAGE_RATIO_B2	Jenkins foliage ratio B2	NUMBER(8,5)
46	JENKINS_ROOT_RATIO_B1	Jenkins root ratio B1	NUMBER(8,5)
47	JENKINS_ROOT_RATIO_B2	Jenkins root ratio B2	NUMBER(8,5)
48	JENKINS_SAPLING_ADJUSTMENT	Jenkins sapling adjustment factor	NUMBER(8,5)
49	WOOD_SPGR_GREENVOL_DRYWT	Green specific gravity wood (green volume and oven-dry weight)	NUMBER(8,5)
50	WOOD_SPGR_GREENVOL_DRYWT_CIT	Green specific gravity wood citation	NUMBER(7)
51	BARK_SPGR_GREENVOL_DRYWT	Green specific gravity bark (green volume and oven-dry weight)	NUMBER(8,5)
52	BARK_SPGR_GREENVOL_DRYWT_CIT	Green specific gravity bark citation	NUMBER(7)
53	MC_PCT_GREEN_WOOD	Moisture content of green wood as a percent of oven-dry weight	NUMBER(8,5)
54	MC_PCT_GREEN_WOOD_CIT	Moisture content of green wood citation	NUMBER(7)
55	MC_PCT_GREEN_BARK	Moisture content of green bark as a percent of oven-dry weight	NUMBER(8,5)
56	MC_PCT_GREEN_BARK_CIT	Moisture content of green bark citation	NUMBER(7)
57	WOOD_SPGR_MC12VOL_DRYWT	Wood specific gravity (12 percent moisture content volume and oven-dry weight)	NUMBER(8,5)
58	WOOD_SPGR_MC12VOL_DRYWT_CIT	Wood specific gravity (12 percent moisture content volume and oven-dry weight) citation	NUMBER(7)
59	BARK_VOL_PCT	Bark volume as a percent of wood volume	NUMBER(8,5)
60	BARK_VOL_PCT_CIT	Bark volume as a percent of wood volume citation	NUMBER(7)
61	RAILE_STUMP_DOB_B1	Raile stump diameter outside bark equation coefficient B1	NUMBER(8,5)
62	RAILE_STUMP_DIB_B1	Raile stump diameter inside bark equation coefficient B1	NUMBER(8,5)

	Column name	Descriptive name	Oracle data type
63	RAILE_STUMP_DIB_B2	Raile stump diameter inside bark equation coefficient B2	NUMBER(8,5)

Type of key	Column(s) order	Tables to link	Abbreviated notation
Primary	(SPCD)	N/A	SPC_PK
Unique	(SPECIES_SYMBOL)	N/A	SPC_UK

Coefficients for calculating total aboveground biomass based on Jenkins and others (2003) equations are included in the REF_SPECIES table. Coefficients for calculating biomass components (stem wood, stem bark, foliage, coarse roots, stump, and sapling) are also included in the REF_SPECIES table. Biomass in branches and treetops (tops and limbs) may be found by subtracting the biomass in stem wood, stem bark, foliage, and stump from total aboveground biomass. Heath and others (2009) provides an overview of the historical use of Jenkins and others (2003) for biomass estimation for the U.S. forest greenhouse gas inventory (U.S. Environmental Protection Agency 2008) and an overview of the approach of the new biomass equations used for FIA data.

1. SPCD — Species code. An FIA tree species code. Refer to appendix F for codes.

2. COMMON_NAME

   Common name. Common name of the species. Refer to appendix F.

3. GENUS — Genus. The genus name associated with the FIA tree species code. Refer to appendix F.

4. SPECIES — Species. The species name associated with the FIA tree species code. Refer to appendix F.

5. VARIETY — Variety. The variety name associated with the FIA tree species code.

6. SUBSPECIES — Subspecies. The subspecies name associated with the FIA tree species code.

7. SPECIES_SYMBOL

   Species symbol. The NRCS PLANTS database code associated with the FIA tree species code.

8. E_SPGRPCD — Eastern species group code. A code indicating the species group assignment for eastern species. Depending on the State in which a tree is tallied, either the eastern or western species group code is associated with the actual TREE, SITETREE, and SEEDLING data. Species group codes and names can be found in appendix G.

9. W_SPGRPCD — Western species group code. A code indicating the FIADB species group assignment for western species. Depending on the State in which a tree is tallied, either the eastern or western species group code is associated with the

actual TREE, SITETREE, and SEEDLING data. Species group codes and names can be found in appendix G.

10. MAJOR_SPGRPCD

Major species group code. A code indicating the major species group, which can be used for reporting purposes.

Code	Description
1	Pine
2	Other conifers
3	Soft hardwood
4	Hard hardwood

11. STOCKING_SPGRPCD

Stocking species group code. A code indicating which stocking equation a species is assigned.

Code	Description
1	Spruce-fir
2	Western larch
3	Black spruce
4	Jack pine
5	Lodgepole pine
6	Shortleaf pine
7	Slash pine
8	Western white pine
9	Longleaf pine
10	Ponderosa pine
11	Red pine
12	Pond pine
13	Eastern white pine
14	Loblolly pine
15	Douglas-fir
16	Northern white cedar
17	Eastern hemlock
18	Western hemlock
19	Redwood
20	Average softwood
25	Red maple
26	Red alder
27	Maple, beech, birch
28	Paper birch
29	Oaks and hickory
30	Black walnut
31	Sweetgum
32	Aspen
33	Cherry, ash, yellow poplar
35	Basswood
36	Elm, ash, cottonwood
37	Average hardwood
38	Dryland species

12. FOREST_TYPE_SPGRPCD

Forest type species group code. A code indicating which initial forest type group a species is assigned.

13. EXISTS_IN_NCRS

Exists in the North Central Research Station. Indicates which species are valid for North Central Research Station States. Trees that are applicable to North Central States are marked with an X.

14. EXISTS_IN_NERS

Exists in the Northeastern Research Station. Indicates which tree species are valid for Northeastern Research Station States. Tree species that are applicable to Northeastern States are marked with an X.

15. EXISTS_IN_PNWRS

Exists in the Pacific Northwest Research Station. Indicates which species are valid for Pacific Northwest Research Station States. Tree species that are applicable to Pacific Northwest States are marked with an X.

16. EXISTS_IN RMRS

Exists in the Rocky Mountain Research Station. Indicates which species are valid for Rocky Mountain Research Station States. Tree species that are applicable to the Rocky Mountain States are marked with an X.

17. EXISTS_IN_SRS

Exists in the Southern Research Station. Indicates which species are valid for Southern Research Station States. Tree species that are applicable to the Southern States are marked with an X.

18. SITETREE Sitetree. Indicates whether the tree species can be coded as a site tree. Tree species that are applicable to have site data collected are marked with an X.

19. SFTWD_HRDWD

Softwood/ hardwood. Indicates whether the species is a softwood or a hardwood. Softwoods are marked with an S and hardwoods with an H.

20. ST_EXISTS_IN_NCRS

Site tree exists in the North Central Research Station. Indicates whether or not the species is valid as a site tree in North Central Research Station States. Tree species that are applicable to have site data collected are marked with an X.

21. ST_EXISTS_IN_NERS

Site tree exists in the Northeastern Research Station. Indicates whether or not the species is valid as a site tree in Northeastern Research Station States. Tree species that are applicable to have site data collected are marked with an X.

22. ST_EXISTS_IN_PNWRS

Site tree exists in the Pacific Northwest Research Station. Indicates whether or not the species is valid for a site tree in Pacific Northwest Research Station States. Tree species that are applicable to have site data collected are marked with an X.

23. ST_EXISTS_IN RMRS

Site tree exists in the Rocky Mountain Research Station. Indicates whether or not the species is valid as a site tree in Rocky Mountain Research Station States. Tree species that are applicable to have site data collected are marked with an X.

24. ST_EXISTS_IN_SRS

Site tree exists in the Southern Research Station. Indicates whether or not the species is valid for a site tree in Southern Research Station States. Tree species that are applicable to have site data collected are marked with an X.

25. EAST East. Indicates if the species can occur in the Eastern United States. Valid eastern species are marked with an E.

26. WEST West. Indicates if the species can occur in the Western United States. Valid western species are marked with a W.

27. WOODLAND Woodland. Indicates if the species is classified as a woodland species, meaning that the diameter is measured as root collar. Woodland species are marked with an X.

28. MANUAL_START

Manual start. The first version of the Field Guide (PLOT.MANUAL) that the species code was used.

29. MANUAL_END

Manual end. The last version of the Field Guide (PLOT. MANUAL) that the species code was valid. When MANUAL_END is blank (null), the code is still valid.

30. CREATED_BY Created by. The employee who created the record. This attribute is intentionally left blank in download files.

31. CREATED_DATE

Created date. The date the record was created. Date will be in the form DD-MON-YYYY.

32. CREATED_IN_INSTANCE

Created in instance. The database instance in which the record was created. Each computer system has a unique database instance code and this attribute stores that information to determine on which computer the record was created.

33. MODIFIED_BY

Modified by. The employee who modified the record. This field will be blank (null) if the data have not been modified since initial creation. This attribute is intentionally left blank in download files.

34. MODIFIED_DATE

Modified date. The date the record was last modified. This field will be blank (null) if the data have not been modified since initial creation. Date will be in the form DD-MON-YYYY.

35. MODIFIED_IN_INSTANCE

Modified in instance. The database instance in which the record was modified. This field will be blank (null) if the data have not been modified since initial creation.

36. CORE — Core. Indicates that the tree species must be tallied (measured) by all FIA work units. Species marked with a Y are core and core optional species are marked with an N.

37. JENKINS_SPGRPCD

Jenkins species group code. A code that identifies a group of similar species, which is used to apply the correct biomass estimation equation and coefficient developed by Jenkins and others (2003). A specific set of biomass equation coefficients are assigned to each group. Additional explanation about how to estimate biomass, and when to use a certain set of coefficients, is provided in appendix J.

Code	Description
1	Cedar/larch
2	Douglas-fir
3	True fir/hemlock
4	Pine
5	Spruce
6	Aspen/alder/cottonwood/willow
7	Soft maple/birch
8	Mixed hardwood
9	Hard maple/oak/hickory/beech
10	Juniper/oak/mesquite

38. JENKINS_TOTAL_B1

Jenkins total B1. Jenkins B1 coefficient used to estimate total aboveground oven-dry biomass (pounds). This is coefficient B_0 from table 4 in Jenkins and others (2003). See appendix J for details on biomass equations.

Use JENKINS_TOTAL_B1 along with JENKINS_TOTAL_B2 to estimate total aboveground biomass (includes stem wood (bole), stump, bark, top, limbs, and foliage) with the equation below:

Total_agb = (Exp(JENKINS_TOTAL_B1 + JENKINS_TOTAL_B2 * ln(DIA*2.54)) * 2.2046)

JENKINS_SPGRPCD	JENKINS_TOTAL_B1
1	-2.03360
2	-2.23040
3	-2.53840
4	-2.53560
5	-2.07730
6	-2.20940
7	-1.91230
8	-2.48000
9	-2.01270
10	-0.71520

39. JENKINS_TOTAL_B2

Jenkins total B2. Jenkins B2 coefficient used to estimate total aboveground oven-dry biomass (pounds). This is coefficient B_1 from table 4 in Jenkins and others (2003). See appendix J for details on biomass equations.

Use JENKINS_TOTAL_B2 along with JENKINS_TOTAL_B1 to estimate total aboveground biomass (includes stem wood (bole), stump, bark, top, limbs, and foliage) with the equation below:

Total_agb = (Exp(JENKINS_TOTAL_B1 + JENKINS_TOTAL_B2 * ln(DIA*2.54)) * 2.2046)

JENKINS_SPGRPCD	JENKINS_TOTAL_B2
1	2.25920
2	2.44350
3	2.48140
4	2.43490
5	2.33230
6	2.38670
7	2.36510
8	2.48350
9	2.43420
10	1.70290

40. JENKINS_STEM_WOOD_RATIO_B1

Jenkins stem wood ratio B1. A coefficient used in computing component ratio biomass. This is equivalent to coefficient B_0 for stem wood from table 6 in Jenkins and others (2003). The appropriate coefficient to use is based on the species category (SFTWD_HRDWD). The stem is defined as that portion of the tree from a 1-foot stump to a 4-inch DOB top (i.e., the merchantable bole.) See appendix J for details on biomass equations.

The average proportion of aboveground biomass in stem wood is calculated using this equation:

stem_ratio = Exp(JENKINS_STEM_WOOD_RATIO_B1 + JENKINS_STEM_WOOD_RATIO_B2 / (DIA*2.54))

Species category	JENKINS_STEM_WOOD_RATIO_B1
Softwood (S)	-0.3737
Hardwood (H)	-0.3065

41. JENKINS_STEM_WOOD_RATIO_B2

Jenkins stem wood ratio B2. A coefficient used in computing component ratio biomass. This is equivalent to coefficient B_1 for stem wood from table 6 in Jenkins and others (2003). The appropriate coefficient to use is based on the species category (SFTWD_HRDWD). The stem is defined as that portion of the tree from a 1-foot stump to a 4-inch DOB top (i.e., the merchantable bole.) See appendix J for details on biomass equations.

The average proportion of aboveground biomass in stem wood is calculated using this equation:

stem_ratio = Exp(JENKINS_STEM_WOOD_RATIO_B1 + JENKINS_STEM_WOOD_RATIO_B2 / (DIA*2.54))

Species category	JENKINS_STEM_WOOD_RATIO_B2
Softwood (S)	-1.8055
Hardwood (H)	-5.4240

42. JENKINS_STEM_BARK_RATIO_B1

Jenkins stem bark ratio B1. A coefficient used in computing component ratio biomass. This is equivalent to coefficient B_0 for stem bark from table 6 in Jenkins and others (2003). The appropriate coefficient to use is based on the species category (SFTWD_HRDWD). This ratio estimates bark biomass on the stem, defined as that portion of the tree from a 1-foot stump to a 4-inch DOB top (i.e., the merchantable bole.) See appendix J for details on biomass equations.

The average proportion of aboveground biomass in stem bark is calculated using this equation:

bark_ratio = Exp(JENKINS_STEM_BARK_RATIO_B1 + JENKINS_STEM_BARK_RATIO_B2 / (DIA*2.54))

Species category	JENKINS_STEM_BARK_RATIO_B1
Softwood (S)	-2.0980
Hardwood (H)	-2.0129

43. JENKINS_STEM_BARK_RATIO_B2

Jenkins stem bark ratio B2. A coefficient used in computing component ratio biomass. This is equivalent to coefficient B_1 for stem bark from table 6 in Jenkins and others (2003). The appropriate coefficient to use is based on the species category (SFTWD_HRDWD). This ratio estimates bark biomass on the stem, defined as that portion of the tree from a 1-foot stump to a 4-inch DOB top (i.e., the merchantable bole.) See appendix J for details on biomass equations.

The average proportion of aboveground biomass in stem bark is calculated using this equation:

bark_ratio = Exp(JENKINS_STEM_BARK_RATIO_B1 + JENKINS_STEM_BARK_RATIO_B2 / (DIA*2.54))

Species category	JENKINS_STEM_BARK_RATIO_B2
Softwood (S)	-1.1432
Hardwood (H)	-1.6805

44. JENKINS_FOLIAGE_RATIO_B1

Jenkins foliage ratio B1. A coefficient used in computing component ratio biomass. This is equivalent to coefficient B_0 for foliage from table 6 in Jenkins and others (2003). The appropriate coefficient to use is based on the species category (SFTWD_HRDWD). See appendix J for details on biomass equations.

The average proportion of aboveground biomass in foliage is calculated using this equation:

foliage_ratio = Exp(JENKINS_FOLIAGE_RATIO_B1 + JENKINS_FOLIAGE_RATIO_B2 / (DIA*2.54))

Species category	JENKINS_FOLIAGE_RATIO_B1
Softwood (S)	-2.9584
Hardwood (H)	-4.0813

45. JENKINS_FOLIAGE_RATIO_B2

Jenkins foliage ratio B2. A coefficient used in computing component ratio biomass. This is equivalent to coefficient B_1 for foliage from table 6 in Jenkins and others (2003). The appropriate coefficient to use is based on the species category (SFTWD_HRDWD). See appendix J for details on biomass equations.

The average proportion of aboveground biomass in foliage is calculated using this equation:

foliage_ratio = Exp(JENKINS_FOLIAGE_RATIO_B1 + JENKINS_FOLIAGE_RATIO_B2 / (DIA*2.54)).

Species category	JENKINS_FOLIAGE_RATIO_B2
Softwood (S)	4.4766
Hardwood (H)	5.8816

46. JENKINS_ROOT_RATIO_B1

Jenkins root ratio B1. A coefficient used in computing component ratio biomass. This is equivalent to coefficient B_0 for coarse roots from table 6 in Jenkins and others (2003). The appropriate coefficient to use is based on the species category (SFTWD_HRDWD). See appendix J for details on biomass equations.

The average proportion of coarse roots to total aboveground biomass is calculated using this equation:

root_ratio = Exp(JENKINS_ROOT_RATIO_B1 + JENKINS_ROOT_RATIO_B2 / (DIA*2.54))

Species category	JENKINS_ROOT_RATIO_B1
Softwood (S)	-1.5619
Hardwood (H)	-1.6911

47. JENKINS_ROOT_RATIO_B2

Jenkins root ratio B2. A coefficient used in computing component ratio biomass. This is equivalent to coefficient B_1 for coarse roots from table 6 in Jenkins and others (2003). The appropriate coefficient to use is based on the

species category (SFTWD_HRDWD). See appendix J for details on biomass equations.

The average proportion of coarse roots to total aboveground biomass is calculated using this equation:

root_ratio = Exp(JENKINS_ROOT_RATIO_B1 + JENKINS_ROOT_RATIO_B2 / (DIA*2.54))

**Species category**	**JENKINS_ROOT_RATIO_B2**
Softwood (S)	0.6614
Hardwood (H)	0.8160

48. JENKINS_SAPLING_ADJUSTMENT

Jenkins sapling adjustment factor. A factor used to compute the biomass of saplings. Sapling biomass is computed by multiplying diameter (DIA) by the appropriate species adjustment factor (from Jenkins and others [2003]). The sapling adjustment factor was computed as a national average ratio of the REGIONAL_DRYBIOT (total dry biomass) divided by the Jenkins total biomass for all 5.0-inch trees, which is the size at which biomass based on volume begins. Because this adjustment factor was computed at the species level, there is a specific adjustment factor for each species. Users can download the REF_SPECIES table, which includes the values of JENKINS_SAPLING_ADJUSTMENT at http://ncrs2.fs.fed.us/fiadb4-downloads/datamart.html. See appendix J for details on biomass equations.

49. WOOD_SPGR_GREENVOL_DRYWT

Green specific gravity of wood (green volume and oven-dry weight). This variable is used to determine the oven-dry weight (in pounds) of live and dead trees based on volume variables in the TREE table (VOLCFSND, VOLCFGRS, VOLCFNET…). These volumes are assumed to be green wood volumes. Oven-dry biomass for the sound volume in a tree can be calculated using this equation:

B_{odw} = VOLCFSND x WOOD_SPGR_GREENVOL_DRYWT x 62.4

Where:

B_{odw} = sound oven-dry biomass of a tree in pounds

VOLCFSND = sound volume of a tree in cubic feet

50. WOOD_SPGR_GREENVOL_DRYWT_CIT

Citation for WOOD_SPGR_GREENVOL_DRYWT. The value of this variable can be linked to the corresponding value in the CITATION_NBR variable in the REF_CITATION table to find the source of the WOOD_SPGR_GREENVOL_DRYWT variable.

51. BARK_SPGR_GREENVOL_DRYWT

Green specific gravity of the bark (green volume and oven-dry weight). There is some shrinkage in bark volume when a live tree is cut and dried. In FIADB, this specific gravity is used on live and dead trees to convert green volume to oven-dry weight in pounds. Oven-dry biomass for bark can be calculated using the volume of a tree using this equation:

B_{odw} = BARK_VOLUME x BARK_SPGR_GREENVOL_DRYWT x 62.4

Where:

B_{odw} = oven-dry biomass of bark on a tree in pounds

BARK_VOLUME = volume of the bark on a tree bole, in cubic feet. Note that bark volume is often estimated by subtracting volume of the bole inside bark from volume of the bole outside bark. Or, an estimate of bark volume can be obtained using any tree volume column along with BARK_VOL_PCT found in this table as follows:

BARK_VOLUME = TREE_VOLUME * (BARK_VOL_PCT/100.0)

52. BARK_SPGR_GREENVOL_DRYWT_CIT

Citation for BARK_SPGR_GREENVOL_DRYWT. The value of this variable can be linked to the corresponding value in the CITATION_NBR variable in the REF_CITATION table to find the source of the BARK_SPGR_GREENVOL_DRYWT variable.

53. MC_PCT_GREEN_WOOD

Moisture content of green wood as a percent of oven-dry weight. Wood and bark are often sold based on green weight. The user is cautioned that green weights can be extremely variable geographically, seasonally, within species and across various portions of individual trees.

54. MC_PCT_GREEN_WOOD_CIT

Citation for MC_PCT_GREEN_WOOD_CIT. The value of this variable can be linked to the corresponding value in the CITATION_NBR variable in the REF_CITATION table to find the source of the MC_PT_GREEN_WOOD variable.

55. MC_PCT_GREEN_BARK

Moisture content of green bark as a percent of oven-dry weight. Wood and bark are often sold based on green weight. The user is cautioned that green weights can be extremely variable geographically, seasonally, within species and across various portions of individual trees.

56. MC_PCT_GREEN_BARK_CIT

Citation for MC_PCT_GREEN_BARK. The value of this variable can be linked to the corresponding value in the CITATION_NBR variable in the REF_CITATION table to find the source of the MC_PCT_GREEN_BARK variable.

57. WOOD_SPGR_MC12VOL_DRYWT

Wood specific gravity (12 percent moisture content volume and oven-dry weight). Used in biomass estimation of forest products (lumber, veneer, etc.)

58. WOOD_SPGR_MC12VOL_DRYWT_CIT

Citation for WOOD_SPGR_MC12VOL_DRYWT. The value of this variable can be linked to the corresponding value in the CITATION_NBR variable in the REF_CITATION table to find the source of the WOOD_SPGR_MC12VOL_DRYWT variable.

59. BARK_VOL_PCT

Bark volume as a percent of wood volume. Bark volume expressed as a percent of wood volume. The volume of bark does not include voids due to ridges and valleys in bark.

60. BARK_VOL_PCT_CIT

Citation for BARK_VOL_PCT. The value of this variable can be linked to the corresponding value in the CITATION_NBR variable in the REF_CITATION table to find the source of the BARK_VOL_PCT variable.

61. RAILE_STUMP_DOB_B1

Raile stump diameter outside bark equation coefficient B1. This is equivalent to coefficient B from table 1 in Raile (1982). See appendix J for details on biomass equations.

This coefficient is used in an equation to estimate diameter outside bark at any point on the stump from ground to 1 foot high. From this, volume outside bark is estimated for the selected height along the stump. Volume inside bark is subtracted from volume outside bark to estimate bark volume. Both volumes are converted to biomass using either wood or bark specific gravities. (DOB and DIA are in inches, HT is in feet.)

DOB = DIA + (DIA * RAILE_STUMP_DOB_B1 * (4.5-HT) / (HT+1))

62. RAILE_STUMP_DIB_B1

Raile stump diameter inside bark equation coefficient B1. This is equivalent to coefficient A from table 2 in Raile (1982). See appendix J for details on biomass equations.

This coefficient is used along with RAILE_STUMP_DIB_B2 in an equation to estimate diameter inside bark at any point on the stump from ground to 1 foot high. From this, volume inside bark is estimated for the selected height along the stump. Volume inside bark is subtracted from volume outside bark to estimate bark volume. Both volumes are converted to biomass using either wood or bark specific gravities. (DIB and DIA are in inches, HT is in feet.)

DIB = (DIA * RAILE_STUMP_DIB_B1) +

(DIA * RAILE_STUMP_DIB_B2 * (4.5-HT) / (HT+1))

63. RAILE_STUMP_DIB_B2

Raile stump diameter inside bark equation coefficient B2. This is equivalent to coefficient B from table 2 in Raile (1982). See appendix J for details on biomass equations.

This coefficient is used along with RAILE_STUMP_DIB_B1 in an equation to estimate diameter inside bark at any point on the stump from ground to 1 foot high. From this, volume inside bark is estimated for the selected height along the stump. Volume inside bark is subtracted from volume outside bark to estimate bark volume. Both volumes are converted to biomass using either wood or bark specific gravities. (DIB and DIA are in inches, HT is in feet.)

DIB = (DIA * RAILE_STUMP_DIB_B1) +

(DIA * RAILE_STUMP_DIB_B2 * (4.5-HT) / (HT+1))

Reference Species Group Table (Oracle table name is REF_SPECIES_GROUP)

	Column name	Descriptive name	Oracle data type
1	SPGRPCD	Species group code	NUMBER(2)
2	NAME	Name	VARCHAR2(35)
3	REGION	Region	VARCHAR2(8)
4	CLASS	Class	VARCHAR2(8)
5	CREATED_BY	Created by	VARCHAR2(30)
6	CREATED_DATE	Created date	DATE
7	CREATED_IN_INSTANCE	Created in instance	VARCHAR2(6)
8	MODIFIED_BY	Modified by	VARCHAR2(30)
9	MODIFIED_DATE	Modified date	DATE
10	MODIFIED_IN_INSTANCE	Modified in instance	VARCHAR2(6)

Type of key	Column(s) order	Tables to link	Abbreviated notation
Primary	(SPGRPCD)	N/A	SGP_PK

1. SPGRPCD Species group code. A code assigned to each tree species in order to group them for reporting purposes on presentation tables. Codes and their associated names (NAME) are shown in appendix G. Individual tree species and corresponding species group codes are shown in appendix F.

2. NAME Name. A descriptive name for each species group code (SPGRPCD). The names associated with these codes are used to label rows or columns in national standard presentation tables.

3. REGION Region. A description of the section of the United States in which the species, and therefore species group is commonly found. Values are 'EASTERN' and 'WESTERN.'

4. CLASS Class. A descriptor for the classification of the species type with the species group. Values are 'SOFTWOOD' and 'HARDWOOD.'

5. CREATED_BY Created by. The employee who created the record. This attribute is intentionally left blank in download files.

6. CREATED_DATE

   Created date. The date the record was created. Date will be in the form DD-MON-YYYY.

7. CREATED_IN_INSTANCE

   Created in instance. The database instance in which the record was created. Each computer system has a unique database instance code and this attribute stores that information to determine on which computer the record was created.

8. MODIFIED_BY

Modified by. The employee who modified the record. This field will be blank (null) if the data have not been modified since initial creation. This attribute is intentionally left blank in download files.

9. MODIFIED_DATE

Modified date. The date the record was last modified. This field will be blank (null) if the data have not been modified since initial creation. Date will be in the form DD-MON-YYYY.

10. MODIFIED_IN_INSTANCE

Modified in instance. The database instance in which the record was modified. This field will be blank (null) if the data have not been modified since initial creation.

Reference Habitat Type Description Table (Oracle table name is REF_HABTYP_DESCRIPTION)

	Column name	Descriptive name	Oracle data type
1	CN	Sequence number	VARCHAR2(34)
2	HABTYPCD	Habitat type code	VARCHAR2(10)
3	PUB_CD	Publication code	VARCHAR2(10)
4	SCIENTIFIC_NAME	Scientific name	VARCHAR2(115)
5	COMMON_NAME	Common name	VARCHAR2(255)
6	VALID	Valid	VARCHAR2(1)
7	CREATED_BY	Created by	VARCHAR2(30)
8	CREATED_DATE	Created date	DATE
9	CREATED_IN_INSTANCE	Created in instance	VARCHAR2(6)
10	MODIFIED_BY	Modified by	VARCHAR2(30)
11	MODIFIED_DATE	Modified date	DATE
12	MODIFIED_IN_INSTANCE	Modified in instance	VARCHAR2(6)

Type of key	Column(s) order	Tables to link	Abbreviated notation
Primary	(CN)	N/A	RHN_PK
Unique	(HABTYPCD, PUB_CD)	N/A	RHN_UK
Foreign	(PUB_CD)	REF_HABTYP_DESCRIPTION to REF_HABTYP_PUBLICATION	RHN_RPN_FK

1. CN — Sequence number. A unique sequence number used to identify a habitat type description record.

2. HABTYPCD — Habitat type code. A code representing a habitat type. Unique codes are determined by combining both habitat type code and publication code (HABTYPCD and PUB_CD).

3. PUB_CD — Publication code. A code indicating the publication that lists the name associated with a particular habitat type code (HABTYPCD).

4. SCIENTIFIC_NAME

   Scientific name. This attribute contains some type of descriptor, usually the Latin name, of the plant(s) associated with the habitat type code. It has values such as the entire scientific name or the shortened synonym of the plant(s) represented by the habitat type code or it may have an English geographic type of descriptor.

5. COMMON_NAME

   Common name. This attribute contains some type of descriptor, usually the common name, of the plant(s) associated with the habitat type code.

6. VALID — Valid. A flag to indicate if this is a valid, documented habitat type code. Values are Y and N.

7. CREATED_BY — Created by. The employee who created the record. This attribute is intentionally left blank in download files.

8. CREATED_DATE

   Created date. The date the record was created. Date will be in the form DD-MON-YYYY.

9. CREATED_IN_INSTANCE

   Created in instance. The database instance in which the record was created. Each computer system has a unique database instance code and this attribute stores that information to determine on which computer the record was created.

10. MODIFIED_BY

    Modified by. The employee who modified the record. This field will be blank (null) if the data have not been modified since initial creation. This attribute is intentionally left blank in download files.

11. MODIFIED_DATE

    Modified date. The date the record was last modified. This field will be blank (null) if the data have not been modified since initial creation. Date will be in the form DD-MON-YYYY.

12. MODIFIED_IN_INSTANCE

    Modified in instance. The database instance in which the record was modified. This field will be blank (null) if the data have not been modified since initial creation.

Reference Habitat Type Publication Table (Oracle table name is REF_HABTYP_PUBLICATION)

	Column name	Descriptive name	Oracle data type
1	CN	Sequence number	VARCHAR2(34)
2	PUB_CD	Publication code	VARCHAR2(10)
3	TITLE	Title of publication	VARCHAR2(200)
4	AUTHOR	Author of publication	VARCHAR2(200)
5	TYPE	Type of publication	VARCHAR2(10)
6	VALID	Valid	VARCHAR2(1)
7	CREATED_BY	Created by	VARCHAR2(30)
8	CREATED_DATE	Created date	DATE
9	CREATED_IN_INSTANCE	Created in instance	VARCHAR2(6)
10	MODIFIED_BY	Modified by	VARCHAR2(30)
11	MODIFIED_DATE	Modified date	DATE
12	MODIFIED_IN_INSTANCE	Modified in instance	VARCHAR2(6)

Type of key	Column(s) order	Tables to link	Abbreviated notation
Primary	(CN)	N/A	RPN_PK
Unique	(PUB_CD)	N/A	RPN_UK

1. CN — Sequence number. A unique sequence number used to identify a habitat type publication record.

2. PUB_CD — Publication code. A code indicating the publication that lists the name associated with a particular habitat type code (REF_HABTYP_DESCRIPTION.HABTYPCD).

3. TITLE — Title. The title of the publication defining particular habitat types.

4. AUTHOR — Author. The author of the publication defining particular habitat types.

5. TYPE — Type. An attribute describing if the habitat type publication describes potential vegetation or existing vegetation. Values are PVREF and EVREF. If it is unknown which type of habitat is being described, then TYPE = ?.

6. VALID — Valid. A flag to indicate if this publication is valid for FIA. Values are Y and N.

7. CREATED_BY — Created by. The employee who created the record. This attribute is intentionally left blank in download files.

8. CREATED_DATE

   Created date. The date the record was created. Date will be in the form DD-MON-YYYY.

9. CREATED_IN_INSTANCE

Created in instance. The database instance in which the record was created. Each computer system has a unique database instance code and this attribute stores that information to determine on which computer the record was created.

10. MODIFIED_BY

Modified by. The employee who modified the record. This field will be blank (null) if the data have not been modified since initial creation. This attribute is intentionally left blank in download files.

11. MODIFIED_DATE

Modified date. The date the record was last modified. This field will be blank (null) if the data have not been modified since initial creation. Date will be in the form DD-MON-YYYY.

12. MODIFIED_IN_INSTANCE

Modified in instance. The database instance in which the record was modified. This field will be blank (null) if the data have not been modified since initial creation.

Reference Citation Table (Oracle table name is REF_CITATION)

	Column name	Descriptive name	Oracle data type
1	CITATION_NBR	Citation number	NUMBER(7)
2	CITATION	Citation	VARCHAR2(2000)
3	CREATED_BY	Created by	VARCHAR2(30)
4	CREATED_DATE	Created date	DATE
5	CREATED_IN_INSTANCE	Created in instance	VARCHAR2(6)
6	MODIFIED_BY	Modified by	VARCHAR2(30)
7	MODIFIED_DATE	Modified date	DATE
8	MODIFIED_IN_INSTANCE	Modified in instance	VARCHAR2(6)

Type of key	Column(s) order	Tables to link	Abbreviated notation
Primary	(CITATION_NBR)	N/A	CIT_PK

1. CITATION_NBR

   Citation number. A unique number used to identify a REF_CITATION record. Citation information is currently available in the database only for information about the source of specific gravity and bark volume percent values contained in the REF_SPECIES table. REF_SPECIES variables ending in "_CIT" link back to the REF_CITATION table through CITATION_NBR.

2. CITATION Citation. This attribute is usually a publication citation. In some cases CITATION may contain more specific information about how data were populated for a field.

3. CREATED_BY Created by. The employee who created the record. This attribute is intentionally left blank in download files.

4. CREATED_DATE

   Created date. The date the record was created. Date will be in the form DD-MON-YYYY.

5. CREATED_IN_INSTANCE

   Created in instance. The database instance in which the record was created. Each computer system has a unique database instance code and this attribute stores that information to determine on which computer the record was created.

6. MODIFIED_BY

Modified by. The employee who modified the record. This field will be blank (null) if the data have not been modified since initial creation. This attribute is intentionally left blank in download files.

7. MODIFIED_DATE

Modified date. The date the record was last modified. This field will be blank (null) if the data have not been modified since initial creation. Date will be in the form DD-MON-YYYY.

8. MODIFIED_IN_INSTANCE

Modified in instance. The database instance in which the record was modified. This field will be blank (null) if the data have not been modified since initial creation.

Reference Forest Inventory and Analysis Database Version Table (Oracle table name is REF_FIADB_VERSION)

	Column name	Descriptive name	Oracle data type
1	VERSION	Version number	NUMBER(3,1)
2	DESCR	Version description	VARCHAR2(2000)
3	CREATED_BY	Created by	VARCHAR2(30)
4	CREATED_DATE	Created date	DATE
5	CREATED_IN_INSTANCE	Created in instance	VARCHAR2(6)
6	MODIFIED_BY	Modified by	VARCHAR2(30)
7	MODIFIED_DATE	Modified date	DATE
8	MODIFIED_IN_INSTANCE	Modified in instance	VARCHAR2(6)
9	INSTALL_TYPE	Install type	VARCHAR2(10)

Type of key	Column(s) order	Tables to link	Abbreviated notation
Primary	(VERSION)	N/A	RFN_PK

1. VERSION Version number. A unique number used to identify a REF_FIADB_VERSION record. VERSION equals the currently available version of the FIADB.

2. DESCR Version description. A description of the FIADB version. This may include a literature citation and internet links to documentation.

3. CREATED_BY Created by. The employee who created the record. This attribute is intentionally left blank in download files.

4. CREATED_DATE

   Created date. The date the record was created. Date will be in the form DD-MON-YYYY.

5. CREATED_IN_INSTANCE

   Created in instance. The database instance in which the record was created. Each computer system has a unique database instance code and this attribute stores that information to determine on which computer the record was created.

6. MODIFIED_BY

   Modified by. The employee who modified the record. This field will be blank (null) if the data have not been modified since initial creation. This attribute is intentionally left blank in download files.

7. MODIFIED_DATE

Modified date. The date the record was last modified. This field will be blank (null) if the data have not been modified since initial creation. Date will be in the form DD-MON-YYYY.

8. MODIFIED_IN_INSTANCE

Modified in instance. The database instance in which the record was modified. This field will be blank (null) if the data have not been modified since initial creation.

9. INSTALL_TYPE

Install type. Intentionally left blank. Will be populated in version 5.0.

Reference State Elevation Table (Oracle table name is REF_STATE_ELEV)

	Column name	Descriptive name	Oracle data type
1	STATECD	State code	NUMBER(4)
2	MIN_ELEV	Minimum elevation	NUMBER(5)
3	MAX_ELEV	Maximum elevation	NUMBER(5)
4	LOWEST_POINT	Lowest point	VARCHAR2(30)
5	HIGHEST_POINT	Highest point	VARCHAR2(30)
6	CREATED_BY	Created by	VARCHAR2(30)
7	CREATED_DATE	Created date	DATE
8	CREATED_IN_INSTANCE	Created in instance	VARCHAR26)
9	MODIFIED_BY	Modified by	VARCHAR2(30)
10	MODIFIED_DATE	Modified date	DATE
11	MODIFIED_IN_INSTANCE	Modified in instance	VARCHAR2(6)

Type of key	Column(s) order	Tables to link	Abbreviated notation
Primary	(STATECD)	N/A	RSE_PK

1. STATECD State code. Bureau of the Census Federal Information Processing Standards (FIPS) two-digit code for each State. Refer to appendix C.

2. MIN_ELEV Minimum elevation. The minimum elevation within the State in feet.

3. MAX_ELEV Maximum elevation. The maximum elevation within the State in feet.

4. LOWEST_POINT

   Lowest point. The name of the lowest point within the State. 'SL' refers to sea level. Negative minimum elevations are listed here.

5. HIGHEST_POINT

   Highest point. The name of the highest point within the State. Alternative names are provided also.

6. CREATED_BY Created by. The employee who created the record. This attribute is intentionally left blank in download files.

7. CREATED_DATE

   Created date. The date the record was created. Date will be in the form DD-MON-YYYY.

8. CREATED_IN_INSTANCE

Created in instance. The database instance in which the record was created. Each computer system has a unique database instance code and this attribute stores that information to determine on which computer the record was created.

9. MODIFIED_BY

Modified by. The employee who modified the record. This field will be blank (null) if the data have not been modified since initial creation. This attribute is intentionally left blank in download files.

10. MODIFIED_DATE

Modified date. The date the record was last modified. This field will be blank (null) if the data have not been modified since initial creation. Date will be in the form DD-MON-YYYY.

11. MODIFIED_IN_INSTANCE

Modified in instance. The database instance in which the record was modified. This field will be blank (null) if the data have not been modified since initial creation.

Reference Unit Table (Oracle table name is REF_UNIT)

	Column name	Descriptive name	Oracle data type
1	STATECD	State code	NUMBER(4)
2	VALUE	Value	NUMBER(2)
3	MEANING	Meaning	VARCHAR2(80)
4	CREATED_BY	Created by	VARCHAR2(30)
5	CREATED_DATE	Created date	DATE
6	CREATED_IN_INSTANCE	Created in instance	VARCHAR2(6)
7	MODIFIED_BY	Modified by	VARCHAR2(30)
8	MODIFIED_DATE	Modified date	DATE
9	MODIFIED_IN_INSTANCE	Modified in instance	VARCHAR2(6)

Type of key	Column(s) order	Tables to link	Abbreviated notation
Primary	(STATECD, VALUE)	N/A	UNT_PK

1. STATECD State code. Bureau of the Census Federal Information Processing Standards (FIPS) two-digit code for each State. Refer to appendix C.

2. VALUE Value. Forest Inventory and Analysis survey unit identification number. Survey units are usually groups of counties within each State. For periodic inventories, survey units may be made up of lands of particular owners. Refer to appendix C for codes.

3. MEANING Meaning. The name corresponding to the survey unit code (VALUE) in the State (STATECD). Refer to appendix C.

4. CREATED_BY Created by. The employee who created the record. This attribute is intentionally left blank in download files.

5. CREATED_DATE

   Created date. The date the record was created. Date will be in the form DD-MON-YYYY.

6. CREATED_IN_INSTANCE

   Created in instance. The database instance in which the record was created. Each computer system has a unique database instance code and this attribute stores that information to determine on which computer the record was created.

7. MODIFIED_BY

   Modified by. The employee who modified the record. This field will be blank (null) if the data have not been modified since initial creation. This attribute is intentionally left blank in download files.

8. MODIFIED_DATE

Modified date. The date the record was last modified. This field will be blank (null) if the data have not been modified since initial creation. Date will be in the form DD-MON-YYYY.

9. MODIFIED_IN_INSTANCE

Modified in instance. The database instance in which the record was modified. This field will be blank (null) if the data have not been modified since initial creation.

Chapter 4 – Calculating Population Estimates and Their Associated Sampling Errors

This chapter presents procedures written in Oracle™ SQL script that can be used to obtain population estimates (and associated sampling errors) for standard FIA attributes from the measurement data stored in the FIADB. These estimates follow the equations presented in Bechtold and Patterson (2005, chapter 4). Population estimates for many attributes can be generated using either the web-based EVALIDator tool or the Forest Inventory Data Online (FIDO) tool, which provides interactive access to the FIADB. These tools can be found at http://fia.fs.fed.us/tools-data.

All data stored in FIADB can be downloaded from http://fia.fs.fed.us/tools-data as either comma delimited files or Microsoft (MS) Access databases. Because of size limitations, data are stored in individual State databases. The SQL scripts used with MS Access differ from Oracle™ SQL scripts described in this chapter; however a number of MS Access queries are provided in the MS Access databases. All of the FIADB 4.0 tables are included in both formats. The MS Access databases have a few additional tables that make using the data and constructing queries easier and simpler. In addition, numerous queries that produce population estimates and standard errors are provided. Users can use these queries as a starting point to create customized queries suitable for local or regional analyses.

The FIADB can be used to estimate many attributes (e.g., forest area, timberland area, number of trees, net volume, biomass) from many different samples (typically State-wide inventories for a specific year or set of years). Therefore, the number of estimates that can be made from the FIADB is very large, and continues to increase as more data are added to the FIADB. This chapter provides examples of a few estimation procedures that can be modified by the user. The resulting estimates shown as output are examples only and are not necessarily the exact numbers a user will obtain using current data.

In addition to the naming conventions used in the FIADB, reference is made to the notation and terminology used in Bechtold and Patterson (2005). To fully understand the statistical basis of the estimation, readers may find it useful to refer to that publication as they review this chapter. Examples that estimate area of timberland, number of live trees on forest land, number of seedlings on timberland, and volume of growing-stock on timberland are presented, along with discussion of how these examples can be modified to estimate other attributes measured in Phase 2.

The basic estimation is broken down into four steps, with additional steps for users who want to go beyond the traditional population level estimates.

1. Selecting the attribute of interest (the quantity that is to be estimated).
2. Selecting an appropriate sample.
3. Linking the appropriate tables in the FIADB to produce estimates for attributes of interest for a population.
4. Producing estimates with sampling errors for attributes of interest for a population.
5. Restricting the attribute of interest to a smaller subset of the population (e.g., filtering the data to include only sawtimber stands on publicly owned timberland, versus all stands in all ownerships).
6. Changing the attribute of interest with user-defined criteria.
7. Estimating change over time on the standard 4-subplot fixed area plot.

1. Selecting the attribute of interest (using the REF_POP_ATTRIBUTE table)

The most common attributes of interest in FIADB estimation are described in the REF_POP_ATTRIBUTE table, which currently contains 92 entries. Attributes are currently defined at three levels (1) condition level attributes for area estimates; (2) tree level attributes for numbers of trees, volume, growth, removals, and mortality estimates; and (3) seedling level attributes for number of seedlings estimates. Estimation of condition level attributes requires accessing data on the PLOT and COND tables. Estimation of tree level attributes requires accessing data on the PLOT, COND, and TREE tables. Estimation of seedling level attributes requires accessing data on the PLOT, COND, and SEEDLING tables. Table 4.1 lists the attributes currently defined in the REF_POP_ATTRIBUTE table.

Table 4.1. Values and Descriptions in the REF_POP_ATTRIBUTE table.

Attribute number (ATTRIBUTE_NBR)	Attribute description (ATTRIBUTE_DESCR)
1	Area sampled and denied access/hazardous (acres)
2	Area of forestland (acres)
3	Area of timberland (acres)
4	Number of all live trees on forestland (trees)
5	Number of growing-stock trees on forestland (trees)
6	Number of standing dead trees 5 inches+ dbh on forestland (trees)
7	Number of all live trees on timberland (trees)
8	Number of growing-stock trees on timberland (trees)
9	Number of standing dead trees 5 inches+ dbh on timberland (trees)
10	All live tree and sapling aboveground biomass on forestland oven-dry (short tons)
11	All live merchantable biomass on forestland oven-dry (short tons)
12	All live merchantable biomass on timberland oven-dry (short tons)
13	All live tree and sapling aboveground biomass on timberland oven-dry (short tons)
14	Volume of all live on forestland (cuft)
15	Volume of growing-stock on forestland (cuft)
16	Volume of sawlog portion on forestland (cuft)
17	Volume of all live on timberland (cuft)
18	Volume of growing-stock on timberland (cuft)
19	Volume of sawlog portion on timberland (cuft)
20	Volume of sawtimber on forestland (bdft)
21	Volume of sawtimber on timberland (bdft)
22	All live gross sawtimber volume on forestland (bdft)
23	All live gross volume on forestland (cuft)
24	All live sound volume on forestland (cuft)
25	Net growth of all live on forestland (cuft per year)
26	Net growth of growing-stock on forestland (cuft per year)
27	Net growth of sawtimber on forestland (bdft per year)
28	Net growth of all live on timberland (cuft per year)
29	Net growth of growing-stock on timberland (cuft per year)
30	Net growth of sawtimber on timberland (bdft per year)
31	Mortality of all live on forestland (cuft per year)
32	Mortality of all live trees on forestland (trees per year)
33	Mortality of growing-stock on forestland (cuft per year)
34	Mortality of sawtimber on forestland (bdft per year)
35	Mortality of all live on timberland (cuft per year)
36	Mortality of all live trees on timberland (trees per year)
37	Mortality of growing-stock on timberland (cuft per year)
38	Mortality of sawtimber on timberland (bdft per year)
39	Removals of all live on forestland (cuft per year)

Attribute number (ATTRIBUTE_NBR)	Attribute description (ATTRIBUTE_DESCR)
40	Removals of growing-stock on forestland (cuft per year)
41	Removals of sawtimber on forestland (bdft per year)
42	Removals of all live on timberland (cuft per year)
43	Removals of growing-stock on timberland (cuft per year)
44	Removals of sawtimber on timberland (bdft per year)
45	Number of live seedlings on forestland (seedlings)
46	Number of live seedlings on timberland (seedlings)
47	Carbon in standing dead trees on forestland (short tons)
48	Carbon in understory aboveground on forestland (short tons)
49	Carbon in understory belowground on forestland (short tons)
50	Carbon in down dead on forestland (short tons)
51	Carbon in litter on forestland (short tons)
52	Soil organic carbon on forestland (short tons)
53	Carbon in live trees and saplings aboveground on forestland (short tons)
54	Carbon in live trees and saplings belowground on forestland (short tons)
55	Carbon in live trees and saplings above and belowground on forestland (short tons)
56	All live top and limb biomass on forestland oven-dry (short tons)
57	All live sapling biomass on forestland oven-dry (short tons)
58	All live stump (ground to 12 inches) biomass on forestland oven-dry (short tons)
59	All live belowground tree and sapling and woodland species biomass on forestland oven-dry (short tons)
60	All live woodland species biomass on forestland oven-dry (short tons)
61	Carbon in standing dead trees on timberland (short tons)
62	Carbon in understory aboveground on timberland (short tons)
63	Carbon in understory belowground on timberland (short tons)
64	Carbon in down dead on timberland (short tons)
65	Carbon in litter on timberland (short tons)
66	Soil organic carbon on timberland (short tons)
67	Carbon in live trees and saplings aboveground on timberland (short tons)
68	Carbon in live trees belowground on timberland (short tons)
69	Carbon in live trees above and belowground on timberland (short tons)
70	All live top and limb biomass on timberland oven-dry (short tons)
71	All live sapling biomass on timberland oven-dry (short tons)
72	All live stump (ground to 12 inches) biomass on timberland oven-dry (short tons)
73	All live belowground tree and sapling and woodland species biomass on timberland oven-dry (short tons)
74	All live woodland species biomass on timberland oven-dry (short tons)
75	Old regional method – All live tree and sapling aboveground biomass on forestland oven-dry (short tons)
76	Old regional method – All live merchantable biomass on forestland oven-dry (short tons)
77	Old regional method – All live merchantable biomass on timberland oven-dry (short tons)
78	Old regional method – All live tree and sapling aboveground biomass on timberland oven-dry (short tons)
79	Area sampled (acres)
80	Harvest removals of all live on forestland (cuft per year)
81	Harvest removals of growing-stock on forestland (cuft per year)
82	Harvest removals of sawtimber on forestland (bdft per year)
83	Harvest removals of all live on timberland (cuft per year)
84	Harvest removals of growing-stock on timberland (cuft per year)
85	Harvest removals of sawtimber on timberland (bdft per year)
86	Other removals of all live on forestland (cuft per year)

Attribute number (ATTRIBUTE_NBR)	Attribute description (ATTRIBUTE_DESCR)
87	Other removals of growing-stock on forestland (cuft per year)
88	Other removals of sawtimber on forestland (bdft per year)
89	Other removals of all live on timberland (cuft per year)
90	Other removals of growing-stock on timberland (cuft per year)
91	Other removals of sawtimber on timberland (bdft per year)
92	Volume of standing dead trees on forestland (cuft)

In this chapter we present examples that estimate:

- Area of timberland (REF_POP_ATTRIBUTE.ATTRIBUTE_NBR = 3).
- Number of live trees on forest land (REF_POP_ATTRIBUTE.ATTRIBUTE_NBR = 4).
- Volume of growing-stock on timberland (REF_POP_ATTRIBUTE.ATTRIBUTE_NBR = 18.
- Number of live seedlings on timberland (REF_POP_ATTRIBUTE.ATTRIBUTE_NBR = 46).

These are examples of condition, tree, and seedling level attributes that can be modified to produce other estimates of attributes at these levels. For each attribute, the REF_POP_ATTRIBUTE table contains a unique ATTRIBUTE_NBR, a description of the attribute (ATTRIBUTE_DESCR), and the variables EXPRESSION and WHERE_CLAUSE that are both portions of the SQL statements used to produce the estimates of the attribute. Table 4.2 lists these four variables for the four examples we are presenting. (Note: in EXPRESSION and WHERE_CLAUSE, ‘c’ stands for COND table, ‘t’ stands for TREE table, ‘s’ stands for SEEDLING table, and ‘pet’ stands for POP_EVAL_TYP table.)

Table 4.2. REF_POP_ATTRIBUTE entries for the four examples presented in this chapter.

ATTRIBUTE NBR	ATTRIBUTE DESCR	EXPRESSION [a]	WHERE CLAUSE
3	Area of timberland (acres)	c.condprop_unadj* decode(c.prop_basis,'MACR',pop_stratum.adj_factor_macr, pop_stratum.adj_factor_subp)	and pet.eval_typ=‘EXPCURR’ and c.cond_status_cd=1 and c.reservcd=0 and c.siteclcd in (1,2,3,4,5,6)
4	Number of all live trees on forestland (trees)	t.tpa_unadj* decode(dia,null,adj_factor_subp, decode(least(dia,5-0.001),dia,adj_factor_micr, decode(least(dia, nvl(macro_breakpoint_dia,9999)-0.001),dia,adj_factor_subp, adj_factor_macr)))	and pet.eval_typ=‘EXPVOL’ and t.plt_cn=c.plt_cn and t.condid=c.condid and c.cond_status_cd=1 and t.statuscd=1 and t.dia>=1.0
18	Volume of growing-stock on timberland (cuft)	t.tpa_unadj* t.volcfnet* decode(dia,null,adj_factor_subp, decode(least(dia,5-0.001),dia,adj_factor_micr, decode(least(dia, nvl(macro_breakpoint_dia,9999)-0.001),dia,adj_factor_subp, adj_factor_macr)))	and pet.eval_typ='EXPVOL' and t.plt_cn=c.plt_cn and t.condid=c.condid and c.cond_status_cd=1 and c.reservcd=0 and c.siteclcd in (1,2,3,4,5,6) and t.statuscd=1 and t.treeclcd=2 and t.dia>=5.0
46	Number of live seedlings on timberland (seedlings)	s.tpa_unadj*adj_factor_micr	and pet.eval_typ=‘EXPVOL’ and s.plt_cn=c.plt_cn and s.condid=c.condid and c.cond_status_cd=1 and c.reservcd=0 and c.siteclcd in (1,2,3,4,5,6)

[a] Note that for Microsoft Access SQL, the decode function is replaced with the IIF function

EXPRESSION is multiplied by the expansion factor POP_STRATUM.EXPNS and summed at the condition level in the estimation procedure. In the notation used in Bechtold and Patterson (2005), this sum is P_{hid} for area estimation (see equation 4.1, page 47) or y_{hid} for the estimation of tree attributes (see equation 4.8, page 53). In all cases, EXPRESSION consists of the product of two terms, the first term (c.condprop_unadj, t.tpa_unadj, and s.tpa_unadj in our examples) is the unadjusted observation of the attribute of interest (on a per acre basis). The second term is the appropriate stratum adjustment factor. The stratum adjustment factor is the inverse of the mean proportion of the sample plot areas that were within the population. Following the notation of Bechtold and Patterson (2005) this adjustment factor is $1/p_{mh}$ (see equation 4.2, page 49). The decode statement simply selects the appropriate adjustment factor to be used for the specific estimate. Area estimates use either ADJ_FACTOR_MACR (in inventories where area estimates are based on the macroplot) or ADJ_FACTOR_SUBP (in inventories where area estimates are based on the subplot) for the adjustment. The adjustment of tree- and seedling-level estimates is based on the plot on which the tree or seedling was sampled (seedlings and trees <5 inches diameter are sampled on the microplot, larger trees are sampled on the subplot or macroplot depending on diameter).

Common selection criteria used often with FIA data when creating queries include various classifications of land and groups of trees as shown below:

Identifying land classes (COND table):

Forest land	COND_STATUS_CD = 1
Timberland	COND_STATUS_CD = 1, SITECLCD <7, RESERVCD = 0
Nonforest land	COND_STATUS_CD = 2
Reserved forest land	COND_STATUS_CD = 1, RESERVCD = 1
Unreserved forest land	COND_STATUS_CD = 1, RESERVCD = 0
Productive forest land	COND_STATUS_CD = 1, SITECLCD <7
Unproductive forest land	COND_STATUS_CD = 1, SITECLCD = 7

Identifying tree characteristics:

Live trees	TREE.STATUSCD = 1
Standing dead trees	TREE.STATUSCD = 2, TREE.STANDING_DEAD_CD = 1
Growing-stock trees	TREE.STATUSCD = 1, TREE.TREECLCD = 2
Growing-stock volume	TREE.STATUSCD = 1, TREE.TREECLCD = 2, TREE.DIA ≥5.0

2. Selecting an appropriate sample (using the POP_EVAL_GRP, POP_EVAL, and POP_EVAL_TYP tables)

In order to compute a sample-based population estimate, the appropriate sample and stratification must be identified. In FIA estimation, the sample is a set of plots that were selected for the attribute of interest that was observed. The stratification consists of an assignment of plots to strata (non-overlapping areas of a known or estimated size) that in aggregate define the population of interest. There is an assignment of plots to every stratum, and all plots are assigned to one, and only one stratum, for each evaluation. FIA uses the term “evaluation” to reference the relationship that links a set of plots to a set of strata for estimation purposes. Thus, an evaluation is a set of plots defined

in the FIADB that can be used to make a statistically valid sample-based estimate for a population (area of land) based on a specific stratification.

Each evaluation used by FIA is identified, named, and stored as a single entry in the POP_EVAL table. The important data items in the POP_EVAL table are listed in table 4.3 for all evaluations that are loaded into the FIADB for data collected in Minnesota through 2006. CN is the control number that uniquely identifies the entry and is used in creating links to other tables. RSCD (Region or Station Code) and EVALID (Evaluation Identifier) are the natural identifiers of a specific record. EVAL_DESCR provides a description of the evaluation. STATECD and LOCATION_NM describe the geographic extent of the population that was sampled and REPORT_YEAR_NM describes the years in which the sample was taken. For older periodic inventories, REPORT_YEAR_NM typically reflects a single reporting year (the one used in the FIA publications), even though the plots may have been measured over several years. Annual inventories (taken since 1999) list the years of data measurements used in the estimation. There are usually multiple evaluations for a specific year because not all plots observed have every attribute of interest, and/or different stratifications are used in the estimation of different attributes of interest. For example, volume estimation can be done on plots measured at only one point in time. However, growth estimates require repeat measurements. Thus, evaluations for the estimation of growth only assign those plots that are repeat measurement plots to strata, and do not include one-time measurement plots.

Table 4.3. Important POP_EVAL entries for Minnesota through 2006 from the FIADB.

	Data item names						
	CN	RSCD	EVALID	EVAL DESCR	STATECD	LOCATION NM	REPORT YEAR NM
**Data item values**	107106457010661	23	277701	Minnesota, 1977: area (periodic)	27	Minnesota	1977
	107106458010661	23	277702	Minnesota, 1977: volume (periodic)	27	Minnesota	1977
	107106459010661	23	277703	Minnesota, 1977: growth (periodic)	27	Minnesota	1977
	107106460010661	23	277704	Minnesota, 1977: mortality (periodic)	27	Minnesota	1977
	107106461010661	23	277705	Minnesota, 1977: removals (periodic)	27	Minnesota	1977
	107106462010661	23	279001	Minnesota, 1990: area (periodic)	27	Minnesota	1990
	107106463010661	23	279002	Minnesota, 1990: volume (periodic)	27	Minnesota	1990
	107106464010661	23	279003	Minnesota, 1990: growth (periodic)	27	Minnesota	1990
	107106465010661	23	279004	Minnesota, 1990: mortality (periodic)	27	Minnesota	1990
	107106466010661	23	279005	Minnesota, 1990: removals (periodic)	27	Minnesota	1990
	107106467010661	23	279006	Minnesota, 1990: change (periodic)	27	Minnesota	1990
	107106444010661	23	270300	Minnesota, 1999-2003: all land	27	Minnesota	1999;2000;2001; 2002;2003
	107106445010661	23	270301	Minnesota, 1999-2003: area/volume	27	Minnesota	1999;2000;2001; 2002;2003
	107106446010661	23	270302	Minnesota, 1990 to 1999-2003: GRM	27	Minnesota	1999;2000;2001; 2002;2003
	107106448010661	23	270400	Minnesota, 2000-2004: all land	27	Minnesota	2000;2001;2002; 2003;2004
	107106449010661	23	270401	Minnesota, 2000-2004: area/volume	27	Minnesota	2000;2001;2002; 2003;2004
	107106450010661	23	270402	Minnesota, 1999 to 2004: GRM	27	Minnesota	2004
	107106451010661	23	270500	Minnesota, 2001-2005: all land	27	Minnesota	2001;2002;2003; 2004;2005
	107106452010661	23	270501	Minnesota, 2001-2005: area/volume	27	Minnesota	2001;2002;2003; 2004;2005
	107106453010661	23	270502	Minnesota, 1999-2000 to 2004-2005: GRM	27	Minnesota	2004;2005
	107106454010661	23	270600	Minnesota, 2002-2006: all land	27	Minnesota	2002;2003;2004; 2005;2006

	Data item names						
	CN	RSCD	EVALID	EVAL DESCR	STATECD	LOCATION NM	REPORT YEAR NM
	107106455010661	23	270601	Minnesota, 2002-2006: area/volume	27	Minnesota	2002;2003;2004; 2005;2006
	107106456010661	23	270602	Minnesota, 1999-2001 to 2004-2006: GRM	27	Minnesota	2004;2005;2006

An evaluation group is the set of evaluations that goes into the contents of a typical FIA report for a State. For example the evaluations that went into the report entitled “Minnesota’s forests 1999-2003 (Part A.)” (Miles and others 2007) are identified by EVALIDs 270300, 270301 and 270302, and are collectively identified by a single record in the POP_EVAL_GRP table. Table 4.4 lists the important attributes for all evaluation groups that are loaded into FIADB for data collected in Minnesota through 2006.

Table 4.4. Important POP_EVAL_GRP entries for Minnesota through 2006 from the FIADB.

Data item names	Data item values					
CN	107114016010661	107114017010661	107114012010661	107114013010661	107114014010661	107114015010661
EVAL_CN_FOR_EXPALL			107106444010661	107106448010661	107106451010661	107106454010661
EVAL_CN_FOR_EXPCURR	107106457010661	107106462010661	107106445010661	107106449010661	107106452010661	107106455010661
EVAL_CN_FOR_EXPVOL	107106458010661	107106463010661	107106445010661	107106449010661	107106452010661	107106455010661
EVAL_CN_FOR_EXPGROW	107106459010661	107106464010661	107106446010661	107106450010661	107106453010661	107106456010661
EVAL_CN_FOR_EXPMORT	107106460010661	107106465010661	107106446010661	107106450010661	107106453010661	107106456010661
EVAL_CN_FOR_EXPREMV	107106461010661	107106466010661	107106446010661	107106450010661	107106453010661	107106456010661
RSCD	23	23	23	23	23	23
EVAL_GRP	271977	271990	272003	272004	272005	272006
EVAL_GRP_DESCR	Minnesota: 1977	Minnesota: 1990	Minnesota: 1999;2000;2001; 2002;2003	Minnesota: 2000;2001;2002; 2003;2004	Minnesota: 2001;2002;2003; 2004;2005	Minnesota: 2002;2003;2004; 2005;2006

In the POP_EVAL_GRP table the data item EVAL_GRP identifies the evaluation group by its State code (first 2 digits) and a year (last 4 digits), which is the year commonly associated with estimates (if EVAL_GRP does not follow this format, see the EVAL_GRP_DESCR for the precise identification). In table 4.4 we see evaluation groups for two periodic inventory estimates (1977 and 1990), and four annual estimates (2003, 2004, 2005 and 2006). The EVAL_GRP_DESCR describes the groups, and indicates that all of the annual inventory estimates are based on 5 years of measurements taken over the 5-year period ending with that date. The data items EVAL_CN_FOR_EXPALL, EVAL_CN_FOR_EXPCURR, EVAL_CN_FOR_EXPVOL, EVAL_CN_FOR_EXPGROW, EVAL_CN_FOR_EXPMORT, and EVAL_CN_FOR_EXPREMV identify the evaluations in POP_EVAL that are appropriate for the estimation of various attributes of interest. EVAL_CN_FOR_EXPCURR identifies the evaluation used in the estimation of most area estimates, such as the area of forestland or the area of timberland. EVAL_CN_FOR_EXPVOL identifies the evaluation used in the estimation of tree-level attributes such as number, volume, and biomass of trees, and seedling-level estimates, such as number of seedlings. EVAL_CN_FOR_EXPGROW, EVAL_CN_FOR_EXPMORT, and EVAL_CN_FOR_EXPREMV identify the evaluations used in the estimation of growth, mortality, and removals, respectively. The evaluation identified by EVAL_CN_FOR_EXPALL is only appropriate for area estimation where the area of hazardous and denied access are of interest. All other evaluations treat hazardous and denied access as non-measured and adjust the estimate to account for these areas.

The POP_EVAL_TYP table was added to the FIADB in the transition from version 3.0 to 4.0 to provide a link between the evaluation groups in POP_EVAL_GRP and the evaluations in POP_EVAL. In FIADB 3.0, users could select the appropriate evaluation sequence number (EVAL_CN_FOR_xxx) from the POP_EVAL_GRP table. This evaluation sequence number allowed them to select the appropriate plots and associated expansions. Evaluations are now also identified by the type of evaluation in the value of POP_EVAL_TYP.EVAL_TYP, which can take on values of "EXPALL," "EXPCURR," "EXPVOL," "EXPGROW," "EXPMORT," or "EXPREMV" to identify the type of attributes that can be estimated from a specific evaluation. This table allows users to perform similar queries on the appropriate evaluation by identifying only the eval_grp (STATECD*10000 + INV_YR) and evaluation type (EVAL_TYP) and allows a variety of evaluations to be added in the future. The methods used in version 3.0 will continue to work in version 4.0. The examples presented here incorporate the POP_EVAL_TYP as the link from the POP_EVAL_GRP to the POP_EVAL table. In the examples below, either of the two joins will select the appropriate evaluation for the estimation of area and volume attributes for the Minnesota 2003 annual inventory.

FIADB 4.0 example:

```
select pev.cn,pev.eval_descr
from pop_eval_typ pet, pop_eval pev, pop_eval_grp peg
where  peg.eval_grp = 272003 and peg.cn = pet.eval_grp_cn and
       pev.cn = pet.eval_cn and pet.eval_typ = 'EXPCURR'
```

FIADB 3.0 example:

```
select pev.cn, pev.eval_descr
from pop_eval pev, pop_eval_grp peg
where  peg.eval_grp = 272003 and
       pev.cn = peg.eval_cn_for_expcurr
```

3. Linking the appropriate tables in FIADB to produce estimates of attributes of interest for a population

The following Oracle™ SQL script can be modified to produce an estimate of any condition-, tree-, or seedling-level attribute listed in the REF_POP_ATTRIBUTE table. In this standard script (example 4.1), the non-bold text applies to all estimates and the bold text is modified by the user, depending on the desired attribute of interest and evaluation group. The line numbers have been added for reference. On line 01, the text in the column EXPRESSION in the REF_POP_ATTRIBUTE table associated with the desired attribute of interest should be inserted. Lines 05 or 06 include either the TREE table or SEEDLING table, and neither line should be included for condition level estimates. Line 05 should be included for tree level estimates and line 06 should be included for seedling level estimates. On line 14, the additions to the SQL where clause from the WHERE_CLAUSE column of the REF_POP_ATTRIBUTE table for the desired attribute of interest should be inserted. Finally, on line 21, the desired evaluation group needs to be indicated by replacing the characters SSYYYY with the desired evaluation group, whereby SS = STATECD of the desired State, and YYYY = year of the desired inventory (if EVAL_GRP does not follow this format, see the EVAL_GRP_DESCR for the precise identification). With these changes, a user can produce the standard estimates for any desired population from the REF_POP_ATTRIBUTE table.

Estimation requires linking the attribute values (on the COND, TREE, and SEEDLING tables) to the stratification information (on the POP_PLOT_STRATUM_ASSGN, POP_STRATUM, and POP_ESTN_UNIT) for the selected evaluation that defines the sample. Those links are provided in lines 15 thru 20 of the script, and these lines do not change. Line 15 links the POP_PLOT_STRATUM_ASSGN record (which contains EXPNS, the plot expansion factor or acres assigned to the plot) to the plot record. Line 16 links the POP_PLOT_STRATUM_ASSGN record to the POP_STRATUM (which identifies each stratum in the estimation unit). Line 17 links the POP_ESTN_UNIT (which identifies each estimation unit in the evaluation) to the POP_ STRATUM record. Line 18 links the POP_EVAL, which identifies each evaluation, to the specific evaluation that is required for the estimation. Lines 19 and 20 link the appropriate evaluation to the attribute and evaluation group for which the estimate is being made. See figure 6 for a schematic of links of some of the FIADB tables.

The following table shows some common aliases or abbreviations used within a SQL script to reduce the overall length of the script and improve readability.

Common aliases for FIADB tables

p	PLOT
c	COND
t	TREE
s	SEEDLING
ppsa	POP_PLOT_STRATUM_ASSGN
psm	POP_STRATUM
peu	POP_ESTN_UNIT
pet	POP_EVAL_TYP
peg	POP_EVAL_GRP
pev	POP_EVAL

Example 4.1. Standard estimation script

```
01  SELECT SUM(psm.expns * EXPRESSION -- insert ref_pop_attribute EXPRESSION here
02          ) estimate
03   FROM cond                  c,
04       plot                   p,
05       tree                   t, -- tree table must be included for tree level estimates
06       seedling               s, -- seedling table must be included for seedling level estimate
07       pop_plot_stratum_assgn ppsa,
08       pop_stratum            psm,
09       pop_estn_unit          peu,
10       pop_eval               pev,
11       pop_eval_typ           pet,
12       pop_eval_grp           peg
13  WHERE p.cn = c.plt_cn
14    WHERE_CLAUSE -- insert ref_pop_attribute WHERE_CLAUSE here
15    AND ppsa.plt_cn = p.cn
16    AND ppsa.stratum_cn = psm.cn
17    AND peu.cn = psm.estn_unit_cn
18    AND pev.cn = peu.eval_cn
19    AND pev.cn = pet.eval_cn
20    AND pet.eval_grp_cn = peg.cn
21    AND peg.eval_grp = SSYYYY -- the desired evaluation group must be specified
```

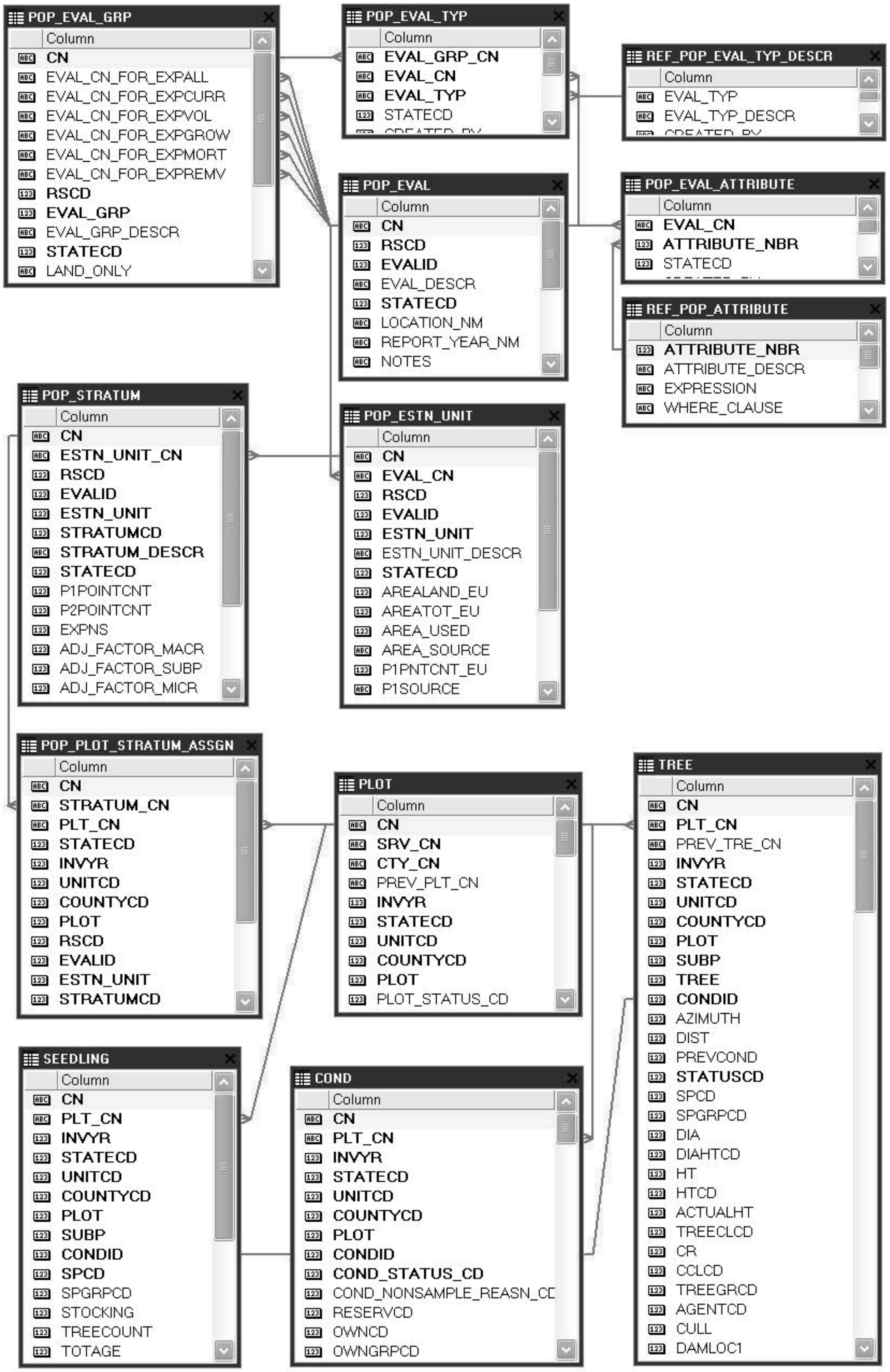

Figure 6. An abbreviated diagram of select FIADB tables. Note that there are more columns in each table than are shown.

In the following four examples (4.2, 4.3, 4.4, and 4.5), the scripts are modified from above to produce condition, tree, and seedling level estimates for the Minnesota 2003 inventory. Here the sections in bold are the sections that changed from the standard estimation script, e.g., the REF_POP_ATTRIBUTE.EXPRESSION and REF_POP_ATTRIBUTE.WHERE_CLAUSE have been inserted, along with the chosen evaluation number.

Example 4.2 Estimate area of timberland (acres)

```
SELECT SUM(psm.expns * c.condprop_unadj *
        decode(c.prop_basis,
            'MACR',
            psm.adj_factor_macr,
            psm.adj_factor_subp) -- this is the expression from ref_pop_attribute table
        ) estimate
 FROM cond                    c,
     plot                     p,
     pop_plot_stratum_assgn  ppsa,
     pop_stratum              psm,
     pop_estn_unit           peu,
     pop_eval                pev,
     pop_eval_typ            pet,
     pop_eval_grp            peg
WHERE p.cn = c.plt_cn
  AND pet.eval_typ = 'EXPCURR'
  AND c.cond_status_cd = 1
  AND c.reservcd = 0
  AND c.siteclcd IN (1, 2, 3, 4, 5, 6) -- this is the where_clause from ref_pop_attribute table
  AND ppsa.plt_cn = p.cn
  AND ppsa.stratum_cn = psm.cn
  AND peu.cn = psm.estn_unit_cn
  AND pev.cn = peu.eval_cn
  AND pev.cn = pet.eval_cn
  AND pet.eval_grp_cn = peg.cn
  AND peg.eval_grp = 272003 -- the desired evaluation group must be specified
```

Produces the following estimate of acres of timberland:

ESTIMATE
14,734,137

Example 4.3 Estimate number of live trees on forest land (trees)

```
 SELECT SUM(psm.expns * t.tpa_unadj *
        decode(dia,
             null,
             adj_factor_subp,
             decode(least(dia, 5 - 0.001),
                  dia,
                  adj_factor_micr,
                  decode(least(dia,
                           nvl(macro_breakpoint_dia, 9999) - 0.001),
                      dia,
                      adj_factor_subp,
                      adj_factor_macr))) -- this is the expression from ref_pop_attribute table
        ) estimate
  FROM cond                    c,
     plot                      p,
     tree                      t, -- tree table must be included for tree level estimates
     pop_plot_stratum_assgn ppsa,
     pop_stratum               psm,
     pop_estn_unit             peu,
     pop_eval                  pev,
     pop_eval_typ              pet,
     pop_eval_grp              peg
 WHERE p.cn = c.plt_cn
   AND pet.eval_typ = 'EXPVOL'
   AND t.plt_cn = c.plt_cn
   AND t.condid = c.condid
   AND c.cond_status_cd = 1
   AND t.statuscd = 1
   AND t.dia >= 1.0 -- additional where_clause from ref_pop_attribute table
   AND ppsa.plt_cn = p.cn
   AND ppsa.stratum_cn = psm.cn
   AND peu.cn = psm.estn_unit_cn
   AND pev.cn = peu.eval_cn
   AND pev.cn = pet.eval_cn
   AND pet.eval_grp_cn = peg.cn
   AND peg.eval_grp = 272003 -- the desired evaluation group must be specified
```

Produces the following estimate of total number of live trees on forest land:

ESTIMATE
12,078,196,211

Example 4.4 Estimate number of live seedlings on timberland (seedlings)

```
SELECT SUM(psm.expns * s.tpa_unadj * adj_factor_micr -- expression from ref_pop_attribute table
          ) estimate
  FROM cond                    c,
      plot                     p,
      seedling                 s, -- seedling table must be included for seedling level estimates
      pop_plot_stratum_assgn ppsa,
      pop_stratum              psm,
      pop_estn_unit            peu,
      pop_eval                 pev,
      pop_eval_typ             pet,
      pop_eval_grp             peg
 WHERE p.cn = c.plt_cn
   AND pet.eval_typ = 'EXPVOL'
   AND s.plt_cn = c.plt_cn
   AND s.condid = c.condid
   AND c.cond_status_cd = 1
   AND c.reservcd = 0
   AND c.siteclcd IN (1, 2, 3, 4, 5, 6) -- additional where_clause from ref_pop_attribute table
   AND ppsa.plt_cn = p.cn
   AND ppsa.stratum_cn = psm.cn
   AND peu.cn = psm.estn_unit_cn
   AND pev.cn = peu.eval_cn
   AND pev.cn = pet.eval_cn
   AND pet.eval_grp_cn = peg.cn
   AND peg.eval_grp = 272003 -- the desired evaluation group must be specified
```

Produces the following estimate of total number of live seedlings on timberland:

ESTIMATE
37,141,783,495

Example 4.5 Estimate volume of growing-stock on timberland (cubic feet)

```
SELECT SUM(psm.expns * t.tpa_unadj * t.volcfnet *
        decode(dia,
              null,
              adj_factor_subp,
              decode(least(dia, 5 - 0.001),
                    dia,
                    adj_factor_micr,
                    decode(least(dia,
                                nvl(macro_breakpoint_dia, 9999) - 0.001),
                          dia,
                          adj_factor_subp,
                          adj_factor_macr))) -- this is the expression from ref_pop_attribute table
        ) estimate
  FROM cond                  c,
     plot                    p,
     tree                    t, -- tree table must be included for tree level estimates
     pop_plot_stratum_assgn ppsa,
     pop_stratum             psm,
     pop_estn_unit           peu,
     pop_eval                pev,
     pop_eval_typ            pet,
     pop_eval_grp            peg
 WHERE p.cn = c.plt_cn
   AND pet.eval_typ = 'EXPVOL'
   AND t.plt_cn = c.plt_cn
   AND t.condid = c.condid
   AND c.cond_status_cd = 1
   AND c.reservcd = 0
   AND c.siteclcd in (1, 2, 3, 4, 5, 6)
   AND t.statuscd = 1
   AND t.treeclcd = 2
   AND t.dia >= 5.0 -- additional where_clause from ref_pop_attribute table
   AND ppsa.plt_cn = p.cn
   AND ppsa.stratum_cn = psm.cn
   AND peu.cn = psm.estn_unit_cn
   AND pev.cn = peu.eval_cn
   AND pev.cn = pet.eval_cn
   AND pet.eval_grp_cn = peg.cn
   AND peg.eval_grp = 272003 -- the desired evaluation group must be specified
```

Produces the following estimate of total growing-stock volume (cubic feet) on timberland:

ESTIMATE
15,242,634,295

Users of the FIADB who wish to produce population estimates should test these four examples to be sure they are obtaining identical estimates before proceeding to more complicated estimation. Important Note: Users who access data from periodic inventories should restrict the estimation only to the standard timberland estimates. In most cases, for periodic inventories, the FIADB contains only condition level information on reserved and unproductive forest lands, and tree level information on timberland.

4. Producing estimates with sampling errors for attributes of interest for a population

Producing population estimates that include error estimates (sampling error or variance of the estimate) along with the estimated total is more complicated. The following Oracle™ SQL script can be used as a template in producing estimates with sampling errors. The line numbers have been added for reference. This example follows the notation used in Bechtold and Patterson (2005, equation 4.14 on page 55). Again, the portions of the script that should be changed by the user to specify the attribute of interest and population are in bold. Besides returning the estimates and sampling errors, this script also outputs the total number of plots in the sample (TOTAL_PLOTS), the number of plots where the attribute of interest was observed to occur (NON_ZERO_PLOTS), and the total population area (TOTAL_POPULATION_ACRES). This procedure produces two intermediate tables: phase_1_summary and phase_2_summary. Phase_1_summary is a stratum-level table that contains the stratification information necessary in the estimation within strata sample sizes (n_h), stratum weights (W_h), and population area (A_T). Phase_2_summary is a stratum-level table that contains a summary of the attribute of interest on per-unit-area basis (y_{hid}), including the sum and sum of the squared plot-level values and the number of plots where the attribute of interest was observed.

Example 4.6. Standard script for estimates with sampling errors

```
SELECT eval_grp,
       SUM(estimate_by_estn_unit.estimate) estimate,
       CASE
         WHEN SUM(estimate_by_estn_unit.estimate) > 0 THEN
          round(sqrt(SUM(estimate_by_estn_unit.var_of_estimate)) /
                SUM(estimate_by_estn_unit.estimate) * 100,
                3)
         ELSE
          0
       END AS se_of_estimate_pct,
       SUM(estimate_by_estn_unit.var_of_estimate) var_of_estimate,
       SUM(estimate_by_estn_unit.total_plots) total_plots,
       SUM(estimate_by_estn_unit.non_zero_plots) non_zero_plots,
       SUM(estimate_by_estn_unit.total_population_area_acres) total_population_acres
  FROM (SELECT pop_eval_grp_cn,
               eval_grp,
               estn_unit_cn,
               SUM(nvl(ysum_hd, 0) * phase_1_summary.expns) estimate,
               SUM(phase_1_summary.n_h) total_plots,
               SUM(phase_2_summary.number_plots_in_domain) domain_plots,
               SUM(phase_2_summary.non_zero_plots) non_zero_plots,
               total_area * total_area / SUM(phase_1_summary.n_h) *
               ((SUM(w_h * phase_1_summary.n_h *
                     (((nvl(ysum_hd_sqr, 0) / phase_1_summary.n_h) -
                     ((nvl(ysum_hd, 0) / phase_1_summary.n_h) *
                     (nvl(ysum_hd, 0) / phase_1_summary.n_h))) /
                     (phase_1_summary.n_h - 1)))) +
               1 / SUM(phase_1_summary.n_h) *
               (SUM((1 - w_h) * phase_1_summary.n_h *
                     (((nvl(ysum_hd_sqr, 0) / phase_1_summary.n_h) -
                     ((nvl(ysum_hd, 0) / phase_1_summary.n_h) *
                     (nvl(ysum_hd, 0) / phase_1_summary.n_h))) /
```

```
                     (phase_1_summary.n_h - 1))))) var_of_estimate,
                total_area total_population_area_acres
           FROM (SELECT peg.eval_grp,
                        peg.cn pop_eval_grp_cn,
                        psm.estn_unit_cn,
                        psm.expns,
                        psm.cn pop_stratum_cn,
                        p1pointcnt /
                        (SELECT SUM(strs.p1pointcnt)
                           FROM pop_stratum strs
                          WHERE strs.estn_unit_cn = psm.estn_unit_cn) w_h,
                        (SELECT SUM(strs.p1pointcnt)
                           FROM pop_stratum strs
                          WHERE strs.estn_unit_cn = psm.estn_unit_cn) n_prime,
                        p1pointcnt n_prime_h,
                        (SELECT SUM(eu_s.area_used)
                           FROM pop_estn_unit eu_s
                          WHERE eu_s.cn = psm.estn_unit_cn) total_area,
                        psm.p2pointcnt n_h
                   FROM pop_estn_unit peu,
                        pop_stratum      psm,
                        pop_eval         pev,
                        pop_eval_grp     peg,
                        pop_eval_typ     pet
                  WHERE peu.cn = psm.estn_unit_cn
                    and pev.cn = peu.eval_cn
                    and pet.eval_cn = pev.cn
                    and pet.eval_grp_cn = peg.cn
                    and pet.eval_typ = 'EXPXXX' -- specify the appropriate expansion
                    AND peg.eval_grp = SSYYYY -- the desired evaluation group must be specified
                ) phase_1_summary,
                (SELECT pop_stratum_cn,
                        SUM(y_hid_adjusted) ysum_hd,
                        SUM(y_hid_adjusted * y_hid_adjusted) ysum_hd_sqr,
                        COUNT(*) number_plots_in_domain,
                        SUM(decode(y_hid_adjusted, 0, 0, NULL, 0, 1)) non_zero_plots
                   FROM (SELECT psm.cn pop_stratum_cn,
                                p.cn plt_cn,
                                SUM(EXPRESSION) y_hid_adjusted
                                               -- the appropriate expression from ref_pop_attribute table
                           FROM cond c,
                                plot        p,
                                tree        t, -- tree table must be included for tree level estimates
                                seedling s, -- seedling table must be included for seedling level estimates
                                pop_plot_stratum_assgn ppsa,
                                pop_stratum            psm,
                                pop_estn_unit          peu,
                                pop_eval               pev,
                                pop_eval_grp           peg,
                                pop_eval_typ           pet
                          WHERE p.cn = c.plt_cn
                            WHERE_CLAUSE -- additional where_clause from ref_pop_attribute table
                            AND ppsa.plt_cn = p.cn
                            AND ppsa.stratum_cn = psm.cn
                            AND peu.cn = psm.estn_unit_cn
                            AND pev.cn = peu.eval_cn
```

```
                              AND pet.eval_cn = pev.cn
                              AND pet.eval_grp_cn = peg.cn
                              AND peg.eval_grp = SSYYYY
                                          -- the desired evaluation group must be specified
                            GROUP BY psm.cn, p.cn)
                    GROUP BY pop_stratum_cn) phase_2_summary
            WHERE phase_1_summary.pop_stratum_cn =
                  phase_2_summary.pop_stratum_cn(+)
            GROUP BY pop_eval_grp_cn,
                     eval_grp,
                     estn_unit_cn,
                     phase_1_summary.total_area) estimate_by_estn_unit
  GROUP BY pop_eval_grp_cn, eval_grp
```

In the following three examples the scripts were modified from above to produce condition, tree, and seedling level estimates for the Minnesota 2003 inventory. Here the sections in bold are the sections that changed from the standard script for estimates with sampling errors.

Example 4.7. Estimate Area of timberland (acres) with sampling error. Note the bold sections in this example match the bold sections in example 4.2, which estimates the same area without sampling errors.

```
SELECT eval_grp,
       SUM(estimate_by_estn_unit.estimate) estimate,
       CASE
         WHEN SUM(estimate_by_estn_unit.estimate) > 0 THEN
          round(sqrt(SUM(estimate_by_estn_unit.var_of_estimate)) /
                SUM(estimate_by_estn_unit.estimate) * 100,
                3)
         ELSE
          0
       END AS se_of_estimate_pct,
       SUM(estimate_by_estn_unit.var_of_estimate) var_of_estimate,
       SUM(estimate_by_estn_unit.total_plots) total_plots,
       SUM(estimate_by_estn_unit.non_zero_plots) non_zero_plots,
       SUM(estimate_by_estn_unit.total_population_area_acres) total_population_acres
  FROM (SELECT pop_eval_grp_cn,
               eval_grp,
               estn_unit_cn,
               SUM(nvl(ysum_hd, 0) * phase_1_summary.expns) estimate,
               SUM(phase_1_summary.n_h) total_plots,
               SUM(phase_2_summary.number_plots_in_domain) domain_plots,
               SUM(phase_2_summary.non_zero_plots) non_zero_plots,
               total_area * total_area / SUM(phase_1_summary.n_h) *
               ((SUM(w_h * phase_1_summary.n_h *
                     (((nvl(ysum_hd_sqr, 0) / phase_1_summary.n_h) -
                     ((nvl(ysum_hd, 0) / phase_1_summary.n_h) *
                     (nvl(ysum_hd, 0) / phase_1_summary.n_h))) /
                     (phase_1_summary.n_h - 1)))) +
               1 / SUM(phase_1_summary.n_h) *
               (SUM((1 - w_h) * phase_1_summary.n_h *
                     (((nvl(ysum_hd_sqr, 0) / phase_1_summary.n_h) -
                     ((nvl(ysum_hd, 0) / phase_1_summary.n_h) *
                     (nvl(ysum_hd, 0) / phase_1_summary.n_h))) /
```

```
            (phase_1_summary.n_h - 1))))) var_of_estimate,
        total_area total_population_area_acres
    FROM (SELECT peg.eval_grp,
              peg.cn pop_eval_grp_cn,
              psm.estn_unit_cn,
              psm.cn pop_stratum_cn,
              psm.expns,
              p1pointcnt /
              (SELECT SUM(strs.p1pointcnt)
                 FROM pop_stratum strs
                WHERE strs.estn_unit_cn = psm.estn_unit_cn) w_h,
              (SELECT SUM(strs.p1pointcnt)
                 FROM pop_stratum strs
                WHERE strs.estn_unit_cn = psm.estn_unit_cn) n_prime,
              p1pointcnt n_prime_h,
              (SELECT SUM(eu_s.area_used)
                 FROM pop_estn_unit eu_s
                WHERE eu_s.cn = psm.estn_unit_cn) total_area,
              psm.p2pointcnt n_h
          FROM pop_estn_unit peu,
              pop_stratum         psm,
              pop_eval            pev,
              pop_eval_grp        peg,
              pop_eval_typ        pet
         WHERE peu.cn = psm.estn_unit_cn
           AND pev.cn = peu.eval_cn
           AND pet.eval_cn = pev.cn
           AND pet.eval_grp_cn = peg.cn
           AND pet.eval_typ = 'EXPCURR' -- specify the appropriate expansion
           AND peg.eval_grp = 272003 -- the desired evaluation group must be specified
        ) phase_1_summary,
        (SELECT pop_stratum_cn,
              SUM(y_hid_adjusted) ysum_hd,
              SUM(y_hid_adjusted * y_hid_adjusted) ysum_hd_sqr,
              COUNT(*) number_plots_in_domain,
              SUM(decode(y_hid_adjusted, 0, 0, NULL, 0, 1)) non_zero_plots
          FROM (SELECT psm.cn pop_stratum_cn,
                    p.cn plt_cn,
                    SUM(c.condprop_unadj *
                        decode(c.prop_basis,
                               'MACR',
                               psm.adj_factor_macr,
                               psm.adj_factor_subp) -- the expression from ref_pop_attribute table
                               ) y_hid_adjusted
                 FROM cond                    c,
                    plot                      p,
                    pop_plot_stratum_assgn ppsa,
                    pop_stratum               psm,
                    pop_estn_unit             peu,
                    pop_eval                  pev,
                    pop_eval_grp              peg,
                    pop_eval_typ              pet
                WHERE p.cn = c.plt_cn
                  AND pet.eval_typ = 'EXPCURR'
                  AND c.cond_status_cd = 1
                  AND c.reservcd = 0
                  AND c.siteclcd IN (1, 2, 3, 4, 5, 6)
                                        -- additional where_clause from ref_pop_attribute table
```

```
                    AND ppsa.plt_cn = p.cn
                    AND ppsa.stratum_cn = psm.cn
                    AND peu.cn = psm.estn_unit_cn
                    AND pev.cn = peu.eval_cn
                   AND pet.eval_cn = pev.cn
                   AND pet.eval_grp_cn = peg.cn
                   AND peg.eval_grp = 272003 -- the desired evaluation group must be specified
                  GROUP BY psm.cn, p.cn)
          GROUP BY pop_stratum_cn) phase_2_summary
      WHERE phase_1_summary.pop_stratum_cn =
           phase_2_summary.pop_stratum_cn(+)
      GROUP BY pop_eval_grp_cn,
             eval_grp,
             estn_unit_cn,
             phase_1_summary.total_area) estimate_by_estn_unit
 GROUP BY pop_eval_grp_cn, eval_grp
```

Produces the following estimate of acres of timberland with sampling error:

**EVAL GRP**	272003
**ESTIMATE**	14,734,137
**SE OF ESTIMATE PCT**	0.7
**VAR OF ESTIMATE**	10,998,768,175
**TOTAL PLOTS**	16,041
**NON ZERO PLOTS**	4,774
**TOTAL POPULATION ACRES**	54,002,539

Example 4.8. Estimate number of live trees on forest land (trees) with sampling error. Note the bold sections in this example match the bold sections in example 4.3, which estimates the same number of trees without sampling errors.

```
SELECT eval_grp,
       SUM(estimate_by_estn_unit.estimate) estimate,
       CASE
         WHEN SUM(estimate_by_estn_unit.estimate) > 0 THEN
          round(sqrt(SUM(estimate_by_estn_unit.var_of_estimate)) /
                SUM(estimate_by_estn_unit.estimate) * 100,
                3)
         ELSE
          0
       END AS se_of_estimate_pct,
       SUM(estimate_by_estn_unit.var_of_estimate) var_of_estimate,
       SUM(estimate_by_estn_unit.total_plots) total_plots,
       SUM(estimate_by_estn_unit.non_zero_plots) non_zero_plots,
       SUM(estimate_by_estn_unit.total_population_area_acres) total_population_acres
  FROM (SELECT pop_eval_grp_cn,
             eval_grp,
             estn_unit_cn,
             sum(nvl(ysum_hd, 0) * phase_1_summary.expns) estimate,
             SUM(phase_1_summary.n_h) total_plots,
             SUM(phase_2_summary.number_plots_in_domain) domain_plots,
             SUM(phase_2_summary.non_zero_plots) non_zero_plots,
             total_area * total_area / SUM(phase_1_summary.n_h) *
```

```
        ((SUM(w_h * phase_1_summary.n_h *
              (((nvl(ysum_hd_sqr, 0) / phase_1_summary.n_h) -
              ((nvl(ysum_hd, 0) / phase_1_summary.n_h) *
              (nvl(ysum_hd, 0) / phase_1_summary.n_h))) /
              (phase_1_summary.n_h - 1)))) +
        1 / SUM(phase_1_summary.n_h) *
        (SUM((1 - w_h) * phase_1_summary.n_h *
              (((nvl(ysum_hd_sqr, 0) / phase_1_summary.n_h) -
              ((nvl(ysum_hd, 0) / phase_1_summary.n_h) *
              (nvl(ysum_hd, 0) / phase_1_summary.n_h))) /
              (phase_1_summary.n_h - 1))))) var_of_estimate,
        total_area total_population_area_acres
    FROM (SELECT peg.eval_grp,
                 peg.cn pop_eval_grp_cn,
                 psm.estn_unit_cn,
                 psm.expns,
                 psm.cn pop_stratum_cn,
                 p1pointcnt /
                 (SELECT SUM(strs.p1pointcnt)
                    FROM pop_stratum strs
                   WHERE strs.estn_unit_cn = psm.estn_unit_cn) w_h,
                 (SELECT SUM(strs.p1pointcnt)
                    FROM pop_stratum strs
                   WHERE strs.estn_unit_cn = psm.estn_unit_cn) n_prime,
                 p1pointcnt n_prime_h,
                 (SELECT SUM(eu_s.area_used)
                    FROM pop_estn_unit eu_s
                   WHERE eu_s.cn = psm.estn_unit_cn) total_area,
                 psm.p2pointcnt n_h
            FROM pop_estn_unit peu,
                 pop_stratum      psm,
                 pop_eval         pev,
                 pop_eval_grp     peg,
                 pop_eval_typ     pet
           WHERE peu.cn = psm.estn_unit_cn
             AND pev.cn = peu.eval_cn
             AND pet.eval_cn = pev.cn
             AND pet.eval_grp_cn = peg.cn
             AND pet.eval_typ = 'EXPVOL' -- specify the appropriate expansion
             AND peg.eval_grp = 272003 -- the desired evaluation group must be specified
          ) phase_1_summary,
        (SELECT pop_stratum_cn,
                 SUM(y_hid_adjusted) ysum_hd,
                 SUM(y_hid_adjusted * y_hid_adjusted) ysum_hd_sqr,
                 COUNT(*) number_plots_in_domain,
                 SUM(decode(y_hid_adjusted, 0, 0, NULL, 0, 1)) non_zero_plots
            FROM (SELECT psm.cn pop_stratum_cn,
                         p.cn plt_cn,
                         SUM(t.tpa_unadj *
                            decode(dia,
                                   NULL,
                                   adj_factor_subp,
                                   decode(least(dia, 5 - 0.001),
                                          dia,
                                          adj_factor_micr,
                                          decode(least(dia,
                                                       nvl(macro_breakpoint_dia,
                                                          9999) - 0.001),
```

```
                                   dia,
                                   adj_factor_subp,
                                   adj_factor_macr))) -- expression from ref_pop_attribute table
                   ) y_hid_adjusted
              FROM cond                   c,
                   plot                   p,
                   tree                   t, -- tree table must be included for tree level estimates
                   pop_plot_stratum_assgn ppsa,
                   pop_stratum            psm,
                   pop_estn_unit          peu,
                   pop_eval               pev,
                   pop_eval_grp           peg,
                   pop_eval_typ           pet
             WHERE p.cn = c.plt_cn
               AND pet.eval_typ = 'EXPVOL'
               AND t.plt_cn = c.plt_cn
               AND t.condid = c.condid
               AND c.cond_status_cd = 1
               AND t.statuscd = 1
               AND t.dia >= 1.0 -- additional where_clause from ref_pop_attribute table
               AND ppsa.plt_cn = p.cn
               AND ppsa.stratum_cn = psm.cn
               AND peu.cn = psm.estn_unit_cn
               AND pev.cn = peu.eval_cn
               AND pet.eval_cn = pev.cn
               AND pet.eval_grp_cn = peg.cn
               AND pev.cn = peg.eval_cn_for_expvol -- specify the appropriate expansion
               AND peg.eval_grp = 272003 -- the desired evaluation group must be specified
             GROUP BY psm.cn, p.cn)
         GROUP BY pop_stratum_cn) phase_2_summary
     WHERE phase_1_summary.pop_stratum_cn =
        phase_2_summary.pop_stratum_cn(+)
     GROUP BY pop_eval_grp_cn,
          eval_grp,
          estn_unit_cn,
          phase_1_summary.total_area) estimate_by_estn_unit
 GROUP BY pop_eval_grp_cn, eval_grp
```

Produces the following estimate of number of live trees on forest land with sampling error:

**EVAL GRP**	272003
**ESTIMATE**	12,078,196,211
**SE OF ESTIMATE PCT**	1.3
**VAR OF ESTIMATE**	25,846,103,844,454,600
**TOTAL PLOTS**	16,041
**NON ZERO PLOTS**	5,069
**TOTAL POPULATION ACRES**	54,002,539

Example 4.9. Estimate number of seedlings on timberland (seedlings) with sampling error.

```
SELECT eval_grp,
       SUM(estimate_by_estn_unit.estimate) estimate,
       CASE
         WHEN SUM(estimate_by_estn_unit.estimate) > 0 THEN
          round(sqrt(SUM(estimate_by_estn_unit.var_of_estimate)) /
                SUM(estimate_by_estn_unit.estimate) * 100,
                3)
         ELSE
          0
       END AS se_of_estimate_pct,
       SUM(estimate_by_estn_unit.var_of_estimate) var_of_estimate,
       SUM(estimate_by_estn_unit.total_plots) total_plots,
       SUM(estimate_by_estn_unit.non_zero_plots) non_zero_plots,
       SUM(estimate_by_estn_unit.total_population_area_acres) total_population_acres
  FROM (SELECT pop_eval_grp_cn,
               eval_grp,
               estn_unit_cn,
               sum(nvl(ysum_hd, 0) * phase_1_summary.expns) estimate,
               SUM(phase_1_summary.n_h) total_plots,
               SUM(phase_2_summary.number_plots_in_domain) domain_plots,
               SUM(phase_2_summary.non_zero_plots) non_zero_plots,
               total_area * total_area / SUM(phase_1_summary.n_h) *
               ((SUM(w_h * phase_1_summary.n_h *
                     (((nvl(ysum_hd_sqr, 0) / phase_1_summary.n_h) -
                     ((nvl(ysum_hd, 0) / phase_1_summary.n_h) *
                     (nvl(ysum_hd, 0) / phase_1_summary.n_h))) /
                     (phase_1_summary.n_h - 1)))) +
               1 / SUM(phase_1_summary.n_h) *
               (SUM((1 - w_h) * phase_1_summary.n_h *
                     (((nvl(ysum_hd_sqr, 0) / phase_1_summary.n_h) -
                     ((nvl(ysum_hd, 0) / phase_1_summary.n_h) *
                     (nvl(ysum_hd, 0) / phase_1_summary.n_h))) /
                     (phase_1_summary.n_h - 1))))) var_of_estimate,
               total_area total_population_area_acres
          FROM (SELECT peg.eval_grp,
                       peg.cn pop_eval_grp_cn,
                       psm.estn_unit_cn,
                       psm.expns,
                       psm.cn pop_stratum_cn,
                       p1pointcnt /
                       (SELECT SUM(strs.p1pointcnt)
                          FROM pop_stratum strs
                         WHERE strs.estn_unit_cn = psm.estn_unit_cn) w_h,
                       (SELECT SUM(strs.p1pointcnt)
                          FROM pop_stratum strs
                         WHERE strs.estn_unit_cn = psm.estn_unit_cn) n_prime,
                       p1pointcnt n_prime_h,
                       (SELECT SUM(eu_s.area_used)
                          FROM pop_estn_unit eu_s
                         WHERE eu_s.cn = psm.estn_unit_cn) total_area,
                       psm.p2pointcnt n_h
                  FROM pop_estn_unit peu,
                       pop_stratum    psm,
                       pop_eval       pev,
                       pop_eval_grp   peg,
                       pop_eval_typ   pet
```

```
          WHERE peu.cn = psm.estn_unit_cn
            AND pev.cn = peu.eval_cn
            AND pet.eval_cn = pev.cn
            AND pet.eval_grp_cn = peg.cn
            AND pet.eval_typ = 'EXPVOL' -- specify the appropriate expansion
            AND peg.eval_grp = 272003 -- the desired evaluation group must be specified
         ) phase_1_summary,
        (SELECT pop_stratum_cn,
                SUM(y_hid_adjusted) ysum_hd,
                SUM(y_hid_adjusted * y_hid_adjusted) ysum_hd_sqr,
                COUNT(*) number_plots_in_domain,
                SUM(decode(y_hid_adjusted, 0, 0, NULL, 0, 1)) non_zero_plots
           FROM (SELECT psm.cn pop_stratum_cn,
                        p.cn plt_cn,
                        SUM(s.tpa_unadj * adj_factor_micr) y_hid_adjusted
                                                -- expression from ref_pop_attribute table
                   FROM cond                c,
                        plot                p,
                        seedling            s,
                                  -- seedling table must be included for seedling level estimates
                        pop_plot_stratum_assgn ppsa,
                        pop_stratum         psm,
                        pop_estn_unit       peu,
                        pop_eval            pev,
                        pop_eval_grp        peg,
                        pop_eval_typ        pet
                  WHERE p.cn = c.plt_cn
                    AND pet.eval_typ = 'EXPVOL'
                    AND s.plt_cn = c.plt_cn
                    AND s.condid = c.condid
                    AND c.cond_status_cd = 1
                    AND c.reservcd = 0
                    AND c.siteclcd IN (1, 2, 3, 4, 5, 6)
                                  -- additional where_clause from ref_pop_attribute table
                    AND ppsa.plt_cn = p.cn
                    AND ppsa.stratum_cn = psm.cn
                    AND peu.cn = psm.estn_unit_cn
                    AND pev.cn = peu.eval_cn
                    AND pet.eval_cn = pev.cn
                    AND pet.eval_grp_cn = peg.cn
                    AND peg.eval_grp = 272003 -- the desired evaluation group must be specified
                  GROUP BY psm.cn, p.cn)
          GROUP BY pop_stratum_cn) phase_2_summary
     WHERE phase_1_summary.pop_stratum_cn =
        phase_2_summary.pop_stratum_cn(+)
     GROUP BY pop_eval_grp_cn,
          eval_grp,
          estn_unit_cn,
          phase_1_summary.total_area) estimate_by_estn_unit
GROUP BY pop_eval_grp_cn, eval_grp
```

Produces the following estimate of number of live seedlings on timberland with sampling error:

**EVAL GRP**	272003
**ESTIMATE**	37,141,783,495
**SE OF ESTIMATE PCT**	1.8
**VAR OF ESTIMATE**	455,665,600,805,109,000
**TOTAL PLOTS**	16,041
**NONZERO PLOTS**	4,304
**TOTAL POPULATION ACRES**	54,002,539

5. Restricting the attribute of interest to a smaller subset of the population

The estimation procedures presented in examples 4.1 through 4.9 can all be modified to restrict the estimation to a subset, referred to as the domain of interest. An example of a domain would be only sawtimber stands on publicly owned timberland. In effect, the attributes identified in the REF_POP_ATTRIBUTE table are a combination of an attribute (e.g., area, number of trees, volume, number of seedlings) and a domain (e.g., forest land, timberland, ownership, growing-stock trees). The attribute of interest is defined in the REF_POP_ATTRIBUTE.EXPRESSION and the domain of interest is defined by REF_POP_ATTRIBUTE.WHERE_CLAUSE. In example 4.2, the attribute of interest is area, and the domain of interest is restricted to timberland only. In example 4.3, the attribute of interest is number of trees, and the domain of interest is restricted to live trees on forest land with diameters 1 inch and larger. In example 4.4, the attribute of interest is number of seedlings, and the domain of interest is restricted to timberland. In example 4.5, the attribute of interest is volume of growing-stock, and the domain of interest is restricted to timberland.

A word of caution when working with periodic data – not all lands and all attributes were sampled in periodic inventories. In some States, only productive, non-reserved lands were sampled in periodic inventories. So, applying estimation of number of trees to all forest land in older periodic inventories will appear to work, but trees were only measured on timberland, so the estimates will only reflect the trees on timberland. Also, in many periodic inventories, seedlings were not tallied.

In the next example, the domain of interest in example 4.3 is further restricted to a specific species (SPCD = 129, eastern white pine), diameter (DIA ≥20, trees 20 inches and larger), and ownership (OWNGRPCD = 40, private owners only). The boxed lines have been added to the procedure. The procedure now provides an estimate of the total number of live eastern white pine, 20 inches and larger on privately owned forest land.

Example 4.10 Estimate number of live eastern white pine trees 20 inches and larger on privately owned forest land (trees).

```
SELECT SUM(psm.expns * t.tpa_unadj *
        decode(dia,
             NULL,
             adj_factor_subp,
             decode(least(dia, 5 - 0.001),
                  dia,
                  adj_factor_micr,
                  decode(least(dia,
                           nvl(macro_breakpoint_dia, 9999) - 0.001),
                       dia,
                       adj_factor_subp,
                       adj_factor_macr)))) estimate -- expression from ref_pop_attribute table
 FROM cond                    c,
     plot                     p,
     tree                     t, -- tree table must be included for tree level estimates
     pop_plot_stratum_assgn ppsa,
     pop_stratum              psm,
     pop_estn_unit            peu,
     pop_eval                 pev,
     pop_eval_grp             peg,
     pop_eval_typ             pet
 WHERE p.cn = c.plt_cn
  AND pet.eval_typ = 'EXPVOL'
  AND t.plt_cn = c.plt_cn
  AND t.condid = c.condid
  AND c.cond_status_cd = 1
 AND t.statuscd = 1
 AND t.dia >= 1.0 -- additional where_clause from ref_pop_attribute table
 AND t.spcd = 129
 AND t.dia >= 20.0
 AND c.owngrpcd = 40 -- user-defined additional where_clause
 AND ppsa.plt_cn = p.cn
 AND ppsa.stratum_cn = psm.cn
 AND peu.cn = psm.estn_unit_cn
 AND pev.cn = peu.eval_cn
 AND pev.cn = pet.eval_cn
 AND pet.eval_grp_cn = peg.cn
 AND peg.eval_grp = 272003 -- the desired evaluation group must be specified
```

Produces the following estimate of total number of live eastern white pine, 20 inches and larger on privately owned forest land:

ESTIMATE
519,317

Adding the same restrictions to the where clause in example 4.8 provides the following output:

**EVAL GRP**	272003
**ESTIMATE**	519,317
**SE OF ESTIMATE PCT**	25.1
**VAR OF ESTIMATE**	17,051,491,226
**TOTAL PLOTS**	16,041
**NON ZERO PLOTS**	20
**TOTAL POPULATION ACRES**	54,002,539

The estimated 519,317 eastern white pine trees, 20 inches and larger on privately owned forest land has a sample error of 25.1 percent. Live eastern white pine 20 inches or larger on private forest land were observed on a total of 20 plots in the State.

6. Changing the attribute of interest with user-defined criteria

Users can define condition level attributes of interest. The standard condition level attributes of interest are sampled land area and all land area (expressed in acres). Sampled land area (adjusted for denied access and hazardous conditions that were not sampled) is the one used for nearly all standard FIA tables that report area estimates. All land area (where denied access and hazardous are considered part of the sample) is only used in estimation that treats denied access (plots on land where field crews were unable to obtain the owner's permission to measure the plot) and hazardous (conditions that were deemed too hazardous to measure the plots) as part of the sample attribute of interest. Most of the other condition level variables that FIA observes are typically used to categorize the condition, and are most often applied as restrictions on the population in defining the domain, and do not lend themselves as an attribute of interest. For example, BALIVE (the basal area of live trees 1 inch diameter and larger) is mainly used to categorize forest land area rather than as an attribute of interest in population level estimation. Users are more interested in knowing how many acres of forest land meets some basal area requirement (say between 50 and 100 square feet per acre), rather than the total basal area of forest land in a State.

An example of a user-defined condition level attribute of interest, for which an estimate of a total might be of interest, would be total land value (see Example 4.11). Here the user would supply a function that assigns value ($ per acre) to forest land, based on attributes in FIADB. As an example, we use a very arbitrary function of site index and basal area of live tree – value per acre = 1000 + (site index x 3) + (basal area x 4), and limit the domain of interest to only private timberland. Modifying example 1 produces the following script and estimate of total value. Since the function is a condition level value per acre, it is simply included in the expression as a multiplication factor, and the domain restriction (private timberland) is added to the where clause. The sections that have been added to example 4.2 are in boxes. The same modifications were added to example 4.7 to produce the estimates with sampling errors.

Example 4.11 Estimated dollar value of private timberland (user defined function).

```
SELECT SUM(psm.expns * c.condprop_unadj *
        decode(c.prop_basis,
            'MACR',
            psm.adj_factor_macr,
            psm.adj_factor_subp) -- expression from ref_pop_attribute table
        * (1000 + c.sicond * 3 + c.balive * 4) -- user-defined value function
        ) estimate
 FROM cond                  c,
    plot                    p,
    pop_plot_stratum_assgn  ppsa,
    pop_stratum             psm,
    pop_estn_unit           peu,
    pop_eval                pev,
    pop_eval_grp            peg,
    pop_eval_typ            pet
 WHERE p.cn = c.plt_cn
  AND pet.eval_typ = 'EXPCURR'
  AND c.cond_status_cd = 1
  AND c.reservcd = 0
  AND c.siteclcd IN (1, 2, 3, 4, 5, 6) -- additional where_clause from ref_pop_attribute table
  AND c.owngrpcd = 40 -- user-defined additional where_clause
  AND ppsa.plt_cn = p.cn
  AND ppsa.stratum_cn = psm.cn
  AND peu.cn = psm.estn_unit_cn
  AND pev.cn = peu.eval_cn
  AND pev.cn = pet.eval_cn
  AND pet.eval_grp_cn = peg.cn
  AND peg.eval_grp = 272003 -- the desired evaluation group must be specified
```

Produces the following estimate only from above example:

ESTIMATE
10,156,384,067

And the same modification to example 4.7 produces the following estimate with sampling errors:

EVAL GRP	272003
ESTIMATE	10,156,384,067
SE OF ESTIMATE PCT	1.4
VAR OF ESTIMATE	18,850,461,684,117,200
TOTAL PLOTS	16,041
NON ZERO PLOTS	2,290
TOTAL POPULATION ACRES	54,002,539

Based on this function, the estimated total value of private timberland in the State is 10.1 billion dollars. This value function is used only as an example, any type of user defined function that assigns quantities, such as value ($ per acre), wildlife population level (animals per acre), productivity (yield per acre), or carbon sequestration potential (tons per acre) could be used as long as it is a function of data items in the FIADB, and/or data attributes from other sources that can be linked to FIA plots.

7. Estimates of change over time on the standard 4-subplot fixed area plot

A number of the attributes described in the REF_POP_ATTRIBUTE table are related to change over time and are based on computed attributes that utilize data from two points in time from the same plot. The attributes identified by values 25-44 (e.g., net growth of all live on forestland represented by 25) of REF_POP_ATTRIBUTE.ATTRIBUTE_NBR are the standard growth, removals and mortality attributes that FIA presents in its reports. The computation of these values as presented in the previous section will provide estimates of these change attributes; however, all estimation is done through the observations made and recorded at the second measurement of the plot. Users often wish (1) to obtain estimates that reflect changes in attributes over the remeasurement of the plot that go beyond these attributes, (2) to classify these standard estimates and other estimates by attributes from the previous measurement, or (3) to cross classify them by changes in various attributes over time. Examples of these types of estimations are:

- Breakdowns of change in area over time by past and current land use, forest type, or other condition attributes.
- Number of trees on forest land that changed to nonforest land.
- Removals of trees on forest land of a specific forest type that changed to a different forest type after removals.
- Mortality of trees that were in a specific diameter range in the previous measurement.
- Change in the number of seedlings per acre over time for a specific forest type.

The estimation of these and many other change attributes require properly selecting the appropriate set of plots that were measured at both points in time and linking data from these two measurements.

Prior to 1999, FIA used periodic inventories with different plot designs. Since 1999, the new annual inventory uses a national standard, 4-subplot fixed area plot design. The change estimation procedures described here are applicable to all plots measured at least twice in the annual inventory, but may not be appropriate for change estimation between periodic and annual inventories.

7.1 Selecting an appropriate set of plots (evaluation) for change estimations

For change estimation, select an evaluation that consists of only remeasured plots, evaluations used for growth, removals, and mortality estimation. These growth-removals-mortality (GRM) evaluations can be identified by either of the following restrictions in the where clause:

and pop_eval.cn = pop_eval_grp.eval_cn_for_expgrow,

or

and pop_eval_typ.eval_grp_cn = pop_eval_grp.cn
and pop_eval_typ.eval_typ = 'EXPGROW'

Either of these statements will restrict the sample plots to only those used in the estimation of growth: only the set of plots that have been measured at two points in time. In the examples we continue linking to evaluations through the POP_EVAL_TYP table (second example).

7.2 Linking tree level data to past condition data

In the following examples, we demonstrate how to produce a tree-level estimate (net growth of all live trees on forest land), and then link it to conditions at two points in time (past and current) to produce a table that breaks down the estimate by condition-level attributes and the two points in time.

First we begin with the script that produces an estimate of total net growth of all live trees on forest land for the 2007 Minnesota inventory. The evaluation used in this estimate (pop_eval.evalid = 270703) consists of plots measured in 1999, 2000, 2001, and 2002 that were remeasured in 2004, 2005, 2006, and 2007, respectively.

Example 4.12 Estimate net growth of all live trees on forest land (cubic feet per year).

```
SELECT SUM(psm.expns * t.tpagrow_unadj * fgrowcfal *
        decode(dia,
            null,
            adj_factor_subp,
            decode(least(dia, 5 - 0.001),
                dia,
                adj_factor_micr,
                decode(least(dia,
                        nvl(macro_breakpoint_dia, 9999) - 0.001),
                    dia,
                    adj_factor_subp,
                    adj_factor_macr)))) estimate -- expression from ref_pop_attribute table
 FROM cond                    c,
     plot                     p,
     tree                     t, -- tree table must be included for tree level estimates
     pop_plot_stratum_assgn ppsa,
     pop_stratum              psm,
     pop_estn_unit            peu,
     pop_eval                 pev,
     pop_eval_grp             peg,
     pop_eval_typ             pet
WHERE p.cn = c.plt_cn
  AND pet.eval_typ = 'EXPGROW'
  AND t.plt_cn = c.plt_cn
  AND t.condid = c.condid -- additional where_clause from ref_pop_attribute table
  AND ppsa.plt_cn = p.cn
  AND ppsa.stratum_cn = psm.cn
  AND peu.cn = psm.estn_unit_cn
  AND pev.cn = peu.eval_cn
  AND pev.cn = pet.eval_cn
  AND pet.eval_grp_cn = peg.cn
  AND peg.eval_grp = 272007 -- the desired evaluation group must be specified
```

The example above produces the following estimate of total net growth of all live trees on forest land:

427,200,491 cubic feet per year

We then modified this example to link not only to the condition record at the current (second) measurement, but also to the condition record at the previous (first) measurement by using the attribute TREE.PREVCOND to link each tree record to its previous condition. We also added a

group by clause to produce the estimates broken down by values of the condition level attributes COND_STATUS_CD (condition status code) and STDSZCD (stand-size class code) at both points in time. This procedure is shown in example 4.13, which was created by adding the bold sections to example 4.12.

Example 4.13 Estimate net growth of all live on forest land (cubic feet per year) by condition status and stand size at two points in time.

```
SELECT c_past.cond_status_cd past_cond_status_cd,
     c_past.stdszcd past_stdszcd,
     c.cond_status_cd current_cond_status_cd,
     c.stdszcd current_stdszcd,
     SUM(psm.expns * t.tpagrow_unadj * fgrowcfal *
        decode(dia,
             null,
             adj_factor_subp,
             decode(least(dia, 5 - 0.001),
                  dia,
                  adj_factor_micr,
                  decode(least(dia,
                            nvl(macro_breakpoint_dia, 9999) - 0.001),
                       dia,
                       adj_factor_subp,
                       adj_factor_macr)))) estimate -- expression from ref_pop_attribute table
 FROM cond                      c,
     cond                       c_past, --past condition is added
     plot                       p,
     tree                       t, -- tree table must be included for tree level estimates
     pop_plot_stratum_assgn ppsa,
     pop_stratum                psm,
     pop_estn_unit              peu,
     pop_eval                   pev,
     pop_eval_grp               peg,
     pop_eval_typ               pet
 WHERE p.cn = c.plt_cn
  AND pet.eval_typ = 'EXPGROW'
  AND t.plt_cn = c.plt_cn
  AND t.condid = c.condid -- additional where_clause from ref_pop_attribute table
  AND ppsa.plt_cn = p.cn
  AND ppsa.stratum_cn = psm.cn
  AND peu.cn = psm.estn_unit_cn
  AND pev.cn = peu.eval_cn
  AND pev.cn = pet.eval_cn
  AND pet.eval_grp_cn = peg.cn
  AND peg.eval_grp = 272007 -- the desired evaluation group must be specified
  AND c_past.plt_cn = p.prev_plt_cn
           -- links to only those conditions at previous measurement of plot
  AND c_past.condid = t.prevcond -- links trees to their past condition
 group by c_past.cond_status_cd,
       c_past.stdszcd,
       c.cond_status_cd,
       c.stdszcd
```

Example 4.13 produces the following estimates of total net growth of all live trees on forest land broken down by past and current COND_STATUS_CD and STDSZCD values.

PAST_COND_ STATUS_CD	PAST_ STDSZCD	CURRENT_COND_ STATUS_CD	CURRENT_ STDSZCD	ESTIMATE
1	1	1	1	81,494,163.3
1	1	1	2	-1,056,519.2
1	1	1	3	-6,077,491.9
1	1	1	5	-4,520,213.8
1	1	2		708,394.8
1	1	5		0.0
1	2	1	1	24,639,163.2
1	2	1	2	121,373,610.5
1	2	1	3	-298,122.7
1	2	1	5	-1,358,131.3
1	2	2		-720,502.7
1	2	3		-41,231.9
1	3	1	1	4,596,722.9
1	3	1	2	29,398,997.6
1	3	1	3	38,089,804.3
1	3	1	5	78,764.7
1	3	2		380,739.8
1	3	3		0.0
1	3	4		-327,337.8
1	5	1	1	1,591,344.2
1	5	1	2	579,855.3
1	5	1	3	135,054.2
1	5	1	5	11,488.4
1	5	2		2,210.0
1	5	5		0.0
2		1	1	67,569,968.1
2		1	2	45,417,363.4
2		1	3	11,180,894.0
2		1	5	557,059.5
2		2		0.0
3		1	1	4,285,796.0
3		1	2	1,838,167.0
3		1	3	1,187,875.3
4		1	1	5,868,590.9
4		1	2	212,902.8
4		1	3	401,112.0
5		1	1	0.0

The following tabulation of estimated net growth on forest land by condition status code and stand-size class at the two points in time can be made from the example 4.13 results. Note that we have added the code labels to the row and column headings, and each cell in the tabulation is the appropriate value from example 4.13.

Estimated total net growth of all live trees on forest land broken down by past and current condition status code and stand-size class, Minnesota, 2007 (cubic feet per year).

		CURRENT_COND_STATUS_CD									Total
		1 Forest land CURRENT_STDSZCD				Total on Forest land	2 Nonforest land	3 Noncensus water	4 Census water	5 Nonsampled	
PAST_COND_ STATUS_CD	PAST_STDSZCD	1 Large diameter	2 Medium diameter	3 Small diameter	5 Nonstocked						
1 Forest land	1 Large diameter	81,494,163.3	-1,056,519.2	-6,077,491.9	-4,520,213.8	69,839,938.4	708,394.8			0.0	70,548,333.2
	2 Medium diameter	24,639,163.2	121,373,610.5	-298,122.7	-1,358,131.3	144,356,519.6	-720,502.7	-41,231.9			143,594,785.1
	3 Small diameter	4,596,722.9	29,398,997.6	38,089,804.3	78,764.7	72,164,289.4	380,739.8	0.0	-327,337.8		72,217,691.4
	5 Nonstocked	1,591,344.2	579,855.3	135,054.2	11,488.4	2,317,742.0	2,210.0			0.0	2,319,952.0
Total on forest land		112,321,393.5	150,295,944.1	31,849,243.9	-5,788,092.1	288,678,489.4	370,842.0	-41,231.9	-327,337.8	0.0	288,680,761.7
2 Nonforest land		67,569,968.1	45,417,363.4	11,180,894.0	557,059.5	124,725,284.9	0.0				124,725,284.9
3 Noncensus water		4,285,796.0	1,838,167.0	1,187,875.3		7,311,838.3					7,311,838.3
4 Census water		5,868,590.9	212,902.8	401,112.0		6,482,605.6					6,482,605.6
5 Nonsampled		0.0				0.0					0.0
Total net growth		190,045,748.4	197,764,377.3	44,619,125.1	-5,231,032.6	427,198,218.2	370,842.0	-41,231.9	-327,337.8	0.0	427,200,490.5

7.3 The SUBP_COND_CHNG_MTRX (CMX) table

The SUBP_COND_CHNG_MTRX (CMX) table was added in the FIADB version 4.0 to facilitate the tracking of area change for the annual inventory. Under this design, a plot measures area change by tracking the movement in condition boundaries within the area of the four subplots. Figure 7 shows what can happen on a plot when a condition boundary (in this case the edge of a beaver pond) moves over time. Beaver activity raised the level of the pond, increasing the pond area and converting some of the forest land to water. The same kind of changes can occur from any number of human-caused events such as timber harvesting, land clearing or road construction, or natural events such as fire, storms, or insect attacks.

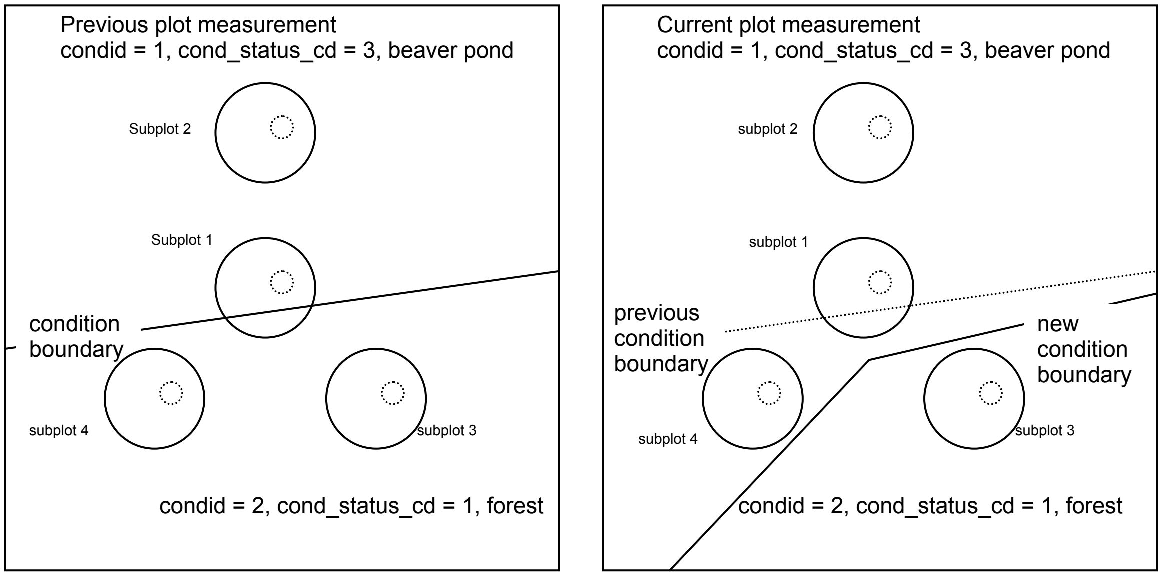

Figure 7. Example plot layout where condition boundaries changed between previous (left panel) and current plot measurements (right panel). The solid circles are the subplots and the smaller dashed circles are the microplots.

It is important to remember that condition boundaries are not just defined along changes in condition status code, but also may occur within forest land. The following tabulation shows how the area change information in figure 7 would be recorded in the CMX table

CMX table data for figure 7

SUBP	SUBPTYP	CONDID	PREVCOND	SUBPTYP_PROP_CHNG
1	1	1	1	.8000
1	1	1	2	.2000
1	2	1	1	1.000
2	1	1	1	1.000
2	2	1	1	1.000
3	1	2	2	1.000
3	2	2	2	1.000
4	1	1	2	1.000
4	2	1	2	1.000

The CMX table tracks the change in condition areas for each of the four subplots (SUBPTYP = 1) and each of the four microplots (SUBPTYP = 2) on this plot. In inventories where the macroplot is used there would also be entries for each macroplot (SUBPTYP = 3). The attribute PROP_BASIS in the COND table identifies how area estimation was conducted for each plot, on the basis of either the macroplot or the subplot. In this example area estimation (and thus area change estimation) is based on the subplot information, not the macroplot. Area estimation is typically based on the largest area sampled (macroplot in States where it is measured, otherwise the subplot) and not on the microplot. Area and area change estimation based on the microplot is only appropriate with another estimate solely collected on the microplot such as number of trees or biomass in trees <5 inches diameter at breast height. The examples of change presented here are based on the subplot, but could easily be modified to obtain estimates based on the microplots.

In the example shown in figure 7, the CMX table has two entries where SUBPTYP = 1 and SUBP = 1. The first entry indicates that 80 percent of the subplot area was in condition 1 (water) at both measurements, and the second entry indicates 20 percent of the subplot area changed from forest to water. For the other three subplots and all four microplots, only one record exists, indicating that the entire subplot or microplot either stayed in the same condition (subplots and microplots 2 and 3) or the entire area changed from one condition to another (subplot and microplot 4). For this remeasured plot, change based on the four subplots is water to water 45 percent, forest to water 30 percent, and forest to forest 25 percent; change based on the four microplots is water to water 50 percent, forest to water 25 percent, and forest to forest 25 percent. The following section presents SQL script that produces these estimates.

7.4 Using the CMX table to estimate area change between two measurements

The estimation of area change over time requires linking past and current conditions through the CMX table to determine the portion of plot area that transitioned from conditions observed at time 1 to those observed at time 2 (methods applicable only between two measurements in the annual inventory). As in examples 4.12 and 4.13, select an evaluation that consists of only remeasured plots. In the examples that follow, we show how to create area change estimates that go with the net growth of all live trees on forest land as obtained from example 4.13.

We begin by modifying the script that produces the estimate of area of forest land so that it uses the net growth evaluation EXPGROW rather than the area evaluation EXPCURR that is standard for area estimations. Example 4.14 shows this modification in bold.

Example 4.14 Estimate area of forest land (acres) based on the net growth evaluation.

```
SELECT SUM(psm.expns * c.condprop_unadj *
         decode(c.prop_basis,
              'MACR',
              psm.adj_factor_macr,
              psm.adj_factor_subp) -- expression from ref_pop_attribute table
         ) estimate
  FROM cond                     c,
       plot                     p,
       pop_plot_stratum_assgn ppsa,
       pop_stratum              psm,
       pop_estn_unit            peu,
       pop_eval                 pev,
       pop_eval_grp             peg,
       pop_eval_typ             pet
 WHERE p.cn = c.plt_cn
   and pet.eval_typ = 'EXPGROW'
   AND c.cond_status_cd = 1 -- additional where_clause from ref_pop_attribute table
   AND ppsa.plt_cn = p.cn
   AND ppsa.stratum_cn = psm.cn
   AND peu.cn = psm.estn_unit_cn
   AND pev.cn = peu.eval_cn
   AND pev.cn = pet.eval_cn
   AND pet.eval_grp_cn = peg.cn
   AND peg.eval_grp = 272007 -- the desired evaluation group must be specified
```

The remeasured plots (12,280 plots) associated with EXPGROW produce an area estimate of 16,962,397.2 acres of forest land versus 16,723,532.5 provided by EXCURR using all plots (17,855 plots). Both estimates of forest area are valid; however, only the one based on the remeasurement sample can be broken down into two points in time.

To estimate area change over time, the script has been further modified to link past and current condition records through the CMX table. This table has entries for every subplot on a remeasured plot and stores the proportion of the area of each subplot by the two points in time in the attribute CMX.SUBTYP_PROP_CHNG. Example 4.15 shows the script that produces the area change estimates that go with the net growth estimates produced in example 4.13. Again, changes and additions from example 4.14 are shown in bold. Line numbers are for reference purposes.

Example 4.15 Estimate area change (acres) by condition status and stand size at two points in time, Minnesota, time 1 from 1999-2002 and time 2 from 2003-2007

**1**	SELECT **c_past.cond_status_cd past_cond_status_cd,**
**2**	**c_past.stdszcd past_stdszcd,**
**3**	**c.cond_status_cd current_cond_status_cd,**
**4**	**c.stdszcd current_stdszcd,**
**5**	SUM(psm.expns * **CMX.subptyp_prop_chng / 4 ***
6	decode(c.prop_basis,
7	'MACR',
8	psm.adj_factor_macr,
9	psm.adj_factor_subp) -- *expression from ref_pop_attribute table*
10	) estimate,
**11**	**count(*) COUNT,**
**12**	**SUM(cmx.subptyp_prop_chng / 4) plot_area**
13	FROM cond c,
14	plot p,
15	pop_plot_stratum_assgn ppsa,
16	pop_stratum psm,
17	pop_estn_unit peu,
18	pop_eval pev,
19	pop_eval_typ pet,
20	pop_eval_grp peg,
**21**	**cond c_past,**
**22**	**subp_cond_chng_mtrx cmx**
23	WHERE p.cn = c.plt_cn
24	AND pet.eval_typ = 'EXPGROW'
**25**	**AND (c.cond_status_cd = 1 or c_past.cond_status_cd = 1)**
26	AND ppsa.plt_cn = p.cn
27	AND ppsa.stratum_cn = psm.cn
28	AND peu.cn = psm.estn_unit_cn
29	AND pev.cn = peu.eval_cn
30	AND pev.cn = pet.eval_cn
31	AND pet.eval_grp_cn = peg.cn
32	AND peg.eval_grp = 272007 -- *the desired evaluation group must be specified*
**33**	**AND p.prev_plt_cn = c_past.plt_cn**
**34**	**AND cmx.prev_plt_cn = c_past.plt_cn**
**35**	**AND cmx.prevcond = c_past.condid**
**36**	**AND cmx.condid = c.condid**
**37**	**AND ((cmx.subptyp = 3 and c.prop_basis = 'MACR') or**
**38**	**(cmx.subptyp = 1 and c.prop_basis = 'SUBP'))**
**39**	**group by c_past.cond_status_cd,**
**40**	**c_past.stdszcd,**
**41**	**c.cond_status_cd,**
**42**	**c.stdszcd**

Example 4.15 can be used as a template to create almost any cross tabulation of past and current area estimates based on a remeasured set of plots. The following changes (bold sections) were made to example 4.14 to facilitate the estimation of area change:

- Line 21 – The table **COND** with the alias **C_PAST** was added to the list of tables to be joined. This provides the condition level attributes for the past (time 1) measurement of the plot.

- Line 22 – The table **SUBP_COND_CHG_MTRX** with the alias **CMX** was added to the list of tables to be joined. This table provides the link between past (time 1) and current (time 2) conditions at the subplot level. Each entry in this table defines the portion (0-1) of the subplot, microplot or macroplot that was observed in a condition at time 2 and observed in a condition at time 1. For a subplot that was entirely in a single condition at both times, there will only be one entry for the subplot, with CMX.SUBPTYP_PROP_CHNG = 1.0. For a subplot that was mapped to be 40 percent in one condition and 60 percent in another condition at both times with no change in boundary, there will be two entries for the subplot, one with CMX.SUBPTYP_PROP_CHNG = 0.4 and the other with CMX.SUBPTYP_PROP_CHNG = 0.6. For subplots where boundaries have changed, there will be entries that account for all the pieces of the subplot area with the total value of CMX.SUBPTYP_PROP_CHNG adding to 1.0.

- Lines 1-4 and 39-42 – As in example 4.13, past and current condition status and stand-size class codes **(group by c_past.cond_status_cd, c_past.stdszcd, c.cond_status_cd, c.stdszcd)** were grouped to obtain estimate breakdowns by these attributes.

- Line 5 – **c.condprop_unadj** (the total plot condition proportions that are within a specific condition) was replaced with **cmx.subptyp_prop_chng / 4** (the subplot condition proportion divided by the number of subplots in the plot). The division by 4 is required because the **CMX** table tracks area at the subplot level (4 subplots per plot).

- Line 25 – The restrictions were changed in the where clause from **AND c.cond_status_cd = 1** to **AND (c.cond_status_cd = 1 or c_past.cond_status_cd = 1),** to select conditions that were forest in at least one of the measurements, not just the current measurement. This query tracks the area of land that moves in and out of forest, as well as changes in stand-size class on land that remains forest.

- Lines 33-38 – These additions to the where clause provide the proper links to the **C_PAST** and **CMX** tables that were added to the table list. Line 33 (**AND p.prev_plt_cn = c_past.plt_cn)** matches the past and current condition records to the same plot, and lines 34-38 provide the other restrictions that link the appropriate conditions at the two measurements through the **CMX** table. Lines 37 and 38 ensure that in inventories where area estimates are based on the macroplot observations, the area change estimates are based on the macroplot observations, and in all other cases the estimates are based on the subplot observations.

- Lines 11 and 12 – **count(*) COUNT** and **SUM(CMX.subptyp_prop_chng / 4) PLOT_AREA** provide two additional summary attributes along with the area estimates. **COUNT** is the total number of subplot pieces that is tracked in the estimation. **PLOT_AREA** is the total portion of plots that is tracked in the estimation.

Example 4.15 produces the following estimates of total area (ESTIMATE), total number of subplots (COUNT), and total portion of plots (PLOT_AREA) broken down by past and current COND_STATUS_CD and STDSZCD values, for land that was forest at measurement time 1, measurement time 2, or both.

PAST_COND_ STATUS_CD	PAST_ STDSZCD	CURRENT_ STATUS_CD	CURRENT_ STDSZCD	ESTIMATE	COUNT	PLOT_AREA
1	1	1	1	3,631,160.4	3208	767.8
1	1	1	2	291,277.3	274	63.1
1	1	1	3	390,763.5	360	83.0
1	1	1	5	58,700.4	53	12.0
1	1	2		70,387.0	117	15.2
1	1	3		3,961.3	10	0.8
1	1	4		2,892.6	9	0.6
1	1	5		2,289.7	2	0.5
1	2	1	1	786,401.0	709	167.1
1	2	1	2	4,648,293.5	4160	996.0
1	2	1	3	620,036.7	571	132.4
1	2	1	5	46,356.9	46	10.2
1	2	2		84,928.1	133	18.8
1	2	3		1,990.6	6	0.4
1	2	4		895.2	1	0.2
1	3	1	1	158,110.2	151	32.5
1	3	1	2	648,108.5	604	138.3
1	3	1	3	4,243,065.9	3884	934.6
1	3	1	5	61,623.3	56	13.1
1	3	2		98,616.9	126	21.4
1	3	3		12,348.1	11	2.1
1	3	4		4,707.5	4	1.0
1	5	1	1	16,820.1	18	3.7
1	5	1	2	18,273.1	20	4.2
1	5	1	3	95,244.4	94	21.5
1	5	1	5	61,597.5	59	14.2
1	5	2		55,411.0	53	11.9
1	5	3		549.8	1	0.1
1	5	5		2,814.4	2	0.5
2		1	1	234,236.1	288	50.7
2		1	2	267,173.3	326	59.6
2		1	3	556,373.0	564	126.3
2		1	5	48,463.7	51	11.0
3		1	1	14,427.4	19	3.1
3		1	2	9,767.2	13	2.3
3		1	3	21,966.0	21	4.3
3		1	5	1,225.5	2	0.3
4		1	1	17,585.0	19	4.0
4		1	2	4,149.1	10	0.9
4		1	3	8,858.1	9	2.0
5		1	1	2,339.8	2	0.5

These results are used to produce the following tabulation of estimated change in forest area by condition status code and stand-size class at two points in time.

Estimated forest land area broken down by past and current condition status code and stand-size class, Minnesota, 2007 (acres). Includes lands classified as forest at either or both measurements. Based on plots first measured in 1999-2002 and remeasured in 2003-2007.

		CURRENT_COND_STATUS_CD									Total
		1 Forest land CURRENT_STDSZCD				Total Forest land	2 Nonforest land	3 Noncensus water	4 Census water	5 Nonsampled	
PAST_COND_ STATUS_CD	PAST_ STDSZCD	1 Large diameter	2 Medium diameter	3 Small diameter	5 Non-stocked						
1 Forest land	1 Large diameter	3,631,160.4	291,277.3	390,763.5	58,700.4	4,371,901.6	70,387.0	3,961.3	2,892.6	2,289.7	4,451,432.2
	2 Medium diameter	786,401.0	4,648,293.5	620,036.7	46,356.9	6,101,088.1	84,928.1	1,990.6	895.2		6,188,902.0
	3 Small diameter	158,110.2	648,108.5	4,243,065.9	61,623.3	5,110,907.8	98,616.9	12,348.1	4,707.5		5,226,580.4
	5 Nonstocked	16,820.1	18,273.1	95,244.4	61,597.5	191,935.1	55,411.0	549.8		2,814.4	250,710.3
Total forest land		4,592,491.7	5,605,952.3	5,349,110.6	228,278.0	15,775,832.6	309,343.1	18,849.8	8,495.3	5,104.1	16,117,624.8
2 Nonforest land		234,236.1	267,173.3	556,373.0	48,463.7	1,106,246.1					1,106,246.1
3 Noncensus water		14,427.4	9,767.2	21,966.0	1,225.5	47,386.1					47,386.1
4 Census water		17,585.0	4,149.1	8,858.1		30,592.1					30,592.1
5 Nonsampled		2,339.8				2,339.8					2,339.8
Total		4,861,080.0	5,887,041.9	5,936,307.6	277,967.2	16,962,396.8	309,343.1	18,849.8	8,495.3	5,104.1	17,304,189.0

The total current forest land area in the table above (16,962,396.8 acres) matches (within 1 acre) the results we obtained in example 4.14 (16,962,397.2 acres). The difference between these two estimates is simply the rounding error introduced by storing and computing condition proportions for each of the individual subplot sections in **cmx.subptyp_prop_chng** versus the total condition proportion in **c.condprop_unadj**. The total past forest land area in the tabulation above (16,117,624.8 acres) is based on the same remeasured plots and comes close, but does not match the 2003 estimate of forest land area (16,230,325.3 acres) one obtains when using example 4.14 and setting pet.eval_typ='EXPCURR' and pop_eval_grp.eval_grp = 272003.

The COUNT and PLOT_AREA values provide data users with the number of measurements associated with each estimate, giving users some information about the reliability of the estimates. For example, conditions that remained as large diameter (COND.STDSZCD equals 1) from time 1 to time 2 had an area estimate of 3,631,160.4 acres at time 2. From time 1 to time 2, 3,208 subplots or portions of subplots maintained their large diameter condition. These subplots or portions of subplots represent an area equivalent to 767.8 total plots. The estimates are based on a considerable number of observations. In contrast, if one is interested in tracking area of water (either census or noncensus water) that converts to or from forest land over time, estimates are based on far fewer observed changes. The estimated area that changed from water (COND.COND_STATUS_CD equals 3 or 4) to forest (COND.COND_STATUS_CD equals 1) is 77,978.2 acres, and the estimated change from forest to water is 27,345.1 acres. The water to forest change is based on observations from 93 subplots where at least a portion of the subplot was observed to change from water to forest. The total area of this observed change is equal to 16.8 plots. The change from forest to water estimate (27,345.1 acres) is based on 42 subplot observations over an area equivalent to 5.2 plots.

Example 4.16 presents sampling errors for the forest to water area change estimate. This script was created from the script presented in example 4.7 with modifications similar to those made in example 4.15. The bold sections indicate where changes were made. The addition of the following code to the where clause restricts the estimation to conditions that change from forest (c_past.cond_status_cd = 1) to water (c.cond_status_cd IN (3,4)):

AND (c.cond_status_cd IN (3,4) AND c_past.cond_status_cd = 1).

Further modifications to this example were made to produce estimates and sampling errors for the water to forest area change and for areas that remained as large diameter conditions as discussed in the previous paragraph. The results are presented in the tabulation that follows example 4.16. Users will note that the sampling errors for the estimates of forest to water and water to forest area change are quite high (29.2 percent and 18.4 percent, respectively) and the sampling error on conditions remaining large diameter is fairly low (2.9 percent). To obtain other area change and sampling error estimates, users should modify the where clause and eval_grp.

Example 4.16. Estimate area change from forest (cond_status_cd equals 1) to water (cond_status_cd equals 3 or 4) with sampling error. Based on the Minnesota 2007 remeasurement sample. Note the bold sections in this example indicate where changes in code from example 4.7 were made.

```
SELECT eval_grp,
    SUM(estimate_by_estn_unit.estimate) estimate,
    CASE
      WHEN SUM(estimate_by_estn_unit.estimate) > 0 THEN
       round(sqrt(SUM(estimate_by_estn_unit.var_of_estimate)) /
```

```
            SUM(estimate_by_estn_unit.estimate) * 100,
            3)
        ELSE
         0
      END AS se_of_estimate_pct,
      SUM(estimate_by_estn_unit.var_of_estimate) var_of_estimate,
      SUM(estimate_by_estn_unit.total_plots) total_plots,
      SUM(estimate_by_estn_unit.non_zero_plots) non_zero_plots,
      SUM(estimate_by_estn_unit.total_population_area_acres) total_population_acres
 FROM (SELECT pop_eval_grp_cn,
              eval_grp,
              estn_unit_cn,
              sum(nvl(ysum_hd, 0) * phase_1_summary.expns) estimate,
              SUM(phase_1_summary.n_h) total_plots,
              SUM(phase_2_summary.number_plots_in_domain) domain_plots,
              SUM(phase_2_summary.non_zero_plots) non_zero_plots,
              total_area * total_area / SUM(phase_1_summary.n_h) *
              ((SUM(w_h * phase_1_summary.n_h *
                  (((nvl(ysum_hd_sqr, 0) / phase_1_summary.n_h) -
                  ((nvl(ysum_hd, 0) / phase_1_summary.n_h) *
                  (nvl(ysum_hd, 0) / phase_1_summary.n_h))) /
                  (phase_1_summary.n_h - 1)))) +
              1 / SUM(phase_1_summary.n_h) *
              (SUM((1 - w_h) * phase_1_summary.n_h *
                  (((nvl(ysum_hd_sqr, 0) / phase_1_summary.n_h) -
                  ((nvl(ysum_hd, 0) / phase_1_summary.n_h) *
                  (nvl(ysum_hd, 0) / phase_1_summary.n_h))) /
                  (phase_1_summary.n_h - 1))))) var_of_estimate,
              total_area total_population_area_acres
        FROM (SELECT peg.eval_grp,
                     peg.cn pop_eval_grp_cn,
                     psm.estn_unit_cn,
                     psm.cn pop_stratum_cn,
                     psm.expns,
                     p1pointcnt /
                     (SELECT SUM(strs.p1pointcnt)
                        FROM pop_stratum strs
                       WHERE strs.estn_unit_cn = psm.estn_unit_cn) w_h,
                     (SELECT SUM(strs.p1pointcnt)
                        FROM pop_stratum strs
                       WHERE strs.estn_unit_cn = psm.estn_unit_cn) n_prime,
                     p1pointcnt n_prime_h,
                     (SELECT SUM(eu_s.area_used)
                        FROM pop_estn_unit eu_s
                       WHERE eu_s.cn = psm.estn_unit_cn) total_area,
                     psm.p2pointcnt n_h
                FROM pop_estn_unit peu,
                     pop_stratum       psm,
                     pop_eval          pev,
                     pop_eval_grp      peg,
                     pop_eval_typ      pet
               WHERE peu.cn = psm.estn_unit_cn
                 AND pev.cn = peu.eval_cn
                 AND pet.eval_cn = pev.cn
                 AND pet.eval_grp_cn = peg.cn
                 AND pet.eval_typ = 'EXPGROW' -- expansion factor tracking change
                 AND peg.eval_grp = 272007 -- desired evaluation group must be specified
              ) phase_1_summary,
```

```
        (SELECT pop_stratum_cn,
              SUM(y_hid_adjusted) ysum_hd,
              SUM(y_hid_adjusted * y_hid_adjusted) ysum_hd_sqr,
              COUNT(*) number_plots_in_domain,
              SUM(decode(y_hid_adjusted, 0, 0, NULL, 0, 1)) non_zero_plots
          FROM (SELECT psm.cn pop_stratum_cn,
                    p.cn plt_cn,
                    SUM(cmx.subptyp_prop_chng / 4 *
                       decode(c.prop_basis,
                            'MACR',
                            psm.adj_factor_macr,
                            psm.adj_factor_subp) -- expression for proportion of tracked plots
                       ) y_hid_adjusted
                 FROM cond                   c,
                    plot                     p,
                    pop_plot_stratum_assgn   ppsa,
                    pop_stratum              psm,
                    pop_estn_unit            peu,
                    pop_eval                 pev,
                    pop_eval_typ             pet,
                    pop_eval_grp             peg,
                    cond                     c_past,
                    subp_cond_chng_mtrx      cmx
                WHERE p.cn = c.plt_cn
                  AND pet.eval_typ = 'EXPGROW'
                  AND (c.cond_status_cd IN (3, 4) AND c_past.cond_status_cd = 1)
                       -- where clause tracking change
                  AND ppsa.plt_cn = p.cn
                  AND ppsa.stratum_cn = psm.cn
                  AND peu.cn = psm.estn_unit_cn
                  AND pev.cn = peu.eval_cn
                  AND pev.cn = pet.eval_cn
                  AND pet.eval_grp_cn = peg.cn
                  AND peg.eval_grp = 272007 -- desired evaluation group must be specified
                  AND p.prev_plt_cn = c_past.plt_cn
                  AND cmx.prev_plt_cn = c_past.plt_cn
                  AND cmx.prevcond = c_past.condid
                  AND cmx.condid = c.condid
                  AND ((cmx.subptyp = 3 and c.prop_basis = 'MACR') or
                    (cmx.subptyp = 1 and c.prop_basis = 'SUBP'))
                         -- join past conditions / change matrix table
                GROUP BY psm.cn, p.cn)
         GROUP BY pop_stratum_cn) phase_2_summary
    WHERE phase_1_summary.pop_stratum_cn =
        phase_2_summary.pop_stratum_cn(+)
    GROUP BY pop_eval_grp_cn,
          eval_grp,
          estn_unit_cn,
          phase_1_summary.total_area) estimate_by_estn_unit
GROUP BY pop_eval_grp_cn, eval_grp
```

Results of Example 4.16:

Area change estimates and sampling errors based on remeasured plots, Minnesota, 2007.

	**Forest to water**	**Water to forest**	**Large diameter forest at both measurements**
**Changes to where clause**	**AND (c.cond_status_cd IN (3,4) AND c_past.cond_status_cd = 1)**	**AND (c.cond_status_cd=1 AND c_past.cond_status_cd IN (3,4))**	**AND (c.cond_status_cd=1 AND c_past.cond_status_cd=1 AND C.STDSZCD = 1 AND c_past.STDSZCD = 1)**
EVAL_GRP	272007	272007	272007
ESTIMATE	27,345.1	77,978.2	3,631,160.4
SE_OF_ESTIMATE_PCT	29.2	18.4	2.9
VAR_OF_ESTIMATE	63,796,853	206,390,712	11,427,498,039
TOTAL_PLOTS	12,280	12,280	12,280
NON_ZERO_PLOTS	32	57	1,007
TOTAL_POPULATION_ ACRES	54,008,479	54,008,479	54,008,479

Literature Cited

Bechtold, W.A.; Patterson, P.L., editors. 2005. The enhanced Forest Inventory and Analysis program – national sampling design and estimation procedures. Gen. Tech. Rep. SRS-80. Asheville, NC: U.S. Department of Agriculture Forest Service, Southern Research Station. 85 p.

Cleland, D.T.; Freeouf, J.A.; Keys, J.E. [and others]. 2007. Ecological subregions: sections and subsections for the conterminous United States. GTR-WO-76. Washington, DC: U.S. Department of Agriculture, Forest Service.

Gillespie, A.J.R. 1999. Rationale for a national annual forest inventory program. Journal of Forestry. 97: 16-20.

Hansen, M.H.; Frieswyk, T.; Glover, J.F.; Kelly, J.F. 1992. The eastwide forest inventory data base: users manual. Gen. Tech. Rep. NC-151. St. Paul, MN: U.S. Department of Agriculture, Forest Service, North Central Forest Experiment Station. 48 p.

Hanson, E.J.; Azuma, D.L.; Hiserote, B.A. 2002. Site index equations and mean annual increment equations for Pacific Northwest Research Station Forest Inventory and Analysis inventories, 1985-2001. Res. Note. PNW-RN-533. Portland, OR: U.S. Department of Agriculture, Forest Service. 24 p.

Hawksworth, F.G. 1979. The 6-class dwarf mistletoe rating system. Gen. Tech. Rep. RM-48. Fort Collins, CO: U.S. Department of Agriculture, Forest Service, Rocky Mountain Forest and Range Experiment Station. 7 p.

Heath, L.S.; Hansen, M. H.; Smith, J.E. [and others]. 2009. Investigation into calculating tree biomass and carbon in the FIADB using a biomass expansion factor approach. In: Forest Inventory and Analysis (FIA) Symposium 2008. RMRS-P-56CD. Fort Collins, CO: U.S. Department of Agriculture, Forest Service, Rocky Mountain Research Station. 1 CD

Jenkins, J.C.; Chojnacky, D.C.; Heath, L.S.; Birdsey, R.A. 2003. National scale biomass estimators for United States tree species. Forest Science. 49: 12-35.

Lister, A.; Scott, C.T.; King, S.L. [and others]. 2005. Strategies for preserving owner privacy in the national information management system of the USDA Forest Service's Forest Inventory and Analysis unit. In: Proceedings of the 4th annual Forest Inventory and Analysis symposium. Gen. Tech. Rep. NC-GTR-252. St. Paul, MN: U.S. Department of Agriculture, Forest Service, North Central Research Station: 163-166.

MacLean, C.D. 1973. Estimating productivity on sites with a low stocking capacity. Res. Paper. PNW-RP-152. Portland, OR: U.S. Department of Agriculture, Forest Service, Pacific Northwest Forest and Range Experiment Station. 18 p.

MacLean C.D.; Bolsinger, C.L. 1974. Stockability equations for California forest land. Res. Note. PNW-RN-233. Portland, OR: U.S. Department of Agriculture, Forest Service. 10 p.

Miles, P.D; Smith, W.B. 2009. Specific gravity and other properties of wood and bark for 156 tree species found in North America. Res. Note. NRS-38. Newtown Square, PA: U.S. Department of Agriculture, Forest Service, Northern Research Station. 35 p.

Miles, P.D.; Brand, G.J.; Alerich, C.L. [and others]. 2001. The forest inventory and analysis database: database description and users manual version 1.0. Gen. Tech. Rep. NC-218. St. Paul, MN: U.S. Department of Agriculture, Forest Service, North Central Research Station. 130 p.

Miles, P.D.; Jacobson, K.; Brand, G.J. [and others]. 2007. Minnesota's forests 1999-2003 (Part A). Reourc. Bull. NRS-12A. Newtown Square, PA: U.S. Department of Agriculture, Forest Service, Northern Research Station. 92 p.

National Atlas of the United States. 2007. Congressional Districts of the United States - 110th Congress. Reston, VA: National Atlas of the United States. Available online: http://nationalatlas.gov/atlasftp.html

Nowacki, G.; Brock, T. 1995. Ecoregions and subregions of Alaska, EcoMap version 2.0 (map). Juneau, AK: U.S. Department of Agriculture, Forest Service, Alaska Region. scale 1:5,000,000. (http://agdcftp1.wr.usgs.gov/pub/projects/fhm/ecomap.gif)

Raile, G.K. 1982. Estimating stump volume. Res. Paper. NC-224. St. Paul, MN: U.S. Department of Agriculture, Forest Service, North Central Forest Experiment Station. 4 p.

Scott, C.T.; Cassell, D.L.; Hazard, J.W. 1993. Sampling design of the U.S. National Forest Health Program. In: Nyyssonen, A.; Poso, S.; Rautala, J. (eds.), Ilvessalo Symposium on National Forest Inventories. Research Parpers 444. [Location unknown]: Finnish Forest Research Institute London. p. 150-157.

Smith, J.E.; Heath, L.S. 2002. A model of forest floor carbon mass for United States forest types. Res. Paper. NE-722. Newtown Square, PA: U.S. Department of Agriculture, Forest Service, Northeastern Research Station. 37 p.

Smith, J.E.; Heath, L.S. 2008. Forest sections of the land use change and forestry chapter, and Annex. In: US Environmental Protection Agency, Inventory of US Greenhouse Gas Emissions and Sinks: 1990-2006. EPA 430-R-08-005. http://www.epa.gov/climatechange/emissions/usinventoryreport.htm (17 October).

Smith, W.B. 2002. Forest inventory and analysis: a national inventory and monitoring program. Environmental Pollution. 116 (Suppl. 1): S233-S242.

U.S. Census Bureau. 1994. The geographic areas reference manual. http://www.census.gov/geo/www/garm.html [Date accessed: June 18, 2008].

U.S. Department of Agriculture, Forest Service. 2008. Accuracy standards. In: Forest Survey Handbook. FSH 4809.11, chapter 10. Washington, DC: U.S. Department of Agriculture, Forest Service. [Not paged]

U.S. Environmental Protection Agency. 2008. Inventory of U.S. greenhouse gas emissions and sinks: 1990–2006. EPA 430-R-08-005. Washington, DC: United States Environmental Protection Agency, Office of Atmospheric Programs. Available online at http://epa.gov/climatechange/emissions/.

Van Hooser, D.D.; Cost, N.D.; Lund, H.G. 1993. The history of the forest survey program in the United States. In: Proceedings of the IUFRO Centennial Meeting. [Location unknown]: Japan Society of Forest Planning Press, Tokyo University of Agriculture: 19-27.

Wilson, H. M. 1900. A Dictionary of Topographic Forms. Journal of the American Geographical Society of New York. 32: 32-41.

Woodall, C.W.; Conkling, B.L.; Amacher, M.C. [and others]. 2010. The Forest Inventory and Analysis database version 4.0: database description and users manual for phase 3. Gen. Tech. Rep. NRS – 61. Newtown Square, PA: U.S. Department of Agriculture, Forest Service, Northern Research Station. 180 p.

Woudenberg, S.W.; Farrenkopf, T.O. 1995. The Westwide forest inventory data base: user's manual. Gen. Tech. Rep. INT-GTR-317. Ogden, UT: U.S. Department of Agriculture, Forest Service, Intermountain Research Station. 67 p.

Appendix A. Index of Column Names

The following table lists column names used in the database tables, their location within the table, and a short description.

Column name with (field guide section)	Table name	Location in table	Description
ACTUALHT (5.15)	TREE	22	Actual height of tree
ADFORCD	COND	15	Administrative forest code
ADJ_FACTOR_MACR	POP_STRATUM	12	Adjustment factor for the macroplot
ADJ_FACTOR_MICR	POP_STRATUM	14	Adjustment factor for the microplot
ADJ_FACTOR_SUBP	POP_STRATUM	13	Adjustment factor for the subplot
AGEDIA (7.2.5)	SITETREE	14	Age at diameter height
AGENTCD (5.21)	TREE	27	Cause of death (agent) code
ALLOWED_IN_FIELD	REF_FOREST_TYPE	6	Allowed in field
ALSTK	COND	53	All-live-tree stocking percent
ALSTKCD	COND	37	All live stocking code
ANN_INVENTORY	SURVEY	8	Annual inventory
AREA_SOURCE	POP_ESTN_UNIT	11	Source of area figures usually Census Bureau or from pixel counts
AREA_USED	POP_ESTN_UNIT	10	Area used to calculate all expansion factors
AREALAND_EU	POP_ESTN_UNIT	8	Land area within the estimation unit
AREATOT_EU	POP_ESTN_UNIT	9	Total area within the estimation unit
ASPECT	COND	34	Aspect
ASPECT (3.7)	SUBPLOT	17	Subplot aspect
ATTRIBUTE_DESCR	REF_POP_ATTRIBUTE	3	Estimation attribute e.g., Area of timberland
ATTRIBUTE_NBR	POP_EVAL_ATTRIBUTE	3	Attribute number
ATTRIBUTE_NBR	REF_POP_ATTRIBUTE	2	Arbitrary unique number
AUTHOR	REF_HABTYP_PUBLICATION	4	Author of publication
AZIMUTH (7.2.8)	SITETREE	19	Azimuth
AZIMUTH (5.4)	TREE	12	Azimuth
AZMCORN (4.2.6)	BOUNDARY	13	Corner azimuth
AZMLEFT (4.2.5)	BOUNDARY	12	Left azimuth
AZMRIGHT (4.2.8)	BOUNDARY	15	Right azimuth
BALIVE	COND	51	Basal area of live trees
BARK_SPGR_GREENVOL_DRYWT	REF_SPECIES	51	Green specific gravity bark (green volume and oven-dry weight)
BARK_SPGR_GREENVOL_DRYWT_CIT	REF_SPECIES	52	Green specific gravity bark citation
BARK_VOL_PCT	REF_SPECIES	59	Bark volume as a percent of wood volume
BARK_VOL_PCT_CIT	REF_SPECIES	60	Bark volume as a percent of wood volume citation
BFSND	TREE	73	Board-foot-cull soundness
BHAGE	TREE	66	Breast height age
BNDCHG (4.2.3)	BOUNDARY	10	Boundary change code
BOLEHT	TREE	76	Bole height
BORED_CD_PNWRS	TREE	125	Tree bored code, Pacific Northwest Research Station
CANOPY_CVR_SAMPLE_METHOD_CD	COND	97	Canopy cover sample method code
CARBON_AG	TREE	121	Carbon aboveground
CARBON_BG	TREE	122	Carbon belowground
CARBON_DOWN_DEAD	COND	67	Carbon in down dead
CARBON_LITTER	COND	68	Carbon in litter

Column name with (field guide section)	Table name	Location in table	Description
CARBON_SOIL_ORG	COND	69	Carbon in soil fine organic material
CARBON_STANDING_DEAD	COND	70	Carbon in standing dead trees
CARBON_UNDERSTORY_AG	COND	71	Carbon in the aboveground portions of seedlings, shrubs, and bushes
CARBON_UNDERSTORY_BG	COND	72	Carbon in the belowground portion of seedlings, shrubs ,and bushes
CCLCD (5.17)	TREE	25	Crown class code
CDENCD (12.9)	TREE	61	Crown density code
CDIEBKCD (12.10)	TREE	62	Crown dieback code
CFSND	TREE	74	Cubic-foot-cull soundness
CITATION	REF_CITATION	2	Citation
CITATION_NBR	REF_CITATION	1	Citation number
CLASS	REF_SPECIES_GROUP	4	Class
CLIGHTCD (12.6)	TREE	59	Crown light exposure code
CN	BOUNDARY	1	Sequence number
CN	COND	1	Sequence number
CN	COUNTY	5	Sequence number
CN	PLOT	1	Sequence number
CN	POP_ESTN_UNIT	1	Sequence number
CN	POP_EVAL	1	Sequence number
CN	POP_EVAL_ATTRIBUTE	1	Sequence number
CN	POP_EVAL_GRP	1	Sequence number
CN	POP_EVAL_TYP	11	Sequence number
CN	POP_PLOT_STRATUM_ ASSGN	1	Sequence number
CN	POP_STRATUM	1	Sequence number
CN	REF_HABTYP_DESCRIPTION	1	Sequence number
CN	REF_HABTYP_PUBLICATION	1	Sequence number
CN	REF_POP_ATTRIBUTE	1	Sequence number
CN	REF_POP_EVAL_TYP_DESCR	9	Sequence number
CN	SEEDLING	1	Sequence number
CN	SITETREE	1	Sequence number
CN	SUBPLOT	1	Sequence number
CN	SUBP_COND	1	Sequence number
CN	SUBP_COND_CHNG_MTRX	1	Sequence number
CN	SURVEY	1	Sequence number
CN	TREE	1	Sequence number
COMMON_NAME	REF_HABTYP_DESCRIPTION	5	Common name
COMMON_NAME	REF_SPECIES	2	Common name of species
CORE	REF_SPECIES	36	Core
COND_NONSAMPLE_REASN_CD (2.4.3)	COND	10	Condition nonsampled reason code
COND_STATUS_CD (2.4.2)	COND	9	Condition status code
CONDID (2.4.1)	COND	8	Condition class number
CONDID (6.3)	SEEDLING	9	Condition class number
CONDID	SITETREE	9	Condition class number
CONDID	SUBP_COND	9	Condition class number
CONDID	SUBP_COND_CHNG_MTRX	6	Condition class number
CONDID (5.3)	TREE	11	Condition class number
CONDLIST	SUBPLOT	15	Subplot/macroplot plot condition list
CONDLIST	SITETREE	24	Condition class list
CONDPROP_UNADJ	COND	29	Condition proportion unadjusted
CONGCD	PLOT	28	Congressional district code
CONTRAST (4.2.4)	BOUNDARY	11	Contrasting condition
COUNTYCD	BOUNDARY	6	County code
COUNTYCD	COND	6	County code
COUNTYCD	COUNTY	3	County code
COUNTYCD (1.2)	PLOT	8	County code

Column name with (field guide section)	Table name	Location in table	Description
COUNTYCD	POP_PLOT_STRATUM_ ASSGN	7	County code
COUNTYCD	SEEDLING	6	County code
COUNTYCD	SITETREE	7	County code
COUNTYCD	SUBPLOT	7	County code
COUNTYCD	SUBP_COND	6	County code
COUNTYCD	TREE	7	County code
COUNTYNM	COUNTY	4	County name
CPOSCD (12.7)	TREE	58	Crown position code
CR (5.19)	TREE	24	Compacted crown ratio
CREATED_BY	BOUNDARY	18	Created by
CREATED_BY	COND	73	Created by
CREATED_BY	COUNTY	6	Created by
CREATED_BY	PLOT	33	Created by
CREATED_BY	POP_ESTN_UNIT	14	Created by
CREATED_BY	POP_EVAL	9	Created by
CREATED_BY	POP_EVAL_ATTRIBUTE	5	Created by
CREATED_BY	POP_EVAL_GRP	13	Created by
CREATED_BY	POP_EVAL_TYP	5	Created by
CREATED_BY	POP_PLOT_STRATUM_ ASSGN	13	Created by
CREATED_BY	POP_STRATUM	15	Created by
CREATED_BY	REF_CITATION	3	Created by
CREATED_BY	REF_FIADB_VERSION	3	Created by
CREATED_BY	REF_FOREST_TYPE	7	Created by
CREATED_BY	REF_HABTYP_DESCRIPTION	7	Created by
CREATED_BY	REF_HABTYP_PUBLICATION	7	Created by
CREATED_BY	REF_POP_ATTRIBUTE	6	Created by
CREATED_BY	REF_POP_EVAL_TYP_DESCR	3	Created by
CREATED_BY	REF_SPECIES	30	Created by
CREATED_BY	REF_SPECIES_GROUP	5	Created by
CREATED_BY	REF_STATE_ELEV	6	Created by
CREATED_BY	REF_UNIT	4	Created by
CREATED_BY	SEEDLING	15	Created by
CREATED_BY	SITETREE	25	Created by
CREATED_BY	SUBPLOT	20	Created by
CREATED_BY	SUBP_COND	10	Created by
CREATED_BY	SUBP_COND_CHNG_MTRX	10	Created by
CREATED_BY	SURVEY	10	Created by
CREATED_BY	TREE	81	Created by
CREATED_BY	TREE_REGIONAL_BIOMASS	5	Created by
CREATED_DATE	BOUNDARY	19	Created date
CREATED_DATE	COND	74	Created date
CREATED_DATE	COUNTY	7	Created date
CREATED_DATE	PLOT	34	Created date
CREATED_DATE	POP_ESTN_UNIT	15	Created date
CREATED_DATE	POP_EVAL	10	Created date
CREATED_DATE	POP_EVAL_ATTRIBUTE	6	Created date
CREATED_DATE	POP_EVAL_GRP	14	Created date
CREATED_DATE	POP_EVAL_TYP	6	Created date
CREATED_DATE	POP_PLOT_STRATUM_ ASSGN	14	Created date
CREATED_DATE	POP_STRATUM	16	Created date
CREATED_DATE	REF_CITATION	4	Created date
CREATED_DATE	REF_FIADB_VERSION	4	Created date
CREATED_DATE	REF_FOREST_TYPE	8	Created date
CREATED_DATE	REF_HABTYP_DESCRIPTION	8	Created date
CREATED_DATE	REF_HABTYP_PUBLICATION	8	Created date
CREATED_DATE	REF_POP_ATTRIBUTE	7	Created date
CREATED_DATE	REF_POP_EVAL_TYP_DESCR	4	Created date
CREATED_DATE	REF_SPECIES	31	Created date
CREATED_DATE	REF_SPECIES_GROUP	6	Created date
CREATED_DATE	REF_STATE_ELEV	7	Created date
CREATED_DATE	REF_UNIT	5	Created date

Column name with (field guide section)	Table name	Location in table	Description
CREATED_DATE	SEEDLING	16	Created date
CREATED_DATE	SITETREE	26	Created date
CREATED_DATE	SUBPLOT	21	Created date
CREATED_DATE	SUBP_COND	11	Created date
CREATED_DATE	SUBP_COND_CHNG_MTRX	11	Created date
CREATED_DATE	SURVEY	11	Created date
CREATED_DATE	TREE	82	Created date
CREATED_DATE	TREE_REGIONAL_BIOMASS	6	Created date
CREATED_IN_INSTANCE	BOUNDARY	20	Created in instance
CREATED_IN_INSTANCE	COND	75	Created in instance
CREATED_IN_INSTANCE	COUNTY	8	Created in instance
CREATED_IN_INSTANCE	PLOT	35	Created in instance
CREATED_IN_INSTANCE	POP_ESTN_UNIT	16	Created in instance
CREATED_IN_INSTANCE	POP_EVAL	11	Created in instance
CREATED_IN_INSTANCE	POP_EVAL_ATTRIBUTE	7	Created in instance
CREATED_IN_INSTANCE	POP_EVAL_GRP	15	Created in instance
CREATED_IN_INSTANCE	POP_EVAL_TYP	7	Created in instance
CREATED_IN_INSTANCE	POP_PLOT_STRATUM_ ASSGN	15	Created in instance
CREATED_IN_INSTANCE	POP_STRATUM	17	Created in instance
CREATED_IN_INSTANCE	REF_CITATION	5	Created in instance
CREATED_IN_INSTANCE	REF_FIADB_VERSION	5	Created in instance
CREATED_IN_INSTANCE	REF_FOREST_TYPE	9	Created in instance
CREATED_IN_INSTANCE	REF_HABTYP_DESCRIPTION	9	Created in instance
CREATED_IN_INSTANCE	REF_HABTYP_PUBLICATION	9	Created in instance
CREATED_IN_INSTANCE	REF_POP_ATTRIBUTE	8	Created in instance
CREATED_IN_INSTANCE	REF_POP_EVAL_TYP_DESCR	5	Created in instance
CREATED_IN_INSTANCE	REF_SPECIES	32	Created in instance
CREATED_IN_INSTANCE	REF_SPECIES_GROUP	7	Created in instance
CREATED_IN_INSTANCE	REF_STATE_ELEV	8	Created in instance
CREATED_IN_INSTANCE	REF_UNIT	6	Created in instance
CREATED_IN_INSTANCE	SEEDLING	17	Created in instance
CREATED_IN_INSTANCE	SITETREE	27	Created in instance
CREATED_IN_INSTANCE	SUBPLOT	22	Created in instance
CREATED_IN_INSTANCE	SUBP_COND	12	Created in instance
CREATED_IN_INSTANCE	SUBP_COND_CHNG_MTRX	12	Created in instance
CREATED_IN_INSTANCE	SURVEY	12	Created in instance
CREATED_IN_INSTANCE	TREE	83	Created in instance
CREATED_IN_INSTANCE	TREE_REGIONAL_BIOMASS	7	Created in instance
CTY_CN	PLOT	3	County sequence number
CULL	TREE	28	Rotten and missing cull, computed and includes percent missing top
CULL_FLD (5.13)	TREE	91	Rotten and missing cull, field-recorded
CULLBF	TREE	71	Board-foot cull
CULLCF	TREE	72	Cubic-foot cull
CULLDEAD	TREE	68	Dead cull
CULLFORM	TREE	69	Form cull
CULLMSTOP	TREE	70	Missing top cull
CVIGORCD (12.8)	TREE	60	Sapling vigor code
CYCLE	BOUNDARY	16	Inventory cycle number
CYCLE	COND	79	Inventory cycle number
CYCLE	PLOT	46	Inventory cycle number
CYCLE	SEEDLING	23	Inventory cycle number
CYCLE	SITETREE	31	Inventory cycle number
CYCLE	SUBPLOT	26	Inventory cycle number
CYCLE	SUBP_COND	21	Inventory cycle number
CYCLE	SURVEY	16	Inventory cycle number
CYCLE	TREE	123	Inventory cycle number
DAMLOC1 (5.20.1)	TREE	29	Damage location 1 code

Column name with (field guide section)	Table name	Location in table	Description
DAMLOC1_PNWRS	TREE	126	Damage location 1, Pacific Northwest Research Station
DAMLOC2 (5.20.4)	TREE	32	Damage location 2 code
DAMLOC2_PNWRS	TREE	127	Damage location 2, Pacific Northwest Research Station
DAMSEV1 (5.20.3)	TREE	31	Damage severity 1 code
DAMSEV2 (5.20.6)	TREE	34	Damage severity 2 code
DAMTYP1 (5.20.2)	TREE	30	Damage type 1 code
DAMTYP2 (5.20.5)	TREE	33	Damage type 2 code
DECAYCD (5.23)	TREE	35	Decay class code
DECLINATION (1.11)	PLOT	40	Declination
DESCR	REF_FIADB_VERSION	2	Version description
DESIGNCD	PLOT	17	Plot design code
DIA (7.2.3)	SITETREE	12	Current diameter
DIA (5.9.2)	TREE	18	Current diameter
DIACALC	TREE	65	Current diameter calculated
DIACHECK (5.12)	TREE	54	Diameter check code
DIACHECK_PNWRS	TREE	128	Diameter check, Pacific Northwest Research Station
DIAHTCD	TREE	19	Diameter height code
DIST (7.2.9)	SITETREE	20	Horizontal distance
DIST (5.5)	TREE	13	Horizontal distance
DISTCORN (4.2.7)	BOUNDARY	14	Corner distance
DMG_AGENT1_CD_PNWRS	TREE	129	Damage agent 1, Pacific Northwest Research Station
DMG_AGENT2_CD_PNWRS	TREE	130	Damage agent 2, Pacific Northwest Research Station
DMG_AGENT3_CD_PNWRS	TREE	131	Damage agent 3, Pacific Northwest Research Station
DRYBIO_BG	TREE	120	Dry biomass belowground
DRYBIO_BOLE	TREE	115	Dry biomass of bole
DRYBIO_SAPLING	TREE	118	Dry biomass of sapling
DRYBIO_STUMP	TREE	117	Dry biomass of stump
DRYBIO_TOP	TREE	116	Dry biomass of top
DRYBIO_WDLD_SPP	TREE	119	Dry biomass of woodland species
DSTRBCD1 (2.5.11)	COND	38	Disturbance 1 code
DSTRBCD2 (2.5.13)	COND	40	Disturbance 2 code
DSTRBCD3 (2.5.15)	COND	42	Disturbance 3 code
DSTRBYR1 (2.5.12)	COND	39	Year of disturbance 1
DSTRBYR2 (2.5.14)	COND	41	Year of disturbance 2
DSTRBYR3 (2.5.16)	COND	43	Year of disturbance 3
E_SPGRPCD	REF_SPECIES	8	East species group code
EAST	REF_SPECIES	25	East
ECO_UNIT_PNW	PLOT	48	Ecological unit used to identify Pacific Northwest Research Station stockability algorithms
ECOSUBCD	PLOT	27	Ecological subsection code
ELEV	PLOT	22	Elevation
EMAP_HEX	PLOT	41	EMAP hexagon
END_INVYR	POP_EVAL	16	End inventory year
ESTN_UNIT	POP_ESTN_UNIT	5	Estimation unit
ESTN_UNIT	POP_PLOT_STRATUM_ ASSGN	11	Estimation unit
ESTN_UNIT	POP_STRATUM	5	Estimation unit
ESTN_UNIT_CN	POP_STRATUM	2	Estimation unit sequence number
ESTN_UNIT_DESCR	POP_ESTN_UNIT	6	Estimation unit description
EVAL_CN	POP_ESTN_UNIT	2	Evaluation sequence number
EVAL_CN	POP_EVAL_ATTRIBUTE	2	Evaluation sequence number
EVAL_CN	POP_EVAL_TYP	2	Evaluation sequence number
EVAL_CN_FOR_EXPALL	POP_EVAL_GRP	2	Evaluation sequence number for expansions of all plots

Column name with (field guide section)	Table name	Location in table	Description
EVAL_CN_FOR_EXPCURR	POP_EVAL_GRP	3	Evaluation sequence number for expansions of current area
EVAL_CN_FOR_EXPGROW	POP_EVAL_GRP	5	Evaluation sequence number for expansions of growth
EVAL_CN_FOR_EXPMORT	POP_EVAL_GRP	6	Evaluation sequence number for expansions of mortality
EVAL_CN_FOR_EXPREMV	POP_EVAL_GRP	7	Evaluation sequence number for expansions of removals
EVAL_CN_FOR_EXPVOL	POP_EVAL_GRP	4	Evaluation sequence number for expansions of volume
EVAL_DESCR	POP_EVAL	4	Evaluation description
EVAL_GRP	POP_EVAL_GRP	9	Reporting year followed by 4 more digits to make the statecd/eval_grp combo unique
EVAL_GRP_CN	POP_EVAL_TYP	1	Evaluation group sequence number
EVAL_GRP_DESCR	POP_EVAL_GRP	10	Evaluation group description
EVAL_TYP	POP_EVAL_TYP	3	Evaluation type
EVAL_TYP	REF_POP_EVAL_TYP_DESCR	1	Evaluation type
EVAL_TYP_DESCR	REF_POP_EVAL_TYP_DESCR	2	Evaluation type description
EVALID	POP_ESTN_UNIT	4	Evaluation identifier
EVALID	POP_EVAL	3	Evaluation identifier
EVALID	POP_PLOT_STRATUM_ ASSGN	10	Evaluation identifier
EVALID	POP_STRATUM	4	Evaluation identifier
EXISTS_IN_NCRS	REF_SPECIES	13	Exists in the North Central Research Station States
EXISTS_IN_NERS	REF_SPECIES	14	Exists in the Northeastern Research Station States
EXISTS_IN_PNWRS	REF_SPECIES	15	Exists in the Pacific Northwest Research Station States
EXISTS_IN_RMRS	REF_SPECIES	16	Exists in the Rocky Mountain Research Station States
EXISTS_IN_SRS	REF_SPECIES	17	Exists in the Southern Research Station States
EXPNS	POP_STRATUM	11	Expansion factor
EXPRESSION	REF_POP_ATTRIBUTE	4	Part of the expression used to produce the estimate
FGROWBFSL	TREE	95	Net annual merchantable board-foot growth of sawtimber tree on forest land
FGROWCFAL	TREE	96	Net annual sound cubic-foot growth of a live tree on forest land
FGROWCFGS	TREE	94	Net annual merchantable cubic-foot growth of growing-stock tree on forest land
FIRE_SRS	COND	87	Fire, Southern Research Station
FLDAGE	COND	52	Field-recorded stand age
FLDSZCD (2.5.4)	COND	21	Field stand-size class code
FLDTYPCD (2.5.3)	COND	17	Field forest type code
FMORTBFSL	TREE	98	Board-foot volume of a sawtimber tree for mortality purposes on forest land
FMORTCFAL	TREE	99	Sound cubic-foot volume of a tree for mortality purposes on forest land
FMORTCFGS	TREE	97	Cubic-foot volume of a growing-stock tree for mortality purposes on forest land.
FOOTNOTE	REF_POP_ATTRIBUTE	12	Footnote
FOREST_TYPE_SPGRPCD	REF_SPECIES	12	Forest type species group code

Column name with (field guide section)	Table name	Location in table	Description
FORINDCD (2.5.8)	COND	14	Private owner industrial status code
FORMCL	TREE	77	Form class
FORTYPCD	COND	16	Forest type code
FORTYPCDCALC	COND	55	Forest type code calculated with a national algorithm
FREMVBFSL	TREE	101	Board-foot volume of a sawtimber tree for removal purposes on forest land
FREMVCFAL	TREE	102	Sound cubic-foot volume of the tree for removal purposes on forest land
FREMVCFGS	TREE	100	Cubic-foot volume of a growing-stock tree for removal purposes on forest land
GENUS	REF_SPECIES	3	Genus
GRAZING_SRS	COND	88	Grazing, Southern Research Station
GROUND_LAND_CLASS_PNW	COND	82	Present ground class code, Pacific Northwest Research Station
GROW_TYP_CD	PLOT	23	Type of annual volume growth code
GROWBFSL	TREE	46	Net annual merchantable board-foot growth of sawtimber size tree on timberland
GROWCFAL	TREE	47	Net annual sound cubic-foot growth of a live tree on timberland
GROWCFGS	TREE	45	Net annual merchantable cubic-foot growth of growing-stock tree on timberland
GSSTK	COND	54	Growing-stock stocking percent
GSSTKCD	COND	36	Growing-stock stocking code
HAPTYPCD	REF_HABTYP_DESCRIPTION	2	Habitat type code
HABTYPCD1	COND	56	Primary condition habitat type
HABTYPCD1_DESCR_PUB_CD	COND	58	Habitat type code 1 description publication code
HABTYPCD1_PUB_CD	COND	57	Habitat type code 1 publication code
HABTYPCD2	COND	59	Secondary condition habitat type
HABTYPCD2_DESCR_PUB_CD	COND	61	Habitat type code 2 description publication code
HABTYPCD2_PUB_CD	COND	60	Habitat type code 2 publication code
HARVEST_TYPE1_SRS	COND	89	Harvest type code 1, Southern Research Station
HARVEST_TYPE2_SRS	COND	90	Harvest type code 2, Southern Research Station
HARVEST_TYPE3_SRS	COND	91	Harvest type code 3, Southern Research Station
HIGHEST_POINT	REF_STATE_ELEV	5	Highest point
HRDWD_CLUMP_CD	TREE	79	Hardwood clump code
HT (7.24)	SITETREE	13	Total height
HT (5.14)	TREE	20	Total height
HTCALC	TREE	78	Current height calculated
HTCD (5.16)	TREE	21	Height method code
HTDMP (5.24)	TREE	88	Length (height) to diameter measurement point
INSTALL_TYPE	REF_FIADB_VERSION	12	Install type
INTENSITY	PLOT	45	Intensity

Column name with (field guide section)	Table name	Location in table	Description
INVASIVE_NONSAMPLE_REASON_CD	SUBPLOT	34	Invasive nonsampled reason code
INVASIVE_SAMPLING_STATUS_CD	PLOT	55	Invasive sampling status code
INVASIVE_SPECIMEN_RULE_CD	PLOT	56	Invasive specimen rule code
INVASIVE_SUBP_STATUS_CD	SUBPLOT	33	Invasive subplot status code
INVYR	BOUNDARY	3	Inventory year
INVYR	COND	3	Inventory year
INVYR	PLOT	5	Inventory year
INVYR	POP_PLOT_STRATUM_ ASSGN	5	Inventory year
INVYR	SEEDLING	3	Inventory year
INVYR	SITETREE	4	Inventory year
INVYR	SUBPLOT	4	Inventory year
INVYR	SUBP_COND	3	Inventory year
INVYR	SURVEY	2	Inventory year
INVYR	TREE	4	Inventory year
JENKINS_FOLIAGE_RATIO_B1	REF_SPECIES	44	Jenkins foliage ratio B1
JENKINS_FOLIAGE_RATIO_B2	REF_SPECIES	45	Jenkins foliage ratio B2
JENKINS_ROOT_RATIO_B1	REF_SPECIES	46	Jenkins root ratio B1
JENKINS_ROOT_RATIO_B2	REF_SPECIES	47	Jenkins root ratio B2
JENKINS_SAPLING_ADJUSTMENT	REF_SPECIES	49	Jenkins sapling adjustment factor
JENKINS_SPGRPCD	REF_SPECIES	37	Jenkins species group code
JENKINS_STEM_BARK_RATIO_B1	REF_SPECIES	42	Jenkins stem bark ratio B1
JENKINS_STEM_BARK_RATIO_B2	REF_SPECIES	43	Jenkins stem bark ratio B2
JENKINS_STEM_WOOD_RATIO_B1	REF_SPECIES	40	Jenkins stem wood ratio B1
JENKINS_STEM_WOOD_RATIO_B2	REF_SPECIES	41	Jenkins stem wood ratio B2
JENKINS_TOTAL_B1	REF_SPECIES	38	Jenkins coefficient B1
JENKINS_TOTAL_B2	REF_SPECIES	39	Jenkins coefficient B2
KINDCD (1.7)	PLOT	16	Sample kind code
KINDCD_NC	PLOT	31	Sample kind code, North Central
LAND_ONLY	POP_EVAL_GRP	12	Land only
LAND_USE_SRS	COND	92	Land use, Southern Research Station
LAT (1.6.7)	PLOT	20	Latitude
LIVE_CANOPY_CVR_PCT	COND	98	Live canopy cover percent
LIVE_MISSING_CANOPY_CVR_PCT	COND	99	Live plus missing canopy cover percent
LOCATION_NM	POP_EVAL	6	Usually State name or super State
LON (1.16.8)	PLOT	21	Longitude
LOWEST_POINT	REF_STATE_ELEV	4	Lowest point
MACRCOND	SUBPLOT	14	Macroplot center condition
MACRCOND_PROP	SUBP_COND	18	Proportion of this macroplot in this condition
MACRO_BREAKPOINT_DIA (1.17)	PLOT	44	Macroplot breakpoint diameter
MACRPROP_UNADJ	COND	32	Macroplot proportion unadjusted
MAJOR_SPGRPCD	REF_SPECIES	10	Major species group code
MANUAL (1.9)	PLOT	29	Manual (field guide) version number
MANUAL_END	REF_FOREST_TYPE	5	Manual end
MANUAL_END	REF_SPECIES	29	Manual end
MANUAL_START	REF_FOREST_TYPE	4	Manual start
MANUAL_START	REF_SPECIES	28	Manual start
MAPDEN	COND	18	Mapping density
MAX_ELEV	REF_STATE_ELEV	3	Maximum elevation
MC_PCT_GREEN BARK	REF_SPECIES	55	Moisture content of green bark as a percent of oven-dry weight
MC_PCT_GREEN_BARK_CIT	REF_SPECIES	56	Moisture content of green bark citation
MC_PCT_GREEN_WOOD	REF_SPECIES	53	Moisture content of green wood as a percent of oven-dry weight

Column name with (field guide section)	Table name	Location in table	Description
MC_PCT_GREEN_WOOD_CIT	REF_SPECIES	54	Moisture content of green wood citation
MEANING	REF_FOREST_TYPE	2	Meaning
MEANING	REF_UNIT	3	Meaning
MEASDAY (1.10.3)	PLOT	14	Measurement day
MEASMON (1.10.2)	PLOT	13	Measurement month
MEASYEAR (1.10.1)	PLOT	12	Measurement year
METHOD	SITETREE	21	Site tree method code
MICRCOND (3.5)	SUBPLOT	12	Microplot center condition
MICRCOND_PROP	SUBP_COND	16	Proportion of this microplot in this condition
MICROPLOT_LOC	PLOT	39	Microplot location
MICRPROP_UNADJ	COND	30	Microplot proportion unadjusted
MIN_ELEV	REF_STATE_ELEV	2	Minimum elevation
MIST_CL_CD (5.26)	TREE	90	Mistletoe class code
MIST_CL_CD_PNWRS	TREE	132	Leafy mistletoe class code, Pacific Northwest Research Station
MIXEDCONFCD	COND	62	Calculated forest type for mixed conifer site
MODIFIED_BY	BOUNDARY	21	Modified by
MODIFIED_BY	COND	76	Modified by
MODIFIED_BY	COUNTY	9	Modified by
MODIFIED_BY	PLOT	36	Modified by
MODIFIED_BY	POP_ESTN_UNIT	17	Modified by
MODIFIED_BY	POP_EVAL	12	Modified by
MODIFIED_BY	POP_EVAL_ATTRIBUTE	8	Modified by
MODIFIED_BY	POP_EVAL_GRP	16	Modified by
MODIFIED_BY	POP_EVAL_TYP	8	Modified by
MODIFIED_BY	POP_PLOT_STRATUM_ASSGN	16	Modified by
MODIFIED_BY	POP_STRATUM	18	Modified by
MODIFIED_BY	REF_CITATION	6	Modified by
MODIFIED_BY	REF_FIADB_VERSION	6	Modified by
MODIFIED_BY	REF_FOREST_TYPE	10	Modified by
MODIFIED_BY	REF_HABTYP_DESCRIPTION	10	Modified by
MODIFIED_BY	REF_HABTYP_PUBLICATION	10	Modified by
MODIFIED_BY	REF_POP_ATTRIBUTE	9	Modified by
MODIFIED_BY	REF_POP_EVAL_TYP_DESCR	6	Modified by
MODIFIED_BY	REF_SPECIES	33	Modified by
MODIFIED_BY	REF_SPECIES_GROUP	8	Modified by
MODIFIED_BY	REF_STATE_ELEV	9	Modified by
MODIFIED_BY	REF_UNIT	7	Modified by
MODIFIED_BY	SEEDLING	18	Modified by
MODIFIED_BY	SITETREE	28	Modified by
MODIFIED_BY	SUBPLOT	23	Modified by
MODIFIED_BY	SUBP_COND	13	Modified by
MODIFIED_BY	SUBP_COND_CHNG_MTRX	13	Modified by
MODIFIED_BY	SURVEY	13	Modified by
MODIFIED_BY	TREE	84	Modified by
MODIFIED_BY	TREE_REGIONAL_BIOMASS	8	Modified by
MODIFIED_DATE	BOUNDARY	22	Modified date
MODIFIED_DATE	COND	77	Modified date
MODIFIED_DATE	COUNTY	10	Modified date
MODIFIED_DATE	PLOT	37	Modified date
MODIFIED_DATE	POP_ESTN_UNIT	18	Modified date
MODIFIED_DATE	POP_EVAL	13	Modified date
MODIFIED_DATE	POP_EVAL_ATTRIBUTE	9	Modified date
MODIFIED_DATE	POP_EVAL_GRP	17	Modified date
MODIFIED_DATE	POP_EVAL_TYP	9	Modified date
MODIFIED_DATE	POP_PLOT_STRATUM_ASSGN	17	Modified date
MODIFIED_DATE	POP_STRATUM	19	Modified date

Column name with (field guide section)	Table name	Location in table	Description
MODIFIED_DATE	REF_CITATION	7	Modified date
MODIFIED_DATE	REF_FIADB_VERSION	7	Modified date
MODIFIED_DATE	REF_FOREST_TYPE	11	Modified date
MODIFIED_DATE	REF_HABTYP_DESCRIPTION	11	Modified date
MODIFIED_DATE	REF_HABTYP_PUBLICATION	11	Modified date
MODIFIED_DATE	REF_POP_ATTRIBUTE	10	Modified date
MODIFIED_DATE	REF_POP_EVAL_TYP_DESCR	7	Modified date
MODIFIED_DATE	REF_SPECIES	34	Modified date
MODIFIED_DATE	REF_SPECIES_GROUP	9	Modified date
MODIFIED_DATE	REF_STATE_ELEV	10	Modified date
MODIFIED_DATE	REF_UNIT	8	Modified date
MODIFIED_DATE	SEEDLING	19	Modified date
MODIFIED_DATE	SITETREE	29	Modified date
MODIFIED_DATE	SUBPLOT	24	Modified date
MODIFIED_DATE	SUBP_COND	14	Modified date
MODIFIED_DATE	SUBP_COND_CHNG_MTRX	14	Modified date
MODIFIED_DATE	SURVEY	14	Modified date
MODIFIED_DATE	TREE	85	Modified date
MODIFIED_DATE	TREE_REGIONAL_BIOMASS	9	Modified date
MODIFIED_IN_INSTANCE	BOUNDARY	78	Modified in instance
MODIFIED_IN_INSTANCE	COND	78	Modified in instance
MODIFIED_IN_INSTANCE	COUNTY	11	Modified in instance
MODIFIED_IN_INSTANCE	PLOT	38	Modified in instance
MODIFIED_IN_INSTANCE	POP_ESTN_UNIT	19	Modified in instance
MODIFIED_IN_INSTANCE	POP_EVAL	14	Modified in instance
MODIFIED_IN_INSTANCE	POP_EVAL_ATTRIBUTE	10	Modified in instance
MODIFIED_IN_INSTANCE	POP_EVAL_GRP	18	Modified in instance
MODIFIED_IN_INSTANCE	POP_EVAL_TYP	10	Modified in instance
MODIFIED_IN_INSTANCE	POP_PLOT_STRATUM_ASSGN	18	Modified in instance
MODIFIED_IN_INSTANCE	POP_STRATUM	20	Modified in instance
MODIFIED_IN_INSTANCE	REF_CITATION	8	Modified in instance
MODIFIED_IN_INSTANCE	REF_FIADB_VERSION	8	Modified in instance
MODIFIED_IN_INSTANCE	REF_FOREST_TYPE	12	Modified in instance
MODIFIED_IN_INSTANCE	REF_HABTYP_DESCRIPTION	12	Modified in instance
MODIFIED_IN_INSTANCE	REF_HABTYP_PUBLICATION	12	Modified in instance
MODIFIED_IN_INSTANCE	REF_POP_ATTRIBUTE	11	Modified in instance
MODIFIED_IN_INSTANCE	REF_POP_EVAL_TYP_DESCR	8	Modified in instance
MODIFIED_IN_INSTANCE	REF_SPECIES	35	Modified in instance
MODIFIED_IN_INSTANCE	REF_SPECIES_GROUP	10	Modified in instance
MODIFIED_IN_INSTANCE	REF_STATE_ELEV	11	Modified in instance
MODIFIED_IN_INSTANCE	REF_UNIT	9	Modified in instance
MODIFIED_IN_INSTANCE	SEEDLING	20	Modified in instance
MODIFIED_IN_INSTANCE	SITETREE	30	Modified in instance
MODIFIED_IN_INSTANCE	SUBPLOT	25	Modified in instance
MODIFIED_IN_INSTANCE	SUBP_COND	15	Modified in instance
MODIFIED_IN_INSTANCE	SUBP_COND_CHNG_MTRX	15	Modified in instance
MODIFIED_IN_INSTANCE	SURVEY	15	Modified in instance
MODIFIED_IN_INSTANCE	TREE	86	Modified in instance
MODIFIED_IN_INSTANCE	TREE_REGIONAL_BIOMASS	10	Modified in instance
MORT_TYP_CD	PLOT	24	Type of annual mortality volume code
MORTBFSL	TREE	49	Board-foot volume of a sawtimber size tree on timberland for mortality purposes
MORTCD (5.7.3)	TREE	87	Mortality code
MORTCFAL	TREE	50	Sound cubic-foot volume of a tree on timberland for mortality purposes
MORTCFGS	TREE	48	Cubic-foot volume of a growing-stock tree on timberland for mortality purposes

Column name with (field guide section)	Table name	Location in table	Description
MORTYR (5.22)	TREE	55	Mortality year
NAME	REF_SPECIES_GROUP	2	Name
NBR_LIVE_STEMS	COND	100	Number of live stems
NF_COND_NONSAMPLE_REASN_CD	COND	96	Nonforest condition nonsampled reason code
NF_COND_STATUS_CD	COND	95	Nonforest condition status code
NF_PLOT_NONSAMPLE_REASN_CD	PLOT	52	Nonforest plot nonsampled reason code
NF_PLOT_STATUS_CD	PLOT	51	Nonforest plot status code
NF_SAMPLING_STATUS_CD	PLOT	50	Nonforest sampling status code
NF_SUBP_NONSAMPLE_REASN_CD	SUBPLOT	30	Nonforest subplot nonsampled reason code
NF_SUBP_STATUS_CD	SUBPLOT	29	Nonforest subplot status code
NONFR_INCL_PCT_MACRO	SUBP_COND	20	Nonforest inclusions percentage of macroplot
NONFR_INCL_PCT_SUBP	SUBP_COND	19	Nonforest inclusions percentage of subplot
NOTES	POP_EVAL	8	Evaluation notes
NOTES	POP_EVAL_GRP	19	Notes
NOTES	SURVEY	9	Notes (about the inventory)
OPERABILITY_SRS	COND	93	Operability in Southern Research Station
OWNCD (2.5.7)	COND	12	Owner class code
OWNGRPCD (2.5.2)	COND	13	Owner group code
P1PNTCNT_EU	POP_ESTN_UNIT	12	Phase 1 point count (total number of pixels) in the estimation unit
P1POINTCNT	POP_STRATUM	9	Phase 1 point count
P1SOURCE	POP_ESTN_UNIT	13	Phase 1 source
P2A_GRM_FLG	SUBPLOT	19	Periodic to annual growth, removal, and mortality flag
P2A_GRM_FLG	TREE	103	Periodic to annual growth, removal, and mortality flag
P2PANEL	PLOT	25	Phase 2 panel number
P2POINTCNT	POP_STRATUM	10	Phase 2 point count
P2VEG_SAMPLING_LEVEL_DETAIL_CD	PLOT	54	P2 vegetation sampling level detail code
P2VEG_SAMPLING_STATUS_CD	PLOT	53	P2 vegetation sampling status code
P2VEG_SUBP_NONSAMPLE_REASN_CD	SUBPLOT	32	P2 vegetation nonsampled reason code
P2VEG_SUBP_STATUS_CD	SUBPLOT	31	P2 vegetation subplot status code
P3_OZONE_IND	SURVEY	3	Phase 3 ozone indicator plot.
P3PANEL	PLOT	26	Phase 3 panel number
PHYSCLCD (2.5.23)	COND	35	Physiographic class code
PLANT_STOCKABILITY_FACTOR_PNW	COND	83	Plant stockability factor, Pacific Northwest Research Station
PLOT	BOUNDARY	7	Phase 2 Plot number
PLOT	COND	7	Phase 2 Plot number
PLOT (1.3)	PLOT	9	Phase 2 Plot number
PLOT	POP_PLOT_STRATUM_ASSGN	8	Phase 2 Plot number
PLOT	SEEDLING	7	Phase 2 Plot number
PLOT	SITETREE	8	Phase 2 Plot number
PLOT	SUBPLOT	8	Phase 2 Plot number
PLOT	SUBP_COND	7	Phase 2 Plot number
PLOT	TREE	8	Phase 2 Plot number
PLOT_NONSAMPLE_REASN_CD (1.5)	PLOT	11	Plot nonsampled reason code
PLOT_STATUS_CD (1.4)	PLOT	10	Plot status code
PLT_CN	BOUNDARY	2	Plot sequence number
PLT_CN	COND	2	Plot sequence number
PLT_CN	POP_PLOT_STRATUM_ASSGN	3	Plot sequence number

Column name with (field guide section)	Table name	Location in table	Description
PLT_CN	SEEDLING	2	Plot sequence number
PLT_CN	SITETREE	2	Plot sequence number
PLT_CN	SUBPLOT	2	Plot sequence number
PLT_CN	SUBP_COND	2	Plot sequence number
PLT_CN	SUBP_COND_CHNG_MTRX	5	Plot sequence number
PLT_CN	TREE	2	Plot sequence number
POINT_NONSAMPLE_REASN_CD (3.3)	SUBPLOT	11	Point nonsampled reason code
PRESNFCD	COND	50	Present nonforest code
PREV_PLT_CN	PLOT	4	Previous plot sequence number
PREV_PLT_CN	SUBP_COND_CHNG_MTRX	7	Previous plot sequence number
PREV_PNTN_SRS	TREE	142	Previous periodic prism point, tree number, Southern Research Station
PREV_SBP_CN	SUBPLOT	3	Previous subplot sequence number
PREV_SIT_CN	SITETREE	3	Previous site tree sequence number
PREV_STATUS_CD (5.6)	TREE	109	Previous tree status code
PREV_TRE_CN	TREE	3	Previous tree sequence number
PREV_WDLDSTEM (5.10)	TREE	110	Previous woodland tree species stem count
PREVCOND	SUBP_COND_CHNG_MTRX	8	Previous condition class number
PREVCOND	TREE	14	Previous condition class number
PREVDIA (5.9.1)	TREE	93	Previous diameter
PROP_BASIS	COND	28	Proportion basis
PUB_CD	REF_HABTYP_DESCRIPTION	3	Publication code
PUB_CD	REF_HABTYP_PUBLICATION	2	Publication code
QA_STATUS (1.14)	PLOT	32	Quality assurance status
RAILE_STUMP_DIB_B1	REF_SPECIES	62	Raile stump diameter inside bark equation coefficient B1
RAILE_STUMP_DIB_B2	REF_SPECIES	63	Raile stump diameter inside bark equation coefficient B2
RAILE_STUMP_DOB_B1	REF_SPECIES	61	Raile stump diameter outside bark equation coefficient B1
RDDISTCD (1.12)	PLOT	18	Horizontal distance to improved road code
RECONCILECD (5.7.1)	TREE	92	Reconcile code
REGION	REF_SPECIES_GROUP	3	Region
REGIONAL_DRYBIOM	TREE_REGIONAL_BIOMASS	4	Regional merchantable stem biomass oven-dry weight
REGIONAL_DRYBIOT	TREE_REGIONAL_BIOMASS	3	Regional total live tree biomass oven-dry weight
REMPER	PLOT	15	Remeasurement period
REMVBFSL	TREE	52	Board-foot volume of a sawtimber size tree on timberland for removal purposes
REMVCFAL	TREE	53	Sound cubic-foot volume of a tree on timberland for removal purposes
REMVCFGS	TREE	51	Cubic-foot volume of a growing-stock tree on timberland for removal purposes
REPORT_YEAR_NM	POP_EVAL	7	Report year name
RESERVCD (2.5.1)	COND	11	Reserved status code
ROOT_DIS_SEV_CD_PNWRS	SUBPLOT	28	Root disease severity rating code, Pacific Northwest Research Station
ROUGHCULL (5.25)	TREE	89	Rough cull percentage
RSCD	POP_ESTN_UNIT	3	Region or Station code

Column name with (field guide section)	Table name	Location in table	Description
RSCD	POP_EVAL	2	Region or Station code
RSCD	POP_EVAL_GRP	8	Region or Station code
RSCD	POP_PLOT_STRATUM_ ASSGN	9	Region or Station code
RSCD	POP_STRATUM	3	Region or Station code
RSCD	SURVEY	7	Region or Station code
SALVCD	TREE	56	Salvable dead code
SAMP_METHOD_CD	PLOT	42	Sample method code
SAWHT	TREE	75	Sawlog height
SCIENTIFIC_NAME	REF_HABTYP_DESCRIPTION	4	Scientific name
SEVERITY1_CD_PNWRS	TREE	133	Damage severity 1, Pacific Northwest Research Station, for years 2001-2004
SEVERITY1A_CD_PNWRS	TREE	134	Damage Severity 1, Pacific Northwest Research Station
SEVERITY1B_CD_PNWRS	TREE	135	Damage severity B, Pacific Northwest Research Station
SEVERITY2_CD_PNWRS	TREE	136	Damage severity 2, Pacific Northwest Research Station, for years 2001-2004
SEVERITY2A_CD_PNWRS	TREE	137	Damage severity 2A, Pacific Northwest Research Station, starting in 2005
SEVERITY2B_CD_PNWRS	TREE	138	Damage severity in 2B, Pacific Northwest Research Station, starting in 2005
SEVERITY3_CD_PNWRS	TREE	139	Damage severity 3, Pacific Northwest Research Station, for years 2001-2004
SFTWD_HRDWD	REF_SPECIES	19	Softwood or hardwood
SIBASE	COND	24	Site index base age
SIBASE	SITETREE	17	Site index base age
SICOND	COND	23	Site index for the condition
SISP	COND	25	Site index species code
SITECL_METHOD	COND	66	Site class method
SITECLCD	COND	22	Site productivity class code
SITECLCDEST	COND	64	Site productivity class code estimated
SITETREE	REF_SPECIES	18	Site tree
SITETREE_TREE	COND	65	Site tree tree number
SITREE	SITETREE	16	Site index for the tree
SITREE	TREE	80	Calculated site index
SITREE_EST	SITETREE	22	Estimated site index for the tree
SLOPE	COND	33	Slope
SLOPE (3.6)	SUBPLOT	16	Subplot slope
SOIL_ROOTING_DEPTH_PNW	COND	81	Soil rooting depth code, Pacific Northwest Research Station
SPCD	REF_SPECIES	1	Species code
SPCD (6.2)	SEEDLING	10	Species code
SPCD (7.2.2)	SITETREE	11	Species code
SPCD (5.8)	TREE	16	Species code
SPECIES	REF_SPECIES	4	Species name
SPECIES_SYMBOL	REF_SPECIES	7	Species symbol
SPGRPCD	REF_SPECIES_GROUP	1	Species group code
SPGRPCD	SEEDLING	11	Species group code
SPGRPCD	SITETREE	15	Species group code
SPGRPCD	TREE	17	Species group code
SRV_CN	PLOT	2	Survey sequence number
ST_EXISTS_IN_NCRS	REF_SPECIES	20	Site tree exists in the North Central Research Station region

Column name with (field guide section)	Table name	Location in table	Description
ST_EXISTS_IN_NERS	REF_SPECIES	21	Site tree exists in the Northeastern Research Station region
ST_EXISTS_IN_PNWRS	REF_SPECIES	22	Site tree exists in the Pacific Northwest Research Station region
ST_EXISTS_IN_RMRS	REF_SPECIES	23	Site tree exists in the Rocky Mountain Research Station region
ST_EXISTS_IN_SRS	REF_SPECIES	24	Site tree exists in the Southern Research Station region
STAND_STRUCTURE_SRS	COND	94	Stand structure, Southern Research Station
STANDING_DEAD_CD (5.7.2)	TREE	108	Standing dead code
START_INVYR	POP_EVAL	15	Start inventory year
STATEAB	SURVEY	5	State abbreviation
STATECD	BOUNDARY	4	State code
STATECD	COND	4	State code
STATECD	COUNTY	1	State code
STATECD (1.1)	PLOT	6	State code
STATECD	POP_ESTN_UNIT	7	State code
STATECD	POP_EVAL	5	State code
STATECD	POP_EVAL_ATTRIBUTE	4	State code
STATECD	POP_EVAL_GRP	11	State code
STATECD	POP_EVAL_TYP	4	State code
STATECD	POP_PLOT_STRATUM_ASSGN	4	State code
STATECD	POP_STRATUM	8	State code
STATECD	REF_STATE_ELEV	1	State code
STATECD	REF_UNIT	1	State code
STATECD	SEEDLING	4	State code
STATECD	SITETREE	5	State code
STATECD	SUBPLOT	5	State code
STATECD	SUBP_COND	4	State code
STATECD	SUBP_COND_CHNG_MTRX	2	State code
STATECD	SURVEY	4	State code
STATECD	TREE	5	State code
STATECD	TREE_REGIONAL_BIOMASS	2	State code
STATENM	SURVEY	6	State name
STATUSCD	TREE	15	Status code
STDAGE (2.5.10)	COND	19	Stand age
STDORGCD	COND	26	Stand origin code
STDORGSP	COND	27	Stand origin species code
STDSZCD	COND	20	Stand-size class code derived by algorithm
STND_COND_CD_PNWRS	COND	84	Stand condition code, Pacific Northwest Research Station
STND_STRUC_CD_PNWRS	COND	85	Stand structure code, Pacific Northwest Research Station
STOCKING	SEEDLING	12	Tree stocking
STOCKING	TREE	36	Tree stocking
STOCKING_SPGRPCD	REF_SPECIES	11	Stocking species group code
STRATUM_CN	POP_PLOT_STRATUM_ASSGN	2	Stratum sequence number
STRATUM_DESCR	POP_STRATUM	7	Stratum description
STRATUMCD	POP_PLOT_STRATUM_ASSGN	12	Stratum code
STRATUMCD	POP_STRATUM	6	Stratum code
STUMP_CD_PNWRS	COND	86	Stump code, Pacific Northwest Research Station
SUBCYCLE	BOUNDARY	17	Inventory subcycle number
SUBCYCLE	COND	80	Inventory subcycle number
SUBCYCLE	PLOT	47	Inventory subcycle number
SUBCYCLE	SEEDLING	24	Inventory subcycle number
SUBCYCLE	SITETREE	32	Inventory subcycle number
SUBCYCLE	SUBPLOT	27	Inventory subcycle number

Column name with (field guide section)	Table name	Location in table	Description
SUBCYCLE	SUBP_COND	22	Inventory subcycle number
SUBCYCLE	SURVEY	17	Inventory subcycle number
SUBCYCLE	TREE	124	Inventory subcycle number
SUBP (4.2.1)	BOUNDARY	8	Subplot number
SUBP (6.1)	SEEDLING	8	Subplot number
SUBP (7.2.7)	SITETREE	18	Subplot number
SUBP (3.1)	SUBPLOT	9	Subplot number
SUBP	SUBP_COND	8	Subplot number
SUBP	SUBP_COND_CHNG_MTRX	3	Subplot number
SUBP (5.1)	TREE	9	Subplot number
SUBP_EXAMINE_CD (1.6)	PLOT	43	Subplots examined code
SUBP_STATUS_CD (3.2)	SUBPLOT	10	Subplot status code
SUBPANEL	PLOT	30	Subpanel
SUBPCOND (3.4)	SUBPLOT	13	Subplot center condition
SUBPCOND_PROP	SUBP_COND	17	Proportion of this subplot in this condition
SUBPPROP_UNADJ	COND	31	Subplot proportion unadjusted
SUBPTYP (4.2.2)	BOUNDARY	9	Subplot type code
SUBPTYP	SUBP_COND_CHNG_MTRX	4	Subplot type code
SUBPTYP_PROP_CHNG	SUBP_COND_CHNG_MTRX	9	Percent change of subplot condition between previous to current inventory
SUBSPECIES	REF_SPECIES	6	Subspecies name
TITLE	REF_HABTYP_PUBLICATION	3	Title of publication
TOPO_POSITION_PNW	PLOT	49	Topographic position, Pacific Northwest Research Station
TOTAGE	SEEDLING	14	Total age of seedling
TOTAGE	TREE	67	Total tree age
TPA_UNADJ	SEEDLING	22	Trees per acre unadjusted
TPA_UNADJ	TREE	111	Trees per acre unadjusted
TPAGROW_UNADJ	TREE	114	Growth trees per acre unadjusted for denied access, hazardous, out of sample conditions
TPAMORT_UNADJ	TREE	112	Mortality trees per acre per year unadjusted for denied access, hazardous, out of sample conditions
TPAREMV_UNADJ	TREE	113	Removal trees per acre per year unadjusted for denied access, hazardous, out of sample conditions
TRANSCD (12.11)	TREE	63	Foliage transparency code
TRE_CN	TREE_REGIONAL_BIOMASS	1	Tree sequence number
TREE	SITETREE	10	Tree number
TREE (5.2)	TREE	10	Tree record number
TREECLCD	TREE	23	Tree class code
TREECLCD_NCRS	TREE	106	Tree class code, North Central Research Station
TREECLCD_NERS	TREE	104	Tree class code, Northeastern Research Station
TREECLCD_RMRS	TREE	107	Tree class code, Rocky Mountain Research Station
TREECLCD_SRS	TREE	105	Tree class code, Southern Research Station
TREECOUNT (6.4)	SEEDLING	13	Tree count for seedlings
TREECOUNT_CALC	SEEDLING	21	Tree count used in calculations
TREEGRCD	TREE	26	Tree grade code
TREEHISTCD	TREE	64	Tree history code
TRTCD1 (2.5.17)	COND	44	Stand Treatment 1 code
TRTCD2 (2.5.19)	COND	46	Stand treatment 2 code
TRTCD3 (2.5.21)	COND	48	Stand Treatment 3 code

Column name with (field guide section)	Table name	Location in table	Description
TRTYR1 (2.5.18)	COND	45	Treatment year 1
TRTYR2 (2.5.20)	COND	47	Treatment year 2
TRTYR3 2.5.22)	COND	49	Treatment year 3
TYPE	REF_HABTYP_PUBLICATION	5	Type of publication
TYPGRPCD	REF_FOREST_TYPE	3	Forest type group code
UNCRCD (5.18, 12.5)	TREE	57	Uncompacted live crown ratio
UNITCD	BOUNDARY	5	Survey unit code
UNITCD	COND	5	Survey unit code
UNITCD	COUNTY	2	Survey unit code
UNITCD	PLOT	7	Survey unit code
UNITCD	POP_PLOT_STRATUM_ ASSGN	6	Survey unit code
UNITCD	SEEDLING	5	Survey unit code
UNITCD	SITETREE	6	Survey unit code
UNITCD	SUBPLOT	6	Survey unit code
UNITCD	SUBP_COND	5	Survey unit code
UNITCD	TREE	6	Survey unit code
UNKNOWN_DAMTYP1_PNWRS	TREE	140	Unknown damage type 1, Pacific Northwest Research Station
UNKNOWN_DAMTYP2_PNWRS	TREE	141	Unknown damage type 2, Pacific Northwest Research Station
VALID	REF_HABTYP_DESCRIPTION	6	Valid
VALID	REF_HABTYP_PUBLICATION	6	Valid
VALIDCD	SITETREE	23	Validity code
VALUE	REF_FOREST_TYPE	1	Value
VALUE	REF_UNIT	2	Value
VARIETY	REF_SPECIES	5	Variety
VERSION	REF_FIADB_VERSION	1	Version number
VOL_LOC_GRP	COND	63	Volume location group
VOLBFGRS	TREE	43	Gross board-foot volume in the sawlog portion
VOLBFNET	TREE	42	Net board-foot volume in the sawlog portion
VOLCFGRS	TREE	39	Gross cubic-foot volume
VOLCFNET	TREE	38	Net cubic-foot volume
VOLCFSND	TREE	44	Sound cubic-foot volume
VOLCSGRS	TREE	41	Gross cubic-foot volume in the sawlog portion
VOLCSNET	TREE	40	Net cubic-foot volume in the sawlog portion
W_SPGRPCD	REF_SPECIES	9	West species group code
WATERCD (1.13)	PLOT	19	Water on plot code
WATERDEP (3.8)	SUBPLOT	18	Water or snow depth
WDLDSTEM (5.11)	TREE	37	Woodland tree species current stem count
WEST	REF_SPECIES	26	West
WHERE_CLAUSE	REF_POP_ATTRIBUTE	5	Part of the where clause
WOOD_SPGR_GREENVOL_ DRYWT	REF_SPECIES	49	Green specific gravity wood (green volume and oven-dry weight)
WOOD_SPGR_GREENVOL_ DRYWT_CIT	REF_SPECIES	50	Green specific gravity wood citation
WOOD_SPGR_MC12VOL_DRYWT	REF_SPECIES	57	Wood specific gravity (12 percent moisture content volume and oven-dry weight)
WOOD_SPGR_MC12VOL_DRYWT_ CIT	REF_SPECIES	58	Wood specific gravity (12 percent moisture content volume and oven-dry weight) citation
WOODLAND	REF_SPECIES	27	Woodland species

Appendix B. Forest Inventory and Analysis (FIA) Plot Design Codes and Definitions by FIA Work Unit

FIA work unit	Plot design code (DESIGNCD)	Definition
[a]NRS-NE, [b]NRS-NC, [c]SRS, [d]RMRS, [e]PNWRS	1	National plot design consists of four 24-foot fixed-radius subplots for trees ≥5 inches DBH, and four 6.8-foot fixed-radius microplots for seedlings and trees ≥1 and <5 inches DBH. Subplot 1 is the center plot, and subplots 2, 3, and 4 are located 120.0 feet, horizontal, at azimuths of 360, 120, and 240, respectively. The microplot center is 12 feet east of the subplot center. Four 58.9-foot fixed-radius macroplots are optional. A plot may sample more than one condition. When multiple conditions are encountered, condition boundaries are delineated (mapped).
[a]NRS-NE	101	Various plot designs. Converted from Eastwide Database format, some fields may be null.
	111	Four-subplot design similar to DESIGNCD 1, except the microplot for seedlings is 1/1000 acre (3.7-foot radius). If the plot is used for growth estimates, it is overlaid on a 5 subplot design, where remeasurement of trees (≥5 inches) is on subplot 1 only. Poletimber-sized trees remeasured on a 24-foot radius plot, sawtimber-sized trees remeasured on a 49-foot radius plot. If the plot is not used for growth estimates, it is an initial plot establishment.
	112	DESIGNCD 111, except that if the plot is used for growth estimates, the remeasurement of trees (≥5 inches) is on the 24-foot-radius subplot 1 only, regardless of tree size or previous plot size or type (varied).
	113	DESIGNCD 111, except that if the plot is used for growth estimates, the remeasurement of trees (≥5 inches) is on the 24-foot-radius subplot 1 only, regardless of tree size or previous plot size or type (single subplot 1/5 acre).
	115	DESIGNCD 1. Overlaid on a FHM 4-subplot plot design. These plots are not used in change estimates.
	116	DESIGNCD 1. Overlaid on 1/5 acre plot for all trees ≥5 inches DBH (1/5 acre plot was an initial measurement). Remeasurement of subplot 1 is only on the 24-foot-radius plot for all trees (≥5 inches), regardless of tree size or previous plot size.
	117	DESIGNCD 1. Overlaid on 1/5 acre plot for all trees ≥5 inches DBH (1/5 acre plot was remeasurement). Remeasurement of subplot 1 is only on the 24-foot-radius plot for all trees (≥5 inches), regardless of tree size or previous plot size.
	118	DESIGNCD 1. Overlaid on 10-subplot, variable-radius design. Remeasurement of trees (≥5 inches) on 5 of the 10 subplots; ingrowth based on trees (≥5 inches) that grew onto five 6.8-foot radius subplots.
[b]NRS-NC	301	Various plot designs. Converted from Eastwide Database format, some fields may be null.
	311	Four-subplot design similar to DESIGNCD 1, except the 1/24 acre and 1/300 acre plots have common centers. Conditions are mapped and boundaries may be within the plots.
	312	DESIGNCD 1. Initial plot establishment.
	313	DESIGNCD 311. Overlaid on previous plots, no remeasurements.
	314	DESIGNCD 1. Overlaid on previous plots, no remeasurements.
	315	DESIGNCD 311. Overlaid on same design. Only trees ≥5 inches DBH are remeasured.
	316	DESIGNCD 1. Overlaid on DESIGNCD 311 Only trees ≥5 inches DBH are remeasured.
	317	DESIGNCD 1. Overlaid on DESIGNCD 326. Only the first 5 points (trees ≥5 inches DBH) and first 3, 1/300 acre plots (trees ≥1 and <5 inches DBH) are remeasured, but conditions were not re-mapped.

FIA work unit	Plot design code (DESIGNCD)	Definition
	318	DESIGNCD 311. Overlaid on DESIGNCD 325. Only the first 5 points (trees ≥5 inches DBH) and first 3, 1/300 acre plots (trees ≥1 and <5 inches DBH) are remeasured.
	319	DESIGNCD 1. Overlaid on DESIGNCD 325. Only the first 5 points (trees ≥5 inches DBH) and first 3, 1/300 acre plots (trees ≥1 and <5 inches DBH) are remeasured.
	320	DESIGNCD 311. Overlaid on modified DESIGNCD 325. Only the first 5 points (trees ≥5 inches DBH) and first 3 1/300 acre plots (trees ≥1 and <5 inches DBH) are remeasured.
	321	DESIGNCD 1. Overlaid on modified DESIGNCD 325. Only the first 5 points (trees ≥5 inches DBH) and first 3 1/300 acre plots (trees ≥1 and <5 inches DBH) are remeasured.
	322	DESIGNCD 311. Overlaid on DESIGNCD 327. Only the first 5 points (trees ≥5 inches DBH) and first 3, 1/300 acre plots (trees ≥1 and <5 inches DBH) are remeasured.
	323	DESIGNCD 1. Overlaid on DESIGNCD 327. Only the first 5 points (trees ≥5 inches DBH) and first 3 1/300 acre plots (trees ≥1 and <5 inches DBH) are remeasured.
	325	Ten variable-radius, 37.5 BAF points, 70 feet apart, for trees ≥5 inches DBH and 10, 1/300 acre plots for seedlings and trees ≥1 and <5 inches DBH. Point and plot center were coincident. Conditions were not mapped. Instead, points were rotated into forest or nonforest based on the condition at point center.
	326	Ten variable-radius, 37.5 BAF points, 70 feet apart, for trees ≥5 and <17.0 inches DBH, 10 1/24 acre plots for trees ≥17.0 inches DBH, and 10, 1/300 acre plots for seedlings and trees ≥1 and <5 inches DBH. Point and plot center were coincident. Conditions were mapped.
	327	Ten variable-radius, 37.5 BAF points, 70 feet apart, for trees ≥5 inches DBH and 10, 1/300 acre plots for seedlings and trees ≥1 and <5 inches DBH. Point and plot center were coincident. Conditions were not mapped. Instead, points were rotated into forest or nonforest based on the condition at point center. Diameters were estimated with a model, but all dead and cut trees were recorded.
	328	DESIGNCD 1. Overlaid on DESIGNCD 311. All trees and saplings are remeasured.
[c]SRS	210	Other plot design installed by previous research stations within the 13-State Southern area not described by DESIGNCD 211-219.
	211	Ten variable-radius, 37.5 BAF points, 70 feet apart. Remeasure first 3 points of same design or new/replacement plot.
	212	Five variable-radius, 37.5 BAF points, 70 feet apart. Remeasure first 5 points of DESIGNCD 211 or new/replacement plot.
	213	Five variable-radius, 37.5 BAF points, 70 feet apart. Remeasure DESIGNCD 212.
	214	Ten variable-radius, 37.5 BAF points, 66 feet apart. Remeasure same design or new/replacement plot.
	215	Five variable-radius, 37.5 BAF points, 66 feet apart. Remeasure first 5 points of DESIGNCD 214 or new/replacement plot.
	216	Ten variable-radius, 37.5 BAF points, 66 feet apart. Remeasure DESIGNCD 215.
	217	Five point cluster plot, point 1 is 1/5th acre sawtimber plot and 1/10th acre poletimber plot, points 2-5 are 37.5 BAF prism points. No remeasurement.
	218	Remeasurement of DESIGNCD 217, point 1 only. Used only for change estimates.
	219	Three point, 2.5 BAF metric prism plot, points 25 meters apart. Remeasure same design or new/replacement plot.

FIA work unit	Plot design code (DESIGNCD)	Definition
	220	Four 1/24 acre plots for trees ≥5 inches DBH and 4, 1/300 acre plots for seedlings and trees ≥1 and <5 inches DBH. The 1/24 acre and 1/300 acre plots have common centers. Conditions are mapped and boundaries may be within the plots. Remeasurement plot not described by 221-229.
	221	DESIGNCD 220. Remeasure same design or new/replacement plot.
	222	DESIGNCD 220. Overlaid on and remeasurement of DESIGNCD 212 or 213.
	223	DESIGNCD 220. Overlaid on and remeasurement of first 5 points of DESIGNCD 214 or 216.
	230	DESIGNCD 1. Remeasurement plot not described by DESIGNCD 231-239.
	231	DESIGNCD 1. Overlaid on and remeasurement of DESIGNCD 212 or DESIGNCD 213.
	232	DESIGNCD 1. Overlaid on and remeasurement of first 5 points of DESIGNCD 214 or 216.
	233	DESIGNCD 1. Overlaid on and remeasurement of DESIGNCD 220, 221, 222, or 223
	240	DESIGNCD 1. Collected in metric and converted to English in the database. Remeasurement not described by 241-249.
	241	DESIGNCD 1. Collected in metric and converted to English in the database. Remeasure same design or new/replacement plot.
	242	DESIGNCD 1. Overlaid on and remeasurement of DESIGNCD 219. Collected in metric and converted to English in the database.
	299	Other plot design not described in DESIGNCD 200-298.
[d]RMRS	403	One 1/10th acre fixed-radius plot divided into 4 quadrants and four 1/300th acre fixed-radius microplots. Timber and woodland tree species <5.0 inches DRC tallied on microplot.
	404	One 1/20th acre fixed-radius plot divided into 4 quadrants and four 1/300th acre fixed-radius microplots. Timber and woodland tree species <5.0 inches DRC tallied on microplot.
	405	One 1/5th acre fixed-radius plot divided into 4 quadrants and four 1/300th acre fixed-radius microplots. Timber and woodland tree species <5.0 inches DRC tallied on microplot.
	410	40 BAF variable-radius plots and 1/300th acre fixed-radius microplots; number of microplots = number of points installed. Timber tree species <5.0 inches DBH; woodland tree species <3.0 inches DRC measured on microplot.
	411	40 BAF variable-radius plots and 1/300th acre fixed-radius microplots; 3 microplots installed on points 1, 2, and 3. Timber tree species <5.0 inches DBH; woodland tree species <3.0 inches DRC measured on microplot.
	412	40 BAF variable-radius plots and 1/300th acre fixed-radius microplots; 3 microplots installed on points 1, 2, and 5. Timber tree species <5.0 inches DBH; woodland tree species <3.0 inches DRC measured on microplot.
	413	20 BAF variable-radius plots and 1/300th acre fixed-radius microplots; number of microplots = number of points installed. Timber tree species <5.0 inches DBH; woodland tree species <3.0 inches DRC measured on microplot.
	414	20 BAF variable-radius plots and 1/300th acre fixed-radius microplots; 3 microplots installed on points 1, 2, and 3. Timber tree species <5.0 inches DBH; woodland tree species <3.0 inches DRC measured on microplot.
	415	20 BAF variable-radius plots and 1/300th acre fixed-radius microplots; 3 microplots installed on points 1, 2, and 5. Timber tree species <5.0 inches DBH; woodland tree species <3.0 inches DRC measured on microplot.
	420	One 1/10th acre fixed-radius plot and one centered 1/100th acre microplot. Timber tree species <5.0 inches DBH; woodland tree species <3.0 inches DRC measured on microplot.

FIA work unit	Plot design code (DESIGNCD)	Definition
	421	One 1/20th acre fixed-radius plot and one centered 1/100th acre microplot. Timber tree species <5.0 inches DBH; woodland tree species <3.0 inches DRC measured on microplot.
	422	One 1/5th acre fixed-radius plot and one centered 1/100th acre microplot. Timber tree species <5.0 inches DBH; woodland tree species <3.0 inches DRC measured on microplot.
	423	One 1/10th acre fixed-radius plot divided into 4 quadrants and four 1/300th acre fixed-radius microplots. Timber tree species <5.0 inches DBH; woodland tree species <3.0 inches DRC measured on microplot.
	424	One 1/20th acre fixed-radius plot divided into 4 quadrants and four 1/300th acre fixed-radius microplots. Timber tree species <5.0 inches DBH; woodland tree species <3.0 inches DRC measured on microplot.
	425	One 1/5th acre fixed-radius plot divided into 4 quadrants and four 1/300th acre fixed-radius microplots. Timber tree species <5.0 inches DBH; woodland tree species <3.0 inches DRC measured on microplot.
[e]PNWRS	501	DESIGNCD 1 with optional macroplot. Trees ≥24 inches DBH are tallied on macroplot.
	502	DESIGNCD 1 with optional macroplot. Trees ≥30 inches DBH are tallied on macroplot.
	503	DESIGNCD 1 with optional macroplot. Trees ≥4 inches DBH are tallied on macroplot. Trees ≥32 inches DBH are tallied on one 1-hectare plot.
	504	DESIGNCD 1 with optional macroplot. Trees ≥24 inches DBH are tallied on macroplot. Trees ≥48 inches DBH are tallied on one 1-hectare plot.
	505	DESIGNCD 1 with optional macroplot. Trees ≥30 inches DBH are tallied on macroplot. Trees ≥48 inches DBH are tallied on one 1-hectare plot.
	550	Five 30.5 BAF points for trees ≥5 inches and <35.4 inches DBH; five 55.8 foot fixed-radius plots for trees ≥35.4 inches DBH; and five 7.7-foot fixed-radius plots for seedlings and saplings <5 inches DBH. Point and plot centers are coincident. Conditions are mapped.
	551	Five 20 BAF points for trees ≥5 inches and <35.4 inches DBH; five 55.6 foot fixed-radius plots for trees ≥35.4 inches DBH; and five 9.7-foot fixed-radius plots for seedlings and saplings <5 inches DBH. Point and plot centers are coincident. Conditions are mapped.
	552	Five 30 BAF points for trees ≥5 inches and <35.4 inches DBH; five 55.6-foot fixed-radius plots for trees ≥35.4 inches DBH; and five 7.9-foot fixed-radius plots for seedlings and saplings <5 inches DBH. Point and plot centers are coincident. Conditions are mapped.
	553	Four 1/24 acre plots for live trees and four 58.9-foot fixed-radius plots for trees ≥11.8 inches DBH. Plot centers are coincident. Conditions are mapped.
	554	Four 1/24 acre plots for live trees and four 58.9-foot fixed-radius plots for trees ≥19.7 inches DBH. Plot centers are coincident. Conditions are mapped.
	555	Five 30.5 BAF points for trees ≥6.9 inches and <35.4 inches DBH; five 55.8-foot fixed-radius plots for trees ≥35.4 inches DBH; and five 10.8-foot fixed-radius plots for seedlings and saplings <6.9 inches DBH. Point and plot centers are coincident. Conditions are mapped.
	556	Five 30.5 BAF points for trees ≥6.9 inches and <35.4 inches DBH; five 55.8-foot fixed-radius plots for trees ≥35.4 inches DBH; five 10.8-foot fixed-radius plots for saplings ≥5 inches and <6.9 inches DBH; and the northeast quadrant of each of the five 10.8-foot fixed-radius plots for trees <5 inches DBH. Point and plot centers are coincident. Conditions are not mapped.
	557	Five 40 BAF points for trees ≥5 inches DBH; and five 6.9-foot fixed-radius plots for saplings ≥1 and <5 inches DBH. Point and plot centers are coincident. Conditions are not mapped.

FIA work unit	Plot design code (DESIGNCD)	Definition
	558	Three 30.5 BAF points for trees ≥6.9 inches and <35.4 inches DBH; three 55.8-foot fixed-radius plots for trees ≥35.4 inches DBH; three 10.8-foot fixed-radius plots for saplings ≥5 inches and <6.9 inches DBH; and the northeast quadrant of each of the three 10.8-foot fixed-radius plots for trees <5 inches DBH. Point and plot centers are coincident. Conditions are mapped, only condition class 1 measured. Overlaid on and remeasurement of same design.
	559	Four 40 BAF points for trees ≥5 inches DBH; and four 6.9-foot fixed-radius plots for saplings ≥1 and <5 inches DBH. Point and plot centers are coincident. Conditions are mapped, only condition class 1 measured. Overlaid on and remeasurement of same design.
[a]NRS-NE, [b]NRS-NC, [c]SRS, [d]RMRS, [e]PNWRS	999	A plot record created to represent reserved or other nonsampled or undersampled areas where there were no ground plots; the plot has no design type; rather, it is a placeholder for area estimates. In all cases where DESIGNCD 999 plots are present, they are only used for estimates of area; they are not used in estimates of numbers of trees, volume or change (i.e., tree level estimates).

[a]Northern Research Station – previously Northeastern
[b]Northern Research Station – previously North Central
[c]Southern Research Station
[d]Rocky Mountain Research Station
[e]Pacific Northwest Research Station

Other acronyms and definitions:
BAF – basal area factor
DRC – diameter at root collar
Sawtimber-sized trees – softwoods ≥9 inches DBH, hardwoods ≥11 inches DBH.
Poletimber-sized trees – softwoods ≥5 inches and <9 inches DBH, hardwoods ≥5 inches and <11 inches DBH

Appendix C. State, Survey Unit, and County Codes

State Code: 1 **State Name:** Alabama **State Abbreviation:** AL **Region/Station Code:** 33

Survey Unit Code: 1 **Survey Unit Name**: Southwest-South

County code and county name					
3	Baldwin	53	Escambia	129	Washington
39	Covington	97	Mobile		

Survey Unit Code: 2 **Survey Unit Name**: Southwest-North

County code and county name							
23	Choctaw	35	Conecuh	99	Monroe	131	Wilcox
25	Clarke	91	Marengo	119	Sumter		

Survey Unit Code: 3 **Survey Unit Name**: Southeast

County code and county name							
1	Autauga	31	Coffee	67	Henry	109	Pike
5	Barbour	41	Crenshaw	69	Houston	113	Russell
11	Bullock	45	Dale	81	Lee	123	Tallapoosa
13	Butler	47	Dallas	85	Lowndes		
17	Chambers	51	Elmore	87	Macon		
21	Chilton	61	Geneva	101	Montgomery		

Survey Unit Code: 4 **Survey Unit Name**: West Central

County code and county name					
7	Bibb	65	Hale	105	Perry
57	Fayette	75	Lamar	107	Pickens
63	Greene	93	Marion	125	Tuscaloosa

Survey Unit Code: 5 **Survey Unit Name**: North Central

County code and county name							
9	Blount	29	Cleburne	73	Jefferson	121	Talladega
15	Calhoun	37	Coosa	111	Randolph	127	Walker
19	Cherokee	43	Cullman	115	St. Clair	133	Winston
27	Clay	55	Etowah	117	Shelby		

Survey Unit Code: 6 **Survey Unit Name**: North

County code and county name							
33	Colbert	71	Jackson	83	Limestone	103	Morgan
49	DeKalb	77	Lauderdale	89	Madison		
59	Franklin	79	Lawrence	95	Marshall		

State Code: 2 **State Name:** Alaska **State Abbreviation:** AK **Region/Station Code:** 27

Survey Unit Code: 1 **Survey Unit Name**: Alaska

County code and county name

13	Aleutians East Borough	170	Matanuska-Susitna Borough
16	Aleutians West Census Area	180	Nome Census Area
20	Anchorage Borough	185	North Slope Borough
50	Bethel Census Area	188	Northwest Arctic Borough
60	Bristol Bay Borough	201	Prince of Wales-Outer Ketchikan Census Area
68	Denali Borough	220	Sitka Borough
70	Dillingham Census Area	232	Skagway-Hoonah-Angoon Census Area
90	Fairbanks North Star Borough	240	Southeast Fairbanks Census Area
100	Haines Borough	261	Valdez-Cordova Census Area
110	Juneau Borough	270	Wade Hampton Census Area
122	Kenai Peninsula Borough	280	Wrangell-Petersburg Census Area
130	Ketchikan Gateway Borough	282	Yakutat Borough
150	Kodiak Island Borough	290	Yukon-Koyukuk Census Area
164	Lake and Peninsula Borough		

State Code: 4 **State Name:** Arizona **State Abbreviation:** AZ **Region/Station Code:** 22

Survey Unit Code: 1 **Survey Unit Name**: Southern

County code and county name

3	Cochise	12	La Paz	21	Pinal
9	Graham	13	Maricopa	23	Santa Cruz
11	Greenlee	19	Pima	27	Yuma

Survey Unit Code: 2 **Survey Unit Name**: Northern

County code and county name

1	Apache	7	Gila	17	Navajo
5	Coconino	15	Mohave	25	Yavapai

State Code: 5 **State Name:** Arkansas **State Abbreviation:** AR **Region/Station Code:** 33

Survey Unit Code: 1 **Survey Unit Name**: South Delta

County code and county name							
1	Arkansas	69	Jefferson	85	Lonoke	117	Prairie
17	Chicot	77	Lee	95	Monroe		
41	Desha	79	Lincoln	107	Phillips		

Survey Unit Code: 2 **Survey Unit Name**: North Delta

County code and county name							
21	Clay	37	Cross	75	Lawrence	123	St. Francis
31	Craighead	55	Greene	93	Mississippi	147	Woodruff
35	Crittenden	67	Jackson	111	Poinsett		

Survey Unit Code: 3 **Survey Unit Name**: Southwest

County code and county name							
3	Ashley	27	Columbia	59	Hot Spring	99	Nevada
11	Bradley	39	Dallas	61	Howard	103	Ouachita
13	Calhoun	43	Drew	73	Lafayette	109	Pike
19	Clark	53	Grant	81	Little River	133	Sevier
25	Cleveland	57	Hempstead	91	Miller	139	Union

Survey Unit Code: 4 **Survey Unit Name**: Ouachita

County code and county name							
51	Garland	105	Perry	125	Saline	149	Yell
83	Logan	113	Polk	127	Scott		
97	Montgomery	119	Pulaski	131	Sebastian		

Survey Unit Code: 5 **Survey Unit Name**: Ozark

County code and county name							
5	Baxter	33	Crawford	71	Johnson	129	Searcy
7	Benton	45	Faulkner	87	Madison	135	Sharp
9	Boone	47	Franklin	89	Marion	137	Stone
15	Carroll	49	Fulton	101	Newton	141	Van Buren
23	Cleburne	63	Independence	115	Pope	143	Washington
29	Conway	65	Izard	121	Randolph	145	White

State Code: 6 **State Name:** California **State Abbreviation:** CA **Region/Station Code:** 26

Survey Unit Code: 1 **Survey Unit Name**: North Coast

County code and county name							
15	Del Norte	23	Humboldt	45	Mendocino	97	Sonoma

Survey Unit Code: 2 **Survey Unit Name**: North Interior

County code and county name					
35	Lassen	89	Shasta	105	Trinity
49	Modoc	93	Siskiyou		

Survey Unit Code: 3 **Survey Unit Name**: Sacramento

County code and county name							
7	Butte	33	Lake	63	Plumas	103	Tehama
11	Colusa	55	Napa	67	Sacramento	113	Yolo
17	El Dorado	57	Nevada	91	Sierra	115	Yuba
21	Glenn	61	Placer	101	Sutter		

Survey Unit Code: 4 **Survey Unit Name**: Central Coast

County code and county name							
1	Alameda	69	San Benito	83	Santa Barbara	111	Ventura
13	Contra Costa	75	San Francisco	85	Santa Clara		
41	Marin	79	San Luis Obispo	87	Santa Cruz		
53	Monterey	81	San Mateo	95	Solano		

Survey Unit Code: 5 **Survey Unit Name**: San Joaquin

County code and county name							
3	Alpine	29	Kern	47	Merced	107	Tulare
5	Amador	31	Kings	51	Mono	109	Tuolumne
9	Calaveras	39	Madera	77	San Joaquin		
19	Fresno	43	Mariposa	99	Stanislaus		

Survey Unit Code: 6 **Survey Unit Name**: Southern

County code and county name							
25	Imperial	37	Los Angeles	65	Riverside	73	San Diego
27	Inyo	59	Orange	71	San Bernardino		

State Code: 8 **State Name:** Colorado **State Abbreviation:** CO **Region/Station Code:** 22

Survey Unit Code: 1 **Survey Unit Name**: Northern Front Range

County code and county name							
13	Boulder	39	Elbert	59	Jefferson	93	Park
19	Clear Creek	41	El Paso	65	Lake	119	Teller
35	Douglas	47	Gilpin	69	Larimer		

Survey Unit Code: 2 **Survey Unit Name**: Southern Front Range

County code and county name							
15	Chaffee	27	Custer	55	Huerfano	101	Pueblo
23	Costilla	43	Fremont	71	Las Animas		

Survey Unit Code: 3 **Survey Unit Name**: West Central

County code and county name							
3	Alamosa	51	Gunnison	97	Pitkin	111	San Juan
21	Conejos	53	Hinsdale	105	Rio Grande	117	Summit
37	Eagle	57	Jackson	107	Routt		
49	Grand	79	Mineral	109	Saguache		

Survey Unit Code: 4 **Survey Unit Name**: Western

County code and county name							
7	Archuleta	45	Garfield	81	Moffat	91	Ouray
29	Delta	67	La Plata	83	Montezuma	103	Rio Blanco
33	Dolores	77	Mesa	85	Montrose	113	San Miguel

Survey Unit Code: 5 **Survey Unit Name**: Eastern

County code and county name							
1	Adams	25	Crowley	75	Logan	115	Sedgwick
5	Arapahoe	31	Denver	87	Morgan	121	Washington
9	Baca	61	Kiowa	89	Otero	123	Weld
11	Bent	63	Kit Carson	95	Phillips	125	Yuma
17	Cheyenne	73	Lincoln	99	Prowers		

State Code: 9 **State Name:** Connecticut **State Abbreviation:** CT **Region/Station Code:** 24

Survey Unit Code: 1 **Survey Unit Name**: Connecticut

County code and county name							
1	Fairfield	5	Litchfield	9	New Haven	13	Tolland
3	Hartford	7	Middlesex	11	New London	15	Windham

State Code: 10 **State Name:** Delaware **State Abbreviation:** DE **Region/Station Code:** 24

Survey Unit Code: 1 **Survey Unit Name**: Delaware

County code and county name					
1	Kent	3	New Castle	5	Sussex

State Code: 11 **State Name:** District of Columbia **State Abbrev.:** DC **Region/Station Code:** 24

State Code: 12 **State Name:** Florida **State Abbreviation:** FL **Region/Station Code:** 33

Survey Unit Code: 1 **Survey Unit Name**: Northeastern

County code and county name							
1	Alachua	31	Duval	79	Madison	123	Taylor
3	Baker	35	Flagler	83	Marion	125	Union
7	Bradford	41	Gilchrist	89	Nassau	127	Volusia
19	Clay	47	Hamilton	107	Putnam		
23	Columbia	67	Lafayette	109	St. Johns		
29	Dixie	75	Levy	121	Suwannee		

Survey Unit Code: 2 **Survey Unit Name**: Northwestern

County code and county name							
5	Bay	39	Gadsden	65	Jefferson	113	Santa Rosa
13	Calhoun	45	Gulf	73	Leon	129	Wakulla
33	Escambia	59	Holmes	77	Liberty	131	Walton
37	Franklin	63	Jackson	91	Okaloosa	133	Washington

Survey Unit Code: 3 **Survey Unit Name**: Central

County code and county name							
9	Brevard	55	Highlands	93	Okeechobee	105	Polk
17	Citrus	57	Hillsborough	95	Orange	111	St. Lucie
27	DeSoto	61	Indian River	97	Osceola	115	Sarasota
49	Hardee	69	Lake	101	Pasco	117	Seminole
53	Hernando	81	Manatee	103	Pinellas	119	Sumter

Survey Unit Code: 4 **Survey Unit Name**: Southern

County code and county name							
11	Broward	25	Dade	71	Lee	99	Palm Beach
15	Charlotte	43	Glades	85	Martin		
21	Collier	51	Hendry	87	Monroe		

State Code: 13 **State Name:** Georgia **State Abbreviation:** GA **Region/Station Code:** 33

Survey Unit Code: 1 **Survey Unit Name**: Southeastern

County code and county name							
1	Appling	51	Chatham	161	Jeff Davis	251	Screven
3	Atkinson	65	Clinch	165	Jenkins	267	Tattnall
5	Bacon	69	Coffee	167	Johnson	271	Telfair
25	Brantley	91	Dodge	175	Laurens	279	Toombs
29	Bryan	101	Echols	179	Liberty	283	Treutlen
31	Bulloch	103	Effingham	183	Long	299	Ware
39	Camden	107	Emanuel	191	McIntosh	305	Wayne
43	Candler	109	Evans	209	Montgomery	309	Wheeler
49	Charlton	127	Glynn	229	Pierce		

Survey Unit Code: 2 **Survey Unit Name**: Southwestern

County code and county name							
7	Baker	81	Crisp	173	Lanier	277	Tift
17	Ben Hill	87	Decatur	185	Lowndes	287	Turner
19	Berrien	93	Dooly	201	Miller	315	Wilcox
27	Brooks	99	Early	205	Mitchell	321	Worth
71	Colquitt	131	Grady	253	Seminole		
75	Cook	155	Irwin	275	Thomas		

Survey Unit Code: 3 **Survey Unit Name**: Central

County code and county name							
9	Baldwin	141	Hancock	211	Morgan	265	Taliaferro
21	Bibb	145	Harris	215	Muscogee	269	Taylor
23	Bleckley	153	Houston	225	Peach	273	Terrell
33	Burke	159	Jasper	231	Pike	289	Twiggs
35	Butts	163	Jefferson	235	Pulaski	293	Upson
37	Calhoun	169	Jones	237	Putnam	301	Warren
53	Chattahoochee	171	Lamar	239	Quitman	303	Washington
61	Clay	177	Lee	243	Randolph	307	Webster
73	Columbia	181	Lincoln	245	Richmond	317	Wilkes
79	Crawford	189	McDuffie	249	Schley	319	Wilkinson
95	Dougherty	193	Macon	259	Stewart		
125	Glascock	197	Marion	261	Sumter		
133	Greene	207	Monroe	263	Talbot		

Survey Unit Code: 4 **Survey Unit Name**: North Central

County code and county name							
11	Banks	97	Douglas	143	Haralson	219	Oconee
13	Barrow	105	Elbert	147	Hart	221	Oglethorpe
45	Carroll	113	Fayette	149	Heard	223	Paulding
59	Clarke	117	Forsyth	151	Henry	233	Polk
63	Clayton	119	Franklin	157	Jackson	247	Rockdale
67	Cobb	121	Fulton	195	Madison	255	Spalding
77	Coweta	135	Gwinnett	199	Meriwether	285	Troup
89	DeKalb	139	Hall	217	Newton	297	Walton

Georgia cont.

Georgia cont.

Survey Unit Code: 5 **Survey Unit Name**: Northern

County code and county name							
15	Bartow	111	Fannin	213	Murray	295	Walker
47	Catoosa	115	Floyd	227	Pickens	311	White
55	Chattooga	123	Gilmer	241	Rabun	313	Whitfield
57	Cherokee	129	Gordon	257	Stephens		
83	Dade	137	Habersham	281	Towns		
85	Dawson	187	Lumpkin	291	Union		

State Code: 15 **State Name:** Hawaii **State Abbreviation:** HI **Region/Station Code:** **26**

County code and county name					
1	Hawaii	5	Kalawao	9	Maui
3	Honolulu	7	Kauai		

State Code: 16 **State Name:** Idaho **State Abbreviation:** ID **Region/Station Code:** 22

Survey Unit Code: 1 **Survey Unit Name**: Northern

County code and county name							
9	Benewah	35	Clearwater	57	Latah	79	Shoshone
17	Bonner	49	Idaho	61	Lewis		
21	Boundary	55	Kootenai	69	Nez Perce		

Survey Unit Code: 2 **Survey Unit Name**: Southwestern

County code and county name							
1	Ada	27	Canyon	73	Owyhee	87	Washington
3	Adams	39	Elmore	75	Payette		
15	Boise	45	Gem	85	Valley		

Survey Unit Code: 3 **Survey Unit Name**: Southeastern

County code and county name							
5	Bannock	25	Camas	43	Fremont	65	Madison
7	Bear Lake	29	Caribou	47	Gooding	67	Minidoka
11	Bingham	31	Cassia	51	Jefferson	71	Oneida
13	Blaine	33	Clark	53	Jerome	77	Power
19	Bonneville	37	Custer	59	Lemhi	81	Teton
23	Butte	41	Franklin	63	Lincoln	83	Twin Falls

State Code: 17 **State Name:** Illinois **State Abbreviation:** IL **Region/Station Code:** 23

Survey Unit Code: 1 **Survey Unit Name**: Southern

County code and county name							
3	Alexander	69	Hardin	145	Perry	165	Saline
55	Franklin	77	Jackson	151	Pope	181	Union
59	Gallatin	87	Johnson	153	Pulaski	193	White
65	Hamilton	127	Massac	157	Randolph	199	Williamson

Survey Unit Code: 2 **Survey Unit Name**: Claypan

County code and county name							
5	Bond	47	Edwards	101	Lawrence	163	St. Clair
13	Calhoun	49	Effingham	117	Macoupin	173	Shelby
23	Clark	51	Fayette	119	Madison	185	Wabash
25	Clay	61	Greene	121	Marion	189	Washington
27	Clinton	79	Jasper	133	Monroe	191	Wayne
33	Crawford	81	Jefferson	135	Montgomery		
35	Cumberland	83	Jersey	159	Richland		

Survey Unit Code: 3 **Survey Unit Name**: Prairie

County code and county name							
1	Adams	53	Ford	105	Livingston	149	Pike
7	Boone	57	Fulton	107	Logan	155	Putnam
9	Brown	63	Grundy	109	McDonough	161	Rock Island
11	Bureau	67	Hancock	111	McHenry	167	Sangamon
15	Carroll	71	Henderson	113	McLean	169	Schuyler
17	Cass	73	Henry	115	Macon	171	Scott
19	Champaign	75	Iroquois	123	Marshall	175	Stark
21	Christian	85	Jo Daviess	125	Mason	177	Stephenson
29	Coles	89	Kane	129	Menard	179	Tazewell
31	Cook	91	Kankakee	131	Mercer	183	Vermilion
37	DeKalb	93	Kendall	137	Morgan	187	Warren
39	De Witt	95	Knox	139	Moultrie	195	Whiteside
41	Douglas	97	Lake	141	Ogle	197	Will
43	DuPage	99	La Salle	143	Peoria	201	Winnebago
45	Edgar	103	Lee	147	Piatt	203	Woodford

State Code: 18 **State Name:** Indiana **State Abbreviation:** IN **Region/Station Code:** 23

Survey Unit Code: 1 **Survey Unit Name**: Lower Wabash

County code and county name							
21	Clay	83	Knox	129	Posey	165	Vermillion
27	Daviess	101	Martin	133	Putnam	167	Vigo
51	Gibson	121	Parke	153	Sullivan		
55	Greene	125	Pike	163	Vanderburgh		

Survey Unit Code: 2 **Survey Unit Name**: Knobs

County code and county name							
13	Brown	61	Harrison	117	Orange	173	Warrick
19	Clark	71	Jackson	119	Owen	175	Washington
25	Crawford	93	Lawrence	123	Perry		
37	Dubois	105	Monroe	143	Scott		
43	Floyd	109	Morgan	147	Spencer		

Survey Unit Code: 3 **Survey Unit Name**: Upland Flats

County code and county name					
29	Dearborn	77	Jefferson	137	Ripley
41	Fayette	79	Jennings	155	Switzerland
47	Franklin	115	Ohio	161	Union

Survey Unit Code: 4 **Survey Unit Name**: Northern

County code and county name							
1	Adams	45	Fountain	87	Lagrange	139	Rush
3	Allen	49	Fulton	89	Lake	141	St. Joseph
5	Bartholomew	53	Grant	91	La Porte	145	Shelby
7	Benton	57	Hamilton	95	Madison	149	Starke
9	Blackford	59	Hancock	97	Marion	151	Steuben
11	Boone	63	Hendricks	99	Marshall	157	Tippecanoe
15	Carroll	65	Henry	103	Miami	159	Tipton
17	Cass	67	Howard	107	Montgomery	169	Wabash
23	Clinton	69	Huntington	111	Newton	171	Warren
31	Decatur	73	Jasper	113	Noble	177	Wayne
33	De Kalb	75	Jay	127	Porter	179	Wells
35	Delaware	81	Johnson	131	Pulaski	181	White
39	Elkhart	85	Kosciusko	135	Randolph	183	Whitley

State Code: 19 **State Name:** Iowa **State Abbreviation:** IA **Region/Station Code:** 23

Survey Unit Code: 1 **Survey Unit Name**: Northeastern

County code and county name							
5	Allamakee	31	Cedar	65	Fayette	105	Jones
11	Benton	37	Chickasaw	67	Floyd	113	Linn
13	Black Hawk	43	Clayton	75	Grundy	131	Mitchell
17	Bremer	45	Clinton	89	Howard	163	Scott
19	Buchanan	55	Delaware	97	Jackson	171	Tama
23	Butler	61	Dubuque	103	Johnson	191	Winneshiek

Survey Unit Code: 2 **Survey Unit Name**: Southeastern

County code and county name							
7	Appanoose	83	Hardin	121	Madison	177	Van Buren
15	Boone	87	Henry	123	Mahaska	179	Wapello
39	Clarke	95	Iowa	125	Marion	181	Warren
49	Dallas	99	Jasper	127	Marshall	183	Washington
51	Davis	101	Jefferson	135	Monroe	185	Wayne
53	Decatur	107	Keokuk	139	Muscatine	187	Webster
57	Des Moines	111	Lee	153	Polk		
77	Guthrie	115	Louisa	157	Poweshiek		
79	Hamilton	117	Lucas	169	Story		

Survey Unit Code: 3 **Survey Unit Name**: Southwestern

County code and county name							
1	Adair	47	Crawford	133	Monona	165	Shelby
3	Adams	71	Fremont	137	Montgomery	173	Taylor
9	Audubon	73	Greene	145	Page	175	Union
27	Carroll	85	Harrison	155	Pottawattamie	193	Woodbury
29	Cass	129	Mills	159	Ringgold		

Survey Unit Code: 4 **Survey Unit Name**: Northwestern

County code and county name							
21	Buena Vista	63	Emmet	119	Lyon	161	Sac
25	Calhoun	69	Franklin	141	O'Brien	167	Sioux
33	Cerro Gordo	81	Hancock	143	Osceola	189	Winnebago
35	Cherokee	91	Humboldt	147	Palo Alto	195	Worth
41	Clay	93	Ida	149	Plymouth	197	Wright
59	Dickinson	109	Kossuth	151	Pocahontas		

State Code: 20 **State Name:** Kansas **State Abbreviation:** KS **Region/Station Code:** 23

Survey Unit Code: 1 **Survey Unit Name**: Northeastern

County code and county name							
5	Atchison	59	Franklin	117	Marshall	177	Shawnee
13	Brown	61	Geary	121	Miami	197	Wabaunsee
27	Clay	85	Jackson	131	Nemaha	201	Washington
41	Dickinson	87	Jefferson	139	Osage	209	Wyandotte
43	Doniphan	91	Johnson	149	Pottawatomie		
45	Douglas	103	Leavenworth	161	Riley		

Survey Unit Code: 2 **Survey Unit Name**: Southeastern

County code and county name							
1	Allen	21	Cherokee	99	Labette	133	Neosho
3	Anderson	31	Coffey	107	Linn	205	Wilson
11	Bourbon	35	Cowley	111	Lyon	207	Woodson
15	Butler	37	Crawford	115	Marion		
17	Chase	49	Elk	125	Montgomery		
19	Chautauqua	73	Greenwood	127	Morris		

Survey Unit Code: 3 **Survey Unit Name**: Western

County code and county name							
7	Barber	71	Greeley	129	Morton	171	Scott
9	Barton	75	Hamilton	135	Ness	173	Sedgwick
23	Cheyenne	77	Harper	137	Norton	175	Seward
25	Clark	79	Harvey	141	Osborne	179	Sheridan
29	Cloud	81	Haskell	143	Ottawa	181	Sherman
33	Comanche	83	Hodgeman	145	Pawnee	183	Smith
39	Decatur	89	Jewell	147	Phillips	185	Stafford
47	Edwards	93	Kearny	151	Pratt	187	Stanton
51	Ellis	95	Kingman	153	Rawlins	189	Stevens
53	Ellsworth	97	Kiowa	155	Reno	191	Sumner
55	Finney	101	Lane	157	Republic	193	Thomas
57	Ford	105	Lincoln	159	Rice	195	Trego
63	Gove	109	Logan	163	Rooks	199	Wallace
65	Graham	113	McPherson	165	Rush	203	Wichita
67	Grant	119	Meade	167	Russell		
69	Gray	123	Mitchell	169	Saline		

State Code: 21 **State Name:** Kentucky **State Abbreviation:** KY **Region/Station Code:** 33

Survey Unit Code: 1 **Survey Unit Name**: Eastern

County code and county name							
71	Floyd	119	Knott	133	Letcher	193	Perry
95	Harlan	131	Leslie	159	Martin	195	Pike

Survey Unit Code: 2 **Survey Unit Name**: Northern Cumberland

County code and county name							
19	Boyd	115	Johnson	165	Menifee	237	Wolfe
43	Carter	127	Lawrence	175	Morgan		
63	Elliott	135	Lewis	197	Powell		
89	Greenup	153	Magoffin	205	Rowan		

Survey Unit Code: 3 **Survey Unit Name**: Southern Cumberland

County code and county name							
13	Bell	65	Estill	125	Laurel	189	Owsley
25	Breathitt	109	Jackson	129	Lee	203	Rockcastle
51	Clay	121	Knox	147	McCreary	235	Whitley

Survey Unit Code: 4 **Survey Unit Name**: Bluegrass

County code and county name							
5	Anderson	67	Fayette	113	Jessamine	187	Owen
11	Bath	69	Fleming	117	Kenton	191	Pendleton
15	Boone	73	Franklin	137	Lincoln	201	Robertson
17	Bourbon	77	Gallatin	151	Madison	209	Scott
21	Boyle	79	Garrard	161	Mason	211	Shelby
23	Bracken	81	Grant	167	Mercer	215	Spencer
37	Campbell	97	Harrison	173	Montgomery	223	Trimble
41	Carroll	103	Henry	181	Nicholas	229	Washington
49	Clark	111	Jefferson	185	Oldham	239	Woodford

Survey Unit Code: 5 **Survey Unit Name**: Pennyroyal

County code and county name							
1	Adair	57	Cumberland	99	Hart	179	Nelson
27	Breckinridge	85	Grayson	123	Larue	199	Pulaski
29	Bullitt	87	Green	155	Marion	207	Russell
45	Casey	91	Hancock	163	Meade	217	Taylor
53	Clinton	93	Hardin	169	Metcalfe	231	Wayne

Survey Unit Code: 6 **Survey Unit Name**: Western Coalfield

County code and county name							
3	Allen	55	Crittenden	141	Logan	213	Simpson
9	Barren	59	Daviess	149	McLean	219	Todd
31	Butler	61	Edmonson	171	Monroe	225	Union
33	Caldwell	101	Henderson	177	Muhlenberg	227	Warren
47	Christian	107	Hopkins	183	Ohio	233	Webster

Survey Unit Code: 7 **Survey Unit Name**: Western

County code and county name							
7	Ballard	75	Fulton	139	Livingston	157	Marshall
35	Calloway	83	Graves	143	Lyon	221	Trigg
39	Carlisle	105	Hickman	145	McCracken		

State Code: 22 **State Name:** Louisiana **State Abbreviation:** LA **Region/Station Code:** 33

Survey Unit Code: 1 **Survey Unit Name**: North Delta

County code and county name					
25	Catahoula	41	Franklin	83	Richland
29	Concordia	65	Madison	107	Tensas
35	East Carroll	67	Morehouse	123	West Carroll

Survey Unit Code: 2 **Survey Unit Name**: South Delta

County code and county name							
1	Acadia	47	Iberville	77	Pointe Coupee	99	St. Martin
5	Ascension	51	Jefferson	87	St. Bernard	101	St. Mary
7	Assumption	55	Lafayette	89	St. Charles	109	Terrebonne
9	Avoyelles	57	Lafourche	93	St. James	113	Vermilion
23	Cameron	71	Orleans	95	St. John the Baptist	121	West Baton Rouge
45	Iberia	75	Plaquemines	97	St. Landry	125	West Feliciana

Survey Unit Code: 3 **Survey Unit Name**: Southwest

County code and county name							
3	Allen	39	Evangeline	59	La Salle	85	Sabine
11	Beauregard	43	Grant	69	Natchitoches	115	Vernon
19	Calcasieu	53	Jefferson Davis	79	Rapides		

Survey Unit Code: 4 **Survey Unit Name**: Southeast

County code and county name							
33	East Baton Rouge	63	Livingston	103	St. Tammany	117	Washington
37	East Feliciana	91	St. Helena	105	Tangipahoa		

Survey Unit Code: 5 **Survey Unit Name**: Northwest

County code and county name							
13	Bienville	27	Claiborne	73	Ouachita	127	Winn
15	Bossier	31	De Soto	81	Red River		
17	Caddo	49	Jackson	111	Union		
21	Caldwell	61	Lincoln	119	Webster		

State Code: 23 **State Name:** Maine **State Abbreviation:** ME **Region/Station Code:** 24

Survey Unit Code: 1 **Survey Unit Name**: Washington

County code and county name	
29	Washington

Survey Unit Code: 2 **Survey Unit Name**: Aroostook

County code and county name	
3	Aroostook

Survey Unit Code: 3 **Survey Unit Name**: Penobscot

County code and county name	
19	Penobscot

Survey Unit Code: 4 **Survey Unit Name**: Hancock

County code and county name	
9	Hancock

Survey Unit Code: 5 **Survey Unit Name**: Piscataquis

County code and county name	
21	Piscataquis

Survey Unit Code: 6 **Survey Unit Name**: Capitol Region

County code and county name							
11	Kennebec	13	Knox	15	Lincoln	27	Waldo

Survey Unit Code: 7 **Survey Unit Name**: Somerset

County code and county name	
25	Somerset

Survey Unit Code: 8 **Survey Unit Name**: Casco Bay

County code and county name							
1	Androscoggin	5	Cumberland	23	Sagadahoc	31	York

Survey Unit Code: 9 **Survey Unit Name**: Western Maine

County code and county name			
7	Franklin	17	Oxford

State Code: 24 **State Name:** Maryland **State Abbreviation:** MD **Region/Station Code:** 24

Survey Unit Code: 2 **Survey Unit Name**: Central

County code and county name							
3	Anne Arundel	15	Cecil	29	Kent	41	Talbot
5	Baltimore	21	Frederick	31	Montgomery	43	Washington
11	Caroline	25	Harford	33	Prince George's	510	Baltimore city
13	Carroll	27	Howard	35	Queen Anne's		

Survey Unit Code: 3 **Survey Unit Name**: Southern

County code and county name					
09	Calvert	17	Charles	37	St. Mary's

Survey Unit Code: 4 **Survey Unit Name**: Lower Eastern Shore

County code and county name							
19	Dorchester	39	Somerset	45	Wicomico	47	Worcester

Survey Unit Code: 5 **Survey Unit Name**: Western

County code and county name			
1	Allegany	23	Garrett

State Code: 25 **State Name:** Massachusetts **State Abbreviation:** MA **Region/Station Code:** 24

Survey Unit Code: 1 **Survey Unit Name**: Massachusetts

County code and county name							
1	Barnstable	9	Essex	17	Middlesex	25	Suffolk
3	Berkshire	11	Franklin	19	Nantucket	27	Worcester
5	Bristol	13	Hampden	21	Norfolk		
7	Dukes	15	Hampshire	23	Plymouth		

State Code: 26 **State Name:** Michigan **State Abbreviation:** MI **Region/Station Code:** 23

Survey Unit Code: 1 **Survey Unit Name**: Eastern Upper Peninsula

County code and county name							
3	Alger	41	Delta	97	Mackinac	153	Schoolcraft
33	Chippewa	95	Luce	109	Menominee		

Survey Unit Code: 2 **Survey Unit Name**: Western Upper Peninsula

County code and county name							
13	Baraga	53	Gogebic	71	Iron	103	Marquette
43	Dickinson	61	Houghton	83	Keweenaw	131	Ontonagon

Survey Unit Code: 3 **Survey Unit Name**: Northern Lower Peninsula

County code and county name							
1	Alcona	39	Crawford	101	Manistee	133	Osceola
7	Alpena	47	Emmet	105	Mason	135	Oscoda
9	Antrim	51	Gladwin	107	Mecosta	137	Otsego
11	Arenac	55	Grand Traverse	111	Midland	141	Presque Isle
17	Bay	69	Iosco	113	Missaukee	143	Roscommon
19	Benzie	73	Isabella	119	Montmorency	165	Wexford
29	Charlevoix	79	Kalkaska	123	Newaygo		
31	Cheboygan	85	Lake	127	Oceana		
35	Clare	89	Leelanau	129	Ogemaw		

Survey Unit Code: 4 **Survey Unit Name**: Southern Lower Peninsula

County code and county name							
5	Allegan	57	Gratiot	91	Lenawee	147	St. Clair
15	Barry	59	Hillsdale	93	Livingston	149	St. Joseph
21	Berrien	63	Huron	99	Macomb	151	Sanilac
23	Branch	65	Ingham	115	Monroe	155	Shiawassee
25	Calhoun	67	Ionia	117	Montcalm	157	Tuscola
27	Cass	75	Jackson	121	Muskegon	159	Van Buren
37	Clinton	77	Kalamazoo	125	Oakland	161	Washtenaw
45	Eaton	81	Kent	139	Ottawa	163	Wayne
49	Genesee	87	Lapeer	145	Saginaw		

State Code: 27 **State Name:** Minnesota **State Abbreviation:** MN **Region/Station Code:** 23

Survey Unit Code: 1 **Survey Unit Name**: Aspen-Birch

County code and county name					
17	Carlton	71	Koochiching	137	St. Louis
31	Cook	75	Lake		

Survey Unit Code: 2 **Survey Unit Name**: Northern Pine

County code and county name							
1	Aitkin	21	Cass	57	Hubbard	87	Mahnomen
5	Becker	29	Clearwater	61	Itasca	135	Roseau
7	Beltrami	35	Crow Wing	77	Lake of the Woods	159	Wadena

Survey Unit Code: 3 **Survey Unit Name**: Central Hardwood

County code and county name							
3	Anoka	49	Goodhue	97	Morrison	141	Sherburne
9	Benton	53	Hennepin	109	Olmsted	145	Stearns
19	Carver	55	Houston	111	Otter Tail	153	Todd
25	Chisago	59	Isanti	115	Pine	157	Wabasha
37	Dakota	65	Kanabec	123	Ramsey	163	Washington
41	Douglas	79	Le Sueur	131	Rice	169	Winona
45	Fillmore	95	Mille Lacs	139	Scott	171	Wright

Survey Unit Code: 4 **Survey Unit Name**: Prairie

County code and county name							
11	Big Stone	67	Kandiyohi	103	Nicollet	143	Sibley
13	Blue Earth	69	Kittson	105	Nobles	147	Steele
15	Brown	73	Lac qui Parle	107	Norman	149	Stevens
23	Chippewa	81	Lincoln	113	Pennington	151	Swift
27	Clay	83	Lyon	117	Pipestone	155	Traverse
33	Cottonwood	85	McLeod	119	Polk	161	Waseca
39	Dodge	89	Marshall	121	Pope	165	Watonwan
43	Faribault	91	Martin	125	Red Lake	167	Wilkin
47	Freeborn	93	Meeker	127	Redwood	173	Yellow Medicine
51	Grant	99	Mower	129	Renville		
63	Jackson	101	Murray	133	Rock		

State Code: 28 **State Name:** Mississippi **State Abbreviation:** MS **Region/Station Code:** 33

Survey Unit Code: 1 **Survey Unit Name**: Delta

County code and county name							
11	Bolivar	55	Issaquena	133	Sunflower	151	Washington
27	Coahoma	83	Leflore	135	Tallahatchie	163	Yazoo
51	Holmes	119	Quitman	143	Tunica		
53	Humphreys	125	Sharkey	149	Warren		

Survey Unit Code: 2 **Survey Unit Name**: North

County code and county name							
3	Alcorn	33	DeSoto	95	Monroe	139	Tippah
9	Benton	43	Grenada	97	Montgomery	141	Tishomingo
13	Calhoun	57	Itawamba	105	Oktibbeha	145	Union
15	Carroll	71	Lafayette	107	Panola	155	Webster
17	Chickasaw	81	Lee	115	Pontotoc	161	Yalobusha
19	Choctaw	87	Lowndes	117	Prentiss		
25	Clay	93	Marshall	137	Tate		

Survey Unit Code: 3 **Survey Unit Name**: Central

County code and county name							
7	Attala	75	Lauderdale	103	Noxubee	129	Smith
23	Clarke	79	Leake	121	Rankin	159	Winston
61	Jasper	99	Neshoba	123	Scott		
69	Kemper	101	Newton	127	Simpson		

Survey Unit Code: 4 **Survey Unit Name**: South

County code and county name							
31	Covington	47	Harrison	77	Lawrence	147	Walthall
35	Forrest	59	Jackson	91	Marion	153	Wayne
39	George	65	Jefferson Davis	109	Pearl River		
41	Greene	67	Jones	111	Perry		
45	Hancock	73	Lamar	131	Stone		

Survey Unit Code: 5 **Survey Unit Name**: Southwest

County code and county name							
1	Adams	29	Copiah	63	Jefferson	113	Pike
5	Amite	37	Franklin	85	Lincoln	157	Wilkinson
21	Claiborne	49	Hinds	89	Madison		

State Code: 29 **State Name:** Missouri **State Abbreviation:** MO **Region/Station Code:** 23

Survey Unit Code: 1 **Survey Unit Name**: Eastern Ozarks

County code and county name

17	Bollinger	65	Dent	179	Reynolds	221	Washington
23	Butler	93	Iron	181	Ripley	223	Wayne
35	Carter	123	Madison	187	St. Francois		
55	Crawford	149	Oregon	203	Shannon		

Survey Unit Code: 2 **Survey Unit Name**: Southwestern Ozarks

County code and county name

9	Barry	91	Howell	153	Ozark	215	Texas
43	Christian	119	McDonald	209	Stone	225	Webster
67	Douglas	145	Newton	213	Taney	229	Wright

Survey Unit Code: 3 **Survey Unit Name**: Northwestern Ozarks

County code and county name

15	Benton	85	Hickory	141	Morgan	185	St. Clair
29	Camden	105	Laclede	161	Phelps		
39	Cedar	125	Maries	167	Polk		
59	Dallas	131	Miller	169	Pulaski		

Survey Unit Code: 4 **Survey Unit Name**: Prairie

County code and county name

1	Adair	53	Cooper	107	Lafayette	171	Putnam
3	Andrew	57	Dade	109	Lawrence	173	Ralls
5	Atchison	61	Daviess	111	Lewis	175	Randolph
7	Audrain	63	DeKalb	113	Lincoln	177	Ray
11	Barton	75	Gentry	115	Linn	195	Saline
13	Bates	77	Greene	117	Livingston	197	Schuyler
21	Buchanan	79	Grundy	121	Macon	199	Scotland
25	Caldwell	81	Harrison	127	Marion	205	Shelby
33	Carroll	83	Henry	129	Mercer	211	Sullivan
37	Cass	87	Holt	137	Monroe	217	Vernon
41	Chariton	95	Jackson	147	Nodaway	227	Worth
45	Clark	97	Jasper	159	Pettis		
47	Clay	101	Johnson	163	Pike		
49	Clinton	103	Knox	165	Platte		

Survey Unit Code: 5 **Survey Unit Name**: Riverborder

County code and county name

19	Boone	73	Gasconade	143	New Madrid	189	St. Louis
27	Callaway	89	Howard	151	Osage	201	Scott
31	Cape Girardeau	99	Jefferson	155	Pemiscot	207	Stoddard
51	Cole	133	Mississippi	157	Perry	219	Warren
69	Dunklin	135	Moniteau	183	St. Charles	510	St. Louis city
71	Franklin	139	Montgomery	186	Ste. Genevieve		

State Code: 30 **State Name:** Montana **State Abbreviation:** MT **Region/Station Code:** 22

Survey Unit Code: 1 **Survey Unit Name**: Northwestern

County code and county name							
29	Flathead	47	Lake	53	Lincoln	89	Sanders

Survey Unit Code: 2 **Survey Unit Name**: Eastern

County code and county name							
3	Big Horn	27	Fergus	71	Phillips	95	Stillwater
5	Blaine	33	Garfield	73	Pondera	97	Sweet Grass
9	Carbon	35	Glacier	75	Powder River	99	Teton
11	Carter	37	Golden Valley	79	Prairie	101	Toole
15	Chouteau	41	Hill	83	Richland	103	Treasure
17	Custer	51	Liberty	85	Roosevelt	105	Valley
19	Daniels	55	McCone	87	Rosebud	109	Wibaux
21	Dawson	65	Musselshell	91	Sheridan	111	Yellowstone
25	Fallon	69	Petroleum				

Survey Unit Code: 3 **Survey Unit Name**: Western

County code and county name							
39	Granite	61	Mineral	63	Missoula	81	Ravalli

Survey Unit Code: 4 **Survey Unit Name**: West Central

County code and county name							
7	Broadwater	43	Jefferson	49	Lewis and Clark	77	Powell
13	Cascade	45	Judith Basin	59	Meagher	107	Wheatland

Survey Unit Code: 5 **Survey Unit Name**: Southwestern

County code and county name					
1	Beaverhead	31	Gallatin	67	Park
23	Deer Lodge	57	Madison	93	Silver Bow

State Code: 31 **State Name:** Nebraska **State Abbreviation:** NE **Region/Station Code:** 23

Survey Unit Code: 1 **Survey Unit Name**: Eastern

County code and county name							
1	Adams	55	Douglas	99	Kearney	151	Saline
11	Boone	59	Fillmore	109	Lancaster	153	Sarpy
19	Buffalo	61	Franklin	119	Madison	155	Saunders
21	Burt	63	Frontier	121	Merrick	159	Seward
23	Butler	65	Furnas	125	Nance	163	Sherman
25	Cass	67	Gage	127	Nemaha	167	Stanton
27	Cedar	73	Gosper	129	Nuckolls	169	Thayer
35	Clay	77	Greeley	131	Otoe	173	Thurston
37	Colfax	79	Hall	133	Pawnee	175	Valley
39	Cuming	81	Hamilton	137	Phelps	177	Washington
41	Custer	83	Harlan	139	Pierce	179	Wayne
43	Dakota	87	Hitchcock	141	Platte	181	Webster
47	Dawson	93	Howard	143	Polk	185	York
51	Dixon	95	Jefferson	145	Red Willow		
53	Dodge	97	Johnson	147	Richardson		

Survey Unit Code: 2 **Survey Unit Name**: Western

County code and county name							
3	Antelope	33	Cheyenne	91	Hooker	123	Morrill
5	Arthur	45	Dawes	101	Keith	135	Perkins
7	Banner	49	Deuel	103	Keya Paha	149	Rock
9	Blaine	57	Dundy	105	Kimball	157	Scotts Bluff
13	Box Butte	69	Garden	107	Knox	161	Sheridan
15	Boyd	71	Garfield	111	Lincoln	165	Sioux
17	Brown	75	Grant	113	Logan	171	Thomas
29	Chase	85	Hayes	115	Loup	183	Wheeler
31	Cherry	89	Holt	117	McPherson		

State Code: 32 **State Name:** Nevada **State Abbreviation:** NV **Region/Station Code:** 22

Survey Unit Code: 1 **Survey Unit Name**: Nevada

County code and county name							
1	Churchill	11	Eureka	21	Mineral	33	White Pine
3	Clark	13	Humboldt	23	Nye	510	Carson City
5	Douglas	15	Lander	27	Pershing		
7	Elko	17	Lincoln	29	Storey		
9	Esmeralda	19	Lyon	31	Washoe		

State Code: 33 **State Name:** New Hampshire **State Abbreviation:** NH **Region/Station Code:** 24

Survey Unit Code: 2 **Survey Unit Name**: Northern

County code and county name					
3	Carroll	7	Coos	9	Grafton

Survey Unit Code: 3 **Survey Unit Name**: Southern

County code and county name							
1	Belknap	11	Hillsborough	15	Rockingham	19	Sullivan
5	Cheshire	13	Merrimack	17	Strafford		

State Code: 34 **State Name:** New Jersey **State Abbreviation:** NJ **Region/Station Code:** 24

Survey Unit Code: 1 **Survey Unit Name**: New Jersey

County code and county name							
1	Atlantic	13	Essex	25	Monmouth	37	Sussex
3	Bergen	15	Gloucester	27	Morris	39	Union
5	Burlington	17	Hudson	29	Ocean	41	Warren
7	Camden	19	Hunterdon	31	Passaic		
9	Cape May	21	Mercer	33	Salem		
11	Cumberland	23	Middlesex	35	Somerset		

State Code: 35 **State Name:** New Mexico **State Abbreviation:** NM **Region/Station Code:** 22

Survey Unit Code: 1 **Survey Unit Name**: Northwestern

County code and county name							
1	Bernalillo	31	McKinley	45	San Juan	61	Valencia
6	Cibola	39	Rio Arriba	49	Santa Fe		
28	Los Alamos	43	Sandoval	55	Taos		

Survey Unit Code: 2 **Survey Unit Name**: Northeastern

County code and county name							
7	Colfax	21	Harding	37	Quay	57	Torrance
19	Guadalupe	33	Mora	47	San Miguel	59	Union

Survey Unit Code: 3 **Survey Unit Name**: Southwestern

County code and county name							
3	Catron	17	Grant	29	Luna	53	Socorro
13	Dona Ana	23	Hidalgo	51	Sierra		

Survey Unit Code: 4 **Survey Unit Name**: Southeastern

County code and county name							
5	Chaves	11	De Baca	25	Lea	35	Otero
9	Curry	15	Eddy	27	Lincoln	41	Roosevelt

State Code: 36 **State Name:** New York **State Abbreviation:** NY **Region/Station Code:** 24

Survey Unit Code: 1 **Survey Unit Name**: Adirondack

County code and county name							
19	Clinton	33	Franklin	45	Jefferson	89	St. Lawrence

Survey Unit Code: 2 **Survey Unit Name**: Lake Plain

County code and county name							
11	Cayuga	53	Madison	69	Ontario	117	Wayne
29	Erie	55	Monroe	73	Orleans	121	Wyoming
37	Genesee	63	Niagara	75	Oswego	123	Yates
51	Livingston	67	Onondaga	99	Seneca		

Survey Unit Code: 3 **Survey Unit Name**: Western Adirondack

County code and county name							
35	Fulton	43	Herkimer	49	Lewis	65	Oneida

Survey Unit Code: 4 **Survey Unit Name**: Eastern Adirondack

County code and county name					
31	Essex	41	Hamilton	113	Warren

Survey Unit Code: 5 **Survey Unit Name**: Southwest Highlands

County code and county name							
3	Allegany	9	Cattaraugus	13	Chautauqua	101	Steuben

Survey Unit Code: 6 **Survey Unit Name**: South-Central Highlands

County code and county name					
7	Broome	23	Cortland	97	Schuyler
15	Chemung	25	Delaware	107	Tioga
17	Chenango	77	Otsego	109	Tompkins

Survey Unit Code: 7 **Survey Unit Name**: Capitol District

County code and county name							
1	Albany	57	Montgomery	91	Saratoga	115	Washington
21	Columbia	83	Rensselaer	93	Schenectady		

Survey Unit Code: 8 **Survey Unit Name**: Catskill-Lower Hudson

County code and county name							
5	Bronx	59	Nassau	81	Queens	103	Suffolk
27	Dutchess	61	New York	85	Richmond	105	Sullivan
39	Greene	71	Orange	87	Rockland	111	Ulster
47	Kings	79	Putnam	95	Schoharie	119	Westchester

State Code: 37 **State Name:** North Carolina **State Abbreviation:** NC **Region/Station Code:** 33

Survey Unit Code: 1 **Survey Unit Name**: Southern Coastal Plain

County code and county name							
17	Bladen	85	Harnett	125	Moore	163	Sampson
19	Brunswick	93	Hoke	129	New Hanover	165	Scotland
47	Columbus	101	Johnston	133	Onslow	191	Wayne
51	Cumberland	103	Jones	141	Pender		
61	Duplin	105	Lee	153	Richmond		
79	Greene	107	Lenoir	155	Robeson		

Survey Unit Code: 2 **Survey Unit Name**: Northern Coastal Plain

County code and county name							
13	Beaufort	53	Currituck	95	Hyde	143	Perquimans
15	Bertie	55	Dare	117	Martin	147	Pitt
29	Camden	65	Edgecombe	127	Nash	177	Tyrrell
31	Carteret	73	Gates	131	Northampton	187	Washington
41	Chowan	83	Halifax	137	Pamlico	195	Wilson
49	Craven	91	Hertford	139	Pasquotank		

Survey Unit Code: 3 **Survey Unit Name**: Piedmont

County code and county name							
1	Alamance	59	Davie	119	Mecklenburg	167	Stanly
3	Alexander	63	Durham	123	Montgomery	169	Stokes
7	Anson	67	Forsyth	135	Orange	171	Surry
25	Cabarrus	69	Franklin	145	Person	179	Union
33	Caswell	71	Gaston	149	Polk	181	Vance
35	Catawba	77	Granville	151	Randolph	183	Wake
37	Chatham	81	Guilford	157	Rockingham	185	Warren
45	Cleveland	97	Iredell	159	Rowan	197	Yadkin
57	Davidson	109	Lincoln	161	Rutherford		

Survey Unit Code: 4 **Survey Unit Name**: Mountains

County code and county name							
5	Alleghany	39	Cherokee	111	McDowell	189	Watauga
9	Ashe	43	Clay	113	Macon	193	Wilkes
11	Avery	75	Graham	115	Madison	199	Yancey
21	Buncombe	87	Haywood	121	Mitchell		
23	Burke	89	Henderson	173	Swain		
27	Caldwell	99	Jackson	175	Transylvania		

State Code: 38 **State Name:** North Dakota **State Abbreviation:** ND **Region/Station Code:** 23

Survey Unit Code: 1 **Survey Unit Name**: Eastern

County code and county name							
1	Adams	29	Emmons	57	Mercer	85	Sioux
3	Barnes	31	Foster	59	Morton	87	Slope
5	Benson	33	Golden Valley	61	Mountrail	89	Stark
7	Billings	35	Grand Forks	63	Nelson	91	Steele
9	Bottineau	37	Grant	65	Oliver	93	Stutsman
11	Bowman	39	Griggs	67	Pembina	95	Towner
13	Burke	41	Hettinger	69	Pierce	97	Traill
15	Burleigh	43	Kidder	71	Ramsey	99	Walsh
17	Cass	45	LaMoure	73	Ransom	101	Ward
19	Cavalier	47	Logan	75	Renville	103	Wells
21	Dickey	49	McHenry	77	Richland	105	Williams
23	Divide	51	McIntosh	79	Rolette		
25	Dunn	53	McKenzie	81	Sargent		
27	Eddy	55	McLean	83	Sheridan		

State Code: 39 **State Name:** Ohio **State Abbreviation:** OH **Region/Station Code:** 24

Survey Unit Code: 1 **Survey Unit Name**: South-Central

County code and county name							
1	Adams	53	Gallia	87	Lawrence	145	Scioto
15	Brown	71	Highland	131	Pike		
25	Clermont	79	Jackson	141	Ross		

Survey Unit Code: 2 **Survey Unit Name**: Southeastern

County code and county name							
9	Athens	105	Meigs	127	Perry	167	Washington
73	Hocking	115	Morgan	163	Vinton		

Survey Unit Code: 3 **Survey Unit Name**: East-Central

County code and county name							
13	Belmont	59	Guernsey	81	Jefferson	121	Noble
19	Carroll	67	Harrison	111	Monroe	157	Tuscarawas
31	Coshocton	75	Holmes	119	Muskingum		

Survey Unit Code: 4 **Survey Unit Name**: Northeastern

County code and county name							
5	Ashland	55	Geauga	103	Medina	155	Trumbull
7	Ashtabula	77	Huron	133	Portage	169	Wayne
29	Columbiana	85	Lake	139	Richland		
35	Cuyahoga	93	Lorain	151	Stark		
43	Erie	99	Mahoning	153	Summit		

Survey Unit Code: 5 **Survey Unit Name**: Southwestern

County code and county name							
17	Butler	45	Fairfield	61	Hamilton	113	Montgomery
23	Clark	47	Fayette	89	Licking	129	Pickaway
27	Clinton	49	Franklin	97	Madison	135	Preble
37	Darke	57	Greene	109	Miami	165	Warren

Survey Unit Code: 6 **Survey Unit Name**: Northwestern

County code and county name							
3	Allen	63	Hancock	107	Mercer	149	Shelby
11	Auglaize	65	Hardin	117	Morrow	159	Union
21	Champaign	69	Henry	123	Ottawa	161	Van Wert
33	Crawford	83	Knox	125	Paulding	171	Williams
39	Defiance	91	Logan	137	Putnam	173	Wood
41	Delaware	95	Lucas	143	Sandusky	175	Wyandot
51	Fulton	101	Marion	147	Seneca		

State Code: 40 **State Name:** Oklahoma **State Abbreviation:** OK **Region/Station Code:** 33

Survey Unit Code: 1 **Survey Unit Name**: Southeast

County code and county name

5	Atoka	29	Coal	79	Le Flore	127	Pushmataha
13	Bryan	61	Haskell	89	McCurtain		
23	Choctaw	77	Latimer	121	Pittsburg		

Survey Unit Code: 2 **Survey Unit Name**: Northeast

County code and county name

1	Adair	41	Delaware	97	Mayes	115	Ottawa
21	Cherokee	91	McIntosh	101	Muskogee	135	Sequoyah

Survey Unit Code: 3 **Survey Unit Name**: Northcentral

County code and county name

35	Craig	113	Osage	131	Rogers	145	Wagoner
37	Creek	117	Pawnee	143	Tulsa	147	Washington
105	Nowata	119	Payne				

Survey Unit Code: 4 **Survey Unit Name**: Southcentral

County code and county name

19	Carter	81	Lincoln	95	Marshall	111	Okmulgee
27	Cleveland	83	Logan	99	Murray	123	Pontotoc
49	Garvin	85	Love	107	Okfuskee	125	Pottawatomie
63	Hughes	87	McClain	109	Oklahoma	133	Seminole
69	Johnston						

Survey Unit Code: 5 **Survey Unit Name**: Southwest

County code and county name

9	Beckham	33	Cotton	57	Harmon	129	Roger Mills
11	Blaine	39	Custer	65	Jackson	137	Stephens
15	Caddo	43	Dewey	67	Jefferson	141	Tillman
17	Canadian	51	Grady	73	Kingfisher	149	Washita
31	Comanche	55	Greer	75	Kiowa		

Survey Unit Code: 6 **Survey Unit Name**: High Plains

County code and county name

7	Beaver	45	Ellis	59	Harper	139	Texas
25	Cimarron						

Survey Unit Code: 7 **Survey Unit Name**: Great Plains

County code and county name

3	Alfalfa	53	Grant	93	Major	151	Woods
47	Garfield	71	Kay	103	Noble	153	Woodward

State Code: 41 **State Name:** Oregon **State Abbreviation:** OR **Region/Station Code:** 26

Survey Unit Code: 0 **Survey Unit Name**: Northwest

County code and county name							
5	Clackamas	27	Hood River	53	Polk	71	Yamhill
7	Clatsop	47	Marion	57	Tillamook		
9	Columbia	51	Multnomah	67	Washington		

Survey Unit Code: 1 **Survey Unit Name**: West Central

County code and county name							
3	Benton	39	Lane	41	Lincoln	43	Linn

Survey Unit Code: 2 **Survey Unit Name**: Southwest

County code and county name					
11	Coos	19	Douglas	33	Josephine
15	Curry	29	Jackson		

Survey Unit Code: 3 **Survey Unit Name**: Central

County code and county name					
13	Crook	31	Jefferson	55	Sherman
17	Deschutes	35	Klamath	65	Wasco
21	Gilliam	37	Lake	69	Wheeler

Survey Unit Code: 4 **Survey Unit Name**: Blue Mountains

County code and county name							
1	Baker	25	Harney	49	Morrow	61	Union
23	Grant	45	Malheur	59	Umatilla	63	Wallowa

State Code: 42 **State Name:** Pennsylvania **State Abbreviation:** PA **Region/Station Code:** 24

Survey Unit Code: 0 **Survey Unit Name**: South Central

County code and county name							
43	Dauphin	61	Huntingdon	99	Perry		
55	Franklin	67	Juniata	109	Snyder		
57	Fulton	87	Mifflin	119	Union		

Survey Unit Code: 5 **Survey Unit Name**: Western

County code and county name							
3	Allegheny	19	Butler	59	Greene	85	Mercer
5	Armstrong	39	Crawford	63	Indiana	125	Washington
7	Beaver	49	Erie	73	Lawrence	129	Westmoreland

Survey Unit Code: 6 **Survey Unit Name**: North Central/Allegheny

County code and county name							
23	Cameron	35	Clinton	81	Lycoming	117	Tioga
27	Centre	47	Elk	83	McKean	121	Venango
31	Clarion	53	Forest	105	Potter	123	Warren
33	Clearfield	65	Jefferson	113	Sullivan		

Survey Unit Code: 7 **Survey Unit Name**: Southwestern

County code and county name							
9	Bedford	21	Cambria	111	Somerset		
13	Blair	51	Fayette				

Survey Unit Code: 8 **Survey Unit Name**: Northeastern/Pocono

County code and county name							
15	Bradford	79	Luzerne	103	Pike	131	Wyoming
25	Carbon	89	Monroe	107	Schuylkill		
37	Columbia	93	Montour	115	Susquehanna		
69	Lackawanna	97	Northumberland	127	Wayne		

Survey Unit Code: 9 **Survey Unit Name**: Southeastern

County code and county name							
1	Adams	41	Cumberland	77	Lehigh	133	York
11	Berks	45	Delaware	91	Montgomery		
17	Bucks	71	Lancaster	95	Northampton		
29	Chester	75	Lebanon	101	Philadelphia		

State Code: 44 **State Name:** Rhode Island **State Abbreviation:** RI **Region/Station Code:** 24

Survey Unit Code: 1 **Survey Unit Name**: Rhode Island

County code and county name					
1	Bristol	5	Newport	9	Washington
3	Kent	7	Providence		

State Code: 45 **State Name:** South Carolina **State Abbreviation:** SC **Region/Station Code:** 33

Survey Unit Code: 1 **Survey Unit Name**: Southern Coastal Plain

County code and county name							
3	Aiken	11	Barnwell	29	Colleton	53	Jasper
5	Allendale	13	Beaufort	35	Dorchester	63	Lexington
9	Bamberg	17	Calhoun	49	Hampton	75	Orangeburg

Survey Unit Code: 2 **Survey Unit Name**: Northern Coastal Plain

County code and county name							
15	Berkeley	31	Darlington	51	Horry	69	Marlboro
19	Charleston	33	Dillon	55	Kershaw	79	Richland
25	Chesterfield	41	Florence	61	Lee	85	Sumter
27	Clarendon	43	Georgetown	67	Marion	89	Williamsburg

Survey Unit Code: 3 **Survey Unit Name**: Piedmont

County code and county name							
1	Abbeville	39	Fairfield	65	McCormick	83	Spartanburg
7	Anderson	45	Greenville	71	Newberry	87	Union
21	Cherokee	47	Greenwood	73	Oconee	91	York
23	Chester	57	Lancaster	77	Pickens		
37	Edgefield	59	Laurens	81	Saluda		

State Code: 46 **State Name:** South Dakota **State Abbreviation:** SD **Region/Station Code:** 23

Survey Unit Code: 1 **Survey Unit Name**: Eastern

County code and county name							
3	Aurora	37	Day	71	Jackson	107	Potter
5	Beadle	39	Deuel	73	Jerauld	109	Roberts
7	Bennett	41	Dewey	75	Jones	111	Sanborn
9	Bon Homme	43	Douglas	77	Kingsbury	115	Spink
11	Brookings	45	Edmunds	79	Lake	117	Stanley
13	Brown	49	Faulk	83	Lincoln	119	Sully
15	Brule	51	Grant	85	Lyman	121	Todd
17	Buffalo	53	Gregory	87	McCook	123	Tripp
21	Campbell	55	Haakon	89	McPherson	125	Turner
23	Charles Mix	57	Hamlin	91	Marshall	127	Union
25	Clark	59	Hand	95	Mellette	129	Walworth
27	Clay	61	Hanson	97	Miner	135	Yankton
29	Codington	65	Hughes	99	Minnehaha	137	Ziebach
31	Corson	67	Hutchinson	101	Moody		
35	Davison	69	Hyde	105	Perkins		

Survey Unit Code: 2 **Survey Unit Name**: Western

County code and county name							
19	Butte	47	Fall River	81	Lawrence	103	Pennington
33	Custer	63	Harding	93	Meade	113	Shannon

State Code: 47 **State Name:** Tennessee **State Abbreviation:** TN **Region/Station Code:** 33

Survey Unit Code: 1 **Survey Unit Name**: West

County code and county name							
17	Carroll	53	Gibson	95	Lake	157	Shelby
23	Chester	69	Hardeman	97	Lauderdale	167	Tipton
33	Crockett	75	Haywood	109	McNairy	183	Weakley
45	Dyer	77	Henderson	113	Madison		
47	Fayette	79	Henry	131	Obion		

Survey Unit Code: 2 **Survey Unit Name**: West Central

County code and county name							
5	Benton	81	Hickman	99	Lawrence	161	Stewart
39	Decatur	83	Houston	101	Lewis	181	Wayne
71	Hardin	85	Humphreys	135	Perry		

Survey Unit Code: 3 **Survey Unit Name**: Central

County code and county name							
3	Bedford	41	DeKalb	117	Marshall	159	Smith
15	Cannon	43	Dickson	119	Maury	165	Sumner
21	Cheatham	55	Giles	125	Montgomery	169	Trousdale
27	Clay	87	Jackson	127	Moore	187	Williamson
31	Coffee	103	Lincoln	147	Robertson	189	Wilson
37	Davidson	111	Macon	149	Rutherford		

Survey Unit Code: 4 **Survey Unit Name**: Plateau

County code and county name							
7	Bledsoe	51	Franklin	133	Overton	153	Sequatchie
13	Campbell	61	Grundy	137	Pickett	175	Van Buren
35	Cumberland	115	Marion	141	Putnam	177	Warren
49	Fentress	129	Morgan	151	Scott	185	White

Survey Unit Code: 5 **Survey Unit Name**: East

County code and county name							
1	Anderson	59	Greene	93	Knox	145	Roane
9	Blount	63	Hamblen	105	Loudon	155	Sevier
11	Bradley	65	Hamilton	107	McMinn	163	Sullivan
19	Carter	67	Hancock	121	Meigs	171	Unicoi
25	Claiborne	73	Hawkins	123	Monroe	173	Union
29	Cocke	89	Jefferson	139	Polk	179	Washington
57	Grainger	91	Johnson	143	Rhea		

State Code: 48 **State Name:** Texas **State Abbreviation:** TX **Region/Station Code:** 33

Survey Unit Code: 1 **Survey Unit Name**: Southeast

County code and county name							
5	Angelina	241	Jasper	351	Newton	455	Trinity
71	Chambers	245	Jefferson	361	Orange	457	Tyler
185	Grimes	289	Leon	373	Polk	471	Walker
199	Hardin	291	Liberty	403	Sabine	473	Waller
201	Harris	313	Madison	405	San Augustine		
225	Houston	339	Montgomery	407	San Jacinto		

Survey Unit Code: 2 **Survey Unit Name**: Northeast

County code and county name							
1	Anderson	183	Gregg	365	Panola	459	Upshur
37	Bowie	203	Harrison	387	Red River	467	Van Zandt
63	Camp	213	Henderson	401	Rusk	499	Wood
67	Cass	315	Marion	419	Shelby		
73	Cherokee	343	Morris	423	Smith		
159	Franklin	347	Nacogdoches	449	Titus		

Survey Unit Code: 3 **Survey Unit Name**: Northcentral

County code and county name							
15	Austin	121	Denton	217	Hill	337	Montague
21	Bastrop	123	De Witt	223	Hopkins	349	Navarro
41	Brazos	139	Ellis	231	Hunt	367	Parker
51	Burleson	145	Falls	237	Jack	379	Rains
55	Caldwell	147	Fannin	251	Johnson	395	Robertson
77	Clay	149	Fayette	257	Kaufman	397	Rockwall
85	Collin	161	Freestone	277	Lamar	439	Tarrant
89	Colorado	175	Goliad	285	Lavaca	477	Washington
97	Cooke	177	Gonzales	287	Lee	497	Wise
113	Dallas	181	Grayson	293	Limestone	503	Young
119	Delta	187	Guadalupe	331	Milam		

Survey Unit Code: 4 **Survey Unit Name**: South

County code and county name							
7	Aransas	157	Fort Bend	273	Kleberg	427	Starr
13	Atascosa	163	Frio	283	La Salle	469	Victoria
25	Bee	167	Galveston	297	Live Oak	479	Webb
39	Brazoria	215	Hidalgo	311	McMullen	481	Wharton
47	Brooks	239	Jackson	321	Matagorda	489	Willacy
57	Calhoun	247	Jim Hogg	323	Maverick	493	Wilson
61	Cameron	249	Jim Wells	355	Nueces	505	Zapata
127	Dimmit	255	Karnes	391	Refugio	507	Zavala
131	Duval	261	Kenedy	409	San Patricio		

Texas cont.

Texas cont.

Survey Unit Code: 5 **Survey Unit Name**: Westcentral

County code and county name							
19	Bandera	99	Coryell	267	Kimble	385	Real
27	Bell	105	Crockett	271	Kinney	399	Runnels
29	Bexar	133	Eastland	281	Lampasas	411	San Saba
31	Blanco	137	Edwards	299	Llano	413	Schleicher
35	Bosque	143	Erath	307	McCulloch	425	Somervell
49	Brown	171	Gillespie	309	McLennan	429	Stephens
53	Burnet	193	Hamilton	319	Mason	435	Sutton
59	Callahan	209	Hays	325	Medina	453	Travis
83	Coleman	221	Hood	327	Menard	463	Uvalde
91	Comal	259	Kendall	333	Mills	465	Val Verde
93	Comanche	265	Kerr	363	Palo Pinto	491	Williamson
95	Concho						

Survey Unit Code: 6 **Survey Unit Name**: Northwest

County code and county name							
3	Andrews	129	Donley	235	Irion	375	Potter
9	Archer	151	Fisher	253	Jones	381	Randall
11	Armstrong	153	Floyd	263	Kent	383	Reagan
17	Bailey	155	Foard	269	King	393	Roberts
23	Baylor	165	Gaines	275	Knox	415	Scurry
33	Borden	169	Garza	279	Lamb	417	Shackelford
45	Briscoe	173	Glasscock	295	Lipscomb	421	Sherman
65	Carson	179	Gray	303	Lubbock	431	Sterling
69	Castro	189	Hale	305	Lynn	433	Stonewall
75	Childress	191	Hall	317	Martin	437	Swisher
79	Cochran	195	Hansford	329	Midland	441	Taylor
81	Coke	197	Hardeman	335	Mitchell	445	Terry
87	Collingsworth	205	Hartley	341	Moore	447	Throckmorton
101	Cottle	207	Haskell	345	Motley	451	Tom Green
107	Crosby	211	Hemphill	353	Nolan	483	Wheeler
111	Dallam	219	Hockley	357	Ochiltree	485	Wichita
115	Dawson	227	Howard	359	Oldham	487	Wilbarger
117	Deaf Smith	233	Hutchinson	369	Parmer	501	Yoakum
125	Dickens						

Survey Unit Code: 7 **Survey Unit Name**: West

County code and county name							
43	Brewster	141	El Paso	371	Pecos	461	Upton
103	Crane	229	Hudsbeth	377	Presidio	475	Ward
109	Culberson	243	Jeff Davis	389	Reeves	495	Winkler
135	Ector	301	Loving	443	Terrell		

State Code: 49 **State Name:** Utah **State Abbreviation:** UT **Region/Station Code:** 22

Survey Unit Code: 1 **Survey Unit Name**: Northern

County code and county name							
3	Box Elder	29	Morgan	43	Summit	51	Wasatch
5	Cache	33	Rich	45	Tooele	57	Weber
11	Davis	35	Salt Lake	49	Utah		

Survey Unit Code: 2 **Survey Unit Name**: Uinta

County code and county name					
9	Daggett	13	Duchesne	47	Uintah

Survey Unit Code: 3 **Survey Unit Name**: Central

County code and county name					
23	Juab	31	Piute	41	Sevier
27	Millard	39	Sanpete	55	Wayne

Survey Unit Code: 4 **Survey Unit Name**: Eastern

County code and county name							
7	Carbon	15	Emery	19	Grand	37	San Juan

Survey Unit Code: 5 **Survey Unit Name**: Southwestern

County code and county name					
1	Beaver	21	Iron	53	Washington
17	Garfield	25	Kane		

State Code: 50 **State Name:** Vermont **State Abbreviation:** VT **Region/Station Code:** 24

Survey Unit Code: 2 **Survey Unit Name**: Northern

County code and county name							
5	Caledonia	11	Franklin	15	Lamoille	19	Orleans
9	Essex	13	Grand Isle	17	Orange	23	Washington

Survey Unit Code: 3 **Survey Unit Name**: Southern

County code and county name					
1	Addison	7	Chittenden	25	Windham
3	Bennington	21	Rutland	27	Windsor

State Code: 51 **State Name:** Virginia **State Abbreviation:** VA **Region/Station Code:** 33

Survey Unit Code: 1 **Survey Unit Name**: Coastal Plain

County code and county name							
1	Accomack	85	Hanover	119	Middlesex	193	Westmoreland
25	Brunswick	87	Henrico	127	New Kent	199	York
33	Caroline	93	Isle Of Wight	131	Northampton	550	Chesapeake city
36	Charles City	95	James City	133	Northumberland	650	Hampton city
41	Chesterfield	97	King And Queen	149	Prince George	700	Newport News city
53	Dinwiddie	99	King George	159	Richmond	800	Suffolk city
57	Essex	101	King William	175	Southampton	810	Virginia Beach city
73	Gloucester	103	Lancaster	181	Surry		
81	Greensville	115	Mathews	183	Sussex		

Survey Unit Code: 2 **Survey Unit Name**: Southern Piedmont

County code and county name							
7	Amelia	37	Charlotte	111	Lunenburg	145	Powhatan
11	Appomattox	49	Cumberland	117	Mecklenburg	147	Prince Edward
19	Bedford	67	Franklin	135	Nottoway		
29	Buckingham	83	Halifax	141	Patrick		
31	Campbell	89	Henry	143	Pittsylvania		

Survey Unit Code: 3 **Survey Unit Name**: Northern Piedmont

County code and county name							
3	Albemarle	61	Fauquier	109	Louisa	157	Rappahannock
9	Amherst	65	Fluvanna	113	Madison	177	Spotsylvania
13	Arlington	75	Goochland	125	Nelson	179	Stafford
47	Culpeper	79	Greene	137	Orange		
59	Fairfax	107	Loudoun	153	Prince William		

Survey Unit Code: 4 **Survey Unit Name**: Northern Mountains

County code and county name							
5	Alleghany	43	Clarke	139	Page	171	Shenandoah
15	Augusta	45	Craig	161	Roanoke	187	Warren
17	Bath	69	Frederick	163	Rockbridge		
23	Botetourt	91	Highland	165	Rockingham		

Survey Unit Code: 5 **Survey Unit Name**: Southern Mountains

County code and county name							
21	Bland	71	Giles	167	Russell	195	Wise
27	Buchanan	77	Grayson	169	Scott	197	Wythe
35	Carroll	105	Lee	173	Smyth		
51	Dickenson	121	Montgomery	185	Tazewell		
63	Floyd	155	Pulaski	191	Washington		

Virginia cont.

Virginia cont.

Cities aggregated into other counties

City code and city name		Associated county code and county name		City code and city name		Associated county code and county name	
510	Alexandria city	59	Fairfax	683	Manassas city	153	Prince William
515	Bedford city	19	Bedford	685	Manassas Park city	153	Prince William
520	Bristol city	191	Washington	690	Martinsville city	89	Henry
530	Buena Vista city	163	Rockbridge	710	Norfolk city	550	Chesapeake City
540	Charlottesville city	3	Albemarle	720	Norton city	195	Wise
560	Clifton Forge city	5	Allegheny	730	Petersburg city	53	Dinwiddie
570	Colonial Heights city	41	Chesterfield	730	Petersburg city	149	Prince George
580	Covington city	5	Allegheny	735	Poquoson city	199	York
590	Danville city	143	Pittsylvania	740	Portsmouth city	550	Chesapeake City
595	Emporia city	81	Greensville	750	Radford city	121	Montgomery
600	Fairfax city	59	Fairfax	760	Richmond city	41	Chesterfield
610	Falls Church city	59	Fairfax	760	Richmond city	87	Henrico
620	Franklin city	175	Southampton	770	Roanoke city	161	Roanoke
630	Fredericksburg city	177	Spotsylvania	775	Salem city	161	Roanoke
640	Galax city	35	Carroll	780	South Boston city	83	Halifax
640	Galax city	77	Grayson	790	Staunton city	15	Augusta
660	Harrisonburg city	165	Rockingham	820	Waynesboro city	15	Augusta
670	Hopewell city	149	Prince George	830	Williamsburg city	95	County of James City
678	Lexington city	163	Rockbridge	840	Winchester city	69	Frederick
680	Lynchburg city	31	Campbell				

State Code: 53 **State Name:** Washington **State Abbreviation:** WA **Region/Station Code:** 26

Survey Unit Code: 5 **Survey Unit Name**: Puget Sound

County code and county name							
29	Island	35	Kitsap	55	San Juan	61	Snohomish
33	King	53	Pierce	57	Skagit	73	Whatcom

Survey Unit Code: 6 **Survey Unit Name**: Olympic Peninsula

County code and county name					
9	Clallam	31	Jefferson	67	Thurston
27	Grays Harbor	45	Mason		

Survey Unit Code: 7 **Survey Unit Name**: Southwest

County code and county name					
11	Clark	41	Lewis	59	Skamania
15	Cowlitz	49	Pacific	69	Wahkiakum

Survey Unit Code: 8 **Survey Unit Name**: Central

County code and county name					
7	Chelan	37	Kittitas	47	Okanogan
17	Douglas	39	Klickitat	77	Yakima

Survey Unit Code: 9 **Survey Unit Name**: Inland Empire

County code and county name							
1	Adams	19	Ferry	43	Lincoln	71	Walla Walla
3	Asotin	21	Franklin	51	Pend Oreille	75	Whitman
5	Benton	23	Garfield	63	Spokane		
13	Columbia	25	Grant	65	Stevens		

State Code: 54 **State Name:** West Virginia **State Abbreviation:** WV **Region/Station Code:** 24

Survey Unit Code: 2 **Survey Unit Name**: Northeastern

County code and county name							
1	Barbour	31	Hardy	65	Morgan	91	Taylor
3	Berkeley	33	Harrison	71	Pendleton	93	Tucker
7	Braxton	37	Jefferson	75	Pocahontas	97	Upshur
23	Grant	41	Lewis	77	Preston	101	Webster
27	Hampshire	57	Mineral	83	Randolph		

Survey Unit Code: 3 **Survey Unit Name**: Southern

County code and county name							
5	Boone	39	Kanawha	59	Mingo	89	Summers
15	Clay	45	Logan	63	Monroe	109	Wyoming
19	Fayette	47	McDowell	67	Nicholas		
25	Greenbrier	55	Mercer	81	Raleigh		

Survey Unit Code: 4 **Survey Unit Name**: Northwestern

County code and county name							
9	Brooke	35	Jackson	69	Ohio	99	Wayne
11	Cabell	43	Lincoln	73	Pleasants	103	Wetzel
13	Calhoun	49	Marion	79	Putnam	105	Wirt
17	Doddridge	51	Marshall	85	Ritchie	107	Wood
21	Gilmer	53	Mason	87	Roane		
29	Hancock	61	Monongalia	95	Tyler		

State Code: 55 **State Name:** Wisconsin **State Abbreviation:** WI **Region/Station Code:** 23

Survey Unit Code: 1 **Survey Unit Name**: Northeastern

County code and county name							
37	Florence	69	Lincoln	83	Oconto	125	Vilas
41	Forest	75	Marinette	85	Oneida		
67	Langlade	78	Menominee	115	Shawano		

Survey Unit Code: 2 **Survey Unit Name**: Northwestern

County code and county name							
3	Ashland	13	Burnett	95	Polk	113	Sawyer
5	Barron	31	Douglas	99	Price	119	Taylor
7	Bayfield	51	Iron	107	Rusk	129	Washburn

Survey Unit Code: 3 **Survey Unit Name**: Central

County code and county name							
1	Adams	53	Jackson	81	Monroe	141	Wood
17	Chippewa	57	Juneau	97	Portage		
19	Clark	73	Marathon	135	Waupaca		
35	Eau Claire	77	Marquette	137	Waushara		

Survey Unit Code: 4 **Survey Unit Name**: Southwestern

County code and county name							
11	Buffalo	49	Iowa	93	Pierce	121	Trempealeau
23	Crawford	63	La Crosse	103	Richland	123	Vernon
33	Dunn	65	Lafayette	109	St. Croix		
43	Grant	91	Pepin	111	Sauk		

Survey Unit Code: 5 **Survey Unit Name**: Southeastern

County code and county name							
9	Brown	39	Fond du Lac	71	Manitowoc	117	Sheboygan
15	Calumet	45	Green	79	Milwaukee	127	Walworth
21	Columbia	47	Green Lake	87	Outagamie	131	Washington
25	Dane	55	Jefferson	89	Ozaukee	133	Waukesha
27	Dodge	59	Kenosha	101	Racine	139	Winnebago
29	Door	61	Kewaunee	105	Rock		

State Code: 56 **State Name:** Wyoming **State Abbreviation:** WY **Region/Station Code:** 22

Survey Unit Code: 1 **Survey Unit Name**: Western

County code and county name							
13	Fremont	23	Lincoln	35	Sublette	39	Teton
17	Hot Springs	29	Park	37	Sweetwater	41	Uinta

Survey Unit Code: 2 **Survey Unit Name**: Central and Southeastern

County code and county name							
1	Albany	9	Converse	21	Laramie	31	Platte
3	Big Horn	15	Goshen	25	Natrona	33	Sheridan
7	Carbon	19	Johnson	27	Niobrara	43	Washakie

Survey Unit Code: 3 **Survey Unit Name**: Northeastern

County code and county name					
5	Campbell	11	Crook	45	Weston

State Code: 72 **State Name:** Puerto Rico **State Abbreviation:** PR **Region/Station Code:** 33

Survey Unit Code: 1 **Survey Unit Name**: Puerto Rico

County code and county name							
1	Adjuntas	41	Cidra	79	Lajas	119	Rio Grande
3	Aguada	43	Coamo	81	Lares	121	Sabana Grande
5	Aguadilla	45	Comerio	83	Las Marias	123	Salinas
7	Aguas Buenas	47	Corozal	85	Las Piedras	125	San German
9	Aibonito	49	Culebra	87	Loiza	127	San Juan
11	Anasco	51	Dorado	89	Luquillo	129	San Lorenzo
13	Arecibo	53	Fajardo	91	Manati	131	San Sebastian
15	Arroyo	54	Florida	93	Maricao	133	Santa Isabel
17	Barceloneta	55	Guanica	95	Maunabo	135	Toa Alta
19	Barranquitas	57	Guayama	97	Mayaguez	137	Toa Baja
21	Bayamon	59	Guayanilla	99	Moca	139	Trujillo Alto
23	Cabo Rojo	61	Guaynabo	101	Morovis	141	Utuado
25	Caguas	63	Gurabo	103	Naguabo	143	Vega Alta
27	Camuy	65	Hatillo	105	Naranjito	145	Vega Baja
29	Canovanas	67	Hormigueros	107	Orocovis	147	Vieques
31	Carolina	69	Humacao	109	Patillas	149	Villalba
33	Catano	71	Isabela Municipio	111	Penuelas	151	Yabucoa
35	Cayey	73	Jayuya	113	Ponce	153	Yauco
37	Ceiba	75	Juana Diaz	115	Quebradillas		
39	Ciales	77	Juncos	117	Rincon		

State Code: 78 **State Name:** U.S. Virgin Islands **State Abbreviation:** VI **Region/Station Code:** 33

Survey Unit Code: 1 **Survey Unit Name**: Virgin Islands

County code and county name					
10	St. Croix Island	20	St. John Island	30	St. Thomas Island

Appendix D. Forest Type Codes and Names

Note: The forest type names used by FIA do not come from a single published reference. The current list of forest type names has been developed over time using sources such as historical FIA lists, lists from the Society of American Foresters, and FIA analysts who developed names to meet current analysis and reporting needs.

Code	Forest type / type group
**100**	**White / red / jack pine group**
101	Jack pine
102	Red pine
103	Eastern white pine
104	Eastern white pine / eastern hemlock
105	Eastern hemlock
**120**	**Spruce / fir group**
121	Balsam fir
122	White spruce
123	Red spruce
124	Red spruce / balsam fir
125	Black spruce
126	Tamarack
127	Northern white-cedar
128	Fraser fir
129	Red spruce / Fraser fir
**140**	**Longleaf / slash pine group**
141	Longleaf pine
142	Slash pine
**150**	**Tropical pine group**
151	Tropical pines
**160**	**Loblolly / shortleaf pine group**
161	Loblolly pine
162	Shortleaf pine
163	Virginia pine
164	Sand pine
165	Table mountain pine
166	Pond pine
167	Pitch pine
168	Spruce pine
**170**	**Other eastern softwoods group**
171	Eastern redcedar
172	Florida softwoods
**180**	**Pinyon / juniper group**
182	Rocky Mountain juniper
184	Juniper woodland
185	Pinyon / juniper woodland
**200**	**Douglas-fir group**
201	Douglas-fir
202	Port-Orford-cedar
203	Bigcone Douglas-fir

Code	Forest type / type group
**220**	**Ponderosa pine group**
221	Ponderosa pine
222	Incense-cedar
224	Sugar pine
225	Jeffrey pine
226	Coulter pine
**240**	**Western white pine group**
241	Western white pine
**260**	**Fir / spruce / mountain hemlock group**
261	White fir
262	Red fir
263	Noble fir
264	Pacific silver fir
265	Engelmann spruce
266	Engelmann spruce / subalpine fir
267	Grand fir
268	Subalpine fir
269	Blue spruce
270	Mountain hemlock
271	Alaska-yellow-cedar
**280**	**Lodgepole pine group**
281	Lodgepole pine
**300**	**Hemlock / Sitka spruce group**
301	Western hemlock
304	Western redcedar
305	Sitka spruce
**320**	**Western larch group**
321	Western larch
**340**	**Redwood group**
341	Redwood
342	Giant sequoia
**360**	**Other western softwoods group**
361	Knobcone pine
362	Southwestern white pine
363	Bishop pine
364	Monterey pine
365	Foxtail pine / bristlecone pine
366	Limber pine
367	Whitebark pine
368	Miscellaneous western softwoods
369	Western juniper
**370**	**California mixed conifer group**
371	California mixed conifer
**380**	**Exotic softwoods group**
381	Scotch pine
383	Other exotic softwoods
384	Norway spruce
385	Introduced larch

Code	Forest type / type group
**390**	**Other softwoods group**
391	Other softwoods
**400**	**Oak / pine group**
401	Eastern white pine / northern red oak / white ash
402	Eastern redcedar / hardwood
403	Longleaf pine / oak
404	Shortleaf pine / oak
405	Virginia pine / southern red oak
406	Loblolly pine / hardwood
407	Slash pine / hardwood
409	Other pine / hardwood
**500**	**Oak / hickory group**
501	Post oak / blackjack oak
502	Chestnut oak
503	White oak / red oak / hickory
504	White oak
505	Northern red oak
506	Yellow-poplar / white oak / northern red oak
507	Sassafras / persimmon
508	Sweetgum / yellow-poplar
509	Bur oak
510	Scarlet oak
511	Yellow-poplar
512	Black walnut
513	Black locust
514	Southern scrub oak
515	Chestnut oak / black oak / scarlet oak
516	Cherry / white ash / yellow-poplar
517	Elm / ash / black locust
519	Red maple / oak
520	Mixed upland hardwoods
**600**	**Oak / gum / cypress group**
601	Swamp chestnut oak / cherrybark oak
602	Sweetgum / Nuttall oak / willow oak
605	Overcup oak / water hickory
606	Atlantic white-cedar
607	Baldcypress / water tupelo
608	Sweetbay / swamp tupelo / red maple
609	Baldcypress / pondcypress
**700**	**Elm / ash / cottonwood group**
701	Black ash / American elm / red maple
702	River birch / sycamore
703	Cottonwood
704	Willow
705	Sycamore / pecan / American elm
706	Sugarberry / hackberry / elm / green ash
707	Silver maple / American elm
708	Red maple / lowland
709	Cottonwood / willow
722	Oregon ash
**800**	**Maple / beech / birch group**
801	Sugar maple / beech / yellow birch

Code	Forest type / type group
802	Black cherry
805	Hard maple / basswood
809	Red maple / upland
**900**	**Aspen / birch group**
901	Aspen
902	Paper birch
903	Gray birch
904	Balsam poplar
905	Pin cherry
**910**	**Alder / maple group**
911	Red alder
912	Bigleaf maple
**920**	**Western oak group**
921	Gray pine
922	California black oak
923	Oregon white oak
924	Blue oak
931	Coast live oak
933	Canyon live oak
934	Interior live oak
935	California white oak (valley oak)
	`
**940**	**Tanoak / laurel group**
941	Tanoak
942	California laurel
943	Giant chinkapin
**960**	**Other hardwoods group**
961	Pacific madrone
962	Other hardwoods
**970**	**Woodland hardwoods group**
971	Deciduous oak woodland
972	Evergreen oak woodland
973	Mesquite woodland
974	Cercocarpus (mountain brush) woodland
975	Intermountain maple woodland
976	Miscellaneous woodland hardwoods
**980**	**Tropical hardwoods group**
982	Mangrove
983	Palms
989	Other tropical hardwoods
**990**	**Exotic hardwoods group**
991	Paulownia
992	Melaleuca
993	Eucalyptus
995	Other exotic hardwoods
999	Nonstocked

Appendix E. Administrative National Forest Codes and Names

Region	Code	National Forest/Grassland/Area
**Region 1**	102	Beaverhead
	102	Beaverhead-Deerlodge [now combined]
	103	Bitterroot
	104	Idaho Panhandle
	105	Clearwater
	108	Custer
	109	Deerlodge
	110	Flathead
	111	Gallatin
	112	Helena
	114	Kootenai
	115	Lewis and Clark
	116	Lolo
	117	Nez Perce
	120	Cedar River NGL (National Grassland)
	121	Little Missouri NGL
	122	Sheyenne NGL
	124	Grand River NGL
	199	Other NFS Areas
**Region 2**	202	Bighorn
	203	Black Hills
	204	Grand Mesa-Uncompahgre-Gunnison
	206	Medicine Bow
	206	Medicine Bow-Routt [now combined]
	207	Nebraska
	209	Rio Grande
	210	Arapaho-Roosevelt
	211	Routt
	212	Pike and San Isabel
	213	San Juan
	214	Shoshone
	215	White River
	216	Samuel R Mckelvie
	217	Cimarron NGL
	218	Commanche NGL
	219	Pawnee NGL
	220	Oglala NGL
	221	Buffalo Gap NGL
	222	Fort Pierre NGL
	223	Thunder Basin NGL
	299	Other NFS Areas
**Region 3**	301	Apache-Sitgreaves
	302	Carson
	303	Cibola
	304	Coconino
	305	Coronado
	306	Gila
	307	Kaibab
	308	Lincoln
	309	Prescott
	310	Santa Fe
	312	Tonto
	399	Other NFS Areas
**Region 4**	401	Ashley
	402	Boise
	403	Bridger-Teton

Region	Code	National Forest/Grassland/Area
	405	Caribou
	406	Challis
	407	Dixie
	408	Fishlake
	409	Humboldt
	410	Manti-La Sal
	412	Payette
	413	Salmon
	413	Salmon-Challis [now combined]
	414	Sawtooth
	415	Targhee
	415	Caribou-Targhee [now combined]
	417	Toiyabe
	417	Humboldt-Toiyabe [now combined]
	418	Uinta
	419	Wasatch-Cache
	420	Desert Range Experiment Station
	499	Other NFS Areas
**Region 5**	501	Angeles
	502	Cleveland
	503	Eldorado
	504	Inyo
	505	Klamath
	506	Lassen
	507	Los Padres
	508	Mendocino
	509	Modoc
	510	Six Rivers
	511	Plumas
	512	San Bernardino
	513	Sequoia
	514	Shasta-Trinity
	515	Sierra
	516	Stanislaus
	517	Tahoe
	519	Lake Tahoe Basin
	599	Other NFS Areas
**Region 6**	601	Deschutes
	602	Fremont
	603	Gifford Pinchot
	604	Malheur
	605	Mt. Baker-Snoqualmie
	606	Mt. Hood
	607	Ochoco
	608	Okanogan
	609	Olympic
	610	Rogue River
	611	Siskiyou
	612	Siuslaw
	614	Umatilla
	615	Umpqua
	616	Wallowa-Whitman
	617	Wenatchee
	618	Willamette
	620	Winema
	621	Colville
	622	Columbia River Gorge NSA
	650	Crooked River National Grassland
	699	Other NFS Areas

Region	Code	National Forest/Grassland/Area
**Region 8**	801	NFS in Alabama
	802	Daniel Boone
	803	Chattahoochee-Oconee
	804	Cherokee
	805	NFS in Florida
	806	Kisatchie
	807	NFS in Mississippi
	808	George Washington
	809	Ouachita
	810	Ozark and St. Francis
	811	NFS in North Carolina
	812	Francis Marion-Sumter
	813	NFS in Texas
	814	Jefferson
	816	El Yunque
	899	Other NFS areas
**Region 9**	902	Chequamagon
	903	Chippewa
	904	Huron-Manistee
	905	Mark Twain
	906	Nicolet
	907	Ottawa
	908	Shawnee
	909	Superior
	910	Hiawatha
	912	Hoosier
	915	Midewin Tallgrass Prairie
	918	Wayne
	919	Allegheny
	920	Green Mountain
	921	Monongahela
	922	White Mountain
	999	Other NFS areas
**Region 10**	1004	Chugach
	1005	Tongass
	1099	Other NFS Areas

Appendix F. Tree Species Codes, Names, and Occurrences

Major groups (MAJGRP) are (1) pines, (2) other softwoods, (3) soft hardwoods, and (4) hard hardwoods. The 48 species groups (SPGRPCD) can be found in appendix G. The FIA work units listed are NC – (former) North Central, NE – (former) Northeastern, PNW – Pacific Northwest, RM – Rocky Mountain, and SO – Southern.

					Occurrence by FIA work unit				
**SPCD**	**COMMON NAME**	**SCIENTIFIC NAME**	**SPGRPCD**	**MAJGRP**	**NC**	**NE**	**PNW**	**RM**	**SO**
0010	fir spp.	*Abies* spp.	6	2	X	X			X
0011	Pacific silver fir	*Abies amabilis*	12	2			X		
0012	balsam fir	*Abies balsamea*	6	2	X	X			X
0014	Santa Lucia fir or bristlecone fir	*Abies bracteata*	12	2			X		
0015	white fir	*Abies concolor*	12	2	X		X	X	
0016	Fraser fir	*Abies fraseri*	9	2	X	X			X
0017	grand fir	*Abies grandis*	12	2			X	X	
0018	corkbark fir	*Abies lasiocarpa* var. *arizonica*	12	2				X	
0019	subalpine fir	*Abies lasiocarpa*	12	2			X	X	
0020	California red fir	*Abies magnifica*	12	2			X	X	
0021	Shasta red fir	*Abies shastensis*	12	2			X	X	
0022	noble fir	*Abies procera*	12	2			X	X	
0040	white-cedar spp.	*Chamaecyparis* spp.	9 E, 24 W	2		X	X		
0041	Port-Orford-cedar	*Chamaecyparis lawsoniana*	24	2			X		
0042	Alaska-yellow-cedar	*Chamaecyparis nootkatensis*	24	2			X		
0043	Atlantic white-cedar	*Chamaecyparis thyoides*	9	2		X			X
0050	cypress	*Cupressus* spp.	24	2			X		
0051	Arizona cypress	*Cupressus arizonica*	24	2			X	X	X
0052	Baker or Modoc cypress	*Cupressus bakeri*	24	2			X		
0053	Tecate cypress	*Cupressus forbesii*	24	2			X		
0054	Monterey cypress	*Cupressus macrocarpa*	24	2			X		
0055	Sargent's cypress	*Cupressus sargentii*	24	2			X		
0056	MacNab's cypress	*Cupressus macnabiana*	9 E, 24 W	2			X		
0057	redcedar / juniper spp.	*Juniperus* spp.	9 E, 23 W	2	X	X			X
0058	Pinchot juniper	*Juniperus pinchotii*	23	2				X	
0059	redberry juniper	*Juniperus coahuilensis*	23	2				X	X
0060	Drooping juniper	*Juniperus flaccida*	23	2					X
0061	Ashe juniper	*Juniperus ashei*	23	2	X				X
0062	California juniper	*Juniperus californica*	23	2			X	X	
0063	alligator juniper	*Juniperus deppeana*	23	2				X	X
0064	western juniper	*Juniperus occidentalis*	24	2			X	X	
0065	Utah juniper	*Juniperus osteosperma*	23	2			X	X	
0066	Rocky Mountain juniper	*Juniperus scopulorum*	9 E, 23 W	2	X		X	X	X
0067	southern redcedar	*Juniperus virginiana* var. *silicicola*	9	2					X
0068	eastern redcedar	*Juniperus virginiana*	9 E, 24 W	2	X	X		X	X
0069	oneseed juniper	*Juniperus monosperma*	23	2				X	X
0070	larch spp.	*Larix* spp.	9	2	X	X			
0071	tamarack (native)	*Larix laricina*	9 E, 24 W	2	X	X	X		
0072	subalpine larch	*Larix lyallii*	24	2			X	X	
0073	western larch	*Larix occidentalis*	19	2			X	X	
0081	incense-cedar	*Calocedrus decurrens*	20	2			X	X	
0090	spruce spp.	*Picea* spp.	6	2	X	X			X
0091	Norway spruce	*Picea abies*	9	2	X	X			X
0092	Brewer spruce	*Picea breweriana*	18	2			X		
0093	Engelmann spruce	*Picea engelmannii*	9 E, 18 W	2	X		X	X	
0094	white spruce	*Picea glauca*	6 E, 18 W	2	X	X	X	X	X
0095	black spruce	*Picea mariana*	6 E, 18 W	2	X	X	X		X
0096	blue spruce	*Picea pungens*	9 E, 18 W	2	X	X		X	X
0097	red spruce	*Picea rubens*	6	2		X			X
0098	Sitka spruce	*Picea sitchensis*	17	2			X		
0100	pine spp.	*Pinus* spp.	9 E, 24 W	1	X	X	X		

SPCD	COMMON NAME	SCIENTIFIC NAME	SPGRPCD	MAJGRP	Occurrence by FIA work unit				
					NC	NE	PNW	RM	SO
0101	whitebark pine	*Pinus albicaulis*	24	1			X	X	
0102	Rocky Mountain bristlecone pine	*Pinus aristata*	24	1				X	
0103	knobcone pine	*Pinus attenuata*	24	1			X		
0104	foxtail pine	*Pinus balfouriana*	24	1			X	X	
0105	jack pine	*Pinus banksiana*	5	1	X	X			
0106	common or two-needle pinyon	*Pinus edulis*	23	1			X	X	X
0107	sand pine	*Pinus clausa*	3	1					X
0108	lodgepole pine	*Pinus contorta*	21	1	X		X	X	
0109	Coulter pine	*Pinus coulteri*	24	1			X		
0110	shortleaf pine	*Pinus echinata*	2	1	X	X			X
0111	slash pine	*Pinus elliottii*	1	1					X
0112	Apache pine	*Pinus engelmannii*	24	1				X	
0113	limber pine	*Pinus flexilis*	24	1	X		X	X	X
0114	southwestern white pine	*Pinus strobiformis*	24	1				X	
0115	spruce pine	*Pinus glabra*	3	1					X
0116	Jeffrey pine	*Pinus jeffreyi*	11	1			X	X	
0117	sugar pine	*Pinus lambertiana*	14	1			X	X	
0118	Chihuahua pine	*Pinus leiophylla*	24	1				X	
0119	western white pine	*Pinus monticola*	15	1			X	X	
0120	bishop pine	*Pinus muricata*	24	1			X		
0121	longleaf pine	*Pinus palustris*	1	1					X
0122	ponderosa pine	*Pinus ponderosa*	9 E, 11 W	1	X		X	X	X
0123	Table mountain pine	*Pinus pungens*	3	1		X			X
0124	Monterey pine	*Pinus radiata*	24	1			X		
0125	red pine	*Pinus resinosa*	4	1	X	X			X
0126	pitch pine	*Pinus rigida*	3	1		X			X
0127	gray pine or California foothill pine	*Pinus sabiniana*	24	1			X		
0128	pond pine	*Pinus serotina*	3	1		X			X
0129	eastern white pine	*Pinus strobus*	4	1	X	X			X
0130	Scotch pine	*Pinus sylvestris*	3 E, 24 W	1	X	X	X	X	X
0131	loblolly pine	*Pinus taeda*	2	1	X	X			X
0132	Virginia pine	*Pinus virginiana*	3	1	X	X			X
0133	singleleaf pinyon	*Pinus monophylla*	23	1			X	X	
0134	border pinyon	*Pinus discolor*	23	1				X	
0135	Arizona pine	*Pinus arizonica*	11	1				X	
0136	Austrian pine	*Pinus nigra*	9 E, 24 W	1	X	X		X	X
0137	Washoe pine	*Pinus washoensis*	24	1			X	X	
0138	four-leaf pine or Parry pinyon pine	*Pinus quadrifolia*	24	1			X		
0139	Torrey pine	*Pinus torreyana*	24	1			X		
0140	Mexican pinyon pine	*Pinus cembroides*	23	1				X	X
0141	papershell pinyon pine	*Pinus remota*	23	1					X
0142	Great Basin bristlecone pine	*Pinus longaeva*	24	1			X	X	
0143	Arizona pinyon pine	*Pinus monophylla* var. *fallax*	23	1				X	
0144	Honduras pine	*Pinus elliottii* var. *elliottii*	9 E, 24 W	1					X
0200	Douglas-fir spp.	*Pseudotsuga* spp.	9 E, 10 W	2	X		X		
0201	bigcone Douglas-fir	*Pseudotsuga macrocarpa*	10	2			X		
0202	Douglas-fir	*Pseudotsuga menziesii*	9 E, 10 W	2	X	X	X	X	
0211	redwood	*Sequoia sempervirens*	16	2			X		
0212	giant sequoia	*Sequoiadendron giganteum*	24	2			X		
0220	baldcypress spp.	*Taxodium* spp.	9 E, 24 W	2	X	X			X
0221	baldcypress	*Taxodium distichum*	8	2	X	X			X
0222	pondcypress	*Taxodium ascendens*	8	2					X
0223	Montezuma baldcypress	*Taxodium mucronatum*	8	2					X
0230	yew spp.	*Taxus* spp.	9 E, 24 W	2	X		X		
0231	Pacific yew	*Taxus brevifolia*	24	2			X	X	
0232	Florida yew	*Taxus floridana*	9 E, 24 W	2					X

SPCD	COMMON NAME	SCIENTIFIC NAME	SPGRPCD	MAJGRP	Occurrence by FIA work unit NC	NE	PNW	RM	SO
0240	Thuja spp.	*Thuja* spp.	9 E, 24 W	2	X		X		
0241	northern white-cedar	*Thuja occidentalis*	9	2	X	X			X
0242	western redcedar	*Thuja plicata*	22	2			X	X	
0250	Torreya (nutmeg) spp.	*Torreya* spp.	9 E, 24 W	2			X		
0251	California torreya (nutmeg)	*Torreya californica*	24	2			X		
0252	Florida torreya (nutmeg)	*Torreya taxifolia*	9	2					X
0260	hemlock spp.	*Tsuga* spp.	7	2	X				X
0261	eastern hemlock	*Tsuga canadensis*	7	2	X	X			X
0262	Carolina hemlock	*Tsuga caroliniana*	7	2					X
0263	western hemlock	*Tsuga heterophylla*	13	2			X	X	
0264	mountain hemlock	*Tsuga mertensiana*	24	2			X	X	
0299	Unknown dead conifer	*Tree evergreen*	9 E, 24 W	2	X	X	X	X	X
0300	acacia spp.	*Acacia* spp.	41 E, 48 W	3			X		
0303	sweet acacia	*Acacia farnesiana*	43 E, 48 W	3				X	X
0304	catclaw acacia	*Acacia greggii*	43 E, 48 W	3			X	X	X
0310	maple spp.	*Acer* spp.	31	4	X	X			X
0311	Florida maple	*Acer barbatum*	31	4					X
0312	bigleaf maple	*Acer macrophyllum*	47	3			X		X
0313	boxelder	*Acer negundo*	41 E, 47 W	3	X	X	X	X	X
0314	black maple	*Acer nigrum*	31	4	X	X			X
0315	striped maple	*Acer pensylvanicum*	43	3	X	X			X
0316	red maple	*Acer rubrum*	32	3	X	X			X
0317	silver maple	*Acer saccharinum*	32	3	X	X			X
0318	sugar maple	*Acer saccharum*	31	4	X	X			X
0319	mountain maple	*Acer spicatum*	43	4	X	X			X
0320	Norway maple	*Acer platanoides*	31 E, 47 W	4	X	X			X
0321	Rocky Mountain maple	*Acer glabrum*	43 E, 48 W	4	X		X		
0322	bigtooth maple	*Acer grandidentatum*	48	4			X	X	
0323	chalk maple	*Acer leucoderme*	31	4					X
0330	buckeye, horsechestnut spp.	*Aesculus* spp.	41 E, 47 W	3	X	X			X
0331	Ohio buckeye	*Aesculus glabra*	41 E, 47 W	3	X	X			X
0332	yellow buckeye	*Aesculus flava*	43	3	X	X			X
0333	California buckeye	*Aesculus californica*	41 E, 47 W	3			X		
0334	Texas buckeye	*Aesculus glabra* var. *arguta*	41	3	X				X
0336	red buckeye	*Aesculus pavia*	43 E, 47 W	3	X	X			X
0337	painted buckeye	*Aesculus sylvatica*	41 E, 47 W	3		X			X
0341	ailanthus	*Ailanthus altissima*	43 E, 47 W	4	X	X	X		X
0345	mimosa, silktree	*Albizia julibrissin*	43	3	X				X
0350	alder spp.	*Alnus* spp.	41 E, 47 W	3	X		X		
0351	red alder	*Alnus rubra*	45	3			X	X	X
0352	white alder	*Alnus rhombifolia*	47	3			X	X	
0353	Arizona alder	*Alnus oblongifolia*	43 E, 47 W	3		X			
0355	European alder	*Alnus glutinosa*	41 E, 47 W	3	X				X
0356	serviceberry spp.	*Amelanchier* spp.	43 E, 48 W	4	X	X			X
0357	common serviceberry	*Amelanchier arborea*	43 E, 48 W	4	X				
0358	roundleaf serviceberry	*Amelanchier sanguinea*	43 E, 48 W	4	X				
0360	Madrone spp.	*Arbutus* spp.	43 E, 47 W	4			X		
0361	Pacific madrone	*Arbutus menziesii*	47	4			X	X	
0362	Arizona madrone	*Arbutus arizonica*	43 E, 47 W	4			X		
0363	Texas madrone	*Arbutus xalapensis*	48	4					X
0367	pawpaw	*Asimina triloba*	43	3	X	X			X
0370	birch spp.	*Betula* spp.	41	4	X	X			X
0371	yellow birch	*Betula alleghaniensis*	30	4	X	X			X
0372	sweet birch	*Betula lenta*	42	4	X	X			X
0373	river birch	*Betula nigra*	41	3	X	X			X
0374	water birch	*Betula occidentalis*	41 E, 47 W	3	X		X		X
0375	paper birch	*Betula papyrifera*	41 E, 47 W	3	X	X	X	X	
0377	Virginia roundleaf birch	*Betula uber*	41 E, 47 W	3					X

SPCD	COMMON NAME	SCIENTIFIC NAME	SPGRPCD	MAJGRP	Occurrence by FIA work unit NC	NE	PNW	RM	SO
0378	northwestern paper birch	*Betula x utahensis*	47	3			X		
0379	gray birch	*Betula populifolia*	41	3	X	X			X
0381	chittamwood,gum bumelia	*Sideroxylon lanuginosum* ssp. *lanuginosum*	43	4	X				X
0391	American hornbeam, musclewood	*Carpinus caroliniana*	43	4	X	X			X
0400	hickory spp.	*Carya* spp.	29	4	X	X			X
0401	water hickory	*Carya aquatica*	29	4	X				X
0402	bitternut hickory	*Carya cordiformis*	29	4	X	X			X
0403	pignut hickory	*Carya glabra*	29	4	X	X			X
0404	pecan	*Carya illinoinensis*	29 E, 47 W	4	X	X		X	X
0405	shellbark hickory	*Carya laciniosa*	29	4	X	X			X
0406	nutmeg hickory	*Carya myristiciformis*	29	4					X
0407	shagbark hickory	*Carya ovata*	29	4	X	X			X
0408	black hickory	*Carya texana*	29	4	X				X
0409	mockernut hickory	*Carya alba*	29	4	X	X			X
0410	sand hickory	*Carya pallida*	29	4	X	X			X
0411	scrub hickory	*Carya floridana*	29 E, 47 W	4					X
0412	red hickory	*Carya ovalis*	29 E, 47 W	4	X	X			X
0413	southern shagbark hickory	*Carya carolinae-septentrionalis*	29 E, 47 W	4					X
0420	chestnut spp.	*Castanea* spp.	43 E, 47 W	3	X	X			X
0421	American chestnut	*Castanea dentata*	43	3	X	X			X
0422	Allegheny chinkapin	*Castanea pumila*	43	3	X	X			X
0423	Ozark chinkapin	*Castanea pumila* var. *ozarkensis*	43	3	X				X
0424	Chinese chestnut	*Castanea mollissima*	43 E, 47 W	3	X	X			X
0431	giant chinkapin,golden chinkapin	*Chrysolepis chrysophylla* var. *chrysophylla*	47	3			X		
0450	catalpa spp.	*Catalpa* spp.	42	4	X	X			X
0451	southern catalpa	*Catalpa bignonioides*	43	4	X				X
0452	northern catalpa	*Catalpa speciosa*	41	3	X	X			X
0460	hackberry spp.	*Celtis*	41	3	X	X			X
0461	sugarberry	*Celtis laevigata*	41 E, 47 W	3	X	X			X
0462	hackberry	*Celtis occidentalis*	41 E, 47 W	3	X	X			X
0463	netleaf hackberry	*Celtis laevigata* var. *reticulata*	41	3	X				X
0471	eastern redbud	*Cercis canadensis*	43	3	X	X			X
0475	curlleaf mountain-mahogany	*Cercocarpus ledifolius*	48	4			X	X	
0481	yellowwood	*Cladrastis kentukea*	43	4	X	X			X
0490	dogwood spp.	*Cornus* spp.	43 E, 47 W	4	X	X	X		
0491	flowering dogwood	*Cornus florida*	42	4	X	X			X
0492	Pacific dogwood	*Cornus nuttallii*	47	4			X	X	
0500	hawthorn spp.	*Crataegus* spp.	43 E, 47 W	4	X	X			X
0501	cockspur hawthorn	*Crataegus crus-galli*	43	4	X	X			X
0502	downy hawthorn	*Crataegus mollis*	43	4	X	X			X
0503	Brainerd's hawthorn	*Crataegus brainerdii*	43 E, 47 W	4	X	X			X
0504	pear hawthorn	*Crataegus calpodendron*	43 E, 47 W	4	X	X			X
0505	fireberry hawthorn	*Crataegus chrysocarpa*	43 E, 47 W	4	X	X			X
0506	broadleaf hawthorn	*Crataegus dilatata*	43 E, 47 W	4	X	X			X
0507	fanleaf hawthorn	*Crataegus flabellata*	43 E, 47 W	4	X	X			X
0508	oneseed hawthorn	*Crataegus monogyna*	43 E, 47 W	4	X	X			X
0509	scarlet hawthorn	*Crataegus pedicellata*	43 E, 47 W	4	X	X			X
5091	Washington hawthorn	*Crataegus phaenopyrum*	43 E, 47 W	4	X	X			X
5092	fleshy hawthorn	*Crataegus succulenta*	43 E, 47 W	4	X	X			X
5093	dwarf hawthorn	*Crataegus uniflora*	43 E, 47 W	4	X	X			X
0510	eucalyptus spp.	*Eucalyptus* spp.	42 E, 47 W	4			X	X	X
0511	Tasmanian bluegum	*Eucalyptus globulus*	43 E, 47 W	4			X		
0512	river redgum	*Eucalyptus camaldulensis*	43 E, 47 W	4			X		
0513	grand eucalyptus	*Eucalyptus grandis*	43 E, 47 W	4			X		X
0514	swampmahogany	*Eucalyptus robusta*	43 E, 47 W	4					X
0520	persimmon spp.	*Diospyros* spp.	43 E, 47 W	4	X	X			X
0521	common persimmon	*Diospyros virginiana*	42	4	X	X			X

					Occurrence by FIA work unit				
**SPCD**	**COMMON NAME**	**SCIENTIFIC NAME**	**SPGRPCD**	**MAJGRP**	**NC**	**NE**	**PNW**	**RM**	**SO**
0522	Texas persimmon	*Diospyros texana*	43 E, 47 W	4					X
0523	Anacua knockaway	*Ehretia anacua*	48	3					X
0531	American beech	*Fagus grandifolia*	33	4	X	X			X
0540	ash spp.	*Fraxinus* spp.	36 E, 47 W	3	X	X	X		X
0541	white ash	*Fraxinus americana*	36	4	X	X			X
0542	Oregon ash	*Fraxinus latifolia*	47	4			X		
0543	black ash	*Fraxinus nigra*	36	3	X	X			X
0544	green ash	*Fraxinus pennsylvanica*	36 E, 47 W	4	X	X		X	X
0545	pumpkin ash	*Fraxinus profunda*	36	3	X	X			X
0546	blue ash	*Fraxinus quadrangulata*	36	4	X	X			X
0547	velvet ash	*Fraxinus velutina*	47	4				X	X
0548	Carolina ash	*Fraxinus caroliniana*	36	4					X
0549	Texas ash	*Fraxinus texensis*	36 E, 47 W	3					X
5491	Berlandier ash	*Fraxinus berlandieriana*	36	3					X
0550	honeylocust spp.	*Gleditsia* spp.	42 E, 47 W	4	X	X	X		
0551	waterlocust	*Gleditsia aquatica*	42	4	X				X
0552	honeylocust	*Gleditsia triacanthos*	42 E, 47 W	4	X	X		X	X
0555	loblolly-bay	*Gordonia lasianthus*	41	3					X
0561	Ginkgo, maidenhair tree	*Ginkgo biloba*	43 E, 47 W	3	X	X	X		
0571	Kentucky coffeetree	*Gymnocladus dioicus*	42	4	X	X			X
0580	silverbell spp.	*Halesia* spp.	43	3	X	X			X
0581	Carolina silverbell	*Halesia carolina*	41 E, 47 W	3					X
0582	two-wing silverbell	*Halesia diptera*	41 E, 47 W	3					X
0583	little silverbell	*Halesia parviflora*	41 E, 47 W	3					X
0591	American holly	*Ilex opaca*	42 E, 47 W	4	X	X	X		X
0600	walnut spp.	*Juglans* spp.	41 E, 47 W	4	X	X	X	X	X
0601	butternut	*Juglans cinerea*	41	3	X	X			X
0602	black walnut	*Juglans nigra*	40 E, 47 W	4	X	X	X	X	X
0603	northern California black walnut	*Juglans hindsii*	47	4			X		
0604	southern California black walnut	*Juglans californica*	47	4			X		
0605	Texas walnut	*Juglans microcarpa*	41 E, 47 W	4	X				X
0606	Arizona walnut	*Juglans major*	43 E, 47 W	4			X		
0611	sweetgum	*Liquidambar styraciflua*	34 E, 47 W	3	X	X			X
0621	yellow-poplar	*Liriodendron tulipifera*	39	3	X	X			X
0631	tanoak	*Lithocarpus densiflorus*	47	4			X		
0641	Osage-orange	*Maclura pomifera*	43	4	X	X			X
0650	magnolia spp.	*Magnolia* spp.	41	3	X	X			X
0651	cucumbertree	*Magnolia acuminata*	41	3	X	X			X
0652	southern magnolia	*Magnolia grandiflora*	41	3		X			X
0653	sweetbay	*Magnolia virginiana*	43	3		X			X
0654	bigleaf magnolia	*Magnolia macrophylla*	43	4		X			X
0655	mountain or Fraser magnolia	*Magnolia fraseri*	41	3		X			X
0657	pyramid magnolia	*Magnolia pyramidata*	41 E, 47 W	3					X
0658	umbrella magnolia	*Magnolia tripetala*	41 E, 47 W	3		X	X		X
0660	apple spp.	*Malus* spp.	43 E, 47 W	4	X	X	X	X	X
0661	Oregon crab apple	*Malus fusca*	47	4			X		
0662	southern crabapple	*Malus angustifolia*	43 E, 47 W	4	X	X			X
0663	sweet crabapple	*Malus coronaria*	43 E, 47 W	4	X	X			X
0664	prairie crabapple	*Malus ioensis*	43 E, 47 W	4	X				
0680	mulberry spp.	*Morus* spp.	42	4	X	X		X	X
0681	white mulberry	*Morus alba*	42	4	X	X			X
0682	red mulberry	*Morus rubra*	42	4	X	X			X
0683	Texas mulberry	*Morus microphylla*	42 E, 47 W	4					X
0684	black mulberry	*Morus nigra*	43 E, 47 W	4		X			X
0690	tupelo spp.	*Nyssa* spp.	35 E, 47 W	3	X	X			X
0691	water tupelo	*Nyssa aquatica*	35	3	X				X
0692	Ogeechee tupelo	*Nyssa ogeche*	43	4					X

SPCD	COMMON NAME	SCIENTIFIC NAME	SPGRPCD	MAJGRP	Occurrence by FIA work unit				
					NC	NE	PNW	RM	SO
0693	blackgum	*Nyssa sylvatica*	35	3	X	X			X
0694	swamp tupelo	*Nyssa biflora*	35	3	X	X			X
0701	eastern hophornbeam	*Ostrya virginiana*	43	4	X	X			X
0711	sourwood	*Oxydendrum arboreum*	43	4	X	X			X
0712	paulownia, empress-tree	*Paulownia tomentosa*	41	3	X	X			X
0720	bay spp.	*Persea* spp.	43 E, 47 W	3		X			X
0721	redbay	*Persea borbonia*	41	3					X
7211	avocado	*Persea americana*	43 E, 47 W	3					X
0722	water-elm, planertree	*Planera aquatica*	43	3	X				X
0729	Sycamore spp.	*Platanus* spp.	41 E, 47 W	3	X	X	X		
0730	California sycamore	*Platanus racemosa*	47	3			X		
0731	American sycamore	*Platanus occidentalis*	41 E, 47 W	3	X	X	X	X	X
0732	Arizona sycamore	*Platanus wrightii*	41 E, 47 W	3			X		
0740	cottonwood and poplar spp.	*Populus* spp.	37 E, 44 W	3	X	X			X
0741	balsam poplar	*Populus balsamifera*	37 E, 44 W	3	X	X		X	X
0742	eastern cottonwood	*Populus deltoides*	37 E, 44 W	3	X	X		X	X
0743	bigtooth aspen	*Populus grandidentata*	37	3	X	X			X
0744	swamp cottonwood	*Populus heterophylla*	37	3	X	X			X
0745	plains cottonwood	*Populus deltoides* ssp. *monilifera*	37 E, 44 W	3	X			X	
0746	quaking aspen	*Populus tremuloides*	37 E, 44 W	3	X	X	X	X	X
0747	black cottonwood	*Populus balsamifera* ssp. *trichocarpa*	37 E, 44 W	4	X		X	X	
0748	Fremont cottonwood	*Populus fremontii*	37 E, 44 W	4			X	X	X
0749	narrowleaf cottonwood	*Populus angustifolia*	37 E, 44 W	3	X			X	X
0752	silver poplar	*Populus alba*	37	3	X				X
0753	Lombardy poplar	*Populus nigra*	37 E, 44 W	3	X	X	X		
0755	mesquite spp.	*Prosopis* spp.	48	4					X
0756	honey mesquite	*Prosopis glandulosa*	48	4			X	X	X
0757	velvet mesquite	*Prosopis velutina*	48	4			X	X	X
0758	screwbean mesquite	*Prosopis pubescens*	48	4			X	X	X
0760	cherry and plum spp.	*Prunus* spp.	43 E, 47 W	4	X	X	X		X
0761	pin cherry	*Prunus pensylvanica*	43	3	X	X			X
0762	black cherry	*Prunus serotina*	41	3	X	X			X
0763	chokecherry	*Prunus virginiana*	43 E, 47 W	4	X	X	X		X
0764	peach	*Prunus persica*	43 E, 47 W	3	X	X			X
0765	Canada plum	*Prunus nigra*	43	4	X				
0766	American plum	*Prunus americana*	43	4	X	X			X
0768	bitter cherry	*Prunus emarginata*	47	4			X		
0769	Allegheny plum	*Prunus alleghaniensis*	43 E, 47 W	3	X	X			X
0770	Chickasaw plum	*Prunus angustifolia*	43 E, 47 W	3	X	X			X
0771	sweet cherry, domesticated	*Prunus avium*	43 E, 47 W	3	X	X	X		
0772	sour cherry, domesticated	*Prunus cerasus*	43 E, 47 W	3	X	X	X		
0773	European plum, domesticated	*Prunus domestica*	43 E, 47 W	3	X	X	X		
0774	Mahaleb cherry, domesticated	*Prunus mahaleb*	43 E, 47 W	3	X	X	X		
0800	oak spp	*Quercus* spp.	42 E, 48 W	4	X	X	X		X
0801	California live oak	*Quercus agrifolia*	46	4			X		
0802	white oak	*Quercus alba*	25	4	X	X			X
0803	Arizona white oak	*Quercus arizonica*	48	4				X	X
0804	swamp white oak	*Quercus bicolor*	25	4	X	X			X
0805	canyon live oak	*Quercus chrysolepis*	46	4			X		
0806	scarlet oak	*Quercus coccinea*	28	4	X	X			X
0807	blue oak	*Quercus douglasii*	46	4			X		
0808	Durand oak	*Quercus sinuata* var. *sinuata*	25	4					X
0809	northern pin oak	*Quercus ellipsoidalis*	28	4	X	X			X
0810	Emory oak	*Quercus emoryi*	48	4				X	X
0811	Engelmann oak	*Quercus engelmannii*	46	4			X		
0812	southern red oak	*Quercus falcata*	28	4	X	X			X

SPCD	COMMON NAME	SCIENTIFIC NAME	SPGRPCD	MAJGRP	Occurrence by FIA work unit				
					NC	NE	PNW	RM	SO
0813	cherrybark oak	*Quercus pagoda*	26	4	X	X			X
0814	Gambel oak	*Quercus gambelii*	48	4				X	X
0815	Oregon white oak	*Quercus garryana*	46	4			X		
0816	scrub oak	*Quercus ilicifolia*	43	4		X			X
0817	shingle oak	*Quercus imbricaria*	28	4	X	X			X
0818	California black oak	*Quercus kelloggii*	46	4			X		
0819	turkey oak	*Quercus laevis*	43	4					X
0820	laurel oak	*Quercus laurifolia*	28	4		X			X
0821	California white oak	*Quercus lobata*	46	4			X		
0822	overcup oak	*Quercus lyrata*	27	4	X	X			X
0823	bur oak	*Quercus macrocarpa*	25 E, 47 W	4	X	X		X	X
0824	blackjack oak	*Quercus marilandica*	28	4	X	X			X
0825	swamp chestnut oak	*Quercus michauxii*	25	4	X	X			X
0826	chinkapin oak	*Quercus muehlenbergii*	25 E, 47 W	4	X	X		X	X
0827	water oak	*Quercus nigra*	28	4	X	X			X
0828	Texas red oak	*Quercus texana*	28	4	X				X
0829	Mexican blue oak	*Quercus oblongifolia*	48	4				X	
0830	pin oak	*Quercus palustris*	28	4	X	X			X
0831	willow oak	*Quercus phellos*	28	4	X	X			X
0832	chestnut oak	*Quercus prinus*	27	4	X	X			X
0833	northern red oak	*Quercus rubra*	26	4	X	X			X
0834	Shumard oak	*Quercus shumardii*	26	4	X	X			X
0835	post oak	*Quercus stellata*	27	4	X	X			X
0836	Delta post oak	*Quercus similis*	27	4					X
0837	black oak	*Quercus velutina*	28	4	X	X			X
0838	live oak	*Quercus virginiana*	27	4					X
0839	interior live oak	*Quercus wislizeni*	46	4			X		
0840	dwarf post oak	*Quercus margarettiae*	27	4	X				X
0841	dwarf live oak	*Quercus minima*	27	4					X
0842	bluejack oak	*Quercus incana*	43	4					X
0843	silverleaf oak	*Quercus hypoleucoides*	48	4				X	X
0844	Oglethorpe oak	*Quercus oglethorpensis*	27	4					X
0845	dwarf chinkapin oak	*Quercus prinoides*	43	4	X				X
0846	gray oak	*Quercus grisea*	48	4				X	X
0847	netleaf oak	*Quercus rugosa*	43 E, 48 W	4				X	
0851	Chisos oak	*Quercus gracilliformis*	26	4					X
8511	Graves oak	*Quercus gravesii*	26	4					X
8512	Mexican white oak	*Quercus polymorpha*	26	4					X
8513	Buckley oak	*Quercus buckleyi*	26	4					X
8514	Lacey oak	*Quercus laceyi*	26	4					X
0852	sea torchwood	*Amyris elemifera*	43 E, 47 W	3					X
0853	pond-apple	*Annona glabra*	43 E, 47 W	3					X
0854	gumbo limbo	*Bursera simaruba*	43 E, 47 W	3					X
0855	sheoak spp.	*Casuarina* spp.	43 E, 47 W	3					X
0856	gray sheoak	*Casuarina glauca*	43 E, 47 W	3					X
0857	belah	*Casuarina lepidophloia*	43 E, 47 W	3					X
0858	camphortree	*Cinnamomum camphora*	43 E, 47 W	3					X
0859	Florida fiddlewood	*Citharexylum fruticosum*	43 E, 47 W	3					X
0860	citrus spp.	*Citrus* spp.	43 E, 47 W	3					X
0863	tietongue, pigeon-plum	*Coccoloba diversifolia*	43 E, 47 W	3					X
0864	soldierwood	*Colubrina elliptica*	43 E, 47 W	3					X
0865	longleaf geigertree	*Cordia sebestena*	43 E, 47 W	3					X
8651	Anacahuita Texas Olive	*Cordia boissieri*	27	4					X
0866	carrotwood	*Cupaniopsis anacardioides*	43 E, 47 W	3					X
0867	bluewood	*Condalia hookeri*	48	4					X
0868	blackbead ebony	*Ebenopsis ebano*	42 E, 47 W	4					X
0869	great leadtree	*Leucaena pulverulenta*	43	3					X
0870	Texas sophora	*Sophora affinis*	42 E	4					X
0873	red stopper	*Eugenia rhombea*	43 E, 47 W	3					X
0874	butterbough, inkwood	*Exothea paniculata*	43 E, 47 W	3					X

SPCD	COMMON NAME	SCIENTIFIC NAME	SPGRPCD	MAJGRP	Occurrence by FIA work unit NC	NE	PNW	RM	SO
0876	Florida strangler fig	*Ficus aurea*	43 E, 47 W	3					X
0877	wild banyantree, shortleaf fig	*Ficus citrifolia*	43 E, 47 W	3					X
0882	beeftree, longleaf blolly	*Guapira discolor*	43 E, 47 W	3					X
0883	manchineel	*Hippomane mancinella*	43 E, 47 W	3					X
0884	false tamarind	*Lysiloma latisiliquum*	43 E, 47 W	3					X
0885	mango	*Mangifera indica*	43 E, 47 W	3					X
0886	Florida poisontree	*Metopium toxiferum*	43 E, 47 W	3					X
0887	fishpoison tree	*Piscidia piscipula*	43 E, 47 W	3					X
0888	octopus tree, schefflera	*Schefflera actinophylla*	43 E, 47 W	3					X
0890	false mastic	*Sideroxylon foetidissimum*	43 E, 47 W	3					X
0891	white bully, willow bustic	*Sideroxylon salicifolium*	43 E, 47 W	3					X
0895	paradisetree	*Simarouba glauca*	43 E, 47 W	3					X
0896	Java plum	*Syzygium cumini*	43 E, 47 W	3					X
0897	tamarind	*Tamarindus indica*	43 E, 47 W	3					X
0901	black locust	*Robinia pseudoacacia*	42 E, 47 W	4	X	X	X		X
0902	New Mexico locust	*Robinia neomexicana*	48	4				X	X
0906	Everglades palm, paurotis-palm	*Acoelorraphe wrightii*	43 E, 47 W	3					X
0907	Florida silver palm	*Coccothrinax argentata*	43 E, 47 W	3					X
0908	coconut palm	*Cocos nucifera*	43 E, 47 W	3					X
0909	royal palm spp.	*Roystonea* spp.	43 E, 47 W	3					X
0911	Mexican palmetto	*Sabal Mexicana*	41 E	3					X
0912	cabbage palmetto	*Sabal palmetto*	43 E, 47 W	3					X
0913	key thatch palm	*Thrinax morrisii*	43 E, 47 W	3					X
0914	Florida thatch palm	*Thrinax radiata*	43 E, 47 W	3					X
0915	other palms	*Family Arecaceae not listed above*	43 E, 47 W	3					X
0919	western soapberry	*Sapindus saponaria* var. *drummondii*	43	4	X				X
0920	willow spp.	*Salix* spp.	43 E, 47 W	3	X	X	X		X
0921	peachleaf willow	*Salix amygdaloides*	43	3	X				X
0922	black willow	*Salix nigra*	41 E, 47 W	3	X	X	X		X
0923	Bebb willow	*Salix bebbiana*	43 E, 47 W	3	X				
0924	Bonpland willow	*Salix bonplandiana*	41 E, 47 W	3					X
0925	coastal plain willow	*Salix caroliniana*	43 E, 47 W	3	X	X			X
0926	balsam willow	*Salix pyrifolia*	43 E, 47 W	3	X	X			
0927	white willow	*Salix alba*	41	3	X	X			X
0928	Scouler's willow	*Salix scouleriana*	41 E, 47 W	3	X		X		
0929	weeping willow	*Salix sepulcralis*	41 E, 47 W	3	X	X			X
0931	sassafras	*Sassafras albidum*	41	3	X	X			X
0934	mountain-ash spp.	*Sorbus* spp.	43 E, 47 W	4	X	X			X
0935	American mountain-ash	*Sorbus americana*	43	4	X	X			X
0936	European mountain-ash	*Sorbus aucuparia*	43	4		X			X
0937	northern mountain-ash	*Sorbus decora*	43 E, 47 W	4	X	X			
0940	West Indian mahogany	*Swietenia mahagoni*	43 E, 47 W	4					X
0950	basswood spp.	*Tilia* spp.	38	3	X	X			X
0951	American basswood	*Tilia americana*	38	3	X	X			X
0952	white basswood	*Tilia americana* var. *heterophylla*	38	3	X	X			X
0953	Carolina basswood	*Tilia americana* var. *caroliniana*	38	3	X				X
0970	elm spp.	*Ulmus*	41	3	X	X			X
0971	winged elm	*Ulmus alata*	41	4	X	X			X
0972	American elm	*Ulmus americana*	41 E, 47 W	3	X	X		X	X
0973	cedar elm	*Ulmus crassifolia*	41	3	X				X
0974	Siberian elm	*Ulmus pumila*	41 E, 47 W	3	X			X	X
0975	slippery elm	*Ulmus rubra*	41	3	X	X			X
0976	September elm	*Ulmus serotina*	41	3	X				X
0977	rock elm	*Ulmus thomasii*	42	4	X	X			X
0981	California-laurel	*Umbellularia californica*	47	4			X		
0982	Joshua tree	*Yucca brevifolia*	43 E, 47 W	3			X		

SPCD	COMMON NAME	SCIENTIFIC NAME	SPGRPCD	MAJGRP	Occurrence by FIA work unit NC	NE	PNW	RM	SO
0986	black-mangrove	*Avicennia germinans*	43 E, 47 W	4					X
0987	button mangrove	*Conocarpus erectus*	43 E, 47 W	4					
0988	white-mangrove	*Laguncularia racemosa*	43 E, 47 W	4					X
0989	American mangrove	*Rhizophora mangle*	43	4					X
0990	desert ironwood	*Olneya tesota*	43 E, 48 W	4			X		
0991	saltcedar	*Tamarix* spp.	43 E, 47 W	3	X	X	X		
0992	melaleuca	*Melaleuca quinquenervia*	41 E, 47 W	3					X
0993	chinaberry	*Melia azedarach*	43	4	X	X			X
0994	Chinese tallowtree	*Triadica sebifera*	43	4					X
0995	tungoil tree	*Vernicia fordii*	43	4					X
0996	smoketree	*Cotinus obovatus*	43	4	X				X
0997	Russian-olive	*Elaeagnus angustifolia*	43 E, 47 W	3	X				X
0998	unknown dead hardwood	*Tree broadleaf*	43 E, 47 W	3	X	X	X		X
0999	other or unknown live tree	*Tree unknown*	43 E, 47 W	3	X	X			X

Appendix G. Tree Species Group Codes

Species group name	Code
**Softwood species groups**	
**Eastern softwood species groups**	
Longleaf and slash pines	1
Loblolly and shortleaf pines	2
Other yellow pines	3
Eastern white and red pines	4
Jack pine	5
Spruce and balsam fir	6
Eastern hemlock	7
Cypress	8
Other eastern softwoods	9
**Western softwood species groups**	
Douglas-fir	10
Ponderosa and Jeffrey pines	11
True fir	12
Western hemlock	13
Sugar pine	14
Western white pine	15
Redwood	16
Sitka spruce	17
Engelmann and other spruces	18
Western larch	19
Incense-cedar	20
Lodgepole pine	21
Western redcedar	22
Western woodland softwoods	23
Other western softwoods	24
**Hardwood species groups**	
**Eastern hardwood species groups**	
Select white oaks	25
Select red oaks	26
Other white oaks	27
Other red oaks	28
Hickory	29
Yellow birch	30
Hard maple	31
Soft maple	32
Beech	33
Sweetgum	34
Tupelo and blackgum	35
Ash	36
Cottonwood and aspen	37
Basswood	38
Yellow-poplar	39
Black walnut	40
Other eastern soft hardwoods	41
Other eastern hard hardwoods	42
Eastern noncommercial hardwoods	43
**Western hardwood species groups**	
Cottonwood and aspen	44
Red alder	45
Oak	46
Other western hardwoods	47
Western woodland hardwoods	48

Appendix H. Damage Agent codes for PNW

Damage Agent is a 2-digit code with values 01 to 91. For Agent and Severity 1, 2 and 3: the agent and severity codes indicate the type of agents that were present on a tree and describe their severity. Several damaging agents are automatically of highest importance and should be coded before any other agents; these agents are grouped as Class I Agents. Class I insects, diseases, or physical injuries can seriously affect vegetation. Failure to account for these agents can result in large differences in predicted outcomes for tree growth, survival, vegetative composition and structure. Class II agents can be important in local situations; recording their incidence and severity provides valuable information for those situations. Class II agents are recorded when present but only after all Class I agents.

Agents and their severity ratings are grouped by broad category. Each category has a general agent and specific agents listed. The general codes should be used if there is any question as to the identity of the specific damaging agent.

Class I Agents

	Agents		Severity	
	**Code**	**Agent**	**Code**	**Severity**
**Bark beetles:**				
	01	General /other bark beetle	1	Unsuccessful current attack
	02	Mountain pine beetle	2	Successful current attack
	03	Douglas-fir beetle	3	Last year's successful attack
	04	Spruce beetle	4	Older dead
	05	Western pine beetle	5	Top kill
	06	Pine engraver beetle		
	07	Fir engraver beetle		
	08	Silver fir beetle		
	09	Red turpentine beetle		
	26	Jeffrey pine beetle		
	**Code**	**Agent**	**Code**	**Severity**
**Defoliators:**			0	No detectable defoliation
	10	General/other	1	Up to 33% of foliage (old and new missing/affected)
	11	Western blackheaded budworm	2	34 to 66% of foliage missing/affected
	12	Pine butterfly	3	67 to 100% of foliage missing/affected
	13	Douglas-fir tussock moth		
	14	Larch casebearer		
	15	Western spruce or Modoc budworm		
	16	Western hemlock looper		
	17	Sawflies		
	18	Needles and sheath miners		
	19	Gypsy moth		

Class I Agents

	Agents		Severity	
	**Code**	**Agent**	**Code**	**Severity**
**Root diseases:**	60	General/other	1	Tree is a live tally tree within 30 ft of a tree or stump that has a root disease to which the tally tree is susceptible
	61	Annosus root disease	2	Live tally tree with signs or symptoms diagnostic for root disease such as characteristic decay, stain, ectotrophic mycelia, mycelial fans, conks or excessive resin flow at the root collar. No visible crown deterioration.
	62	Armillaria root disease	3	Live tally tree with signs or symptoms diagnostic for root disease such as characteristic decay, stain, ectotrophic mycelia, mycelial fans, conks, or excessive resin flow at the root collar. Visible crown deterioration such as thinning chlorotic foliage, reduced terminal growth, and/or stress cones.
	63	Black stain root disease		
	65	Laminated root rot		
	66	Port-Orford-cedar root disease		
	**Code**	**Agent**	**Code**	**Severity**
**White pine blister rust:**	36	White pine blister rust	1	Branch infections located more than 2.0 feet from tree bole.
			2	Branch infections located 0.5 to 2.0 feet from bole.
			3	Bole infections present, Or: branch infections within 0.5 feet of bole
	**Code**	**Agent**	**Code**	**Severity**
**Sudden oak death (tanoak, coast live oak, black oak):**	31	Sudden oak death symptoms	1	Bleeding present on bole
			2	Bleeding present on bole and adjacent mortality present
			3	Laboratory confirmed sudden oak death

Class II Agents

	Agents		Severity	
	**Code**	**Agent**	**Code**	**Severity**
**Other insects:**	20	General	1	Bottlebrush or shortened leaders, 0-2 forks on the tree's stem, Or: <20% of the branches affected, Or: <50% of the bole has visible larval galleries.
	21	Shoot moths		
	22	Weevils		
	23	Wood borers		
	24	Balsam wooly adelgid (aphid)	2	3 or more forks on the tree's bole, Or: 20% or more of the branches are affected, Or: the terminal leader is dead, Or: ≥50% of the bole has visible larval galleries.
	25	Sitka spruce terminal weevil		
	**Code**	**Agent**	**Code**	**Severity**
**Stem-branch cankers:**	33	Diplodia blight	1	Branch infections present. <50% of the crown affected
	40	General/other		
	41	Western gall rust (*Pinus ponderosa, Pinus contorta*)	2	Branch infections present. ≥50% of the crown affected, Or: any infection on the bole.
	42	Commandra blister rust (*Pinus ponderosa*)		
	43	Stalactiform rust (*Pinus contorta*)		
	44	Atropellis canker (*Pinus* spp.*)*		
	45	Cytospoa or Phomopsis (*Pseudotsuga menziesii, Abies* spp.)		
	**Code**	**Agent**	**Code**	**Severity**
**Pitch canker:**	32	Pitch canker (CA *Pinus* spp.)	1	No bole canker + <10 infected branch tips
			2	No bole canker + ≥10 infected branch tips
			3	1 or more bole cankers + <10 infected branch tips
			4	1 or more bole cankers + ≥10 infected branch tips
	**Code**	**Agent**	**Code**	**Severity**
**Stem decays:**	46	General/other	1	1 conk on the stem or present at ground level
	47	Red ring rot (*Phellinus pini*)		
	48	Indian paint rot (*Echinodontium tinctorium*)	2	2 or more conks separated by <16 feet on bole
	49	Brown cubical rot (*Phaeolus schweinitzii)*	3	2 or more conks separated by ≥16 feet on bole
			4	No conks. Visible decay in the interior of the bole

Class II Agents

	Agents		Severity	
	**Code**	**Agent**	**Code**	**Severity**
**Special agents:**				
	50	Suppression	No severity rating	
	51	Excessively deformed sapling		
	**Code**	**Agent**	**Code**	**Severity**
**Foliar pathogens:**				
	55	General/other	1	<20% of foliage affected, or <20% of the crown contains brooms.
	56	Rhabdocline (only on *Pseudotsuga menziesii*)		
	57	Elytroderma (only on *Pinus ponderosa*)	2	≥20% of foliage affected, or ≥20% of the crown contains brooms.
	58	Broom rusts (only on *Abies, Picea, and Juniperus occidentalis*)		
	59	Swiss needle cast (only on *Pseudotsuga menziesii*)		
	**Code**	**Agent**	**Code**	**Severity**
**Animal agents:**				
	70	Animal; general/unknown	1	<20% of the crown is affected. Bole damage is restricted to less than half of circumference.
	71	Mountain beaver		
	72	Livestock		
	73	Deer or elk	2	≥20% of the crown is affected. Bole damage to half or more of circumference.
	74	Porcupines		
	75	Pocket gophers, squirrels, mice, voles, rabbits, hares		
	76	Beaver		
	77	Bear		
	78	Human (not logging)		
	**Code**	**Agent**	**Code**	**Severity**
**Weather agents:**				
	80	Weather; general/unknown	1	<20% of the crown is affected.
	81	Windthrow or wind breakage	2	≥20% of the crown is affected or any damage to the bole.
	82	Snow/ice bending or breakage		
	83	Frost damage on shoots		
	84	Winter desiccation		
	85	Drought/moisture deficiency		
	86	Sun scald		
	87	Lightning		

Class II Agents

	Agents		Severity	
	**Code**	**Agent**	**Code**	**Severity**
**Physical injury:**				
	90	Other; general/unknown	1	<20% of the crown is affected.
	91	Logging damage	2	≥20% of the crown is affected or any damage to the bole.
	92	Fire; basal scars or scorch		
	93	Improper planting		
	94	Air pollution or other chemical damage		
	**Code**	**Agent**	**Code**	**Severity**
**Physical defect:**				
	95	Unspecified physical defect	0	Severity is not rated
	96	Broken/missing top		
	97	Dead top		
	98	Forks and crooks (only if caused by old top out or dead top)		
	99	Checks/bole cracks		

Appendix I. FIA Inventories by State, Year, and Type

State code	State name	Date(s) of available periodic inventory data	Initiation of annual inventory
1	Alabama	1972, 1982, 1990, 2000	2001
2	Alaska	1998	2004
4	Arizona	1985, 1999	2001
5	Arkansas	1978, 1988, 1995	2000
6	California	1994	2001
8	Colorado	1984	2002
9	Connecticut	1985, 1998	2003
10	Delaware	1986, 1999	2004
12	Florida	1970, 1980, 1987, 1995	2003
13	Georgia	1972, 1982, 1989, 1987	1997
16	Idaho	1991	2004
17	Illinois	1985, 1998	2001
18	Indiana	1986, 1998	1999
19	Iowa	1990	1999
20	Kansas	1981, 1994	2001
21	Kentucky	1988	1999
22	Louisiana	1974, 1984, 1991	2001
23	Maine	1995	1999
24	Maryland	1986, 1999	2004
25	Massachusetts	1985, 1998	2003
26	Michigan	1980, 1993	2000
27	Minnesota	1977, 1990	1999
28	Mississippi	1977, 1987, 1994	2006
29	Missouri	1989	1999
30	Montana	1989	2003
31	Nebraska	1983, 1994	2001
32	Nevada	1989	2004 [1]
33	New Hampshire	1983, 1997	2002
34	New Jersey	1987, 1999	2004
35	New Mexico	1987, 1999	
36	New York	1993	2002
37	North Carolina	1984, 1990, 2002	2003
38	North Dakota	1980, 1995	2001
39	Ohio	1991	2001
40	Oklahoma	1989 (central/west),<br>1976, 1986, 1993 (east)	2008 (east)
41	Oregon	1992, 1999	2001
42	Pennsylvania	1989	2000
44	Rhode Island	1985, 1998	2003
45	South Carolina	1968, 1978, 1986, 1993	1999
46	South Dakota	1980, 1995	2001
47	Tennessee	1980, 1989, 1999	2000
48	Texas	1975, 1986, 1992	2001
49	Utah	1993	2000

State code	State name	Date(s) of available periodic inventory data	Initiation of annual inventory
50	Vermont	1983, 1997	2003
51	Virginia	1977, 1985, 1992	1998
53	Washington	1991, 2001	2002
54	West Virginia	1989, 2000	2004
55	Wisconsin	1983, 1996	2000
56	Wyoming	1984, 2000	
72	Puerto Rico	2001, 2002, 2003, 2004	
78	US Virgin Islands	2004	

[1] insufficient funding to continue annual inventory after 2005

Appendix J. Biomass Estimation in the FIADB

In previous versions of the FIADB, a variety of regional methods were used to estimate tree biomass for live and dead trees in the TREE table. In FIADB 4.0, a new nationally consistent method of estimating tree biomass has been implemented. This new approach, called the component ratio method (CRM) (Heath and others 2009), involves calculating the dry weight of individual components before estimating the total aboveground or belowground biomass. The CRM approach is based on:

- converting the sound volume of wood (VOLCFSND) in the merchantable bole to biomass using a compiled set of wood specific gravities (Miles and Smith 2009) (see REF_SPECIES table for values)
- calculating the biomass of bark on the merchantable bole using a compiled set of percent bark estimates and bark specific gravities (Miles and Smith 2009) (see REF_SPECIES table for values)
- calculating the biomass of the entire tree (total aboveground biomass), merchantable bole (including bark), and belowground biomass, using equations from Jenkins and others (2003)
- calculating the volume of the stump (wood and bark) based on equations in Raile (1982) and converting this to biomass using the same specific gravities used for the bole wood and bark
- calculating the top biomass (tree tip and all branches) by subtracting all other biomass components from the total aboveground estimate
- calculating an adjustment factor by developing a ratio between bole biomass calculated from VOLCFSND to bole biomass using equations from Jenkins and others (2003)
- applying the adjustment factor to all tree components derived from both Jenkins and Raile

The CRM approach is based on assumptions that the definition of merchantable bole in the volume prediction equations is equivalent to the bole (stem wood) in Jenkins and others (2003), and that the component ratios accurately apply.

The tables in this appendix describe the equations used in FIADB 4.0 to estimate components of tree biomass, including stem wood (bole), top and branches combined, bark, stump, and coarse roots. Most of these components are estimated through a series of ratio equations as described by Jenkins and others (2003). Stem wood biomass is calculated directly from the sound cubic-foot volume of the tree bole, percentage of bark on the bole, and specific gravities of both wood and bark.

The individual component biomass values for bole, top, and stump are not available in FIADB for sapling-size timber tree species and all woodland tree species. Because saplings (trees from 1 to 4.9 inches in diameter) have no volume in FIADB, a ratio method was developed to compute a factor that is applied to saplings based on diameter and species, and the result is stored in DRYBIO_SAPLING. For woodland species (trees where diameter is measured at the root collar [DRC]), volume is calculated from the root collar to a 1½-inch top diameter. Because this volume accounts for a larger portion of the tree than timber species volume equations do, it was determined that the top and stump equations were not applicable to woodland species. Woodland tree volume is converted to biomass and stored in DRYBIO_WDLD_SPP, which is an estimate for total aboveground biomass, excluding foliage, the tree tip (top of the tree above 1½ inches in diameter), and a portion of the stump from ground to DRC. Therefore, only total aboveground and belowground biomass values are estimated for saplings and woodland species.

Definitions of each biomass component and the equations used to estimate the oven-dry weight in pounds are shown in appendix tables J-1 through J-4.

- Appendix table J-1 defines the columns that are stored in the TREE table, and clarifies the set of trees (species, dimensions, live or dead, etc) that are used in each calculation.

- Appendix table J-2 defines the Jenkins component equations and explains how the equation results are used to estimate biomass. The 'Estimate name' in this table is the same name found in the coefficient definitions described in the biomass-related columns 38 to 49 of the REF_SPECIES table.

- Appendix table J-3 contains the Jenkins equations used to estimate each biomass component. The equations use the exact coefficient column names found in the REF_SPECIES table (for example, JENKINS_TOTAL_B1 in appendix table J-3 is the column name in REF_SPECIES that holds the value of the coefficient needed in the total aboveground biomass equation). The Jenkins equations use the measured tree diameter to produce an estimate.

- Appendix table J-4 contains the actual equations used in the FIADB to estimate the biomass components stored in the TREE table. These equations are a blend of Jenkins ratios, calculated bole biomass (based on calculated volume from the TREE table), and adjustment factors. The adjustment factor is an important step because it relates measurement-based bole biomass (DRYBIO_BOLE) to generalized equation-based bole biomass to improve or adjust the computed results of the Jenkins equations.

For more information please consult the publication by Heath and others (2009), titled *Investigation into Calculating Tree Biomass and Carbon in the FIADB Using a Biomass Expansion Factor Approach*.

Appendix table J-1. Definition of Biomass Components stored in the TREE table.

**Component**	**Column name**	**Biomass Component Definition (all are oven-dry biomass, pounds)**
Merchantable stem (bole)	DRYBIO_BOLE	Merchantable bole of the tree, includes stem wood and bark, from a 1-foot stump to a 4-inch top diameter. Based on VOLCFSND and specific gravity for the species. For timber species with a DIA ≥5 inches. Includes live and dead trees.<br>(Note that VOLCFGRS or VOLCFNET might be used after adjustment based on national averages, if VOLCFSND is not available.)
Top	DRYBIO_TOP	Top of the tree above 4 inches diameter and all branches; includes wood and bark and excludes foliage. For live and dead timber species with a DIA ≥5 inches.
Stump	DRYBIO_STUMP	Stump of the tree, the portion of a tree bole from ground to 1 foot high, includes wood and bark. For live and dead timber species with a DIA ≥5 inches.
Belowground	DRYBIO_BG	Coarse roots of trees and saplings with a DIA ≥1 inch. For timber and woodland species, and live and dead trees.
Saplings	DRYBIO_SAPLING	Total aboveground portion of live trees, excluding foliage. For timber species with a DIA ≥1 inch and <5 inches.
Woodland tree species	DRYBIO_WDLD_SPP	Total aboveground portion of a tree, excluding foliage, the tree tip (top of the tree above 1½ inches in diameter) and a portion of the stump from ground to DRC. For live and dead woodland species with a DIA ≥1 inch. Woodland species can be identified by REF_SPECIES.WOODLAND = X, TREE.DIAHTCD = 2, or TREE.WDLDSTEM >0

Appendix table J-2. Jenkins Biomass Component Equation Definitions
(Refer to the REF_SPECIES table for equation coefficients and adjustment factors).

Component	Estimate name	Definition
Total aboveground biomass	total_AG_biomass_ Jenkins	Total biomass (oven-dry, pounds) of the aboveground portion of a tree. Includes stem wood, stump, bark, top, branches, and foliage.
Stem wood biomass ratio	stem_ratio	A ratio that estimates biomass of the merchantable bole of the tree by applying the ratio to total_AG_biomass_Jenkins. Includes wood only. This is the portion of the tree from a 1-foot stump to a 4-inch top diameter.
Stem bark biomass ratio	bark_ratio	A ratio that estimates biomass of the bark on the merchantable bole of the tree by applying the ratio to total_AG_biomass_Jenkins.
Foliage biomass ratio	foliage_ratio	A ratio that estimates biomass of the foliage on the entire tree by applying the ratio to total_AG_biomass_Jenkins.
Coarse root biomass ratio	root_ratio	A ratio that estimates biomass of the belowground portion of the tree by applying the ratio to total_AG_biomass_Jenkins.
Stump biomass	stump_biomass	An estimate of the stump biomass of a tree, from the ground to 1 foot high. Uses a series of equations that first estimates the inside and outside bark diameters, then estimates inside and outside bark volumes (Raile 1982). Wood and bark volumes are converted to biomass using specific gravity for the species.
Sapling biomass adjustment	JENKINS_SAPLING _ADJUSTMENT	An adjustment factor that is used to estimate sapling biomass for the tree by applying the factor to the total aboveground estimate, excluding foliage. The adjustment factor was computed as a national average ratio of the DRYBIOT (total dry biomass) divided by the Jenkins total biomass for all 5.0-inch trees, which is the size at which biomass, based on volume, begins. This is used on timber and woodland species.

Appendix table J-3. Jenkins Biomass Equations (Actual B1 and B2 coefficients and adjustment factors are stored in the REF_SPECIES table.) Note: these equations are used in appendix table J-4 to estimate the biomass components stored in the TREE table.

Component	Equation
**total_AG_biomass_Jenkins** (pounds) (total aboveground biomass, includes wood and bark for stump, bole, top, branches, and foliage)	= exp(JENKINS_TOTAL_B1 + JENKINS_TOTAL_B2 * ln(DIA*2.54)) * 2.2046
**stem_ratio**	= exp(JENKINS_STEM_WOOD_RATIO_B1 + JENKINS_STEM_WOOD_RATIO_B2 / (DIA*2.54))
**bark_ratio**	= exp(JENKINS_STEM_BARK_RATIO_B1 + JENKINS_STEM_BARK_RATIO_B2 / (DIA*2.54))
**foliage_ratio**	= exp(JENKINS_FOLIAGE_RATIO_B1 + JENKINS_FOLIAGE_RATIO_B2 / (DIA*2.54))
**root_ratio**	= exp(JENKINS_ROOT_RATIO_B1 + JENKINS_ROOT_RATIO_B2 / (DIA*2.54))
**stem_biomass_Jenkins** (pounds)	= total_AG_biomass_Jenkins * stem_ratio
**bark_biomass_Jenkins** (pounds)	= total_AG_biomass_Jenkins * bark_ratio
**bole_biomass_Jenkins** (pounds)	= stem_biomass_Jenkins + bark_ biomass_Jenkins
**foliage_biomass_Jenkins** (pounds)	= total_AG_biomass_Jenkins * foliage_ratio
**root_biomass_Jenkins** (pounds)	= total_AG_biomass_Jenkins * root_ratio
**stump_biomass** (pounds)	Volumes of wood and bark are based on diameter inside bark (DIB) and DOB equations from Raile 1982.<br>DIB = (DIA * RAILE_STUMP_DIB_B1) + (DIA * RAILE_STUMP_DIB_B2 * (4.5-HT) / (HT+1))<br>DOB = DIA + (DIA * RAILE_STUMP_DOB_B1 * (4.5-HT) / (HT+1))<br>Volume is estimated for 0.1ft (HT) slices from ground to 1 foot high (HT), and summed to compute stump volume.<br>Bark_volume = Volume_outside_bark – Volume_inside_bark<br>Bark and wood volumes are multiplied by their respective specific gravities and added together to estimate biomass
**top_biomass_Jenkins** (pounds)	= total_AG_biomass_Jenkins – stem_biomass_Jenkins – bark_biomass_Jenkins – foliage_biomass_Jenkins – stump_biomass_Jenkins

Appendix table J-4. Equations used to calculate Biomass Components stored in the TREE table

**Column name**	**Equation (refer to Appendix table J-3 for details on variables found in equations below)**
	**AdjFac** = DRYBIO_BOLE / bole_biomass_Jenkins<br>**AdjFac_woodland** = DRYBIO_WDLD_SPP / (total_AG_biomass_Jenkins – foliage_biomass_Jenkins)
**DRYBIO_BOLE**<br><br>(wood and bark)<br>(see note below)<br><br>(timber species only)	VOLUME = VOLCFSND (or VOLCFGRS, VOLCFNET that are adjusted for the percent sound)<br>Volume = includes the volume of wood from a 1-foot stump to a 4-inch top diameter<br><br>= (VOLUME * (BARK_VOL_PCT / 100.0) * (BARK_SPGR_GREENVOL_DRYWT * 62.4)) +<br>(VOLUME * (WOOD_SPGR_GREENVOL_DRYWT * 62.4))
**DRYBIO_TOP**<br>(timber species only)	= top_biomass_Jenkins * **AdjFac**
**DRYBIO_STUMP**<br>(timber species only)	= stump_biomass * **AdjFac**
**DRYBIO_SAPLING**<br>(timber species only)	= (total_AG_biomass_Jenkins – foliage_biomass_Jenkins) * JENKINS_SAPLING_ADJUSTMENT
**DRYBIO_WDLD_SPP**<br>(woodland species only)	Woodland species are identified by REF_SPECIES.WOODLAND = X, TREE.DIAHTCD = 2, and/or TREE.WDLDSTEM >0<br><br>For woodland species, volume equations produce volume of wood and bark, from DRC to a 1½-inch top diameter, and includes branches. Biomass equations for each component are not available, therefore stem volume is converted to biomass and stored in DRYBIO_WDLD_SPP. This is an estimate of total aboveground biomass for woodland species, which includes wood and bark for the stem and branches and excludes foliage, the tree tip (top of the tree above 1½ inches in diameter), and a portion of the stump from the ground to the point of diameter measurement.<br><br>For trees with a DRC ≥5 inches:<br>VOLUME = VOLCFSND (or VOLCFGRS, VOLCFNET that are adjusted for the percent sound)<br>VOLUME = includes the volume of wood, bark, and branches<br><br>Wood and bark volumes need to be separated before converting to biomass as follows:<br>= (VOLUME * (BARK_VOL_PCT / 100.0) * (BARK_SPGR_GREENVOL_DRYWT * 62.4)) +<br>((VOLUME – (VOLUME * (BARK_VOL_PCT / 100.0))) * (WOOD_SPGR_GREENVOL_DRYWT * 62.4))<br><br>For trees with a DRC <5 inches:<br>= (total_AG_biomass_Jenkins – foliage_biomass_Jenkins) * JENKINS_SAPLING_ADJUSTMENT

Column name	Equation (refer to Appendix table J-3 for details on variables found in equations below)
**DRYBIO_BG** (timber and woodland species)	= root_biomass_Jenkins * **AdjFac** (for timber spp ≥5 inches DBH)<br>= root_biomass_Jenkins * JENKINS_SAPLING_ADJUSTMENT (for timber species <5 inches DBH)<br>= root_biomass_Jenkins * **AdjFac_woodland** (for woodland species ≥1 inch DRC)
Note:<br>If DIA ≥ 5.0 and VOLCFSND >0 then VOLUME = VOLCFSND<br>If DIA ≥ 5.0 and VOLCFSND = (0 or null) and VOLCFGRS >0 then VOLUME = VOLCFGRS * Percent Sound<br>If DIA ≥ 5.0 and VOLCFSND and VOLCFGRS = (0 or null) then VOLUME = VOLCFNET * (Average ratio of cubic foot sound to cubic foot net volume, calculated as national averages by species group and diameter)	

The Rocky Mountain Research Station develops scientific information and technology to improve management, protection, and use of the forests and rangelands. Research is designed to meet the needs of the National Forest managers, Federal and State agencies, public and private organizations, academic institutions, industry, and individuals. Studies accelerate solutions to problems involving ecosystems, range, forests, water, recreation, fire, resource inventory, land reclamation, community sustainability, forest engineering technology, multiple use economics, wildlife and fish habitat, and forest insects and diseases. Studies are conducted cooperatively, and applications may be found worldwide.

Station Headquarters
Rocky Mountain Research Station
240 W Prospect Road
Fort Collins, CO 80526
(970) 498-1100

Research Locations

Flagstaff, Arizona
Fort Collins, Colorado
Boise, Idaho
Moscow, Idaho
Bozeman, Montana
Missoula, Montana
Reno, Nevada
Albuquerque, New Mexico
Rapid City, South Dakota
Logan, Utah
Ogden, Utah
Provo, Utah

The U.S. Department of Agriculture (USDA) prohibits discrimination in all its programs and activities on the basis of race, color, national origin, age, disability, and where applicable, sex, marital status, familial status, parental status, religion, sexual orientation, genetic information, political beliefs, reprisal, or because all or part of an individual's income is derived from any public assistance program. (Not all prohibited bases apply to all programs.) Persons with disabilities who require alternative means for communication of program information (Braille, large print, audiotape, etc.) should contact USDA's TARGET Center at (202) 720-2600 (voice and TDD). To file a complaint of discrimination, write to USDA, Director, Office of Civil Rights, 1400 Independence Avenue, S.W., Washington, DC 20250-9410, or call (800) 795-3272 (voice) or (202) 720-6382 (TDD). USDA is an equal opportunity provider and employer.

Federal Recycling Program Printed on Recycled Paper
