# Supplementary material for: Variation in the diversity-productivity relationship in young forests of the eastern United States
Source: PLoS One. 2017 Nov 15;12(11):e0187106. doi: 10.1371/journal.pone.0187106 (PMC5687711; doi:10.1371/journal.pone.0187106)
Supplement: S5 File — (PDF) [file pone.0187106.s011.pdf]

United States
Department of
Agriculture

Forest Service

**Northern
Research Station**

Research Note NRS-38

Specific Gravity and Other Properties of Wood and Bark for 156 Tree Species Found in North America

Patrick D. Miles
W. Brad Smith

Abstract

Much information is available for specific gravity and other properties of wood and bark, but it is widely scattered in the literature. This paper compiles information for estimation of biomass for 156 tree species found in North America for use in national forest inventory applications. We present specific gravities based on average green volume as well as 12 percent moisture content volume for calculation of oven-dry biomass. Additional information is included on bark thickness, bark voids, and bark percentages by species and green and dry weight of wood and bark.

Manuscript received for publication 29 June 2009

Published by:
U.S. FOREST SERVICE
11 CAMPUS BLVD SUITE 200
NEWTOWN SQUARE PA 19073-3294

October 2009

For additional copies:
U.S. Forest Service
Publications Distribution
359 Main Road
Delaware, OH 43015-8640
Fax: (740)368-0152

Visit our homepage at: **http://www.nrs.fs.fed.us/**

INTRODUCTION

National forest inventories (NFIs) are a vital source of data for estimating the volume, biomass, and carbon in trees. In the United States, these estimates are based on data collected by the Forest Inventory and Analysis (FIA) program of the U.S. Forest Service during routine field inventories. Converting basic tree measurements from these inventories to volume, biomass, and carbon requires information for wood and bark, such as volume estimation equations, specific gravity, and percentage bark. This paper provides the variables needed to convert green volume estimates for the sound wood in the central stem from FIA data to biomass estimates for stemwood and bark. While there are more than 800 species of trees in U.S. forests (Little 1979), over 95 percent of the nation's forest tree volume resides in the 156 species presented in Table 1A. Specific gravity and other properties of wood and bark are presented in Tables 1 through 3 for these 156 North American tree species. Table 4 contains specific gravity estimates for all species currently tallied on FIA field plots in the continental United States.

METHODS

We collected specific gravity data for wood and bark based on green volume from published sources. These sources were selected on the basis of availability of data reporting specific gravity estimates from green volume and oven-dry weight (green specific gravity) for use with field inventory data. Table 1A presents values for green specific gravity from Jenkins et al. (2004) but with updated references. Additionally, specific gravity based on 12 percent moisture content (MC) volume and oven-dry weight is provided to facilitate estimating biomass from measurements of volumes of processed forest products (lumber, veneer, etc.). It should be noted that conversions involving biomass of primary forest products, such as saw logs and pulpwood, should be based on green specific gravity. Table 1B provides values for total oven-dry and green weight for combined wood and bark when only wood volume is known.

Information was collected for bark volume as a percentage of green wood volume from published sources as noted in the Tables 2A, 2B, and 3. Bark estimates based solely on double bark thickness or diameter inside (DIB) and outside (DOB) bark relationships will overestimate the true bark percentage due to unaccounted voids and fissures characteristically found in tree bark. It is unclear which of the referenced studies on bark accounted for this factor so estimates presented may overstate the average bark percent by 10 to 25 percent of the reported value depending on the species.

The Authors

PATRICK D. MILES is a Research Forester, U.S. Department of Agriculture, Forest Service, Northern Research Station, 1992 Folwell Ave., St. Paul, MN 55108; e-mail: pmiles@fs.fed.us

W. BRAD SMITH is the National Forest Inventory and Analysis Associate Program Manager, U.S. Department of Agriculture, Forest Service, Washington Office, 1601 N. Kent Street, 4th Floor, Arlington VA 22209; e-mail: bsmith12@fs.fed.us

Table 2A look-up values for average bark percent by diameter at breast height (d.b.h.) class (final percentages adjusted for estimated bark voids) were derived using the equation:

$$DBT = b0 + b1 * DIAM_{ob} \quad (1)$$

where
DBT = double bark thickness in inches
b0 and *b1* = regression coefficients from Table 2A
$DIAM_{ob}$ = diameter (inches) outside bark at specified stem location

This equation was used to estimate DBT at d.b.h. and at 4 inches top diameter outside bark. The DBT value was subtracted from each outside diameter to derive inside bark diameter at that point. The bark percentage at each point was then calculated. The bark percentage values at d.b.h. and 4 inches were averaged and then adjusted by the bark void factor to calculate the final bark percentage for each species and d.b.h. class as shown in Table 2A.

Table 2B provides estimates of bark percentage based on diameter inside bark regression data found in Hilt et al. (1983). Estimates of bark percentage were computed at d.b.h. and at 4 inches outside bark, averaged, and then adjusted for estimated bark voids. Table 2B look-up values for average bark percent by d.b.h. class were estimated using the equation:

$$DIB = b0 * DOB + b1\ DOB^2/DBH \quad (2)$$

where
DIB = diameter (inches) inside bark
b0 and *b1* = regression coefficients from Table 2B
DOB = diameter (inches) outside bark as specified stem location
DBH = diameter (inches) outside bark at breast height

Table 2A provides information on bark as a percentage of wood volume based on equations from published double bark thickness (DBT) regressions adjusted for bark voids; Table 2B provides information based on DIB/DOB relationships adjusted for bark voids. Table 3 presents bark percentages by species from averaged values in Tables 2A and 2B and other referenced studies. Species values in Table 3 were used to assign bark percentages for species with similar bark characteristics in Table 1A.

Calculating Biomass

Once central stem net wood volume has been estimated by a volume equation or other means, this value becomes the basis for central stem biomass estimates of both wood and bark. Central stem wood biomass is estimated using the equation:

$$B_{odw} = V_{gw} * SG_{gw} * W \quad (3)$$

where
B_{odw} = oven-dry biomass (pounds) of wood
V_{gw} = net volume (cu. ft.) of green wood in the central stem
SG_{gw} = green specific gravity of wood from Table 1A
W = weight of cu. ft. of water (62.4 pounds)

Central stem bark biomass is derived from:

$$B_{odb} = V_{gw} * BV\% * SG_{gb} * W \qquad (4)$$

where
B_{odb} = oven-dry biomass (pounds) of bark
V_{gw} = net volume (cu.ft.) of green wood in the central stem
BV% = bark as a percentage of wood volume (look-up values by d.b.h. class from Table 2 or average values from Table 1A or 3)
SG_{gb} = green specific gravity of bark from Table 1A
W = weight of cu. ft. of water (62.4 pounds)

To calculate total central stem biomass, the following equation was used:

$$B_{odt} = B_{odw} + B_{odb} \qquad (5)$$

where
B_{odw} = oven-dry biomass (pounds) of wood
B_{odb} = oven-dry biomass (pounds) of bark

And finally, to calculate total wood product biomass, this equation was used:

$$B_{odp} = V_p * SG_{12} * W \qquad (6)$$

where
B_{odp} = oven-dry biomass (pounds) of wood product (lumber, veneer, etc.)
V_p = volume (cu. ft.) of wood product
SG_{12} = specific gravity based on 12% MC volume of wood from Table 1A
W = weight of cu. ft. of water (62.4 pounds)

Working with Bark Data when Only Total Volume is Available

Generally, NFI data are presented in terms of wood volume and Eq. 3 and Eq. 4 are used to estimate total volume and biomass of wood and bark. If only gross volume of wood and bark is available, separate estimates of bark and wood volume may be useful. Tables 2 and 3 provide information on bark as a percentage of total wood and bark for several species and species groups. The percents are derived using the following formula:

$$BV\%_{total} = 100 * (BV\%_{wood} / (100 + BV\%_{wood})) \qquad (7)$$

where
$BV\%_{wood}$ = Bark volume expressed as a percentage of wood volume
$BV\%_{total}$ = Bark volume expressed as a percentage of wood and bark volume

Biomass Adjustments

Forest trees are subject to many damaging agents, such as weather, insects, disease, and fire that can affect tree volume and biomass estimates. Thus, adjustments may be needed to account for rotten or missing wood caused by these agents. This requires additional descriptive inventory data which may or may not be available. In the absence of such data,

models may be derived to estimate the average volume deduction for these factors to adjust final tree volume and biomass estimates. This need for adjustment is noted here as a caution when more precise values are required; such issues are not addressed in this paper.

Green Weight

In today's wood markets, wood is often sold based on green weight. Values are presented for average green weight in pounds per cubic foot and kilograms per cubic meter[1] to accommodate this calculation for both wood and bark. Green weights can be extremely variable geographically, seasonally, within species, and across various portions of individual trees. The values presented in Table 1A are useful for large-scale estimates but should be considered as rough estimates for localized areas. An average value for wood and bark combined may be derived with the following equation:

$$GWT_{wb} = GWT_w * (1 - BV\%_{total}/100) + GWT_b * BV\%_{total}/100 \qquad (8)$$

where

$$BV\%_{total} = 100 * (BV\%_{wood} / (100 + BV\%_{wood})) \qquad (9)$$

GWT_{wb} = average green weight per cubic foot of combined wood and bark
GWT_w = average green weight per cubic foot of wood
GWT_b = average green weight per cubic foot of bark

Table 1B provides estimates of oven-dry and green weight for combined wood and bark when only wood volume is known.

Comprehensive list of specific gravities

Currently there are 465 trees species listed in the reference species table of the FIA database (FIADB; USDA For. Serv. 2009), and as previously stated, 156 of these species account for more than 95 percent of the tree biomass in the United States. However specific gravity estimates are needed for all 465 species (Table 4) to provide estimates of total tree biomass on U.S. forest land. Specific gravity values for the 309 uncommon species are derived as follows:

> If a tree species is not listed in Table 1A but multiple tree species of the same genus are listed, then the unlisted species is assigned the average of the specific gravities of the listed species of the same genus. There were 142 species in Table 4 that are assigned specific gravities in this manner.
>
> If a tree species is not listed in Table 1A but there is one tree species of the same genus in Table 1A, then the unlisted species is assigned the specific gravity of the listed species. Twenty-seven species in Table 4 are assigned specific gravities in this manner.
>
> If a tree species is not listed in Table 1A and there are no tree species of the same genus listed in Table 1A, then the unlisted tree species is assigned either the average specific gravity of all the softwoods in Table 1A or all the hardwoods in Table 1A. There are 115 species in Table 4 that are assigned specific gravities in this manner.

[1]Biomass usually is reported in oven-dry metric tons. For convenience, tables in this report also include densities for each species in kilograms per cubic meter

Today all trees are identified to species. In earlier inventories trees may have been identified only to genus. To accommodate this older data, the reference species Table 5 contains 25 records where only the genus is listed. An example of this is the record "Fir spp". The specific gravity used for the "Fir spp" record is the average for all of the specific gravities for all of the fir species listed in Table 1A. The specific gravities for the other 24 genus-only records are similarly estimated.

ADDITIONAL INFORMATION

Specific gravity of wood and bark can be quite variable depending on many factors, including the geographic location of trees and moisture content, which varies by species, d.b.h., age, and stem position. The values presented here are averages and should be used with caution for estimates targeting small geographic areas requiring more precise values. Two excellent sources of information on wood and bark, including how to make adjustments for alternative moisture contents and other factors, are found in Bowyer et al. (2007) and Forest Products Laboratory publications (USDA For. Serv. 1999, Simpson 1993). Additional information on individual species characteristics is also available at the PLANTS database (USDA Nat. Res. Conserv. Srvc. 2009).

The tables for this publication are available as electronic worksheets. These worksheets and other information on the FIA program may be found at http://www.fia.fs.fed.us.

LITERATURE CITED

1. Alden, Harry A. 1995. **Hardwoods of North America.** Gen. Tech. Rep. FPL-83. Madison, WI: U.S. Department of Agriculture, Forest Service, Forest Products Laboratory. 136 p.

2. Alden, Harry A. 1997. **Softwoods of North America.** Gen. Tech. Rep. FPL-102. Madison, WI: U.S. Department of Agriculture, Forest Service, Forest Products Laboratory. 151 p.

3. Barger, R.L.; Ffolliott, P.F. 1972. **The physical characteristics and utilization of major woodland tree species in Arizona.** Res. Pap. RM-83. Ft. Collins, CO: U.S. Department of Agriculture, Forest Service, Rocky Mountain Forest and Range Experiment Station. 80 p.

4. Bowyer, J.L.; Shmulsky, R.; Haygreen, J.G. 2007. **Forest products and wood science An introduction.** New York, NY: John Wiley & Sons. 576 p.

5. Clark, A.; Phillips, D.R.; Frederick, D.J. 1985. **Weight, volume, and physical properties of major hardwood species in the Gulf and Atlantic coastal plains.** Res. Pap. SE-250. Asheville, NC: U.S. Department of Agriculture, Forest Service, Southeastern Forest Experiment Station. 66 p.

6. Clark, A.; Phillips, D.R.; Frederick, D.J. 1986. **Weight, volume, and physical properties of major hardwood species in the Piedmont.** Res. Pap. SE-255. Asheville, NC: U.S. Department of Agriculture, Forest Service, Southeastern Forest Experiment Station. 78 p.

7. Einsphar, D.W.; Harder, M. 1976. **Hardwood bark properties important to the manufacture of fiber products.** Forest Products Journal. 26(6): 28-31.

8. Forbes, R.D. 1956. **Forestry handbook.** New York, NY: Ronald Press. 1143 p.

9. Gevorkiantz, S.R.; Olsen, L.P. 1955. **Composite volume tables for timber and their application in the Lake States.** USDA Tech. Bull. No. 1104. Washington, DC: U.S. Department of Agriculture. 51 p.

10. Harkin, J.M.; Rowe, J.W. 1971. **Bark and its possible uses.** Res. Pap. FPL-091. Madison, WI: U.S. Department of Agriculture, Forest Service, Forest Products Laboratory. 56 p.

11. Hilt, D.E.; Rast, E.D.; Bailey, H.J. 1983. **Predicting diameters inside bark for 10 important hardwood species.** Res. Pap. NE-531. Broomall, PA: U.S. Department of Agriculture, Forest Service, Northeastern Forest Experiment Station. 7 p.

12. Isenberg, I.H.; Harder, M.L.; Louden, L. 1980. **Pulpwoods of the United States and Canada: volume I - conifers.** Appleton, WI: Institute of Paper Chemistry. 219 p.

13. Isenberg, I.H.; Harder, M.L.; Louden, L. 1981. **Pulpwoods of the United States and Canada: volume II - conifers.** Appleton, WI: Institute of Paper Chemistry. 168 p.

14. Jenkins, J.; Chojnacky, D.; Heath, L.; Birdsey, R. 2004. **Comprehensive database of diameter-based biomass regressions for North American tree species.** Gen. Tech. Rep. NE-319. Newtown Square, PA: U.S. Department of Agriculture, Forest Service, Northeastern Research Station. 45 p.

15. Koch, P.; Mullen, J.F. 1971. **Thickness and specific gravity of inner and outer bark of red oak and yellow poplar.** Wood Science. 3(4): 214-17.

16. Little, Elbert L., Jr. 1979. **Checklist of United States trees (native and naturalized).** Agric. Handb. 541. Washington, DC: U.S. Department of Agriculture, Forest Service. 375 p.

17. Manwiller , F.G. 1975. **Wood and bark moisture contents of small-diameter hardwoods growing on southern pine sites.** Wood Science. 8(1): 384-388.

18. Markwardt, L.J.; Wilson, T.R.C. 1935. **Strength and related properties of woods grown in the United States.** USDA Tech. Bull. 479. Madison WI: U.S. Department of Agriculture, Forest Service, Forest Products Laboratory. 479 p.

19. McCormack, J.F. 1955. **An allowance for bark increment in computing tree diameter growth for southeastern species.** Stn. Paper SE-60. Asheville, NC: U.S. Department of Agriculture, Forest Service, Southeastern Forest Experiment Station.

20. Simpson, W.T. 1993. **Specific gravity, moisture content, and density relationship for wood.** Gen. Tech. Rep. FPL-76. Madison, WI: U.S. Department of Agriculture, Forest Service, Forest Products Laboratory. 13 p.

21. Smith, J.H.G.; Kozak, A. 1967. **Thickness and percentage of bark of the commercial trees of British Columbia.** Vancouver, BC: Faculty of Forestry, University of British Columbia. 33 p.

22. Smith, J.H.G.; Kozak, A. 1971. **Thickness, moisture content, and specific gravity of inner and outer bark of some Pacific Northwest trees.** Forest Products Journal. 21(2): 38-40.

23. Smith, W.B. 1991. **Assessing removals for North Central forest inventories.** Res. Pap. NC-299. St. Paul, MN: U.S. Dept. of Agriculture, Forest Service, North Central Research Station. 48 p.

24. Stayton, C.L.; Hoffman, M. 1970. **Estimating sugar maple bark thickness and volume.** Res. Pap. NC-38. St. Paul, MN: U.S. Dept. of Agriculture, Forest Service, North Central Forest Experiment Station. 8 p.

25. USDA Forest Service. 1999. **Wood handbook- Wood as an engineering material.** Gen. Tech. Rep. FPL-113. Madison WI: U.S. Department of Agriculture, Forest Service, Forest Products Laboratory. 463 p.

26. USDA Forest Service. 1959. **Ed. No. 7. Volume tables, converting factors and other information applicable to timber in the South.** Atlanta, GA: U.S. Department of Agriculture, Forest Service, Region 8, State and Private Forestry.

27. USDA Forest Service. 2009. **FIA library: database documentation.** Washington, DC: U.S. Department of Agriculture, Forest Service. Available: http://fia.fs.fed.us/library/database-documentation/. [Accessed 2009 Aug. 14].

28. USDA Natural Resources Conservation Service. 2009. **Plants database.** Washington, DC: U.S. Department of Agriculture, Natural Resources Conservation Service. Available: www.plants.usda.gov. [Accessed 2009 Aug. 14].

29. Wenger, Karl F. 1984. **Forestry handbook.** New York, NY: Wiley. 1360 p.

30. Wilson, P.L.; Funck, W.J.; Avery, R.B. 1987. **Fuelwood characteristics of northwestern conifers and hardwoods.** Res. Bul. 60. Corvallis, OR: Oregon State Univ. 42 p.

31. Windsorplywood. http://www.windsorplywood.com/worldofwoods/ [Accessed 2009 Jun. 14].

Table 1A.—Specific gravity and oven-dry weight and green weight of wood and bark for tree species found in North America. Reference numbers in this table refer to numbered citations found in Literature Cited section of this report.

				Specific gravity and oven-dry weight of wood								Average mositure content (MC) and green weight of wood *				Specific gravity and oven-dry weight of bark				Average mositure content (MC) and green weight of bark *				Bark volume	
				12% MC volume basis				Green volume basis								Green volume basis									
Common name	Genus	Species	FIA code	Specific gravity	Reference	Avg. oven-dry weight (lb/cf)	Avg. oven-dry weight (kg/m3)	Specific gravity	Reference	Avg. oven-dry weight (lb/cf)	Avg. oven-dry weight (kg/m3)	Avg. moisture content as a % of oven-dry weight	Reference	Avg. green wt. (lb/cf)	Avg. green wt. (kg/m3)	Specific gravity	Reference	Avg. oven-dry weight (lb/cf)	Avg. oven-dry weight (kg/m3)	Avg. MC as a % of oven-dry weight	Reference	Avg. green wt. (lb/cf)	Avg. green wt. (kg/m3)	Avg. bark volume as % of wood volume	Reference
Pacific silver fir	Abies	amabilis	11	0.43	25	26.8	430	0.40	25	25.0	400	70	30	42	680	0.44	30	27.5	440	64	22	45	721	14.0	30
Balsam fir	Abies	balsamea	12	0.35	25	21.8	350	0.33	25	20.6	330	119	12	45	721	0.40	12	25.0	400	100	2	50	801	12.0	9
White fir	Abies	concolor	15	0.39	25	24.3	390	0.37	25	23.1	370	104	12	47	753	0.56	10	34.9	560	63	22	57	913	12.0	a
Grand fir	Abies	grandis	17	0.37	25	23.1	370	0.35	25	21.8	350	106	12	45	721	0.57	10	35.6	570	63	22	58	929	12.0	a
Subalpine fir	Abies	lasiocarpa	19	0.32	25	20.0	320	0.31	25	19.3	310	45	12	28	448	0.50	10	31.2	500	63	22	51	817	10.8	21
California red fir	Abies	magnifica	20	0.38	25	23.7	380	0.36	25	22.5	360	114	12	48	769	0.44	10	27.5	440	20	30	33	529	10.8	a
Noble fir	Abies	procera	22	0.39	25	24.3	390	0.37	25	23.1	370	30	12	30	481	0.49	10	30.6	490	64	22	50	801	10.8	a
Port-Orford-cedar	Chamaecyparis	lawsoniana	41	0.43	25	26.8	430	0.39	25	24.3	390	74	25	42	678	0.40	30	25.0	400	64	25	41	657	11.6	a
Alaska yellow-cedar	Chamaecyparis	nootkatensis	42	0.44	25	27.5	440	0.42	25	26.2	420	72	29	45	721	0.40	22	25.0	400	112	22	53	849	11.6	21
Atlantic white-cedar	Chamaecyparis	thyoides	43	0.32	25	20.0	320	0.31	25	19.3	310	86	29	36	577	0.40	14	25.0	400	100	30	50	801	11.6	a
Alligator juniper	Juniperus	deppeana	63	0.51	2	31.8	510	0.48	2	30.0	480	34	28	40	641	0.40	b	25.0	400	60	b	40	641	12.0	a
Utah juniper	Juniperus	osteosperma	65	0.72	a	44.9	720	0.68	3	42.6	682	35	b	57	920	0.40	b	25.0	400	60	b	40	641	12.0	a
Southern redcedar	Juniperus	virginiana	67	0.44	2	27.5	440	0.42	2	26.2	420	41	b	37	593	0.40	b	25.0	400	60	b	40	641	12.0	a
Eastern redcedar	Juniperus	virginiana	68	0.47	25	29.3	470	0.44	25	27.5	440	35	29	37	593	0.40	23	25.0	400	60	b	40	641	12.0	23
Tamarack (native)	Larix	laricina	71	0.53	25	33.1	530	0.49	25	30.6	490	54	12	47	753	0.30	23	18.7	300	98	2	37	593	14.0	23
Western larch	Larix	occidentalis	73	0.52	25	32.4	520	0.48	25	30.0	480	60	12	48	769	0.33	12	20.6	330	65	2	34	545	14.0	a
Incense-cedar	Calocedrus	decurrens	81	0.37	25	23.1	370	0.35	25	21.8	350	106	30	45	721	0.25	10	15.6	250	28	30	20	320	17.0	a
Engelmann spruce	Picea	engelmannii	93	0.35	25	21.8	350	0.33	25	20.6	330	89	12	39	625	0.51	12	31.8	510	79	2	57	913	11.2	21
White spruce	Picea	glauca	94	0.40	25	25.0	400	0.37	25	23.1	370	52	12	35	561	0.39	12	24.3	390	77	30	43	689	13.0	a
Black spruce	Picea	mariana	95	0.46	25	28.7	460	0.38	25	23.7	380	48	12	35	561	0.42	12	26.2	420	91	2	50	801	13.0	a
Red spruce	Picea	rubens	97	0.40	25	25.0	400	0.37	25	23.1	370	47	12	34	545	0.32	14	20.0	320	75	b	35	561	13.0	a
Sitka spruce	Picea	sitchensis	98	0.36	25	22.5	360	0.33	25	20.6	330	60	12	33	529	0.55	10	34.3	550	81	22	62	993	12.5	21
Knobcone pine	Pinus	attenuata	103	0.42	a	26.2	420	0.39	30	24.3	390	105	b	50	801	0.38	30	23.7	380	69	b	40	641	12.0	30
Jack pine	Pinus	banksiana	105	0.43	25	26.8	430	0.40	25	25.0	400	100	12	50	801	0.41	12	25.6	410	92	2	49	785	14.0	9
Common or two-needle pinyon	Pinus	edulis	106	0.57	2	35.6	570	0.50	2	31.2	500	28	b	40	641	0.40	b	25.0	400	60	b	40	641	13.4	a
Sand pine	Pinus	clausa	107	0.48	25	30.0	480	0.46	25	28.7	460	32	12	38	609	0.45	14	28.1	450	89	2	53	849	15.0	26
Lodgepole pine	Pinus	contorta	108	0.41	25	25.6	410	0.38	25	23.7	380	64	12	39	625	0.38	12	23.7	380	64	2	39	625	8.9	21
Shortleaf pine	Pinus	echinata	110	0.51	25	31.8	510	0.47	25	29.3	470	77	12	52	833	0.35	12	21.8	350	60	b	35	561	16.0	26
Slash pine	Pinus	elliottii	111	0.59	25	36.8	590	0.54	25	33.7	540	72	12	58	929	0.35	12	21.8	350	88	2	41	657	18.0	26
Limber pine	Pinus	flexilis	113	0.42	2	26.2	420	0.37	2	23.1	370	95	b	45	721	0.50	b	31.2	500	12	b	35	561	13.4	a
Spruce pine	Pinus	glabra	115	0.44	25	27.5	440	0.41	25	25.6	410	76	b	45	721	0.45	14	28.1	450	25	b	35	561	13.4	a
Jeffrey pine	Pinus	jeffreyi	116	0.42	25	26.2	420	0.37	30	23.1	370	104	12	47	753	0.36	30	22.5	360	34	2	30	481	25.6	a
Sugar pine	Pinus	lambertiana	117	0.36	25	22.5	360	0.34	25	21.2	340	130	25	49	782	0.35	10	21.8	350	88	2	41	657	25.6	a

Table 1A.—continued

				Specific gravity and oven-dry weight of wood								Average mositure content (MC) and green weight of wood *				Specific gravity and oven-dry weight of bark				Average mositure content (MC) and green weight of bark *				Bark volume	
				12% MC volume basis				Green volume basis								Green volume basis									
Common name	Genus	Species	FIA code	*Specific gravity*	Reference	*Avg. oven-dry weight (lb/cf)*	*Avg. oven-dry weight (kg/m3)*	*Specific gravity*	Reference	*Avg. oven-dry weight (lb/cf)*	*Avg. oven-dry weight (kg/m3)*	*Avg. moisture content as a % of oven-dry weight*	Reference	*Avg. green wt. (lb/cf)*	*Avg. green wt. (kg/m3)*	*Specific gravity*	Reference	*Avg. oven-dry weight (lb/cf)*	*Avg. oven-dry weight (kg/m3)*	*Avg. MC as a % of oven-dry weight*	Reference	*Avg. green wt. (lb/cf)*	*Avg. green wt. (kg/m3)*	*Avg. bark volume as % of wood volume*	Reference
Western white pine	Pinus	monticola	119	0.38	25	23.7	380	0.36	25	22.5	360	56	12	35	561	0.47	12	29.3	470	84	22	54	865	12.6	21
Bishop pine	Pinus	muricata	120	0.49	a	30.6	490	0.45	30	28.1	450	60	b	45	721	0.45	31	28.1	450	42	b	40	641	22.0	a
Longleaf pine	Pinus	palustris	121	0.59	25	36.8	590	0.54	25	33.7	540	63	12	55	881	0.45	12	28.1	450	89	2	53	849	14.0	26
Ponderosa pine	Pinus	ponderosa	122	0.40	25	25.0	400	0.38	25	23.7	380	90	12	45	721	0.35	12	21.8	350	33	2	29	464	25.6	21
Table Mountain pine	Pinus	pungens	123	0.52	2	32.4	520	0.49	2	30.6	490	77	b	54	865	0.45	b	28.1	450	60	b	45	721	13.4	a
Monterey pine	Pinus	radiata	124	0.53	2	33.0	529	0.40	2	25.0	400	100	b	50	801	0.40	b	25.0	400	60	b	40	641	13.4	a
Red pine	Pinus	resinosa	125	0.46	25	28.7	460	0.41	25	25.6	410	64	12	42	673	0.27	12	16.8	270	90	b	32	513	16.0	23
Pitch pine	Pinus	rigida	126	0.52	25	32.4	520	0.47	25	29.3	470	70	12	50	801	0.34	10	21.2	340	89	2	40	641	13.4	a
Gray or California foothill pine	Pinus	sabiniana	127	0.43	a	26.8	430	0.40	30	25.0	400	80	b	45	721	0.40	30	25.0	400	60	b	40	641	22.0	30
Pond pine	Pinus	serotina	128	0.56	25	34.9	560	0.51	25	31.8	510	60	b	51	817	0.33	b	20.6	330	94	b	40	641	13.4	a
Eastern white pine	Pinus	strobus	129	0.35	25	21.8	350	0.34	25	21.2	340	65	b	35	561	0.47	12	29.3	470	70	b	50	801	16.0	9
Loblolly pine	Pinus	taeda	131	0.51	25	31.8	510	0.47	25	29.3	470	81	12	53	849	0.33	12	20.6	330	99	2	41	657	16.6	19
Virginia pine	Pinus	virginiana	132	0.48	25	30.0	480	0.45	25	28.1	450	78	b	50	801	0.54	12	33.7	540	90	b	64	1025	13.4	a
Douglas-fir	Pseudotsuga	menziesii	202	0.48	25	30.0	480	0.45	25	28.1	450	35	12	38	609	0.44	26	27.5	440	89	2	52	833	17.3	21
Redwood	Sequoia	sempervirens	211	0.38	25	23.7	380	0.36	25	22.5	360	123	12	50	801	0.43	10	26.8	430	16	30	31	497	18.0	30
Giant sequoia	Sequoiadendron	giganteum	212	0.38	a	23.7	380	0.34	30	21.2	340	178	30	59	945	0.34	30	21.2	340	18	b	25	400	18.0	a
Baldcypress	Taxodium	distichum	221	0.46	25	28.7	460	0.42	25	26.2	420	95	29	51	817	0.50	10	31.2	500	99	b	62	993	20.0	26
Pacific yew	Taxus	brevifolia	231	0.67	2	41.8	670	0.60	2	37.4	600	47	30	55	881	0.59	30	36.8	590	98	30	73	1169	4.0	30
Northern white-cedar	Thuja	occidentalis	241	0.31	25	19.3	310	0.29	25	18.1	290	99	23	36	577	0.42	10	26.2	420	91	b	50	801	14.0	9
Western redcedar	Thuja	plicata	242	0.32	25	20.0	320	0.31	25	19.3	310	40	12	27	433	0.37	12	23.1	370	56	22	36	577	10.6	21
Eastern hemlock	Tsuga	canadensis	261	0.40	25	25.0	400	0.38	25	23.7	380	111	12	50	801	0.46	10	28.7	460	99	2	57	913	17.0	9
Western hemlock	Tsuga	heterophylla	263	0.45	25	28.1	450	0.42	25	26.2	420	56	12	41	657	0.50	26	31.2	500	102	22	63	1009	15.8	21
Mountain hemlock	Tsuga	mertensiana	264	0.45	25	28.1	450	0.42	25	26.2	420	68	12	44	705	0.41	10	25.6	410	92	b	49	785	15.8	a
Bigleaf maple	Acer	macrophyllum	312	0.48	25	30.0	480	0.44	25	27.5	440	71	13	47	753	0.48	10	30.0	480	114	22	64	1025	10.0	30
Boxelder	Acer	negundo	313	0.46	31	28.7	460	0.42	31	26.2	420	91	b	50	801	0.50	b	31.2	500	92	b	60	961	8.6	a
Black maple	Acer	nigrum	314	0.57	25	35.6	570	0.52	25	32.4	520	70	b	55	881	0.54	14	33.7	540	90	1	64	1025	15.6	a
Striped maple	Acer	pensylvanicum	315	0.46	1	28.7	460	0.44	1	27.5	440	71	b	47	753	0.50	b	31.2	500	89	b	59	945	8.6	a
Red maple	Acer	rubrum	316	0.54	25	33.7	540	0.49	25	30.6	490	64	13	50	801	0.60	13	37.4	600	79	1	67	1073	8.6	11
Silver maple	Acer	saccharinum	317	0.47	25	29.3	470	0.44	25	27.5	440	68	13	46	737	0.57	13	35.6	570	80	b	64	1025	8.6	a
Sugar maple	Acer	saccharum	318	0.63	25	39.3	630	0.56	25	34.9	560	57	13	55	881	0.54	13	33.7	540	90	1	64	1025	15.6	11
Yellow buckeye	Aesculus	flava	332	0.36	1	22.5	360	0.33	1	20.6	330	143	13	50	801	0.50	b	31.2	500	89	b	59	945	15.0	a
Ailanthus	Ailanthus	altissima	341	0.53	1	33.1	530	0.46	1	28.7	460	74	b	50	801	0.45	b	28.1	450	78	b	50	801	15.0	a
Red alder	Alnus	rubra	351	0.41	25	25.6	410	0.37	25	23.1	370	99	13	46	737	0.56	26	34.9	560	75	22	61	977	12.0	21
Serviceberry spp.	Amelanchier	spp.	356	0.79	1	49.3	790	0.66	1	41.2	660	48	26	61	977	0.50	b	31.2	500	60	b	50	801	8.6	a

Table 1A.—continued

				Specific gravity and oven-dry weight of wood								Average mositure content (MC) and green weight of wood *				Specific gravity and oven-dry weight of bark				Average mositure content (MC) and green weight of bark *				Bark volume	
				12% MC volume basis				Green volume basis								Green volume basis									
Common name	Genus	Species	FIA code	*Specific gravity*	Reference	*Avg. oven-dry weight (lb/cf)*	*Avg. oven-dry weight (kg/m3)*	*Specific gravity*	Reference	*Avg. oven-dry weight (lb/cf)*	*Avg. oven-dry weight (kg/m3)*	*Avg. moisture content as a % of oven-dry weight*	Reference	*Avg. green wt. (lb/cf)*	*Avg. green wt. (kg/m3)*	*Specific gravity*	Reference	*Avg. oven-dry weight (lb/cf)*	*Avg. oven-dry weight (kg/m3)*	*Avg. MC as a % of oven-dry weight*	Reference	*Avg. green wt. (lb/cf)*	*Avg. green wt. (kg/m3)*	*Avg. bark volume as % of wood volume*	Reference
Pacific madrone	Arbutus	menziesii	361	0.65	1	40.6	650	0.58	1	36.2	580	66	26	60	961	0.60	25	37.4	600	60	b	60	961	15.0	a
Yellow birch	Betula	alleghaniensis	371	0.62	25	38.7	620	0.55	25	34.3	550	72	25	59	945	0.62	10	38.7	620	60	b	62	993	9.8	21
Sweet birch	Betula	lenta	372	0.65	25	40.6	650	0.60	25	37.4	600	73	25	65	1038	0.62	b	38.7	620	53	1	59	945	9.8	a
River birch	Betula	nigra	373	0.56	1	34.9	560	0.49	1	30.6	490	86	b	57	913	0.55	b	34.3	550	46	b	50	801	9.8	a
Paper birch	Betula	papyrifera	375	0.55	25	34.3	550	0.48	25	30.0	480	74	25	52	833	0.56	13	34.9	560	52	22	53	849	12.6	c
Gray birch	Betula	populifolia	379	0.51	1	31.8	510	0.45	1	28.1	450	64	13	46	737	0.55	b	34.3	550	63	1	56	897	12.6	a
American hornbeam, musclewood	Carpinus	caroliniana	391	0.70	1	43.7	700	0.58	1	36.2	580	46	26	53	849	0.55	b	34.3	550	89	b	65	1041	8.6	a
Water hickory	Carya	aquatica	401	0.62	25	38.7	620	0.61	25	38.1	610	84	25	70	1121	0.60	14	37.4	600	60	b	60	961	16.0	a
Bitternut hickory	Carya	cordiformis	402	0.66	25	41.2	660	0.60	25	37.4	600	71	25	64	1025	0.60	23	37.4	600	60	b	60	961	16.0	a
Pignut hickory	Carya	glabra	403	0.75	25	46.8	750	0.66	25	41.2	660	65	13	68	1089	0.60	23	37.4	600	60	b	60	961	16.0	a
Pecan	Carya	illinoinensis	404	0.66	25	41.2	660	0.60	25	37.4	600	66	25	62	993	0.60	14	37.4	600	60	b	60	961	16.0	a
Shellbark hickory	Carya	laciniosa	405	0.69	25	43.1	690	0.62	25	38.7	620	65	13	64	1025	0.60	14	37.4	600	60	b	60	961	16.0	a
Nutmeg hickory	Carya	myristiciformis	406	0.60	25	37.4	600	0.56	25	34.9	560	77	29	62	993	0.60	14	37.4	600	60	b	60	961	16.0	a
Shagbark hickory	Carya	ovata	407	0.72	25	44.9	720	0.64	25	39.9	640	60	13	64	1025	0.72	13	44.9	720	34	b	60	961	16.0	a
Mockernut hickory	Carya	alba	409	0.72	25	44.9	720	0.64	25	39.9	640	63	25	65	1041	0.60	23	37.4	600	60	b	60	961	16.0	a
American chestnut	Castanea	dentata	421	0.43	25	26.8	430	0.40	25	25.0	400	120	25	55	881	0.50	14	31.2	500	89	b	59	945	15.0	a
Giant chinkapin, golden chinkapin	Chrysolepis	chrysophylla	431	0.46	1	28.7	460	0.42	1	26.2	420	133	26	61	977	0.42	30	26.2	420	91	b	50	801	12.0	30
Northern catalpa	Catalpa	speciosa	452	0.41	1	25.6	410	0.38	1	23.7	380	73	26	41	657	0.50	b	31.2	500	89	b	59	945	15.0	a
Hackberry	Celtis	occidentalis	462	0.53	25	33.1	530	0.49	25	30.6	490	64	29	50	801	0.49	23	30.6	490	90	b	58	929	15.0	23
Flowering dogwood	Cornus	florida	491	0.73	1	45.6	730	0.64	1	39.9	640	33	b	53	849	0.58	b	36.2	580	91	b	69	1105	15.0	a
Pacific dogwood	Cornus	nuttallii	492	0.62	a	38.7	620	0.58	18	36.2	580	46	b	53	849	0.58	18	36.2	580	91	b	69	1105	15.0	a
Common persimmon	Diospyros	virginiana	521	0.74	26	46.2	740	0.64	26	39.9	640	58	26	63	1009	0.50	b	31.2	500	89	b	59	945	15.0	a
American beech	Fagus	grandifolia	531	0.64	25	39.9	640	0.56	25	34.9	560	55	13	54	865	0.67	13	41.8	670	89	b	79	1265	6.0	11
White ash	Fraxinus	americana	541	0.60	25	37.4	600	0.55	25	34.3	550	46	25	50	801	0.50	13	31.2	500	89	b	59	945	16.0	a
Oregon ash	Fraxinus	latifolia	542	0.55	25	34.3	550	0.50	25	31.2	500	60	b	50	801	0.50	14	31.2	500	89	b	59	945	16.0	a
Black ash	Fraxinus	nigra	543	0.49	25	30.6	490	0.45	25	28.1	450	85	13	52	833	0.43	10	26.8	430	90	b	51	817	16.0	a
Green ash	Fraxinus	pennsylvanica	544	0.56	25	34.9	560	0.53	25	33.1	530	57	29	52	833	0.48	13	30.0	480	70	5	51	817	16.0	26
Pumpkin ash	Fraxinus	profunda	545	0.52	1	32.4	520	0.48	1	30.0	480	67	b	50	801	0.45	b	28.1	450	89	b	53	849	16.0	a
Blue ash	Fraxinus	quadrangulata	546	0.58	25	36.2	580	0.53	25	33.1	530	51	b	50	801	0.39	14	24.3	390	89	b	46	737	16.0	a
Honeylocust	Gleditsia	triacanthos	552	0.65	a	40.6	650	0.60	25	37.4	600	60	26	60	961	0.50	14	31.2	500	89	b	59	945	15.0	a
Kentucky coffeetree	Gymnocladus	dioicus	571	0.60	1	37.4	600	0.53	1	33.1	530	51	b	50	801	0.50	b	31.2	500	60	b	50	801	15.0	a
Silverbell spp.	Halesia	spp.	580	0.45	1	28.1	450	0.42	1	26.2	420	68	26	44	705	0.50	b	31.2	500	89	b	59	945	15.0	a
American holly	Ilex	opaca	591	0.57	1	35.6	570	0.50	1	31.2	500	83	26	57	913	0.50	b	31.2	500	89	b	59	945	15.0	a

Table 1A.—continued

				Specific gravity and oven-dry weight of wood								Average mositure content (MC) and green weight of wood *				Specific gravity and oven-dry weight of bark				Average mositure content (MC) and green weight of bark *				Bark volume	
				12% MC volume basis				Green volume basis								Green volume basis									
Common name	Genus	Species	FIA code	Specific gravity	Reference	Avg. oven-dry weight (lb/cf)	Avg. oven-dry weight (kg/m3)	Specific gravity	Reference	Avg. oven-dry weight (lb/cf)	Avg. oven-dry weight (kg/m3)	Avg. moisture content as a % of oven-dry weight	Reference	Avg. green wt. (lb/cf)	Avg. green wt. (kg/m3)	Specific gravity	Reference	Avg. oven-dry weight (lb/cf)	Avg. oven-dry weight (kg/m3)	Avg. MC as a % of oven-dry weight	Reference	Avg. green wt. (lb/cf)	Avg. green wt. (kg/m3)	Avg. bark volume as % of wood volume	Reference
Butternut	Juglans	cinerea	601	0.38	25	23.7	380	0.36	25	22.5	360	105	13	46	737	0.40	14	25.0	400	88	b	47	753	15.0	a
Black walnut	Juglans	nigra	602	0.55	25	34.3	550	0.51	25	31.8	510	79	13	57	913	0.33	10	20.6	330	89	b	39	625	15.0	a
Sweetgum	Liquidambar	styraciflua	611	0.52	25	32.4	520	0.46	25	28.7	460	74	13	50	801	0.42	13	26.2	420	91	1	50	801	15.0	5
Yellow-poplar	Liriodendron	tulipifera	621	0.42	25	26.2	420	0.40	25	25.0	400	95	25	49	780	0.38	13	23.7	380	124	5	53	849	18.0	5
Tanoak	Lithocarpus	densiflorus	631	0.62	a	38.7	620	0.58	25	36.2	580	80	26	65	1041	0.62	10	38.7	620	60	b	62	993	19.0	30
Osage-orange	Maclura	pomifera	641	0.85	1	53.0	850	0.76	1	47.4	760	31	26	62	993	0.60	b	37.4	600	60	b	60	961	15.0	a
Cucumbertree	Magnolia	acuminata	651	0.48	25	30.0	480	0.44	25	27.5	440	78	13	49	785	0.44	14	27.5	440	89	b	52	833	15.0	a
Southern magnolia	Magnolia	grandiflora	652	0.50	25	31.2	500	0.46	25	28.7	460	106	13	59	945	0.44	14	27.5	440	89	b	52	833	15.0	a
Sweetbay	Magnolia	virginiana	653	0.46	1	28.7	460	0.42	1	26.2	420	87	b	49	785	0.44	b	27.5	440	104	1	56	897	15.0	a
Mountain or Fraser magnolia	Magnolia	fraseri	655	0.44	1	27.5	440	0.40	1	25.0	400	96	b	49	785	0.44	b	27.5	440	89	b	52	833	15.0	a
Apple spp.	Malus	spp.	660	0.67	26	41.8	670	0.61	26	38.1	610	78	25	68	1085	0.50	b	31.2	500	70	b	53	849	15.0	a
Water tupelo	Nyssa	aquatica	691	0.50	25	31.2	500	0.46	25	28.7	460	95	29	56	897	0.58	10	36.2	580	82	1	66	1057	14.0	a
Blackgum	Nyssa	sylvatica	693	0.50	25	31.2	500	0.46	25	28.7	460	101	25	58	924	0.44	13	27.5	440	71	1	47	753	14.0	26
Eastern hophornbeam	Ostrya	virginiana	701	0.70	1	43.7	700	0.63	1	39.3	630	53	26	60	961	0.50	b	31.2	500	89	b	59	945	15.0	a
Sourwood	Oxydendrum	arboreum	711	0.55	1	34.3	550	0.50	18	31.2	500	70	26	53	849	0.60	b	37.4	600	60	b	60	961	15.0	a
American sycamore	Platanus	occidentalis	731	0.49	25	30.6	490	0.46	25	28.7	460	81	13	52	833	0.60	13	37.4	600	84	6	69	1105	8.0	23
Balsam poplar	Populus	balsamifera	741	0.34	25	21.2	340	0.31	25	19.3	310	107	13	40	641	0.50	23	31.2	500	86	1	58	929	22.0	a
Eastern cottonwood	Populus	deltoides	742	0.40	25	25.0	400	0.37	25	23.1	370	117	29	50	801	0.38	13	23.7	380	56	b	37	593	22.0	30
Bigtooth aspen	Populus	grandidentata	743	0.39	25	24.3	390	0.36	25	22.5	360	91	13	43	689	0.59	10	36.8	590	90	b	70	1121	14.4	a
Quaking aspen	Populus	tremuloides	746	0.38	25	23.7	380	0.35	25	21.8	350	129	29	50	801	0.50	13	31.2	500	102	22	63	1009	14.4	21
Black cottonwood	Populus	balsamifera	747	0.35	25	21.8	350	0.31	25	19.3	310	138	13	46	737	0.40	13	25.0	400	100	31	50	801	16.3	21
Fremont cottonwood	Populus	fremontii	748	0.45	a	28.1	450	0.41	30	25.6	410	56	b	40	641	0.41	30	25.6	410	92	b	49	785	22.0	a
Mesquite spp.	Prosopis	spp.	755	0.82	1	51.2	820	0.78	31	48.7	780	21	b	59	945	0.65	b	40.6	650	41	b	57	913	15.0	a
Blackcherry	Prunus	serotina	762	0.50	25	31.2	500	0.47	25	29.3	470	53	29	45	721	0.63	10	39.3	630	91	b	75	1201	9.2	11
White oak	Quercus	alba	802	0.68	25	42.4	680	0.60	25	37.4	600	68	13	63	1009	0.56	13	34.9	560	89	17	66	1057	16.0	5
Swamp white oak	Quercus	bicolor	804	0.72	25	44.9	720	0.64	25	39.9	640	58	13	63	1009	0.55	b	34.3	550	89	b	65	1041	16.0	a
Canyon live oak	Quercus	chrysolepis	805	0.74	a	46.2	740	0.70	30	43.7	700	74	13	76	1217	0.64	14	39.9	640	90	b	76	1217	16.0	a
Scarlet oak	Quercus	coccinea	806	0.67	25	41.8	670	0.60	25	37.4	600	71	13	64	1025	0.71	10	44.3	710	49	6	66	1057	22.0	a
Southern red oak	Quercus	falcata	812	0.59	25	36.8	590	0.52	25	32.4	520	97	13	64	1025	0.68	10	42.4	680	48	6	63	1009	22.0	5
Cherrybark oak	Quercus	pagoda	813	0.69	25	43.1	690	0.61	25	38.1	610	68	13	64	1025	0.63	14	39.3	630	91	17	75	1201	22.0	a
Gambel oak	Quercus	gambelii	814	0.63	a	39.3	630	0.61	3	38.1	610	66	13	63	1009	0.63	b	39.2	629	66	b	65	1041	22.0	a
Oregon white oak	Quercus	garryana	815	0.72	1	44.9	720	0.64	1	39.9	640	58	13	63	1009	0.63	30	39.3	630	65	b	65	1041	16.0	a
California black oak	Quercus	kelloggii	818	0.55	a	34.3	550	0.51	18	31.8	510	101	13	64	1025	0.45	14	28.1	450	89	b	53	849	22.0	a
Laurel oak	Quercus	laurifolia	820	0.63	25	39.3	630	0.56	25	34.9	560	83	13	64	1025	0.50	b	31.2	500	121	5	69	1105	16.0	a

Table 1A.—continued

				Specific gravity and oven-dry weight of wood								Average mositure content (MC) and green weight of wood *				Specific gravity and oven-dry weight of bark				Average mositure content (MC) and green weight of bark *				Bark volume	
				12% MC volume basis				Green volume basis								Green volume basis									
Common name	Genus	Species	FIA code	Specific gravity	Reference	Avg. oven-dry weight (lb/cf)	Avg. oven-dry weight (kg/m3)	Specific gravity	Reference	Avg. oven-dry weight (lb/cf)	Avg. oven-dry weight (kg/m3)	Avg. moisture content as a % of oven-dry weight	Reference	Avg. green wt. (lb/cf)	Avg. green wt. (kg/m3)	Specific gravity	Reference	Avg. oven-dry weight (lb/cf)	Avg. oven-dry weight (kg/m3)	Avg. MC as a % of oven-dry weight	Reference	Avg. green wt. (lb/cf)	Avg. green wt. (kg/m3)	Avg. bark volume as % of wood volume	Reference
California white oak	Quercus	lobata	821	0.58	a	36.2	580	0.55	30	34.3	550	84	13	63	1009	0.55	30	34.3	550	89	b	65	1041	16.0	a
Overcup oak	Quercus	lyrata	822	0.63	25	39.3	630	0.57	25	35.6	570	77	13	63	1009	0.51	14	31.8	510	89	b	60	961	22.0	a
Bur oak	Quercus	macrocarpa	823	0.64	25	39.9	640	0.58	25	36.2	580	74	13	63	1009	0.54	10	33.7	540	90	b	64	1025	16.0	a
Swamp chestnut oak	Quercus	michauxii	825	0.67	25	41.8	670	0.60	25	37.4	600	68	13	63	1009	0.51	14	31.8	510	89	b	60	961	23.0	a
Water oak	Quercus	nigra	827	0.63	25	39.3	630	0.56	25	34.9	560	83	13	64	1025	0.62	14	38.7	620	73	5	67	1073	16.0	a
Pin oak	Quercus	palustris	830	0.63	25	39.3	630	0.58	25	36.2	580	77	13	64	1025	0.60	14	37.4	600	90	17	71	1137	22.0	a
Willow oak	Quercus	phellos	831	0.69	25	43.1	690	0.56	25	34.9	560	83	13	64	1025	0.59	10	36.8	590	90	b	70	1121	16.0	a
Chestnut oak	Quercus	prinus	832	0.66	25	41.2	660	0.57	25	35.6	570	77	13	63	1009	0.54	10	33.7	540	60	6	54	865	23.0	5
Northern red oak	Quercus	rubra	833	0.63	25	39.3	630	0.56	25	34.9	560	83	13	64	1025	0.68	13	42.4	680	91	17	81	1298	20.0	9
Post oak	Quercus	stellata	835	0.67	25	41.8	670	0.60	25	37.4	600	71	13	64	1025	0.51	10	31.8	510	89	17	60	961	22.0	a
Black oak	Quercus	velutina	837	0.61	25	38.1	610	0.56	25	34.9	560	83	13	64	1025	0.60	10	37.4	600	90	17	71	1137	18.5	11
Live oak	Quercus	virginiana	838	0.88	25	54.9	880	0.80	25	49.9	800	52	13	76	1217	0.51	14	31.8	510	89	b	60	961	16.0	a
Black locust	Robinia	pseudoacacia	901	0.69	25	43.1	690	0.66	25	41.2	660	41	26	58	929	0.29	10	18.1	290	88	b	34	545	15.0	a
Black willow	Salix	nigra	922	0.39	25	24.3	390	0.36	25	22.5	360	127	13	51	817	0.50	14	31.2	500	99	1	62	993	16.0	a
Sassafras	Sassafras	albidum	931	0.46	25	28.7	460	0.42	25	26.2	420	68	26	44	705	0.50	14	31.2	500	89	b	59	945	15.0	a
American basswood	Tilia	americana	951	0.37	25	23.1	370	0.32	25	20.0	320	105	25	41	657	0.48	10	30.0	480	90	b	57	913	10.5	c
Winged elm	Ulmus	alata	971	0.66	1	41.2	660	0.60	1	37.4	600	42	b	53	849	0.45	b	28.1	450	75	b	49	785	14.0	a
American elm	Ulmus	americana	972	0.50	25	31.2	500	0.46	25	28.7	460	94	25	56	892	0.44	10	27.5	440	78	b	49	785	14.0	a
Cedar elm	Ulmus	crassifolia	973	0.64	1	39.9	640	0.59	1	36.8	590	66	25	61	977	0.45	b	28.1	450	75	b	49	785	14.0	a
Slippery elm	Ulmus	rubra	975	0.53	25	33.1	530	0.48	25	30.0	480	77	b	53	849	0.29	10	18.1	290	171	b	49	785	14.0	a
Rock elm	Ulmus	thomasii	977	0.63	25	39.3	630	0.57	25	35.6	570	51	25	54	860	0.50	14	31.2	500	57	b	49	785	14.0	a
California-laurel	Umbellularia	californica	981	0.55	1	34.3	550	0.51	1	31.8	510	67	30	53	849	0.55	30	34.3	550	43	b	49	785	15.0	a

* Moisture content is extremely variable and the values shown are averages or estimates based on the literature cited.

a No reference source available, estimated based on similar species.

b Based on green volume specific gravity and bark moisture content of similar species.

c Adapted from McCormack (1955) using supplemental data from Forbes (1956) and Koch (1971)

Table 1B.—Average oven-dry and green weight of wood and bark when only wood volume is known for tree species in North America. Reference numbers in this table refer to numbered citations found in Literature Cited section of this report.

Common name	Genus	Species	FIA code	Wood reference	Bark reference	Bark % reference (Tab 3)	Total oven-dry and green weight of wood and bark per cubic foot of wood *			
							Avg. oven-dry weight of wood and bark (lb/cf)	*Avg. oven-dry weight of wood and bark (kg/m3)*	*Avg. green weight of wood and bark (lb/cf)*	*Avg. green weight of wood and bark (kg/m3)*
Pacific silver fir	Abies	amabilis	11	25	30	30	29	461	49	781
Balsam fir	Abies	balsamea	12	25	12	9	24	378	51	817
White fir	Abies	concolor	15	25	12	a	27	437	54	862
Grand fir	Abies	grandis	17	25	12	a	26	418	52	832
Subalpine fir	Abies	lasiocarpa	19	25	12	21	23	364	33	536
California red fir	Abies	magnifica	20	25	12	a	25	407	52	826
Noble fir	Abies	procera	22	25	12	a	26	423	35	567
Port-Orford-cedar	Chamaecyparis	lawsoniana	41	25	25	a	27	436	47	754
Alaska yellow-cedar	Chamaecyparis	nootkatensis	42	25	29	21	29	466	51	819
Atlantic white-cedar	Chamaecyparis	thyoides	43	25	29	a	22	356	42	669
Alligator juniper	Juniperus	deppeana	63	2	28	a	33	528	45	718
Utah juniper	Juniperus	osteosperma	65	3	b	a	46	730	62	997
Southern redcedar	Juniperus	virginiana	67	2	b	a	29	468	42	670
Eastern redcedar	Juniperus	virginiana	68	25	29	23	30	488	42	670
Tamarack (native)	Larix	laricina	71	25	12	23	33	532	52	836
Western larch	Larix	occidentalis	73	25	12	a	33	526	53	845
Incense-cedar	Calocedrus	decurrens	81	25	30	a	24	392	48	775
Engelmann spruce	Picea	engelmannii	93	25	12	21	24	387	45	727
White spruce	Picea	glauca	94	25	12	a	26	421	41	650
Black spruce	Picea	mariana	95	25	12	a	27	434	42	665
Red spruce	Picea	rubens	97	25	12	a	26	411	39	618
Sitka spruce	Picea	sitchensis	98	25	12	21	25	399	41	653
Knobcone pine	Pinus	attenuata	103	30	b	30	27	435	55	878
Jack pine	Pinus	banksiana	105	25	12	9	29	457	57	911
Common or two-needle pinyon	Pinus	edulis	106	2	b	a	35	553	45	727
Sand pine	Pinus	clausa	107	25	12	26	33	527	46	736
Lodgepole pine	Pinus	contorta	108	25	12	21	26	413	42	680
Shortleaf pine	Pinus	echinata	110	25	12	26	33	526	58	923
Slash pine	Pinus	elliottii	111	25	12	26	38	603	65	1047
Limber pine	Pinus	flexilis	113	2	b	a	27	437	50	796
Spruce pine	Pinus	glabra	115	25	b	a	29	470	50	796
Jeffrey pine	Pinus	jeffreyi	116	30	12	a	29	462	55	876
Sugar pine	Pinus	lambertiana	117	25	25	a	27	429	59	950
Western white pine	Pinus	monticola	119	25	12	21	26	419	42	670
Bishop pine	Pinus	muricata	120	30	b	a	34	549	54	862
Longleaf pine	Pinus	palustris	121	25	12	26	38	603	62	1000
Ponderosa pine	Pinus	ponderosa	122	25	12	21	29	469	52	840
Table Mountain pine	Pinus	pungens	123	2	b	a	34	550	60	962
Monterey pine	Pinus	radiata	124	2	b	a	28	454	55	887
Red pine	Pinus	resinosa	125	25	12	23	28	453	47	755
Pitch pine	Pinus	rigida	126	25	12	a	32	515	55	887

Table 1B.—continued

Common name	Genus	Species	FIA code	Wood reference	Bark reference	Bark % reference (Tab 3)	Total oven-dry and green weight of wood and bark per cubic foot of wood * Avg. oven-dry weight of wood and bark (lb/cf)	*Avg. oven-dry weight of wood and bark (kg/m3)*	*Avg. green weight of wood and bark (lb/cf)*	*Avg. green weight of wood and bark (kg/m3)*
Gray or California foothill pine	Pinus	sabiniana	127	30	b	30	30	488	54	862
Pond pine	Pinus	serotina	128	25	b	a	35	554	56	903
Eastern white pine	Pinus	strobus	129	25	b	9	26	415	43	689
Loblolly pine	Pinus	taeda	131	25	12	19	33	525	60	958
Virginia pine	Pinus	virginiana	132	25	b	a	33	522	59	938
Douglas-fir	Pseudotsuga	menziesii	202	25	12	21	33	526	47	753
Redwood	Sequoia	sempervirens	211	25	12	30	27	437	56	890
Giant sequoia	Sequoiadendron	giganteum	212	30	30	a	25	401	63	1017
Baldcypress	Taxodium	distichum	221	25	29	26	32	520	63	1016
Pacific yew	Taxus	brevifolia	231	2	30	30	39	623	58	928
Northern white-cedar	Thuja	occidentalis	241	25	23	9	22	349	43	689
Western redcedar	Thuja	plicata	242	25	12	21	22	349	31	493
Eastern hemlock	Tsuga	canadensis	261	25	12	9	29	458	60	956
Western hemlock	Tsuga	heterophylla	263	25	12	21	31	499	51	816
Mountain hemlock	Tsuga	mertensiana	264	25	12	a	30	484	52	829
Bigleaf maple	Acer	macrophyllum	312	25	13	30	30	488	53	855
Boxelder	Acer	negundo	313	31	b	a	29	463	55	884
Black maple	Acer	nigrum	314	25	b	a	38	604	65	1041
Striped maple	Acer	pensylvanicum	315	1	b	a	30	483	52	834
Red maple	Acer	rubrum	316	25	13	11	34	541	56	893
Silver maple	Acer	saccharinum	317	25	13	a	31	489	52	825
Sugar maple	Acer	saccharum	318	25	13	11	40	644	65	1041
Yellow buckeye	Aesculus	flava	332	1	13	a	25	405	59	943
Ailanthus	Ailanthus	altissima	341	1	b	a	33	527	58	921
Red alder	Alnus	rubra	351	25	13	21	27	437	53	855
Serviceberry spp.	Amelanchier	spp.	356	1	26	a	44	703	65	1046
Pacific madrone	Arbutus	menziesii	361	1	26	a	42	670	69	1105
Yellow birch	Betula	alleghaniensis	371	25	25	21	38	611	65	1043
Sweet birch	Betula	lenta	372	25	25	a	41	661	71	1130
River birch	Betula	nigra	373	1	b	a	34	544	62	992
Paper birch	Betula	papyrifera	375	25	25	c	34	550	59	940
Gray birch	Betula	populifolia	379	1	13	a	32	519	53	850
American hornbeam, musclewood	Carpinus	caroliniana	391	1	26	a	39	627	59	939
Water hickory	Carya	aquatica	401	25	25	a	44	706	80	1275
Bitternut hickory	Carya	cordiformis	402	25	25	a	43	696	74	1179
Pignut hickory	Carya	glabra	403	25	13	a	47	756	78	1243
Pecan	Carya	illinoinensis	404	25	25	a	43	696	72	1147
Shellbark hickory	Carya	laciniosa	405	25	13	a	45	716	74	1179
Nutmeg hickory	Carya	myristiciformis	406	25	29	a	41	656	72	1147
Shagbark hickory	Carya	ovata	407	25	13	a	47	755	74	1179
Mockernut hickory	Carya	alba	409	25	25	a	46	736	75	1195
American chestnut	Castanea	dentata	421	25	25	a	30	475	64	1023

Table 1B.—continued

Common name	Genus	Species	FIA code	Wood reference	Bark reference	Bark % reference (Tab 3)	Total oven-dry and green weight of wood and bark per cubic foot of wood *			
							Avg. oven-dry weight of wood and bark (lb/cf)	*Avg. oven-dry weight of wood and bark (kg/m3)*	*Avg. green weight of wood and bark (lb/cf)*	*Avg. green weight of wood and bark (kg/m3)*
Giant chinkapin, golden chinkapin	Chrysolepis	chrysophylla	431	1	26	30	29	470	67	1073
Northern catalpa	Catalpa	speciosa	452	1	26	a	28	455	50	799
Hackberry	Celtis	occidentalis	462	25	29	23	35	563	59	940
Flowering dogwood	Cornus	florida	491	1	b	a	45	727	63	1015
Pacific dogwood	Cornus	nuttallii	492	18	b	a	42	667	63	1015
Common persimmon	Diospyros	virginiana	521	26	26	a	45	715	72	1151
American beech	Fagus	grandifolia	531	25	13	11	37	600	59	941
White ash	Fraxinus	americana	541	25	25	a	39	630	59	952
Oregon ash	Fraxinus	latifolia	542	25	b	a	36	580	59	952
Black ash	Fraxinus	nigra	543	25	13	a	32	519	60	964
Green ash	Fraxinus	pennsylvanica	544	25	29	26	38	607	60	964
Pumpkin ash	Fraxinus	profunda	545	1	b	a	34	552	58	937
Blue ash	Fraxinus	quadrangulata	546	25	b	a	37	592	57	919
Honeylocust	Gleditsia	triacanthos	552	25	26	a	42	675	69	1103
Kentucky coffeetree	Gymnocladus	dioicus	571	1	b	a	38	605	58	921
Silverbell spp.	Halesia	spp.	580	1	26	a	31	495	53	847
American holly	Ilex	opaca	591	1	26	a	36	575	66	1055
Butternut	Juglans	cinerea	601	25	13	a	26	420	53	850
Black walnut	Juglans	nigra	602	25	13	a	35	559	63	1007
Sweetgum	Liquidambar	styraciflua	611	25	13	5	33	523	58	921
Yellow-poplar	Liriodendron	tulipifera	621	25	25	5	29	468	58	932
Tanoak	Lithocarpus	densiflorus	631	25	26	30	44	697	77	1230
Osage-orange	Maclura	pomifera	641	1	26	a	53	850	71	1137
Cucumbertree	Magnolia	acuminata	651	25	13	a	32	506	57	910
Southern magnolia	Magnolia	grandiflora	652	25	13	a	33	526	67	1070
Sweetbay	Magnolia	virginiana	653	1	b	a	30	486	57	919
Mountain or Fraser magnolia	Magnolia	fraseri	655	1	b	a	29	466	57	910
Apple spp.	Malus	spp.	660	26	25	a	43	685	76	1213
Water tupelo	Nyssa	aquatica	691	25	29	a	34	541	65	1045
Blackgum	Nyssa	sylvatica	693	25	25	26	33	521	64	1030
Eastern hophornbeam	Ostrya	virginiana	701	1	26	a	44	705	69	1103
Sourwood	Oxydendrum	arboreum	711	18	26	a	37	590	62	993
American sycamore	Platanus	occidentalis	731	25	13	23	32	508	58	921
Balsam poplar	Populus	balsamifera	741	25	13	a	26	420	53	845
Eastern cottonwood	Populus	deltoides	742	25	29	30	28	453	58	931
Bigtooth aspen	Populus	grandidentata	743	25	13	a	28	445	53	850
Quaking aspen	Populus	tremuloides	746	25	29	21	26	422	59	946
Black cottonwood	Populus	balsamifera	747	25	13	21	23	375	54	868
Fremont cottonwood	Populus	fremontii	748	30	b	a	31	500	51	813
Mesquite spp.	Prosopis	spp.	755	31	b	a	55	877	68	1082
Black cherry	Prunus	serotina	762	25	29	11	33	528	52	831
White oak	Quercus	alba	802	25	13	5	43	689	74	1178

Table 1B.—continued

Common name	Genus	Species	FIA code	Wood reference	Bark reference	Bark % reference (Tab 3)	Total oven-dry and green weight of wood and bark per cubic foot of wood *			
							Avg. oven-dry weight of wood and bark (lb/cf)	*Avg. oven-dry weight of wood and bark (kg/m3)*	*Avg. green weight of wood and bark (lb/cf)*	*Avg. green weight of wood and bark (kg/m3)*
Swamp white oak	Quercus	bicolor	804	25	13	a	45	728	73	1176
Canyon live oak	Quercus	chrysolepis	805	30	13	a	50	802	88	1412
Scarlet oak	Quercus	coccinea	806	25	13	a	47	756	79	1258
Southern red oak	Quercus	falcata	812	25	13	5	42	669	78	1247
Cherrybark oak	Quercus	pagoda	813	25	13	a	47	748	80	1289
Gambel oak	Quercus	gambelii	814	3	13	a	47	748	77	1238
Oregon white oak	Quercus	garryana	815	1	13	a	46	740	73	1176
California black oak	Quercus	kelloggii	818	18	13	a	38	609	76	1212
Laurel oak	Quercus	laurifolia	820	25	13	a	40	640	75	1202
California white oak	Quercus	lobata	821	30	13	a	40	638	73	1176
Overcup oak	Quercus	lyrata	822	25	13	a	43	682	76	1221
Bur oak	Quercus	macrocarpa	823	25	13	a	42	666	73	1173
Swamp chestnut oak	Quercus	michauxii	825	25	13	a	45	717	77	1230
Water oak	Quercus	nigra	827	25	13	a	41	659	75	1197
Pin oak	Quercus	palustris	830	25	13	a	44	712	80	1275
Willow oak	Quercus	phellos	831	25	13	a	41	654	75	1205
Chestnut oak	Quercus	prinus	832	25	13	5	43	694	75	1208
Northern red oak	Quercus	rubra	833	25	13	9	43	696	80	1285
Post oak	Quercus	stellata	835	25	13	a	44	712	77	1237
Black oak	Quercus	velutina	837	25	13	11	42	671	77	1236
Live oak	Quercus	virginiana	838	25	13	a	55	881	86	1371
Black locust	Robinia	pseudoacacia	901	25	26	a	44	703	63	1011
Black willow	Salix	nigra	922	25	13	a	27	440	61	976
Sassafras	Sassafras	albidum	931	25	26	a	31	495	53	847
American basswood	Tilia	americana	951	25	25	c	23	370	47	753
Winged elm	Ulmus	alata	971	1	b	a	41	663	60	959
American elm	Ulmus	americana	972	25	25	a	33	521	63	1002
Cedar elm	Ulmus	crassifolia	973	1	25	a	41	653	68	1087
Slippery elm	Ulmus	rubra	975	25	b	a	32	520	60	959
Rock elm	Ulmus	thomasii	977	25	25	a	40	640	61	970
California-laurel	Umbellularia	californica	981	1	30	a	37	592	60	967

** Moisture content is extremely variable and the values shown are averages or estimates based on the literature cited.*

a No reference source available, estimated based on similar species.

b Based on green volume specific gravity and bark moisture content of similar species.

c Adapted from McCormack (1955) using supplemental data from Forbes (1956) and Koch (1971)

Table 2A.—Double bark thickness (DBT) regression coeeficients, bark void factors and average bark percentage by species and d.b.h. (Equation: DBT = b0 + b1 x DIAMob). Reference numbers in this table refer to numbered citations found in Literature Cited section of this report.

Species	FIA code	EQ	Bark void factor	DBT eq. coef. *b0*	DBT eq. coef. *b1*	Reference	4	8	12	16	20	24	28+	AVG
							bark as percent of wood volume							
Subalpine fir	19	1	0.27	0.05	0.06	22	11.2	11.0	10.8	10.7	10.6	10.6	10.6	10.8
Alaska yellow-cedar	42	2	0.27	0.24	0.03	22	13.4	12.5	11.6	11.2	10.9	10.8	10.6	11.6
Engelmann/white spruce	93	3	0.27	0.15	0.04	22	12.3	11.8	11.2	11.0	10.8	10.7	10.6	11.2
Silka spruce	98	4	0.27	0.39	0.01	22	15.5	14.0	12.6	11.9	11.5	11.3	11.1	12.5
Lodgepole pine	108	5	0.20	0.07	0.04	22	9.4	9.2	8.9	8.7	8.6	8.6	8.6	8.9
Western white pine	119	6	0.27	0.11	0.05	22	11.6	11.2	10.9	10.7	10.5	10.5	10.4	12.6
Ponderosa pine	122	7	0.26	0.21	0.10	22	27.6	26.6	25.6	25.2	24.9	24.7	24.6	25.6
Eastern white pine	129	8	0.27	0.02	0.10	a	18.5	18.4	18.3	18.3	18.3	18.3	18.3	18.3
Loblolly pine	131	9	0.27	0.04	0.09	a	16.1	15.9	15.7	15.7	15.6	15.6	15.5	15.7
Douglas-fir, coastal	202	10	0.27	(0.23)	0.14	22	15.2	16.2	17.3	17.8	18.1	18.3	18.5	17.3
Douglas-fir, interior	202	11	0.27	(0.40)	0.17	22	14.5	16.2	18.1	19.0	19.6	20.0	20.3	18.3
W. redcedar, coastal	242	12	0.27	0.43	0.03	22	20.4	18.7	17.0	16.2	15.8	15.5	15.3	17.0
W. redcedar, interior	242	13	0.27	0.30	0.01	22	12.8	11.7	10.6	10.1	9.8	9.6	9.4	10.6
Eastern hemlock	261	14	0.27	0.18	0.08	a	21.5	20.7	20.0	19.6	19.4	19.2	19.1	19.9
W. hemlock, coastal	263	15	0.27	0.31	0.04	22	18.7	17.5	16.3	15.7	15.4	15.2	15.0	16.3
W. hemlock, interior	263	16	0.27	0.04	0.09	22	16.1	15.9	15.8	15.7	15.6	15.6	15.6	15.8
Red alder	351	17	0.23	0.16	0.04	22	13.3	12.7	12.1	11.8	11.6	11.5	11.4	12.0
Yellow birch	371	18	0.23	0.15	0.03	22	10.9	10.4	9.8	9.6	9.4	9.3	9.2	9.8
White birch	375	19	0.23	0.13	0.05	a	13.7	13.2	12.6	12.4	12.2	12.1	12.1	12.6
Ash	541	20	0.25	0.38	0.05	a	23.6	21.9	20.4	19.6	19.2	18.9	18.7	19.0
Trembling aspen	746	21	0.15	0.10	0.07	22	16.9	16.4	15.9	15.7	15.6	15.5	15.4	14.4
Black cottonwood	747	22	0.23	0.06	0.08	22	16.9	16.6	16.3	16.2	16.1	16.1	16.0	16.3
Red oak	806	23	0.20	0.19	0.07	a	19.7	18.8	18.0	17.6	17.4	17.2	17.1	18.0
Basswood	951	24	0.23	0.05	0.05	a	10.5	10.3	10.1	10.1	10.0	10.0	9.9	10.5
Softwoods, generic		25	0.27	0.30	0.02		13.8	12.7	11.6	11.1	10.8	10.6	10.4	13.4
Hardwoods, generic		26	0.23	0.12	0.06		15.0	14.5	14.0	13.8	13.6	13.5	13.5	15.0

(Columns 4 through AVG: d.b.h. class (inches))

a Adapted from McCormack (1955) using supplemental data from Forbes (1956) and Koch (1971)

NOTE: Bark voids are estimated based on data in Bowyer, J.L.; et al. 2007.

Table 2B.—Diameter inside bark (DIB) regression coeeficients, bark void factors, and average bole bark as a percentage of wood volume by species and d.b.h. class (Equation: DIB = b0 * DOB + b1 DOB2/DBH). Reference numbers in this table refer to numbered citations found in Literature Cited section of this report.

Species	FIA code	EQ	Bark void factor	DBT eq. coef. *b0*	DBT eq. coef. *b1*	Reference	4	8	12	16	20	24	28+	AVG
							--------------------------*bark as percent of wood volume*----------------------------							
Redmaple	316	27	0.23	0.92	0.05	11	7.2	7.9	8.6	8.9	9.1	9.3	9.4	8.6
Sugarmaple	318	28	0.23	0.87	0.06	11	13.4	14.4	15.5	16.1	16.4	16.6	16.8	15.6
Beech	531	29	0.05	0.93	0.04	11	4.7	5.3	6.0	6.3	6.5	6.6	6.7	6.0
Yellow-poplar	621	30	0.23	0.84	0.09	11	15.5	17.1	18.8	19.6	20.2	20.5	20.8	18.9
Blackcherry	762	31	0.23	0.93	0.02	11	8.7	8.9	9.2	9.3	9.4	9.4	9.5	9.2
Whiteoak	802	32	0.23	0.88	0.06	11	12.5	13.4	14.4	14.9	15.2	15.4	15.6	14.5
Southernredoak	812	33	0.23	0.89	0.04	11	13.7	14.4	15.1	15.4	15.7	15.8	15.9	15.1
Chestnutoak	832	34	0.23	0.77	0.15	11	18.7	21.7	24.9	26.7	27.8	28.5	29.1	25.3
Blackoak	837	35	0.23	0.83	0.10	11	14.5	16.4	18.3	19.4	20.0	20.4	20.7	18.5

(Columns 4 through 28+: D.b.h. class (inches))

Table 3.—Estimated average green bark volume as a percent of green wood volume. Reference numbers in this table refer to numbered citations found in Literature Cited section of this report.

Species	Reference	FIA code	Bark %	Species	Reference	FIA code	Bark %
Pacific silver fir	30	11	14.0	Bigleaf maple	30	312	10.0
Balsam Fir	9	12	12.0	Red maple	11	316	8.6
Subalpine fir	21	19	10.8	Sugar maple	11	318	15.6
Alaska yellow cedar	21	42	11.6	Red alder	21	351	12.0
Eastern redcedar	23	68	12.0	Yellow birch	21	371	9.8
Tamarack (native)	23	71	14.0	White birch	a	375	12.6
Spruce	10	90	13.0	Hickory	9	400	16.0
Engelmann spruce	21	93	11.2	Giant chinkapin	30	431	12.0
Silka spruce	21	98	12.5	Hackberry	23	462	15.0
Knobcone pine	30	103	12.0	Beech	11	531	6.0
Jack pine	9	105	14.0	Ash	9	540	16.0
Sand pine	26	107	15.0	Green Ash	26	544	16.0
Lodgepole pine	21	108	8.9	Walnut	23	600	15.0
Shortleaf pine	26	110	16.0	Sweetgum	5	611	15.0
Slash pine	26	111	18.0	Yellow-poplar	5	621	18.0
Western white pine	21	119	12.6	Tanoak	30	631	19.0
Longleaf Pine	26	121	14.0	Blackgum	26	693	14.0
Ponderosa pine	21	122	25.6	Sycamore	23	731	8.0
Red pine	23	125	16.0	E. cottonwood	30	742	22.0
Digger pine	30	127	22.0	Trembling aspen	21	746	14.4
Eastern white pine	9	129	16.0	Black cottonwood	21	747	16.3
Loblolly pine	19	131	16.6	Black cherry	11	762	9.2
Douglas-fir, coastal	21	202	17.3	White oak	5	802	16.0
Douglas-fir, interior	21	202	18.3	S. red oak	5	812	22.0
Redwood	30	211	18.0	Chestnut oak	5	832	23.0
Baldcypress	26	221	20.0	N. red oak	9	833	20.0
Pacific Yew	30	231	4.0	Black oak	11	837	18.5
N. white-cedar	9	241	14.0	Willow	23	920	16.0
W. redcedar, coastal	21	242	17.0	Basswood	a	951	10.5
W. redcedar, interior	21	242	10.6	Elm	23	970	14.0
Eastern hemlock	9	261	17.0	**Hardwoods, generic**		**999**	**15.0**
W. hemlock, coastal	21	263	16.3				
W. hemlock, interior	21	263	15.8				
**Softwoods, generic**		**299**	**13.4**				

a Adapted from McCormack (1955) using supplemental data from Forbes (1956) and Koch (1971)

NOTE: To express bark volume as a percentage of total volume, multiply the shown value by 1.0/(1.0 + shown value/100)

Table 4.—Specific gravity and bark percent assignment for trees in FIADB reference species table. Reference numbers in this table refer to numbered citations found in Literature Cited section of this report.

Common name	Genus	Species	FIA Code	Wood Specific gravity (green volume basis dry weight)	Reference	Bark Specific gravity (green volume basis dry weight)	Reference	Avg. moisture content of wood as a % of oven-dry weight	Reference	Avg. moisture content of bark as a % of oven-dry weight	Reference	Wood Specific gravity (12 pct MC volume basis dry weight)	Reference	Bark volume %	Reference
Fir spp.	Abies	spp.	10	0.36	b	0.49	b	84	b	62	b	0.38	b	11.8	f
Pacific silver fir	Abies	amabilis	11	0.40	25	0.44	30	70	30	64	22	0.43	25	14.0	30
Balsam fir	Abies	balsamea	12	0.33	25	0.40	12	119	12	100	2	0.35	25	12.0	9
Santa Lucia or bristlecone fir	Abies	bracteata	14	0.36	a	0.49	a	84	a	62	a	0.38	a	11.8	a
White fir	Abies	concolor	15	0.37	25	0.56	10	104	12	63	22	0.39	25	12.0	f
Fraser fir	Abies	fraseri	16	0.36	a	0.49	a	84	a	62	a	0.38	a	11.8	a
Grand fir	Abies	grandis	17	0.35	25	0.57	10	106	12	63	22	0.37	25	12.0	f
Corkbark fir	Abies	lasiocarpa var. arizonica	18	0.36	a	0.49	a	84	a	62	a	0.38	a	11.8	a
Subalpine fir	Abies	lasiocarpa	19	0.31	25	0.50	10	45	12	63	22	0.32	25	10.8	21
California red fir	Abies	magnifica	20	0.36	25	0.44	10	114	12	20	30	0.38	25	10.8	f
Shasta red fir	Abies	shastensis	21	0.36	a	0.49	a	84	a	62	a	0.38	a	11.8	a
Noble fir	Abies	procera	22	0.37	25	0.49	10	30	12	64	22	0.39	25	10.8	f
White-cedar spp.	Chamaecyparis	spp.	40	0.37	b	0.40	b	77	b	92	b	0.40	b	11.6	f
Port-Orford-cedar	Chamaecyparis	lawsoniana	41	0.39	25	0.40	30	74	25	64	25	0.43	25	11.6	f
Alaska yellow-cedar	Chamaecyparis	nootkatensis	42	0.42	25	0.40	22	72	29	112	22	0.44	25	11.6	21
Atlantic white-cedar	Chamaecyparis	thyoides	43	0.31	25	0.40	14	86	29	100	30	0.32	25	11.6	f
Cypress	Cupressus	spp.	50	0.41	c	0.42	c	74	c	71	c	0.44	c	14.3	c
Arizona cypress	Cupressus	arizonica	51	0.41	c	0.42	c	74	c	71	c	0.44	c	14.3	c
Baker or Modoc cypress	Cupressus	bakeri	52	0.41	c	0.42	c	74	c	71	c	0.44	c	14.3	c
Tecate cypress	Cupressus	forbesii	53	0.41	c	0.42	c	74	c	71	c	0.44	c	14.3	c
Monterey cypress	Cupressus	macrocarpa	54	0.41	c	0.42	c	74	c	71	c	0.44	c	14.3	c
Sargent's cypress	Cupressus	sargentii	55	0.41	c	0.42	c	74	c	71	c	0.44	c	14.3	c
MacNab's cypress	Cupressus	macnabiana	56	0.41	c	0.42	c	74	c	71	c	0.44	c	14.3	c
Redcedar/juniper spp.	Juniperus	spp.	57	0.45	b	0.40	b	36	b	60	b	0.47	b	12.0	f
Pinchot juniper	Juniperus	pinchotii	58	0.45	a	0.40	a	36	a	60	a	0.47	a	12.0	a
Redberry juniper	Juniperus	coahuilensis	59	0.45	a	0.40	a	36	a	60	a	0.47	a	12.0	a
Drooping juniper	Juniperus	flaccida	60	0.45	a	0.40	a	36	a	60	a	0.47	a	12.0	a
Ashe juniper	Juniperus	ashei	61	0.45	a	0.40	a	36	a	60	a	0.47	a	12.0	a
California juniper	Juniperus	californica	62	0.45	a	0.40	a	36	a	60	a	0.47	a	12.0	a

Table 4.—continued

Common name	Genus	Species	FIA Code	Wood Specific gravity (green volume basis dry weight)	Reference	Bark Specific gravity (green volume basis dry weight)	Reference	Avg. moisture content of wood as a % of oven-dry weight	Reference	Avg. moisture content of bark as a % of oven-dry weight	Reference	Wood Specific gravity (12 pct MC volume basis dry weight)	Reference	Bark volume %	Reference
Alligator juniper	Juniperus	deppeana	63	0.48	2	0.40	e	34	28	60	e	0.51	2	12.0	f
Western juniper	Juniperus	occidentalis	64	0.45	a	0.40	a	36	a	60	a	0.47	a	12.0	a
Utah juniper	Juniperus	osteosperma	65	0.68	3	0.40	e	35	e	60	e	0.72	f	12.0	f
Rocky Mountain juniper	Juniperus	scopulorum	66	0.45	a	0.40	a	36	a	60	a	0.47	a	12.0	a
Southern redcedar	Juniperus	virginiana	67	0.42	2	0.40	e	41	e	60	e	0.44	2	12.0	f
Eastern redcedar	Juniperus	virginiana	68	0.44	25	0.40	23	35	29	60	e	0.47	25	12.0	23
Oneseed juniper	Juniperus	monosperma	69	0.45	a	0.40	a	36	a	60	a	0.47	a	12.0	a
Larch spp.	Larix	spp.	70	0.49	b	0.32	b	57	b	82	b	0.53	b	14.0	f
Tamarack (native)	Larix	laricina	71	0.49	25	0.30	23	54	12	98	2	0.53	25	14.0	23
Subalpine larch	Larix	lyallii	72	0.49	a	0.32	a	57	a	82	a	0.53	a	14.0	a
Western larch	Larix	occidentalis	73	0.48	25	0.33	12	60	12	65	2	0.52	25	14.0	f
Incense-cedar	Calocedrus	decurrens	81	0.35	25	0.25	10	106	30	28	30	0.37	25	17.0	f
Spruce spp.	Picea	spp.	90	0.36	b	0.44	b	59	b	81	b	0.39	b	12.6	10
Norway spruce	Picea	abies	91	0.36	a	0.44	a	59	a	81	a	0.39	a	12.6	a
Brewer spruce	Picea	breweriana	92	0.36	a	0.44	a	59	a	81	a	0.39	a	12.6	a
Engelmann spruce	Picea	engelmannii	93	0.33	25	0.51	12	89	12	79	2	0.35	25	11.2	21
White spruce	Picea	glauca	94	0.37	25	0.39	12	52	12	77	30	0.40	25	13.0	f
Black spruce	Picea	mariana	95	0.38	25	0.42	12	48	12	91	2	0.46	25	13.0	f
Blue spruce	Picea	pungens	96	0.36	a	0.44	a	59	a	81	a	0.39	a	12.6	a
Red spruce	Picea	rubens	97	0.37	25	0.32	14	47	12	75	e	0.40	25	13.0	f
Sitka spruce	Picea	sitchensis	98	0.33	25	0.55	10	60	12	81	22	0.36	25	12.5	21
Pine spp.	Pinus	spp.	100	0.43	b	0.40	b	76	b	68	b	0.47	b	16.1	f
Whitebark pine	Pinus	albicaulis	101	0.43	a	0.40	a	76	a	68	a	0.47	a	16.1	a
Rocky Mountain bristlecone pine	Pinus	aristata	102	0.43	a	0.40	a	76	a	68	a	0.47	a	16.1	a
Knobcone pine	Pinus	attenuata	103	0.39	30	0.38	30	105	e	69	e	0.42	f	12.0	30
Foxtail pine	Pinus	balfouriana	104	0.43	a	0.40	a	76	a	68	a	0.47	a	16.1	a
Jack pine	Pinus	banksiana	105	0.40	25	0.41	12	100	12	92	2	0.43	25	14.0	9
Common or two-needle pinyon	Pinus	edulis	106	0.50	2	0.40	e	28	e	60	e	0.57	2	13.4	f
Sand pine	Pinus	clausa	107	0.46	25	0.45	14	32	12	89	2	0.48	25	15.0	26
Lodgepole pine	Pinus	contorta	108	0.38	25	0.38	12	64	12	64	2	0.41	25	8.9	21

Table 4.—continued

Common name	Genus	Species	FIA Code	Wood Specific gravity (green volume basis dry weight)	Reference	Bark Specific gravity (green volume basis dry weight)	Reference	Avg. moisture content of wood as a % of oven-dry weight	Reference	Avg. moisture content of bark as a % of oven-dry weight	Reference	Wood Specific gravity (12 pct MC volume basis dry weight)	Reference	Bark volume %	Reference
Coulter pine	Pinus	coulteri	109	0.43	a	0.40	a	76	a	68	a	0.47	a	16.1	a
Shortleaf pine	Pinus	echinata	110	0.47	25	0.35	12	77	12	60	e	0.51	25	16.0	26
Slash pine	Pinus	elliottii	111	0.54	25	0.35	12	72	12	88	2	0.59	25	18.0	26
Apache pine	Pinus	engelmannii	112	0.43	a	0.40	a	76	a	68	a	0.47	a	16.1	a
Limber pine	Pinus	flexilis	113	0.37	2	0.50	e	95	e	12	e	0.42	2	13.4	f
Southwestern white pine	Pinus	strobiformis	114	0.43	a	0.40	a	76	a	68	a	0.47	a	16.1	a
Spruce pine	Pinus	glabra	115	0.41	25	0.45	14	76	e	25	e	0.44	25	13.4	f
Jeffrey pine	Pinus	jeffreyi	116	0.37	30	0.36	30	104	12	34	2	0.42	25	25.6	f
Sugar pine	Pinus	lambertiana	117	0.34	25	0.35	10	130	25	88	2	0.36	25	25.6	f
Chihuahua pine	Pinus	leiophylla	118	0.43	a	0.40	a	76	a	68	a	0.47	a	16.1	a
Western white pine	Pinus	monticola	119	0.36	25	0.47	12	56	12	84	22	0.38	25	12.6	21
Bishop pine	Pinus	muricata	120	0.45	30	0.45	30	60	e	42	e	0.49	f	22.0	f
Longleaf pine	Pinus	palustris	121	0.54	25	0.45	12	63	12	89	2	0.59	25	14.0	26
Ponderosa pine	Pinus	ponderosa	122	0.38	25	0.35	12	90	12	33	2	0.40	25	25.6	21
Table Mountain pine	Pinus	pungens	123	0.49	2	0.45	e	77	e	60	e	0.52	2	13.4	f
Monterey pine	Pinus	radiata	124	0.40	2	0.40	e	100	e	60	e	0.53	2	13.4	f
Red pine	Pinus	resinosa	125	0.41	25	0.27	12	64	12	90	e	0.46	25	16.0	23
Pitch pine	Pinus	rigida	126	0.47	25	0.34	10	70	12	89	2	0.52	25	13.4	f
Gray or California foothill pine	Pinus	sabiniana	127	0.40	30	0.40	30	80	e	60	e	0.43	f	22.0	30
Pond pine	Pinus	serotina	128	0.51	25	0.33	e	60	e	94	e	0.56	25	13.4	f
Eastern white pine	Pinus	strobus	129	0.34	25	0.47	12	65	e	70	e	0.35	25	16.0	9
Scotch pine	Pinus	sylvestris	130	0.43	a	0.40	a	76	a	68	a	0.47	a	16.1	a
Loblolly pine	Pinus	taeda	131	0.47	25	0.33	12	81	12	99	2	0.51	25	16.6	19
Virginia pine	Pinus	virginiana	132	0.45	25	0.54	12	78	e	90	e	0.48	25	13.4	f
Singleleaf pinyon	Pinus	monophylla	133	0.43	a	0.40	a	76	a	68	a	0.47	a	16.1	a
Border pinyon	Pinus	discolor	134	0.43	a	0.40	a	76	a	68	a	0.47	a	16.1	a
Arizona pine	Pinus	arizonica	135	0.43	a	0.40	a	76	a	68	a	0.47	a	16.1	a
Austrian pine	Pinus	nigra	136	0.43	a	0.40	a	76	a	68	a	0.47	a	16.1	a
Washoe pine	Pinus	washoensis	137	0.43	a	0.40	a	76	a	68	a	0.47	a	16.1	a
Four-leaf or Parry pinyon pine	Pinus	quadrifolia	138	0.43	a	0.40	a	76	a	68	a	0.47	a	16.1	a

Table 4.—continued

Common name	Genus	Species	FIA Code	Wood Specific gravity (green volume basis dry weight)	Reference	Bark Specific gravity (green volume basis dry weight)	Reference	Avg. moisture content of wood as a % of oven-dry weight	Reference	Avg. moisture content of bark as a % of oven-dry weight	Reference	Wood Specific gravity (12 pct MC volume basis dry weight)	Reference	Bark volume %	Reference
Torrey pine	Pinus	torreyana	139	0.43	a	0.40	a	76	a	68	a	0.47	a	16.1	a
Mexican pinyon pine	Pinus	cembroides	140	0.43	a	0.40	a	76	a	68	a	0.47	a	16.1	a
Papershell pinyon pine	Pinus	remota	141	0.43	a	0.40	a	76	a	68	a	0.47	a	16.1	a
Great Basin bristlecone pine	Pinus	longaeva	142	0.43	a	0.40	a	76	a	68	a	0.47	a	16.1	a
Arizona pinyon pine	Pinus	monophylla	143	0.43	a	0.40	a	76	a	68	a	0.47	a	16.1	a
Honduras pine	Pinus	elliottii	144	0.43	a	0.40	a	76	a	68	a	0.47	a	16.1	a
Douglas-fir spp.	Pseudotsuga	spp.	200	0.45	b	0.44	b	35	b	89	b	0.48	b	17.3	f
Bigcone Douglas-fir	Pseudotsuga	macrocarpa	201	0.45	a	0.44	a	35	a	89	a	0.48	a	17.3	a
Douglas-fir	Pseudotsuga	menziesii	202	0.45	25	0.44	26	35	12	89	2	0.48	25	17.3	21
Redwood	Sequoia	sempervirens	211	0.36	25	0.43	10	123	12	16	30	0.38	25	18.0	30
Giant sequoia	Sequoiadendron	giganteum	212	0.34	30	0.34	30	178	30	18	e	0.38	f	18.0	f
Baldcypress spp.	Taxodium	spp.	220	0.42	b	0.50	b	95	b	99	b	0.46	b	20.0	f
Baldcypress	Taxodium	distichum	221	0.42	25	0.50	10	95	29	99	e	0.46	25	20.0	26
Pondcypress	Taxodium	ascendens	222	0.42	a	0.50	a	95	a	99	a	0.46	a	20.0	a
Montezuma baldcypress	Taxodium	mucronatum	223	0.42	a	0.50	a	95	a	99	a	0.46	a	20.0	a
Yew spp.	Taxus	spp.	230	0.60	b	0.59	b	47	b	98	b	0.67	b	4.0	f
Pacific yew	Taxus	brevifolia	231	0.60	2	0.59	30	47	30	98	30	0.67	2	4.0	30
Florida yew	Taxus	floridana	232	0.60	a	0.59	a	47	a	98	a	0.67	a	4.0	a
Thuja spp.	Thuja	spp.	240	0.30	b	0.40	b	70	b	74	b	0.32	b	12.5	f
Northern white-cedar	Thuja	occidentalis	241	0.29	25	0.42	10	99	23	91	e	0.31	25	14.0	9
Western redcedar	Thuja	plicata	242	0.31	25	0.37	12	40	12	56	22	0.32	25	10.6	21
Torreya (nutmeg) spp.	Torreya	spp.	250	0.41	c	0.42	c	74	c	71	c	0.44	c	14.3	c
California torreya (nutmeg)	Torreya	californica	251	0.41	c	0.42	c	74	c	71	c	0.44	c	14.3	c
Florida torreya (nutmeg)	Torreya	taxifolia	252	0.41	c	0.42	c	74	c	71	c	0.44	c	14.3	c
Hemlock spp.	Tsuga	spp.	260	0.41	b	0.46	b	78	b	98	b	0.43	b	16.2	f
Eastern hemlock	Tsuga	canadensis	261	0.38	25	0.46	10	111	12	99	2	0.40	25	17.0	9
Carolina hemlock	Tsuga	caroliniana	262	0.41	a	0.46	a	78	a	98	a	0.43	a	16.2	a
Western hemlock	Tsuga	heterophylla	263	0.42	25	0.50	26	56	12	102	22	0.45	25	15.8	21
Mountain hemlock	Tsuga	mertensiana	264	0.42	25	0.41	10	68	12	92	e	0.45	25	15.8	f
Unknown dead conifer	Tree	evergreen	299	0.41	c	0.42	c	74	c	71	c	0.44	c	14.3	c

Table 4.—continued

Common name	Genus	Species	FIA Code	Wood Specific gravity (green volume basis dry weight)	Reference	Bark Specific gravity (green volume basis dry weight)	Reference	Avg. moisture content of wood as a % of oven-dry weight	Reference	Avg. moisture content of bark as a % of oven-dry weight	Reference	Wood Specific gravity (12 pct MC volume basis dry weight)	Reference	Bark volume %	Reference
Acacia spp.	Acacia	spp.	300	0.52	c	0.53	c	75	c	80	c	0.58	c	15.2	c
Sweet acacia	Acacia	farnesiana	303	0.52	c	0.53	c	75	c	80	c	0.58	c	15.2	c
Catclaw acacia	Acacia	greggii	304	0.52	c	0.53	c	75	c	80	c	0.58	c	15.2	c
Maple spp.	Acer	spp.	310	0.47	b	0.53	b	70	b	91	b	0.52	b	10.9	f
Florida maple	Acer	barbatum	311	0.47	a	0.53	a	70	a	91	a	0.52	a	10.9	a
Bigleaf maple	Acer	macrophyllum	312	0.44	25	0.48	10	71	13	114	22	0.48	25	10.0	30
Boxelder	Acer	negundo	313	0.42	31	0.50	e	91	e	92	e	0.46	31	8.6	f
Black maple	Acer	nigrum	314	0.52	25	0.54	14	70	e	90	1	0.57	25	15.6	f
Striped maple	Acer	pensylvanicum	315	0.44	1	0.50	e	71	e	89	e	0.46	1	8.6	f
Red maple	Acer	rubrum	316	0.49	25	0.60	13	64	13	79	1	0.54	25	8.6	11
Silver maple	Acer	saccharinum	317	0.44	25	0.57	13	68	13	80	e	0.47	25	8.6	f
Sugar maple	Acer	saccharum	318	0.56	25	0.54	13	57	13	90	1	0.63	25	15.6	11
Mountain maple	Acer	spicatum	319	0.47	a	0.53	a	70	a	91	a	0.52	a	10.9	a
Norway maple	Acer	platanoides	320	0.47	a	0.53	a	70	a	91	a	0.52	a	10.9	a
Rocky Mountain maple	Acer	glabrum	321	0.47	a	0.53	a	70	a	91	a	0.52	a	10.9	a
Bigtooth maple	Acer	grandidentatum	322	0.47	a	0.53	a	70	a	91	a	0.52	a	10.9	a
Chalk maple	Acer	leucoderme	323	0.47	a	0.53	a	70	a	91	a	0.52	a	10.9	a
Buckeye, horsechestnut spp.	Aesculus	spp.	330	0.33	d	0.50	d	143	d	89	d	0.36	d	15.0	d
Ohio buckeye	Aesculus	glabra	331	0.33	d	0.50	d	143	d	89	d	0.36	d	15.0	d
Yellow buckeye	Aesculus	flava	332	0.33	1	0.50	e	143	13	89	e	0.36	1	15.0	f
California buckeye	Aesculus	californica	333	0.33	d	0.50	d	143	d	89	d	0.36	d	15.0	d
Texas buckeye	Aesculus	glabra	334	0.33	d	0.50	d	143	d	89	d	0.36	d	15.0	d
Red buckeye	Aesculus	pavia	336	0.33	d	0.50	d	143	d	89	d	0.36	d	15.0	d
Painted buckeye	Aesculus	sylvatica	337	0.33	d	0.50	d	143	d	89	d	0.36	d	15.0	d
Ailanthus	Ailanthus	altissima	341	0.46	1	0.45	e	74	e	78	e	0.53	1	15.0	f
Mimosa, silktree	Albizia	julibrissin	345	0.52	c	0.53	c	75	c	80	c	0.58	c	15.2	c
Alder spp.	Alnus	spp.	350	0.37	b	0.56	b	99	b	75	b	0.41	b	11.5	f
Red alder	Alnus	rubra	351	0.37	25	0.56	26	99	13	75	22	0.41	25	12.0	21
White alder	Alnus	rhombifolia	352	0.37	a	0.56	a	99	a	75	a	0.41	a	11.5	a
Arizona alder	Alnus	oblongifolia	353	0.37	a	0.56	a	99	a	75	a	0.41	a	11.5	a

Table 4.—continued

Common name	Genus	Species	FIA Code	Wood Specific gravity (green volume basis dry weight)	Reference	Bark Specific gravity (green volume basis dry weight)	Reference	Avg. moisture content of wood as a % of oven-dry weight	Reference	Avg. moisture content of bark as a % of oven-dry weight	Reference	Wood Specific gravity (12 pct MC volume basis dry weight)	Reference	Bark volume %	Reference
European alder	Alnus	glutinosa	355	0.37	a	0.56	a	99	a	75	a	0.41	a	11.5	a
Serviceberry spp.	Amelanchier	spp.	356	0.66	1	0.50	e	48	26	60	e	0.79	1	8.6	f
Common serviceberry	Amelanchier	arborea	357	0.66	a	0.50	a	48	a	60	a	0.79	a	8.6	a
Roundleaf serviceberry	Amelanchier	sanguinea	358	0.66	a	0.50	a	48	a	60	a	0.79	a	8.6	a
Madrone spp.	Arbutus	spp.	360	0.58	d	0.60	d	66	d	60	d	0.65	d	15.0	d
Pacific madrone	Arbutus	menziesii	361	0.58	1	0.60	25	66	26	60	e	0.65	1	15.0	f
Arizona madrone	Arbutus	arizonica	362	0.58	d	0.60	d	66	d	60	d	0.65	d	15.0	d
Texas madrone	Arbutus	xalapensis	363	0.58	d	0.60	d	66	d	60	d	0.65	d	15.0	d
Pawpaw	Asimina	triloba	367	0.52	c	0.53	c	75	c	80	c	0.58	c	15.2	c
Birch spp.	Betula	spp.	370	0.51	b	0.58	b	74	b	55	b	0.58	b	11.0	f
Yellow birch	Betula	alleghaniensis	371	0.55	25	0.62	10	72	25	60	e	0.62	25	9.8	21
Sweet birch	Betula	lenta	372	0.60	25	0.62	e	73	25	53	1	0.65	25	9.8	f
River birch	Betula	nigra	373	0.49	1	0.55	e	86	e	46	e	0.56	1	9.8	f
Water birch	Betula	occidentalis	374	0.51	a	0.58	a	74	a	55	a	0.58	a	11.0	a
Paper birch	Betula	papyrifera	375	0.48	25	0.56	13	74	25	52	22	0.55	25	12.6	8
Virginia roundleaf birch	Betula	uber	377	0.51	a	0.58	a	74	a	55	a	0.58	a	11.0	a
Northwestern paper birch	Betula	x utahensis	378	0.51	a	0.58	a	74	a	55	a	0.58	a	11.0	a
Gray birch	Betula	populifolia	379	0.45	1	0.55	e	64	13	63	1	0.51	1	12.6	f
Chittamwood, gum bumelia	Sideroxylon	lanuginosum	381	0.52	c	0.53	c	75	c	80	c	0.58	c	15.2	c
American hornbeam, musclewood	Carpinus	caroliniana	391	0.58	1	0.55	e	46	26	89	e	0.70	1	8.6	f
Hickory spp.	Carya	spp.	400	0.62	b	0.62	b	69	b	57	5	0.68	b	16.0	9
Water hickory	Carya	aquatica	401	0.61	25	0.60	14	84	25	60	e	0.62	25	16.0	f
Bitternut hickory	Carya	cordiformis	402	0.60	25	0.60	23	71	25	60	e	0.66	25	16.0	f
Pignut hickory	Carya	glabra	403	0.66	25	0.60	23	65	13	60	e	0.75	25	16.0	f
Pecan	Carya	illinoinensis	404	0.60	25	0.60	14	66	25	60	e	0.66	25	16.0	f
Shellbark hickory	Carya	laciniosa	405	0.62	25	0.60	14	65	13	60	e	0.69	25	16.0	f
Nutmeg hickory	Carya	myristiciformis	406	0.56	25	0.60	14	77	29	60	e	0.60	25	16.0	f
Shagbark hickory	Carya	ovata	407	0.64	25	0.72	13	60	13	34	e	0.72	25	16.0	f
Black hickory	Carya	texana	408	0.62	a	0.62	a	69	a	57	a	0.68	a	16.0	a
Mockernut hickory	Carya	alba	409	0.64	25	0.60	23	63	25	60	e	0.72	25	16.0	f

Table 4.—continued

Common name	Genus	Species	FIA Code	Wood Specific gravity (green volume basis dry weight)	Reference	Bark Specific gravity (green volume basis dry weight)	Reference	Avg. moisture content of wood as a % of oven-dry weight	Reference	Avg. moisture content of bark as a % of oven-dry weight	Reference	Wood Specific gravity (12 pct MC volume basis dry weight)	Reference	Bark volume %	Reference
Sand hickory	Carya	pallida	410	0.62	a	0.62	a	69	a	57	a	0.68	a	16.0	a
Scrub hickory	Carya	floridana	411	0.62	a	0.62	a	69	a	57	a	0.68	a	16.0	a
Red hickory	Carya	ovalis	412	0.62	a	0.62	a	69	a	57	a	0.68	a	16.0	a
Southern shagbark hickory	Carya	carolinae-septentrionalis	413	0.62	a	0.62	a	69	a	57	a	0.68	a	16.0	a
Chestnut spp.	Castanea	spp.	420	0.40	d	0.50	d	120	d	89	d	0.43	d	15.0	d
American chestnut	Castanea	dentata	421	0.40	25	0.50	14	120	25	89	e	0.43	25	15.0	f
Allegheny chinkapin	Castanea	pumila	422	0.40	d	0.50	d	120	d	89	d	0.43	d	15.0	d
Ozark chinkapin	Castanea	pumila	423	0.40	d	0.50	d	120	d	89	d	0.43	d	15.0	d
Chinese chestnut	Castanea	mollissima	424	0.40	d	0.50	d	120	d	89	d	0.43	d	15.0	d
Giant chinkapin, golden chinkapin	Chrysolepis	chrysophylla	431	0.42	1	0.42	30	133	26	91	e	0.46	1	12.0	30
Catalpa spp.	Catalpa	spp.	450	0.38	d	0.50	d	73	d	89	d	0.41	d	15.0	d
Southern catalpa	Catalpa	bignonioides	451	0.38	d	0.50	d	73	d	89	d	0.41	d	15.0	d
Northern catalpa	Catalpa	speciosa	452	0.38	1	0.50	e	73	26	89	e	0.41	1	15.0	f
Hackberry spp.	Celtis	spp.	460	0.49	d	0.49	d	64	d	90	d	0.53	d	15.0	d
Sugarberry	Celtis	laevigata	461	0.49	d	0.49	d	64	d	90	d	0.53	d	15.0	d
Hackberry	Celtis	occidentalis	462	0.49	25	0.49	23	64	29	90	e	0.53	25	15.0	23
Netleaf hackberry	Celtis	laevigata	463	0.49	d	0.49	d	64	d	90	d	0.53	d	15.0	d
Eastern redbud	Cercis	canadensis	471	0.52	c	0.53	c	75	c	80	c	0.58	c	15.2	c
Curlleaf mountain-mahogany	Cercocarpus	ledifolius	475	0.52	c	0.53	c	75	c	80	c	0.58	c	15.2	c
Yellowwood	Cladrastis	kentukea	481	0.52	c	0.53	c	75	c	80	c	0.58	c	15.2	c
Dogwood spp.	Cornus	spp.	490	0.61	d	0.58	d	40	d	91	d	0.68	d	15.0	d
Flowering dogwood	Cornus	florida	491	0.64	1	0.58	e	33	e	91	e	0.73	1	15.0	f
Pacific dogwood	Cornus	nuttallii	492	0.58	18	0.58	18	46	e	91	e	0.62	f	15.0	f
Hawthorn spp.	Crataegus	spp.	500	0.52	c	0.53	c	75	c	80	c	0.58	c	15.2	c
Cockspur hawthorn	Crataegus	crus-galli	501	0.52	c	0.53	c	75	c	80	c	0.58	c	15.2	c
Downy hawthorn	Crataegus	mollis	502	0.52	c	0.53	c	75	c	80	c	0.58	c	15.2	c
Brainerd’s hawthorn	Crataegus	brainerdii	503	0.52	c	0.53	c	75	c	80	c	0.58	c	15.2	c
Pear hawthorn	Crataegus	calpodendron	504	0.52	c	0.53	c	75	c	80	c	0.58	c	15.2	c
Fireberry hawthorn	Crataegus	chrysocarpa	505	0.52	c	0.53	c	75	c	80	c	0.58	c	15.2	c
Broadleaf hawthorn	Crataegus	dilatata	506	0.52	c	0.53	c	75	c	80	c	0.58	c	15.2	c

Table 4.—continued

Common name	Genus	Species	FIA Code	Wood Specific gravity (green volume basis dry weight)	Reference	Bark Specific gravity (green volume basis dry weight)	Reference	Avg. moisture content of wood as a % of oven-dry weight	Reference	Avg. moisture content of bark as a % of oven-dry weight	Reference	Wood Specific gravity (12 pct MC volume basis dry weight)	Reference	Bark volume %	Reference
Fanleaf hawthorn	Crataegus	flabellata	507	0.52	c	0.53	c	75	c	80	c	0.58	c	15.2	c
Oneseed hawthorn	Crataegus	monogyna	508	0.52	c	0.53	c	75	c	80	c	0.58	c	15.2	c
Scarlet hawthorn	Crataegus	pedicellata	509	0.52	c	0.53	c	75	c	80	c	0.58	c	15.2	c
Eucalyptus spp.	Eucalyptus	spp.	510	0.52	c	0.53	c	75	c	80	c	0.58	c	15.2	c
Tasmanian bluegum	Eucalyptus	globulus	511	0.52	c	0.53	c	75	c	80	c	0.58	c	15.2	c
River redgum	Eucalyptus	camaldulensis	512	0.52	c	0.53	c	75	c	80	c	0.58	c	15.2	c
Grand eucalyptus	Eucalyptus	grandis	513	0.52	c	0.53	c	75	c	80	c	0.58	c	15.2	c
Swampmahogany	Eucalyptus	robusta	514	0.52	c	0.53	c	75	c	80	c	0.58	c	15.2	c
Persimmon spp.	Diospyros	spp.	520	0.64	d	0.50	d	58	d	89	d	0.74	d	15.0	d
Common persimmon	Diospyros	virginiana	521	0.64	26	0.50	e	58	26	89	e	0.74	26	15.0	f
Texas persimmon	Diospyros	texana	522	0.64	d	0.50	d	58	d	89	d	0.74	d	15.0	d
Anacua knockaway	Ehretia	anacua	523	0.52	c	0.53	c	75	c	80	c	0.58	c	15.2	c
American beech	Fagus	grandifolia	531	0.56	25	0.67	13	55	13	89	e	0.64	25	6.0	11
Ash spp.	Fraxinus	spp.	540	0.51	b	0.46	b	61	b	86	b	0.55	b	16.0	9
White ash	Fraxinus	americana	541	0.55	25	0.50	13	46	25	89	e	0.60	25	16.0	f
Oregon ash	Fraxinus	latifolia	542	0.50	25	0.50	14	60	e	89	e	0.55	25	16.0	f
Black ash	Fraxinus	nigra	543	0.45	25	0.43	10	85	13	90	e	0.49	25	16.0	f
Green ash	Fraxinus	pennsylvanica	544	0.53	25	0.48	13	57	29	70	5	0.56	25	16.0	26
Pumpkin ash	Fraxinus	profunda	545	0.48	1	0.45	e	67	e	89	e	0.52	1	16.0	f
Blue ash	Fraxinus	quadrangulata	546	0.53	25	0.39	14	51	e	89	e	0.58	25	16.0	f
Velvet ash	Fraxinus	velutina	547	0.51	a	0.46	a	61	a	86	a	0.55	a	16.0	a
Carolina ash	Fraxinus	caroliniana	548	0.51	a	0.46	a	61	a	86	a	0.55	a	16.0	a
Texas ash	Fraxinus	texensis	549	0.51	a	0.46	a	61	a	86	a	0.55	a	16.0	a
Honeylocust spp.	Gleditsia	spp.	550	0.60	d	0.50	d	60	d	89	d	0.65	d	15.0	d
Waterlocust	Gleditsia	aquatica	551	0.60	d	0.50	d	60	d	89	d	0.65	d	15.0	d
Honeylocust	Gleditsia	triacanthos	552	0.60	25	0.50	14	60	26	89	e	0.65	f	15.0	f
Loblolly-bay	Gordonia	lasianthus	555	0.52	c	0.53	c	75	c	80	c	0.58	c	15.2	c
Ginkgo, maidenhair tree	Ginkgo	biloba	561	0.52	c	0.53	c	75	c	80	c	0.58	c	15.2	c
Kentucky coffeetree	Gymnocladus	dioicus	571	0.53	1	0.50	e	51	e	60	e	0.60	1	15.0	f
Silverbell spp.	Halesia	spp.	580	0.42	1	0.50	e	68	26	89	e	0.45	1	15.0	f

Table 4.—continued

Common name	Genus	Species	FIA Code	Wood Specific gravity (green volume basis dry weight)	Reference	Bark Specific gravity (green volume basis dry weight)	Reference	Avg. moisture content of wood as a % of oven-dry weight	Reference	Avg. moisture content of bark as a % of oven-dry weight	Reference	Wood Specific gravity (12 pct MC volume basis dry weight)	Reference	Bark volume %	Reference
Carolina silverbell	Halesia	carolina	581	0.42	a	0.50	a	68	a	89	a	0.45	a	15.0	a
Two-wing silverbell	Halesia	diptera	582	0.42	a	0.50	a	68	a	89	a	0.45	a	15.0	a
Little silverbell	Halesia	parviflora	583	0.42	a	0.50	a	68	a	89	a	0.45	a	15.0	a
American holly	Ilex	opaca	591	0.50	1	0.50	e	83	26	89	e	0.57	1	15.0	f
Walnut spp.	Juglans	spp.	600	0.44	b	0.37	b	92	b	89	b	0.47	b	15.0	23
Butternut	Juglans	cinerea	601	0.36	25	0.40	14	105	13	88	e	0.38	25	15.0	f
Black walnut	Juglans	nigra	602	0.51	25	0.33	10	79	13	89	e	0.55	25	15.0	f
Northern California black walnut	Juglans	hindsii	603	0.44	a	0.37	a	92	a	89	a	0.47	a	15.0	a
Southern California black walnut	Juglans	californica	604	0.44	a	0.37	a	92	a	89	a	0.47	a	15.0	a
Texas walnut	Juglans	microcarpa	605	0.44	a	0.37	a	92	a	89	a	0.47	a	15.0	a
Arizona walnut	Juglans	major	606	0.44	a	0.37	a	92	a	89	a	0.47	a	15.0	a
Sweetgum	Liquidambar	styraciflua	611	0.46	25	0.42	13	74	13	91	1	0.52	25	15.0	5
Yellow-poplar	Liriodendron	tulipifera	621	0.40	25	0.38	13	95	25	124	5	0.42	25	18.0	5
Tanoak	Lithocarpus	densiflorus	631	0.58	25	0.62	10	80	26	60	e	0.62	f	19.0	30
Osage-orange	Maclura	pomifera	641	0.76	1	0.60	e	31	26	60	e	0.85	1	15.0	f
Magnolia spp.	Magnolia	spp.	650	0.43	b	0.44	b	92	b	93	b	0.47	b	15.0	f
Cucumbertree	Magnolia	acuminata	651	0.44	25	0.44	14	78	13	89	e	0.48	25	15.0	f
Southern magnolia	Magnolia	grandiflora	652	0.46	25	0.44	14	106	13	89	e	0.50	25	15.0	f
Sweetbay	Magnolia	virginiana	653	0.42	1	0.44	e	87	e	104	1	0.46	1	15.0	f
Bigleaf magnolia	Magnolia	macrophylla	654	0.43	a	0.44	a	92	a	93	a	0.47	a	15.0	a
Mountain or Fraser magnolia	Magnolia	fraseri	655	0.40	1	0.44	e	96	e	89	e	0.44	1	15.0	f
Pyramid magnolia	Magnolia	pyramidata	657	0.43	a	0.44	a	92	a	93	a	0.47	a	15.0	a
Umbrella magnolia	Magnolia	tripetala	658	0.43	a	0.44	a	92	a	93	a	0.47	a	15.0	a
Apple spp.	Malus	spp.	660	0.61	26	0.50	e	78	25	70	e	0.67	26	15.0	f
Oregon crab apple	Malus	fusca	661	0.61	a	0.50	a	78	a	70	a	0.67	a	15.0	a
Southern crab apple	Malus	angustifolia	662	0.61	a	0.50	a	78	a	70	a	0.67	a	15.0	a
Sweet crab apple	Malus	coronaria	663	0.61	a	0.50	a	78	a	70	a	0.67	a	15.0	a
Prairie crab apple	Malus	ioensis	664	0.61	a	0.50	a	78	a	70	a	0.67	a	15.0	a
Mulberry spp.	Morus	spp.	680	0.52	c	0.53	c	75	c	80	c	0.58	c	15.2	c
White mulberry	Morus	alba	681	0.52	c	0.53	c	75	c	80	c	0.58	c	15.2	c

Table 4.—continued

Common name	Genus	Species	FIA Code	Wood Specific gravity (green volume basis dry weight)	Reference	Bark Specific gravity (green volume basis dry weight)	Reference	Avg. moisture content of wood as a % of oven-dry weight	Reference	Avg. moisture content of bark as a % of oven-dry weight	Reference	Wood Specific gravity (12 pct MC volume basis dry weight)	Reference	Bark volume %	Reference
Red mulberry	Morus	rubra	682	0.52	c	0.53	c	75	c	80	c	0.58	c	15.2	c
Texas mulberry	Morus	microphylla	683	0.52	c	0.53	c	75	c	80	c	0.58	c	15.2	c
Black mulberry	Morus	nigra	684	0.52	c	0.53	c	75	c	80	c	0.58	c	15.2	c
Tupelo spp.	Nyssa	spp.	690	0.46	b	0.51	b	98	b	77	b	0.50	b	14.0	f
Water tupelo	Nyssa	aquatica	691	0.46	25	0.58	10	95	29	82	1	0.50	25	14.0	f
Ogeechee tupelo	Nyssa	ogeche	692	0.46	a	0.51	a	98	a	77	a	0.50	a	14.0	a
Blackgum	Nyssa	sylvatica	693	0.46	25	0.44	13	101	25	71	1	0.50	25	14.0	26
Swamp tupelo	Nyssa	biflora	694	0.46	a	0.51	a	98	a	77	a	0.50	a	14.0	a
Eastern hophornbeam	Ostrya	virginiana	701	0.63	1	0.50	e	53	26	89	e	0.70	1	15.0	f
Sourwood	Oxydendrum	arboreum	711	0.50	18	0.60	e	70	26	60	e	0.55	1	15.0	f
Paulownia, empress-tree	Paulownia	tomentosa	712	0.52	c	0.53	c	75	c	80	c	0.58	c	15.2	c
Bay spp.	Persea	spp.	720	0.52	c	0.53	c	75	c	80	c	0.58	c	15.2	c
Redbay	Persea	borbonia	721	0.52	c	0.53	c	75	c	80	c	0.58	c	15.2	c
Water-elm, planertree	Planera	aquatica	722	0.52	c	0.53	c	75	c	80	c	0.58	c	15.2	c
Sycamore spp.	Platanus	spp.	729	0.46	d	0.60	d	81	d	84	d	0.49	d	8.0	d
California sycamore	Platanus	racemosa	730	0.46	d	0.60	d	81	d	84	d	0.49	d	8.0	d
American sycamore	Platanus	occidentalis	731	0.46	25	0.60	13	81	13	84	6	0.49	25	8.0	23
Arizona sycamore	Platanus	wrightii	732	0.46	d	0.60	d	81	d	84	d	0.49	d	8.0	d
Cottonwood and poplar spp.	Populus	spp.	740	0.35	b	0.46	b	106	b	88	b	0.39	b	18.4	f
Balsam poplar	Populus	balsamifera	741	0.31	25	0.50	23	107	13	86	1	0.34	25	22.0	f
Eastern cottonwood	Populus	deltoides	742	0.37	25	0.38	13	117	29	56	e	0.40	25	22.0	30
Bigtooth aspen	Populus	grandidentata	743	0.36	25	0.59	10	91	13	90	e	0.39	25	14.4	f
Swamp cottonwood	Populus	heterophylla	744	0.35	a	0.46	a	106	a	88	a	0.39	a	18.4	a
Plains cottonwood	Populus	deltoides	745	0.35	a	0.46	a	106	a	88	a	0.39	a	18.4	a
Quaking aspen	Populus	tremuloides	746	0.35	25	0.50	13	129	29	102	22	0.38	25	14.4	21
Black cottonwood	Populus	balsamifera	747	0.31	25	0.40	13	138	13	100	30	0.35	25	16.3	21
Fremont cottonwood	Populus	fremontii	748	0.41	30	0.41	30	56	e	92	e	0.45	f	22.0	f
Narrowleaf cottonwood	Populus	angustifolia	749	0.35	a	0.46	a	106	a	88	a	0.39	a	18.4	a
Silver poplar	Populus	alba	752	0.35	a	0.46	a	106	a	88	a	0.39	a	18.4	a
Lombardy poplar	Populus	nigra	753	0.35	a	0.46	a	106	a	88	a	0.39	a	18.4	a

Table 4.—continued

Common name	Genus	Species	FIA Code	Wood Specific gravity (green volume basis dry weight)	Reference	Bark Specific gravity (green volume basis dry weight)	Reference	Avg. moisture content of wood as a % of oven-dry weight	Reference	Avg. moisture content of bark as a % of oven-dry weight	Reference	Wood Specific gravity (12 pct MC volume basis dry weight)	Reference	Bark volume %	Reference
Mesquite spp.	Prosopis	spp.	755	0.78	31	0.65	e	21	e	41	e	0.82	1	15.0	f
Honey mesquite	Prosopis	glandulosa	756	0.78	a	0.65	a	21	a	41	a	0.82	a	15.0	a
Velvet mesquite	Prosopis	velutina	757	0.78	a	0.65	a	21	a	41	a	0.82	a	15.0	a
Screwbean mesquite	Prosopis	pubescens	758	0.78	a	0.65	a	21	a	41	a	0.82	a	15.0	a
Cherry and plum spp.	Prunus	spp.	760	0.47	b	0.63	b	53	b	91	b	0.50	b	9.2	f
Pin cherry	Prunus	pensylvanica	761	0.47	a	0.63	a	53	a	91	a	0.50	a	9.2	a
Black cherry	Prunus	serotina	762	0.47	25	0.63	10	53	29	91	e	0.50	25	9.2	11
Chokecherry	Prunus	virginiana	763	0.47	a	0.63	a	53	a	91	a	0.50	a	9.2	a
Peach	Prunus	persica	764	0.47	a	0.63	a	53	a	91	a	0.50	a	9.2	a
Canada plum	Prunus	nigra	765	0.47	a	0.63	a	53	a	91	a	0.50	a	9.2	a
American plum	Prunus	americana	766	0.47	a	0.63	a	53	a	91	a	0.50	a	9.2	a
Bitter cherry	Prunus	emarginata	768	0.47	a	0.63	a	53	a	91	a	0.50	a	9.2	a
Allegheny plum	Prunus	alleghaniensis	769	0.47	a	0.63	a	53	a	91	a	0.50	a	9.2	a
Chickasaw plum	Prunus	angustifolia	770	0.47	a	0.63	a	53	a	91	a	0.50	a	9.2	a
Sweet cherry, domesticated	Prunus	avium	771	0.47	a	0.63	a	53	a	91	a	0.50	a	9.2	a
Sour cherry, domesticated	Prunus	cerasus	772	0.47	a	0.63	a	53	a	91	a	0.50	a	9.2	a
European plum, domesticated	Prunus	domestica	773	0.47	a	0.63	a	53	a	91	a	0.50	a	9.2	a
Mahaleb cherry, domesticated	Prunus	mahaleb	774	0.47	a	0.63	a	53	a	91	a	0.50	a	9.2	a
Oak spp	Quercus	spp.	800	0.59	b	0.58	b	75	b	83	b	0.66	b	19.1	f
California live oak	Quercus	agrifolia	801	0.59	a	0.58	a	75	a	83	a	0.66	a	19.1	a
White oak	Quercus	alba	802	0.60	25	0.56	13	68	13	89	17	0.68	25	16.0	5
Arizona white oak	Quercus	arizonica	803	0.59	a	0.58	a	75	a	83	a	0.66	a	19.1	a
Swamp white oak	Quercus	bicolor	804	0.64	25	0.55	e	58	13	89	e	0.72	25	16.0	f
Canyon live oak	Quercus	chrysolepis	805	0.70	30	0.64	14	74	13	90	e	0.74	f	16.0	f
Scarlet oak	Quercus	coccinea	806	0.60	25	0.71	10	71	13	49	6	0.67	25	22.0	f
Blue oak	Quercus	douglasii	807	0.59	a	0.58	a	75	a	83	a	0.66	a	19.1	a
Durand oak	Quercus	sinuata	808	0.59	a	0.58	a	75	a	83	a	0.66	a	19.1	a
Northern pin oak	Quercus	ellipsoidalis	809	0.59	a	0.58	a	75	a	83	a	0.66	a	19.1	a
Emory oak	Quercus	emoryi	810	0.59	a	0.58	a	75	a	83	a	0.66	a	19.1	a
Engelmann oak	Quercus	engelmannii	811	0.59	a	0.58	a	75	a	83	a	0.66	a	19.1	a

Table 4.—continued

Common name	Genus	Species	FIA Code	Wood Specific gravity (green volume basis dry weight)	Reference	Bark Specific gravity (green volume basis dry weight)	Reference	Avg. moisture content of wood as a % of oven-dry weight	Reference	Avg. moisture content of bark as a % of oven-dry weight	Reference	Wood Specific gravity (12 pct MC volume basis dry weight)	Reference	Bark volume %	Reference
Southern red oak	Quercus	falcata	812	0.52	25	0.68	10	97	13	48	6	0.59	25	22.0	5
Cherrybark oak	Quercus	pagoda	813	0.61	25	0.63	14	68	13	91	17	0.69	25	22.0	f
Gambel oak	Quercus	gambelii	814	0.61	3	0.63	e	66	13	66	e	0.63	f	22.0	f
Oregon white oak	Quercus	garryana	815	0.64	1	0.63	30	58	13	65	e	0.72	1	16.0	f
Scrub oak	Quercus	ilicifolia	816	0.59	a	0.58	a	75	a	83	a	0.66	a	19.1	a
Shingle oak	Quercus	imbricaria	817	0.59	a	0.58	a	75	a	83	a	0.66	a	19.1	a
California black oak	Quercus	kelloggii	818	0.51	18	0.45	14	101	13	89	e	0.55	f	22.0	f
Turkey oak	Quercus	laevis	819	0.59	a	0.58	a	75	a	83	a	0.66	a	19.1	a
Laurel oak	Quercus	laurifolia	820	0.56	25	0.50	e	83	13	121	5	0.63	25	16.0	f
California white oak	Quercus	lobata	821	0.55	30	0.55	30	84	13	89	e	0.58	f	16.0	f
Overcup oak	Quercus	lyrata	822	0.57	25	0.51	14	77	13	89	e	0.63	25	22.0	f
Bur oak	Quercus	macrocarpa	823	0.58	25	0.54	10	74	13	90	e	0.64	25	16.0	f
Blackjack oak	Quercus	marilandica	824	0.59	a	0.58	a	75	a	83	a	0.66	a	19.1	a
Swamp chestnut oak	Quercus	michauxii	825	0.60	25	0.51	14	68	13	89	e	0.67	25	23.0	f
Chinkapin oak	Quercus	muehlenbergii	826	0.59	a	0.58	a	75	a	83	a	0.66	a	19.1	a
Water oak	Quercus	nigra	827	0.56	25	0.62	14	83	13	73	5	0.63	25	16.0	f
Texas red oak	Quercus	texana	828	0.59	a	0.58	a	75	a	83	a	0.66	a	19.1	a
Mexican blue oak	Quercus	oblongifolia	829	0.59	a	0.58	a	75	a	83	a	0.66	a	19.1	a
Pin oak	Quercus	palustris	830	0.58	25	0.60	14	77	13	90	17	0.63	25	22.0	f
Willow oak	Quercus	phellos	831	0.56	25	0.59	10	83	13	90	e	0.69	25	16.0	f
Chestnut oak	Quercus	prinus	832	0.57	25	0.54	10	77	13	60	6	0.66	25	23.0	5
Northern red oak	Quercus	rubra	833	0.56	25	0.68	13	83	13	91	17	0.63	25	20.0	9
Shumard oak	Quercus	shumardii	834	0.59	a	0.58	a	75	a	83	a	0.66	a	19.1	a
Post oak	Quercus	stellata	835	0.60	25	0.51	10	71	13	89	17	0.67	25	22.0	f
Delta post oak	Quercus	similis	836	0.59	a	0.58	a	75	a	83	a	0.66	a	19.1	a
Black oak	Quercus	velutina	837	0.56	25	0.60	10	83	13	90	17	0.61	25	18.5	11
Live oak	Quercus	virginiana	838	0.80	25	0.51	14	52	13	89	e	0.88	25	16.0	f
Interior live oak	Quercus	wislizeni	839	0.59	a	0.58	a	75	a	83	a	0.66	a	19.1	a
Dwarf post oak	Quercus	margarettiae	840	0.59	a	0.58	a	75	a	83	a	0.66	a	19.1	a
Dwarf live oak	Quercus	minima	841	0.59	a	0.58	a	75	a	83	a	0.66	a	19.1	a

Table 4.—continued

Common name	Genus	Species	FIA Code	Wood Specific gravity (green volume basis dry weight)	Reference	Bark Specific gravity (green volume basis dry weight)	Reference	Avg. moisture content of wood as a % of oven-dry weight	Reference	Avg. moisture content of bark as a % of oven-dry weight	Reference	Wood Specific gravity (12 pct MC volume basis dry weight)	Reference	Bark volume %	Reference
Bluejack oak	Quercus	incana	842	0.59	a	0.58	a	75	a	83	a	0.66	a	19.1	a
Silverleaf oak	Quercus	hypoleucoides	843	0.59	a	0.58	a	75	a	83	a	0.66	a	19.1	a
Oglethorpe oak	Quercus	oglethorpensis	844	0.59	a	0.58	a	75	a	83	a	0.66	a	19.1	a
Dwarf chinkapin oak	Quercus	prinoides	845	0.59	a	0.58	a	75	a	83	a	0.66	a	19.1	a
Gray oak	Quercus	grisea	846	0.59	a	0.58	a	75	a	83	a	0.66	a	19.1	a
Netleaf oak	Quercus	rugosa	847	0.59	a	0.58	a	75	a	83	a	0.66	a	19.1	a
Chisos oak	Quercus	graciliformis	851	0.59	a	0.58	a	75	a	83	a	0.66	a	19.1	a
Sea torchwood	Amyris	elemifera	852	0.52	c	0.53	c	75	c	80	c	0.58	c	15.2	c
Pond-apple	Annona	glabra	853	0.52	c	0.53	c	75	c	80	c	0.58	c	15.2	c
Gumbo limbo	Bursera	simaruba	854	0.52	c	0.53	c	75	c	80	c	0.58	c	15.2	c
Sheoak spp.	Casuarina	spp.	855	0.52	c	0.53	c	75	c	80	c	0.58	c	15.2	c
Gray sheoak	Casuarina	glauca	856	0.52	c	0.53	c	75	c	80	c	0.58	c	15.2	c
Belah	Casuarina	lepidophloia	857	0.52	c	0.53	c	75	c	80	c	0.58	c	15.2	c
Camphortree	Cinnamomum	camphora	858	0.52	c	0.53	c	75	c	80	c	0.58	c	15.2	c
Florida fiddlewood	Citharexylum	fruticosum	859	0.52	c	0.53	c	75	c	80	c	0.58	c	15.2	c
Citrus spp.	Citrus	spp.	860	0.52	c	0.53	c	75	c	80	c	0.58	c	15.2	c
Tietongue, pigeon-plum	Coccoloba	diversifolia	863	0.52	c	0.53	c	75	c	80	c	0.58	c	15.2	c
Soldierwood	Colubrina	elliptica	864	0.52	c	0.53	c	75	c	80	c	0.58	c	15.2	c
Largeleaf geigertree	Cordia	sebestena	865	0.52	c	0.53	c	75	c	80	c	0.58	c	15.2	c
Carrotwood	Cupaniopsis	anacardioides	866	0.52	c	0.53	c	75	c	80	c	0.58	c	15.2	c
Bluewood	Condalia	hookeri	867	0.52	c	0.53	c	75	c	80	c	0.58	c	15.2	c
Blackbead ebony	Ebenopsis	ebano	868	0.52	c	0.53	c	75	c	80	c	0.58	c	15.2	c
Great leucaene	Leucaena	pulverulenta	869	0.52	c	0.53	c	75	c	80	c	0.58	c	15.2	c
Texas sophora	Sophora	affinis	870	0.52	c	0.53	c	75	c	80	c	0.58	c	15.2	c
Red stopper	Eugenia	rhombea	873	0.52	c	0.53	c	75	c	80	c	0.58	c	15.2	c
Butterbough, inkwood	Exothea	paniculata	874	0.52	c	0.53	c	75	c	80	c	0.58	c	15.2	c
Florida strangler fig	Ficus	aurea	876	0.52	c	0.53	c	75	c	80	c	0.58	c	15.2	c
Wild banyantree, shortleaf fig	Ficus	citrifolia	877	0.52	c	0.53	c	75	c	80	c	0.58	c	15.2	c
Beeftree, longleaf blolly	Guapira	discolor	882	0.52	c	0.53	c	75	c	80	c	0.58	c	15.2	c
Manchineel	Hippomane	mancinella	883	0.52	c	0.53	c	75	c	80	c	0.58	c	15.2	c

Table 4.—continued

Common name	Genus	Species	FIA Code	Wood Specific gravity (green volume basis dry weight)	Reference	Bark Specific gravity (green volume basis dry weight)	Reference	Avg. moisture content of wood as a % of oven-dry weight	Reference	Avg. moisture content of bark as a % of oven-dry weight	Reference	Wood Specific gravity (12 pct MC volume basis dry weight)	Reference	Bark volume %	Reference
False tamarind	Lysiloma	latisiliquum	884	0.52	c	0.53	c	75	c	80	c	0.58	c	15.2	c
Mango	Mangifera	indica	885	0.52	c	0.53	c	75	c	80	c	0.58	c	15.2	c
Florida poisontree	Metopium	toxiferum	886	0.52	c	0.53	c	75	c	80	c	0.58	c	15.2	c
Fishpoison tree	Piscidia	piscipula	887	0.52	c	0.53	c	75	c	80	c	0.58	c	15.2	c
Octopus tree, schefflera	Schefflera	actinophylla	888	0.52	c	0.53	c	75	c	80	c	0.58	c	15.2	c
False mastic	Sideroxylon	foetidissimum	890	0.52	c	0.53	c	75	c	80	c	0.58	c	15.2	c
White bully, willow bustic	Sideroxylon	salicifolium	891	0.52	c	0.53	c	75	c	80	c	0.58	c	15.2	c
Paradisetree	Simarouba	glauca	895	0.52	c	0.53	c	75	c	80	c	0.58	c	15.2	c
Java plum	Syzygium	cumini	896	0.52	c	0.53	c	75	c	80	c	0.58	c	15.2	c
Tamarind	Tamarindus	indica	897	0.52	c	0.53	c	75	c	80	c	0.58	c	15.2	c
Black locust	Robinia	pseudoacacia	901	0.66	25	0.29	10	41	26	88	e	0.69	25	15.0	f
New Mexico locust	Robinia	neomexicana	902	0.66	d	0.29	d	41	d	88	d	0.69	d	15.0	d
Everglades palm, paurotis-palm	Acoelorraphe	wrightii	906	0.52	c	0.53	c	75	c	80	c	0.58	c	15.2	c
Florida silver palm	Coccothrinax	argentata	907	0.52	c	0.53	c	75	c	80	c	0.58	c	15.2	c
Coconut palm	Cocos	nucifera	908	0.52	c	0.53	c	75	c	80	c	0.58	c	15.2	c
Royal palm spp.	Roystonea	spp.	909	0.52	c	0.53	c	75	c	80	c	0.58	c	15.2	c
Mexican palmetto	Sabal	mexicana	911	0.52	c	0.53	c	75	c	80	c	0.58	c	15.2	c
Cabbage palmetto	Sabal	palmetto	912	0.52	c	0.53	c	75	c	80	c	0.58	c	15.2	c
Key thatch palm	Thrinax	morrisii	913	0.52	c	0.53	c	75	c	80	c	0.58	c	15.2	c
Florida thatch palm	Thrinax	radiata	914	0.52	c	0.53	c	75	c	80	c	0.58	c	15.2	c
Other palms	Family Arecaceae	not listed above	915	0.52	c	0.53	c	75	c	80	c	0.58	c	15.2	c
Western soapberry	Sapindus	saponaria	919	0.52	c	0.53	c	75	c	80	c	0.58	c	15.2	c
Willow spp.	Salix	spp.	920	0.36	b	0.50	b	127	b	99	b	0.39	b	16.0	23
Peachleaf willow	Salix	amygdaloides	921	0.36	a	0.50	a	127	a	99	a	0.39	a	16.0	a
Black willow	Salix	nigra	922	0.36	25	0.50	14	127	13	99	1	0.39	25	16.0	f
Bebb willow	Salix	bebbiana	923	0.36	a	0.50	a	127	a	99	a	0.39	a	16.0	a
Bonpland willow	Salix	bonplandiana	924	0.36	a	0.50	a	127	a	99	a	0.39	a	16.0	a
Coastal plain willow	Salix	caroliniana	925	0.36	a	0.50	a	127	a	99	a	0.39	a	16.0	a
Balsam willow	Salix	pyrifolia	926	0.36	a	0.50	a	127	a	99	a	0.39	a	16.0	a
White willow	Salix	alba	927	0.36	a	0.50	a	127	a	99	a	0.39	a	16.0	a

Table 4.—continued

Common name	Genus	Species	FIA Code	Wood Specific gravity (green volume basis dry weight)	Reference	Bark Specific gravity (green volume basis dry weight)	Reference	Avg. moisture content of wood as a % of oven-dry weight	Reference	Avg. moisture content of bark as a % of oven-dry weight	Reference	Wood Specific gravity (12 pct MC volume basis dry weight)	Reference	Bark volume %	Reference
Scouler's willow	Salix	scouleriana	928	0.36	a	0.50	a	127	a	99	a	0.39	a	16.0	a
Weeping willow	Salix	sepulcralis	929	0.36	a	0.50	a	127	a	99	a	0.39	a	16.0	a
Sassafras	Sassafras	albidum	931	0.42	25	0.50	14	68	26	89	e	0.46	25	15.0	f
Mountain-ash spp.	Sorbus	spp.	934	0.52	c	0.53	c	75	c	80	c	0.58	c	15.2	c
American mountain-ash	Sorbus	americana	935	0.52	c	0.53	c	75	c	80	c	0.58	c	15.2	c
European mountain-ash	Sorbus	aucuparia	936	0.52	c	0.53	c	75	c	80	c	0.58	c	15.2	c
Northern mountain-ash	Sorbus	decora	937	0.52	c	0.53	c	75	c	80	c	0.58	c	15.2	c
West Indian mahogany	Swietenia	mahagoni	940	0.52	c	0.53	c	75	c	80	c	0.58	c	15.2	c
Basswood spp.	Tilia	spp.	950	0.32	b	0.48	b	105	b	90	b	0.37	b	10.5	f
American basswood	Tilia	americana	951	0.32	25	0.48	10	105	25	90	e	0.37	25	10.5	8
White basswood	Tilia	americana	952	0.32	a	0.48	a	105	a	90	a	0.37	a	10.5	a
Carolina basswood	Tilia	americana	953	0.32	a	0.48	a	105	a	90	a	0.37	a	10.5	a
Elm spp.	Ulmus	spp.	970	0.54	b	0.43	b	66	b	91	6	0.59	b	14.0	23
Winged elm	Ulmus	alata	971	0.60	1	0.45	e	42	e	75	e	0.66	1	14.0	f
American elm	Ulmus	americana	972	0.46	25	0.44	10	94	25	78	e	0.50	25	14.0	f
Cedar elm	Ulmus	crassifolia	973	0.59	1	0.45	e	66	25	75	e	0.64	1	14.0	f
Siberian elm	Ulmus	pumila	974	0.54	a	0.43	a	66	a	91	a	0.59	a	14.0	a
Slippery elm	Ulmus	rubra	975	0.48	25	0.29	10	77	e	171	e	0.53	25	14.0	f
September elm	Ulmus	serotina	976	0.54	a	0.43	a	66	a	91	a	0.59	a	14.0	a
Rock elm	Ulmus	thomasii	977	0.57	25	0.50	14	51	25	57	e	0.63	25	14.0	f
California-laurel	Umbellularia	californica	981	0.51	1	0.55	30	67	30	43	e	0.55	1	15.0	f
Joshua tree	Yucca	brevifolia	982	0.52	c	0.53	c	75	c	80	c	0.58	c	15.2	c
Black-mangrove	Avicennia	germinans	986	0.52	c	0.53	c	75	c	80	c	0.58	c	15.2	c
Buttonwood-mangrove	Conocarpus	erectus	987	0.52	c	0.53	c	75	c	80	c	0.58	c	15.2	c
White-mangrove	Laguncularia	racemosa	988	0.52	c	0.53	c	75	c	80	c	0.58	c	15.2	c
American mangrove	Rhizophora	mangle	989	0.52	c	0.53	c	75	c	80	c	0.58	c	15.2	c
Desert ironwood	Olneya	tesota	990	0.52	c	0.53	c	75	c	80	c	0.58	c	15.2	c
Saltcedar	Tamarix	spp.	991	0.52	c	0.53	c	75	c	80	c	0.58	c	15.2	c
Melaleuca	Melaleuca	quinquenervia	992	0.52	c	0.53	c	75	c	80	c	0.58	c	15.2	c
Chinaberry	Melia	azedarach	993	0.52	c	0.53	c	75	c	80	c	0.58	c	15.2	c

Table 4.—continued

Common name	Genus	Species	FIA Code	Wood Specific gravity (green volume basis dry weight)	Reference	Bark Specific gravity (green volume basis dry weight)	Reference	Avg. moisture content of wood as a % of oven-dry weight	Reference	Avg. moisture content of bark as a % of oven-dry weight	Reference	Wood Specific gravity (12 pct MC volume basis dry weight)	Reference	Bark volume %	Reference
Chinese tallowtree	Triadica	sebifera	994	0.52	c	0.53	c	75	c	80	c	0.58	c	15.2	c
Tungoil tree	Vernicia	fordii	995	0.52	c	0.53	c	75	c	80	c	0.58	c	15.2	c
Smoketree	Cotinus	obovatus	996	0.52	c	0.53	c	75	c	80	c	0.58	c	15.2	c
Russian-olive	Elaeagnus	angustifolia	997	0.52	c	0.53	c	75	c	80	c	0.58	c	15.2	c
Unknown dead hardwood	Tree	broadleaf	998	0.52	c	0.53	c	75	c	80	c	0.58	c	15.2	c
Other or unknown live tree	Tree	unknown	999	0.52	c	0.53	c	75	c	80	c	0.58	c	15.2	c
Washington hawthorn	Crataegus	phaenopyrum	5091	0.52	c	0.53	c	75	c	80	c	0.58	c	15.2	c
Fleshy hawthorn	Crataegus	succulenta	5092	0.52	c	0.53	c	75	c	80	c	0.58	c	15.2	c
Dwarf hawthorn	Crataegus	uniflora	5093	0.52	c	0.53	c	75	c	80	c	0.58	c	15.2	c
Berlandier ash	Fraxinus	berlandieriana	5491	0.51	a	0.46	a	61	a	86	a	0.55	a	16.0	a
Avocado	Persea	americana	7211	0.52	c	0.53	c	75	c	80	c	0.58	c	15.2	c
Graves oak	Quercus	gravesii	8511	0.59	a	0.58	a	75	a	83	a	0.66	a	19.1	a
Mexican white oak	Quercus	polymorpha	8512	0.59	a	0.58	a	75	a	83	a	0.66	a	19.1	a
Buckley oak	Quercus	buckleyi	8513	0.59	a	0.58	a	75	a	83	a	0.66	a	19.1	a
Lacey oak	Quercus	laceyi	8514	0.59	a	0.58	a	75	a	83	a	0.66	a	19.1	a
Anacahuita Texas Olive	Cordia	boissieri	8651	0.52	c	0.53	c	75	c	80	c	0.58	c	15.2	c

a Assigned average value of the trees of the same genus in Table 1A

b Assigned genus value from Table 5

c Assigned the average value of softwood trees or average value of hardwood trees from Table 1A

d Assigned value of the tree of the same genus in Table 1A

e Based on green volume specific gravity and bark moisture content of similar species

f No reference source available, estimated based on similar species

Corrected Table 5.—Average specific gravity, dry weight, green weight, and moisture content for tree Genus groups found in North America. (The values in the columns with unit measurements lb/cf and kg/m3 were corrected on Feb. 10, 2010.)

			Specific gravity and oven-dry weight of wood						Average mositure content (MC) and green weight of wood *			Specific gravity and oven-dry weight of bark			Average mositure content (MC) and green weight of bark *			Bark volume
			12% MC volume basis			Green volume basis						Green volume basis						
Common name	Genus	No. of species	Average specific gravity	Avg. oven-dry weight (lb/cf)	Avg. oven-dry weight (kg/m3)	Average specific gravity	Avg. oven-dry weight (lb/cf)	Avg. oven-dry weight (kg/m3)	Avg. moisture content as a % of oven-dry weight	Avg. green wt. (lb/cf)	Avg. green wt. (kg/m3)	Average specific gravity	Avg. oven-dry weight (lb/cf)	Avg. oven-dry weight (kg/m3)	Avg. MC as a % of oven-dry weight	Avg. green wt. (lb/m3)	Avg. green wt. (kg/m3)	Avg. bark volume as % of wood volume
Fir	*Abies*	7	0.38	23.4	376	0.36	22.2	356	83.79	40.8	653	0.49	30.3	485	62.51	49.1	787	11.76
Cedar	*Chamaecyparis*	3	0.40	24.8	396	0.37	23.3	373	77.27	41.1	659	0.40	25.0	400	92.32	48.0	769	11.58
Juniper	*Juniperus*	4	0.54	33.4	535	0.51	31.5	505	36.11	42.9	687	0.40	25.0	400	60.25	40.0	641	12.00
Larch	*Larix*	2	0.53	32.8	525	0.49	30.3	485	56.98	47.5	761	0.32	19.7	315	81.38	35.5	569	14.00
Spruce	*Picea*	5	0.39	24.6	394	0.36	22.2	356	59.22	35.2	564	0.44	27.3	438	80.51	49.4	791	12.55
Pine	*Pinus*	24	0.47	29.3	469	0.43	26.8	429	76.22	46.8	749	0.40	25.0	401	68.37	41.9	671	16.13
Aborvitae	*Thuja*	2	0.32	19.7	315	0.30	18.7	300	69.26	31.5	505	0.40	24.6	395	73.35	43.0	689	12.28
Hemlock	*Tsuga*	3	0.43	27.0	433	0.41	25.4	406	78.40	45.0	721	0.46	28.5	456	97.35	56.3	902	16.18
Maple	*Acer*	7	0.52	32.2	515	0.47	29.5	473	70.16	50.0	801	0.53	33.3	533	90.54	63.1	1,011	10.81
Birch	*Betula*	5	0.58	36.1	578	0.51	32.1	514	73.75	55.8	893	0.58	36.2	580	54.66	56.0	897	10.94
Hickory	*Carya*	8	0.68	42.3	677	0.62	38.5	616	68.93	64.9	1039	0.62	38.4	615	56.91	60.0	961	16.00
Dogwood	*Cornus*	2	0.68	42.1	675	0.61	38.1	610	39.58	53.0	849	0.58	36.2	580	90.66	69.0	1,105	15.00
Ash	*Fraxinus*	6	0.55	34.3	550	0.51	31.6	506	61.08	50.7	812	0.46	28.6	458	86.05	53.2	852	16.00
Walnut	*Juglans*	2	0.47	29.0	465	0.44	27.1	435	91.95	51.5	825	0.37	22.8	365	88.85	43.0	689	15.00
Magnolia	*Magnolia*	4	0.47	29.3	470	0.43	26.8	430	91.83	51.5	825	0.44	27.5	440	93.03	53.0	849	15.00
Tupelo	*Nyssa*	2	0.50	31.2	500	0.46	28.7	460	98.04	56.8	911	0.51	31.8	510	76.77	56.5	905	14.00
Poplar	*Populus*	6	0.39	24.0	385	0.35	21.9	352	106.31	44.8	718	0.46	28.9	463	87.64	54.5	873	18.52
Oak	*Quercus*	22	0.66	41.2	661	0.59	37.1	594	75.30	64.7	1036	0.58	36.1	579	82.95	65.7	1,053	19.12
Elm	*Ulmus*	5	0.59	36.9	592	0.54	33.7	540	65.84	55.3	886	0.43	26.6	426	91.07	49.0	785	14.00
Softwood		56	0.44	27.8	445	0.41	25.7	411	74.28	44.1	707	0.42	26.0	417	70.51	44.5	712	14.47
Hardwood		100	0.58	36.0	577	0.52	32.6	523	75.12	56.1	899	0.53	32.9	527	80.64	58.8	942	15.32

* Moisture content is extremely variable and the values shown are averages or estimates based on the literature cited.

Miles, Patrick D.; Smith, W. Brad. 2009. **Specific gravity and other properties of wood and bark for 156 tree species found in North America.** Res. Note NRS-38. Newtown Square, PA: U.S. Department of Agriculture, Forest Service, Northern Research Station. 35 p.

This paper reports information for the estimation of biomass for 156 tree species found in North America for use in national forest inventory applications. We present specific gravities based on average green volume as well as 12 percent moisture content volume for calculation of oven-dry biomass. Additional information is included on bark thickness, bark voids, and bark percentages by species and green and dry weight of wood and bark.

KEY WORDS: bark percentage, bark thickness, biomass, tree volume, dry weight, green weight

The U.S. Department of Agriculture (USDA) prohibits discrimination in all its programs and activities on the basis of race, color, national origin, age, disability, and where applicable, sex, marital status, familial status, parental status, religion, sexual orientation, genetic information, political beliefs, reprisal, or because all or part of an individual's income is derived from any public assistance program. (Not all prohibited bases apply to all programs.) Persons with disabilities who require alternate means for communication of program information (Braille, large print, audiotape, etc.) should contact USDA's TARGET Center at (202)720-2600 (voice and TDD). To file a complaint of discrimination, write to USDA, Director, Office of Civil Rights, 1400 Independence Avenue, S.W., Washington, DC 20250-9410, or call (800)795-3272 (voice) or (202)720-6382 (TDD). USDA is an equal opportunity provider and employer.

Printed on Recycled Paper

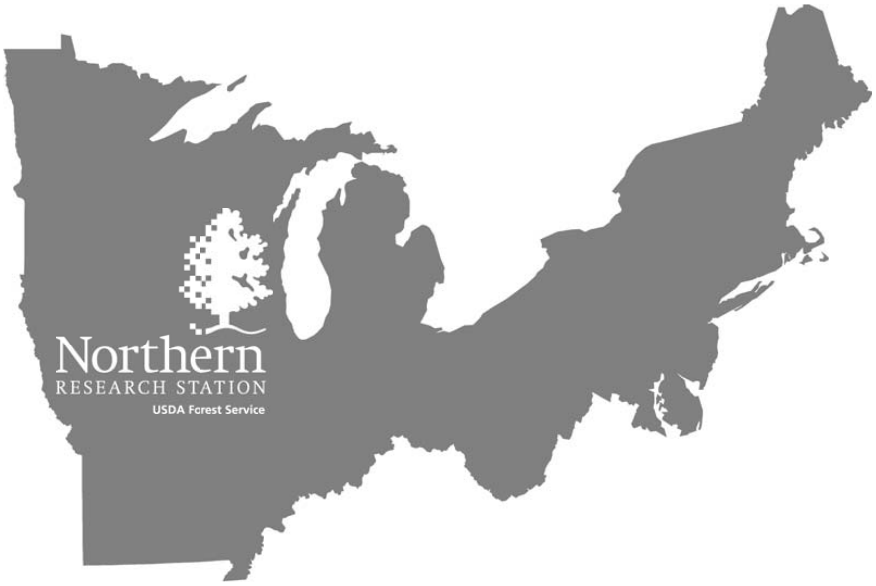

Capitalizing on the strengths of existing science capacity in the Northeast and Midwest to attain a more integrated, cohesive, landscape-scale research program

www.nrs.fs.fed.us
